# Supplementary material for: Ethiopia National Food and Nutrition Survey to inform the Ethiopian National Food and Nutrition Strategy: a study protocol
Source: BMJ Open. 2023 Apr 25;13(4):e067641. doi: 10.1136/bmjopen-2022-067641 (PMC10151871; doi:10.1136/bmjopen-2022-067641)
Supplement: Supplementary data [file bmjopen-2022-067641supp014.pdf]

Table 1S. Key indicators used to estimate sample size for each target group

Target Group	Key indicators used to estimate sample size
Children under 5 years of age (0-59 months)	Vitamin A deficiency<br>Total goiter prevalence<br>Stunting<br>Any anemia<br>Zinc deficiency<br>Prevalence of inadequate intake of zinc
Women of reproductive age (15-49 years)	Vitamin A deficiency<br>Total goiter prevalence<br>Any anemia<br>Zinc deficiency<br>RBC folate deficiency<br>Serum folate deficiency<br>Vitamin B_{12} deficiency<br>Iodized salt coverage<br>Prevalence of inadequate intake of iron<br>Prevalence of inadequate intake of zinc<br>Prevalence of inadequate intake vitamin A
School age children (6 to 12 years)	Vitamin A deficiency<br>Total goiter prevalence<br>Any anemia<br>Iodine deficiency<br>Zinc deficiency
Adolescent girls (10 to 19 years)	Any anemia
