# Supplementary material for: Ethiopia National Food and Nutrition Survey to inform the Ethiopian National Food and Nutrition Strategy: a study protocol
Source: BMJ Open. 2023 Apr 25;13(4):e067641. doi: 10.1136/bmjopen-2022-067641 (PMC10151871; doi:10.1136/bmjopen-2022-067641)
Supplement: Supplementary data [file bmjopen-2022-067641supp015.pdf]

Table 2S. Sample size determination and allocation

Region	Indicator used to estimate sample size	Number of EA	Number of HH	Expected number pre-school children (0-59 months)	Expected number of WRA (15-49 years)	Expected number school age children (6-12 years)	Expected number of adolescent girls (10-19 years)	Total population (2019 year projection)
Tigray	Any anemia	55	1,432	590	1,236	753	516	5,443,095
Afar	Stunting	51	1,328	539	1,096	695	406	1,901,863
Amhara	Stunting	61	1,585	619	1,253	843	531	21,842,548
Oromia	IDD	62	1,622	891	1,539	1,111	739	37,267,225
Somali	IDD	55	1,424	855	1,268	1,000	492	6,050,851
Benishangul-Gumuz	Stunting	49	1,282	555	1,127	732	475	1,126,656
SNNPR	Any anemia	59	1,528	818	1,492	1,000	692	15,763,484
Gambela	Any anemia	47	1,211	428	1,018	568	373	463,203
Harari	Any anemia	45	1,164	375	978	499	348	257,362
Addis Ababa	IDD/ TGR	54	1,411	413	1,274	405	262	3,685,684
Dire Dawa	Stunting	47	1,215	382	1,128	480	360	492,819
Sidama	Any anemia	54	1,395	747	1,363	914	632	4,322,685
Total sample size		639	16,596	7,213	14,772	9,001	5,824	**98,617,475**
