# Supplementary material for: Ethiopia National Food and Nutrition Survey to inform the Ethiopian National Food and Nutrition Strategy: a study protocol
Source: BMJ Open. 2023 Apr 25;13(4):e067641. doi: 10.1136/bmjopen-2022-067641 (PMC10151871; doi:10.1136/bmjopen-2022-067641)
Supplement: Supplementary data [file bmjopen-2022-067641supp001.pdf]

	Name of institute<br><br>**ETHIOPIAN PUBLIC HEALTH INSTITUTE**	Doc. number:<br><br>EPHI/ TL/ FNL/TM/7.2.11	Version No: 3<br><br>Copy no_____<br><br>Page 1 of 7
Document Title: **DETERMIANTION OF IODINE FROM SALT SAMPLE**		Effective date: November 20, 2020	

1. **Purpose**

This procedure used to determine the amount of iodine content in the form of potassium iodate (KIO_3) by iodometric titration from iodized salt.

2. **Abbreviations:**

**g**	gram	**KI**	Potassium Iodide
**ppm**	Parts Per million	**H_2SO_4**	sulfuric acid
**M**	Molarity	**KIO_3**	potassium iodate
**ml**	mili liter	**IDD**	Iodine deficiency disorder
**N**	Normality	**$Na_2S_2O_3.5H_2O$**	sodium thiosulfate penta hydrate

3. **Principle:**

Iodine released from potassium iodate by the action of sulphuric acid and the released iodine trapped with potassium iodide and titrate with sodium thiosulphate.

4. **Material and methods**

4.1 Reagents

- 0.005M $Na_2S_2O_3.5H_2O$
- 2N H_2SO_4
- 10% KI
- 1% Starch

4.2 Reagents preparation:

- **1%starch:** Dissolve 1 g of soluble starch in 100ml boiled distilled water heat the solution till starch dissolve completely.
- **10% KI;** Dissolve 10gm of KI in 100ml deionized water**.**

Prepared by: Addisu Legesse and Teshome Assefa	Authority: EPHI

NOTE: This is a CONTROLLED document for internal use only. Any documents appearing in paper form are not controlled and should be checked against the electronic version prior to use.

- **2N H_2SO_4;** Slowly add 6 ml of concentrated H_2SO_4 to 90 ml of deionized water make the final volume 100 ml.
- **0.005M $Na_2S_2O_3$;** Dissolve 1.24gm of $Na_2S_2O_3.5H_2O$ in 1000 ml of deionized water

4.3 Reagents stability and storage:

- $Na_2S_2O3.5H_2O$ & 10% KI reagents store in a cool & dark place for six months.
- H_2SO_4 store at room temperature it stable indefinitely.
- Starch should be prepared daily.

5. Supplies and Equipment

- Balance (Four-beam pan): Sensitivity = 0.01g, Capacity = 410g
- Flask, volumetric, 1000mL, 100mL
- Measuring cylinder, 10mL, 100mL
- Beakers (Pyrex)
- Flasks, Erlenmeyer (conical) with stopper, 250mL
- Pipette, volumetric, 1mL, 5mL
- Burette w/straight stopcock 10mL
- Burette stand
- Laboratory safety glasses
- Parafilm, for covering beakers
- Glass bottles with stoppers, for reagents, 250mL
- Spatula Lab single blade 150mm SS length
- Dropper bottle, glass 25-60mL
- Hot plate

6. Sample

- Sample type: salt
- Amount required: 50-100g
- Transport and storage: At room temperature avoid exposure to direct sunlight

- Stability: At room temperature for 3 months.

7. Special Safety and Precautions

- While titrate the sample wear eye google.
- The reaction mixture should be kept in the dark before titration because a side reaction can occur when the solution is exposed to light that causes iodide ions to be oxidized to iodine.
- Inaccurate results may occur if starch solution is used while still warm.
- If starch indicator is added too early, a strong iodine-starch complex is formed, which reacts slowly, and gives falsely elevated results.
- The reaction should be performed at mild room temperature (<30 ^{0}C), since the iodine is volatile, and the indicator solution loses sensitivity when exposed to high temperatures.

8. Procedure

8.1 Weigh 10g of the salt sample into a 250mL Erlenmeyer flask with a stopper.

8.2 Add approximately 50 mL water, swirl to dissolve salt sample.

8.3 Add 1 ml 2N H_2SO_4

8.4 Add 5mL 10% KI. The solution should turn yellow if iodine is present.

8.5 Close the flask with stopper & put it in the dark place for 10 minutes in closed box (cupboard or drawer).

8.6 Rinse and fill burette with 0.005M $Na_2S_2O_3$, and adjust level to zero.

8.7 After 10 minutes take the flask out from drawer, and add some $Na_2S_2O_3$ from the titration burette Until the solution turns pale yellow.

8.8 Add approximately 2mL of starch indicator solution (the solution should turn dark purple) and continue titrating with 0.005M $Na_2S_2O_3$ until the solution becomes pink, and finally colorless.

8.9 Record the level of thiosulfate in the burette and convert to parts per million (ppm)

9. **Quality control**:
   - **Control material**: KIO_3
   - **Level of iodine**: 59.3-60.3ppm
   - **Stability**: stable at room temperature for long period of time.
   - **Frequency**: per batch

10. **Quality control preparation**: Prepare 0.0047M KIO_3 in 100ml. By weighing 0.10058g in 100ml deionized water. From this solution Pipette out 1 ml into conical flask and follow the procedure proceed from step 2 as described above.

11. **Calculation**

$$\textbf{Iodine}(\text{ppm}) = \mathbf{10.6 * V\ Na_2S_2O_3(ml)}$$

Where: V$Na_2S_2O_3$: Volume of sodium thiosulphate takes to titrate iodine in salt

12. **Result Interpretation**
   - **<5ppm** to indicate salt with no added iodine
   - **5-14.9** ppm to indicate inadequately iodized salt
   - **15-39.9** ppm to indicate salt is adequately iodized
   - **>40** ppm of iodine is not recommended.

13. **Other Records**
   - Data Log sheet
   - QC chart

14. **References**

- De Maeyer, Lowenstein & Thilly, 1979; World Health Organization (WHO), United Nations Children Fund (UNICEF) & ICCIDD, 2007.
- AOAC 925.56 2016

BMJ Publishing Group Limited (BMJ) disclaims all liability and responsibility arising from any reliance placed on this supplemental material which has been supplied by the author(s)

	Name of institute<br><br>**ETHIOPIAN PUBLIC HEALTH INSTITUTE**	Doc. number:<br><br>EPHI/ TL/ FNL/TM/7.2.11	Version No: 3<br><br>Copy no_____<br><br>Page 5 of 7
Document Title: **DETERMIANTION OF IODINE FROM SALT SAMPLE**		Effective date: November 20, 2020	

15. Annex

Conversion chart for iodine in fortified salt in the form of KIO_3 (PPM)

Volume Thiosulphate (mL)	Iodine (ppm)	Volume Thiosulphate (mL)	Iodine (ppm)	Volume Thiosulphate (mL)	Iodine (ppm)	Volume Thiosulphate (mL)	Iodine (ppm)	Volume Thiosulphate (mL)	Iodine (ppm)	Volume Thiosulphate (mL)	Iodine (ppm)
0.1	1.1	2.0	21.2	3.9	41.3	5.8	61.5	7.7	81.6	9.6	101.8
0.2	2.1	2.1	22.2	4.0	42.4	5.9	62.5	7.8	82.7	9.7	102.8
0.3	3.2	2.2	23.3	4.1	43.5	6.0	63.6	7.9	83.4	9.8	103.9
0.4	4.2	2.3	24.4	4.2	44.5	6.1	64.7	8.0	84.8	9.9	104.9
0.5	5.3	2.4	25.4	4.3	45.6	6.2	65.7	8.1	85.9	10.0	106.0
0.6	6.4	2.5	26.5	4.4	46.4	6.3	66.8	8.2	86.9	10.1	107.1
0.7	7.4	2.6	27.6	4.5	47.7	6.4	67.8	8.3	88.0	10.2	108.1
0.8	8.5	2.7	28.6	4.6	48.8	6.5	68.9	8.4	89.0	10.3	109.2
0.9	9.4	2.8	29.7	4.7	49.8	6.6	70.0	8.5	90.1	10.4	110.2
1.0	10.6	2.9	30.7	4.8	50.9	6.7	71.0	8.6	91.2	10.5	111.3
1.1	11.7	3.0	31.8	4.9	51.9	6.8	72.1	8.7	92.2	10.6	112.4
1.2	12.2	3.1	32.9	5.0	53.0	6.9	73.1	8.8	93.3	10.7	113.4
1.3	13.8	3.2	33.9	5.1	54.1	7.0	74.2	8.9	94.3	10.8	114.5
1.4	14.8	3.3	35.0	5.2	55.1	7.1	75.3	9.0	95.4	10.9	115.5
1.5	15.9	3.4	36.0	5.3	56.2	7.2	76.3	9.1	96.5	11.0	116.6
1.6	17.0	3.5	37.1	5.4	57.2	7.3	77.4	9.2	97.5	11.1	117.7
1.7	18.0	3.6	38.2	5.5	58.3	7.4	78.4	9.3	98.6	11.2	118.7
1.8	19.1	3.7	39.2	5.6	59.4	7.5	79.5	9.4	99.7	11.3	119.8
1.9	20.1	3.8	40.3	5.7	60.4	7.6	80.6	9.5	100.7	11.4	120.8

Prepared by: Addisu Legesse and Teshome Assefa	Authority: EPHI

NOTE: This is a CONTROLLED document for internal use only. Any documents appearing in paper form are not controlled and should be checked against the electronic version prior to use.

	Name of institute<br>**ETHIOPIAN PUBLIC HEALTH INSTITUTE**	Doc. number:<br>EPHI/ TL/ FNL/TM/7.2.11	Version No: 3<br>Copy no_____<br>Page 6 of 7
Document Title: **DETERMIANTION OF IODINE FROM SALT SAMPLE**		Effective date: November 20, 2020	

Name Signature

Prepared by: ------------------------- ----------------------------

Reviewed by: ------------------------ ----------------------------

Approved by: ------------------------- -----------------------------

(Director of Food science and Nutrition research directorate)

Declaration

I, the undersigned laboratory personnel, certify that I am conducting every steps of the procedures incorporated in this SOP after a prior reading.

**Name**	**Signature and Date**
……………………………..	……………………………..
……………………………..	……………………………..
……………………………..	……………………………..
……………………………..	……………………………..
……………………………..	……………………………..

Prepared by: Addisu Legesse and Teshome Assefa	Authority: EPHI

NOTE: This is a CONTROLLED document for internal use only. Any documents appearing in paper form are not controlled and should be checked against the electronic version prior to use.

Document Change History

Revision No.	Date approved	Nature of revision
1	February, 2019	Initial release
2	November, 2020	Typographical adjustment<br>Document revision table is added

Prepared by: Addisu Legesse and Teshome Assefa	Authority: EPHI

NOTE: This is a CONTROLLED document for internal use only. Any documents appearing in paper form are not controlled and should be checked against the electronic version prior to use.
