# Supplementary material for: Ethiopia National Food and Nutrition Survey to inform the Ethiopian National Food and Nutrition Strategy: a study protocol
Source: BMJ Open. 2023 Apr 25;13(4):e067641. doi: 10.1136/bmjopen-2022-067641 (PMC10151871; doi:10.1136/bmjopen-2022-067641)
Supplement: Supplementary data [file bmjopen-2022-067641supp002.pdf]

Determination of serum retinol using hexane as the serum sample extraction procedure

Contents

1. Reagents and materials 2
2. Instrument (HPLC) parameters – we have Shimadzu prominence HPLC. 2
3. Sample extraction procedure 2
4. Calculation 3
5. Reference 3

Woldeyohannes M, *et al. BMJ Open* 2023; 13:e067641. doi: 10.1136/bmjopen-2022-067641

1. Reagents and materials

- Methanol (HPLC grade)
- Hexane (HPLC grade)
- Ethanol (HPLC grade/AR grade)
- Normal saline solution (0.9% NaCl)
- Retinol acetate (as internal standard)
- Retinol (as external standard)
- CRM (Certified Reference Material) for serum retinol
- 15 ml centrifuge tube
- Vortex mixer
- Centrifuge (up to 4000 rpm capacity)
- Centrifuge tube rack
- Micropipettes (10-100µl and 100-1000µl)
- Micropipettes tips for both types
- Stopwatch
- HPLC Vials (1ml)

2. Instrument (HPLC) parameters – we have Shimadzu prominence HPLC.

- Detector - SPD-10A UV/VIS at 325 nm
- Column – SUPELCOSIL™LC-NH_2-NP, 25cm × 4.6mm, 5µm
- Mobile phase – HPLC grade methanol
- Elution system – isocratic

3. Sample extraction procedure

- Add 200 µl serum sample to 15ml plastic test tube.
- Add an equal volume of 50 µg/dl retinol acetate and ethanol.
- Take 200 µl of a serious of standards (10, 20, 40, 60, 75 µg/dl) to the 15 ml centrifuge tube and add the same volume of retinol acetate and normal saline solution.
- Mix the standard and sample solutions with a vortex mixer
- Add 1ml of HPLC grade hexane and mix for 45 seconds
- Centrifuge the solution at 4000rpm for 7 minutes and transfer the supernatant to other test tubes
- The solution has to be extract twice by adding 1 ml hexane.

Woldeyohannes M, *et al. BMJ Open* 2023; 13:e067641. doi: 10.1136/bmjopen-2022-067641

- Dry the hexane using nitrogen gas and reconstitute by 500µl HPLC grade methanol
- It has to be mix for 30 minutes using a vortex mixer and transfer to 1 ml HPLC vials
- Analyze the extracted solutions using reversed-phase HPLC by isocratic elution system, at the flow rate of 1.5 ml/min, and injection volume 30µl

N: B: - Extract and analyse the CRM using the sample extraction procedure.

4. Calculation

- Plot the calibration curve using area ratio of retinol (standard) and retinol acetate (internal standard) vs concentration ratio (retinol: retinol acetate).
- From the linear equation formula, Y = mx + b, the concentration of serum retinol can be calculated.
  Where Y – is area ratio (retinol: retinol acetate) of sample
  M – is slope and b – is Y intercept
  X – is the concentration of serum retinol in µg/dl

5. Reference

Quadro, Y.-K. K. and L. (2013). Reverse-Phase High-Performance Liquid Chromatography (HPLC) Analysis of Retinol and Retinyl Esters in Mouse Serum and Tissues. *Methods Mol Biol. 2010; 652: 263–275. Doi:10.1007/978-1-60327-325-1_15.*, (12), 1–10. https://doi.org/10.1007/978-1-60327-325-1

von Lucke, A., Russell, R. M., Stephensen, C. B., Gannon, B. M., Craft, N. E., Haskell, M. J., … Raiten, D. J. (2016). Biomarkers of Nutrition for Development (BOND)—Vitamin A Review. *The Journal of Nutrition*, 1816s-1848s. https://doi.org/10.3945/jn.115.229708.FIGURE

Prepared by: Nahom Tefera (September 2020)

Reviewed by: Meseret W/yohannis

Approved by: Dr. Masresha Tessema
