# Supplementary material for: Ethiopia National Food and Nutrition Survey to inform the Ethiopian National Food and Nutrition Strategy: a study protocol
Source: BMJ Open. 2023 Apr 25;13(4):e067641. doi: 10.1136/bmjopen-2022-067641 (PMC10151871; doi:10.1136/bmjopen-2022-067641)
Supplement: Supplementary data [file bmjopen-2022-067641supp003.pdf]

	Name of institute	Doc. number:	Version No: 3
	**ETHIOPIAN PUBLIC HEALTH INSTITUTE**	**EPHI/TL/FNL/TM7.2-17**	Copy No. _____
			Page 1 of 5
Document Title: DETERMINATION OF IODINE IN URINE		Effective date: December, 2020	

1 Scope

This test method is applicable for the determination of iodine from a urine sample.

2 Purpose

This procedure provides methods to control the amount of iodine and it's toxicity by ammonium persulfate digestion with spectrophotometric detection of the Sandell-Kolthoff Reaction method from urine samples.

3 Principle

Urine is digested with ammonium persulfate. Iodide is the catalyst in the reduction of ceric ammonium sulfate (yellow) to cerous form (colorless) and is detected by the rate of color disappearance (Sandell-Kolthoff reaction).

4 Chemicals and Apparatus

a) Chemicals

- Ammonium persulphate
- Arsenic trioxide
- Ceric ammonium sulphate
- Potassium iodate
- Deionized water
- Sodium chloride

b) Apparatus

- Hot plate
- Oven
- UV-Vis spectrophotometer
- Volumetric flasks (100 – 2000 ml)
- Beakers (100- 1000 ml)

- Micropipette (100 – 1000 µl)
- Pipette (5 – 10 ml)
- Vortex mixer
- Measuring cylinder (100 – 1000 ml)
- Glass test tubes (13*100 mm)
- Analytical balance (nearest to the 0.0001 g)
- Erlenmeyer flask (2000 ml)

5 Reagents preparation

a) 1 M Ammonium persulphate

- Dissolve 114.1 g H_2 N_2 O_8 S_2 in deionized water and makeup to 500 ml with H_2O. Store away from light. Stable for at least one month.

b) 5 N H_2SO_4

- Slowly add 139 ml concentrated (36 N) H_2SO_4 to about 700 ml deionized water (careful - this generates heat!). When cool, adjust with deionized water to a final volume of 1 liter.

c) 3.5 N H_2SO_4

- Slowly add 97 ml concentrated (36 N) H_2SO_4 to about 800 ml deionized water (careful - this generates heat!), and when cool, adjusting with deionized water to a final volume of 1 liter

d) Ceric ammonium sulphate solution

- Dissolve 48 g ceric ammonium sulphate in 1 liter 3.5 N H_2SO_4. Store in a dark bottle away from light at room temperature. The solution is stable for months.

e) Arsenious acid solution

- In a 2000 ml Erlenmeyer flask, place 20 g As_2O_3 and 50 g NaCl, then slowly add 400 ml 5 N H_2SO_4. Add water to about 1 liter, heat gently to dissolve, cool to room temperature, dilute with water to 2 liters, filter, store in a dark bottle away from light at room temperature. The solution is stable for months.

6 Standards preparation

a) Stock standard solution (1 mg/ml)

- Dissolve 0.1685 g KIO_3 in deionized water to a final volume of 100 ml (1.68 g KIO_3 contains 1.0 g iodine). KIO_3 is preferred over KI because it is more stable. It may be more convenient to make a more concentrated solution, e.g., 10 or 100 mg iodine/ml.

b) Intermediate standard solution (1 µg/ml)

- Dilute 100 µl stock iodine standard (1mg/ml) to 100 ml deionized water. Store in a dark bottle. The solution is stable for months. Useful standards are 20, 50, 100, 150, 200, and 300 µg/l.

c) Serial standard dilutions

- From intermediate standards, prepare 50, 100, 150, 200, and 250 µg/L useful standards for calibration curve purposes.

Note: All standard solutions should store in a dark place. The solutions are stable for months.

7 Procedures

- Mix urine to suspend sediment using a vortex mixer.
- Pipette 250 µl of each urine sample into a 13 x 100 mm test tube.
- Pipette 250 µl each iodine serial standards also into a test tube.
- Add 1 ml 1.0 M ammonium persulfate solution to each tube.
- Vortex all tubes using a vortex mixer
- Heat all tubes for 60 minutes at 100° C in the oven.
- Cool tubes to room temperature in a fume hood.
- Add 2.5 ml arsenious acid solution. Mix by a vortex. Let stand for 15 minutes.
- Add 300 µl of ceric ammonium sulfate solution to each tube (quickly mixing) at 15-30 second intervals between successive tubes. A stopwatch should be used for this. With practice, a 20-second interval is convenient.

- Allow sitting at room temperature. Exactly 30 minutes after the addition of ceric ammonium sulfate to the first tube, read its absorbance by using UV-Vis spectrophotometer at 420 nm. Read successive tubes at the same interval as when adding the ceric ammonium sulfate.

8 Calculation

- Construct a standard curve on graph paper by plotting log absorbance of each concentration versus iodine concentration of each standard.
- Urinary Iodine in µg/l = ((log A-b)/(m)) * 10
  Where log A is log absorbance of sample, b is Y-intercept of the calibration graph, m is the slope of the graph, and 10 is used as a conversion factor when we prepare serials of standards in µg/dl (in this case we multiply the final concentration (µg/ dl) by 10 to get the concentration in µg /l).

9 Quality control and safety precautions

- In each batch, the urine quality control sample (CRM or in-house prepared) should run together with sample and standards.
- While working the urine analysis and reagent preparation wear gloves, lab coat, eye goggle, lab shoe, and mouth cover.

Remember: During the analysis of iodine from urine, you should ensure that the laboratory working environment should free from salt samples (especially iodized salt samples) to avoid contamination.

Limitations: If the urine sample is analyzed at a high temperature the loss of iodine occurred and the method has to detect very low iodine concentration in urine.

10 References

- ICCIDD, UNICEF, WHO. Dunn JT et al. Methods for measuring iodine in urine. The Netherlands, ICCIDD, 1993.

Prepared by	Signature	Date
Prepared by: Nahom Tefera	___________	_________________
Reviewed by: Meseret W/yohannes	___________	__________________
Approved by: Dr. Masresha Tessema	___________	__________________
