# Supplementary material for: Ethiopia National Food and Nutrition Survey to inform the Ethiopian National Food and Nutrition Strategy: a study protocol
Source: BMJ Open. 2023 Apr 25;13(4):e067641. doi: 10.1136/bmjopen-2022-067641 (PMC10151871; doi:10.1136/bmjopen-2022-067641)
Supplement: Supplementary data [file bmjopen-2022-067641supp004.pdf]

0003333795190c501V10.0

AAGP2

cobas®

Tina-quant α1-Acid Glycoprotein Gen.2

Order information

REF	CONTENT		Analyzer(s) on which **cobas c** pack(s) can be used
**03333795** 190	Tina-quant α1-Acid Glycoprotein Gen.2 100 tests	System-ID 07 6758 1	Roche/Hitachi **cobas c** 311, **cobas c** 501/502
**11355279** 216	Calibrator f.a.s. Proteins (5 x 1 mL)	Code 656	
**11355279** 160	Calibrator f.a.s. Proteins (5 x 1 mL, for USA)	Code 656	
**10557897** 122	Precinorm Protein (3 x 1 mL)	Code 302	
**10557897** 160	Precinorm Protein (3 x 1 mL, for USA)	Code 302	
**11333127** 122	Precipath Protein (3 x 1 mL)	Code 303	
**11333127** 160	Precipath Protein (3 x 1 mL, for USA)	Code 303	
**05117003** 190	PreciControl ClinChem Multi 1 (20 x 5 mL)	Code 391	
**05947626** 190	PreciControl ClinChem Multi 1 (4 x 5 mL)	Code 391	
**05947626** 160	PreciControl ClinChem Multi 1 (4 x 5 mL, for USA)	Code 391	
**05117216** 190	PreciControl ClinChem Multi 2 (20 x 5 mL)	Code 392	
**05947774** 190	PreciControl ClinChem Multi 2 (4 x 5 mL)	Code 392	
**05947774** 160	PreciControl ClinChem Multi 2 (4 x 5 mL, for USA)	Code 392	
**04489357** 190	Diluent NaCl 9 % (50 mL)	System-ID 07 6869 3	

English

System information

For **cobas c** 311/501 analyzers:

AAGP2: ACN 229

For **cobas c** 502 analyzer:

AAGP2: ACN 8229

Intended use

In vitro test for the quantitative determination of α_1-acid glycoprotein in human serum and plasma on Roche/Hitachi **cobas c** systems.

Summary[1,2,3,4,5]

α_1-Acid glycoprotein is synthesized in hepatocytes and consists of a polypeptide chain having 5 carbohydrate chains N-glycosidically bonded to it (molar mass 41000 daltons). Structurally, it belongs to the lipocalin superfamily of secretory proteins (such as α_1-microglobulin and retinol-binding protein). α_1-Acid glycoprotein promotes fibroblast growth and interacts with collagen.

It is a sensitive acute phase reactant whose concentration can increase by a factor of 3 within 24-48 hours when inflammation occurs. α_1-Acid glycoprotein can also be used to differentiate between acute phase reactions (elevated serum level) and estrogen effects (normal or decreased serum level) whereas the serum level of other positive reactants such as ceruloplasmin and haptoglobin increases during such reactions. Along with haptoglobin it is perhaps the best protein for identifying slight in vivo hemolysis. An increased α_1-acid glycoprotein level and normal haptoglobin values indicate an acute phase reaction with concomitant slight in vivo hemolysis. Moderate and isolated increases occur when glomerular filtration is inhibited in the early stages of uremia. The determination is used in the assessment of the activity of acute and recurring inflammations as well as of tumors with cell necrosis.

Various assay methods for α_1-acid glycoprotein determination are available such as kinetic nephelometry, radial immunodiffusion (RID) and turbidimetry. The Roche α_1-acid glycoprotein assay is based on the principle of immunological agglutination.

Test principle[2]

Immunoturbidimetric assay.

Anti-α_1-acid glycoprotein antibodies react with antigen in the sample to form an antigen/antibody complex. Following agglutination, this is measured turbidimetrically.

Reagents - working solutions

R1 TRIS buffer: 50 mmol/L, pH 8.0; NaCl: 300 mmol/L; PEG: 7 %; preservative; stabilizer

R2 Polyclonal anti-human α_1-acid glycoprotein antibody (goat): dependent on titer; TRIS buffer: 13 mmol/L, pH 7.5; NaCl: 198 mmol/L; preservative

R1 is in position B and R2 is in position C.

Precautions and warnings

For in vitro diagnostic use.
Exercise the normal precautions required for handling all laboratory reagents.
Disposal of all waste material should be in accordance with local guidelines.
Safety data sheet available for professional user on request.

For USA: Caution: Federal law restricts this device to sale by or on the order of a physician.

Reagent handling

Ready for use

Storage and stability

AAGP2

Shelf life at 2-8 °C:	See expiration date on **cobas c** pack label.
On-board in use and refrigerated on the analyzer:	12 weeks

Diluent NaCl 9 %

Shelf life at 2-8 °C:	See expiration date on **cobas c** pack label.
On-board in use and refrigerated on the analyzer:	12 weeks

Specimen collection and preparation

For specimen collection and preparation only use suitable tubes or collection containers.

Only the specimens listed below were tested and found acceptable.
Serum
Plasma: Li-heparin and K_2-EDTA plasma.

The sample types listed were tested with a selection of sample collection tubes that were commercially available at the time of testing, i.e. not all available tubes of all manufacturers were tested. Sample collection systems from various manufacturers may contain differing materials which could affect the test results in some cases. When processing samples in primary tubes (sample collection systems), follow the instructions of the tube manufacturer.

Centrifuge samples containing precipitates before performing the assay.

Stability:[6]	< 72 hours at 4 °C
	6 months at -20 °C

Woldeyohannes M, *et al. BMJ Open* 2023; 13:e067641. doi: 10.1136/bmjopen-2022-067641

AAGP2

Tina-quant α1-Acid Glycoprotein Gen.2

cobas®

Materials provided

See “Reagents – working solutions” section for reagents.

Materials required (but not provided)

- See “Order information” section
- General laboratory equipment

Assay

For optimum performance of the assay follow the directions given in this document for the analyzer concerned. Refer to the appropriate operator’s manual for analyzer-specific assay instructions.

The performance of applications not validated by Roche is not warranted and must be defined by the user.

Application for serum and plasma

cobas c 311 **test definition**

Assay type	2-Point End		
Reaction time/Assay points	10/6-32		
Wavelength (sub/main)	660/340 nm		
Reaction direction	Increase		
Units	g/L (µmol/L, mg/dL)		
Reagent pipetting		Diluent (H_2O)	
R1	120 µL	–	
R2	40 µL	–	
Sample volumes	*Sample*	*Sample dilution*	
		Sample	*Diluent (NaCl)*
Normal	12 µL	9 µL	180 µL
Decreased	12 µL	4 µL	122 µL
Increased	12 µL	9 µL	180 µL

cobas c 501 **test definition**

Assay type	2-Point End		
Reaction time/Assay points	10/10-48		
Wavelength (sub/main)	660/340 nm		
Reaction direction	Increase		
Units	g/L (µmol/L, mg/dL)		
Reagent pipetting		Diluent (H_2O)	
R1	120 µL	–	
R2	40 µL	–	
Sample volumes	*Sample*	*Sample dilution*	
		Sample	*Diluent (NaCl)*
Normal	12 µL	9 µL	180 µL
Decreased	12 µL	4 µL	122 µL
Increased	12 µL	9 µL	180 µL

cobas c 502 **test definition**

Assay type	2-Point End		
Reaction time/Assay points	10/10-48		
Wavelength (sub/main)	660/340 nm		
Reaction direction	Increase		
Units	g/L (µmol/L, mg/dL)		
Reagent pipetting		Diluent (H_2O)	
R1	120 µL	–	
R2	40 µL	–	
Sample volumes	*Sample*	*Sample dilution*	
		Sample	*Diluent (NaCl)*
Normal	12 µL	9 µL	180 µL
Decreased	12 µL	4 µL	122 µL
Increased	12 µL	18 µL	180 µL

Calibration

Calibrators	S1: H_2O
	S2-S6: C.f.a.s. Proteins
	Multiply the lot-specific C.f.a.s. Proteins calibrator value by the factors below to determine the standard concentrations for the 6-point calibration curve:
	S2: 0.140 S5: 1.40
	S3: 0.280 S6: 2.81
	S4: 0.700
Calibration mode	RCM2
Calibration frequency	Full calibration<br>- after reagent lot change<br>- as required following quality control procedures

Calibration interval may be extended based on acceptable verification of calibration by the laboratory.

Traceability: This method has been standardized against the reference preparation of the IRMM (Institute for Reference Materials and Measurements) BCR470/CRM470 (RPPHS - Reference Preparation for Proteins in Human Serum).[7]

Quality control

For quality control, use control materials as listed in the "Order information" section.

In addition, other suitable control material can be used.

The control intervals and limits should be adapted to each laboratory’s individual requirements. Values obtained should fall within the defined limits. Each laboratory should establish corrective measures to be taken if values fall outside the defined limits.

Follow the applicable government regulations and local guidelines for quality control.

Calculation

Roche/Hitachi **cobas c** systems automatically calculate the analyte concentration of each sample.

Conversion factors:	g/L x 25 = µmol/L	mg/dL x 0.01 = g/L
	mg/dL x 0.25 = µmol/L	g/L x 100 = mg/dL

Limitations - interference

Criterion: Recovery within ± 10 % of initial value at an α_1-acid glycoprotein concentration of 0.5 g/L (12.5 µmol/L, 50 mg/dL).

Icterus:[8] No significant interference up to an I index of 60 for conjugated and unconjugated bilirubin (approximate conjugated and unconjugated bilirubin concentration: 1026 µmol/L or 60 mg/dL).

Hemolysis:[8] No significant interference up to an H index of 1000 (approximate hemoglobin concentration: 621 µmol/L or 1000 mg/dL).

Lipemia (Intralipid):[8] No significant interference up to an L index of 650. There is poor correlation between the L index (corresponds to turbidity) and triglycerides concentration.

Rheumatoid factors up to 1200 IU/mL do not interfere.

High dose hook-effect: No false result occurs up to an α_1-acid glycoprotein concentration of 11 g/L (275 µmol/L, 1100 mg/dL).

Drugs: No interference was found at therapeutic concentrations using common drug panels.[9, 10]

In very rare cases, gammopathy, in particular type IgM (Waldenström’s macroglobulinemia), may cause unreliable results.[11]

0003333795190c501V10.0

AAGP2

Tina-quant α1-Acid Glycoprotein Gen.2

For diagnostic purposes, the results should always be assessed in conjunction with the patient's medical history, clinical examination and other findings.

ACTION REQUIRED

Special Wash Programming: The use of special wash steps is mandatory when certain test combinations are run together on Roche/Hitachi **cobas c** systems. The latest version of the carry-over evasion list can be found with the NaOHD-SMS-SmpCln1+2-SCCS Method Sheets. For further instructions refer to the operator's manual. **cobas c** 502 analyzer: All special wash programming necessary for avoiding carry-over is available via the **cobas** link, manual input is not required.

Where required, special wash/carry-over evasion programming must be implemented prior to reporting results with this test.

Limits and ranges

Measuring range

0.1-4.0 g/L (2.5-100 µmol/L, 10-400 mg/dL)

Determine samples having higher concentrations via the rerun function. Dilution of samples via the rerun function is a 1:1.5 dilution. Results from samples diluted using the rerun function are automatically multiplied by a factor of 1.5.

Lower limits of measurement

Lower detection limit of the test

0.1 g/L (2.5 µmol/L, 10 mg/dL)

The lower detection limit represents the lowest measurable analyte level that can be distinguished from zero. It is calculated as the value lying 3 standard deviations above that of the lowest standard (standard 1 + 3 SD, repeatability, n = 21).

Expected values[12]

0.5-1.2 g/L (12.5-30 µmol/L, 50-120 mg/dL)

Each laboratory should investigate the transferability of the expected values to its own patient population and if necessary determine its own reference ranges.

Specific performance data

Representative performance data on the analyzers are given below. Results obtained in individual laboratories may differ.

Precision

Precision was determined using human samples and controls in an internal protocol with repeatability (n = 21) and intermediate precision (3 aliquots per run, 1 run per day, 21 days). The following results were obtained:

Repeatability	*Mean* g/L (µmol/L, mg/dL)	*SD* g/L (µmol/L, mg/dL)	*CV* %
Precinorm Protein	0.724 (18.1, 72.4)	0.00 (0.0, 0.0)	0.6
Precipath Protein	1.21 (30.3, 121)	0.01 (0.3, 1)	0.5
Human serum 1	0.642 (16.1, 64.2)	0.00 (0.0, 0.0)	0.7
Human serum 2	1.07 (26.8, 107)	0.01 (0.3, 1)	0.7

Intermediate precision	*Mean* g/L (µmol/L, mg/dL)	*SD* g/L (µmol/L, mg/dL)	*CV* %
Precinorm Protein	0.710 (17.8, 71.0)	0.007 (0.2, 1.0)	0.9
Precipath Protein	1.19 (30.0, 119)	0.01 (0.3, 1)	0.9
Human serum 3	0.660 (16.5, 66.0)	0.010 (0.3, 1.0)	1.5
Human serum 4	1.21 (30.3, 121)	0.02 (0.5, 2)	1.5

Method comparison

α_1-Acid glycoprotein values for human serum and plasma samples obtained on a Roche/Hitachi **cobas c** 501 analyzer (y) were compared with those determined using the corresponding reagent on a Roche/Hitachi 917 analyzer (x).

Sample size (n) = 119

Passing/Bablok[13]	Linear regression
y = 1.012x - 0.070 g/L	y = 0.998x - 0.056 g/L
τ = 0.973	r = 0.999

The sample concentrations were between 0.489 and 3.25 g/L (12.2 and 81.3 µmol/L, 48.9 and 325 mg/dL).

References

1 Schmid K. α1-Acid glycoprotein. In: The Plasma Proteins, 2nd ed. Putnam FW, ed. New York: Academic Press 1975;183-228.

2 Greiling H, Gressner AM, eds. Lehrbuch der Klinischen Chemie und Pathobiochemie, 3rd ed. Stuttgart/New York: Schattauer Verlag 1995:236.

3 Tietz NW, ed. Clinical Guide to Laboratory Tests, 3rd ed. Philadelphia, PA: WB Saunders Company 1995;66-67.

4 Ganrot K. Plasma protein pattern in acute infectious disease. Scand J Clin Lab Invest 1974;34:75-81.

5 Lievens M, Bienvenu J, Buitrago JMG, et al. Evaluation of four new Tina-quant assays for determination of α1-acid glycoprotein, α1-antitrypsin, haptoglobin and prealbumin. Clin Lab 1996;42:515-520.

6 Wu AHB, ed. Tietz Clinical Guide to Laboratory Tests, 4th ed. St. Louis (MO): Saunders Elsevier 2006:42.

7 Baudner S, Bienvenu J, Blirup-Jensen S, et al. The certification of a matrix reference material for immunochemical measurement of 14 human serum proteins CRM470. Report EUR 15243 EN 1993;1-186.

8 Glick MR, Ryder KW, Jackson SA. Graphical Comparisons of Interferences in Clinical Chemistry Instrumentation. Clin Chem 1986;32:470-475.

9 Breuer J. Report on the Symposium "Drug effects in Clinical Chemistry Methods". Eur J Clin Chem Clin Biochem 1996;34:385-386.

10 Sonntag O, Scholer A. Drug interference in clinical chemistry: recommendation of drugs and their concentrations to be used in drug interference studies. Ann Clin Biochem 2001;38:376-385.

11 Bakker AJ, Mücke M. Gammopathy interference in clinical chemistry assays: mechanisms, detection and prevention. Clin Chem Lab Med 2007;45(9):1240-1243.

12 Dati F, Schumann G, Thomas L, et al. Consensus of a group of professional societies and diagnostic companies on guidelines for interim reference ranges for 14 proteins in serum based on the standardization against the IFCC/BCR/CAP reference material (CRM 470). Eur J Clin Chem Clin Biochem 1996;34:517-520.

13 Bablok W, Passing H, Bender R, et al. A general regression procedure for method transformation. Application of linear regression procedures for method comparison studies in clinical chemistry, Part III. J Clin Chem Clin Biochem 1988 Nov;26(11):783-790.

A point (period/stop) is always used in this Method Sheet as the decimal separator to mark the border between the integral and the fractional parts of a decimal numeral. Separators for thousands are not used.

Symbols

Roche Diagnostics uses the following symbols and signs in addition to those listed in the ISO 15223-1 standard (for USA: see https://usdiagnostics.roche.com for definition of symbols used):

CONTENT	Contents of kit
→	Volume after reconstitution or mixing
GTIN	Global Trade Item Number

Woldeyohannes M, *et al. BMJ Open* 2023; 13:e067641. doi: 10.1136/bmjopen-2022-067641

0003333795190c501V10.0

AAGP2

Tina-quant α1-Acid Glycoprotein Gen.2

cobas®

FOR US CUSTOMERS ONLY: LIMITED WARRANTY

Roche Diagnostics warrants that this product will meet the specifications stated in the labeling when used in accordance with such labeling and will be free from defects in material and workmanship until the expiration date printed on the label. THIS LIMITED WARRANTY IS IN LIEU OF ANY OTHER WARRANTY, EXPRESS OR IMPLIED, INCLUDING ANY IMPLIED WARRANTY OF MERCHANTABILITY OR FITNESS FOR PARTICULAR PURPOSE. IN NO EVENT SHALL ROCHE DIAGNOSTICS BE LIABLE FOR INCIDENTAL, INDIRECT, SPECIAL OR CONSEQUENTIAL DAMAGES.

COBAS, COBAS C, PRECICONTROL, PRECINORM, PRECIPATH and TINA-QUANT are trademarks of Roche.
All other product names and trademarks are the property of their respective owners.
Additions, deletions or changes are indicated by a change bar in the margin.
© 2017, Roche Diagnostics

CE

Roche Diagnostics GmbH, Sandhofer Strasse 116, D-68305 Mannheim
www.roche.com
Distribution in USA by:
Roche Diagnostics, Indianapolis, IN
US Customer Technical Support 1-800-428-2336

Roche
