# Supplementary material for: Ethiopia National Food and Nutrition Survey to inform the Ethiopian National Food and Nutrition Strategy: a study protocol
Source: BMJ Open. 2023 Apr 25;13(4):e067641. doi: 10.1136/bmjopen-2022-067641 (PMC10151871; doi:10.1136/bmjopen-2022-067641)
Supplement: Supplementary data [file bmjopen-2022-067641supp005.pdf]

Supplemental material BMJ Publishing Group Limited (BMJ) disclaims all liability and responsibility arising from any reliance placed on this supplemental material which has been supplied by the author(s) *BMJ Open*

ms_05894913190V7.0

Vitamin D total

cobas®

25-Hydroxyvitamin D

REF	Σ	SYSTEM
**05894913** 190	100	Elecsys 2010<br>MODULAR ANALYTICS E170<br>**cobas e** 411<br>**cobas e** 601<br>**cobas e** 602

English

Intended use

This assay is intended for the quantitative determination of total 25-hydroxyvitamin D in human serum and plasma. This assay is to be used as an aid in the assessment of vitamin D sufficiency.

The electrochemiluminescence binding assay is intended for use on Elecsys and **cobas e** immunoassay analyzers.

Summary

Vitamin D is a fat-soluble steroid hormone precursor that is mainly produced in the skin by exposure to sunlight. Vitamin D is biologically inert and must undergo two successive hydroxylations in the liver and kidney to become the biologically active 1,25-dihydroxyvitamin D.[1]

The two most important forms of vitamin D are vitamin D_3 (cholecalciferol) and vitamin D_2 (ergocalciferol). In contrast to vitamin D_3, the human body cannot produce vitamin D_2 which is taken up with fortified food or given by supplements. In human plasma vitamin D_3 and D_2 are bound to the vitamin D binding protein and transported to the liver where both are hydroxylated to form vitamin D (25-OH), i.e. 25-hydroxyvitamin D. It is commonly agreed that vitamin D (25-OH) is the metabolite to determine the overall vitamin D status as it is the major storage form of vitamin D in the human body. This primary circulating form of vitamin D is biologically inactive with levels approximately 1000-fold greater than the circulating 1,25-dihydroxyvitamin D. The half-life of circulating vitamin D (25-OH) is 2-3 weeks.

Most of the vitamin D (25-OH), measurable in serum, is vitamin D_3 (25-OH) whereas vitamin D_2 (25-OH) reaches measurable levels only in patients taking vitamin D_2 supplements.[2,3,4] Vitamin D_2 is considered to be less effective.[5]

Vitamin D is essential for bone health. In children, severe deficiency leads to bone-malformation, known as rickets. Milder degrees of insufficiency are believed to cause reduced efficiency in the utilization of dietary calcium.[6] Vitamin D deficiency causes muscle weakness; in elderly, the risk of falling has been attributed to the effect of vitamin D on muscle function.[7] Vitamin D deficiency is a common cause of secondary hyperparathyroidism.[8,9] Elevations of PTH levels, especially in elderly vitamin D deficient adults can result in osteomalacia, increased bone turnover, reduced bone mass and risk of bone fractures.[10] Low vitamin D (25-OH) concentrations are also associated with lower bone mineral density.[11] In conjunction with other clinical data, the results may be used as an aid in the assessment of bone metabolism.

So far, vitamin D has been shown to affect expression of over 200 different genes. Insufficiency has been linked to diabetes, different forms of cancer, cardiovascular disease, autoimmune diseases and innate immunity.[2]

The Elecsys Vitamin D total assay employs a vitamin D binding protein (VDBP) as capture protein to bind vitamin D_3 (25-OH) and vitamin D_2 (25-OH).

Test principle

Competition principle. Total duration of assay: 27 minutes.

- 1st incubation: By incubating the sample (15 µL) with pretreatment reagent 1 and 2, bound vitamin D (25-OH) is released from the vitamin D binding protein.
- 2nd incubation: By incubating the pretreated sample with the ruthenium labeled vitamin D binding protein, a complex between the vitamin D (25-OH) and the ruthenylated vitamin D binding protein is formed.
- 3rd incubation: After addition of streptavidin-coated microparticles and vitamin D (25-OH) labeled with biotin, unbound ruthenium labeled vitamin D binding proteins become occupied. A complex consisting of the ruthenylated vitamin D binding protein and the biotinylated vitamin D (25-OH) is formed and becomes bound to the solid phase via interaction of biotin and streptavidin.
- The reaction mixture is aspirated into the measuring cell where the microparticles are magnetically captured onto the surface of the electrode. Unbound substances are then removed with ProCell/ProCell M. Application of a voltage to the electrode then induces chemiluminescent emission which is measured by a photomultiplier.
- Results are determined via a calibration curve which is instrument-specifically generated by 2-point calibration and a master curve provided via the reagent barcode.

Reagents - working solutions

The reagent rackpack (M, R1, R2) and the pretreatment reagents (PT1, PT2) are labeled as VITD-T.

PT1 Pretreatment reagent 1 (white cap), 1 bottle, 4 mL:
Dithiothreitol 1 g/L, pH 5.5.

PT2 Pretreatment reagent 2 (gray cap), 1 bottle, 4 mL:
Sodium hydroxide 55 g/L.

M Streptavidin-coated microparticles (transparent cap), 1 bottle, 6.5 mL:
Streptavidin-coated microparticles 0.72 mg/mL; preservative.

R1 Vitamin D binding protein-BPRu (gray cap), 1 bottle, 9 mL:
Ruthenium labeled vitamin D binding protein 150 µg/L; bis-tris propane buffer 200 mmol/L; albumin (human) 25 g/L; pH 7.5; preservative.

R2 25-hydroxyvitamin D~biotin (black cap), 1 bottle, 8.5 mL:
Biotinylated vitamin D (25-OH) 14 µg/L; bis-tris propane buffer 200 mmol/L; pH 8.6; preservative.

Precautions and warnings

For in vitro diagnostic use.
Exercise the normal precautions required for handling all laboratory reagents.
Disposal of all waste material should be in accordance with local guidelines.
Safety data sheet available for professional user on request.

This kit contains components classified as follows in accordance with the Regulation (EC) No. 1272/2008:

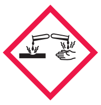

Danger

H290	May be corrosive to metals.
H314	Causes severe skin burns and eye damage.

Prevention:

P280	Wear protective gloves/ protective clothing/ eye protection/ face protection.

Response:

Woldeyohannes M, *et al. BMJ Open* 2023; 13:e067641. doi: 10.1136/bmjopen-2022-067641

ms_05894913190V7.0

Vitamin D total

cobas®

25-Hydroxyvitamin D

P303 + P361 + P353	IF ON SKIN (or hair): Remove/Take off immediately all contaminated clothing. Rinse skin with water/shower.
P304 + P340 + P310	IF INHALED: Remove person to fresh air and keep comfortable for breathing. Immediately call a POISON CENTER or doctor/physician.
P305 + P351 + P338 + P310	IF IN EYES: Rinse cautiously with water for several minutes. Remove contact lenses, if present and easy to do. Continue rinsing. Immediately call a POISON CENTER or doctor/ physician.

Product safety labeling primarily follows EU GHS guidance.

Contact phone: all countries: +49-621-7590

All human material should be considered potentially infectious. All products derived from human blood are prepared exclusively from the blood of donors tested individually and shown to be free from HBsAg and antibodies to HCV and HIV. The testing methods applied were FDA-approved or cleared in compliance with the European Directive 98/79/EC, Annex II, List A.

However, as no testing method can rule out the potential risk of infection with absolute certainty, the material should be handled with the same level of care as a patient specimen. In the event of exposure, the directives of the responsible health authorities should be followed.[12,13]

Avoid foam formation in all reagents and sample types (specimens, calibrators and controls).

Reagent handling

The reagents in the kit have been assembled into a ready-for-use unit that cannot be separated.

All information required for correct operation is read in from the respective reagent barcodes.

Storage and stability

Store at 2-8 °C.

Do not freeze.

Store the Elecsys reagent kit **upright** in order to ensure complete availability of the microparticles during automatic mixing prior to use.

Stability:	
unopened at 2-8 °C	up to the stated expiration date
after opening at 2-8 °C	56 days (8 weeks)
on Elecsys 2010 and **cobas e** 411	21 days (3 weeks)
on MODULAR ANALYTICS E170, **cobas e** 601 and **cobas e** 602	28 days (4 weeks)

Specimen collection and preparation

Only the specimens listed below were tested and found acceptable.

Serum collected using standard sampling tubes or tubes containing separating gel.

Li-heparin, K_2- and K_3-EDTA plasma as well as Li-heparin plasma tubes containing separating gel.

Criterion: Method comparison serum versus plasma, slope 0.9-1.1 + intercept within < ± 2 x LoB + coefficient of correlation > 0.9.

Serum, Li-heparin, K_2- and K_3-EDTA plasma: Vitamin D (25-OH) is stable for 8 hours at 18-25 °C, 4 days at 2-8 °C, 24 weeks at -20 °C.

The stability of vitamin D (25-OH) found with the Elecsys Vitamin D total assay is in line with earlier studies using a vitamin D binding protein assay and mass spectrometry.[14]

The sample types listed were tested with a selection of sample collection tubes that were commercially available at the time of testing, i.e. not all available tubes of all manufacturers were tested. Sample collection systems from various manufacturers may contain differing materials which could affect the test results in some cases. When processing samples in primary tubes (sample collection systems), follow the instructions of the tube manufacturer.

Centrifuge samples containing precipitates before performing the assay.

Do not use heat-inactivated samples.

Do not use samples and controls stabilized with azide.

Ensure the samples, calibrators and controls are at 20-25 °C prior to measurement.

Due to possible evaporation effects, samples, calibrators and controls on the analyzers should be analyzed/measured within 2 hours.

Materials provided

See "Reagents – working solutions" section for reagents.

Materials required (but not provided)

- REF 05894921190, Vitamin D total CalSet, for 4 x 1 mL
- REF 05618860190, PreciControl Varia, for 2 x 3 mL each of PreciControl Varia 1 and 2
- REF 11732277122, Diluent Universal, 2 x 16 mL sample diluent or
  REF 03183971122, Diluent Universal, 2 x 36 mL sample diluent
- General laboratory equipment
- Elecsys 2010, MODULAR ANALYTICS E170 or **cobas e** analyzer

Accessories for Elecsys 2010 and **cobas e** 411 analyzers:

- REF 11662988122, ProCell, 6 x 380 mL system buffer
- REF 11662970122, CleanCell, 6 x 380 mL measuring cell cleaning solution
- REF 11930346122, Elecsys SysWash, 1 x 500 mL washwater additive
- REF 11933159001, Adapter for SysClean
- REF 11706802001, Elecsys 2010 AssayCup, 60 x 60 reaction vessels
- REF 11706799001, Elecsys 2010 AssayTip, 30 x 120 pipette tips

Accessories for MODULAR ANALYTICS E170, **cobas e** 601 and **cobas e** 602 analyzers:

- REF 04880340190, ProCell M, 2 x 2 L system buffer
- REF 04880293190, CleanCell M, 2 x 2 L measuring cell cleaning solution
- REF 03023141001, PC/CC-Cups, 12 cups to prewarm ProCell M and CleanCell M before use
- REF 03005712190, ProbeWash M, 12 x 70 mL cleaning solution for run finalization and rinsing during reagent change
- REF 03004899190, PreClean M, 5 x 600 mL detection cleaning solution
- REF 12102137001, AssayTip/AssayCup Combimagazine M, 48 magazines x 84 reaction vessels or pipette tips, waste bags
- REF 03023150001, WasteLiner, waste bags
- REF 03027651001, SysClean Adapter M

Accessories for all analyzers:

- REF 11298500316, ISE Cleaning Solution/Elecsys SysClean, 5 x 100 mL system cleaning solution

Assay

For optimum performance of the assay follow the directions given in this document for the analyzer concerned. Refer to the appropriate operator's manual for analyzer-specific assay instructions.

Resuspension of the microparticles takes place automatically prior to use. Read in the test-specific parameters via the reagent barcode. If in exceptional cases the barcode cannot be read, enter the 15-digit sequence of numbers.

MODULAR ANALYTICS E170, **cobas e** 601 and **cobas e** 602 analyzers: PreClean M solution is necessary.

Bring the cooled reagents to approximately 20 °C and place on the reagent disk (20 °C) of the analyzer. Avoid foam formation. The system automatically regulates the temperature of the reagents and the opening/closing of the bottles.

Calibration

Traceability: This method has been standardized against LC-MS/MS[15] which in turn has been standardized to the NIST standard.[16]

Every Elecsys reagent set has a barcoded label containing specific information for calibration of the particular reagent lot. The predefined master curve is adapted to the analyzer using the relevant CalSet.

ms_05894913190V7.0

Vitamin D total

cobas®

25-Hydroxyvitamin D

Calibration frequency: Calibration must be performed once per reagent lot using fresh reagent (i.e. not more than 24 hours since the reagent kit was registered on the analyzer). Renewed calibration is recommended as follows:

- after 1 month (28 days) when using the same reagent lot
- after 7 days (when using the same reagent kit on the analyzer)
- as required: e.g. quality control findings outside the defined limits

Quality control

For quality control, use PreciControl Varia.

In addition, other suitable control material can be used.

Controls for the various concentration ranges should be run individually at least once every 24 hours when the test is in use, once per reagent kit, and following each calibration.

The control intervals and limits should be adapted to each laboratory's individual requirements. Values obtained should fall within the defined limits. Each laboratory should establish corrective measures to be taken if values fall outside the defined limits.

Follow the applicable government regulations and local guidelines for quality control.

Calculation

The analyzer automatically calculates the analyte concentration of each sample (either in ng/mL or nmol/L).

Conversion factors: nmol/L x 0.40 = ng/mL
ng/mL x 2.50 = nmol/L

Limitations - interference

Samples showing visible signs of hemolysis may cause interference.

Hemoglobin concentrations > 2 g/L (> 0.124 mmol/L) may lead to elevated results.

The assay is unaffected by icterus (bilirubin < 1129 µmol/L or < 66 mg/dL), lipemia (Intralipid < 400 mg/dL) and biotin (< 287 nmol/L or < 70 ng/mL).

Criterion: For concentrations from LoQ up to 15 ng/mL, deviation is ≤ 1.5 ng/mL; for concentrations > 15 ng/mL, deviation is ≤ 10 %.

Samples should not be taken from patients receiving therapy with high biotin doses (i.e. > 5 mg/day) until at least 8 hours following the last biotin administration.

In vitro tests were performed on 17 commonly used pharmaceuticals and 5 special therapeutic drugs (Bonviva (Ibandronate), EinsAlpha (Alfacalcidol), Fosamax (Alendronate), Pamidron HEXAL (Pamidronate) and Zometa (Zoledronate)). No interference with the assay was found.

In rare cases, interference due to extremely high titers of antibodies to analyte-specific antibodies, streptavidin or ruthenium can occur. These effects are minimized by suitable test design.

For diagnostic purposes, the results should always be assessed in conjunction with the patient's medical history, clinical examination and other findings.

Limits and ranges

Measuring range

3.00-70.0 ng/mL or 7.50-175 nmol/L (defined by the Limit of Detection and the maximum of the master curve). Values below the Limit of Detection are reported as < 3.00 ng/mL (< 7.50 nmol/L). Values above the measuring range are reported as > 70.0 ng/mL (> 175 nmol/L).

Lower limits of measurement

Limit of Blank (LoB), Limit of Detection (LoD) and Limit of Quantitation (LoQ)

Limit of Blank = 2.00 ng/mL (5.00 nmol/L)

Limit of Detection = 3.00 ng/mL (7.50 nmol/L)

Limit of Quantitation = 5.00 ng/mL (12.5 nmol/L) with a total allowable relative error of ≤ 30 %

The Limit of Blank, Limit of Detection and Limit of Quantitation were determined in accordance with the CLSI (Clinical and Laboratory Standards Institute) EP17-A requirements.

The Limit of Blank is the 95th percentile value from n ≥ 60 measurements of analyte-free samples over several independent series. The Limit of Blank corresponds to the concentration below which analyte-free samples are found with a probability of 95 %.

The Limit of Detection is determined based on the Limit of Blank and the standard deviation of low concentration samples. The Limit of Detection corresponds to the lowest analyte concentration which can be detected (value above the Limit of Blank with a probability of 95 %).

The Limit of Quantitation is defined as the lowest amount of analyte in a sample that can be accurately quantitated with a total allowable relative error of ≤ 30 %.

The total error concept describes the maximum possible error of a test result taking into account the imprecision (SD) and inaccuracy (bias) of the test system. The Total Error (TE) was determined using the RMS (Root Mean Square) model (CLSI EP17-A2). The relative allowable total error refers to the respective concentration of the sample.

Dilution

Samples with vitamin D (25-OH) concentrations above the measuring range can be manually diluted with Diluent Universal or a suitable human serum with a low analyte concentration. The recommended dilution is 1:2. The concentration of the diluted sample must be > 30.0 ng/mL (> 75.0 nmol/L). After manual dilution, multiply the results by the dilution factor 2. The endogenous analyte concentration of the human serum used for dilution has to be taken into account.

Expected values

Due to different standardizations between methods, result variation may arise. Clinical assessment should be taken into consideration when interpreting results.

Health based reference values (recommended for use):

Currently there is no standard definition of the optimal vitamin D status. Many specialists consider the commonly used population based reference values too low. Health based reference values are recommended to replace population based reference values.[17]

Most experts agree that vitamin D deficiency should be defined as vitamin D (25-OH) of ≤ 20 ng/mL (≤ 50 nmol/L).[18] Vitamin D insufficiency is recognized as 21-29 ng/mL.[18] Similarly, the US National Kidney Foundation considers levels < 30 ng/mL to be insufficient or deficient.[19]

The preferred level for vitamin D (25-OH) by many experts is now recommended to be ≥ 30 ng/mL (≥ 75 nmol/L).[18,20,21,22]

Reference values measured in an apparently healthy population:

It should be taken into consideration that differences in vitamin D (25-OH) levels may exist with respect to gender, age, season, geographical latitude and ethnic groups.[18,20]

Each laboratory should investigate the transferability of the expected values to its own patient population and if necessary determine its own reference ranges.

Population based reference ranges should not be taken as clinical cutoff to recommend or dissuade from vitamin D supplementation. Guidance for supplementation should be taken from recent literature.[18,19]

A reference range study was conducted with samples from apparently healthy individuals of Caucasian heritage. The age range was 20-77 years. Samples were collected between November and July in northern Germany.

The values given are for information only and may vary from other published data.

			Gender			
	All (n = 453)		Female (n = 252)		Male (n = 201)	
Unit	ng/mL	nmol/L	ng/mL	nmol/L	ng/mL	nmol/L
Mean	20.6	51.5	21.6	54.0	19.4	48.5
2.5th percentile	5.26	13.2	6.23	15.6	4.92	12.3
97.5th percentile	47.0	118	49.9	125	42.7	107

A lower recovery may be found in particular clinical cohorts, for example dialysis patients.[23]

Specific performance data

Representative performance data on the analyzers are given below. Results obtained in individual laboratories may differ.

Woldeyohannes M, *et al. BMJ Open* 2023; 13:e067641. doi: 10.1136/bmjopen-2022-067641

ms_05894913190V7.0

Vitamin D total

cobas®

25-Hydroxyvitamin D

Precision

Precision was determined using Elecsys reagents, pooled human sera and controls in a protocol (EP5-A2) of the CLSI (Clinical and Laboratory Standards Institute): 2 runs per day in duplication each for 21 days (n = 84). The following results were obtained:

Elecsys 2010 and **cobas e** 411 analyzers					
			**Repeatability**		
Sample	Mean		SD		CV
	ng/mL	nmol/L	ng/mL	nmol/L	%
HS[a] 1	6.76	16.9	0.525	1.31	7.8
HS 2	15.0	37.5	0.770	1.93	5.1
HS 3	28.0	70.0	0.860	2.15	3.1
HS 4	67.0	168	1.15	2.88	1.7
PC[b] Varia 1	19.9	49.8	0.948	2.37	4.8
PC Varia 2	38.3	95.8	1.05	2.63	2.7

a) HS = human serum

b) PC = PreciControl

Elecsys 2010 and **cobas e** 411 analyzers					
			**Intermediate precision**		
Sample	Mean		SD		CV
	ng/mL	nmol/L	ng/mL	nmol/L	%
HS 1	6.76	16.9	0.724	1.81	10.7
HS 2	15.0	37.5	1.28	3.20	8.5
HS 3	28.0	70.0	1.46	3.65	5.2
HS 4	67.0	168	1.46	3.65	2.2
PC Varia 1	19.9	49.8	1.23	3.08	6.2
PC Varia 2	38.3	95.8	1.41	3.53	3.7

MODULAR ANALYTICS E170, **cobas e** 601 and **cobas e** 602 analyzers					
			**Repeatability**		
Sample	Mean		SD		CV
	ng/mL	nmol/L	ng/mL	nmol/L	%
HS 1	8.35	20.9	0.567	1.42	6.8
HS 2	15.8	39.5	0.824	2.06	5.2
HS 3	28.3	70.8	1.11	2.78	3.9
HS 4	69.6	174	1.50	3.75	2.2
PC Varia 1	20.2	50.5	0.924	2.31	4.6
PC Varia 2	39.6	99.0	1.06	2.65	2.7

MODULAR ANALYTICS E170, **cobas e** 601 and **cobas e** 602 analyzers					
			**Intermediate precision**		
Sample	Mean		SD		CV
	ng/mL	nmol/L	ng/mL	nmol/L	%
HS 1	8.35	20.9	1.10	2.75	13.1
HS 2	15.8	39.5	1.18	2.95	7.5
HS 3	28.3	70.8	1.83	4.58	6.5
HS 4	69.6	174	2.37	5.93	3.4
PC Varia 1	20.2	50.5	0.954	2.39	4.7
PC Varia 2	39.6	99.0	1.38	3.45	3.5

Method comparison

1) A comparison of the Elecsys Vitamin D total assay (y) using samples measured with LC-MS/MS (x) gave the following correlations (ng/mL):

Number of samples measured: 903

Passing/Bablok[24] y = 1.09x - 0.510

Pearson r = 0.894

The sample concentrations were between approximately 3 ng/mL (7.5 nmol/L) and 81 ng/mL (203 nmol/L).

2) A comparison of the Elecsys Vitamin D total assay (y) using samples measured with a commercially available vitamin D (25-OH) immunoassay (x) gave the following correlations (ng/mL):

Number of samples measured: 451

Passing/Bablok[24] y = 1.29x + 1.71

Pearson r = 0.803

The sample concentrations were between approximately 5 ng/mL (12.5 nmol/L) and 81 ng/mL (203 nmol/L).

Analytical specificity

The specificity was assessed at 50 % B_0 and the results are summarized in the following table:

Cross-reactant	Cross-reactivity (%)
25-hydroxyvitamin D_3	100
25-hydroxyvitamin D_2	92
24,25-dihydroxyvitamin D_3	149
C3-epimer of 25-hydroxyvitamin D_3	91
1,25-dihydroxyvitamin D_3	non detectable
1,25-dihydroxyvitamin D_2	non detectable
Vitamin D_3	non detectable
Vitamin D_2	non detectable

Functional sensitivity

The functional sensitivity is the lowest analyte concentration that can be reproducibly measured with an intermediate precision CV of ≤ 20 %.
8 samples with concentrations between 0.722 ng/mL and 10.1 ng/mL were measured on several days. The functional sensitivity was determined to be 4.01 ng/mL (CV 18.5 %).

References

1 Holick M. Vitamin D: the underappreciated D-lightful hormone that is important for skeletal and cellular health. Curr Opin Endocrinol Diabetes 2002;9(1)87-98.

2 Holick MF. Vitamin D deficiency. N Engl J Med 2007;357:266-281.

3 Houghton LA, Vieth R. The case against ergocalciferol (vitamin D2) as a vitamin supplement. Am J Clin Nutr 2006;84:694-697.

4 Hart GR, Furniss JL, Laurie D, et al. Measurement of vitamin D Status: background, clinical use and methodologies. Clin Lab 2006;52(7-8):335-343.

5 Armas LAG, Hollis BW, Heaney RP. Vitamin D2 is much less effective than Vitamin D3 in humans. J Clin Endocrinol Metab 2004;89(11):5387-5391.

6 Steingrimsdottir L, Gunnarsson O, Indridason OS, et al. Relationship between serum parathyroid hormone levels, vitamin D sufficiency, and calcium intake. JAMA 2005 Nov 9;294(18):2336-2341.

7 Venning G. Recent developments in vitamin D deficiency and muscle weakness among elderly people. BMJ 2005;330:524-526.

8 Lips P. Vitamin D deficiency and secondary hyperparathyroidism in the elderly: consequences for bone loss and fractures and therapeutic implications. Endocr Rev 2001 Aug;22(4):447-501.

9 Souberbielle JC, Lawson-Body E, Hammadi B, et al. The use in clinical practice of parathyroid hormone normative values established in vitamin D-sufficient subjects. J Clin Endocrinol Metab 2003 Aug;88(8):3501-3504.

10 Willett AM. Vitamin D status and its relationship with parathyroid hormone and bone mineral status in older adolescents. Proceeding of the Nutrition Society 2005;64:193-203.

ms_05894913190V7.0

Vitamin D total

cobas®

25-Hydroxyvitamin D

11 Kuchukk NO, van Schoor NM, Pluijm SM, et al. Vitamin D status, parathyroid function, bone turnover, and BMD in postmenopausal women with osteoporosis: global perspective. J Bone Miner Res 2009;24:693-701.

12 Occupational Safety and Health Standards: Bloodborne pathogens. (29 CFR Part 1910.1030). Fed. Register.

13 Directive 2000/54/EC of the European Parliament and Council of 18 September 2000 on the protection of workers from risks related to exposure to biological agents at work.

14 Lewis JG, Elder PA. Serum 25-OH Vitamin D2 and D3 are Stable under Exaggerated Conditions. Clin Chem 2008;54:1931-1932.

15 Vogeser M, Kyriatsoulis A, Huber E, et al. Candidate Reference Method for the Quantification of Circulating 25-Hydroxyvitamin D3 by Liquid Chromatography-Tandem Mass Spectrometry. Clin Chem 2004;50:1415-1417.

16 Phinney KW. Development of a standard reference material for vitamin D in serum. Am J Clin Nutr 2008;88(suppl):511-512.

17 Bischoff-Ferrari HA, Giovannucci E, Willett WC, et al. Estimation of optimal serum concentrations of 25-hydroxyvitamin D for multiple health outcomes. Am J Clin Nutr 2006;84:18-28.

18 Holick MF. Vitamin D status: measurement, interpretation, and clinical application. Ann Epidemiol 2009;19(2):73-78.

19 KDOQI Clinical Practice Guidelines for Bone Metabolism and Disease in Children With Chronic Kidney Disease. http://www.kidney.org/PROFESSIONALS/kdoqi/guidelines_pedbone/guide8.htm

20 Souberbielle JC, Body JJ, Lappe JM, et al. Vitamin D and musculoskeletal health, cardiocascular disease, autoimmunity and cancer: Recommendations for clinical practice. Autoimmun Rev 2010;9:709-715.

21 Dawson-Hughes B, Heaney RP, Holick MF, et al. Estimates of optimal vitamin D status. Osteoporos Int 2005;16:713-716.

22 Vieth R. Why the minimum desirable serum 25-hydroxyvitamin D level should be 75 nmol/L (30 ng/mL). Best Pract Res Clin Endocrinol Metab 2011;25(4):681-691.

23 Heijboer AC, Blankenstein MA, Kema IP, et al. Accuracy of 6 routine 25-hydroxyvitamin D assays: influence of vitamin D binding protein concentration. Clin Chem 2012;58:543-548.

24 Bablok W, Passing H, Bender R, et al. A general regression procedure for method transformation. Application of linear regression procedures for method comparison studies in clinical chemistry, Part III. J Clin Chem Clin Biochem 1988 Nov;26(11):783-790.

For further information, please refer to the appropriate operator's manual for the analyzer concerned, the respective application sheets, the product information and the Method Sheets of all necessary components (if available in your country).

A point (period/stop) is always used in this Method Sheet as the decimal separator to mark the border between the integral and the fractional parts of a decimal numeral. Separators for thousands are not used.

Symbols

Roche Diagnostics uses the following symbols and signs in addition to those listed in the ISO 15223-1 standard.

CONTENT	Contents of kit
SYSTEM	Analyzers/Instruments on which reagents can be used
REAGENT	Reagent
CALIBRATOR	Calibrator
→	Volume after reconstitution or mixing
GTIN	Global Trade Item Number

COBAS, COBAS E, ELECSYS and PRECICONTROL are trademarks of Roche. INTRALIPID is a trademark of Fresenius Kabi AB.

All other product names and trademarks are the property of their respective owners.

Additions, deletions or changes are indicated by a change bar in the margin.

© 2015, Roche Diagnostics

CE

Roche Diagnostics GmbH, Sandhofer Strasse 116, D-68305 Mannheim
www.roche.com

Roche
