# Supplementary material for: Ethiopia National Food and Nutrition Survey to inform the Ethiopian National Food and Nutrition Strategy: a study protocol
Source: BMJ Open. 2023 Apr 25;13(4):e067641. doi: 10.1136/bmjopen-2022-067641 (PMC10151871; doi:10.1136/bmjopen-2022-067641)
Supplement: Supplementary data [file bmjopen-2022-067641supp006.pdf]

ms_07212771190V1.0

Vitamin B12 II

Vitamin B12

REF	Σ	SYSTEM
**07212771** 190	100	Elecsys 2010<br>MODULAR ANALYTICS E170<br>**cobas e** 411<br>**cobas e** 601<br>**cobas e** 602

English

Intended use

Binding assay for the in vitro quantitative determination of vitamin B12 in human serum and plasma.

The **e**lectro**c**hemi**l**uminescence **i**mmuno**a**ssay “ECLIA” is intended for use on Elecsys and **cobas e** immunoassay analyzers.

Summary

Vitamin B12, also referred to as cobalamin, is a complex organometallic compound in which a cobalt atom is situated within a corrin ring. It is a water-soluble vitamin which is synthesized by microorganisms. It cannot be synthesized in the human body and is seldom found in products of plant origin. Main sources of vitamin B12 are meat, fish, eggs and dairy products.[1] The uptake in the gastrointestinal tract depends on intrinsic factor, which is synthesized by the gastric parietal cells, and on the “cubam receptor” in the distal ileum. The most frequent cause of severe vitamin B12 deficiency is a lack of intrinsic factor due to autoimmune atrophic gastritis. The disease is historically called “pernicious anemia”, even though many patients present with mainly neurologic manifestations. Examples of other causes for vitamin B12 deficiency are malabsorption due to gastrectomy, inflammatory bowel disease or dietary deficiency, e.g. in strict vegetarians (vegans).[2]

Vitamin B12 is the cofactor for two enzymes, methionine synthase and methylmalonyl CoA mutase.[2,3] Methionine synthase, located in the cytoplasm, requires vitamin B12 in the form of methylcobalamin and catalyzes the conversion of homocysteine to methionine, an essential amino acid. During this step a methyl group is transferred from methyltetrahydrofolate to the amino acid.[3] This enzyme links the methylation pathway through synthesis of the methyl donor S-Adenosyl methionine and the pathway in which purine and pyrimidine are synthesized via generation of tetrahydrofolate.[3] In the form of 5’-deoxyadenosylcobalamin, vitamin B12 is also required for the mitochondrial enzyme methylmalonyl CoA mutase, which converts methylmalonyl CoA to succinyl CoA. This is a step in the oxidation of odd-chain fatty acids and catabolism of ketogenic amino acids.[3] Thus, vitamin B12 is important for DNA synthesis, regenerating methionine for protein synthesis and methylation, as well as for the development and initial myelination of the central nervous system (CNS) and for the maintenance of normal CNS function.[2,3]

Vitamin B12 deficiencies are common in wealthier countries principally among the elderly and are most prevalent in poorer populations. In general the prevalence increases with age.[4,5]

Vitamin B12 deficiency impacts red blood cell synthesis, resulting in megaloblastic anemia due to abnormal DNA synthesis.[3] In addition it impairs neurological function, in particular demyelination of nerves in part due to abnormal methylation, leading to peripheral neuropathy, dementia, poor cognitive performance, and depression.[3] Other effects of vitamin B12 deficiency or depletion are increased risk of neural tube defects, osteoporosis, cerebrovascular and cardiovascular diseases.[3] Early diagnosis is essential, because of the latent nature of this disorder and the risk of permanent neurological damage.[3,5]

Generally, the primary test performed to confirm the diagnosis of vitamin B12 deficiency is measurement of serum vitamin B12 level.[2] Recent publications suggest that in addition the following biomarkers should be measured to improve the specificity of diagnosis: folate, methylmalonic acid (MMA), homocysteine and holotranscobalamin.[2,5,6,7]

The Elecsys Vitamin B12 II assay employs a competitive test principle using intrinsic factor specific for vitamin B12. Vitamin B12 in the sample competes with the added vitamin B12 labeled with biotin for the binding sites on the ruthenium-labeled intrinsic factor complex[a)].

a) Tris(2,2'-bipyridyl)ruthenium(II)-complex (Ru(bpy)$_3^{2+}$)

Test principle

Competition principle. Total duration of assay: 27 minutes.

- 1st incubation: By incubating the sample (15 µL) with the vitamin B12 pretreatment 1 and pretreatment 2, bound vitamin B12 is released.
- 2nd incubation: By incubating the pretreated sample with the ruthenium labeled intrinsic factor, a vitamin B12-binding protein complex is formed, the amount of which is dependent upon the analyte concentration in the sample.
- 3rd incubation: After addition of streptavidin-coated microparticles and vitamin B12 labeled with biotin, the still-vacant sites of the ruthenium labeled intrinsic factor become occupied, with formation of a ruthenium labeled intrinsic factor vitamin B12 biotin complex. The entire complex becomes bound to the solid phase via interaction of biotin and streptavidin.
- The reaction mixture is aspirated into the measuring cell where the microparticles are magnetically captured onto the surface of the electrode. Unbound substances are then removed with ProCell/ProCell M. Application of a voltage to the electrode then induces chemiluminescent emission which is measured by a photomultiplier.
- Results are determined via a calibration curve which is instrument-specifically generated by 2-point calibration and a master curve provided via the reagent barcode.

Reagents - working solutions

The reagent rackpack (M, R1, R2) and the pretreatment reagents (PT1, PT2) are labeled as B12 II.

PT1 Pretreatment reagent 1 (white cap), 1 bottle, 4 mL:
Dithiothreitol 1.028 g/L; stabilizer, pH 5.5.

PT2 Pretreatment reagent 2 (gray cap), 1 bottle, 4 mL:
Sodium hydroxide 40 g/L; sodium cyanide 2.205 g/L.

M Streptavidin-coated microparticles (transparent cap), 1 bottle, 6.5 mL:
Streptavidin-coated microparticles 0.72 mg/mL; preservative.

R1 Intrinsic factor~Ru(bpy)$_3^{2+}$ (gray cap), 1 bottle, 10 mL:
Ruthenium labeled recombinant porcine intrinsic factor 4 µg/L; cobinamide dicyanide 15 µg/L; stabilizer; human serum albumin; phosphate buffer, pH 5.5; preservative.

R2 Vitamin B12~biotin (black cap), 1 bottle, 8.5 mL:
Biotinylated vitamin B12 25 µg/L; biotin 3 µg/L; phosphate buffer, pH 7.0; preservative.

Precautions and warnings

For in vitro diagnostic use.
Exercise the normal precautions required for handling all laboratory reagents.
Disposal of all waste material should be in accordance with local guidelines.
Safety data sheet available for professional user on request.

This kit contains components classified as follows in accordance with the Regulation (EC) No. 1272/2008:

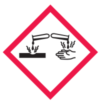

Danger

Vitamin B12 II

cobas®

Vitamin B12

H290	May be corrosive to metals.
H314	Causes severe skin burns and eye damage.
H412	Harmful to aquatic life with long lasting effects.

Prevention:

P234	Keep only in original container.
P264	Wash skin thoroughly after handling.
P273	Avoid release to the environment.
P280	Wear protective gloves/ protective clothing/ eye protection/ face protection.

Response:

P301 + P330 + P331	IF SWALLOWED: Rinse mouth. Do NOT induce vomiting.
P303 + P361 + P353	IF ON SKIN (or hair): Remove/Take off immediately all contaminated clothing. Rinse skin with water/shower.
P304 + P340	IF INHALED: Remove person to fresh air and keep comfortable for breathing.
P305 + P351 + P338	IF IN EYES: Rinse cautiously with water for several minutes. Remove contact lenses, if present and easy to do. Continue rinsing.
P310	Immediately call a POISON CENTER or doctor/physician.
P363	Wash contaminated clothing before reuse.
P390	Absorb spillage to prevent material damage.

Storage:

P405	Store locked up.
P406	Store in corrosive resistant stainless steel container with a resistant inner liner.

Disposal:

P501	Dispose of contents/container to an approved waste disposal plant.

Product safety labeling primarily follows EU GHS guidance.

Contact phone: all countries: +49-621-7590

All human material should be considered potentially infectious. All products derived from human blood are prepared exclusively from the blood of donors tested individually and shown to be free from HBsAg and antibodies to HCV and HIV. The testing methods applied were FDA-approved or cleared in compliance with the European Directive 98/79/EC, Annex II, List A.

However, as no testing method can rule out the potential risk of infection with absolute certainty, the material should be handled with the same level of care as a patient specimen. In the event of exposure, the directives of the responsible health authorities should be followed.[8,9]

Avoid foam formation in all reagents and sample types (specimens, calibrators and controls).

Reagent handling

The reagents in the kit have been assembled into a ready-for-use unit that cannot be separated.

All information required for correct operation is read in from the respective reagent barcodes.

Storage and stability

Store at 2-8 °C.

Do not freeze.

Store the Elecsys reagent kit **upright** in order to ensure complete availability of the microparticles during automatic mixing prior to use.

Stability:	
unopened at 2-8 °C	up to the stated expiration date
after opening at 2-8 °C	84 days (12 weeks)
on the analyzers	35 days (5 weeks) onboard<br>or<br>60 days when stored alternatively in the refrigerator and on the analyzer, with the total time onboard on the analyzer not exceeding<br>10 x 8 hours

Specimen collection and preparation

Only the specimens listed below were tested and found acceptable.

Serum collected using standard sampling tubes or tubes containing separating gel.

Na-heparin, Li-heparin, K_2-EDTA and K_3-EDTA plasma. Li-heparin plasma tubes containing separating gel can be used.

Criterion: Slope 0.9-1.1 + intercept within < ± 2x Limit of Blank (LoB) + coefficient of correlation ≥ 0.95.

Stable for 2 hours at 15-25 °C, 48 hours at 2-8 °C, 56 days at (-15)-(-25) °C. Freeze once only.

Stability of serum obtained with separating tubes: 24 hours at 2-8 °C (note the data provided by the tube manufacturer).

The sample types listed were tested with a selection of sample collection tubes that were commercially available at the time of testing, i.e. not all available tubes of all manufacturers were tested. Sample collection systems from various manufacturers may contain differing materials which could affect the test results in some cases. When processing samples in primary tubes (sample collection systems), follow the instructions of the tube manufacturer.

Centrifuge samples containing precipitates before performing the assay.

Do not use heat-inactivated samples.

Avoid hemolysis.

Do not use samples and controls stabilized with azide.

Vitamin B12 determinations should be performed on serum or plasma samples from fasting patients.

Note: Samples with extremely high total protein concentrations (e.g. patients suffering from Waldenström's macroglobulinemia) are not suitable for use in this assay, since they may lead to the formation of protein gel in the assay cup. Processing protein gel may cause a run abort. The critical protein concentration is dependent upon the individual sample composition. The formation of protein gel was seen in samples (spiked with human IgG or human serum albumin) having a total protein concentration > 160 g/L.

Ensure the samples, calibrators and controls are at 20-25 °C prior to measurement.

Due to possible evaporation effects, samples, calibrators and controls on the analyzers should be analyzed/measured within 2 hours.

Materials provided

See “Reagents – working solutions” section for reagents.

Materials required (but not provided)

- REF 07212780190, Vitamin B12 II CalSet, for 4 x 1 mL
- REF 05618860190, PreciControl Varia, for 2 x 3 mL each of PreciControl Varia 1 and 2
- REF 11732277122, Diluent Universal, 2 x 16 mL sample diluent or REF 03183971122, Diluent Universal, 2 x 36 mL sample diluent
- General laboratory equipment
- Elecsys 2010, MODULAR ANALYTICS E170 or **cobas e** analyzer

Accessories for Elecsys 2010 and **cobas e** 411 analyzers:

- REF 11662988122, ProCell, 6 x 380 mL system buffer
- REF 11662970122, CleanCell, 6 x 380 mL measuring cell cleaning solution

ms_07212771190V1.0

Vitamin B12 II

Vitamin B12

- REF 11930346122, Elecsys SysWash, 1 x 500 mL washwater additive
- REF 11933159001, Adapter for SysClean
- REF 11706802001, Elecsys 2010 AssayCup, 60 x 60 reaction vessels
- REF 11706799001, Elecsys 2010 AssayTip, 30 x 120 pipette tips

Accessories for MODULAR ANALYTICS E170, **cobas e** 601 and **cobas e** 602 analyzers:

- REF 04880340190, ProCell M, 2 x 2 L system buffer
- REF 04880293190, CleanCell M, 2 x 2 L measuring cell cleaning solution
- REF 03023141001, PC/CC-Cups, 12 cups to prewarm ProCell M and CleanCell M before use
- REF 03005712190, ProbeWash M, 12 x 70 mL cleaning solution for run finalization and rinsing during reagent change
- REF 03004899190, PreClean M, 5 x 600 mL detection cleaning solution
- REF 12102137001, AssayTip/AssayCup Combimagazine M, 48 magazines x 84 reaction vessels or pipette tips, waste bags
- REF 03023150001, WasteLiner, waste bags
- REF 03027651001, SysClean Adapter M

Accessories for all analyzers:

- REF 11298500316, ISE Cleaning Solution/Elecsys SysClean, 5 x 100 mL system cleaning solution

Assay

For optimum performance of the assay follow the directions given in this document for the analyzer concerned. Refer to the appropriate operator's manual for analyzer-specific assay instructions.

Resuspension of the microparticles takes place automatically prior to use. Read in the test-specific parameters via the reagent barcode. If in exceptional cases the barcode cannot be read, enter the 15-digit sequence of numbers.

MODULAR ANALYTICS E170, **cobas e** 601 and **cobas e** 602 analyzers: PreClean M solution is necessary.

Bring the cooled reagents to approximately 20 °C and place on the reagent disk (20 °C) of the analyzer. Avoid foam formation. The system automatically regulates the temperature of the reagents and the opening/closing of the bottles.

Calibration

Traceability: This method has been standardized against the Vitamin B12 assay (REF 04745736).

Every Elecsys reagent set has a barcoded label containing specific information for calibration of the particular reagent lot. The predefined master curve is adapted to the analyzer using the relevant CalSet.

Calibration frequency: Calibration must be performed once per reagent lot using fresh reagent (i.e. not more than 24 hours since the reagent kit was registered on the analyzer). Renewed calibration is recommended as follows:

- after 1 month (28 days) when using the same reagent lot
- after 7 days (when using the same reagent kit on the analyzer)
- as required: e.g. quality control findings outside the defined limits

Quality control

For quality control, use PreciControl Varia.

In addition, other suitable control material can be used.

Controls for the various concentration ranges should be run individually at least once every 24 hours when the test is in use, once per reagent kit, and following each calibration.

The control intervals and limits should be adapted to each laboratory's individual requirements. Values obtained should fall within the defined limits. Each laboratory should establish corrective measures to be taken if values fall outside the defined limits.

Follow the applicable government regulations and local guidelines for quality control.

Calculation

The analyzer automatically calculates the analyte concentration of each sample (either in pmol/L or pg/mL).

Conversion factors:	pmol/L x 1.36 = pg/mL
	pg/mL x 0.738 = pmol/L

Limitations - interference

The assay is unaffected by icterus (bilirubin ≤ 1112 µmol/L or ≤ 65 mg/dL), hemolysis (Hb ≤ 0.025 mmol/L or ≤ 0.04 g/dL), lipemia (triglycerides ≤ 17.1 mmol/L or ≤ 1500 mg/dL), biotin (≤ 205 nmol/L or ≤ 50 ng/mL), IgG ≤ 28 g/L, IgA ≤ 16 g/L and IgM ≤ 10 g/L.

Criterion: Recovery within ± 10 % of initial value with samples > 200 pg/mL and ≤ ± 20 pg/mL with samples ≤ 200 pg/mL.

Samples should not be taken from patients receiving therapy with high biotin doses (i.e. > 5 mg/day) until at least 8 hours following the last biotin administration.

No interference was observed from rheumatoid factors up to a concentration of 1500 IU/mL.

In vitro tests were performed on 16 commonly used pharmaceuticals. No interference with the assay was found.

In rare cases, interference due to extremely high titers of antibodies to analyte-specific antibodies, streptavidin or ruthenium can occur. These effects are minimized by suitable test design.

Because intrinsic factor is typically used as the binding protein in serum vitamin B12 assays, anti-intrinsic factor antibodies (which are common in pernicious anemia) can lead to elevated vitamin B12 measurement values.[2,10,11] The Elecsys Vitamin B12 II assay is designed to avoid interference due to anti-intrinsic factor antibodies.[12]

For diagnostic purposes, the results should always be assessed in conjunction with the patient's medical history, clinical examination and other findings.

Note: The presence of immunoglobulin-vitamin B12 complexes may cause unexpectedly high values of vitamin B12.[13,14]

Limits and ranges

Measuring range

50.0-2000 pg/mL or 36.9-1476 pmol/L (defined by the Limit of Blank and the maximum of the master curve). Values below the Limit of Blank are reported as < 50.0 pg/mL or < 36.9 pmol/L. Values above the measuring range are reported as > 2000 pg/mL or > 1476 pmol/L.

Lower limits of measurement

Limit of Blank (LoB), Limit of Detection (LoD) and Limit of Quantitation (LoQ)

Limit of Blank = 50 pg/mL (36.9 pmol/L)

Limit of Detection = 100 pg/mL (73.8 pmol/L)

Limit of Quantitation = 150 pg/mL (111 pmol/L) with a allowable imprecision of ≤ 20 %

The Limit of Blank, Limit of Detection and Limit of Quantitation were determined in accordance with the CLSI (Clinical and Laboratory Standards Institute) EP17-A requirements.

The Limit of Blank is the 95th percentile value from n ≥ 60 measurements of analyte-free samples over several independent series. The Limit of Blank corresponds to the concentration below which analyte-free samples are found with a probability of 95 %.

The Limit of Detection is determined based on the Limit of Blank and the standard deviation of low concentration samples. The Limit of Detection corresponds to the lowest analyte concentration which can be detected (value above the Limit of Blank with a probability of 95 %).

The Limit of Quantitation is defined as the lowest amount of analyte in a sample that can be accurately quantitated with a allowable imprecision of ≤ 20 %.

It has been determined using low concentration vitamin B12 samples.

Dilution

Samples with vitamin B12 concentrations above the measuring range can be manually diluted 1:2 with Diluent Universal. The concentration of the diluted sample must be > 738 pmol/L or > 1000 pg/mL. After manual dilution, multiply the results by the dilution factor 2.

Note: Sample-dependent non-linearity upon dilution is seen with samples having analyte levels beyond the measuring range. As Diluent Universal may contain low levels of endogenous vitamin B12, it is recommended that linearity studies be performed using a known low analyte-containing serum

Woldeyohannes M, *et al. BMJ Open* 2023; 13:e067641. doi: 10.1136/bmjopen-2022-067641

Supplemental material BMJ Publishing Group Limited (BMJ) disclaims all liability and responsibility arising from any reliance placed on this supplemental material which has been supplied by the author(s) BMJ Open

pool. Samples outside the measuring range can be diluted 1:2 with Diluent Universal; the effect of endogenous vitamin B12 concentration is insignificant at these levels.

Expected values

Because differences may exist with respect to population and dietary status, it is recommended that normal ranges be determined by each laboratory over a suitable period of time and in a statistically significant number of assays before clinical significance is attached to the results of these tests.

The values shown below were performed on samples from an apparently healthy population, using the Elecsys Vitamin B12 II assay. The calculation is based on 135 sera (68 men, 67 women). The age range was between 20 and 78 years. Pregnant women were excluded. The reference population was selected according to normal homocysteine values.

N	Median		Range (2.5^{th}-97.5^{th} percentile)	
	pg/mL	pmol/L	pg/mL	pmol/L
135	425	314	197-771	145-569

These values should only be used as guidelines.

Each laboratory should investigate the transferability of the expected values to its own patient population and if necessary determine its own reference ranges.

Specific performance data

Representative performance data on the analyzers are given below. Results obtained in individual laboratories may differ.

Precision

Precision was determined using Elecsys reagents, pooled human sera and controls in a protocol (EP5-A2) of the CLSI (Clinical and Laboratory Standards Institute): 2 runs per day in duplication each for 21 days (n = 84). The following results were obtained:

Elecsys 2010 and **cobas e** 411 analyzers					
		**Repeatability**		**Intermediate precision**	
Sample	Mean	SD	CV	SD	CV
	pg/mL	pg/mL	%	pg/mL	%
Human serum 1	176	8.86	5.0	12.7	7.2
Human serum 2	405	13.0	3.2	17.5	4.3
Human serum 3	960	19.7	2.1	31.0	3.2
Human serum 4	1230	27.4	2.2	46.4	3.8
Human serum 5	1940	40.9	2.1	72.6	3.7
PreciControl Varia1	447	12.2	2.7	18.6	4.2
PreciControl Varia2	934	20.2	2.2	38.4	4.1

Elecsys 2010 and **cobas e** 411 analyzers					
		**Repeatability**		**Intermediate precision**	
Sample	Mean	SD	CV	SD	CV
	pmol/L	pmol/L	%	pmol/L	%
Human serum 1	130	6.54	5.0	9.37	7.2
Human serum 2	299	9.59	3.2	12.9	4.3
Human serum 3	708	14.5	2.1	22.9	3.2
Human serum 4	908	20.2	2.2	34.2	3.8
Human serum 5	1432	30.2	2.1	53.6	3.7
PreciControl Varia1	330	9.00	2.7	13.7	4.2
PreciControl Varia2	689	14.9	2.2	28.3	4.1

MODULAR ANALYTICS E170, **cobas e** 601 and **cobas e** 602 analyzers					
		**Repeatability**		**Intermediate precision**	
Sample	Mean	SD	CV	SD	CV
	pg/mL	pg/mL	%	pg/mL	%
Human serum 1	176	5.84	3.3	9.14	5.2
Human serum 2	407	8.24	2.0	12.7	3.1
Human serum 3	1010	13.2	1.3	21.1	2.1
Human serum 4	1230	19.8	1.6	28.8	2.3
Human serum 5	1890	29.8	1.6	41.5	2.2
PreciControl Varia1	448	7.16	1.6	15.3	3.4
PreciControl Varia2	917	12.0	1.3	27.8	3.0

MODULAR ANALYTICS E170, **cobas e** 601 and **cobas e** 602 analyzers					
		**Repeatability**		**Intermediate precision**	
Sample	Mean	SD	CV	SD	CV
	pmol/L	pmol/L	%	pmol/L	%
Human serum 1	130	4.31	3.3	6.75	5.2
Human serum 2	300	6.08	2.0	9.37	3.1
Human serum 3	745	9.74	1.3	15.6	2.1
Human serum 4	908	14.6	1.6	21.3	2.3
Human serum 5	1395	22.0	1.6	30.6	2.2
PreciControl Varia1	331	5.28	1.6	11.3	3.4
PreciControl Varia2	677	8.86	1.3	20.5	3.0

Method comparison

a) A comparison of the Elecsys Vitamin B12 assay (calibrated with Vitamin B12 CalSet II; x) and the Elecsys Vitamin B12 II assay (calibrated with Vitamin B12 II CalSet; y) using clinical samples gave the following correlations (pg/mL):

Number of samples measured: 100

Passing/Bablok[15]	Linear regression
y = 0.952x + 15.1	y = 0.957x + 11.6
τ = 0.977	r = 0.999

The sample concentrations were between 69 and 1890 pg/mL (51 and 1395 pmol/L).

b) A comparison of the Elecsys Vitamin B12 II assay (y) and a commercially available method (x) using clinical samples gave the following correlations (pg/mL):

Number of samples measured: 106

Passing/Bablok[15]	Linear regression
y = 0.923x + 4.90	y = 0.881x + 27.6
τ = 0.952	r = 0.993

The sample concentrations were between 182 and 1797 pg/mL (134 and 1326 pmol/L).

c) A comparison of the Elecsys Vitamin B12 II assay on the **cobas e** 601 analyzer (y) and the Elecsys Vitamin B12 II assay on the **cobas e** 411 analyzer (x) using clinical samples gave the following correlations (pg/mL):

Number of samples measured: 117

Passing/Bablok[15]	Linear regression
y = 1.01x - 2.77	y = 1.01x + 3.22
τ = 0.933	r = 0.995

The sample concentrations were between 56 and 1887 pg/mL (41 and 1393 pmol/L).

Woldeyohannes M, *et al. BMJ Open* 2023; 13:e067641. doi: 10.1136/bmjopen-2022-067641

ms_07212771190V1.0

Vitamin B12 II

cobas®

Vitamin B12

Analytical specificity

The following cross-reactivities were found, tested with vitamin B12 concentrations of 129 pg/mL and 550 pg/mL.

Cross-reactant	Maximum concentration tested ng/mL	Cross-reactivity %
Cobinamide dicyanide	210	0.003

References

1 Thomas L. Clinical Laboratory Diagnostics: Use and Assessment of Clinical Laboratory Results; 1st Edition, Frankfurt/Main: TH-Books-Verl.-Ges.,1998:424-431.
2 Stabler SP. Vitamin B12 deficiency. N Engl J Med 2013;368:149-160.
3 Allen LH. Vitamin B-12. Adv Nutr 2012;3(1):54-55. doi: 10.3945/an.111.001370. Epub 2012 Jan 5.
4 Allen LH. How common is vitamin B-12 deficiency? Am J Clin Nutr 2009;89(2):693S-696S. Epub 2008 Dec 30.
5 Chatthanawaree W. Biomarkers of cobalamin (vitamin B12) deficiency and its application. J Nutr Health Aging 2011 Mar;15(3):227-313.
6 Yetley EA, Pfeiffer CM, Phinney KW, et al., Biomarkers of vitamin B-12 status in NHANES: a roundtable summary. Am J Clin Nutr 2011 Jul;94(1):313S-321S.
7 Hvas AM, Nexo E. Diagnosis and treatment of vitamin B12 deficiency - an update. Haematologica 2006;91(11):1506-1512.
8 Occupational Safety and Health Standards: bloodborne pathogens. (29 CFR Part 1910.1030). Fed. Register.
9 Directive 2000/54/EC of the European Parliament and Council of 18 September 2000 on the protection of workers from risks related to exposure to biological agents at work.
10 Yang DT, Cook RJ. Spurious elevations of vitamin B12 with pernicious anemia. N Engl J Med 2012;366:1742-1743.
11 Carmel R, Agrawal YP. Failures of cobalamin assays in pernicious anemia. N Engl J Med 2012;367:385-386. [Erratum, N Engl J Med 2012;367:976.]
12 Schilling KA, Wiesgigl M. The Elecsys® Vitamin B12 assay is not affected by anti-intrinsic factor antibodies. Clin Chem Lab Med 2013 Jun 29;51(11):e251-e252.
13 Jeffery J, Millar H, MacKenzie P, et al. An IgG complexed form of vitamin B12 is a common cause of elevated serum concentrations. Clin Biochem 2010 Jan;43(1-2):82-88. doi: 10.1016/j.clinbiochem.2009.08.022. Epub 2009 Sep 8.
14 Bowen RA, Drake SK, Vanjani R, et al. Markedly increased vitamin B12 concentrations attributable to IgG-IgM-vitamin B12 immune complexes. Clin Chem 2006;52(11):2107-2114.
15 Bablok W, Passing H, Bender R, et al. A general regression procedure for method transformation. Application of linear regression procedures for method comparison studies in clinical chemistry, Part III. J Clin Chem Clin Biochem 1988 Nov;26(11):783-790.

For further information, please refer to the appropriate operator's manual for the analyzer concerned, the respective application sheets, the product information and the Method Sheets of all necessary components (if available in your country).

A point (period/stop) is always used in this Method Sheet as the decimal separator to mark the border between the integral and the fractional parts of a decimal numeral. Separators for thousands are not used.

Symbols

Roche Diagnostics uses the following symbols and signs in addition to those listed in the ISO 15223-1 standard.

CONTENT	Contents of kit
SYSTEM	Analyzers/Instruments on which reagents can be used
REAGENT	Reagent
CALIBRATOR	Calibrator
→	Volume after reconstitution or mixing
GTIN	Global Trade Item Number

COBAS, COBAS E, ELECSYS and PRECICONTROL are trademarks of Roche. INTRALIPID is a trademark of Fresenius Kabi AB.
All other product names and trademarks are the property of their respective owners.
Additions, deletions or changes are indicated by a change bar in the margin.
© 2015, Roche Diagnostics

CE

Roche Diagnostics GmbH, Sandhofer Strasse 116, D-68305 Mannheim
www.roche.com

Roche
