# Supplementary material for: Ethiopia National Food and Nutrition Survey to inform the Ethiopian National Food and Nutrition Strategy: a study protocol
Source: BMJ Open. 2023 Apr 25;13(4):e067641. doi: 10.1136/bmjopen-2022-067641 (PMC10151871; doi:10.1136/bmjopen-2022-067641)
Supplement: Supplementary data [file bmjopen-2022-067641supp007.pdf]

0107227841190c501V1.0

STFR2

Tina-quant Soluble Transferrin Receptor II

Order information

REF	CONTENT		Analyzer(s) on which **cobas c** pack(s) can be used
07227841190	Tina-quant Soluble Transferrin Receptor II(100 tests)	System-ID 07 7473 1	**cobas c** 311, **cobas c** 501/502
08753776190	Calibrator sTfR II (3 x 1 mL)	Codes 697	
08278202190	ControlSet sTfR II		
	Level I (3 x 1 mL)	Level I Code 153	
	Level II (3 x 1 mL)	Level II Code 154	
04489357190	Diluent NaCl 9 % (50 mL)	System-ID 07 6869 3	

English

System information

For **cobas c** 311/501 analyzers:

STFR2: ACN 439

For **cobas c** 502 analyzer:

STFR2: ACN 8439

Intended use

In vitro test for the quantitative determination of soluble transferrin receptor (sTfR) in human serum and plasma on **cobas c** systems.

Summary[1,2,3,4,5,6,7]

The transferrin receptor is an integral membrane glycoprotein having a molecular weight of 190 kilodalton (kDa). It consists of two identical subunits linked by disulfide bridges. Each of the monomers has an 85 kDa C-terminal component which can bind an iron-laden transferrin molecule. Proteolysis leads to the soluble form of the transferrin receptor (sTfR). In plasma, the soluble transferrin receptor is present in the form of a complex with transferrin having a molecular weight of approximately 320 kD. The serum concentration of sTfR is directly proportional to the concentration of the receptor on the membrane.

The uptake of iron by the body's cells is controlled by expression of the transferrin receptor (TfR). If the intracellular iron stores are exhausted - corresponding to a ferritin concentration of less than 12 µg/L - then more TfR is expressed. The affinity of the transferrin receptor to transferrin depends on the latter's loading state. As 80-95 % of the transferrin receptor molecules are localized on erythropoietic cells, the TfR concentration (and hence also the sTfR concentration) reflects the iron requirement of these cells. When iron deficiency exists, the sTfR concentration in serum rises even before the hemoglobin concentration is significantly depressed. The sTfR concentration can therefore describe the functional iron status while ferritin reflects the iron storage status. A precise assessment of the iron status can be obtained by determining the sTfR index
(= sTfR concentration/log ferritin concentration).

As - in contrast to ferritin - the concentration of sTfR is not affected by acute-phase reactions, acute liver function disorders or malignant tumors, it is possible to differentiate between anemia of chronic disease (ACD) and iron deficiency anemia (IDA). Elevated sTfR values are also found in polycythemia, hemolytic anemia, thalassemia, hereditary spherocytosis, sickle cell anemia, megaloblastic anemia, myelodysplastic syndrome and vitamin B_{12} deficiency. Elevated sTfR concentrations occur during pregnancy when there is a deficiency of functional iron.

Parameter	Change	IDA	ACD		IDA + ACD	
Ferritin	iron stores	↓	↑	—	or	↑
TIBC/TRSF	iron status	↑	↓	↑	or	—
Serum iron	iron status	↓	↓		↓	
sTfR	functional iron deficiency	↑	—		↑	

↓ decreased, ↑ increased, — unchanged

Test principle[8]

Particle enhanced immunoturbidimetric assay.

Human soluble transferrin receptor agglutinates with latex particles coated with anti-soluble transferrin receptor antibodies. The precipitate is determined photometrically.

Reagents - working solutions

R1 TRIS buffer: with bovine serum albumin; preservatives

R2 Latex particles coated with monoclonal anti-human sTfR antibodies (mouse) in glycine buffer; preservative

R1 is in position B and R2 is in position C.

Precautions and warnings

For in vitro diagnostic use.
Exercise the normal precautions required for handling all laboratory reagents.
Disposal of all waste material should be in accordance with local guidelines.
Safety data sheet available for professional user on request.

This kit contains components classified as follows in accordance with the Regulation (EC) No. 1272/2008:

Contains 2-methyl-2H-isothiazol-3-one hydrochloride

EUH 208 May produce an allergic reaction.

Product safety labeling follows EU GHS guidance.

Reagent handling

Ready for use

Carefully invert reagent container several times prior to use to ensure that the reagent components are mixed.

Storage and stability

STFR2

Shelf life at 2-8 °C:	See expiration date on **cobas c** pack label.
On-board in use and refrigerated on the analyzer:	26 weeks

Diluent NaCl 9 %

Shelf life at 2-8 °C:	See expiration date on **cobas c** pack label.
On-board in use and refrigerated on the analyzer:	12 weeks

Specimen collection and preparation

For specimen collection and preparation only use suitable tubes or collection containers.

Only the specimens listed below were tested and found acceptable.
Serum.
Plasma: Heparin (Li-, Na-, $NH4^{+}$-) plasma

The sample types listed were tested with a selection of sample collection tubes that were commercially available at the time of testing, i.e. not all available tubes of all manufacturers were tested. Sample collection systems from various manufacturers may contain differing materials which could affect the test results in some cases. When processing samples in primary tubes (sample collection systems), follow the instructions of the tube manufacturer.

Centrifuge samples containing precipitates before performing the assay.

See the limitations and interferences section for details about possible sample interferences.

Stability:	6 days at 15-25 °C
	15 days at 2-8 °C
	13 weeks at -20 °C (±5 °C) (freeze only once)

STFR2

Tina-quant Soluble Transferrin Receptor II

Sample stability claims were established by experimental data by the manufacturer or based on reference literature and only for the temperatures/time frames as stated in the method sheet. It is the responsibility of the individual laboratory to use all available references and/or its own studies to determine specific stability criteria for its laboratory.

Materials provided

See “Reagents – working solutions” section for reagents.

Materials required (but not provided)

- See “Order information” section
- General laboratory equipment

Assay

For optimum performance of the assay follow the directions given in this document for the analyzer concerned. Refer to the appropriate operator’s manual for analyzer-specific assay instructions.

The performance of applications not validated by Roche is not warranted and must be defined by the user.

Application for serum and plasma

cobas c 311 **test definition**

Assay type	2-Point End		
Reaction time/Assay points	10 / 8-21		
Wavelength (sub/main)	800/570 nm		
Reaction direction	Increase		
Unit	mg/L (mg/dL, nmol/L)		
Reagent pipetting		Diluent (H_2O)	
R1	100 µL	-	
R2	40 µL	-	
Sample volumes	*Sample*	*Sample dilution*	
		Sample	*Diluent (NaCl)*
Normal	2 µL	-	-
Decreased	2 µL	25 µL	75 µL
Increased	2 µL		

cobas c 501 **test definition**

Assay type	2-Point End		
Reaction time/Assay points	10 / 13-30		
Wavelength (sub/main)	800/570 nm		
Reaction direction	Increase		
Unit	mg/L (mg/dL, nmol/L)		
Reagent pipetting		Diluent (H_2O)	
R1	100 µL	-	
R2	40 µL	-	
Sample volumes	*Sample*	*Sample dilution*	
		Sample	*Diluent (NaCl)*
Normal	2 µL	-	-
Decreased	2 µL	25 µL	75 µL
Increased	2 µL		

cobas c 502 **test definition**

Assay type	2-Point End		
Reaction time/Assay points	10 / 13-30		
Wavelength (sub/main)	800/570 nm		
Reaction direction	Increase		
Unit	mg/L (mg/dL, nmol/L)		
Reagent pipetting		Diluent (H_2O)	
R1	100 µL	-	
R2	40 µL	-	
Sample volumes	*Sample*	*Sample dilution*	
		Sample	*Diluent (NaCl)*
Normal	2 µL	-	-
Decreased	2 µL	25 µL	75 µL
Increased	2 µL		

Calibration

Calibrators	S1: H_2O
	S2-S6: Calibrator sTfR II
Calibration mode	Non-linear
Calibration frequency	Full calibration<br>• after reagent lot change<br>• after 12 weeks on-board the analyzer<br>• after 6 months when using a single reagent lot<br>• as required following quality control procedures

Calibration interval may be extended based on acceptable verification of calibration by the laboratory.

Traceability: This method has been standardized against an in-house reference preparation.

Quality control

For quality control, use control materials as listed in the "Order information" section.

In addition, other suitable control material can be used.

The control intervals and limits should be adapted to each laboratory’s individual requirements. Values obtained should fall within the defined limits. Each laboratory should establish corrective measures to be taken if values fall outside the defined limits.

Follow the applicable government regulations and local guidelines for quality control.

Calculation

cobas c systems automatically calculate the analyte concentration of each sample in the unit mg/L (mg/dL, nmol/L)

Conversion factors:	mg/L x 11.8 = nmol/L[9,a)]
	mg/L x 0.1 = mg/dL

a) Based on a molecular mass of 85 kDa for circulating transferrin receptor.

Limitations – interference

Criterion: Recovery within ± 0.2 mg/L (2.36 nmol/L) of initial values of samples ≤ 2 mg/L (23.6 nmol/L) and within ± 10 % for samples > 2 mg/L.

Icterus:[10] No significant interference up to an I index of 60 for conjugated and unconjugated bilirubin (approximate conjugated and unconjugated bilirubin concentration: 1026 µmol/L or 60 mg/dL).

Hemolysis:[10] No significant interference up to an H index of 1000 (approximate hemoglobin concentration: 622 µmol/L or 1000 mg/dL).

Lipemia (Intralipid):[10] No significant interference up to an L index of 2000. There is poor correlation between the L index (corresponds to turbidity) and triglycerides concentration.

Rheumatoid factors: No significant interference from rheumatoid factors up to a concentration of 1200 IU/mL.

High dose hook-effect: No false result occurs up to an sTfR concentration of 80 mg/L (944 nmol/L).

The antibodies are specific for sTfR. There is no cross-reactivity with diferrotransferrin, apotransferrin or ferritin under the assay conditions.

Drugs: No interference was found at therapeutic concentrations using common drug panels.[11,12]

0107227841190c501V1.0

STFR2

cobas®

Tina-quant Soluble Transferrin Receptor II

In very rare cases, gammopathy, in particular type IgM (Waldenström's macroglobulinemia), may cause unreliable results.[13]

In very rare cases, patient samples may contain particle agglutinating proteins (e.g. heterophilic antibodies or antibodies due to abnormal immunoglobulin synthesis, such as gammopathies like MGUS* or Waldenström's macroglobulinemia) which may lead to incorrect low or high results with this assay. Correct results cannot be obtained by sample dilution and these samples should be analyzed by an alternative method.

*Monoclonal Gammopathy of unknown significance

For diagnostic purposes, the results should always be assessed in conjunction with the patient's medical history, clinical examination and other findings.

ACTION REQUIRED

Special Wash Programming: The use of special wash steps is mandatory when certain test combinations are run together on **cobas c** systems. The latest version of the carry-over evasion list can be found with the NaOHD-SMS-SmpCln1+2-SCCS Method Sheets. For further instructions refer to the operator's manual. **cobas c** 502 analyzer: All special wash programming necessary for avoiding carry-over is available via the **cobas** link, manual input is required in certain cases.

Where required, special wash/carry-over evasion programming must be implemented prior to reporting results with this test.

Limits and ranges

Measuring range

0.50-20.0 mg/L (5.9-236 nmol/L)

Determine samples having higher concentrations via the rerun function. Dilution of samples via the rerun function is a 1:4 dilution. Results from samples diluted using the rerun function are automatically multiplied by a factor of 4.

Lower limits of measurement

Limit of Blank, Limit of Detection and Limit of Quantitation

Limit of Blank	= 0.25 mg/L (2.95 nmol/L)
Limit of Detection	= 0.40 mg/L (4.72 nmol/L)
Limit of Quantitation	= 0.50 mg/L (5.90 nmol/L)

The Limit of Blank and Limit of Detection were determined in accordance with the CLSI (Clinical and Laboratory Standards Institute) EP17-A2 requirements.

The Limit of Blank is the 95th percentile value from n ≥ 60 measurements of analyte-free samples over several independent series. The Limit of Blank corresponds to the concentration below which analyte-free samples are found with a probability of 95 %.

The Limit of Detection is determined based on the Limit of Blank and the standard deviation of low concentration samples.

The Limit of Detection corresponds to the lowest analyte concentration which can be detected (value above the Limit of Blank with a probability of 95 %).

The Limit of Quantitation is the lowest analyte concentration that can be reproducibly measured with a total error of 20 %. It has been determined using low concentration sTfR samples.

Expected values

The values shown below were performed on samples from an apparently healthy population, using the Tina-quant Soluble Transferrin Receptor II assay (STFR2). The calculation is based on 165 sera (101 men, 64 women). The age range was between 22 and 83 years. The analysis of the data with the 2.5 % and the 97.5 % percentile gave a soluble transferrin receptor (sTfR) range from 1.71 mg/L (20.2 nmol/L) to 4.13 mg/L (48.7 nmol/L).

Each laboratory should investigate the transferability of the expected values to its own patient population and if necessary determine its own reference ranges.

Specific performance data

Representative performance data on the analyzers are given below. Results obtained in individual laboratories may differ.

Precision

Precision was determined using human samples and controls in accordance with the CLSI (Clinical and Laboratory Standards Institute) EP5-A3 requirements with repeatability (n = 84) and intermediate precision (2 aliquots per run, 2 runs per day, 21 days)

The following results were obtained:

Repeatability	*Mean*	*SD*	*CV*
	mg/L	*mg/L*	*%*
Control Set sTfR II L 1	2.56	0.0389	1.5
Control Set sTfR II L 2	6.91	0.0626	0.9
Human serum 1	1.21	0.0375	3.1
Human serum 2	2.00	0.0438	2.2
Human serum 3	5.27	0.0526	1.0
Human serum 4	9.23	0.108	1.2
Human serum 5	17.7	0.157	0.9

Intermediate precision	*Mean*	*SD*	*CV*
	mg/L	*mg/L*	*%*
Control Set sTfR II L 1	2.56	0.0444	1.7
Control Set sTfR II L 2	6.91	0.0732	1.1
Human serum 1	1.21	0.0388	3.2
Human serum 2	2.00	0.0475	2.4
Human serum 3	5.27	0.0675	1.3
Human serum 4	9.31	0.118	1.3
Human serum 5	17.7	0.192	1.1

Method comparison

sTfR values for human serum and plasma samples obtained on a **cobas c** 501 analyzer (y) were compared with those determined using the Soluble Transferrin Receptor assay (STFR) on a **cobas c** 501 analyzer(x).

Sample size (n) = 87

Passing/Bablok[14]	Linear regression
y = 0.987x + 0.0347 mg/L	y = 0.989x + 0.0264 mg/L
τ = 0.939	r = 0.996

The sample concentrations were between 0.660 and 19.1 mg/L.

References

1 Wick M, Pinggera W, Lehmann P, eds. Iron Metabolism, Diagnosis and Therapy of Anemias. 4th ed. Vienna/New York: Springer Verlag 1999.

2 Ferguson BJ, Skikne BS, Simpson KM, et al. Serum transferrin receptor distinguishes the anemia of chronic diseases from iron deficiency anemia. J Lab Clin Med 1992;19:385-390.

3 Baynes RD, Skikne BS, Cook JD. Circulating transferrin receptors and assessment of iron status. J Nutr Biochem 1994;5:322-330.

4 Kuiper-Kramer EPA, Huisman CMS, van Raan J, et al. Analytical and clinical implications of soluble transferrin receptors. Eur J Clin Chem Clin Biochem 1996;34:645-649.

5 Baynes RD. Assessment of iron status. Clin Biochem 1996;29:209-215.

6 Punnonen K, Irjala K, Rajamäki A. Serum transferrin receptor and its ratio to serum ferritin in the diagnosis of iron deficiency. Blood 1997;89:1052-1057.

7 Suominen P, Punnonen K, Rajamäki A, et al. Serum transferrin receptor and transferrin receptor-ferritin index identify healthy subjects with subclinical iron deficits. Blood 1998;92:2934-2939.

8 Haeckel R, ed. Evaluation methods in laboratory medicine. VCH Verlagsgesellschaft, Weinheim 1993.

9 Cook JD, Skikne BS, Baynes RD. Serum transferrin receptor. Annu Rev Med 1993;44:63-74.

10 Glick MR, Ryder KW, Jackson SA. Graphical Comparisons of Interferences in Clinical Chemistry Instrumentation. Clin Chem 1986;32:470-475.

Woldeyohannes M, *et al. BMJ Open* 2023; 13:e067641. doi: 10.1136/bmjopen-2022-067641

0107227841190c501V1.0

STFR2

Tina-quant Soluble Transferrin Receptor II

cobas®

11 Breuer J. Report on the Symposium "Drug effects in Clinical Chemistry Methods". Eur J Clin Chem Clin Biochem 1996;34:385-386.

12 Sonntag O, Scholer A. Drug interference in clinical chemistry: recommendation of drugs and their concentrations to be used in drug interference studies. Ann Clin Biochem 2001;38:376-385.

13 Bakker AJ, Mücke M. Gammopathy interference in clinical chemistry assays: mechanisms, detection and prevention. Clin Chem Lab Med 2007;45(9):1240-1243.

14 Bablok W, Passing H, Bender R, et al. A general regression procedure for method transformation. Application of linear regression procedures for method comparison studies in clinical chemistry, Part III. J Clin Chem Clin Biochem 1988 Nov;26(11):783-790.

A point (period/stop) is always used in this Method Sheet as the decimal separator to mark the border between the integral and the fractional parts of a decimal numeral. Separators for thousands are not used.

Symbols

Roche Diagnostics uses the following symbols and signs in addition to those listed in the ISO 15223-1 standard (for USA: see dialog.roche.com for definition of symbols used):

CONTENT	Contents of kit
→	Volume for reconstitution
GTIN	Global Trade Item Number

COBAS, COBAS C and TINA-QUANT are trademarks of Roche.

All other product names and trademarks are the property of their respective owners.

Additions, deletions or changes are indicated by a change bar in the margin.

© 2020, Roche Diagnostics

CE

Roche Diagnostics GmbH, Sandhofer Strasse 116, D-68305 Mannheim
www.roche.com

Roche
