# Supplementary material for: Ethiopia National Food and Nutrition Survey to inform the Ethiopian National Food and Nutrition Strategy: a study protocol
Source: BMJ Open. 2023 Apr 25;13(4):e067641. doi: 10.1136/bmjopen-2022-067641 (PMC10151871; doi:10.1136/bmjopen-2022-067641)
Supplement: Supplementary data [file bmjopen-2022-067641supp008.pdf]

ms_03737551190V14.0

Ferritin

Ferritin

cobas®

REF	Σ	SYSTEM
**03737551** 190	100	Elecsys 2010<br>MODULAR ANALYTICS E170<br>**cobas e** 411<br>**cobas e** 601<br>**cobas e** 602

English

Intended use

Immunoassay for the in vitro quantitative determination of ferritin in human serum and plasma.

The **e**lectro**c**hemi**l**uminescence **i**mmuno**a**ssay "ECLIA" is intended for use on Elecsys and **cobas e** immunoassay analyzers.

Summary

Ferritin is a macromolecule with a molecular weight of at least 440 kDa (depending on the iron content) and consists of a protein shell (apoferritin) of 24 subunits and an iron core containing an average of approx. 2500 Fe^{3+} ions (in liver and spleen ferritin).[1]

Ferritin tends to form oligomers, and when it is present in excess in the cells of the storage organs there is a tendency for condensation to semicrystalline hemosiderin to occur in the lysosomes.

At least 20 isoferritins can be distinguished with the aid of isoelectric focusing.[2] This microheterogeneity is due to differences in the contents of the acidic H and weakly basic L subunits. The basic isoferritins are responsible for the long-term iron storage function, and are found mainly in the liver, spleen, and bone marrow.[1,3]

Acidic isoferritins are found mainly in the myocardium, placenta, and tumor tissue. They have a lower iron content and presumably function as intermediaries for the transfer of iron in various syntheses.[4,5,6]

The determination of ferritin is a suitable method for ascertaining the iron metabolism situation. Determination of ferritin at the beginning of therapy provides a representative measure of the body's iron reserves. A storage deficiency in the reticulo-endothelial system (RES) can be detected at a very early stage.[7]

Clinically, a threshold value of 20 µg/L (ng/mL) has proved useful in the detection of prelatent iron deficiency. This value provides a reliable indication of exhaustion of the iron reserves that can be mobilized for hemoglobin synthesis. Latent iron deficiency is defined as a fall below the 12 µg/L (ng/mL) ferritin threshold. These two values necessitate no further laboratory elucidation, even when the blood picture is still morphologically normal. If the depressed ferritin level is accompanied by hypochromic, microcytal anemia, then manifest iron deficiency is present.[1]

When the ferritin level is elevated and the possibility of a distribution disorder can be ruled out, this is a manifestation of iron overloading in the body. 400 µg/L (ng/mL) ferritin is used as the threshold value. Elevated ferritin values are also encountered with the following tumors: acute leukemia, Hodgkin's disease and carcinoma of the lung, colon, liver and prostate. The determination of ferritin has proved to be of value in liver metastasis. Studies indicate that 76 % of all patients with liver metastasis have ferritin values above 400 µg/L (ng/mL). Reasons for the elevated values could be cell necrosis, blocked erythropoiesis or increased synthesis in tumor tissue.

Two monoclonal mouse antibodies - M-4.184 and M-3.170 - are used to form the sandwich complex in the assay.

Test principle

Sandwich principle. Total duration of assay: 18 minutes.

- 1st incubation: 10 µL of sample, a biotinylated monoclonal ferritin-specific antibody, and a monoclonal ferritin-specific antibody labeled with a ruthenium complex[a)] form a sandwich complex.
- 2nd incubation: After addition of streptavidin-coated microparticles, the complex becomes bound to the solid phase via interaction of biotin and streptavidin.
- The reaction mixture is aspirated into the measuring cell where the microparticles are magnetically captured onto the surface of the electrode. Unbound substances are then removed with ProCell/ProCell M. Application of a voltage to the electrode then induces chemiluminescent emission which is measured by a photomultiplier.
- Results are determined via a calibration curve which is instrument-specifically generated by 2-point calibration and a master curve provided via the reagent barcode.

a) Tris(2,2'-bipyridyl)ruthenium(II)-complex ($Ru(bpy)_3^{2+}$)

Reagents - working solutions

The reagent rackpack is labeled as FERR.

M Streptavidin-coated microparticles (transparent cap), 1 bottle, 6.5 mL:
Streptavidin-coated microparticles 0.72 mg/mL; preservative.

R1 Anti-Ferritin-Ab~biotin (gray cap), 1 bottle, 10 mL:
Biotinylated monoclonal anti-ferritin antibody (mouse) 3.0 mg/L; phosphate buffer 100 mmol/L, pH 7.2; preservative.

R2 Anti-ferritin-Ab~$Ru(bpy)_3^{2+}$ (black cap), 1 bottle, 10 mL:
Monoclonal anti-ferritin antibody (mouse) labeled with ruthenium complex 6.0 mg/L; phosphate buffer 100 mmol/L, pH 7.2; preservative.

Precautions and warnings

For in vitro diagnostic use.
Exercise the normal precautions required for handling all laboratory reagents.
Disposal of all waste material should be in accordance with local guidelines.
Safety data sheet available for professional user on request.

Avoid foam formation in all reagents and sample types (specimens, calibrators and controls).

Reagent handling

The reagents in the kit have been assembled into a ready-for-use unit that cannot be separated.

All information required for correct operation is read in from the respective reagent barcodes.

Storage and stability

Store at 2-8 °C.

Do not freeze.

Store the Elecsys reagent kit **upright** in order to ensure complete availability of the microparticles during automatic mixing prior to use.

Stability:	
unopened at 2-8 °C	up to the stated expiration date
after opening at 2-8 °C	12 weeks
on the analyzers	6 weeks

Specimen collection and preparation

Only the specimens listed below were tested and found acceptable.

Serum collected using standard sampling tubes.

Li-, Na-heparin, K_3-EDTA and sodium citrate plasma.

When sodium citrate is used, the results must be corrected by + 10 %.

Criterion: Recovery within 90-110 % of serum value or slope 0.9-1.1 + intercept within < ± 2x analytical sensitivity (LDL) + coefficient of correlation > 0.95.

Stable for 7 days at 2-8 °C, 12 months at -20 °C.[8]

ms_03737551190V14.0

Ferritin

Ferritin

The sample types listed were tested with a selection of sample collection tubes that were commercially available at the time of testing, i.e. not all available tubes of all manufacturers were tested. Sample collection systems from various manufacturers may contain differing materials which could affect the test results in some cases. When processing samples in primary tubes (sample collection systems), follow the instructions of the tube manufacturer.

Centrifuge samples containing precipitates before performing the assay.

Do not use heat-inactivated samples.

Do not use samples and controls stabilized with azide.

Ensure the samples, calibrators and controls are at 20-25 °C prior to measurement.

Due to possible evaporation effects, samples, calibrators and controls on the analyzers should be analyzed/measured within 2 hours.

Materials provided

See "Reagents – working solutions" section for reagents.

Materials required (but not provided)

- REF 03737586190, Ferritin CalSet, 4 x 1 mL
- REF 11776452122, PreciControl Tumor Marker, for 2 x 3 mL each of PreciControl Tumor Marker 1 and 2 or
  REF 05618860190, PreciControl Varia, for 2 x 3 mL each of PreciControl Varia 1 and 2
- REF 11732277122, Diluent Universal, 2 x 16 mL sample diluent or
  REF 03183971122, Diluent Universal, 2 x 36 mL sample diluent
- General laboratory equipment
- Elecsys 2010, MODULAR ANALYTICS E170 or **cobas e** analyzer

Accessories for Elecsys 2010 and **cobas e** 411 analyzers:

- REF 11662988122, ProCell, 6 x 380 mL system buffer
- REF 11662970122, CleanCell, 6 x 380 mL measuring cell cleaning solution
- REF 11930346122, Elecsys SysWash, 1 x 500 mL washwater additive
- REF 11933159001, Adapter for SysClean
- REF 11706802001, Elecsys 2010 AssayCup, 60 x 60 reaction vessels
- REF 11706799001, Elecsys 2010 AssayTip, 30 x 120 pipette tips

Accessories for MODULAR ANALYTICS E170, **cobas e** 601 and **cobas e** 602 analyzers:

- REF 04880340190, ProCell M, 2 x 2 L system buffer
- REF 04880293190, CleanCell M, 2 x 2 L measuring cell cleaning solution
- REF 03023141001, PC/CC-Cups, 12 cups to prewarm ProCell M and CleanCell M before use
- REF 03005712190, ProbeWash M, 12 x 70 mL cleaning solution for run finalization and rinsing during reagent change
- REF 12102137001, AssayTip/AssayCup Combimagazine M, 48 magazines x 84 reaction vessels or pipette tips, waste bags
- REF 03023150001, WasteLiner, waste bags
- REF 03027651001, SysClean Adapter M

Accessories for all analyzers:

- REF 11298500316, Elecsys SysClean, 5 x 100 mL system cleaning solution

Assay

For optimum performance of the assay follow the directions given in this document for the analyzer concerned. Refer to the appropriate operator's manual for analyzer-specific assay instructions.

Resuspension of the microparticles takes place automatically prior to use. Read in the test-specific parameters via the reagent barcode. If in exceptional cases the barcode cannot be read, enter the 15-digit sequence of numbers.

Bring the cooled reagents to approx. 20 °C and place on the reagent disk (20 °C) of the analyzer. Avoid foam formation. The system automatically regulates the temperature of the reagents and the opening/closing of the bottles.

Calibration

Traceability: The Ferritin assay (REF 03737551) has been standardized against the Ferritin assay (REF 11820982). The Ferritin assay (REF 11820982) has been standardized against the Enzymun-Test Ferritin method. This in turn has been standardized against the 1st International Standard (IS) NIBSC (National Institute for Biological Standards and Control) "Reagent for Ferritin (human liver)" 80/602.

Recovery studies, including a published study,[9] to assess traceability of the Elecsys Ferritin assay to more recent international standards (2nd IS 80/578 and 3rd IS 94/572) have been conducted, with results showing very good agreement.

Every Elecsys reagent set has a barcoded label containing specific information for calibration of the particular reagent lot. The predefined master curve is adapted to the analyzer using the relevant CalSet.

Calibration frequency: Calibration must be performed once per reagent lot using fresh reagent (i.e. not more than 24 hours since the reagent kit was registered on the analyzer). Renewed calibration is recommended as follows:

- after 1 month (28 days) when using the same reagent lot
- after 7 days (when using the same reagent kit on the analyzer)
- as required: e.g. quality control findings outside the defined limits

Quality control

For quality control, use PreciControl Tumor Marker or PreciControl Varia.

In addition, other suitable control material can be used.

Controls for the various concentration ranges should be run individually at least once every 24 hours when the test is in use, once per reagent kit, and following each calibration.

The control intervals and limits should be adapted to each laboratory's individual requirements. Values obtained should fall within the defined limits. Each laboratory should establish corrective measures to be taken if values fall outside the defined limits.

Follow the applicable government regulations and local guidelines for quality control.

Calculation

The analyzer automatically calculates the analyte concentration of each sample (either in µg/L or ng/mL).

Limitations - interference

The assay is unaffected by icterus (bilirubin < 1112 µmol/L or < 65 mg/dL), hemolysis (Hb < 0.31 mmol/L or < 0.5 g/dL), lipemia (Intralipid < 3300 mg/dL) and biotin (< 205 nmol/L or < 50 ng/mL).

Criterion: Recovery within ± 10 % of initial value.

Samples should not be taken from patients receiving therapy with high biotin doses (i.e. > 5 mg/day) until at least 8 hours following the last biotin administration.

No interference was observed from rheumatoid factors up to a concentration of 2500 IU/mL.

There is no high-dose hook effect at ferritin concentrations up to 100000 µg/L (ng/mL).

In vitro tests were performed on 19 commonly used pharmaceuticals. No interference with the assay was found.

Iron^{2+}- and iron^{3+}-ions at therapeutic concentrations do not interfere with the Elecsys Ferritin assay.

In rare cases, interference due to extremely high titers of antibodies to analyte-specific antibodies, streptavidin or ruthenium can occur. These effects are minimized by suitable test design.

For diagnostic purposes, the results should always be assessed in conjunction with the patient's medical history, clinical examination and other findings.

Limits and ranges

Measuring range

0.500-2000 µg/L (ng/mL) (defined by the lower detection limit and the maximum of the master curve). Values below the lower detection limit are reported as < 0.500 µg/L (ng/mL). Values above the measuring range are reported as > 2000 µg/L (ng/mL) (or up to 100000 µg/L (ng/mL) for 50-fold diluted samples).

Woldeyohannes M, *et al. BMJ Open* 2023; 13:e067641. doi: 10.1136/bmjopen-2022-067641

ms_03737551190V14.0

Ferritin

cobas®

Ferritin

Lower limits of measurement

Lower detection limit of the test

Lower detection limit: 0.50 µg/L (ng/mL)

The lower detection limit represents the lowest measurable analyte level that can be distinguished from zero. It is calculated as the value lying two standard deviations above that of the lowest standard (master calibrator, standard 1 + 2 SD, repeatability study, n = 21).

Dilution

Samples with ferritin concentrations above the measuring range can be diluted with Diluent Universal. The recommended dilution is 1:50 (either automatically by the MODULAR ANALYTICS E170, Elecsys 2010 or **cobas e** analyzers or manually). The concentration of the diluted sample must be > 40 µg/L (ng/mL).

After manual dilution, multiply the result by the dilution factor.

After dilution by the analyzers, the MODULAR ANALYTICS E170, Elecsys 2010 and **cobas e** software automatically takes the dilution into account when calculating the sample concentration.

Expected values

Results of a study with the Enzymun-Test Ferritin method on samples from 224 healthy test subjects (104 women - mainly premenopausal - and 120 men) are given below. The values correspond to the 5th and 95th percentiles.[10]

Men, 20-60 years: 30-400 µg/L (ng/mL)

Women, 17-60 years: 13-150 µg/L (ng/mL)

Each laboratory should investigate the transferability of the expected values to its own patient population and if necessary determine its own reference ranges.

Specific performance data

Representative performance data on the analyzers are given below. Results obtained in individual laboratories may differ.

Precision

Precision was determined using Elecsys reagents, pooled human sera and controls in a protocol (EP5-A2) of the CLSI (Clinical and Laboratory Standards Institute): 2 runs per day in duplication each for 21 days (n = 84). The following results were obtained:

Elecsys 2010 and **cobas e** 411 analyzers					
		**Repeatability**		**Intermediate precision**	
Sample	Mean µg/L (ng/mL)	SD µg/L (ng/mL)	CV %	SD µg/L (ng/mL)	CV %
Human serum 1	1.45	0.101	7.0	0.168	11.6
Human serum 2	11.9	0.411	3.5	0.798	6.7
Human serum 3	19.2	0.780	4.1	1.47	7.7
Human serum 4	376	10.8	2.9	17.2	4.6
Human serum 5	1361	26.5	1.9	84.4	6.2
PreciControl Varia 1	134	1.96	1.5	2.75	2.1
PreciControl Varia 2	858	15.1	1.8	21.7	2.5

MODULAR ANALYTICS E170, **cobas e** 601 and **cobas e** 602 analyzers					
		**Repeatability**		**Intermediate precision**	
Sample	Mean µg/L (ng/mL)	SD µg/L (ng/mL)	CV %	SD µg/L (ng/mL)	CV %
Human serum 1	1.12	0.139	12.4	0.263	23.4
Human serum 2	12.3	0.467	3.8	0.789	6.4
Human serum 3	20.5	0.837	4.1	1.67	8.1
Human serum 4	392	8.14	2.1	16.9	4.3
Human serum 5	1449	35.6	2.5	92.8	6.4
PreciControl Varia 1	140	2.31	1.7	3.53	2.5
PreciControl Varia 2	900	14.4	1.6	25.0	2.8

Method comparison

A comparison of the Ferritin assay, REF 03737551 (y) with the Ferritin assay, REF 11820982 (x) using clinical samples gave the following correlations:

Number of samples measured: 134

Passing/Bablok[11]	Linear regression
y = 1.00x + 0.72	y = 0.99x + 4.11
τ = 0.984	r = 0.999

The sample concentrations were between approximately 2.68 and 1891 µg/L (ng/mL).

Analytical specificity

Human liver ferritin: 100 % recovery

Human spleen ferritin: 85 % recovery

Human heart ferritin: 1 % recovery

References

1 Wick M, Pinggera W, Lehmann P. Ferritin in Iron Metabolism - Diagnosis of Anemieas (second edition). Springer-Verlag 1995;ISBN 3-211-82525-8 and ISBN 0-387-82525-8.

2 Arosio P, Levi S, Gabri E, et al. Heterogeneity of ferritin II: Immunological aspects. In: Albertini A, Arosio P, Chiancone E, Drysdale J (eds). Ferritins and isoferritins as biochemical markers. Elsevier, Amsterdam 1984;33-47.

3 Kaltwasser JP, Werner E. Serumferritin: Methodische und Klinische Aspekte. Springer Verlag (1980).

4 Morikawa K, Oseko F, Morikawa S. A role for ferritin in hematopoiesis and the immune system. Leuk-Lymphoma 1995;18(5-6):429-433.

5 Borch-Iohnson B. Determination of Iron status: brief review of physiological effects on iron measures. Analyst 1995;120(3):891-903.

6 Cook JD, Skikne BS, Baynes RD. Iron deficiency: the global perspective. Adv-Exp-Med-Biol 1994;356:219-228.

7 Albertini A, Arosio P, Chiancone E, et al. (eds). Functional aspects of isoferritins. In: Jacobs A, Hodgetts J, Hoy TG. Ferritins and isoferritins as biochemical markers. Elsevier, Amsterdam 1984;113-127.

8 Guder WG, Narayanan S, Wisser H, et al. List of Analytes; Preanalytical Variables. Brochure in: Samples: From the Patient to the Laboratory. GIT-Verlag, Darmstadt 1996:14. ISBN 3-928865-22-6.

9 Blackmore S, Hamilton M, Lee A, et al. Automated immunoassay methods for ferritin: recovery studies to assess traceability to an international standard. Clin Chem Lab Med 2008;46(10):1450-1457.

10 Lotz J, Hafner G, Prellwitz W. Reference Study for Ferritin Assays. Kurzmitteilung Clin Lab 1997;43(11):993-994.

11 Bablok W, Passing H, Bender R, et al. A general regression procedure for method transformation. Application of linear regression procedures for method comparison studies in clinical chemistry, Part III. J Clin Chem Clin Biochem 1988 Nov;26(11):783-790.

For further information, please refer to the appropriate operator's manual for the analyzer concerned, the respective application sheets, the product information and the Method Sheets of all necessary components (if available in your country).

A point (period/stop) is always used in this Method Sheet as the decimal separator to mark the border between the integral and the fractional parts of a decimal numeral. Separators for thousands are not used.

Woldeyohannes M, *et al. BMJ Open* 2023; 13:e067641. doi: 10.1136/bmjopen-2022-067641

ms_03737551190V14.0

Ferritin

Ferritin

cobas®

Symbols

Roche Diagnostics uses the following symbols and signs in addition to those listed in the ISO 15223-1 standard.

CONTENT	Contents of kit
SYSTEM	Analyzers/Instruments on which reagents can be used
REAGENT	Reagent
CALIBRATOR	Calibrator
→	Volume after reconstitution or mixing

COBAS, COBAS E, ELECSYS, MODULAR and PRECICONTROL are trademarks of Roche. INTRALIPID is a trademark of Fresenius Kabi AB.

All other product names and trademarks are the property of their respective owners.

Significant additions or changes are indicated by a change bar in the margin.

© 2013, Roche Diagnostics

CE

Roche Diagnostics GmbH, Sandhofer Strasse 116, D-68305 Mannheim
www.roche.com

Roche
