# Supplementary material for: Ethiopia National Food and Nutrition Survey to inform the Ethiopian National Food and Nutrition Strategy: a study protocol
Source: BMJ Open. 2023 Apr 25;13(4):e067641. doi: 10.1136/bmjopen-2022-067641 (PMC10151871; doi:10.1136/bmjopen-2022-067641)
Supplement: Supplementary data [file bmjopen-2022-067641supp009.pdf]

05944295500V8.0

Elecsys Folate RBC

cobas®

REF	(i)	Σ	SYSTEM
05944295190	05944295500	100	**cobas e** 411<br>**cobas e** 601<br>**cobas e** 602

English

System information

For **cobas e** 411 analyzer: test number 1210
For **cobas e** 601 and **cobas e** 602 analyzers: Application Code Number 272

Intended use

This assay is used for the in vitro quantitative determination of folate in erythrocytes (red blood cells, RBC). The electrochemiluminescence binding assay is intended for use on the Elecsys and **cobas e** immunoassay analyzers.

Summary

Folate belongs to the family of B-group vitamins composed of an aromatic pteridine ring linked through a methylene group to p-aminobenzoic acid and a glutamate residue. Folate (folic acid) is vital for normal cellular functions and plays an essential role in nucleic acid synthesis, methionine regeneration, shuttling and redox reactions of one-carbon units required for normal metabolism and regulation.[1,2]

The folate metabolism can be exemplified as a cycle, where folate facilitates the transfer of one-carbon units from one molecule to another required in various biochemical reactions: for example, tetrahydrofolate (THF) accepts a single carbon unit from serine, which is reduced in a number of steps to 5-methyltetrahydrofolate (5-MTHF). 5-MTHF gives its methyl group to homocysteine, which is - with involvement of methionine synthase and vitamin B12 - enzymatically converted to methionine. The resulting THF starts again the cycle of methyl group synthesis. From methionine, the methyl groups are transferred to S-adenosylmethionine (SAM).[3] SAM serves as a methyl group donor in several methylation reactions, like DNA, RNA and protein methylation.[1]

The methionine cycle is highly sensitive to folate deficiency: with a low folate status, the ability of the cell to re-methylate homocysteine is impaired and this results in increased homocysteine concentrations in plasma.[2]

Folate also plays an essential role in the synthesis of purine and pyrimidine precursors of nucleic acids. Altered distribution of methyl groups and impaired DNA synthesis play an essential role in the development of cancers. Abnormal folate status has also been linked with the development of diseases like cardiovascular diseases, neural tube defects, cleft lip and palate, late pregnancy complications, neurodegenerative and psychiatric disorders.[1,2]

Folate belongs to the group of essential vitamins, i.e. it cannot be synthesized by the human organism and therefore must be absorbed from diet. Primary sources of folates are green and leafy vegetables, sprouts, fruits, brewer's yeast and liver.[1,2]

Folate deficiency can be caused by decreased nutritional intake, poor absorption of ingested folate in the intestine or increased demand of folate, for example during physical activity or pregnancy. Deficiency of folate can also be a result of liver diseases or impaired folate metabolism due to genetic defects or drug interactions.[2]

A clinical manifestation of both folate and vitamin B12 deficiency is the so called megaloblastic (macrocytic) anemia: due to the affected DNA synthesis and cell maturation, especially involving the cells of erythropoiesis, the total count of erythrocytes is significantly reduced. The hemoglobin synthesis capacity however is normal, which leads to abnormally large erythrocyte precursors ("macrocytes" or "megaloblasts"), which have an elevated hemoglobin content ("hyperchromic anemia").[3,4]

Serum folate concentrations may be affected by recent folate intakes, whereas red blood cell (RBC) folate is a measure of the folate intake across the 90-120 days lifespan of erythrocytes. Thus, folate concentrations in RBC give a more accurate picture of a patient's underlying folate status than serum folate concentrations, and are considered by experts as the better measure for folate status.[5]

Because vitamin B12 and folate are closely interrelated in the cellular one-carbon unit metabolism, and also hematologic and clinical consequences of the two vitamin deficiency states might be similar, it is advisable to determine both parameters simultaneously in patients with the relevant symptoms of vitamin deficiency.[3,4]

Test principle

Competition principle. Total duration of assay: 27 minutes.

Whole blood treated with anticoagulants (heparin or EDTA) is mixed with ascorbic acid solution and incubated for approximately 90 minutes at 20-25 °C. Lysis of the erythrocytes takes place, with liberation and stabilization of the intracellular folate. The resulting hemolysate sample is then used for subsequent measurement.

- 1st incubation: By incubating 25 µL of hemolysate sample with the folate pretreatment reagents 1 and 2, bound folate is released from endogenous folate binding proteins.
- 2nd incubation: By incubating the pretreated sample with the ruthenium labeled folate binding protein, a folate complex is formed, the amount of which is dependent upon the analyte concentration in the sample.
- 3rd incubation: After addition of streptavidin-coated microparticles and folate labeled with biotin, the unbound sites of the ruthenium labeled folate binding protein become occupied, with formation of a ruthenium labeled folate binding protein-folate biotin complex. The entire complex becomes bound to the solid phase via interaction of biotin and streptavidin.
- The reaction mixture is aspirated into the measuring cell where the microparticles are magnetically captured onto the surface of the electrode. Unbound substances are then removed with ProCell/ProCell M. Application of a voltage to the electrode then induces chemiluminescent emission which is measured by a photomultiplier.
- Results are determined via a calibration curve which is instrument-specifically generated by 2-point calibration and a master curve provided via the reagent barcode or e-barcode.

Reagents - working solutions

The reagent rackpack (M, R1, R2) is labeled as RBC-FOL.

PT1 Pretreatment reagent 1 (white cap), 1 bottle, 4 mL:
Sodium 2-mercaptoethanesulfonate (MESNA) 40 g/L, pH 5.5.

PT2 Pretreatment reagent 2 (gray cap), 1 bottle, 5 mL:
Sodium hydroxide 25 g/L.

M Streptavidin-coated microparticles (transparent cap), 1 bottle, 6.5 mL:
Streptavidin-coated microparticles 0.72 mg/mL; preservative.

R1 Folate binding protein~Ru(bpy)$_3^{2+}$ (gray cap), 1 bottle, 9 mL:
Ruthenium labeled folate binding protein 75 µg/L; human serum albumin (stabilizer); borate/phosphate/citrate buffer 70 mmol/L, pH 5.5; preservative.

R2 Folate~biotin (black cap), 1 bottle, 8 mL:
Biotinylated folate 17 µg/L; biotin 120 µg/L; human serum albumin (stabilizer); borate buffer 100 mmol/L, pH 9.0; preservative.

Precautions and warnings

For in vitro diagnostic use.
Exercise the normal precautions required for handling all laboratory reagents.
Disposal of all waste material should be in accordance with local guidelines.
Safety data sheet available for professional user on request.

This kit contains components classified as follows in accordance with the Regulation (EC) No. 1272/2008:

2-methyl-2H-isothiazol-3-one hydrochloride

EUH 208 May produce an allergic reaction.

Woldeyohannes M, *et al. BMJ Open* 2023; 13:e067641. doi: 10.1136/bmjopen-2022-067641

05944295500V8.0

Elecsys Folate RBC

cobas®

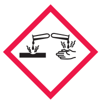

Danger

H290	May be corrosive to metals.
H314	Causes severe skin burns and eye damage.

Prevention:

P280	Wear protective gloves/ protective clothing/ eye protection/ face protection.

Response:

P301 + P330 + P331	IF SWALLOWED: Rinse mouth. Do NOT induce vomiting.
P303 + P361 + P353	IF ON SKIN (or hair): Take off immediately all contaminated clothing. Rinse skin with water.
P304 + P340 + P310	IF INHALED: Remove person to fresh air and keep comfortable for breathing. Immediately call a POISON CENTER/ doctor.
P305 + P351 + P338 + P310	IF IN EYES: Rinse cautiously with water for several minutes. Remove contact lenses, if present and easy to do. Continue rinsing. Immediately call a POISON CENTER/ doctor.
P390	Absorb spillage to prevent material damage.

Product safety labeling follows EU GHS guidance.

Contact phone: all countries: +49-621-7590

All human material should be considered potentially infectious. All products derived from human blood are prepared exclusively from the blood of donors tested individually and shown to be free from HBsAg and antibodies to HCV and HIV. The testing methods used assays approved by the FDA or cleared in compliance with the European Directive 98/79/EC, Annex II, List A.

However, as no testing method can rule out the potential risk of infection with absolute certainty, the material should be handled with the same level of care as a patient specimen. In the event of exposure, the directives of the responsible health authorities should be followed.[6,7]

Avoid foam formation in all reagents and sample types (specimens, calibrators and controls).

Reagent handling

The reagents in the kit have been assembled into a ready-for-use unit that cannot be separated.

All information required for correct operation is read in from the respective reagent barcodes.

Storage and stability

Store at 2-8 °C.

Do not freeze.

Store the Elecsys reagent kit **upright** in order to ensure complete availability of the microparticles during automatic mixing prior to use.

Stability:	
unopened at 2-8 °C	up to the stated expiration date
after opening at 2-8 °C	8 weeks
on the analyzers	2 weeks

Specimen collection and preparation

Only the specimens listed below were tested and found acceptable.

Hemolysate prepared from whole blood treated with anticoagulants (Na-heparin or K_3-EDTA).

- *For the determination of folate in RBC:* Determine hematocrit in whole blood samples and record the value.
- *Preparation of the hemolysate sample*

  Mix 3.0 mL of Folate RBC Hemolyzing Reagent (ascorbic acid solution, 0.2 %) and 100 µL of well-mixed whole blood, avoiding foam formation. Incubate with closed caps for 90 ± 15 minutes at 20-25 °C.

Stability:

Whole blood: 2 hours at 20-25 °C[8], 24 hours at 2-8 °C, 1 month at -20 °C (± 5 °C) (only EDTA-blood).

Hemolysate sample: 1 month at -20 °C (± 5 °C), freeze only once.

The sample types listed were tested with a selection of sample collection tubes that were commercially available at the time of testing, i.e. not all available tubes of all manufacturers were tested. Sample collection systems from various manufacturers may contain differing materials which could affect the test results in some cases. When processing samples in primary tubes (sample collection systems), follow the instructions of the tube manufacturer.

Samples should not subsequently be altered with additives (biocides, anti-oxidants or substances possibly changing the pH of the sample) in order to avoid erroneous folate recovery.

Ensure the samples, calibrators and controls are at 20-25 °C prior to measurement.

Due to possible evaporation effects, samples, calibrators and controls on the analyzers should be analyzed/measured within 2 hours.

If measurements cannot be carried out within 2 hours please store the hemolysate sample at -20 °C (± 5 °C).

Materials provided

See "Reagents – working solutions" section for reagents.

Materials required (but not provided)

- REF 05944309190, Folate RBC CalSet, for 4 x 1.0 mL
- REF 05944317190, Folate RBC Hemolyzing Reagent kit for 4 x 200 mL, contains ascorbic acid
- General laboratory equipment
- **cobas e** analyzer

Additional materials for the **cobas e** 411 analyzer:

- REF 11662988122, ProCell, 6 x 380 mL system buffer
- REF 11662970122, CleanCell, 6 x 380 mL measuring cell cleaning solution
- REF 11930346122, Elecsys SysWash, 1 x 500 mL washwater additive
- REF 11933159001, Adapter for SysClean
- REF 11706802001, AssayCup, 60 x 60 reaction cups
- REF 11706799001, AssayTip, 30 x 120 pipette tips
- REF 11800507001, Clean-Liner

Additional materials for **cobas e** 601 and **cobas e** 602 analyzers:

- REF 04880340190, ProCell M, 2 x 2 L system buffer
- REF 04880293190, CleanCell M, 2 x 2 L measuring cell cleaning solution
- REF 03023141001, PC/CC-Cups, 12 cups to prewarm ProCell M and CleanCell M before use
- REF 03005712190, ProbeWash M, 12 x 70 mL cleaning solution for run finalization and rinsing during reagent change
- REF 03004899190, PreClean M, 5 x 600 mL detection cleaning solution
- REF 12102137001, AssayTip/AssayCup, 48 magazines x 84 reaction cups or pipette tips, waste bags
- REF 03023150001, WasteLiner, waste bags
- REF 03027651001, SysClean Adapter M

Additional materials for all analyzers:

- REF 11298500316, ISE Cleaning Solution/Elecsys SysClean, 5 x 100 mL system cleaning solution

05944295500V8.0

Elecsys Folate RBC

cobas®

Assay

The well-mixed hemolysate sample is placed in the sample zone of the analyzer and recorded by entering the sample identification data. Complete determinations on the analyzer within 2 hours after finalizing the preparation of the hemolysate sample.

For optimum performance of the assay follow the directions given in this document for the analyzer concerned. Refer to the appropriate operator's manual for analyzer-specific assay instructions.

Resuspension of the microparticles takes place automatically prior to use. Read in the test-specific parameters via the reagent barcode. If in exceptional cases the barcode cannot be read, enter the 15-digit sequence of numbers.

cobas e 601 and **cobas e** 602 analyzers: PreClean M solution is necessary.

Bring the cooled reagents to approximately 20 °C and place on the reagent disk (20 °C) of the analyzer. Avoid foam formation. The system automatically regulates the temperature of the reagents and the opening/closing of the bottles.

Calibration

Traceability: This method has been standardized against the Elecsys Folate III assay (REF 04476433190)/RBC application.

The standardization of the Elecsys Folate RBC assay includes the volume correction to account for the preparation of hemolysate sample (1:31 vol/vol).

Every Elecsys reagent set has a barcoded label containing specific information for calibration of the particular reagent lot. The predefined master curve is adapted to the analyzer using the relevant CalSet.

Calibration frequency: Calibration must be performed once per reagent lot using fresh reagent (i.e. not more than 24 hours since the reagent kit was registered on the analyzer).

Calibration interval may be extended based on acceptable verification of calibration by the laboratory.

Renewed calibration is recommended as follows:

- after 1 month (28 days) when using the same reagent lot
- after 7 days (when using the same reagent kit on the analyzer)
- as required: e.g. quality control findings outside the defined limits

Quality control

For quality control, use commercially available whole blood control material.

Controls for the various concentration ranges should be run individually at least once every 24 hours when the test is in use, once per reagent kit, and following each calibration.

The control intervals and limits should be adapted to each laboratory's individual requirements. Values obtained should fall within the defined limits. Each laboratory should establish corrective measures to be taken if values fall outside the defined limits.

If necessary, repeat the measurement of the samples concerned.

Follow the applicable government regulations and local guidelines for quality control.

Calculation

1. Whole blood folate (from hemolysate sample)

The standardization of the Elecsys Folate RBC assay includes the volume correction to account for the preparation of hemolysate sample (1:31 vol/vol).

The analyzer automatically calculates the analyte concentration of each sample in nmol/L or ng/mL.

Conversion factors: nmol/L x 0.44 = ng/mL
ng/mL x 2.27 = nmol/L

2. RBC folate

To calculate the folate concentration in the erythrocyte fraction of the sample **(RBC folate)**, the predetermined sample specific hematocrit value must be taken into account using the following equation:

$$\text{RBC folate} = \frac{\text{analyzer result}}{\text{\% hematocrit}} \times 100$$

Limitations - interference

The assay is unaffected by icterus (bilirubin < 564 µmol/L or < 33 mg/dL), lipemia (Intralipid < 1500 mg/dL), biotin (< 86.1 nmol/L or < 21 ng/mL), IgG < 16 g/L and IgA < 4.0 g/L.

Criterion: Recovery within ± 10 % of initial value with samples > 155 ng/mL and ≤ ± 15.5 ng/mL with samples ≤ 155 ng/mL.

Samples should not be taken from patients receiving therapy with high biotin doses (i.e. > 5 mg/day) until at least 8 hours following the last biotin administration.

No interference was observed from rheumatoid factors up to a concentration of 1000 IU/mL.

In vitro tests were performed on 16 commonly used pharmaceuticals and in addition on human erythropoietin. No interference with the assay was found.

It is contraindicated to measure samples of patients receiving therapy with certain pharmaceuticals, e.g. methotrexate or leucovorin, because of the cross-reactivity of folate binding protein with these compounds.

In rare cases, interference due to extremely high titers of antibodies to analyte-specific antibodies, streptavidin or ruthenium can occur. These effects are minimized by suitable test design.

For diagnostic purposes, the results should always be assessed in conjunction with the patient's medical history, clinical examination and other findings.

In rare cases, samples with low erythrocyte folate concentration, but high serum folate concentration can occur. In these cases, a correction of the folate concentration in erythrocytes by the serum folate concentration with the following equation is recommended*:

* expected values can be used as an indicator for high serum folate concentration

Corrected RBC folate concentration =

$$\text{RBC folate concentration} - \left(\text{serum folate concentration} \times \frac{100 - \text{\% hematocrit}}{\text{\% hematocrit}}\right)$$

<u>Example</u>

RBC folate concentration: 241 (ng/mL RBC);

serum folate concentration: 10.5 (ng/mL S);

hematocrit measured (%) = 45

Corrected RBC folate concentration =

$$241 \text{ ng/mL RBC} - \left(10.5 \text{ ng/mL S} \times \frac{100 - 45}{45}\right) = 228 \text{ ng/mL RBC}$$

Limits and ranges

Measuring range

120-620 ng/mL or 272-1407 nmol/L (defined by the Limit of Quantitation and the maximum of the master curve). Values below the Limit of Quantitation are reported as < 120 ng/mL (< 272 nmol/L). Values above the measuring range are reported as > 620 ng/mL (> 1407 nmol/L). Values are not corrected for the sample hematocrit.

Lower limits of measurement

Limit of Blank, Limit of Detection and Limit of Quantitation:

Limit of Blank = 20.0 ng/mL

Limit of Detection = 46.5 ng/mL

Limit of Quantitation = 120 ng/mL

The Limit of Blank, Limit of Detection and Limit of Quantitation were determined in accordance with the CLSI (Clinical and Laboratory Standards Institute) EP17-A requirements.

The Limit of Blank is the 95th percentile value from n ≥ 60 measurements of analyte-free samples over several independent series. The Limit of Blank corresponds to the concentration below which analyte-free samples are found with a probability of 95 %.

The Limit of Detection is determined based on the Limit of Blank and the standard deviation of low concentration samples. The Limit of Detection corresponds to the lowest analyte concentration which can be detected (value above the Limit of Blank with a probability of 95 %).

The Limit of Quantitation is defined as the lowest amount of analyte in a sample that can be accurately quantitated with a total allowable relative

Woldeyohannes M, *et al. BMJ Open* 2023; 13:e067641. doi: 10.1136/bmjopen-2022-067641

error of ≤ 30 %. It has been determined using low concentration folate samples.

Dilution

Hemolysate samples with folate concentrations above the measuring range can be diluted manually with Elecsys Folate RBC Hemolyzing Reagent (ascorbic acid solution, 0.2 %). The recommended dilution is 1:2. The concentration of the diluted sample must be > 265 ng/mL or > 602 nmol/L. After manual dilution, multiply the results by the dilution factor 2.

Expected values

The values shown below were measured on samples from an apparently healthy population, using the Elecsys Folate III/RBC application. The values can be applied for the Elecsys Folate RBC assay on all Elecsys and **cobas e** analyzers. The calculation is based on 290 sera (96 men, 194 women) from an European population. The age range was between 18 and 65 years. Pregnant or lactating women were excluded. The reference population was selected according to normal homocysteine values. The following values were obtained:

Whole blood folate (from hemolysate samples)					
	N	Median		2.5th-97.5th percentile	
		nmol/L	ng/mL	nmol/L	ng/mL
Europe	290	673	296	481-1212	212-534

The measured hematocrit value in this study showed a range from 37.1-46.1 %.

RBC folate (folate in erythrocyte fraction)					
	N	Median		2.5th-97.5th percentile	
		nmol/L	ng/mL	nmol/L	ng/mL
Europe	290	1657	730	1187-2854	523-1257

If pathologically low hematocrit values are considered for calculation of RBC folate in the erythrocyte fraction, elevated RBC folate concentrations may be observed. No medical conclusion should be based on the calculation considering hematocrit values in such cases. Instead, whole blood folate results (from hemolysate samples) and suitable expected values may be used.

Each laboratory should investigate the transferability of the expected values to its own patient population and if necessary determine its own reference ranges.

Specific performance data

Representative performance data on the analyzers are given below. Results obtained in individual laboratories may differ.

Precision

Precision was determined using Elecsys reagents and hemolysate samples in a protocol (EP5-A2) of the CLSI (Clinical and Laboratory Standards Institute): 2 runs per day in duplicate each for 21 days (n = 84). Results are given as whole blood folate (from hemolysate sample). The following results were obtained:

**cobas e** 411 analyzer								
			**Repeatability**			**Intermediate precision**		
Sample	Mean		SD		CV	SD		CV
	nmol/L	ng/mL	nmol/L	ng/mL	%	nmol/L	ng/mL	%
HL[a)] 1	154	68.0	11.7	5.17	7.6	21.9	9.65	14.2
HL 2	352	155	17.5	7.73	5.0	27.7	12.2	7.9
HL 3	618	272	25.4	11.2	4.1	38.4	16.9	6.2
HL 4	1195	527	38.8	17.1	3.3	56.3	24.8	4.7

a) HL = Hemolysate

**cobas e** 601 and **cobas e** 602 analyzers								
			**Repeatability**			**Intermediate precision**		
Sample	Mean		SD		CV	SD		CV
	nmol/L	ng/mL	nmol/L	ng/mL	%	nmol/L	ng/mL	%
HL 1	138	61.0	12.1	5.31	8.8	14.3	6.32	10.4
HL 2	434	191	26.1	11.5	6.0	28.4	12.5	6.5
HL 3	586	258	32.0	14.1	5.5	34.3	15.1	5.9
HL 4	1317	580	29.1	12.8	2.2	44.7	19.7	3.4

Method comparison

a) A comparison of the Elecsys Folate RBC assay (calibrated with Folate RBC CalSet; y) and the Elecsys Folate III/RBC application (calibrated with Folate III CalSet; x) using hemolyzed clinical samples gave the following correlations (ng/mL). Results are given as whole blood folate (from hemolysate sample).

Number of samples measured: 187

Passing/Bablok[9]	Linear regression
y = 1.02x - 14.1	y = 1.00x - 12.0
τ = 0.869	r = 0.985

The sample concentrations were between 151 and 551 ng/mL (343 and 1251 nmol/L).

b) A comparison of the Elecsys Folate RBC assay on the MODULAR ANALYTICS E170 analyzer (y) with the Elecsys Folate RBC assay on the Elecsys 2010 analyzer (x) (both tests have been calibrated with Folate RBC CalSet) using hemolyzed clinical samples gave the following correlations (ng/mL). Results are given as whole blood folate (from hemolysate sample).

Number of samples measured: 187

Passing/Bablok	Linear regression
y = 1.04x + 1.94	y = 1.02x + 8.07
τ = 0.814	r = 0.970

The sample concentrations were between 137 and 557 ng/mL (311 and 1264 nmol/L).

References

1 Nazki FH, Sameer AS, Ganaie BA. Folate: Metabolism, genes, polymorphisms and the associated diseases. Gene 2014;533(1):11-20.

2 Scaglione F, Panzavolta G. Folate, folic acid and 5-methyltetrahydrofolate are not the same thing. Xenobiotica 2014;44(5):480-488.

3 Reynolds EH. The neurology of folic acid deficiency. Handb Clin Neurol 2014;120:927-43.

4 Wick M, Pinggera W, Lehmann P. Clinical Aspects and Laboratory. Iron metabolism, Anemias. Springer Verlag, Wien, New York, 6th edition 2011:41-42.

5 Yetley EA, Pfeiffer CM, Phinney KW, et al. Biomarkers of folate status in NHANES: a roundtable summary. Am J Clin Nutr 2011;94(1):303S-312S.

6 Occupational Safety and Health Standards: Bloodborne pathogens. (29 CFR Part 1910.1030). Fed. Register.

7 Directive 2000/54/EC of the European Parliament and Council of 18 September 2000 on the protection of workers from risks related to exposure to biological agents at work.

8 Eijsden M, van der Wal MF, Hornstra G, et al. Can whole blood samples be stored over 24 hours without compromising stability of C-Reactive Protein, Retinol, Ferritin, Folic Acid and Fatty Acids in Epidemiology Research? Clin Chem 2005;51(1):230-232.

9 Bablok W, Passing H, Bender R, et al. A general regression procedure for method transformation. Application of linear regression procedures for method comparison studies in clinical chemistry, Part III. J Clin Chem Clin Biochem 1988 Nov;26(11):783-790.

05944295500V8.0

Elecsys Folate RBC

cobas®

For further information, please refer to the appropriate operator's manual for the analyzer concerned, the respective application sheets, the product information and the Method Sheets of all necessary components (if available in your country).

A point (period/stop) is always used in this Method Sheet as the decimal separator to mark the border between the integral and the fractional parts of a decimal numeral. Separators for thousands are not used.

Symbols

Roche Diagnostics uses the following symbols and signs in addition to those listed in the ISO 15223-1 standard (for USA: see dialog.roche.com for definition of symbols used):

CONTENT	Contents of kit
SYSTEM	Analyzers/Instruments on which reagents can be used
REAGENT	Reagent
CALIBRATOR	Calibrator
→	Volume after reconstitution or mixing
GTIN	Global Trade Item Number

COBAS, COBAS E, ELECSYS and PRECICONTROL are trademarks of Roche. INTRALIPID is a trademark of Fresenius Kabi AB.

All other product names and trademarks are the property of their respective owners.

Additions, deletions or changes are indicated by a change bar in the margin.

© 2020, Roche Diagnostics

CE

Roche Diagnostics GmbH, Sandhofer Strasse 116, D-68305 Mannheim
www.roche.com

Roche

Woldeyohannes M, *et al. BMJ Open* 2023; 13:e067641. doi: 10.1136/bmjopen-2022-067641
