# Supplementary material for: Ethiopia National Food and Nutrition Survey to inform the Ethiopian National Food and Nutrition Strategy: a study protocol
Source: BMJ Open. 2023 Apr 25;13(4):e067641. doi: 10.1136/bmjopen-2022-067641 (PMC10151871; doi:10.1136/bmjopen-2022-067641)
Supplement: Supplementary data [file bmjopen-2022-067641supp010.pdf]

0004628918190c501V12.0

CRPHS

cobas®

Cardiac C-Reactive Protein (Latex) High Sensitive

Order information

REF	CONTENT		Analyzer(s) on which **cobas c** pack(s) can be used
**04628918** 190	Cardiac C-Reactive Protein (Latex) High Sensitive (300 tests)	System-ID 07 6866 9	Roche/Hitachi **cobas c** 311, **cobas c** 501/502
**11355279** 216	Calibrator f.a.s. Proteins (5 x 1 mL)	Code 656	
**11355279** 160	Calibrator f.a.s. Proteins (5 x 1 mL, for USA)	Code 656	
**20766321** 322	CRP T Control N (5 x 0.5 mL)	Code 235	
**10557897** 122	Precinorm Protein (3 x 1 mL)	Code 302	
**10557897** 160	Precinorm Protein (3 x 1 mL, for USA)	Code 302	
**05117003** 190	PreciControl ClinChem Multi 1 (20 x 5 mL)	Code 391	
**05947626** 190	PreciControl ClinChem Multi 1 (4 x 5 mL)	Code 391	
**05947626** 160	PreciControl ClinChem Multi 1 (4 x 5 mL, for USA)	Code 391	
**04489357** 190	Diluent NaCl 9 % (50 mL)	System-ID 07 6869 3	

English

System information

For **cobas c** 311/501 analyzers:

CRPHS: ACN 217

For **cobas c** 502 analyzer:

CRPHS: ACN 8217

Intended use

In vitro test for the quantitative determination of C-reactive protein (CRP) in human serum and plasma on Roche/Hitachi **cobas c** systems. Measurement of CRP is of use for the detection and evaluation of inflammatory disorders and associated diseases, infection and tissue injury. Highly sensitive measurement of CRP may also be used as an aid in the assessment of the risk of future coronary heart disease. When used as an adjunct to other laboratory evaluation methods of acute coronary syndromes, it may also be an additional independent indicator of recurrent event prognosis in patients with stable coronary disease or acute coronary syndrome.

Summary[1,2,3,4,5,6,7,8,9,10,11,12,13,14,15,16,17,18,19,20,21]

C-reactive protein is the classic acute phase protein in inflammatory reactions. It is synthesized by the liver and consists of five identical polypeptide chains that form a five-member ring having a molecular weight of 105000 daltons. CRP is the most sensitive of the acute phase reactants and its concentration increases rapidly during inflammatory processes. Complexed CRP activates the complement system beginning with C1q. CRP then initiates opsonization and phagocytosis of invading cells, but its main function is to bind and detoxify endogenous toxic substances produced as a result of tissue damage.

CRP assays are used to detect systemic inflammatory processes (apart from certain types of inflammation such as SLE and Colitis ulcerosa); to assess treatment of bacterial infections with antibiotics; to detect intrauterine infections with concomitant premature amniorrhexis; to differentiate between active and inactive forms of disease with concurrent infection, e.g. in patients suffering from SLE or Colitis ulcerosa; to therapeutically monitor rheumatic disease and assess anti-inflammatory therapy; to determine the presence of post-operative complications at an early stage, such as infected wounds, thrombosis and pneumonia, and to distinguish between infection and bone marrow transplant rejection.

Sensitive CRP measurements have been used and discussed for early detection of infection in pediatrics and risk assessment of coronary heart disease. Several studies came to the conclusion that the highly sensitive measurement of CRP could be used as a marker to predict the risk of coronary heart disease in apparently healthy persons and as an indicator of recurrent event prognosis. Increases in CRP values are non-specific and should not be interpreted without a complete clinical history. The American Heart Association and the Centers for Disease Control and Prevention have made several recommendations concerning the use of high sensitivity C-Reactive Protein (hsCRP) in cardiovascular risk assessment.[21] Testing for any risk assessment should not be performed while there is an indication of infection, systemic inflammation or trauma. Patients with persistently unexplained hsCRP levels above 10 mg/L (95.2 nmol/L) should be evaluated for non-cardiovascular etiologies. When using hsCRP to assess the risk of coronary heart disease, measurements should be made on metabolically stable patients and compared to previous values.

Optimally, the average of hsCRP results repeated two weeks apart should be used for risk assessment. Screening the entire adult population for hsCRP is not recommended, and hsCRP is not a substitute for traditional cardiovascular risk factors. Acute coronary syndrome management should not depend solely on hsCRP measurements. Similarly, application of secondary prevention measures should be based on global risk assessment and not solely on hsCRP measurements. Serial measurements of hsCRP should not be used to monitor treatment.

Various assay methods are available for CRP determination, such as nephelometry and turbidimetry. The Roche CRP assay is based on the principle of particle-enhanced immunological agglutination.

Test principle[22,23]

Particle enhanced immunoturbidimetric assay.

Human CRP agglutinates with latex particles coated with monoclonal anti-CRP antibodies. The precipitate is determined turbidimetrically.

Reagents - working solutions

R1 TRIS buffer with bovine serum albumin and immunoglobulins (mouse); preservative; stabilizers

R2 Latex particles coated with anti-CRP (mouse) in glycine buffer; preservative; stabilizers

R1 is in position B and R2 is in position C.

Precautions and warnings

For in vitro diagnostic use.
Exercise the normal precautions required for handling all laboratory reagents.
Disposal of all waste material should be in accordance with local guidelines.
Safety data sheet available for professional user on request.

For USA: Caution: Federal law restricts this device to sale by or on the order of a physician.

Reagent handling

Ready for use

Mix **cobas c** pack well before placing on the analyzer.

Carefully invert reagent container several times prior to use to ensure that the reagent components are mixed.

Storage and stability

CRPHS

Shelf life at 2-8 °C:	See expiration date on **cobas c** pack label.
On-board in use and refrigerated on the analyzer:	12 weeks

Diluent NaCl 9 %

Shelf life at 2-8 °C:	See expiration date on **cobas c** pack label.
On-board in use and refrigerated on the analyzer:	12 weeks

Woldeyohannes M, *et al. BMJ Open* 2023; 13:e067641. doi: 10.1136/bmjopen-2022-067641

0004628918190c501V12.0

CRPHS

Cardiac C-Reactive Protein (Latex) High Sensitive

Specimen collection and preparation

For specimen collection and preparation only use suitable tubes or collection containers.

Only the specimens listed below were tested and found acceptable.
Serum.
Plasma: Li-heparin and K_2-EDTA plasma

The sample types listed were tested with a selection of sample collection tubes that were commercially available at the time of testing, i.e. not all available tubes of all manufacturers were tested. Sample collection systems from various manufacturers may contain differing materials which could affect the test results in some cases. When processing samples in primary tubes (sample collection systems), follow the instructions of the tube manufacturer.

Centrifuge samples containing precipitates before performing the assay.

See the limitations and interferences section for details about possible sample interferences.

Sample stability claims were established by experimental data by the manufacturer or based on reference literature and only for the temperatures/time frames as stated in the method sheet. It is the responsibility of the individual laboratory to use all available references and/or its own studies to determine specific stability criteria for its laboratory.

Stability:[24]	11 days at 15-25 °C
	2 months at 2-8 °C
	3 years at (-15)-(-25) °C

Materials provided

See “Reagents – working solutions” section for reagents.

Materials required (but not provided)

- See “Order information” section
- General laboratory equipment

Assay

For optimum performance of the assay follow the directions given in this document for the analyzer concerned. Refer to the appropriate operator’s manual for analyzer-specific assay instructions.

The performance of applications not validated by Roche is not warranted and must be defined by the user.

Application for serum and plasma

cobas c 311 **test definition**

Assay type	Rate A	
Reaction time / Assay points	10/7-57	
Wavelength (sub/main)	– /546 nm	
Reaction direction	Increase	
Units	mg/L (nmol/L, mg/dL)	
Reagent pipetting		Diluent (H_2O)
R1	82 µL	42 µL
R2	28 µL	20 µL

Sample volumes	*Sample*	*Sample dilution*	
		Sample	*Diluent (NaCl)*
Normal	6 µL	–	–
Decreased	6 µL	10 µL	140 µL
Increased	6 µL	–	–

cobas c 501 **test definition**

Assay type	Rate A	
Reaction time / Assay points	10/12-70	
Wavelength (sub/main)	– /546 nm	
Reaction direction	Increase	
Units	mg/L (nmol/L, mg/dL)	
Reagent pipetting		Diluent (H_2O)
R1	82 µL	42 µL
R2	28 µL	20 µL

Sample volumes	*Sample*	*Sample dilution*	
		Sample	*Diluent (NaCl)*
Normal	6 µL	–	–
Decreased	6 µL	10 µL	140 µL
Increased	6 µL	–	–

cobas c 502 **test definition**

Assay type	Rate A	
Reaction time / Assay points	10/12-70	
Wavelength (sub/main)	– /546 nm	
Reaction direction	Increase	
Units	mg/L (nmol/L, mg/dL)	
Reagent pipetting		Diluent (H_2O)
R1	82 µL	42 µL
R2	28 µL	20 µL

Sample volumes	*Sample*	*Sample dilution*	
		Sample	*Diluent (NaCl)*
Normal	6 µL	–	–
Decreased	6 µL	10 µL	140 µL
Increased	12 µL	–	–

Calibration

Calibrators	S1: H_2O	
	S2: C.f.a.s. Proteins	
	Multiply the lot-specific C.f.a.s. Proteins calibrator value by the factors below to determine the standard concentrations for the 6-point calibration curve:	
	S2: 0.0125	S5: 0.100
	S3: 0.0250	S6: 0.200
	S4: 0.0500	
Calibration mode	Line Graph	
Calibration frequency	Full calibration<br>• after reagent lot change<br>• as required following quality control procedures	

Calibration interval may be extended based on acceptable verification of calibration by the laboratory.

Traceability: This method has been standardized against the reference preparation of the IRMM (Institute for Reference Materials and Measurements) BCR470/CRM470 (RPPHS - Reference Preparation for Proteins in Human Serum).[25]

Quality control

For quality control, use control materials as listed in the "Order information" section.

In addition, other suitable control material can be used.

The control intervals and limits should be adapted to each laboratory’s individual requirements. Values obtained should fall within the defined

0004628918190c501V12.0

CRPHS

cobas®

Cardiac C-Reactive Protein (Latex) High Sensitive

limits. Each laboratory should establish corrective measures to be taken if values fall outside the defined limits.

Follow the applicable government regulations and local guidelines for quality control.

Calculation

Roche/Hitachi **cobas c** systems automatically calculate the analyte concentration of each sample.

Conversion factors:	mg/L x 9.52 = nmol/L
	mg/L x 0.1 = mg/dL

Limitations - interference

Criterion: Recovery within ± 10 % of initial values at CRP levels of 1.0 mg/L.

Icterus:[26] No significant interference up to an I index of 60 for conjugated bilirubin and unconjugated bilirubin (approximate conjugated and unconjugated bilirubin concentration: 60 mg/dL or 1026 µmol/L).

Hemolysis:[26] No significant interference up to an H index of 1000 (approximate hemoglobin concentration: 622 µmol/L or 1000 mg/dL).

Lipemia (Intralipid):[26] No significant interference up to an L index of 600. There is poor correlation between the L index (corresponds to turbidity) and triglycerides concentration.

Rheumatoid factors: No significant interference from rheumatoid factors up to a concentration of 1200 IU/mL.

Drugs: No interference was found at therapeutic concentrations using common drug panels.[27,28]

Therapeutic drugs: Significantly decreased CRP values may be obtained from samples taken from patients who have been treated with carboxypenicillins.

High dose hook-effect: No false result occurs up to a CRP concentration of 1000 mg/L.

In very rare cases, gammopathy, in particular type IgM (Waldenström’s macroglobulinemia), may cause unreliable results.[29]

Although measures were taken to minimize interference caused by human anti-mouse antibodies, erroneous findings may be obtained from samples taken from patients who have been treated with monoclonal mouse antibodies or have received them for diagnostic purposes.

For diagnostic purposes, the results should always be assessed in conjunction with the patient’s medical history, clinical examination and other findings.

ACTION REQUIRED
Special Wash Programming: The use of special wash steps is mandatory when certain test combinations are run together on Roche/Hitachi **cobas c** systems. The latest version of the carry-over evasion list can be found with the NaOHD-SMS-SmpCln1+2-SCCS Method Sheets. For further instructions refer to the operator’s manual. **cobas c** 502 analyzer: All special wash programming necessary for avoiding carry-over is available via the **cobas** link, manual input is required in certain cases.

Where required, special wash/carry-over evasion programming must be implemented prior to reporting results with this test.

Limits and ranges

Measuring range

0.15-20.0 mg/L (1.43-190 nmol/L, 0.015-2.0 mg/dL)

Determine samples having higher concentrations via the rerun function. Dilution of samples via the rerun function is a 1:15 dilution. Results from samples diluted using the rerun function are automatically multiplied by a factor of 15.

Lower limits of measurement

Lower detection limit of the test

0.15 mg/L (1.43 nmol/L, 0.015 mg/dL)

The lower detection limit represents the lowest measurable analyte level that can be distinguished from zero. It is calculated as the value lying 3 standard deviations above that of the lowest standard (standard 1 + 3 SD, repeatability, n = 21).

Functional sensitivity

0.3 mg/L (2.96 nmol/L, 0.03 mg/dL)

The functional sensitivity is the lowest CRP concentration that can be reproducibly measured with an inter-assay coefficient of variation of < 10 %.

Expected values

Consensus reference interval for adults:[30]

IFCC/CRM 470

mg/dL	mg/L	nmol/L
< 0.5	< 5.0	< 47.6

The CDC/AHA recommended the following hsCRP cut-off points (tertiles) for CVD risk assessment:[21,31]

hsCRP level (mg/L)	hsCRP level (nmol/L)	Relative risk
< 1.0	< 9.52	low
1.0-3.0	9.52-28.6	average
> 3.0	> 28.6	high

Patients with higher hsCRP concentrations are more likely to develop myocardial infarction and severe peripheral vascular disease.

5-95 % reference intervals of neonates and children:[32]

Neonates (0-3 weeks): 0.1-4.1 mg/L (0.95-39.0 nmol/L)

Children (2 months-15 years): 0.1-2.8 mg/L (0.95-26.7 nmol/L)

It is important to monitor the CRP concentration during the acute phase of the illness.

Roche has not evaluated reference ranges in a pediatric population.

Each laboratory should investigate the transferability of the expected values to its own patient population and if necessary determine its own reference ranges.

Increases in CRP values are non-specific and should not be interpreted without a complete clinical history.

When using hsCRP to assess the risk of coronary heart disease, measurements should be made on metabolically stable patients and compared to previous values. Optimally, the average of hsCRP results repeated two weeks apart should be used for risk assessment. Measurements should be compared to previous values. When the results are being used for risk assessment, patients with persistently unexplained hsCRP levels of above 10 mg/L (95.2 nmol/L) should be evaluated for non-cardiovascular origins. Testing for any risk assessment should not be performed while there is indication of infection, systemic inflammation or trauma.[21]

Specific performance data

Representative performance data on the analyzers are given below. Results obtained in individual laboratories may differ.

Precision

Precision was determined using human samples and controls in an internal protocol with repeatability (n = 21) and intermediate precision (3 aliquots per run, 1 run per day, 21 days). The following results were obtained:

Repeatability	*Mean*	*SD*	*CV*
	mg/L (nmol/L, mg/dL)	*mg/L (nmol/L, mg/dL)*	*%*
Precinorm Protein	9.00 (85.7, 0.900)	0.10 (1.0, 0.010)	1.2
CRP T Control N	4.34 (41.3, 0.434)	0.04 (0.4, 0.004)	1.0
Human serum 1	15.9 (151, 1.59)	0.1 (1, 0.01)	0.4
Human serum 2	0.54 (5.14, 0.054)	0.01 (0.10, 0.001)	1.6

Intermediate precision	*Mean*	*SD*	*CV*
	mg/L (nmol/L, mg/dL)	*mg/L (nmol/L, mg/dL)*	*%*
Precinorm Protein	9.06 (86.3, 0.906)	0.11 (1.1, 0.011)	1.3
CRP T Control N	4.28 (40.8, 0.428)	0.11 (1.1, 0.011)	2.6
Human serum 3	13.3 (126, 1.33)	0.3 (3, 0.03)	2.1
Human serum 4	0.53 (5.05, 0.053)	0.05 (0.48, 0.005)	8.4

Method comparison

CRP values for human serum and plasma samples obtained on a Roche/Hitachi **cobas c** 501 analyzer (y) were compared with those

Woldeyohannes M, *et al. BMJ Open* 2023; 13:e067641. doi: 10.1136/bmjopen-2022-067641

0004628918190c501V12.0

CRPHS

cobas®

Cardiac C-Reactive Protein (Latex) High Sensitive

determined using the corresponding reagent on a Roche/Hitachi 917 analyzer (x).

Sample size (n) = 192

Passing/Bablok[33]	Linear regression
y = 0.992x + 0.254 mg/L	y = 0.946x + 0.514 mg/L
τ = 0.944	r = 0.996

The sample concentrations were between 0.500 and 19.7 mg/L (4.76 and 188 nmol/L, 0.050 and 1.97 mg/dL).

References

1 Henry JB, ed. Clinical Diagnosis and Management by Laboratory Methods. Vol II. Philadelphia, Pa: WB Saunders 1979.

2 Greiling H, Gressner AM, eds. Lehrbuch der Klinischen Chemie und Pathobiochemie, 3rd ed. Stuttgart/New York: Schattauer Verlag 1995:234-236.

3 Thomas L, Messenger M. Pathobiochemie und Labordiagnostik der Entzündung. Lab med 1993;17:179–194.

4 Young B, Gleeson M, Cripps AW. C-reactive protein: A critical review. Pathology 1991;23:118-124.

5 Tietz NW. Clinical Guide to Laboratory Tests. 3rd ed. Philadelphia, Pa: WB Saunders Co; 1995.

6 Wasunna A, Whitelaw A, Gallimore R, et al. C-reactive protein and bacterial infection in preterm infants. Eur J Pediatr 1990 Mar;149(6):424-427.

7 Liuzzo G, Biasucci LM, Gallimore JR, et al. The prognostic value of C-reactive protein and serum amyloid A protein in severe unstable angina. N Engl J Med 1994;331:417-424.

8 Kuller LH, Tracy RP, Shaten J, et al. Relation of c-reactive protein and coronary heart disease in the MRFIT nested case control study. Am J Epidem 1996;144:537-547.

9 Ridker PM, Glynn RJ, Hennekens CH, et al. C-Reactive Protein Adds to the Predictive Value of Total and HDL Cholesterol in Determining Risk of First Myocardial Infarction. Circulation 1998;97:2007-2011.

10 Ridker PM, Cushman M, Stampfer MJ, et al. Plasma Concentration of C-Reactive Protein and Risk of Developing Peripheral Vascular Disease. Circulation 1998;97:425-428.

11 Ridker PM, Cushman M, Stampfer MJ, et al. Inflammation, Aspirin, and the Risk of Cardiovascular Disease in Apparently Healthy Men. N Eng J Med 1997;336(14):973-979.

12 Danesh J, Wheeler JG, Hirschfield GM, et al. C-Reactive Protein and Other Circulating Markers of Inflammation in the Prediction of Coronary Heart Disease. N Eng J Med 2004;350(14):1387-1397.

13 Ridker PM, Hennekens CH, Buring JE, et al. C-Reactive Protein and Other Markers of Inflammation in the Prediction of Cardiovascular Disease in Women. N Engl J Med 2000;342(12):836-843.

14 Tracy RP, Lemaitre RN, Psaty BM, et al. Relationship of C-Reactive Protein to Risk of Cardiovascular Disease in the Elderly. Arterioscler Thromb Vasc Biol 1997;17:1121-1127.

15 Järvisalo MJ, Harmoinen A, Hakanen M, et al. Elevated Serum C-Reactive Protein Levels and Early Arterial Changes in Healthy Children. Arterioscler Thromb Vasc Biol, (August) 2002;1323-1328.

16 Almagor M, Keren A, Banai S. Increased C-Reactive Protein Level after Coronary Stent Implantation in Patients with Stable Coronary Artery Disease. American Heart Journal 2003;145 (2):248-253.

17 Katritsis D, Korovesis S, Giazitzoglou E, et al. C-Reactive Protein Concentrations and Angiographic Characteristics of Coronary Lesions. Clin Chem 2001;47(5):882-886.

18 Beattie MS, Shlipak, MG, Liu H, et al. C-Reactive Protein and Ischemia in Users and Nonusers of β-Blockers and Statins. Circulation 2003;107:245-250.

19 Plenge JK, Hernandez TL, Weil KM, et al. Simvastatin Lowers C-Reactive Protein Within 14 Days. An Effect Independent of Low-Density Lipoprotein Cholesterol Reduction. Circulation 2002;106:1447-1452.

20 Lindahl B, Toss H, Siegbahn A, et al. Markers of Myocardial Damage and Inflammation in Relation to Long-Term Mortality in Unstable Coronary Artery Disease. N Eng J Med 2000;343(16):1139-1147.

21 Pearson TA, Mensah GA, Alexander RW, et al. Markers of Inflammation and Cardiovascular Disease. Application to Clinical and Public Health Practice. A Statement for Healthcare Professionals From the Centers for Disease Control and Prevention and the American Heart Association. Circulation 2003;107:499-511.

22 Price CP, Trull AK, Berry D, et al. Development and validation of a particle-enhanced turbidimetric immunoassay for C-reactive protein. J Immunol Methods 1987;99:205-211.

23 Eda S, Kaufmann J, Roos W, et al. Development of a New Microparticle-Enhanced Turbidimetric Assay for C-reactive Protein with Superior Features in Analytical Sensitivity and Dynamic Range. J Clin Lab Anal 1998;12:137-144.

24 Use of Anticoagulants in Diagnostic Laboratory Investigations. WHO Publication WHO/DIL/LAB/99.1 Rev. 2: Jan 2002.

25 Baudner S, Bienvenu J, Blirup-Jensen S, et al. The certification of a matrix reference material for immunochemical measurement of 14 human serum proteins CRM470. Report EUR 15243 EN 1993;1-186.

26 Glick MR, Ryder KW, Jackson SA. Graphical Comparisons of Interferences in Clinical Chemistry Instrumentation. Clin Chem 1986;32:470-475.

27 Breuer J. Report on the Symposium “Drug effects in Clinical Chemistry Methods”. Eur J Clin Chem Clin Biochem 1996;34:385-386.

28 Sonntag O, Scholer A. Drug interference in clinical chemistry: recommendation of drugs and their concentrations to be used in drug interference studies. Ann Clin Biochem 2001;38:376-385.

29 Bakker AJ, Mücke M. Gammopathy interference in clinical chemistry assays: mechanisms, detection and prevention. Clin Chem Lab Med 2007;45(9):1240-1243.

30 Dati F, Schumann G, Thomas L, et al. Consensus of a group of professional societies and diagnostic companies on guidelines for interim reference ranges for 14 proteins in serum based on the standardization against the IFCC/BCR/CAP reference material (CRM 470). Eur J Clin Chem Clin Biochem 1996;34:517-520.

31 Ridker PM. Clinical Application of C-Reactive Protein for Cardiovascular Disease Detection and Prevention. Circulation 2003;107:363-369.

32 Schlebusch H, Liappis N, Kalina E, et al. High Sensitive CRP and Creatinine: Reference Intervals from Infancy to Childhood. J Lab Med 2002;26:341-346.

33 Bablok W, Passing H, Bender R, et al. A general regression procedure for method transformation. Application of linear regression procedures for method comparison studies in clinical chemistry, Part III. J Clin Chem Clin Biochem 1988 Nov;26(11):783-790.

A point (period/stop) is always used in this Method Sheet as the decimal separator to mark the border between the integral and the fractional parts of a decimal numeral. Separators for thousands are not used.

Symbols

Roche Diagnostics uses the following symbols and signs in addition to those listed in the ISO 15223-1 standard (for USA: see https://usdiagnostics.roche.com for definition of symbols used):

CONTENT	Contents of kit
→	Volume after reconstitution or mixing
GTIN	Global Trade Item Number

0004628918190c501V12.0

CRPHS

cobas®

Cardiac C-Reactive Protein (Latex) High Sensitive

FOR US CUSTOMERS ONLY: LIMITED WARRANTY

Roche Diagnostics warrants that this product will meet the specifications stated in the labeling when used in accordance with such labeling and will be free from defects in material and workmanship until the expiration date printed on the label. THIS LIMITED WARRANTY IS IN LIEU OF ANY OTHER WARRANTY, EXPRESS OR IMPLIED, INCLUDING ANY IMPLIED WARRANTY OF MERCHANTABILITY OR FITNESS FOR PARTICULAR PURPOSE. IN NO EVENT SHALL ROCHE DIAGNOSTICS BE LIABLE FOR INCIDENTAL, INDIRECT, SPECIAL OR CONSEQUENTIAL DAMAGES.

COBAS, COBAS C, PRECICONTROL and PRECINORM are trademarks of Roche.
All other product names and trademarks are the property of their respective owners.
Additions, deletions or changes are indicated by a change bar in the margin.
© 2019, Roche Diagnostics

CE

Roche Diagnostics GmbH, Sandhofer Strasse 116, D-68305 Mannheim
www.roche.com

Distribution in USA by:
Roche Diagnostics, Indianapolis, IN
US Customer Technical Support 1-800-428-2336

Roche
