# Supplementary material for: Ethiopia National Food and Nutrition Survey to inform the Ethiopian National Food and Nutrition Strategy: a study protocol
Source: BMJ Open. 2023 Apr 25;13(4):e067641. doi: 10.1136/bmjopen-2022-067641 (PMC10151871; doi:10.1136/bmjopen-2022-067641)
Supplement: Supplementary data [file bmjopen-2022-067641supp011.pdf]

07559992500V4.0

Elecsys Folate III

cobas®

REF	(Consult instructions)	Σ	SYSTEM
07559992190	07559992500	100	**cobas e** 411<br>**cobas e** 601<br>**cobas e** 602

English

System information

For **cobas e** 411 analyzer: test number 1520
For **cobas e** 601 and **cobas e** 602 analyzers: Application Code Number 721

Intended use

Binding assay for the in vitro quantitative determination of folate in human serum and plasma.

The binding assay is intended for use on Elecsys and **cobas e** immunoassay analyzers.

Summary

Folate belongs to the family of B-group vitamins composed of an aromatic pteridine ring linked through a methylene group to p-aminobenzoic acid and a glutamate residue. Folate (folic acid) is vital for normal cellular functions and plays an essential role in nucleic acid synthesis, methionine regeneration, shuttling and redox reactions of one-carbon units required for normal metabolism and regulation.[1,2]

The folate metabolism can be exemplified as a cycle, where folate facilitates the transfer of one-carbon units from one molecule to another required in various biochemical reactions: for example, tetrahydrofolate (THF) accepts a single carbon unit from serine, which is reduced in a number of steps to 5-methyltetrahydrofolate (5-MTHF). 5-MTHF gives its methyl group to homocysteine, which is - with involvement of methionine synthase and vitamin B12 - enzymatically converted to methionine. The resulting THF starts again the cycle of methyl group synthesis. From methionine, the methyl groups are transferred to S-adenosylmethionine (SAM).[3] SAM serves as a methyl group donor in several methylation reactions, like DNA, RNA and protein methylation.[1]

The methionine cycle is highly sensitive to folate deficiency: with a low folate status, the ability of the cell to re-methylate homocysteine is impaired and this results in increased homocysteine concentrations in plasma.[2]

Folate also plays an essential role in the synthesis of purine and pyrimidine precursors of nucleic acids. Altered distribution of methyl groups and impaired DNA synthesis play an essential role in the development of cancers. Abnormal folate status has also been linked with the development of diseases like cardiovascular diseases, neural tube defects, cleft lip and palate, late pregnancy complications, neurodegenerative and psychiatric disorders.[1,2]

Folate belongs to the group of essential vitamins, i.e. it cannot be synthesized by the human organism and therefore must be absorbed from diet. Primary sources of folates are green and leafy vegetables, sprouts, fruits, brewer's yeast and liver.[1,2]

Folate deficiency can be caused by decreased nutritional intake, poor absorption of ingested folate in the intestine or increased demand of folate, for example during physical activity or pregnancy. Deficiency of folate can also be a result of liver diseases or impaired folate metabolism due to genetic defects or drug interactions.[2]

A clinical manifestation of both folate and vitamin B12 deficiency is the so called megaloblastic (macrocytic) anemia: due to the affected DNA synthesis and cell maturation, especially involving the cells of erythropoiesis, the total count of erythrocytes is significantly reduced. The hemoglobin synthesis capacity however is normal, which leads to abnormally large erythrocyte precursors ("macrocytes" or "megaloblasts"), which have an elevated hemoglobin content ("hyperchromic anemia").[3,4]

Because vitamin B12 and folate are closely interrelated in the cellular one-carbon unit metabolism, and also hematologic and clinical consequences of the two vitamin deficiency states might be similar, it is advisable to determine both parameters simultaneously in patients with the relevant symptoms of vitamin deficiency.[3,4]

Test principle

Competition principle. Total duration of assay: 27 minutes.

- 1st incubation: By incubating 25 µL of sample with the folate pretreatment reagents 1 and 2, bound folate is released from endogenous folate binding proteins.
- 2nd incubation: By incubating the pretreated sample with the ruthenium labeled folate binding protein, a folate complex is formed, the amount of which is dependent upon the analyte concentration in the sample.
- 3rd incubation: After addition of streptavidin-coated microparticles and folate labeled with biotin, the unbound sites of the ruthenium labeled folate binding protein become occupied, with formation of a ruthenium labeled folate binding protein-folate biotin complex. The entire complex becomes bound to the solid phase via interaction of biotin and streptavidin.
- The reaction mixture is aspirated into the measuring cell where the microparticles are magnetically captured onto the surface of the electrode. Unbound substances are then removed with ProCell/ProCell M. Application of a voltage to the electrode then induces chemiluminescent emission which is measured by a photomultiplier.
- Results are determined via a calibration curve which is instrument-specifically generated by 2-point calibration and a master curve provided via the reagent barcode or e-barcode.

Reagents - working solutions

The reagent rackpack (M, R1, R2) and the pretreatment reagents (PT1, PT2) are labeled as Fol III.

PT1 Pretreatment reagent 1 (white cap), 1 bottle, 4 mL:
Sodium 2-mercaptoethanesulfonate (MESNA) 40 g/L, pH 5.5.

PT2 Pretreatment reagent 2 (gray cap), 1 bottle, 5 mL:
Sodium hydroxide 25 g/L.

M Streptavidin-coated microparticles (transparent cap), 1 bottle, 6.5 mL:
Streptavidin-coated microparticles 0.72 mg/mL; preservative.

R1 Folate binding protein~$Ru(bpy)_3^{2+}$ (gray cap), 1 bottle, 9 mL:
Ruthenium labeled folate binding protein 75 µg/L; human serum albumin (stabilizer); borate/phosphate/citrate buffer 70 mmol/L, pH 5.5; preservative.

R2 Folate~biotin (black cap), 1 bottle, 8 mL:
Biotinylated folate 17 µg/L; biotin 120 µg/L; human serum albumin (stabilizer); borate buffer 100 mmol/L, pH 9.0; preservative.

Precautions and warnings

For in vitro diagnostic use.
Exercise the normal precautions required for handling all laboratory reagents.
Disposal of all waste material should be in accordance with local guidelines.
Safety data sheet available for professional user on request.

This kit contains components classified as follows in accordance with the Regulation (EC) No. 1272/2008:

2-methyl-2H-isothiazol-3-one hydrochloride

EUH 208 May produce an allergic reaction.

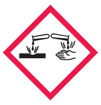

Danger

H290 May be corrosive to metals.

Woldeyohannes M, *et al. BMJ Open* 2023; 13:e067641. doi: 10.1136/bmjopen-2022-067641

07559992500V4.0

Elecsys Folate III

cobas®

H314	Causes severe skin burns and eye damage.

Prevention:

P280	Wear protective gloves/ protective clothing/ eye protection/ face protection.

Response:

P301 + P330 + P331	IF SWALLOWED: Rinse mouth. Do NOT induce vomiting.
P303 + P361 + P353	IF ON SKIN (or hair): Take off immediately all contaminated clothing. Rinse skin with water.
P304 + P340 + P310	IF INHALED: Remove person to fresh air and keep comfortable for breathing. Immediately call a POISON CENTER/ doctor.
P305 + P351 + P338 + P310	IF IN EYES: Rinse cautiously with water for several minutes. Remove contact lenses, if present and easy to do. Continue rinsing. Immediately call a POISON CENTER/ doctor.
P390	Absorb spillage to prevent material damage.

Product safety labeling follows EU GHS guidance.

Contact phone: all countries: +49-621-7590

All human material should be considered potentially infectious. All products derived from human blood are prepared exclusively from the blood of donors tested individually and shown to be free from HBsAg and antibodies to HCV and HIV. The testing methods used assays approved by the FDA or cleared in compliance with the European Directive 98/79/EC, Annex II, List A.

However, as no testing method can rule out the potential risk of infection with absolute certainty, the material should be handled with the same level of care as a patient specimen. In the event of exposure, the directives of the responsible health authorities should be followed.[5,6]

Avoid foam formation in all reagents and sample types (specimens, calibrators and controls).

Reagent handling

The reagents in the kit have been assembled into a ready-for-use unit that cannot be separated.

All information required for correct operation is read in from the respective reagent barcodes.

Storage and stability

Store at 2-8 °C.

Do not freeze.

Store the Elecsys reagent kit **upright** in order to ensure complete availability of the microparticles during automatic mixing prior to use.

Stability:	
unopened at 2-8 °C	up to the stated expiration date
after opening at 2-8 °C	56 days (8 weeks)
on the analyzers	14 days (2 weeks) onboard<br>or<br>28 days (4 weeks) when stored alternatively in the refrigerator and on the analyzer, with the total time onboard on the analyzer not exceeding 10 x 8 hours

Specimen collection and preparation

Only the specimens listed below were tested and found acceptable.

Serum collected using standard sampling tubes or tubes containing separating gel.

Li-heparin plasma. Li-heparin plasma tubes containing separating gel can be used.

Criterion: Method comparison serum versus Li-heparin plasma, slope 0.9-1.1 + intercept within < ± 2x Limit of Blank (LoB), coefficient of correlation ≥ 0.95.

Serum: Stable for 2 hours at 15-25 °C, 48 hours at 2-8 °C, 28 days at -20 °C (± 5 °C). Freeze only once. Protect from light. Store the samples at 2-8 °C if they cannot be measured immediately.

Li-heparin plasma: Stable for 2 hours at 15-25 °C, 48 hours at 2-8 °C, 28 days at -20 °C (± 5 °C). Freeze only once. Protect from light. Store the samples at 2-8 °C if they cannot be measured immediately.

The sample types listed were tested with a selection of sample collection tubes that were commercially available at the time of testing, i.e. not all available tubes of all manufacturers were tested. Sample collection systems from various manufacturers may contain differing materials which could affect the test results in some cases. When processing samples in primary tubes (sample collection systems), follow the instructions of the tube manufacturer.

Samples should not subsequently be altered with additives (biocides, anti-oxidants or substances possibly changing the pH of the sample) in order to avoid erroneous folate recovery.

Centrifuge samples containing precipitates before performing the assay.

Do not use heat-inactivated samples.

Ensure the samples, calibrators and controls are at 20-25 °C prior to measurement.

Due to possible evaporation effects, samples, calibrators and controls on the analyzers should be analyzed/measured within 2 hours.

Note: Hemolysis may significantly increase folate values due to high concentrations of folate in red blood cells. Therefore, hemolyzed samples are not suitable for use in this assay. Samples for folate determinations should be collected from fasting persons.

Materials provided

See “Reagents – working solutions” section for reagents.

Materials required (but not provided)

- REF 07560001190, Folate III CalSet, for 4 x 1.0 mL
- REF 05618860190, PreciControl Varia, for 4 x 3.0 mL
- REF 11732277122, Diluent Universal, 2 x 16 mL sample diluent or REF 03183971122, Diluent Universal, 2 x 36 mL sample diluent
- General laboratory equipment
- **cobas e** analyzer

Additional materials for the **cobas e** 411 analyzer:

- REF 11662988122, ProCell, 6 x 380 mL system buffer
- REF 11662970122, CleanCell, 6 x 380 mL measuring cell cleaning solution
- REF 11930346122, Elecsys SysWash, 1 x 500 mL washwater additive
- REF 11933159001, Adapter for SysClean
- REF 11706802001, AssayCup, 60 x 60 reaction cups
- REF 11706799001, AssayTip, 30 x 120 pipette tips
- REF 11800507001, Clean-Liner

Additional materials for **cobas e** 601 and **cobas e** 602 analyzers:

- REF 04880340190, ProCell M, 2 x 2 L system buffer
- REF 04880293190, CleanCell M, 2 x 2 L measuring cell cleaning solution
- REF 03023141001, PC/CC-Cups, 12 cups to prewarm ProCell M and CleanCell M before use
- REF 03005712190, ProbeWash M, 12 x 70 mL cleaning solution for run finalization and rinsing during reagent change
- REF 03004899190, PreClean M, 5 x 600 mL detection cleaning solution
- REF 12102137001, AssayTip/AssayCup, 48 magazines x 84 reaction cups or pipette tips, waste bags
- REF 03023150001, WasteLiner, waste bags
- REF 03027651001, SysClean Adapter M

Additional materials for all analyzers:

07559992500V4.0

Elecsys Folate III

cobas®

- REF 11298500316, ISE Cleaning Solution/Elecsys SysClean, 5 x 100 mL system cleaning solution

Assay

For optimum performance of the assay follow the directions given in this document for the analyzer concerned. Refer to the appropriate operator's manual for analyzer-specific assay instructions.

Resuspension of the microparticles takes place automatically prior to use. Read in the test-specific parameters via the reagent barcode. If in exceptional cases the barcode cannot be read, enter the 15-digit sequence of numbers.

cobas e 601 and **cobas e** 602 analyzers: PreClean M solution is necessary.

Bring the cooled reagents to approximately 20 °C and place on the reagent disk (20 °C) of the analyzer. Avoid foam formation. The system automatically regulates the temperature of the reagents and the opening/closing of the bottles.

Calibration

Traceability: This method has been standardized against the WHO International Standard NIBSC code: 03/178.

Every Elecsys reagent set has a barcoded label containing specific information for calibration of the particular reagent lot. The predefined master curve is adapted to the analyzer using the relevant CalSet.

Calibration frequency: Calibration must be performed once per reagent lot using fresh reagent (i.e. not more than 24 hours since the reagent kit was registered on the analyzer).

Calibration interval may be extended based on acceptable verification of calibration by the laboratory.

Renewed calibration is recommended as follows:

- after 1 month (28 days) when using the same reagent lot
- after 7 days (when using the same reagent kit on the analyzer)
- as required: e.g. quality control findings outside the defined limits

Quality control

For quality control, use PreciControl Varia.

In addition, other suitable control material can be used.

Controls for the various concentration ranges should be run individually at least once every 24 hours when the test is in use, once per reagent kit, and following each calibration.

The control intervals and limits should be adapted to each laboratory's individual requirements. Values obtained should fall within the defined limits. Each laboratory should establish corrective measures to be taken if values fall outside the defined limits.

If necessary, repeat the measurement of the samples concerned.

Follow the applicable government regulations and local guidelines for quality control.

Calculation

The analyzer automatically calculates the analyte concentration of each sample (either in nmol/L or ng/mL).

Conversion factors: nmol/L x 0.44 = ng/mL
ng/mL x 2.27 = nmol/L

Limitations - interference

The assay is unaffected by icterus (bilirubin ≤ 496 µmol/L or ≤ 29 mg/dL), lipemia (Intralipid ≤ 1500 mg/dL), biotin (≤ 86.1 nmol/L or ≤ 21 ng/mL), IgG ≤ 16 g/L, IgA ≤ 4.0 g/L and IgM ≤ 10 g/L.

Criterion: Recovery within ± 10 % of initial value with samples > 4 ng/mL and ≤ ± 0.4 ng/mL with samples ≤ 4 ng/mL.

Hemolysis may significantly increase folate values due to high concentrations of folate in red blood cells. Therefore, hemolyzed samples are not suitable for use in this assay.

Samples should not be taken from patients receiving therapy with high biotin doses (i.e. > 5 mg/day) until at least 8 hours following the last biotin administration.

No interference was observed from rheumatoid factors up to a concentration of 1000 IU/mL.

In vitro tests were performed on 16 commonly used pharmaceuticals and in addition on human erythropoietin. No interference with the assay was found.

It is contraindicated to measure samples of patients receiving therapy with certain pharmaceuticals, e.g. methotrexate or leucovorin, because of the cross-reactivity of folate binding protein with these compounds.

Samples with extremely high total protein concentrations (hyperproteinemia) are not suitable for use in this assay. Hyperproteinemia may be caused by, but not limited to, the following conditions: Lymphoma[7,8], bone marrow disorders such as multiple myeloma, monoclonal gammopathy of undetermined significance (MGUS), Waldenström macroglobulinemia, plasmocytoma[7,8,9,10,11,12,13], Amyloidosis[13,14]. Respective samples may lead to the formation of protein gel in the assay cup, which may cause a run abort. The critical total protein concentration is dependent upon the individual sample composition.

In rare cases, interference due to extremely high titers of antibodies to streptavidin and ruthenium can occur. These effects are minimized by suitable test design.

For diagnostic purposes, the results should always be assessed in conjunction with RBC folate, the patient's medical history, clinical examination, and other findings.

Limits and ranges

Measuring range

0.6-20.0 ng/mL or 1.36-45.4 nmol/L (defined by the Limit of Blank and the maximum of the master curve). Values below the Limit of Blank are reported as < 0.6 ng/mL (< 1.36 nmol/L). Values above the measuring range are reported as > 20.0 ng/mL (> 45.4 nmol/L).

Lower limits of measurement

Limit of Blank, Limit of Detection and Limit of Quantitation

Limit of Blank = 0.6 ng/mL (1.36 nmol/L)

Limit of Detection = 1.2 ng/mL (2.72 nmol/L)

Limit of Quantitation = 2.0 ng/mL (4.54 nmol/L)

The Limit of Blank, Limit of Detection and Limit of Quantitation were determined in accordance with the CLSI (Clinical and Laboratory Standards Institute) EP17-A requirements.

The Limit of Blank is the 95th percentile value from n ≥ 60 measurements of analyte-free samples over several independent series. The Limit of Blank corresponds to the concentration below which analyte-free samples are found with a probability of 95 %.

The Limit of Detection is determined based on the Limit of Blank and the standard deviation of low concentration samples. The Limit of Detection corresponds to the lowest analyte concentration which can be detected (value above the Limit of Blank with a probability of 95 %).

The Limit of Quantitation is defined as the lowest amount of analyte in a sample that can be accurately quantitated with a total allowable relative error of ≤ 20 %.

It has been determined using low concentration folate samples.

Dilution

Samples with folate concentrations above the measuring range can be diluted manually with Diluent Universal. The recommended dilution is 1:2. The concentration of the diluted sample must be > 8.5 ng/mL or > 19.3 nmol/L. After manual dilution, multiply the results by the dilution factor 2.

Expected values

Referring to "The American Journal of Clinical Nutrition"[15] serum folate (folic acid) values were found as follows:

Sex	Age	N	Median		2.5th-97.5th percentile	
	years		ng/mL	nmol/L	ng/mL	nmol/L
Both	all	23345	13.0	29.5	4.6-34.8	10.4-78.9
Male	all	11387	12.3	27.9	4.5-32.2	10.2-73.0
Female	all	11958	13.6	30.1	4.8-37.3	10.9-84.5
Both	4-11	3595	17.2	39.0	8.6-37.7	19.5-85.4
Both	12-19	6390	12.1	27.4	5.0-27.2	11.3-61.6
Both	20-59	8689	11.6	26.3	4.4-31.0	10.0-70.2

Woldeyohannes M, *et al. BMJ Open* 2023; 13:e067641. doi: 10.1136/bmjopen-2022-067641

Elecsys Folate III

cobas®

Sex	Age	N	Median		2.5th-97.5th percentile	
	years		ng/mL	nmol/L	ng/mL	nmol/L
Both	≥ 60	4671	16.6	37.6	5.6-45.8	12.7-103.8

These values were obtained in the USA during the National Health and Nutrition Examination Survey (NHANES), 1999-2004.

The values shown below were performed on samples from an apparently healthy population, using the Elecsys Folate III assay.

The calculation is based on 404 sera (177 men, 227 women). The age range was between 20 and 65 years. Pregnant or lactating women were excluded. The reference population was selected according to normal homocysteine values.

N	Median		2.5th-97.5th percentile	
	ng/mL	nmol/L	ng/mL	nmol/L
404	8.94	20.3	3.89-26.8	8.83-60.8

Please note: These values should only be used as a guideline.

It should be taken into consideration that differences in the expected values may exist with respect to population and dietary status.

Each laboratory should investigate the transferability of the expected values to its own patient population and if necessary determine its own reference ranges.

Folate deficient sample values

25 samples considered to be deficient[a)] in serum folate concentration were assessed using the Elecsys Folate III assay. All samples were found to be below the 2.5th percentile as given in the table above.

a) Folate deficiency was assessed by measurement of serum folate by two commercially available folate assays.

Specific performance data

Representative performance data on the analyzers are given below. Results obtained in individual laboratories may differ.

Precision

Precision was determined using Elecsys reagents, pooled human sera and controls in a protocol (EP5-A2) of the CLSI (Clinical and Laboratory Standards Institute): 2 runs per day in duplicate each for 21 days (n = 84). The following results were obtained:

**cobas e** 411 analyzer					
		**Repeatability**		**Intermediate precision**	
Sample	Mean	SD	CV	SD	CV
	ng/mL	ng/mL	%	ng/mL	%
Human serum 1	1.88	0.150	8.0	0.205	10.9
Human serum 2	3.92	0.200	5.1	0.318	8.1
Human serum 3	11.9	0.346	2.9	0.571	4.8
Human serum 4	13.4	0.301	2.2	0.574	4.3
Human serum 5	17.8	0.440	2.5	0.666	3.7
PreciControl Varia1	3.24	0.215	6.6	0.309	9.5
PreciControl Varia2	11.6	0.314	2.7	0.566	4.9

**cobas e** 411 analyzer					
		**Repeatability**		**Intermediate precision**	
Sample	Mean	SD	CV	SD	CV
	nmol/L	nmol/L	%	nmol/L	%
Human serum 1	4.27	0.341	8.0	0.465	10.9
Human serum 2	8.90	0.454	5.1	0.722	8.1
Human serum 3	27.0	0.785	2.9	1.30	4.8
Human serum 4	30.4	0.683	2.2	1.30	4.3
Human serum 5	40.4	0.999	2.5	1.51	3.7
PreciControl Varia1	7.35	0.488	6.6	0.701	9.5
PreciControl Varia2	26.3	0.713	2.7	1.28	4.9

**cobas e** 601 and **cobas e** 602 analyzers					
		**Repeatability**		**Intermediate precision**	
Sample	Mean	SD	CV	SD	CV
	ng/mL	ng/mL	%	ng/mL	%
Human serum 1	1.66	0.255	15.4	0.268	16.1
Human serum 2	4.10	0.219	5.4	0.303	7.4
Human serum 3	11.1	0.449	4.1	0.503	4.6
Human serum 4	12.2	0.454	3.7	0.467	3.8
Human serum 5	16.4	0.502	3.1	0.625	3.8
PreciControl Varia1	2.34	0.189	8.1	0.228	9.8
PreciControl Varia2	10.1	0.443	4.4	0.489	4.9

**cobas e** 601 and **cobas e** 602 analyzers					
		**Repeatability**		**Intermediate precision**	
Sample	Mean	SD	CV	SD	CV
	nmol/L	nmol/L	%	nmol/L	%
Human serum 1	3.77	0.579	15.4	0.608	16.1
Human serum 2	9.31	0.497	5.4	0.688	7.4
Human serum 3	25.2	1.02	4.1	1.14	4.6
Human serum 4	27.7	1.03	3.7	1.06	3.8
Human serum 5	37.2	1.14	3.1	1.42	3.8
PreciControl Varia1	5.31	0.429	8.1	0.518	9.8
PreciControl Varia2	22.9	1.01	4.4	1.11	4.9

Method comparison

a) A comparison of the Elecsys Folate III assay (traceable to WHO IS 03/178; y) and the Elecsys Folate III assay prior to standardization against WHO IS 03/178 (x) using clinical samples gave the following correlations (ng/mL):

Number of samples measured: 113

Passing/Bablok[16]	Linear regression
y = 1.14x - 1.97	y = 1.11x - 1.77
τ = 0.939	r = 0.994

The sample concentrations were between 2.1 and 18 ng/mL (4.8 and 41 nmol/L).

b) A comparison of the Elecsys Folate III assay (y) with a commercially available method (x) using clinical samples gave the following correlations (ng/mL):

Number of samples measured: 106

Passing/Bablok[16]	Linear regression
y = 0.980x - 0.095	y = 1.09x - 0.659
τ = 0.924	r = 0.984

The sample concentrations were between 1.9 and 17 ng/mL (4.3 and 39 nmol/L).

07559992500V4.0

Elecsys Folate III

cobas®

c) A comparison of the Elecsys Folate III assay on the **cobas e** 601 analyzer (y) with the Elecsys Folate III assay on the **cobas e** 411 analyzer (x) using clinical samples gave the following correlations (ng/mL):

Number of samples measured: 105

Passing/Bablok[16]	Linear regression
y = 1.05x - 0.303	y = 0.981x + 0.143
τ = 0.868	r = 0.982

The sample concentrations were between 1.6 and 19 ng/mL (3.6 and 43 nmol/L).

Analytical specificity

The following cross-reactivities were found, tested with folate concentrations of approximately 3.5 ng/mL, 10 ng/mL and 19 ng/mL.

Cross-reactant	Concentration tested ng/mL	Cross-reactivity %
Amethopterin	750	2.5
Aminopterin	750	4.4
Folinic acid	750	0.7

References

1 Nazki FH, Sameer AS, Ganaie BA. Folate: Metabolism, genes, polymorphisms and the associated diseases. Gene 2014;533(1):11-20.

2 Scaglione F, Panzavolta G. Folate, folic acid and 5-methyltetrahydrofolate are not the same thing. Xenobiotica 2014;44(5):480-488.

3 Reynolds EH. The neurology of folic acid deficiency. Handb Clin Neurol 2014;120:927-43.

4 Wick M, Pinggera W, Lehmann P. Clinical Aspects and Laboratory. Iron metabolism, Anemias. Springer Verlag, Wien, New York, 6th edition 2011:41-42.

5 Occupational Safety and Health Standards: Bloodborne pathogens. (29 CFR Part 1910.1030). Fed. Register.

6 Directive 2000/54/EC of the European Parliament and Council of 18 September 2000 on the protection of workers from risks related to exposure to biological agents at work.

7 Wu AHB. Tietz clinical guide to laboratory tests, 4th ed. St. Louis, Saunders/Elsevier 2006:608-609, 916-917.

8 Paricaud K, Moulis G, Combis MS, et al. Causes of protidemia above 100 g/L. Eur J Intern Med 2014;25:e123.

9 Filippatos TD, Liamis G, Christopoulou F, et al. Ten common pitfalls in the evaluation of patients with hyponatremia. Eur J Intern Med 2016;29:22-25.

10 Mailankody S, Landgren O. Monoclonal gammopathy of undetermined significance and Waldenström's macroglobulinemia. Best Pract Res Clin Haematol 2016;29:187-193.

11 Morel P, Duhamel A, Gobbi P, et al. International prognostic scoring system for Waldenström macroglobulinemia. Blood 2009;113:4163-4170.

12 Rajkumar SV. Multiple Myeloma. Curr Probl Cancer 2009;33:7-64.

13 Gertz MA. Immunoglobulin light chain amyloidosis: 2016 update on diagnosis, prognosis, and treatment. Am J Hematol 2016;91:947-956.

14 Wu AHB. Tietz clinical guide to laboratory tests, 4th ed. St. Louis, Saunders/Elsevier 2006: 916-917, 925.

15 Pfeiffer CM, Johnson CL, Jain RB, et al. Trends in blood folate and vitamin B-12 concentrations in the United States, 1988-2004. Am J Clin Nutr 2007;86:718-727.

16 Bablok W, Passing H, Bender R, et al. A general regression procedure for method transformation. Application of linear regression procedures for method comparison studies in clinical chemistry, Part III. J Clin Chem Clin Biochem 1988 Nov;26(11):783-790.

For further information, please refer to the appropriate operator's manual for the analyzer concerned, the respective application sheets, the product information and the Method Sheets of all necessary components (if available in your country).

A point (period/stop) is always used in this Method Sheet as the decimal separator to mark the border between the integral and the fractional parts of a decimal numeral. Separators for thousands are not used.

Symbols

Roche Diagnostics uses the following symbols and signs in addition to those listed in the ISO 15223-1 standard (for USA: see dialog.roche.com for definition of symbols used):

CONTENT	Contents of kit
SYSTEM	Analyzers/Instruments on which reagents can be used
REAGENT	Reagent
CALIBRATOR	Calibrator
⟶	Volume after reconstitution or mixing
GTIN	Global Trade Item Number

COBAS, COBAS E, ELECSYS and PRECICONTROL are trademarks of Roche. INTRALIPID is a trademark of Fresenius Kabi AB.

All other product names and trademarks are the property of their respective owners.

Additions, deletions or changes are indicated by a change bar in the margin.

© 2019, Roche Diagnostics

CE

Roche Diagnostics GmbH, Sandhofer Strasse 116, D-68305 Mannheim
www.roche.com

Roche
