# Supplementary material for: Ethiopia National Food and Nutrition Survey to inform the Ethiopian National Food and Nutrition Strategy: a study protocol
Source: BMJ Open. 2023 Apr 25;13(4):e067641. doi: 10.1136/bmjopen-2022-067641 (PMC10151871; doi:10.1136/bmjopen-2022-067641)
Supplement: Supplementary data [file bmjopen-2022-067641supp012.pdf]

Data collection tool

Household Listing Form

FNS/1

FNS EA no______

Serial no of structure	Address description of structure	Residence (Y/N)	Serial no House/HH in the structure	Name of HH head	Occupied (Y/N)	Serial no occupied EA	Remark(mark "X" for selected HH)

Woldeyohannes M, *et al. BMJ Open* 2023; 13:e067641. doi: 10.1136/bmjopen-2022-067641

Annex 2: Nutrition direct and indirect interventions/indicators assessment questionnaire

Nutrition direct and indirect Interventions Questionnaire

Module 1: Household identifier, characteristics and socio-demographic status

**Household identifier and characteristics**						
101	Region Code	|__||__|				
102	Woreda Code	|__||__|				
103	Kebele Code	|__||__|				
104	Gote Code	|__||__|				
105	Household Code	|__||__| |__||__||__||__||__||__|				GPS Coordinates
106	Unique Household Code	|__||__||__|	|__||__|	|__||__|	|__||__|	|__||__||__|
		Region Code	Woreda Code	Kebele Code	EA Code	Household Code
107	Residence	1 = Urban<br>2 = Rural				|__|
108	Do you own this house?	1 = Yes<br>0 = No				|__|
109	What is the main material of the walls?<br><br>**Observe**	1 = No walls<br>2 = Natural materials (cane, wood, mud, straw)<br>3 = Stone with mud<br>4 = Stone/bricks with cement<br>99 = Other				|__||__|
110	What is the main floor material?<br><br>**Observe**	1 = Natural floor (earth/sand/dung)<br>2 = Rudimentary floor (wood/palm/bamboo)<br>3=Finished floor (polished wood/ vinyl/ tiles/cement/carpet)<br>99 = Other				|__||__|
111	What is the main material of the roof?<br>**Observe**	1 = Thatch/grass or leaves<br>2 = Iron sheets or tiles<br>99 = Other				|__||__|
112	What type of fuel does your household mostly use for cooking?<br><br>**Do not read list**	1 = Dung<br>2 = Firewood/straw<br>3 = Charcoal<br>4 = Kerosene<br>5 = Gas (methane/biogas)<br>6 = Electricity<br>99 = Other				|__||__|
113	Is the house connected to electricity?	1 = Yes<br>0 = No				|__|
114	In total, how many of the following items are owned by residents of this household?<br><br>**Add the household total for each item**	A kerosene lamp/pressure lamp				|__|
115		Mobile phone				|__|
116		Cart				|__|
117		Bicycle				|__|
118		Motorcycle				|__|
119		Radio				|__|
120		Television				|__|

121		Car/tractor/Bajaj	|__|
122	Does this household own any livestock, herds, other farm animals, or poultry?	1 = Yes<br>0 = No **(Go to→131)**	
123	In total, how many of the following animals are owned by residents of this household?<br>**Add the household total for each item**	Milk cows, oxen or bulls?	|__|
124		Other cattle?	|__|
125		Horses, donkeys, or mules?	|__|
126		Camels	|__|
127		Goats?	|__|
128		Sheep?	|__|
129		Chickens or other poultry?	|__|
130		Beehives?	|__|
131	Does any member of this household own any agricultural land?	1 = Yes<br>0 = No	|__|
132	How often does anyone smoke inside your house?<br>*Would you say daily, weekly, monthly, less often than once a month, or never?*	1 = Daily<br>2 = Weekly<br>3 = Monthly<br>4 = Less once monthly<br>5 = Never	|__|
**Household head socio-demographic status**			
133	Age in years		|__||__|
134	Marital status	1. Single<br>2. Married<br>3. Divorced<br>4. Separated<br>5. Widowed	|__|
135	What is the highest level of school the head of the household completed?	1. None<br>2. Primary<br>3. Secondary<br>4. Technical/vocational certificate<br>5. Higher / university/ college<br>98. Don't know<br>99. Other (specify)______	|__||__|
136	What is the religion of the head of the HH?	1. Orthodox<br>2. Protestant<br>3. Catholic/ other Christian<br>4. Muslim<br>5. No religion<br>98. Don't know<br>99. Other religion (specify)__________	|__||__|
137	Ethnicity	Specify	

Woldeyohannes M, *et al. BMJ Open* 2023; 13:e067641. doi: 10.1136/bmjopen-2022-067641

Module 2: Child health

Now I would like to ask some questions about the health of your children born in the last 5 years. We will talk about each separately, starting with the youngest.			
201	Child's code		|__||__|
202	Mother's name	Mother's given name	
203	Mother's age		
204	Mother's education level	1=None<br>2=Primary<br>3=Secondary<br>4=Technical/vocational certificate<br>5=Higher / university/ college<br>98=Don't know<br>99. Other (specify)_________	
205	Mother's marital status	1=Single<br>2 =Married<br>3=Divorced<br>4 =Separated<br>5=Widowed	
206	Mother's religion	1=Orthodox<br>2=Protestant<br>3=Catholic/ other Christian<br>4=Muslim<br>5=No religion<br>98=Don't know<br>99=Other religion (specify)______	
207	Mother's ethnicity	Specify	
208	Child's name	Child's given name	
209	Child (NAME) sex	1 = Boy<br>2 = Girl	|__|
210	Child (NAME) age?	Age in months or age at the time of the child's death	|__||__|
211	In the last six months, was (NAME) given any vitamin A supplement?	1 = Yes<br>0 = No<br>98 = Don't know	|__||__|
212	When was the child (NAME) given the vitamin A supplement?	Specify	
213	In the last 12 months, was (NAME) given any iron tablet or syrup or supplement?	1 = Yes<br>0 = No<br>98 = Don't know	|__||__|
214	In the last 6 months, was (name) given any medicine for intestinal worms?	1 = Yes<br>0 = No<br>98 = Don't know	|__||__|
215	In the last 3 months, has any healthcare provider measured?	1 = Yes, 0 = No, 98 = Don't know	
		Weight	|__||__|
		Height/length	|__||__|
		MUAC	|__||__|
216	Has (name) had diarrhea in the last 2 weeks?	1 = Yes<br>0 = No **(Go to 224)**	|__||__|

		98 = Don't know	
217	Now I would like to know how much was the child given to drink during diarrhea, including breast milk. Was the child given less than usual to drink, about the same amount, or more than usual to drink?	1 = Much less<br>2 = Somewhat less<br>3 = About the same<br>4 = More<br>5 = Nothing to drink<br>98 = Don't know	|__|
218	When the child had diarrhea, was he/she given less than usual to eat, about the same amount, more than usual, or nothing to eat?	1 = Much less<br>2 = Somewhat less<br>3 = About the same<br>4 = More<br>5 = Nothing to drink<br>98 = Don't know	|__|
219	Did you seek advice or treatment for the diarrhea from any source?	1 = Yes<br>0 = No **(Go to 221)**	|__|
220	Where did you seek advice or treatment? Anywhere else?<br><br>*Probe to identify the type of source. If unable to determine if public, private, or NGO sector, record '21' and write the name Of the place(s).*	1= Government hospital<br>2= Government health center<br>3= Government health post<br>4 = Mobile clinic<br>5 = Community health worker/ fieldworker<br>6 = Other public sector (specify)<br>7 = Private hospital<br>8 = Private clinic<br>9 = Pharmacy<br>10 = Private doctor<br>11 = Mobile clinic<br>12 = Community health worker/fieldworker<br>13 = Other private medical sector (specify)<br>14 = NGO hospital<br>15 = NGO clinic<br>16 = Other NGO medical sector (specify)<br>17 = Shop<br>18 = Traditional practitioner<br>19 = Market<br>20 = Itinerant drug seller<br>99 = Other (specify)_______	|__||__|
	Was (name) given any of the following at any time since (name) started having diarrhea:	1 = Yes, 0 = No, 98 = Don't know	
221		Fluid from ORS packet	|__||__|
222		Zinc	|__||__|
223		Homemade fluid	|__||__|
224	Has (name) been ill with a fever at any time in the last 2 weeks?	1 = Yes<br>0 = No (**Go to 226**)<br>98 = Don't know	|__||__|
225	Where did you seek advice or treatment for	1= Government hospital	

	fever?<br>Anywhere else?<br><br>*Probe to identify the type of source.*<br>*If unable to determine if public, private, or NGO sector, record '21' and write the name Of the place(s).*	2= Government health center<br>3= Government health post<br>4 = Mobile clinic<br>5 = Community health worker/ fieldworker<br>6 = Other public sector (specify)<br>7 = Private hospital<br>8 = Private clinic<br>9 = Pharmacy<br>10 = Private doctor<br>11 = Mobile clinic<br>12 = Community health worker/fieldworker<br>13 = Other private medical sector (specify)<br>14 = NGO hospital<br>15 = NGO clinic<br>16 = Other NGO medical sector (specify)<br>17 = Shop<br>18 = Traditional practitioner<br>19 = Market<br>20 = Itinerant drug seller<br>99 = Other (specify)_____	|__||__|
226	Was Child (Name) ever breastfed?	1 = Yes<br>0 = No (**Go 228**)	|__|
227	How many months the child (NAME) was breastfed?		|__|
**Anthropometric and clinical nutrition assessment**			
228	Weight		|__||__|
229	Height/length		|__||__|
230	MUAC		|__||__|
231	Presence of bilateral oedema for children 6-59 months	1 = Yes<br>0 = No	|__|
232	Bitot spot	1 = Yes<br>0 = No	

Module 3: Infant and young child feeding practices

**For children 0-23 months**			
This module is to be administered to the mother/caregiver of children born 0-23 months before the survey living with respondents. Verify that the respondent you are speaking to is the mother/caregiver of the child.			
301	Was Child (Name) ever breastfed?	1 = Yes<br>0 = No (**Go to 304)**<br>98 = Don't know	|__||__|
302	Was Child (NAME) given the first milk (colostrum) after birth?	1 = Yes<br>0 = No<br>98 = Don't know	|__||__|
303	How long after birth did you first put (NAME) to the breast, even if your breast milk did not arrive?	1 = Immediately after birth, or within 1 hour<br>2 = Between 1 and 24 hours<br>3 = More than 24 hours after delivery<br>98 = Don't know	|__||__|
304	Child (NAME) alive now?	1 = Yes<br>0 = No (**Go to 401**)	|__||__|
305	Was *(NAME)* breastfed yesterday from sunrise until today sunrise?<br>NB: Breastfeeding could be by the mother herself or by wet mother.	1 = Yes<br>0 = No (**Go to 307**)<br>98 = Don't know	|__||__|
306	*Sometimes babies are fed breast milk in different ways, for example by spoon, cup or bottle. This can happen when the mother cannot always be with her baby. Sometimes babies are breastfed by another woman, or given breast milk from another woman by spoon, cup or bottle or some other way. This can happen if a mother cannot breastfeed her own baby.*<br><br>Did *(NAME)* consume breast milk in any of these ways yesterday from sunrise until today sunrise?	1 = Yes<br>0 = No<br>98 = Don't know	|__||__|
307	Now I would like to ask you about some medicines and vitamins that are sometimes given to infants.<br><br>Was *(NAME)* given any vitamin drops or other medicines as drops yesterday from sunrise until today sunrise?	1 = Yes<br>0 = No<br>98 = Don't know	|__||__|
308	Was *(NAME)* given Lemlem or ORS in the last two weeks?	1 = Yes<br>0 = No<br>98 = Don't know	|__||__|

309	In the last 6 months, did any healthcare provider or community health worker talk with you about how and what to feed your child?		1 = Yes<br>0 = No<br>98 = Don't know	|__||__|
310	Now, I would like to ask you about some liquids that *(NAME)* may have had yesterday from sunrise until today sunrise?<br><br>**If yes to Q310, read the list of liquids starting with 'plain water'.**		Did *(NAME)* have any *(item from list)*?<br>1 = Yes<br>0 = No **(Go to 321)**<br>98 = Don't know	|__||__|
311		Plain water	1 = Yes<br>0 = No<br>98 = Don't know	|__||__|
312		Infant formula such as S-26?	1 = Yes<br>0 = No **(Go to 314)**<br>98 = Don't know	|__||__|
313		How many times infant formula such as S-26?		|__||__|
314		Milk such as tinned, powdered, or fresh animal milk?	1 = Yes<br>0 = No **(Go to 316)**<br>98 = Don't know	|__||__|
315		How many times milk drink?		|__||__|
316		Yogurt drink?	1 = Yes<br>0 = No<br>98 = Don't know	|__||__|
317		Chocolate flavored drink?	1 = Yes<br>0 = No<br>98 = Don't know	|__||__|
318		Sodas, malt drinks or energy drinks?	1 = Yes<br>0 = No<br>98 = Don't know	|__||__|
319		Clear broth or clear soup?	1 = Yes<br>0 = No<br>98 = Don't know	|__||__|
320		Any other liquids?	1 = Yes<br>0 = No<br>98 = Don't know	|__||__|
Now I would like to ask you about foods that (NAME) had yesterday during the day or night. I am interested in foods your child ate whether at home or somewhere else. I will ask you about different types of foods, and I would like to know whether your child ate the food even if it was combined with other foods. Please do not answer 'yes' for any food or ingredient used in a small amount to add flavor to a dish.<br><br>OTHER FOODS: Please write down other foods in this box that respondent mentioned but are not in the list below<br><br>Yesterday during the day or at night, did (NAME) eat:				
321	Did the child ate any solid or semi-solid food yesterday?			Eaten?<br>1 = Yes

			0 = No **(Go to 342)**<br>98 = Don't know
322	Yogurt, other than yogurt drink?		|__|
323	How many times did child (NAME) eat yogurt?		|__|
324	Injera, bread, rice, noodles, pasta, macaroni, porridge, or other foods made from grains such as tef, oats, maize, barley?		|__|
325	Any commercially fortified baby food like Fafa, Hilina, Cerilak, Cerifam, Mother Choice?		
326	Pumpkin, carrots, squash, or sweet potatoes that are yellow or orange inside?		|__|
327	White potatoes, white yams, bulla, kocho, manioc, cassava or any other foods made from roots?		|__|
328	Any dark green leafy vegetables (kale, dark green lettuce, moringa …)?		|__|
329	Any other vegetable?		
330	Ripe mangoes, ripe papayas (insert other local vitamin a-rich fruits)?		|__|
331	Any other fruit?		|__|
332	Liver, kidney, heart, or other organ meats?		|__|
333	Any meat, such as beef, pork, lamb, goat, chicken?		|__|
334	Egg?		|__|
335	Fresh or dried fish, shellfish, or seafood?		|__|
336	Any foods made from beans, peas, lentils, nuts, or seeds?		|__|
337	Cheese or other food made from milk?		|__|
338	Any sugary foods such as chocolates, sweets, candies, pastries, cakes, or biscuits		|__|
339	Any savory junk foods, such as crisps/chips/salted biscuits/instant noodles?		|__|
340	Any other solid, semi-solid, or soft food?		|__|
341	How many times did *(NAME)* eat solid, semi-solid, or soft foods other than liquids yesterday during the day or at night?	Fill in the number of times.<br>98 = Don't know	|__||__|
342	Did *(NAME)* drink anything from a bottle with a nipple yesterday during the day or night?	1 = Yes<br>0 = No<br>98 = Don't know	|__||__|

Woldeyohannes M, *et al. BMJ Open* 2023; 13:e067641. doi: 10.1136/bmjopen-2022-067641

Module 4: KAP of mothers or caregivers on children's care and feeding

I am going to read you some knowledge questions about breastfeeding. Please tell me your answers on these questions.			
401	How long after birth should a baby start breastfeeding?	1 = Immediately, within 1 hour of delivery<br>2 = Some hours later but within 24 hours<br>3 = After 1 day<br>4 = After 2 days<br>5 = After >3 days<br>6 = Does not think a baby should be breastfed<br>98 = Don't know	|__||__|
402	How long should a baby receive nothing other than breast milk?	1 = From birth to six months<br>2 = Other<br>98 = Don't know	|__||__|
403	How often should a baby younger than six months be breastfed or fed with breast milk?	1 = On-demand, whenever the baby wants<br>2 = Other<br>98 = Don't know	|__||__|
404	How much should a child be fed when he/she is sick?	1 = Less frequent than usual<br>2 = Same as usual<br>3 = More than usual<br>98 = Don't know	|__||__|
405	How often should a child be fed when he/she is sick?	1 = Less frequently than usual<br>2 = Same as usual<br>3 = More frequently than usual<br>98 = Don't know	|__||__|
406	At what age should a baby first start to receive foods in addition to breast milk?	Months of age (Specify)<br>98= Don't know	|__||__|
407	At what age should children begin observing fasting days if that is their culture or religion?<br>(If <2 years, enter age in months.)	Years of age (Specify)<br>98=Don't know/remember	|__||__|
408	Have you ever heard of child stunting?	1 = Yes<br>0 = No<br>98 = Don't know	|__||__|
409	What age are children at the highest risk of becoming stunted?	Years of age (Specify)<br>Months of age (Specify)<br>98 = Don't know/remember	|__||__|
410	What are the consequences of stunting for young children?<br>Mark all that are mentioned by the respondent	1 = Higher risk of severe infectious diseases<br>2 = Poor educational performance<br>3 = Weaker immune system<br>4 = Low adult wages<br>5 = Lost productivity<br>6 = Excessive weight gain in later life	|__||__|

Woldeyohannes M, *et al. BMJ Open* 2023; 13:e067641. doi: 10.1136/bmjopen-2022-067641

		7 = Increased risk of nutrition-related chronic diseases in adult life<br>8 = Increased mortality rate<br>98= Don't know<br>99 = Other	
411	Poor diet during pregnancy and the first two years of child age can cause child stunting	1 = Agree<br>2 = Do not agree<br>98 = Don't know	|__||__|
I am going to read you some statements about breastfeeding and complementary feeding made by other mothers who live in a community like yours. Please tell me if you agree with these statements. Remember, there are no correct answers! I would like to know your opinion.			
412	The colostrum (the "first yellowish milk") is not good for the baby and should be discarded	1 = Strongly disagree<br>2 = Disagree<br>3 = Agree somewhat<br>4 = Agree<br>5 = Strongly agree<br>98 = Don't know	|__||__|
413	It is good to exclusively breastfeed give a baby only breast milk and no other foods or liquids for the first six months	1 = Strongly disagree<br>2 = Disagree<br>3 = Agree somewhat<br>4 = Agree<br>5 = Strongly agree<br>98 = Don't know	|__||__|
414	If a child is sick (for example has fever/diarrhea) breastfeeding must be continued	1 = Strongly disagree<br>2 = Disagree<br>3 = Agree somewhat<br>4 = Agree<br>5 = Strongly agree<br>98 = Don't know	|__||__|
415	A child should eat eggs, cow milk, or meat even on fasting days	1 = Strongly disagree<br>2 = Disagree<br>3 = Agree somewhat<br>4 = Agree<br>5 = Strongly agree<br>98 = Don't know	|__||__|
416	Eating a meal from different food groups is not necessary until children are old enough to go to school	1 = Strongly disagree<br>2 = Disagree<br>3 = Agree somewhat<br>4 = Agree<br>5 = Strongly agree<br>98 = Don't know	|__||__|
417	It is good to feed a two years child at least four times each day	1 = Strongly disagree<br>2 = Disagree<br>3 = Agree somewhat<br>4 = Agree<br>5 = Strongly agree<br>98 = Don't know	|__||__|
418	A mother should eat nutritious food	1 = Strongly disagree	|__||__|

	(four) times daily from the time of pregnancy	2 = Disagree<br>3 = Agree somewhat<br>4 = Agree<br>5 = Strongly agree<br>98 = Don't know	
419	A mother should take iron folic acid tablets during pregnancy	1 = Strongly disagree<br>2 = Disagree<br>3 = Agree somewhat<br>4 = Agree<br>5 = Strongly agree<br>98 = Don't know	|__||__|
420	A mother should take iodized salt during pregnancy	1 = Strongly disagree<br>2 = Disagree<br>3 = Agree somewhat<br>4 = Agree<br>5 = Strongly agree<br>98 = Don't know	|__||__|
421	A mother should take de-worming medicines during pregnancy	1 = Strongly disagree<br>2 = Disagree<br>3 = Agree somewhat<br>4 = Agree<br>5 = Strongly agree<br>98 = Don't know	|__||__|

Woldeyohannes M, *et al. BMJ Open* 2023; 13:e067641. doi: 10.1136/bmjopen-2022-067641

Module 5: Adolescent girls (10-19 Years)

Hint; This section is administered for Adolescent girls 10-19 years old. Provide a paper copy of both the informed consent and Assent Form to the respondent Read the consent (for mothers of adolescent girls) and Assent (adolescent girls) form			
501	Girl's code		|__||__|
502	Girl's name	Given name	
503	Girl's age		
504	Girl's education level	1=None<br>1=Primary<br>2=Secondary<br>3=Technical/vocational certificate<br>98=Don't know<br>99=Other (specify)	
505	Girl's marital status	1 = Single<br>2 = Married<br>3 = Divorced<br>4 = Separated<br>5 = Widowed	
506	Girl's religion	1=Orthodox<br>2=Protestant<br>3=Catholic/ other Christian<br>4=Muslim<br>5=No religion<br>98=Don't know<br>Other religion (specify)	
507	Girl's ethnicity	specify	
508	Are you currently a student	1 = Yes<br>0 = No	|__|
509	Were you given any iron/foliate tablets at school or out of school? (***show the tablet)***	1 = Yes<br>0 = No (**Go to 511)**	|__|
510	How many weeks per month have you taken the iron tablets?	Weeks per month (specify)<br>98 = don't know	|__||__|
511	Were you given any drug for intestinal worms at school or out of school in the last six months?	1 = Yes<br>0 = No	|__|
512	Have you received any nutrition counseling in the last six months?	1 = Yes<br>0 = No	|__|
513	Did you receive nutritional assessment services in health facilities when you went for any kind of health service?	1 = Yes<br>0 = No	|__|
514	Is there any food taboo for adolescent girls in your community?	1 = Yes<br>0 = No (**Go to 516**)	|__|
515	Mention types of food taboo?		
Anthropometry and clinical nutrition assessment			
516	Weight (in kg)		
517	Height (in CM)		
518	Waist circumference (in CM)		

519	Goiter	1 = Yes, 0 = No

Module 6: Reproductive age women (15-49 Years)

For currently pregnant women			
601	Woman's code		
602	Woman's name	Given name	
603	Woman's age		
604	Woman's education level	1=None<br>2=Primary<br>3=Secondary<br>4=Technical/vocational certificate<br>5=Higher / university/ college<br>98=Don't know<br>99=Other (specify)	
605	Woman's marital status	1 = Single<br>2 = Married<br>3 = Divorced<br>4 = Separated<br>5 = Widowed	
606	Woman's religion	1=Orthodox<br>2=Protestant<br>3=Catholic/ other Christian<br>4=Muslim<br>5=No religion<br>98=Don't know<br>99=Other religion (specify)	
607	Woman's ethnicity	Specify	
608	What was your age at first marriage	Year (specify)<br>96=Not applicable<br>98=Don't know	|__||__|
609	What was your age at your first pregnancy	Year (specify)<br>96=Not applicable<br>98=Don't know	|__||__|
610	Were you pregnant in the last 3 years?	1= Yes<br>0=No **(Go to 629)**	
611	Did you see anyone for antenatal care for the pregnancy?	1 = Yes<br>0 = No (**Go to 629**)	|__|
612	Whom did you see for antenatal care?<br>Probe to identify each type of person and record all	1=Health personnel<br>2 = Doctor<br>3 = Nurse<br>4 = Midwife<br>5= Health officer<br>6 = Health extension worker<br>7 = Other person<br>8 = Traditional birth Attendant<br>99 = Other (specify)	|__||__|
613	Where did you receive antenatal care for this pregnancy?<br>Anywhere else?	1= My home<br>2 = Her home<br>3 = Other home<br>4 = Health center	|__||__|

		5 = Government Hospital<br>6 = Private Clinic or Hospital<br>7 = Health post<br>8 = NGO clinic or hospital<br>99 = Other SPECIFY	
614	How many months pregnant were you when you first received antenatal care for this pregnancy?	Months (Specify)<br>98 = Don't know	|__||__|
615	How many times did you receive antenatal care during this pregnancy?	Number of times (specify)<br>98 = Don't know	|__||__|
616	As part of your antenatal care during this pregnancy, were any of the following done at least once:	1 = Yes 0 = No	
		Was your blood pressure measured?	|__|
		Did you give a urine sample?	|__|
		Did you give a blood sample?	|__|
		Did a health care provider talk with you about which foods to eat while pregnant?	|__|
		Did a health care provider weigh you?	|__|
		Did a health care provider talk with you about your weight?	|__|
		Did a health care provider talk with you about breastfeeding?	|__|
617	During this pregnancy, did you ever receive food or cash assistance from government, an NGO, religious institution or other group?	1 = Yes<br>0 = No (**Go to 619**)	|__|
618	What type of assistance did you receive?	1 = Cash only<br>2 = Food only<br>3 = Cash and food mix<br>99 = Other (specify)	|__||__|
619	During this pregnancy, were you given or did you buy any iron tablets or iron syrup?<br><br>***show tablets/syrup/multiple micronutrient supplement***	1 = Yes<br>0 = No **(Go to 622)**<br>98 = Don't Know **(Go to 622)**	|__||__|
620	Where did you get the iron tablet or syrup from?	1 = Govt. Health Facility<br>2 = Private Health Facility<br>3 = Mobile Clinic<br>4 = CHW<br>5 = [Mass Distribution Campaign – Add Local Name]<br>6 = Pharmacy<br>7 = Shop/Market<br>8 = School<br>98 = Don't Know<br>99= Other	|__||__|
621	During this pregnancy, for how many days did you take the iron tablets?<br>***If answer is not numeric, probe for***	Number of days (specify)<br>98 = Don't Know	|__||__|

	approximate number of days.		
622	During this pregnancy, did you take any drug for intestinal worms?	1 = Yes<br>0 = No<br>98 = Don't Know	|__||__|
623	During this pregnancy, did any health care provider talk with you about breastfeeding?	1 = Yes<br>0 = No<br>98 = Don't Know	|__||__|
624	During this pregnancy, did you practice fasting?	1 = Yes<br>0 = No<br>98 = Don't Know	|__||__|
625	At your last ANC visit, did the health provider weigh you?	1 = Yes<br>0 = No<br>98 = Don't Know	|__||__|
626	During this pregnancy has your health provider given you information about your weight gain?	1 = Yes<br>0 = No (**Go to 629**)<br>98 = Don't Know	|__||__|
627	During your pregnancy have you been thin for your height?	1 = Yes<br>0 = No<br>98 = Don't Know	|__||__|
628	Did you received treatment for malnutrition?	1 = Yes<br>0 = No	|__||__|
**Anthropometry**			
629	Weight (in kg)		
630	Height (in CM)		
631	MUAC		
632	Waist circumference (in cm)		
633	Goiter	1 = Yes<br>0 = No	

Woldeyohannes M, *et al. BMJ Open* 2023; 13:e067641. doi: 10.1136/bmjopen-2022-067641

Module 7: Women Dietary Diversity

"Now I'd like to ask you about foods and drinks that you ate or drank yesterday during the day or night, whether you ate it at home or anywhere else.		
Yesterday, during the day or at night did you eat or drink:		
S.N	Question	Response<br>1 = Yes<br>0 = No
701	Woman's code	
702	Woman's name	Given name
703	Woman's age	
704	Woman's education level	0=None<br>1=Primary<br>2=Secondary<br>3=Technical/vocational certificate<br>4=Higher / university/ college<br>98=Don't know<br>99=Other (specify)
705	Woman's marital status	1= Single<br>2= Married<br>3= Divorced<br>4= Separated<br>5= Widowed
706	Woman's religion	1=Orthodox<br>2=Protestant<br>3=Catholic/other Christian<br>4=Muslim<br>5=No religion<br>98=Don't know<br>99=Other religion (specify)
707	Woman's ethnicity	Specify
708	Any vegetables or roots that are orange-colored inside, like: pumpkin, carrots, squash, or sweet potatoes that are yellow or orange inside	|__|
709	Any white roots and tubers or plantains, such as: white potatoes, white yams, manioc/cassava/yucca, cocoyam, taro or any other foods made from white-fleshed roots or tubers, or plantains	|__|
710	Any dark green leafy vegetables, such as: [list examples of any medium-to-dark green leafy vegetables including wild/foraged leaves]	|__|
711	Any fruits that are dark yellow or orange inside, like: ripe mango, ripe papaya	|__|
712	Any other fruits	|__|
713	Any other vegetables	|__|
714	Any meat made from animal organs, such as: liver, kidney, heart or other organ meats or blood-based foods, including from wild game	|__|
715	Any other types of meat or poultry, like: beef, pork, lamb, goat, rabbit, wild game meat, chicken, duck, other birds	|__|

716	Any eggs	|__|
717	Any fish or seafood, whether fresh or dried	|__|
718	Any beans or peas, such as: mature beans or peas (fresh or dried seed), lentils, or bean/pea products, including hummus, tofu and tempeh	|__|
719	Any nuts or seeds, like tree nut, groundnut/peanut, or certain seeds or nut/seed “butters” or pastes	|__|
720	Any milk or milk products, such as: milk, cheese, yoghurt or other milk products, but not including butter, ice cream, cream or sour cream	|__|
721	Any oils and fats	|__|
722	Any savory and fried snacks, such as: crisps and chips, fried dough, other fried snacks	|__|
723	Any sweets, such as: sugary foods, such as chocolates, candies, cookies/sweet biscuits and cakes, sweet pastries or ice cream	|__|
724	Any sugar-sweetened beverages, like: sweetened fruit juices and “juice drinks”, soft drinks/fizzy drinks, chocolate drinks, malt drinks, yoghurt drinks, sweet tea or coffee with sugar	|__|
725	Any condiments and seasonings, such as: ingredients used in small quantities for flavour, such as chilies, spices, herbs, fish powder, tomato paste, flavor cubes or seeds	|__|
726	Any other beverages and foods like tela, tej, bordea, arkea, cheka, tselo	|__|
727	Did you eat anything (meal or snack) OUTSIDE of the home yesterday?	|__|
728	Did you fast yesterday during the day or night?	|__|

Woldeyohannes M, *et al. BMJ Open* 2023; 13:e067641. doi: 10.1136/bmjopen-2022-067641

Module 8: SELF-REPORTING QUESTIONNAIRE (SRQ-20)

No	Question	Answer<br>1 = Yes<br>0 = No	
801	Do you often have headaches?		|__|
802	Is your appetite poor?		|__|
803	Do you sleep badly?		|__|
804	Are you easily frightened?		|__|
805	Do your hands shake?		|__|
806	Do you feel nervous, tense or worried?		|__|
807	Is your digestion poor?		|__|
808	Do you have trouble thinking clearly?		|__|
809	Do you feel unhappy?		|__|
810	Do you cry more than usual?		|__|
811	Do you find it difficult to enjoy your daily activities?		|__|
812	Do you find it difficult to make decisions?		|__|
813	Is your daily work suffering?		|__|
814	Are you unable to play a useful part in life?		|__|
815	Have you lost interest in things?		|__|
816	Do you feel that you are a worthless person?		|__|
817	Has the thought of ending your life been on your mind?		|__|
818	Do you feel tired all the time?		|__|
819	Do you have uncomfortable feelings in your stomach?		|__|
820	Are you easily tired?		|__|

Woldeyohannes M, *et al. BMJ Open* 2023; 13:e067641. doi: 10.1136/bmjopen-2022-067641

Module 9: Women empowerment

901	Identify the most senior mother of the mothers who have a selected child. She is the mother who should respond to the rest of this interview from this point.		|__||__|
902	In the past major growing season (Meher) and minor growing (Belg) season, not including the current season, did you work on the family farm?	1 = Yes<br>0 = No → **Go to 913)**	|__|
903	What sort of work you did on the family farm?	1 = Home (kitchen) gardening	|__|
904		2 = Fieldwork	|__|
905		3 = Cash crop farming	|__|
906		4 = Dairy processing	|__|
907		5 = Poultry rearing	
908		6 = Raising livestock	|__|
909		7 = Fishpond/ aquaculture	|__|
910		99 = Other (specify)	|__|
911	From the work that you did on the farm did your household earn any money?	1 = Yes<br>0 = No<br>98 = Don't know	|__|
912	Who usually decides how the money you earn will be used?<br><br>**READ THE LIST.**	1 = Self<br>2 = Husband<br>3 = Self and husband jointly<br>4 = Someone else	|__|
913	Who usually makes decisions about major household purchases/sell such as cattle or livestock?<br><br>**READ THE LIST.**	1 = Self<br>2 = Husband<br>3 = Self and husband jointly<br>4 = Someone else	|__|
914	Who usually makes decisions about minor household purchases/sell such as spices/oils, soap, utensils, or daily household needs?<br><br>**READ THE LIST.**	1 = Self<br>2 = Husband<br>3 = Self and husband jointly<br>4 = Someone else	|__|
915	Who usually makes decisions about health care for your children?<br><br>**READ THE LIST.**	1 = Self<br>2 = Husband<br>3 = Self and husband jointly<br>4 = Someone else	|__|
916	Do you have husband?	1 = Yes<br>0 = No (**Go to 918**)	|__|
917	Who usually decides how the money your husband earns will be used?<br><br>**READ THE LIST.**	1 = Self<br>2 = Husband<br>3 = Self and husband jointly<br>4 = Someone else	

918	Do you have children?	1 = Yes<br>0 = No (**Go to 923)**	
919	Does your husband help you care for the children?	1 = Yes<br>0 = No (**Go to 923)**	|__|
920	Does he help care for the children almost every day, at least once a week, or rarely?	1 = Every day<br>2 = At least once a week<br>3 = Rarely	|__|
921	Does your husband help you with household chores like cooking, cleaning the house, fetching water, collecting firewood or other domestic work?	1 = Yes<br>0 = No (**Go to 923)**	|__|
922	Does he help almost every day, at least once a week, or rarely?	1 = Every day<br>2 = At least once a week<br>3 = Rarely	|__|

923	At any time during the previous major seasons (Meher) and minor season (Belg) not including the current season,<br><br>Did you:	Use improved seed varieties for any of your crops?	1 = Yes<br>0 = No<br>98 = Don't know	|__||__|
924		Keep improved varieties of livestock?	1 = Yes<br>0 = No<br>98 = Don't know	|__||__|
925		Use animal manure to improve you crops yield?	1 = Yes<br>0 = No<br>98 = Don't know	|__||__|
926		Use any other source of fertilizer on your crops?	1 = Yes<br>0 = No<br>98 = Don't know	|__||__|
927		Irrigate your crops?	1 = Yes<br>0 = No<br>98 = Don't know	|__||__|
928		Rotate your crops from one field to another when planting?	1 = Yes<br>0 = No<br>98 = Don't know	|__||__|
929		Harvest water during the rains?	1 = Yes<br>0 = No<br>98 = Don't know	|__||__|
930		Practice intercropping?	1 = Yes<br>0 = No<br>98 = Don't know	|__||__|
931	Have you ever taken any steps to reduce soil erosion on your farm?		1 = Yes<br>0 = No (**Go to 936)**<br>98 = Don't know (**Go to 936)**	|__||__|
	What steps did you take to reduce soil erosion?		For each mentioned: 1=Yes 0=No	
932			Plant trees or shrubs	|__|
933			Terracing	|__|

934		Use drainage system	|__|
935		Other	|__|
936	Have you received any inputs for your farm from a social/government program?	1 = Yes<br>0 = No (**Go to 1001)**	|__|
937	What farm inputs have you received?	For each mentioned: 1=Yes 0=No	
938		Seeds	|__|
939		Improved seeds	|__|
940		Livestock or poultry	|__|
941		Improved varieties of livestock/ poultry	|__|
942		Aquaculture (fish)	|__|
943		Fertilizer	|__|
944		Other	|__|

Woldeyohannes M, *et al. BMJ Open* 2023; 13:e067641. doi: 10.1136/bmjopen-2022-067641

Module 10: WASH

No	Question	choices	
1001	What is the main source of drinking water for the household?<br><br>**Do not read list**	1 = Piped into dwelling<br>2 = Piped to yard/plot<br>3 = Piped to neighbour<br>4 = Public tap/standpipe<br>5 = Tube well or borehole<br>6 = Protected well<br>7 = Unprotected well<br>8 = Protected spring<br>9 = Unprotected spring<br>10 = Rainwater<br>11 = Tanker truck<br>12 = Cart with small tank<br>13 = Surface water (river/dam/ Lake/pond/stream/canal/ irrigation channel)<br>14 = Bottled water<br>99 = Other	|__||__|
1002	Do you do anything to your household water to make it safer to drink?	1 = Yes<br>0 = No (**Go to 1004)**<br>98 = Don't know (**Go to 1004)**	|__||__|
1003	What is the main thing you do to make the water safer?	1 = Boil<br>2 = Add bleach/chlorine<br>3 = Strain through a cloth<br>4 = Use water filter (ceramic/ Sand/composite/etc)<br>5 = Solar disinfection<br>6 = Let it stand and settle<br>7 = Other(specify)<br>98 = Don't know	|__||__|
1004	What kind of toilet facility do members of your household usually use? If not possible to determine, ask for Permission to observe the facility.	1 = Flush to piped sewer system<br>2 = Flush to septic tank<br>3 = Flush to pit latrine<br>4 = Flush to somewhere else<br>5 = Flush, don't know where<br>6 = Ventilated improved pit latrine<br>7 = Pit latrine with slab<br>8 = Pit latrine without slab/open pit<br>9 = Composting toilet<br>10 = Bucket toilet<br>11 = Hanging toilet/hanging latrine<br>12 = No facility/bush/field<br>99 = Other (specify)________	|__||__|
1005	How does your HH primarily dispose of HH waste?	1 = Collected by municipality<br>2 = Buried<br>3 = Collected by private establishment<br>4 = Dumped in street/open space	|__||__|

		5 = Disposed in the compound<br>6 = Dumped in river<br>7 = Burned<br>98 = Other	
1006	Do you have separate cooking room?	1 = Yes<br>2 = No **(Go to 1008)**	
1007	Does the stove or cooking room have a chimney?	1 = yes<br>2 = No	|__||__|
1008	What type of fuel or energy source is used in this cook stove?	1 = Alcohol/ethanol<br>2 = Gasoline/diesel<br>3 = Kerosene/paraffin<br>4 = Coal/lignite<br>5 = Charcoal<br>6 = Wood<br>7 = Straw/shrubs/grass<br>8 = Agricultural crop<br>9 = Animal dung/waste<br>10 = Processed biomass (pellets) or woodchips<br>11 = Garbage/plastic<br>12 = Sawdust<br>96 = Other (specify)	|__||__|
1009	Do you have a confined space (beret/gata) to keep livestock?	1 = Yes<br>0 = No<br>96 = Not applicable	|__|
1010	Do you keep poultry in cages/confined systems (kote)?	1 = Yes<br>0 = No<br>96 = Not applicable	|__|
1011	What do you think are the activities before which you should wash your hands with soap?<br><br>**DO NOT PROMPT.**	For each mentioned: 1**=Yes 0=No**	
1012		Before preparing food	|__|
1013		Before touching or eating food	|__|
1014		Before feeding a child or other person	|__|
1015		Praying	|__|
1016		Don't know	|__|
1017	What do you think are the activities after which you should wash your hands with soap?<br><br>**DO NOT PROMPT.**	For each mentioned: **1=Yes 0=No**	
1018		After defecation or urinating	|__|
1019		After handling animals and their waste	|__|
1020		After housework or fieldwork	|__|
1021		After touching pets or handling animals and their waste	|__|
1022		After blowing nose or coughing	|__|
1023		After cleaning a child's bottom	|__|
1024		None	|__|
1025	What do you think are the reasons to keep poultry and livestock in a confined space? For each mentioned: **1=Yes 0=No**	To keep out of house |__|	
1026		To keep away from water source	|__|
1027		To reduce infectious disease	|__|

1028	**DO NOT PROMPT.**	To protect livestock/poultry	**|__|**
1029		Other	

Module 11: Food fortification (household coverage of fortifiable foods)

SN	Question	Response	
1101	Does your household use cooking oil to prepare foods or add to foods at home?	1 = Yes<br>0 = No →**Go to 1103**	|__|
1102	The last time your household get cooking oil, where did you get it from?	1=Purchased from market/shop/kiosk/wholesaler/street vendor<br>2= Homemade or obtained from local farm or local small factory/processor….<br><br>3= Received from food aid/social protection program<br>4 = Other (specify): ____________<br><br>98= Don't know/remember	|__||__|
1103	Does your household prepare foods using wheat flour at home, such as bread, kita, injera?	1 = Yes<br>0 = No →**Go to 1105**	|__|
1104	The last time your household get wheat flour, where did you get it from?	1=Purchased from market/shop/kiosk/wholesaler/street vendor<br>2= Homemade or obtained from local farm or local small factory/processor….<br><br>3= Received from food aid/social protection program<br>4 = Other (specify): ____________<br><br>98= Don't know/remember	|__||__|
1105	I would like to check whether the salt used in your household is iodized. May I have a sample of the salt used to cook meals in your household? Test salt for iodine	1=Iodine present<br>2= No iodine<br>3= Household uses salt but there is no salt in household<br>4= Household does not use salt<br>5= Salt not tested, specify reason________	|__|
1106	The last time your household get salt, where did you get it from?	1=Purchased from market/shop/kiosk/wholesaler/street vendor<br>2= Homemade or obtained from local farm or local small factory/processor….<br><br>3= Received from food aid/social protection program<br>98= Don't know/remember	|__||__|

		99 = Other (specify): _____________	

Module 12 – Agriculture practices

**About the household**			
1201	Does any member of the household own any agricultural land (purchased or own?	1 = Yes<br>0 = No (**Go to 1212)**	|__|
1202	How many hectares of agricultural land do members of this household own?<br><br>*Note: Convert local land measurement unit into hector after discussing with agriculture focal person/AEW.*	Enter total number of hectares (If less than 1, Enter in decimals (example 0.5)<br>Enter 9999 if hectares are not known	|__|__|__|__|
1203	In the past major growing season (Meher) and minor growing (Belg) season, not including the current season, did you work on the family farm?	1 = Yes<br>0 = No (**Go to 1211)**	|__|
	What sort of work did you do on the family farm?<br><br>READ THE LIST	1 = Yes 0 = No	
		1 = Home (kitchen) gardening	|__|
1204		2 = Fieldwork	|__|
1205		3 = Cash crop farming	|__|
1206		4 = Producing dairy	|__|
1207		5 = Rearing poultry	|__|
1208		6 = Raising livestock	|__|
1209		7 = Fishpond/ aquaculture	|__|
1210		99 = Other …………………..	|__|
1211	Does this household own any livestock, herds, other farm animals, or poultry?	1 = Yes<br>0 = No → **Go to 1221**	|__|
	How many of the following animals do this household own?	For each: Enter number. **If none, enter 0**	
1212		Chickens	|__|__|__|
1213		Goats	|__|__|__|
1214		Sheep	|__|__|__|
1215		Donkeys	|__|__|__|
1216		Horses	|__|__|__|
1217		Mules	|__|__|__|
1218		Camels	|__|__|__|
1219		Milk cows	|__|__|__|
1220		Oxen	|__|__|__|

Woldeyohannes M, *et al. BMJ Open* 2023; 13:e067641. doi: 10.1136/bmjopen-2022-067641

In the past 2 growing seasons (Meher and Belg), not including the current season, please describe all the crops (cereals, legumes, vegetables, fruits, seeds, and other crops) grown on your household farm.

Please also describe all animal source foods (meat, eggs, milk, dairy, fish, other) that you have produced on your household farm in same period.

Write down all crops and animals mentioned by the respondent. When the respondent has finished, probe for crops and animal source foods not mentioned. Then ask about production/yields in the relevant units. Ask the respondent to estimate the amount of the total production that went to sales, food consumption, and storage/losses/other uses.

	Group	Crop	Did HH cultivate crop? 1 = yes 0 = No	Season 1=Meher 2=Belg 3=Both	Amount	During the previous Major seasons (Meher) and Minor season (Belg) not including the current season		
						Sold	Consumed	Storage, losses, animal feed or other uses
1221	Staples	Maize	|__|		|__|	|__||__|	|__||__|	|__||__|
1222		Teff	|__|		|__|	|__||__|	|__||__|	|__||__|
1223		Wheat	|__|		|__|	|__||__|	|__||__|	|__||__|
1224		Barley	|__|		|__|	|__||__|	|__||__|	|__||__|
1225		Sorghum	|__|		|__|	|__||__|	|__||__|	|__||__|
1226		Millet	|__|		|__|	|__||__|	|__||__|	|__||__|
1227		Rice	|__|		|__|	|__||__|	|__||__|	|__||__|
1228		Emmer wheat (oaths)	|__|		|__|	|__||__|	|__||__|	|__||__|
1229		Other cereals	|__|		|__|	|__||__|	|__||__|	|__||__|
1230	Pulses (legumes)	Bean	|__|		|__|	|__||__|	|__||__|	|__||__|
1231		Haricot bean	|__|		|__|	|__||__|	|__||__|	|__||__|
1232		Lentil (Miser)	|__|		|__|	|__||__|	|__||__|	|__||__|
1233		Grass pea (guaya)	|__|		|__|	|__||__|	|__||__|	|__||__|
1234		Chickpea	|__|		|__|	|__||__|	|__||__|	|__||__|
1235		Field pea (Ater)	|__|		|__|	|__||__|	|__||__|	|__||__|
1236		Soya bean	|__|		|__|	|__||__|	|__||__|	|__||__|

<table>
<tr><td>1237</td><td></td><td>Other legumes</td><td>|__|</td><td></td><td>|__|</td><td>|__||__|</td><td>|__||__|</td><td>|__||__|</td></tr>
<tr><td>1238</td><td rowspan="8">Oil Crops</td><td>Niger seed (Nug)</td><td>|__|</td><td></td><td>|__|</td><td>|__||__|</td><td>|__||__|</td><td>|__||__|</td></tr>
<tr><td>1239</td><td>Sunflower</td><td>|__|</td><td></td><td>|__|</td><td>|__||__|</td><td>|__||__|</td><td>|__||__|</td></tr>
<tr><td>1240</td><td>Sesame</td><td>|__|</td><td></td><td>|__|</td><td>|__||__|</td><td>|__||__|</td><td>|__||__|</td></tr>
<tr><td>1241</td><td>Linseed</td><td>|__|</td><td></td><td>|__|</td><td>|__||__|</td><td>|__||__|</td><td>|__||__|</td></tr>
<tr><td>1242</td><td>Rapeseed (Gomenzer)</td><td>|__|</td><td></td><td>|__|</td><td>|__||__|</td><td>|__||__|</td><td>|__||__|</td></tr>
<tr><td>1243</td><td>Lupine (Gibto)</td><td>|__|</td><td></td><td>|__|</td><td>|__||__|</td><td>|__||__|</td><td>|__||__|</td></tr>
<tr><td>1244</td><td>Nuts</td><td></td><td></td><td></td><td>|__||__|</td><td>|__||__|</td><td>|__||__|</td></tr>
<tr><td>1245</td><td>Other oil crops</td><td>|__|</td><td></td><td>|__|</td><td>|__||__|</td><td>|__||__|</td><td>|__||__|</td></tr>
<tr><td>1246</td><td rowspan="15">Root crops/ tubers/ vegetables</td><td>Cassava</td><td>|__|</td><td></td><td>|__|</td><td>|__||__|</td><td>|__||__|</td><td>|__||__|</td></tr>
<tr><td>1247</td><td>Enset</td><td>|__|</td><td></td><td>|__|</td><td>|__||__|</td><td>|__||__|</td><td>|__||__|</td></tr>
<tr><td>1248</td><td>Irish potato</td><td>|__|</td><td></td><td>|__|</td><td>|__||__|</td><td>|__||__|</td><td>|__||__|</td></tr>
<tr><td>1249</td><td>Sweet potato</td><td>|__|</td><td></td><td>|__|</td><td>|__||__|</td><td>|__||__|</td><td>|__||__|</td></tr>
<tr><td>1250</td><td>Sweet potato - orange flesh</td><td>|__|</td><td></td><td>|__|</td><td>|__||__|</td><td>|__||__|</td><td>|__||__|</td></tr>
<tr><td>1251</td><td>Onion</td><td>|__|</td><td></td><td>|__|</td><td>|__||__|</td><td>|__||__|</td><td>|__||__|</td></tr>
<tr><td>1252</td><td>Pepper</td><td>|__|</td><td></td><td>|__|</td><td>|__||__|</td><td>|__||__|</td><td>|__||__|</td></tr>
<tr><td>1253</td><td>Tomato</td><td>|__|</td><td></td><td>|__|</td><td>|__||__|</td><td>|__||__|</td><td>|__||__|</td></tr>
<tr><td>1254</td><td>Cabbage</td><td>|__|</td><td></td><td>|__|</td><td>|__||__|</td><td>|__||__|</td><td>|__||__|</td></tr>
<tr><td>1255</td><td>Other light green leafy vegetables</td><td>|__|</td><td></td><td>|__|</td><td>|__||__|</td><td>|__||__|</td><td>|__||__|</td></tr>
<tr><td>1256</td><td>Kale</td><td>|__|</td><td></td><td>|__|</td><td>|__||__|</td><td>|__||__|</td><td>|__||__|</td></tr>
<tr><td>1257</td><td>Other dark green leafy vegetables</td><td>|__|</td><td></td><td>|__|</td><td>|__||__|</td><td>|__||__|</td><td>|__||__|</td></tr>
<tr><td>1258</td><td>Carrot</td><td>|__|</td><td></td><td>|__|</td><td>|__||__|</td><td>|__||__|</td><td>|__||__|</td></tr>
<tr><td>1259</td><td>Other roots or tubers</td><td>|__|</td><td></td><td>|__|</td><td>|__||__|</td><td>|__||__|</td><td>|__||__|</td></tr>
<tr><td>1260</td><td>Other vegetables</td><td>|__|</td><td></td><td>|__|</td><td>|__||__|</td><td>|__||__|</td><td>|__||__|</td></tr>
<tr><td>1261</td><td rowspan="6">Perennial crops/ fruits</td><td>Coffee</td><td>|__|</td><td></td><td>|__|</td><td>|__||__|</td><td>|__||__|</td><td>|__||__|</td></tr>
<tr><td>1262</td><td>Chat (khat)</td><td>|__|</td><td></td><td>|__|</td><td>|__||__|</td><td>|__||__|</td><td>|__||__|</td></tr>
<tr><td>1263</td><td>Banana</td><td>|__|</td><td></td><td>|__|</td><td>|__||__|</td><td>|__||__|</td><td>|__||__|</td></tr>
<tr><td>1264</td><td>Orange</td><td>|__|</td><td></td><td>|__|</td><td>|__||__|</td><td>|__||__|</td><td>|__||__|</td></tr>
<tr><td>1265</td><td>Mango</td><td>|__|</td><td></td><td>|__|</td><td>|__||__|</td><td>|__||__|</td><td>|__||__|</td></tr>
<tr><td>1266</td><td>Hop</td><td>|__|</td><td></td><td>|__|</td><td>|__||__|</td><td>|__||__|</td><td>|__||__|</td></tr>
</table>

Woldeyohannes M, *et al. BMJ Open* 2023; 13:e067641. doi: 10.1136/bmjopen-2022-067641

		(Gesho)						
1267		Avocado	|__|		|__|	|__||__|	|__||__|	|__||__|
1268		Lemon	|__|		|__|	|__||__|	|__||__|	|__||__|
1269		Papaya	|__|		|__|	|__||__|	|__||__|	|__||__|
1270		Guava	|__|		|__|	|__||__|	|__||__|	|__||__|
1271		Water Melon	|__|		|__|	|__||__|	|__||__|	|__||__|
1272		Tirngo fruit	|__|		|__|	|__||__|	|__||__|	|__||__|
1273		Other perennial crops	|__|		|__|	|__||__|	|__||__|	|__||__|
1274		Other fruits	|__|		|__|	|__||__|	|__||__|	|__||__|

Woldeyohannes M, *et al. BMJ Open* 2023; 13:e067641. doi: 10.1136/bmjopen-2022-067641

	Group	Animal source food (unit)	Does HH produce? **1=yes 0=no** *(If no, skip to the next item)*	**Amount**	**During the previous Major seasons (Meher) and Minor season (Belg) not including the current season**			
					Season 1=Meher 2=Belg 3=Both	**How much?**		
						Sold	Consumed	Storage, losses, animal feed or others
1275	All	Chicken eggs		|__|	|__|	|__|__|	|__|__|	|__|__|
1276		Chicken meat		|__|	|__|	|__|__|	|__|__|	|__|__|
1277		Goat milk		|__|	|__|	|__|__|	|__|__|	|__|__|
1278		Goat meat		|__|	|__|	|__|__|	|__|__|	|__|__|
1279		Camel milk		|__|	|__|	|__|__|	|__|__|	/__/__/
1280		Sheep meat		|__|	|__|	|__|__|	|__|__|	/__/__/
1281		Cow milk		|__|	|__|	|__|__|	|__|__|	/__/__/
1282		Cow other dairy		|__|	|__|	|__|__|	|__|__|	/__/__/
1283		Beef		|__|	|__|	|__|__|	|__|__|	/__/__/
1284		Other meat (e.g. camel, horse)		|__|	|__|	|__|__|	|__|__|	/__/__/
1285		Farmed fish		|__|	|__|	|__|__|	|__|__|	/__/__/

Woldeyohannes M, *et al. BMJ Open* 2023; 13:e067641. doi: 10.1136/bmjopen-2022-067641

Module 13: Household food insecurity

Now I would like to ask you some questions about food. During the last 12 MONTHS, was there a time when:			
SN	Questions	Answers	
1301	You or others in your household worried about not having enough food to eat because of a lack of money or other resources?	1=Yes<br>0= No<br>98=Don't know<br>97=Refused	|__||__|
1302	Still thinking about the last 12 MONTHS, was there a time when you or others in your household were unable to eat healthy and nutritious food because of a lack of money or other resources?	1=Yes<br>0= No<br>98=Don't know<br>97=Refused	|__||__|
1303	Was there a time when you or others in your household ate only a few kinds of foods because of a lack of money or other resources?	1=Yes<br>0= No<br>98=Don't know<br>97=Refused	|__||__|
1304	Was there a time when you or others in your household had to skip a<br>meal because there was not enough money or other resources to get<br>food?	1=Yes<br>0= No<br>98=Don't know<br>97=Refused	|__||__|
1305	Still thinking about the last 12 MONTHS, was there a time when you or others in your household ate less than you thought you should because of a lack of money or other resources?	1=Yes<br>0= No<br>98=Don't know<br>97=Refused	|__||__|
1306	Was there a time when your household ran out of food because of a lack of money or other resources?	1=Yes<br>0= No<br>98=Don't know<br>97=Refused	|__||__|
1307	Was there a time when you or others in your household were hungry but did not eat because there was not enough money or other resources for food?	1=Yes<br>0= No<br>98=Don't know<br>97=Refused	|__||__|
1308	Was there a time when you or others in your household went without eating for a whole day because of a lack of money or other resources?	1=Yes<br>0= No<br>98=Don't know<br>97=Refused	|__||__|

Module 14: Employment and social protection

S.N.	Questions	Response	
1401	Since last year, what has been the main livelihood or income source of the HH?<br><br>**(DO NOT READ LIST. PROBE FOR ONE RESPONSE)**	1 = Sale of self-produced<br>2 = horticulture crops Sale of self-produced field crops<br>3 = Own business (including commerce, livestock rearing)<br>4 = Wage employment<br>5 = Remittances<br>6 = Property income<br>7 = Government transfers/NGO support<br>8 = Pension<br>99 = Other	|__||__|
1402	Since last year, have there been other livelihood or income sources for the HH?<br><br>**(DO NOT READ LIST. PROBE FOR ALL RESPONSES)**	1 = Sale of self-produced<br>2 = horticulture crops Sale of self-produced field crops<br>3 = Own business (including commerce, livestock rearing)<br>4 = Wage employment<br>5 = Remittances<br>6 = Property income<br>7 = Government transfers/NGO support<br>8 = Pension<br>99 = Other	|__||__|
1403	Since last year, did anyone in your HH receive any kind of food or cash assistance from the government, NGO, or other agencies? *Clarify: This is not formal employment or pension. However, it may or may not be conditional on work.*	1 = Yes<br>0 = No<br>98 = Don't know	|__||__|
1404	Since last year, which members of this HH were targeted to receive this support?	1 = All HH members<br>2 = Specific HH members<br>98 = Don't know	|__||__|
1405	Which specific HH members received food or cash assistance?<br>*Clarify: This includes children whose parents receive cash on their behalf.*	Link this back to the HH roster and have interviewer select names.<br>98 = Don't know	|__||__|
1406	Which of these categories apply to the persons who received food or cash assistance?<br><br>(READ RESPONSES ALOUD. SELECT ALL THAT APPLY)	1 = Pregnant women<br>2 = Lactating women<br>3 = Children under 5 years<br>4 = Elderly<br>5 = Disabled person<br>6 = None of the above	|__||__|
1407	Since last year, which food or social assistance program did members of the HH receive support from? (DO NOT READ LIST ALOUD. PROBE FOR ALL RESPONSES)	1 = PSNP<br>2 = Community Care Coalition<br>3 = Other assistance program<br>98 = Don't know	|__||__|
1408	Since last year, what was the form of assistance that members of your HH received form these programs: food, cash or both food and cash?	1 = Cash only transfer<br>2 = Food only transfer<br>3 = Cash and food mix<br>99 = Other<br>98 = Don't know	|__||__|

1409	Is this HH currently receiving food or cash?	1 = Yes<br>0 = No<br>98 = Don't know	|__||__|
1410	Since last year, how has your HH used the food received? (READ RESPONSES ALOUD. SELECT ALL THAT APPLY)	1 = HH consumption<br>2 = Sold food for cash<br>3 = Other activities<br>98 = Don't know	

Woldeyohannes M, et al. BMJ Open 2023; 13:e067641. doi: 10.1136/bmjopen-2022-067641

1501.	How do you preserve soil fertility?<br><br>***[Multiple answer is allowed****! Do not read the choices. Listen and mark the one they mention*]	1 = Fertilization (Chemicals, animal manure, green manure etc)
		2 = Crop rotation (Cultivation of a series of dissimilar types of crops in the same area in sequential seasons)
		3=Intercropping (Cultivation of two or more dissimilar types of crops in the same area in the same season)
		4=Tillage
		99=Other (specify): …………………………………………………………………………………
1502.	Do you apply fertilizers?	1= Yes
		0= No (Go to 1506)
1503.	Which kinds of fertilizers do you use more often?	1= Chemical fertilizers
		2 = Organic fertilizers (non-chemicals like animal manure, green manure, compost, etc.) (Go to→1505)
1504.	Which chemical fertilizers are used most often, can you specify the type/name?	1 = UREA
		2 = DAP
		3= NPS
		98= Don't know
		99= Other (specify): …………………………………..
1505.	Which organic fertilizers are used more often, can you specify the type?	1 = Livestock manure
		2 = Poultry manure
		99=Other (specify) …………………………………………
1506.	What is the most dominant cereals/crop you produce on your farm?<br>***[Only one answer is allowed****! Do not read the choices. Listen, mark/specify the one they mention*]	1 = Wheat
		2 = Teff
		3 = Maize
		99= Other (Specify): …………………………………..
1507.	How far is your agricultural land from your house?<br>*[In case they own many farms, Consider only the one with the dominant crop]*	1= within 500 meter radius
		2= 500 -1000 meter radius
		3= 1000 - 3000 meter radius
		4= More than 3000 meter

Module 15: Soil information questionnaire

Observational checklist for soil sampling

Woldeyohannes M, *et al. BMJ Open* 2023; 13:e067641. doi: 10.1136/bmjopen-2022-067641

1	Region
2	Zone
3	Woreda
4	Kebele
5	Gote Code
6	Household Code
	Sample code …………………..
7	**Crop history**
7.1	Last two-year crop (Please, specify the crop harvested in 2011, growing season) …………………
7.2	Last crop (Please, specify the crop harvested from the previous growing season) …………………
7.3	Crop to be planted for the current season………………………..
7.4	Please write the intended planting dates (Year and Month) ……………………….,
7.5	When was the **last time** the agricultural field gets tilled?
	1 = 0 to 3 months ago
	2 = 3 to 6 months ago
	1 = 6 to 12 months ago
	2 = before a year
8	**Fertilizer utilization**
8.1	Which fertilizer is applied
	1 = Chemical fertilizer
	2 = Organic fertilizer **(Go to 8.4)**
	3 = Both 1&2
	4 = Fertilizer not applied **(Go to 9)**
8.2	Which Chemical fertilizer is applied
	1 = UREA
	2 = DAP
	3 = NPS
	4 = Other, please specify……………………………………..
8.3	Please write the last date (Year and Month) you applied chemical fertilizer?…………
8.4	Which Organic fertilizer is applied
	1 = Animal Manure
	2 = Green Manure
	3 = Compost
	4 = Other, please specify……………………………………..
8.5	Please write the **last date** (Year and Month) you applied organic fertilizer?…………
9	**Soil characteristics**

Woldeyohannes M, *et al. BMJ Open* 2023; 13:e067641. doi: 10.1136/bmjopen-2022-067641

9.1	Observation: What is the colour of the soil you are about to sample
	1 = Dark brown/Black
	2 = Red
	3 = Grey
	4 = Other, please specify…………………………………....
9.1	Observation: Field area landscape
	1 = plains/level grounds
	2 = Sloppy/Inclined
9.4	Observation: Is there a standing crop on the sampling field or to the nearby farmland.
	1 = Yes
	2 = No
9.3	Observation: Is sampling field tilled/ is it being tilled at the time of sampling.
	1 = Yes
	2 = No
10	Distance of the farmland to the nearby houses
	1 = below 100meter
	2 = 100 to 500meter
	3 = 500 to 1000meter
	4 = More than 1000meter
11	Please take picture for the surrounding environment i.e., plot, houses, anything permanent or even moving cattle
12	Please capture GPS for the sampled farmland
13	Name of sample collector………………………………………….....
	LIST ANY ABNORMAL CONDITIONS OR SPECIFIC INFORMATION DESIRED:

Module 16: A Dietary assessment questionnaire

Note for the data collectors: Among the household members, this module questionnaire is to be filled for the child under-five years of age and women of the reproductive age in the household.

Part 1: Before 24-hr recall

**24-hour dietary recall**			
**EA code |__|__|__| Household code |__|__| Line Number |__|__| Child ID |__|__|**<br>**Unique ID Woman: |__|__|__|__|__|__|__|__| Unique ID Child: |__|__|__|__|__|__|__|__|__|__|**<br>Interview Date: Date - _ _/_ _/_ _ _ _ **Day -** 01=Mon 02=Tue 03=Wed 04=Thu 05=Fri 06=Sat 07=Sun<br>Date of food intake - _ _/_ _/_ _ _ _			
	**Question**	**Coding category**	**skip**
1.	Enumerator Code:	______________________________	
2.	For which target group is the recall being done?	0. Woman 1. Child	
3.	Recall number	0. Recall 1<br>1. Recall 2	
4.	Name of the woman interviewed	____________________	
5.	Age of the woman (in complete years)	Age in years (__________)	
6.	Name of child	______________________________	
7.	Date of birth (DOB): Use Ethiopian calendar	|__|__|/|__|__|/20|__|__|	
8.	Age of the child (in complete month)	________months	
9.	Child's sex:	0=Male 1= Female	
10.	Food weighing scale number:	|__|__|	
11.	Was yesterday's food intake different from your usual diet?	1=Yes 0=No	No →13
12.	If yes,	1=Holyday/celebration<br>2=I was sick<br>3=Other	
13.	Was [child name] yesterday's food intake different from your usual diet?	1=Yes<br>0=No	No →15

14.	If yes,	1=Holyday/celebration<br>2=I was sick<br>3=Other	
15.	Did you take medicine/supplement yesterday?	1=Yes<br>0=No<br>***If yes, name:*** ___________________	
16.	Did [child name] take medicine/supplement yesterday?	1=Yes<br>0=No<br>***If yes, name:***__________________	

Procedures to collect the required information

1. **Pass 1: list all foods and drinks consumed during the 24-hour period.**
   Now I would like to ask you about the foods and drinks that [YOU/ YOUR CHILD] consumed yesterday from the time you work up until you went to sleep, sunrise yesterday to sunrise today. Please list all foods or drinks you ate, weather you ate or drank them at home or somewhere else. Please think about snacks and small meals as well as main meals.

   1) "WHAT WAS THE FIRST THING [you/ your child] ATE YESTERDAY AFTER SUNRISE?"
   2) "WHEN WAS THAT"
   3) "DID [you/your child] HAVE ANYTHING WITH THAT?
   4) "WHAT DID [you/he/she] HAVE?"
   5) "WHAT IS THE NEXT THING [you/ your child] ATE OR DRANK AND WHEN WAS THAT?"
   6) REPEAT questions 3-5 until you have a full record for both DAY AND NIGHT
      a. The reference period is from sunrise yesterday to sunrise this morning. If they wake up at a different time than sunrise, you can use the time from waking up yesterday until waking up today
2. **Pass 2: get more detail about each food**.
   7) "NOW, PLEASE DESCRIBE EACH FOOD [you/ your child] ATE YESTERDAY"
   8) "WHAT TYPE WAS IT?"
   9) "WHERE DID YOU GET IT?"
   10) "WHAT ARE THE INGREDIENTS?"
      a. Use standard "probes" (probing questions) to get these details for each food.
   11) "HOW MUCH DID THIS RECIPE MAKE?" or "WHAT WAS THE TOTAL AMOUNT THIS MADE?"
   12) "HOW WAS The Recipe PREPARED?"
      a. Identify the cooking methods used (particularly if raw, fermented, or fried in oil).

3. **Pass 3: estimate the amount consumed of each food on the list**

   13) "HOW MUCH OF [name the first food] DID [you/ your child] CONSUME?"
      a. Help the mother remember and **estimate the amount** of each food or recipe that her child ate and that she herself ate.

   14) "WAS ANY LEFT OVER?"
      a. If any food is leftover from what the mother served to the child, enter that amount.

   15) "PLEASE HELP ME ESTIMATE THE AMOUNT OF FOOD YOU ARE OR USED IN THE RECIPE"

   Use following portion size estimation method to estimate the amount of food/ingredient eaten or used in a recipie 1. Direct weight (g) 2.Proxy weight (g) 3. Water (g) 4. Number 5. Other (specify).

4. **Pass 4: verify everything consumed**
      a. Quickly read the information back to the respondent, "HAVE I FORGOTTEN TO ADD ANYTHING?"

Part 2. Quick list

Pass 1

Please describe the foods the foods and drinks that [YOU/ YOUR CHILD] consumed yesterday from the time you work up until you went to sleep (sunrise yesterday to sunrise today). Please list all foods or drinks you ate, weather you ate or drank them at home or somewhere else. Please think about snacks and small meals as well as main meals.

Write down all foods and drinks mentioned. When composite dishes are mentioned, ask for the list of ingredients
When the respondent has finished, probe for meals and snacks not mentioned.

Early morning	Mid-morning	Noon	Afternoon	Evening	Late evening

**Food No.**	**What was the first thing [YOU/ YOUR CHILD] ate or drank after sunrise yesterday? Any else?**	**Time of meal**	**Please describe this food / beverage/ ingredient:**	**How was this prepared?**	**Place of preparation**	**How was the food / Ing. [illegible]**	**Amount served**	**Amount left over**	**Amount eaten**	**Recipe information**			
										**State of each [illegible]**	**Cooking method of [illegible]**	**Total amount of recipe [illegible]**	**Links to food/ [illegible]**
1													
	Ingredient:		Description										
					NA								
					NA								
					NA								
					NA								
					NA								
2													
					NA								
					NA								
					NA								
					NA								
					NA								
3													
					NA								
					NA								
					NA								
					NA								
					NA								

Time of meal: 1. Early morning 2. Mid-Morning 3. Noon 4. Afternoon 5. Evening 6. Late evening

Place of preparation: 1. Home 2. Outside home

How was it prepared: 1=raw/ no change/ as purchased; 2=fermented; 3=fried; 04=cooked or boiled – wet heat; 5=baked/ grilled/ broiled – dry heat; 6=local miller; 7=blanched (dipped in boiling water); 8=other

Food No.	What was the first thing [YOU/ YOUR CHILD] ate or drank after sunrise yesterday? Any else?	Time of meal	Please describe this food / beverage/ ingredient:	How was this prepared?	Place of preparation	How was the food / Ing. measured	Amount served	Amount left over	Amount eaten	Recipe information			
										State of each ingredient	Cooking method of the recipe	Total amount of recipe prepared	Links to food/ recipes
1													
	Ingredient:		Description										
					NA								
					NA								
					NA								
					NA								
					NA								
2													
					NA								
					NA								
					NA								
					NA								
					NA								
3													
					NA								
					NA								
					NA								
					NA								

	What was the first	Time of	Please describe this food /	How was	Place of	Ho [illegible]	Am [illegible]	Am [illegible]	Am [illegible]	Recipe information			
					NA								

Time of meal: 1. Early morning 2. Mid-Morning 3. Noon 4. Afternoon 5. Evening 6. Late evening

Place of preparation: 1. Home 2. Outside home

How was it prepared: 1=raw/ no change/ as purchased; 2=fermented; 3=fried; 04=cooked or boiled – wet heat; 5=baked/ grilled/ broiled – dry heat; 6=local miller; 7=blanched (dipped in boiling water); 8=other

Module 17: Biomarkers collection tools

PRESCHOOL AGE CHILDREN (6-59 MONTHS)
ETHIOPIAN FOOD AND NUTRITION STRATEGY BASELINE SURVEY 2020/21
Biochemical and Health Related Data Collection Tool

IDENTIFICATION	
**HH00.** CLUSTER (EA) NAME……………………………	**HH01**. CLUSTER NUMBER: ☐☐☐
**HH02**. HH NUMBER: ☐☐	**HH03**. RESIDENCE (RURAL=1, URBAN=2): ☐☐
**HH04**. RESPONDENT LINE NUMBER: (SHOULD BE MOTHER/CAREGIVER) ☐☐	**HH05** CHILD LINE NUMBER ☐☐
**HH06. INTERVIEWER NAME__________________** **CODE:___________________**	**HH07. TEAM LEADER, NAME**:__________________ **CODE:___________________**
**HH08. SUPERVISOR NAME:___________________** **CODE.____________________**	

PRESCHOOL CHILDREN 6-59 MONTHS OLD

PART I: CHILD HEALTH QUESTIONS

I would like to ask you some health and food questions about your child. Fill or Circle the correct answer

**S.N**	**Questions**	**Response**	**SKIP**
1	**What is the birth date of the child? In day/month/ year (How many months old is this child?)**<br><br>NOTE FOR INTERVIEWERS<br>*(Screening question to verify that the date of birth of the child)*	Birth Date:<br>_____________<br><br>(Day/Month/Year)<br><br>Age in months ☐☐	
2	**Has (**child's name**) been diagnosed with anemia in the past 6 months?**	No……………………….0<br>Yes ……………………..1<br>Don't know………….98	

PART II: CHILD BIOCHEMICAL MEASUREMENT

Consent given for: (Y OR N) **PL01** Blood ☐ **PL02** Stool ☐	
**PL03** Code for Laboratory Technician: ☐☐	Lab Tech Name ____________
**PL04** BLUE TOP TUBE (METAL FREE)<br>Not collected =00.0 Refused = 77.7	ML. ☐☐ • ☐
**PL05** PURPLE TOP TUBE (EDTA)<br>Not collected =00.0<br>Refused = 77.7	ML. ☐☐ • ☐
**PL06** RED TOP TUBE (EDTA)<br>Not collected =00.0<br>Refused = 77.7	ML. ☐☐ • ☐
**PL07** Date blood sample taken (Ethiopian Day/Month/Year)	Date: ______/_______/__________<br>Day / Month / Year
**PL08** TIME BLOOD DRAW (Ethiopian time)	Blood draw ___ ___ : ___ __ Hour Minute
**PL09 When did you eat your most recent meal (food)?** (Ethiopian time)	___ ___ : ___ ___<br>Hour Minute
**PL10** MALARIA RESULTS (RDK)	NEGATIVE...0<br>POSITIVE P FALCIPARUM ....1<br>Positive P VIVAX..................................2<br>INVALID...3
**PL11** FEVER in last 24 HR?	NO...0<br>YES 1
**PL12 HEMOGLOBIN** RESULTS	g/dL ☐☐ • ☐
**In order to determine if you have worms in the stool, we would like to collect a stool sample from your child. If you can provide this now, we appreciate it. If not now, we can come back to pick up the sample at a later time.**<br>*INSTRUCTIONS IF UNABLE TO PRODUCE AT WILL*:<br>**For stool**: We will return tomorrow to pick up your stool. We would like the freshest stool you can give us. Please use one cup to collect the first stool you pass.	
**PL13** STOOL COLLECTED?	NO.............................0 YES 1
**PL14** Date stool sample taken (Ethiopian Day/Month/Year)	Date: ______/_______/__________<br>Day / Month / Year
**PL15** TIME: STOOL COLLECTED (Ethiopian time)	___ ___ : ___ ___

	Hour Minute
**PL16** TIME: STOOL PASSED, Ethiopian time (as recorded on cup)	___ ___ : ___ ___<br>Hour Minute
**PL17 Time Blood centrifuged (Ethiopian time)**	___ ___ : ___ ___<br>Hour Minute

Thank you for completing this interview.

INTERVIEWER'S OBSERVATIONS
TO BE FILLED IN AFTER COMPLETING INTERVIEW
COMMENTS ABOUT RESPONDENT:

____
____
____
____
____
____
____
____

SCHOOL AGE CHILDREN 6-12 YEARS
ETHIOPIAN FOOD AND NUTRITION STRATEGY BASELINE SURVEY 2020/21
Biochemical and Health Related Data Collection Tool

IDENTIFICATION	
**SG01**. CLUSTER NUMBER: ☐☐☐	
**SG02**. HH NUMBER: ☐☐	
**SG03**. RESPONDENT LINE NUMBER: (SHOULD BE MOTHER/CAREGIVER) ☐☐	
**SG04** SCHOOL CHILD LINE NUMBER ☐☐	

In general, for children 6-10 years of age: get parental report (ask the questions of the caretaker and enter the child's name into the parentheses)
For children 11-12 years of age who are present and can provide information: get self-report (ask questions directly of the child and enter "you" or "yourself" into the parentheses)

PART I: CHILD HEALTH RELATED QUESTIONS

No.	QUESTION	CODING CATEGORIES		SKIP
**S1**	How old are you/is your child? *(**Verify that the age is the same age as written on the household listing)***	Years		
**S2**	Have you/ your child ever attended school?	No.............................<br>Yes	00<br>01	**00** →S4
**S3**	What is the highest level of school (name of child) completed?	None.............................0<br>Primary 1		

PART II: CHILD BIOCHEMICAL MEASUREMENT

Verbal consent given for: **SL01** Blood **SL02** Urine **SL03** Stool<br>0= No OR 1= yes	
**SL04** Phlebotomist Code	
**SL5** BLUE TOP TUBE (METAL FREE)<br>**Did not work =00.0**<br>**Refused = 77.7**	ML. __ __ • __
**SL6** PURPLE TOP TUBE (EDTA)<br>**Did not work =00.0**<br>**Refused = 77.7**	ML. __ __ • __
**SL7** REDTOP TUBE (EDTA)<br>**Did not work =00.0**<br>**Refused = 77.7**	ML. __ __ • __
**SL8** DATE BLOOD SAMPLE TAKEN (Ethiopian calendar)	Date: ______/_______/__________<br>Day / Month / Year
**SL9** TIME BLOOD DRAW (Ethiopian time)	Blood draw ___ ___ : ___ ___<br>Hour Minute
**SL10 When did you eat your most recent meal (food)?** (Ethiopian time)	Last Meal Eaten ___ ___ : ___ ___<br>Hour Minute
**SL11** FEVER in last 24 HR? (Since same time yesterday)	No..00<br>Yes 01
**SL12** MALARIA RESULTS (RDK)	NEGATIVE....................................00<br>POSITIVE P *falciparum* ....01<br>POSITIVE P *vivax* ....02<br>INVALID 03

**SL13** HEMOGLOBIN RESULTS	g/dL [ ][ ] . [ ]
**SL14** Is that finger prick or venous sample taken?	Finger prick………………………………00<br>Venous ……………………………………01
**In order to determine if you have blood in urine or worms in stool we would like to collect a urine and stool sample. If you can provide this now, we appreciate it. If not now, we can come back to pick up the sample at a later time.**	
**SL15 U**rine collected?	No…………………………………………………..00<br>yes …………………………………………………..01
**SL16** Blood in urine RESULTS	Negative……………………………………………..00<br>positive ………………………………………………01
**SL17** Stool collected?	No……………………………………………………..00<br>yes……………………………………………………..01
**SL18** Date and **t**ime when stool passed by the respondent (as recorded on cup) (Ethiopian time)	Date: __/_____/______ and ___ ___ : ___ ___<br>Day / Month /Year Hour Minute
**SL19** Date stool sample taken (Ethiopian calendar)	Date: ______/_______/__________<br>Day / Month / Year
**SL20** Time when stool collected from the respondent (Ethiopian time)	___ ___ : ___ ___<br>Hour Minute
**SL21** TIME BLOOD centrifuged (Ethiopian time)	___ ___ : ___ ___<br>Hour Minute

Thank the respondent and tell them that the lab team will be arriving later.

INTERVIEWER'S OBSERVATIONS

TO BE FILLED IN AFTER COMPLETING INTERVIEW

COMMENTS:

____

____

____

____

ADELESCENT GIRLS (10-19 YEARS)
ETHIOPIAN FOOD AND NUTRITION STRATEGY BASELINE SURVEY 2020/21
Biochemical and Health Related Data Collection Tool

IDENTIFICATION	
**HH00.** CLUSTER (EA) NAME……………………………	**HH01**. CLUSTER NUMBER:
**HH02**. HH NUMBER:	**HH03**. RESIDENCE (RURAL=1, URBAN=2):
**HH04**. RESPONDENT LINE NUMBER: (SHOULD BE MOTHER/CAREGIVER)	**HH05** WOMEN LINE NUMBER
**HH06**. INTERVIEWER NAME__________________ CODE:___________________	**HH07**. TEAM LEADER, NAME:__________________ CODE:____________________
**HH08.** SUPERVISOR NAME:___________________ CODE.____________________	

PART I: HEALTH RELATED QUESTIONS

I would like to ask you some health and food questions about yourself. Fill or Circle the correct answer

No.	Question	Coding categories	Skip
1	How old are you?<br>(*verify that the age is the same age as written on the household listing*)	Years	
2	Have you been diagnosed with anemia in the past six months?	No……………………………………0<br>Yes ………………………………1<br>Don't know………………………98	
3	Do you smoke? (do not include the powder and chew type)	No………………………0<br>Yes ………………………1	

PART II: ADOLESCENT BIOCHEMICAL MEASUREMENT

Consent given for: 0= No or 1= Yes AG**01** Blood AG**L02** Stool	
**AG03** BLUE TOP TUBE (METAL FREE)<br>**Did not work =00.0**	ML.

**Refused = 77.7**	
**AG04** PURPLE TOP TUBE (EDTA)<br>**Did not work =00.0**<br>**Refused = 77.7**	ML. ☐☐ • ☐
**AG05** REDTOP TUBE (EDTA)<br>**Did not work =00.0**<br>**Refused = 77.7**	ML. ☐☐ • ☐
**AG06** Date blood sample taken (Ethiopian calendar)	Date: ______/_______/__________<br>Day / Month / Year
**AG07** TIME BLOOD DRAW (Ethiopian time)	Blood draw ___ ___ : ___ ___<br>Hour Minute
**AG08 When did you eat your most recent meal (food)?** (Ethiopian date and time)	______/_______/__________ __ ___ : ___ ___<br>Date /Month/ Year Hour Minute
**AG09 Is it Finger prick or venous blood sample taken?**	01 Finger prick<br>02 Venous
**AG09** MALARIA RESULTS (RDT)	NEGATIVE…………………………………. 00<br>POSITIVE P *falciparum* …………………………. 01<br>POSITIVE P *vivax* …………………………………. 02<br>POSITIVE FOR BOTH P *falciparum and* P *vivax …… 03*<br>INVALID ………………………………………………………. 04
**AG10 HEMOGLOBIN** RESULTS	g/dL ☐☐ • ☐

In order to determine if you have worms in the stool we would like to collect a stool sample. If you can provide this now, we appreciate it. If not now, we can come back to pick up the sample at a later time.
INSTRUCTIONS IF UNABLE TO PRODUCE AT WILL:
For stool: We will return tomorrow to pick up your stool. We would like the fresh stool you can give us. Please use one cup to collect the first stool you pass.

**AG11** Stool collected?	No……………………………………………………00<br>yes ……………………………………………………01
**AG12** Date stool sample taken (Ethiopian calendar)	Date: ______/_______/__________<br>Day / Month / Year
**AG13** Time when stool passed by the respondent (as recorded on cup) (Ethiopian time)	___ ___ : ___ ___<br>Hour Minute
**AG14** Time when stool collected from the respondent (Ethiopian time)	___ ___ : ___ ___

	Hour Minute
**AG15** TIME BLOOD centrifuged (Ethiopian time)	___ ___ : ___ ___<br>Hour Minute

OBSERVATIONS

TO BE FILLED IN AFTER COMPLETING INTERVIEW

COMMENTS:
____
____
____

WOMEN OF REPRODUCTIVE AGE 15-49 YEAR OLDS

ETHIOPIAN FOOD AND NUTRITION STRATEGY BASELINE SURVEY 2020/21

Biochemical and Health Related Data Collection Tool

IDENTIFICATION	
**HH00.** CLUSTER (EA) NAME……...........................	**HH01**. CLUSTER NUMBER: ☐☐☐
**HH02**. HH NUMBER: ☐☐	**HH03**. RESIDENCE (RURAL=1, URBAN=2): ☐☐
**HH04**. RESPONDENT LINE NUMBER: (SHOULD BE MOTHER/CAREGIVER) ☐☐	**HH05** WOMEN LINE NUMBER ☐☐
HH06. INTERVIEWER NAME__________________ CODE:___________________	HH07. TEAM LEADER, NAME:_____________ CODE:____________________
HH08. SUPERVISOR NAME:___________________ CODE.____________________	

PART I: HEALTH RELATED QUESTIONS

**S.N**	**QUESTION**	**Response**	**SKIP**
1	How old are you?<br>(*verify that the age is the same age as written on the household listing*)	☐☐<br>Years	
Now I would like to ask you some questions about your health. I will first ask you about the last 6 months.			
2	Have you been diagnosed with anemia in the past six months?	No…………….............0	

		Yes ……………………1<br>Don't know…………98	
2	Have you been ill with malaria in the past 2 weeks?	No………………………..0<br><br>Yes ……………………1<br>Don't know…………98	
3	Do you smoke? (do not include the powder and chew type)	No……………….0<br><br>Yes ……………..1	
4	Are you currently lactating?	No……………….0<br><br>Yes ……………..1	
5	During that last pregnancy (that resulted in a live birth) did you have difficulty with your vision at night ("Dafent" night blindness in local language)?	No………………………..0<br><br>Yes ……………………1<br>Don't know…………98	
6	In the first two months after delivery, did you receive a<br><br>vitamin A dose (like this)?<br><br>*SHOW THE CAPSULE*	No………………………..0<br><br>Yes …………………1<br>Don't know…………98	

PART II: WOMEN BIOCHEMICAL MEASUREMENT

**If the women is pregnant do not collect venous blood**	
Consent given for: 0= No or 1= Yes	**WL01** Blood ☐ **WL02** Urine ☐ **WL03** Stool ☐
**WL4** BLUE TOP TUBE (METAL FREE)<br>**Did not work =00.0**<br>**Refused = 77.7**<br>**Pregnant = 99.9**	ML. ☐☐ • ☐
**WL5** PURPLE TOP TUBE (EDTA)<br>**Did not work =00.0**<br>**Refused = 77.7**<br>**Pregnant = 99.9**	ML. ☐☐ • ☐
**WL6** REDTOP TUBE (EDTA)<br>**Did not work =00.0**<br>**Refused = 77.7**<br>**Pregnant = 99.9**	ML. ☐☐ • ☐
**WL7** Date blood sample taken (Ethiopian calendar)	Date: ______/_______/__________

	Day / Month / Year
**WL8** TIME BLOOD DRAW (Ethiopian time)	Blood draw ___ ___ : ___ ___<br><br>Hour Minute
**WL9 When did you eat your most recent meal (food)?** (Ethiopian date and time)	______/_______/__________ __ ___ : ___ ___<br><br>Date /Month/ Year Hour Minute
**WL 10 Finger prick or venous sample taken?**	01 Finger prick<br><br>02 Venous
**WL11** MALARIA RESULTS (RDT)	NEGATIVE…………………………………………….. 00<br>POSITIVE P *falciparum* ……………………………….. 01<br>POSITIVE P *vivax* …………………………………….. 02<br>POSITIVE FOR BOTH P *falciparum and* P *vivax* …………… *03*<br>INVALID ……………………………………………………….. 04
**WL12** HEMOGLOBIN RESULTS	g/dL [ ][ ] • [ ]

**In order to determine if you have blood in the urine or worms in the stool we would like to collect a urine and stool sample. If you can provide this now, we appreciate it. If not now, we can come back to pick up the sample at a later time.**<br>*INSTRUCTIONS IF UNABLE TO PRODUCE AT WILL*:<br>**For stool**: We will return tomorrow to pick up your stool. We would like the fresh stool you can give us. Please use one cup to collect the first stool you pass.<br>**For urine**: We will return tomorrow to pick up your urine.

**WL13 U**rine collected?	No……………………………………………………….00<br>yes ……………………………………………………..01
**WL14** RESULTS (blood in urine)<br>**Ask the women if she is Menstruating**<br>(Don't test if the women is in Menstruation)	Negative…………………………………………….00<br>positive ……………………………………………..01<br><br>Women is Menstruating……………………….03
**WL15** Stool collected?	No……………………………………………………00<br>yes ……………………………………………………01
**WL16** Date stool sample taken (Ethiopian calendar)	Date: ______/_______/__________<br><br>Day / Month / Year
**WL17** Time when stool passed by the respondent (as recorded on cup) (Ethiopian time)	___ ___ : ___ ___<br>Hour Minute
**WL18** Time when stool collected from the respondent (Ethiopian time)	___ ___ : ___ ___

	Hour Minute
**WL19** TIME BLOOD centrifuged (Ethiopian time)	___ ___ : ___ ___<br>Hour Minute

OBSERVATIONS

TO BE FILLED IN AFTER COMPLETING INTERVIEW

COMMENTS:

____
____
____
____
____
