# Supplementary material for: Ethiopia National Food and Nutrition Survey to inform the Ethiopian National Food and Nutrition Strategy: a study protocol
Source: BMJ Open. 2023 Apr 25;13(4):e067641. doi: 10.1136/bmjopen-2022-067641 (PMC10151871; doi:10.1136/bmjopen-2022-067641)
Supplement: Supplementary data [file bmjopen-2022-067641supp013.pdf]

Supplementary consent 1

1. **Informed Consent Form for Household Head**

Ethiopian Food and Nutrition Strategy (FNS) Baseline Survey

Investigator(s): Dr.Masresha Tessema (PI), Meseret W/Yohannes, Dr. Meron Girma, Alemnesh Petros, Dr Aregash Samuel, Arnaud Laillou, Stanley Chitekwe, Kaleab Baye, Ramadhani Noor, Anne Sophie Donze and other co-authors

You are being invited to take part in this research because you are head of household. There are about [16596] households taking part in this research. We will ask you about household characteristics, and socio-economic status of your household. We will also assess dietary intake, anthropometric status, nutrition sensitive and specific indicators and micronutrient status of your household member.

Box 1. Taking part in this research is voluntary

- You **can refuse** to take part in this study.
- You **can withdraw** your participation from the study at any time

Information related to the study

The FNS baseline survey will be conducted in the 12 regions of Ethiopia. The study population will be children age 0-59 months having caregivers/mothers, school-age children (6-12 years), adolescent girls (10-19 years), reproductive-age women (15-49 years), pregnant and lactating women, and household head. The indicators that will be collected for the survey will be dietary intake, anthropometric status, nutrition sensitive and specific indicators and micronutrient status.

The expected possible adverse effects: there is no adverse effect by participating in this study

The objective of this research: to produce information on anthropometric status, dietary intakes, and micronutrient status of different population groups in Ethiopia, and assess the coverage of direct and indirect nutrition interventions.

Study design: A population-based, cross-sectional design

The schedule of the study: The study will be conducted from July, 2021 to April, 2023

Foreseeable risks and expected benefits arising from participation in the study

**Foreseeable risks**	**Expected benefits**
Risks to study participants for involvement in the coverage survey are low. There may be risks associated	The findings of the study will help the ministry of health and other stakeholders engaged in nutrition

with COVID pandemic. Interviewers will be trained to minimize this risk and will use appropriate prevention measures.	to improve and/or design appropriate health and nutrition intervention programs in the country.

Occurrences that may take place during the study period

**Occurrences**	**How to manage**
Withdrawal of volunteers from the study	In such a case, we would respect the volunteer's decision to withdraw and also get a clear understanding of the reason for their withdrawal

At the end of the study, you will not be receiving any financial benefits, but will get your results for height, weight, mid upper arm and waist circumference measurements, anemia and goiter status for time you spent and participation.

All data collected from the study will be kept confidential. If you have any questions related to the study you may contact directly Dr. Masresha Tessema who is the project PI.

The contact persons

1. Dr. Masresha Tessema Tel. [+251 919782082] E-mail: [masresha88@gmail.com] or
2. [Mr. Ibrahim Kedir] Tel. [+251 911957161] EPHI's IRB

**Certificate of Consent**	
I have read the foregoing information. I have an opportunity to ask questions and all my quest have been answered to my satisfaction. I volunteer to give consent to participate in this research study<br><br>_________________________<br>Printed name of the participant<br>________________________<br>Signature of the participant<br>Date ________________<br>day/month/year	I confirm that the participant was given an opportunity to ask questions about the study and all questions have been answered correctly. I confirm that the consent has been given voluntarily<br><br>_______________________________<br>Printed name of the person taking the consent<br>______________________<br>Signature of the person taking the consent<br>Date___________________<br>day/month/year

2. Informed Consent Form for Women of Reproductive Age

Ethiopian Food and Nutrition Strategy (FNS) Baseline Survey

Investigator(s): Dr.Masresha Tessema (PI), Meseret W/Yohannes, Dr. Meron Girma, Alemnesh Petros, Dr Aregash Samuel, Arnaud Laillou, Stanley Chitekwe, Kaleab Baye, Ramadhani Noor, Anne Sophie Donze and other co-authors

You are being invited to take part in this research because you are women of reproductive age. There are [16596] households taking part in this research. We will assess your dietary intake, anthropometric status, nutrition sensitive and specific indicators and micronutrient status

Box 1. Taking part in this research is voluntary

- You **can refuse** to take part in this study.
- You **can withdraw** your participation from the study at any time

Information related to the study

The FNS baseline survey will be conducted in the 12 regions of Ethiopia. The study population will be children age 0-59 months having caregivers/mothers, school-age children (6-12 years), adolescent girls (10-19 years), reproductive-age women (15-49 years), pregnant and lactating women, and household head. The indicators that will be collected for the survey will be dietary intake, anthropometric status, nutrition sensitive and specific indicators and micronutrient status.

The expected possible adverse effects: there is no adverse effect by participating in this study

The objective of this research: to produce information on anthropometric status, dietary intakes, and micronutrient status of different population groups in Ethiopia, and assess the coverage of direct and indirect nutrition interventions.

Study design: A population-based, cross-sectional design

The schedule of the study: The study will be conducted from July, 2021 to April, 2023

Foreseeable risks and expected benefits arising from participation in the study

Foreseeable risks	Expected benefits
Risks to study participants for involvement in the coverage survey are low. There may be risks associated with COVID pandemic. Interviewers will be trained to minimize this risk and will use appropriate prevention measures.	The findings of the study will help the ministry of health and other stakeholders engaged in nutrition to improve and/or design appropriate health and nutrition intervention programs in the country.

Occurrences that may take place during the study period

**Occurrences**	**How to manage**
Withdrawal of volunteers from the study	In such a case, we would respect the volunteer's decision to withdraw and also get a clear understanding of the reason for their withdrawal

At the end of the study, you will not be receiving any financial benefits, but will get your results for height, weight, mid upper arm and waist circumference measurements, anemia and goiter status for time you spent and participation.

All data collected from the study will be kept confidential. If you have any questions related to the study you may contact directly Dr. Masresha Tessema who is the project PI.

The contact persons

1. Dr. Masresha Tessema Tel. [+251 919782082] E-mail: [masresha88@gmail.com] or

2. [Mr. Ibrahim Kedir] Tel. [+251 911957161] EPHI's IRB

**Certificate of Consent**	
I have read the foregoing information. I have an opportunity to ask questions and all my quest have been answered to my satisfaction. I volunteer to give consent to participate in this research study	I confirm that the participant was given an opportunity to ask questions about the study and all questions have been answered correctly. I confirm that the consent has been given voluntarily
________________________ Printed name of the participant<br>_______________________ Signature of the participant<br>Date ________________<br>day/month/year	______________________________ Printed name of the person taking the consent<br>______________________ Signature of the person taking the consent<br>Date___________________<br>day/month/year

3. Informed Consent Form for Pregnant Women

Ethiopian Food and Nutrition Strategy (FNS) Baseline Survey

Investigator(s): Dr.Masresha Tessema (PI), Meseret W/Yohannes, Dr. Meron Girma, Alemnesh Petros, Dr Aregash Samuel, Arnaud Laillou, Stanley Chitekwe, Kaleab Baye, Ramadhani Noor, Anne Sophie Donze and other co-authors

You are being invited to take part in this research because you are pregnant women. There are [16596] households taking part in this research. We will assess your, anthropometric status, nutrition sensitive and specific indicators and anemia status

Box 1. Taking part in this research is voluntary

- You **can refuse** to take part in this study.
- You **can withdraw** your participation from the study at any time

Information related to the study

The FNS baseline survey will be conducted in the 12 regions of Ethiopia. The study population will be children age 0-59 months having caregivers/mothers, school-age children (6-12 years), adolescent girls (10-19 years), reproductive-age women (15-49 years), pregnant and lactating women, and household head. The indicators that will be collected for the survey will be dietary intake, anthropometric status, nutrition sensitive and specific indicators and micronutrient status.

The expected possible adverse effects: There is no adverse effect by participating in this study

The objective of this research: to produce information on anthropometric status, dietary intakes, and micronutrient status of different population groups in Ethiopia, and assess the coverage of direct and indirect nutrition interventions.

Study design: a population-based, cross-sectional design

The schedule of the study: The study will be conducted from July, 2021 to April, 2023

**Foreseeable risks and expected benefits arising from participation in the study**	
**Foreseeable risks**	**Expected benefits**
Risks to study participants for involvement in the coverage survey are low. There may be risks associated with COVID pandemic. Interviewers will be trained to minimize this risk and will use appropriate prevention measures.	The findings of the study will help the ministry of health and other stakeholder engaged in nutrition to improve and/or design appropriate health and nutrition intervention programs in the country.
**Occurrences that may take place during the study period**	

**Occurrences**	**How to manage**
Withdrawal of volunteers from the study	In such a case, we would respect the volunteer's decision to withdraw and also get a clear understanding of the reason for their withdrawal

At the end of the study, you will not be receiving any financial benefits, but will get your results for height, weight, mid upper arm circumference measurements, anemia and goiter status for time you spent and participation.

All data collected from the study will be kept confidential. If you have any questions related to the study you may contact directly Dr. Masresha Tessema who is the PI

The contact persons

1. Dr. Masresha Tessema

Tel. [+251 919782082] E-mail: [masresha88@gmail.com]

2. [Mr. Ibrahim Kedir] Tel. [+251 911957161]

**Certificate of Consent**	
I have read the foregoing information. I have an opportunity to ask questions and all my quest have been answered to my satisfaction. I volunteer to give consent to participate in this research study	I confirm that the participant was given an opportunity to ask questions about the study and all questions have been answered correctly. I confirm that the consent has been given voluntarily
________________________<br>Printed name of the participant<br>_______________________<br>Signature of the participant<br>Date ________________<br>day/month/year	______________________________<br>Printed name of the person taking the consent<br>______________________<br>Signature of the person taking the consent Date<br>___________________<br>day/month/year

4. Informed Consent Form for Preschool Child

Ethiopian Food and Nutrition Strategy (FNS) Baseline Survey

Investigator(s): Dr.Masresha Tessema (PI), Meseret W/Yohannes, Dr. Meron Girma, Alemnesh Petros, Dr

Aregash Samuel, Arnaud Laillou, Stanley Chitekwe, Kaleab Baye, Ramadhani Noor, Anne Sophie Donze and other co-authors

You are being invited to take part in this research because you are either a mother or caregiver who has a child under the age of 5 years (0-59 months). There are [16596] households taking part in this research. We would collect a sample of your child's dietary information, blood, urine and stool. And, we will also measure your child's height/ length, weight, and mid upper arm circumference. Finally we will assess, your child's eye for bitot spot

Box 1. Taking part in this research is voluntary

☐You **can refuse** to take part in this study.

☐You **can withdraw** your participation from the study at any time

Information related to the study

The FNS baseline survey will be conducted in the 12 regions of Ethiopia. The study population will be children age 0-59 months having caregivers/mothers, school-age children 6-12 years), adolescent girls (10-19 years), reproductive-age women (15-49 years), pregnant and lactating women, and household head. The indicators that will be collected for the survey will be dietary intake, anthropometric status, nutrition sensitive and specific indicators and micronutrient status.

The expected possible adverse effects :there is no adverse effect by participating in this study

The objective of this research: to produce information on anthropometric status, dietary intakes, and micronutrient status of different population groups in Ethiopia, and assess the coverage of direct and indirect nutrition interventions.

**Study design:** a population-based, cross-sectional design	
**The schedule of the study:** The study will be conducted from July, 2021 to April, 2023	
**Foreseeable risks and expected benefits arising from participation in the study**	
**Foreseeable risks**	**Expected benefits**
Risks to study participants for involvement in the coverage survey are low. There may be risks associated with COVID pandemic. Interviewers will be trained to minimize this risk and will use appropriate prevention measures.	The findings of the study will help the ministry of health and other stakeholder engaged in nutrition to improve and/or design appropriate health and nutrition intervention programs in the country.
**Occurrences that may take place during the study period**	
**Occurrences**	**How to manage**

Withdrawal of volunteers from the study	in such a case, we would respect the volunteer's decision to withdraw and also get a clear understanding of the<br><br>reason for their withdrawal

At the end of the study, you will not be receiving any financial benefits, but will get your results for height/length, weight, mid upper arm circumference measurements, and anemia for time you spent and participation.

All data collected from the study will be kept confidential. If you have any questions related to the study you may contact directly Dr. Masresha Tessema who is the project principal investigator

The contact persons

1. Dr. Masresha Tessema

Tel. [+251 919782082] E-mail: [masresha88@gmail.com]

2. [Mr. Ibrahim Kedir] Tel. [+251 911957161] EPHI's IRB

Certificate of Consent	
I have read the foregoing information. I have an opportunity to ask questions and all my quest have been answered to my satisfaction. I volunteer to give consent to participate in this research study<br><br>_________________________<br>Printed name of the participant<br>________________________<br>Signature of the participant's parent or guardian<br>Date ________________<br>day/month/year	I confirm that the participant was given an opportunity to ask questions about the study and all questions have been answered correctly. I confirm that the consent has been given voluntarily<br><br>_______________________________<br>Printed name of the person taking the consent<br>______________________<br>Signature of the person taking the consent Date<br>___________________<br>day/month/year

5. **Informed Consent Form for School Age Children**

Ethiopian Food and Nutrition Strategy (FNS) Baseline Survey

Investigator(s): Dr.Masresha Tessema (PI), Meseret W/Yohannes, Dr. Meron Girma, Alemnesh Petros, Dr Aregash Samuel, Arnaud Laillou, Stanley Chitekwe, Kaleab Baye, Ramadhani Noor, Anne Sophie Donze and other co-authors

You are being invited to take part in this research because you are either a mother or caregiver who has a school-age child. Among children 6 – 12 years, we will collect your child's blood, urine and stool.

Box 1. Taking part in this research is voluntary

☐ You **can refuse** to take part in this study.

☐ You **can withdraw** your participation from the study at any time

Information related to the study

The FNS baseline survey will be conducted in the 12 regions of Ethiopia. The study population will be children age 0-59 months having caregivers/mothers, school-age children (6-12 years), adolescent girls (10-19 years), reproductive-age women (15-49 years), pregnant and lactating women, household head. The indicators that will be collected for the survey will be dietary intake, anthropometric status, nutrition sensitive and specific indicators and micronutrient status.

The expected possible adverse effects: there is no adverse effect by participating in this study

The objective of this research: to produce information on anthropometric status, dietary intakes, and micronutrient status of different population groups in Ethiopia, and assess the coverage of direct and indirect nutrition interventions.

Study design: a population-based, cross-sectional design

The schedule of the study: The study will be conducted from July, 2021 to April, 2023

**Foreseeable risks and expected benefits arising from participation in the study**	
**Foreseeable risks**	**Expected benefits**
Risks to study participants for involvement in the coverage survey are low. There may be risks associated with COVID pandemic. Interviewers will be trained to minimize this risk and will use appropriate prevention measures.	The findings of the study will help the ministry of health and other stakeholder engaged in nutrition to improve and/or design appropriate health and nutrition intervention programs in the country.
**Occurrences that may take place during the study period**	
**Occurrences**	**How to manage**

Withdrawal of volunteers from the study	In such a case, we would respect the volunteer's decision to withdraw and also get a clear understanding of the reason for their withdrawal

At the end of the study, you will not be receiving any financial benefits, but you will get your **anemia** and **goiter** status for time you spent and participation.

All data collected from the study will be kept confidential. If you have any questions related to the study you may contact directly Dr. Masresha Tessema who is the project's PI

The contact persons

1. Dr. Masresha Tessema

Tel. [+251 919782082] E-mail: [masresha88@gmail.com]

2. [Mr. Ibrahim Kedir] Tel. [+251 911957161] EPHI's IRB

**Certificate of Consent**	
I have read the foregoing information. I have an opportunity to ask questions and all my quest have been answered to my satisfaction. I volunteer to give consent to participate in this research study<br>________________________<br>Printed name of the participant<br>_______________________<br>Signature of the participant's parent or guardian<br><br>Date ________________<br>day/month/year	I confirm that the participant was given an opportunity to ask questions about the study and all questions have been answered correctly. I confirm that the consent has been given voluntarily<br><br>______________________________<br>Printed name of the person taking the consent<br>______________________<br>Signature of the person taking the consent Date<br>___________________<br>day/month/year

6. Assent form for Adolescent Girls (10-19 years)

Ethiopian Food and Nutrition Strategy (FNS) Baseline Survey

Investigator(s): Dr.Masresha Tessema (PI), Meseret W/Yohannes, Dr. Meron Girma, Alemnesh Petros, Dr Aregash Samuel, Arnaud Laillou, Stanley Chitekwe, Kaleab Baye, Ramadhani Noor, Anne Sophie Donze and other co-authors

You are being invited to take part in this research because you are Adolescent girl. There are [16596] households taking part in this research. We will measure your dietary information (for those adolescent girls aged 15-17 years), information related to nutrition -sensitive and nutrition-specific practices, blood, and stool, we will also measure your height, weight, and mid upper arm and waist circumference and your goiter status

Box 1. Taking part in this research is voluntary

☐ You **can refuse** to take part in this study.

☐ You **can withdraw** your participation from the study at any time

Information related to the study

The FNS baseline survey will be conducted in the 12 regions of Ethiopia. The study population will be children age 0-59 months having caregivers/mothers, school-age children (6-12 years), adolescent girls (10-19 years), reproductive-age women (15-49 years), pregnant and lactating women, and household head. The indicators that will be collected for the survey will be dietary intake, anthropometric status, nutrition sensitive and specific indicators and micronutrient status

The expected possible adverse effects: There is no adverse effect by participating in this study

The objective of this research: to produce information on anthropometric status, dietary intakes, and micronutrient status of different population groups in Ethiopia, and assess the coverage of direct and indirect nutrition interventions.

**Study design:** a population-based, cross-sectional design	
**The schedule of the study:** The study will be conducted from July, 2021 to April, 2023	
**Foreseeable risks and expected benefits arising from participation in the study**	
**Foreseeable risks**	**Expected benefits**
Risks to study participants for involvement in the coverage survey are low. There may be risks associated with COVID pandemic. Interviewers will be trained to minimize this risk and will use appropriate prevention measures.	The findings of the study will help the ministry of health and other stakeholder engaged in nutrition to improve and/or design appropriate health and nutrition intervention programs in the country.

**Occurrences that may take place during the study period**	
**Occurrences**	**How to manage**
Withdrawal of volunteers from the study	In such a case, we would respect the volunteer's decision to withdraw and also get a clear understanding of the reason for their withdrawal

At the end of the study, you will not be receiving any financial benefits, but will get your results for height, weight, mid upper arm and waist circumference measurements, anemia and goiter status for time you spent and participation.

All data collected from the study will be kept confidential. If you have any questions related to the study you may contact directly Dr. Masresha Tessema who is the project's PI

The contact persons

1. Dr. Masresha Tessema

Tel. [+251 919782082] E-mail: [masresha88@gmail.com]

2. [Mr. Ibrahim Kedir] Tel. [+251 911957161] EPHI's IRB

**Certificate of Assent**	
I have read the foregoing information. I have an opportunity to ask questions and all my quest have been answered to my satisfaction. I volunteer to give assent to participate in this research study<br><br>________________________<br>Printed name of the participant<br>________________________<br>Signature of the participant<br>Date ________________<br>day/month/year	I confirm that the participant was given an opportunity to ask questions about the study and all questions have been answered correctly. I confirm that the assent has been given voluntarily<br><br>______________________________<br>Printed name of the person taking the assent<br>______________________<br>Signature of the person taking the assent Date<br>___________________<br>day/month/year
