# Supplementary material for: Ethiopia National Food and Nutrition Survey to inform the Ethiopian National Food and Nutrition Strategy: a study protocol
Source: BMJ Open. 2023 Apr 25;13(4):e067641. doi: 10.1136/bmjopen-2022-067641 (PMC10151871; doi:10.1136/bmjopen-2022-067641)
Supplement: Author's manuscript [file bmjopen-2022-067641.draft_revisions.pdf]

BMJ Open

BMJ Open is committed to open peer review. As part of this commitment we make the peer review history of every article we publish publicly available.

When an article is published we post the peer reviewers' comments and the authors' responses online. We also post the versions of the paper that were used during peer review. These are the versions that the peer review comments apply to.

The versions of the paper that follow are the versions that were submitted during the peer review process. They are not the versions of record or the final published versions. They should not be cited or distributed as the published version of this manuscript.

BMJ Open is an open access journal and the full, final, typeset and author-corrected version of record of the manuscript is available on our site with no access controls, subscription charges or pay-per-view fees (http://bmjopen.bmj.com).

If you have any questions on BMJ Open's open peer review process please email info.bmjopen@bmj.com

BMJ Open

Ethiopia National Food and Nutrition Survey to inform the Ethiopian Food and Nutrition Strategy: A study protocol

Journal:	*BMJ Open*
Manuscript ID	bmjopen-2022-067641
Article Type:	Protocol
Date Submitted by the Author:	25-Aug-2022
Complete List of Authors:	Woldeyohannes, Meseret ; Ethiopian Public Health Institute<br>Girma, Meron; Ethiopian Public Health Institute, National Information Platforms for Nutrition<br>Petros, Alemnesh; Ethiopian Public Health Institute<br>Hussen, Alemayehu ; Ethiopian Public Health Institute<br>Samuel, Aregash; Ethiopian Public Health Institute<br>Dinssa, Danial ; Ethiopian Public Health Institute, Environmental and Public Health Research<br>Challa, Feyissa; Ethiopian Public Health Institute<br>Laillou, Arnaud; UNICEF, Dakar, Senegal<br>Chitekwe, Stanley; UNICEF Ethiopia<br>Baye, Kaleab ; Addis Ababa University,<br>Noor , Ramadhani; UNICEF Ethiopia<br>Donze, Anne Sophie; UNICEF Ethiopia<br>Tollera, Getachew; Ethiopian Public Health Institute<br>Dangiso, Mesay; Ethiopian Public Health Institute<br>Tadesse, Lia; Ethiopia Ministry of Health<br>Zelalem, Meseret; Ethiopia Ministry of Health<br>Tessema, Masresha; Ethiopian Public Health Institute,
Keywords:	EPIDEMIOLOGY, NUTRITION & DIETETICS, PUBLIC HEALTH

SCHOLARONE™
Manuscripts

Ethiopia National Food and Nutrition Survey to Inform the Ethiopian Food and Nutrition Strategy: A Study Protocol

Meseret Woldeyohannes[1], Meron Girma[1], Alemnesh Petros[1], Alemayehu Hussen[1], Aregash Samuel[1], Daniel Abera Dinssa[1], Feyissa Challa[1], Arnaud Laillou[2], Stanley Chitekwe[3], Kaleab Baye[4], Ramadhani Noor[3], Anne Sophie Donze[3], Getachew Tollera[1], Mesay Hailu Dangiso[1], Lia Tadesse[5], Meseret Zelalem[5], Masresha Tessema[1*]

[1] Ethiopian Public Health Institute, Addis Ababa, Ethiopia
[2] Regional Nutrition Specialist, United Nations Children's Fund (UNICEF) Regional Office, Dakar, Senegal
[3] United Nations Children's Fund (UNICEF), Addis Ababa 12000, Ethiopia
[4] Center for Food Science and Nutrition, Addis Ababa University, Addis Ababa 12000, Ethiopia
[5] Ethiopian Ministry of Health, Addis Ababa, Ethiopia

*Correspondence: Masresha Tessema, P.O.Box 1242 Ethiopian Public Health Institute, dr.masresha.tessema@gmail.com

Running title: National Food and Nutrition Strategy Survey

For peer review only

For peer review only - http://bmjopen.bmj.com/site/about/guidelines.xhtml

ABSTRACT

Introduction Ethiopia has made significant progress in reducing malnutrition in the past two decades. Despite such improvements, a substantial segment of the country's population remains chronically undernourished and suffers from not only micronutrient deficiencies but also from increasing diet-related non-communicable diseases. This survey aims to assess the anthropometric status, dietary intake and micronutrient status of Ethiopian children, women, and adolescent girls. The study will also assess the coverage of direct and indirect nutrition-related interventions, and map agricultural soil nutrients. The survey will serve as a baseline for the recently developed Ethiopian food system transformation plan and will inform the implementation of the National Food and Nutrition Strategy.

Methods and analysis Using a population-based cross-sectional survey, the study will collect data in the ten regions and two city administrations of Ethiopia. The study population will be women of reproductive age, children aged 0-59 months, school-aged children and adolescent girls. A total of 16,596 households will be surveyed, allowing us to generate national and regional estimates for selected indicators. A two-stage stratified cluster sampling procedure will be used to select households. In the first stage, 639 enumeration areas will be selected using probability-proportional to size allocation. In the second stage 26 eligible households will be selected within each enumeration area using systematic random selection. Primary outcomes include coverage of direct and indirect nutrition interventions, infant and young child feeding practices, food insecurity, dietary intakes for women, mental health of women and children, anthropometric status, micronutrient status, and soil nutrient status.

Ethics and dissemination The study protocol was approved by the Institutional Review Board of the Ethiopian Public Health Institute (protocol no: EPHI-IRB-317-2020). The finding of this survey will be disseminated in scientific forums, national research conference and stakeholder meetings; and will be submitted for publication in peer reviewed journals.

Strengths and limitations of this study

- To the best of our knowledge, this will be among -if not-the first study to simultaneously collect data on anthropometric status, 24hr recall quantitative dietary intakes and the determination of micronutrient status in the same participants or household, while at the same time capturing data on the food system.
- Previously, nutrition programs in Ethiopia have relied on data from small-scale studies and population-based surveys such as the Ethiopia Demographic and Health Surveys. The study will help to improve understanding of nutritional problem across multiple facets—from agricultural soil to people to the environment.
- Inherent to the cross-sectional design of the study, the findings of this study cannot be used to establish cause and effect.
- The study design prevents us from considering seasonal differences in nutritional outcomes and determinants.

For peer review only - http://bmjopen.bmj.com/site/about/guidelines.xhtml

INTRODUCTION

Globally, one in three are affected by one of more forms of malnutrition.[1] Women and children are particularly vulnerable to malnutrition due to increased physiological nutrient needs required to support fetal and child growth.[2] Nutritional deprivation during early life impairs growth and development, leading to poor school performance, reduced productivity, and loss of earnings in later life.[3] Consequently, the first 1000 days of life, from conception to the child's second year of life, was recognized as a critical window of opportunity to effectively prevent malnutrition.[3 4] Adolescence is also identified as a second window of opportunity to correct nutritional inadequacies and adversities faced in early life, but little is known about this life stage.

Despite significant progress over the past two decades, the burden of malnutrition in Ethiopia remains high.[5-7] Nationally, 37% of Ethiopian children under five years of age are stunted[7], and 22% of women of reproductive age (WRA) are chronically undernourished (Body Mass Index (BMI) < 18.5 Kg/m^2).[5] Only 14% of children under two years of age consumed the minimum number of recommended food groups.[5] Furthermore, micronutrient deficiencies co-exist with chronic energy deficiency.[8] This along with the ongoing nutrition transition, characterized by shifts in diets[9], is further complicating the nutrition landscape by increasing the prevalence of overweight and non-communicable diseases.[5] Nearly a fifth (16%) of Ethiopian adults are estimated to be hypertensive, and 3% are diabetic.[10] Therefore, addressing not only undernutrition but all forms of malnutrition is critical.

The Sustainable Development Goals (SDGs) recognize the importance of nutrition, primarily driven by the need to mitigate its detrimental consequences. Further, the 2012 World Health Assembly (WHA) identified global targets to be achieved by 2025 that aim to reduce stunting, anemia, low birth weight, and childhood obesity. These targets are used to track progress in SDG goal 2: Zero hunger.[11]Recognizing the importance of good nutrition, the Government of Ethiopia has made ending malnutrition a national priority. Ethiopia started implementing its first National Nutrition Program in 2008.[12] The second phase of this program (2011-2016) was a multisectoral program aimed at accelerating progress in reducing malnutrition.[13] Moreover, Ethiopia's first Food and Nutrition Policy was endorsed in 2018[14] , followed the National Food and Nutrition Strategy (FNS)[15] which was launched in 2021 to provide a framework for the operationalization of the policy. Acceleration of progress in the reduction of malnutrition requires the design and implementation of direct and indirect nutrition interventions that can be implemented at scale. To this end, understanding the various factors contributing to the different forms of malnutrition is critical.

Multiple factors operating at the immediate, underlying, and basic levels contribute to malnutrition.[2] Inadequate dietary intake and poor health are immediate determinants.[2] Household food security, child care practices, access to health services, and healthy environments are underlying determinants.[16] Structural and contextual factors such as economic structures, political, environmental, social and cultural factors are the basic determinants of malnutrition.[2] The contribution of these factors varies across different contexts, and target groups, but studies capturing all these factors in a single survey are scant. The lack of timely and comprehensive information on nutritional status across critical life stages and their determinants is a bottleneck that is preventing Ethiopia from designing effective interventions. Up to date and comprehensive data on the coverage of direct and indirect nutrition

For peer review only - http://bmjopen.bmj.com/site/about/guidelines.xhtml

interventions delivered across various implementing sectors of the food and nutrition strategy is not available yet. This is unfortunate as such data could inform the implementation of the food and nutrition strategy, but also serve as a baseline to which progress can be tracked against.

Therefore, this study aimed to provide the first ever comprehensive information on the nutritional status of different populations in Ethiopia to support evidence-based implementation of the national food and nutrition strategy.

Objectives of the survey

The overall goal of this study is to produce nationally and regionally representative estimates on anthropometric status, coverage of nutrition interventions, dietary intakes, and micronutrient status for children, adolescent girls, and women of reproductive age in Ethiopia.

Specific objectives include;

1. Assess the coverage of nutrition-specific and nutrition-sensitive interventions.
2. Assess food consumption patterns and nutrients intakes of children aged 6–59 months, and women of reproductive age.
3. Assess the micronutrient status of children (vitamin A, anemia, iron, iodine and zinc), adolescent girls, and women of reproductive age (vitamin A, vitamin D, anemia, iron, iodine, zinc, folate, vitamin B_{12})
4. Assess the anthropometric status of under-5 children, school-age children (6-12 years), adolescent girls, and women of reproductive age.
5. Assess the geographical distribution of soil micronutrient status in Ethiopian agricultural soil.

METHODS AND ANALYSIS

Study design

This study is a nationally and sub-nationally (regionally) representative cross-sectional survey that will characterize dietary intake, micronutrient status, and access to nutrition-related services for different target populations. Given that soil nutrient content can influence micronutrient content of foods and hence affect nutrient intake, the soil nutrient composition will also be analyzed . The study will have four main components. The first component will assess nutrition-specific and nutrition-sensitive indicators (NSS) for all target groups (children aged 0-59 months and WRA, school-age children, adolescent girls)using semi-structured questionnaires. The second component will measure quantitative dietary intake for children aged 6-59 months and WRA (15-49 years). The third component of the survey will collect biomarker samples from all children (6-59 months), school-age children (6-12 years), adolescent girls (10-19 years), and WRA (15-49 years). The final component of the study will measure micronutrients in agricultural soils. The study data will be collected from July 2021 to December 2022.

Study setting

Ethiopia has an estimated population size of 120 million and is the second most populous country in Africa.[17] The majority of its population resides in rural areas (70%).[17] Agriculture accounts for 40% of the country's gross domestic product.[17] Children aged 15 years and younger make up 40% of the Ethiopian population in 2021.[18] Ethiopia is administratively divided into 10 regions and two city administrations. This study will be conducted in all of the regions and city administrations of the country. Figure 1 provides a geographic representation of the study areas.

For peer review only - http://bmjopen.bmj.com/site/about/guidelines.xhtml

Study participants

The target population of this study are i) women of reproductive age (WRA) aged 15-49 years ii) children aged 0-59 months iii) school age children aged 6-12 years, and iv) adolescent girls aged 10-19 years, and V) household head.

Sample size calculations

Sample size was estimated to guarantee adequate precision to generate national and regional estimates for selected indicators for each study target group. Indicators used for each target group are shown in Table 1S in supplemental materials. The required number of households and target groups was calculated using a single population proportion formula at the regional level. We used region-specific prevalence estimates for indicators, a 5% margin-of-error, a design effect of 1.5, a household response rate of 95%, and an individual response rate of 80%. The initial sample size was then adjusted for region-specific average household size and percentage of the target population from the total population. An indicator that provides the maximum number of households was used to estimate the final sample size for each region. Regional sample sizes were summed up to derive the total (national) sample size. Based on these calculations, the total sample size for the overall survey was 16,596 households (Supplemental material Table 2S).

For WRA, dietary and biomarker data will be collected in half of the selected households within each Enumeration Area (EA). This selection will yield a total sample size of 7,386 WRA (50% of the expected 14,772 WRA). The sample size needed to assess dietary intakes and micronutrient status of WRA was calculated using the prevalence of inadequate zinc intake, which yielded the largest sample size.[8 19]

Sampling procedures

A two-stage stratified cluster sampling procedure will be used to select households. In the first stage, 639 EAs, 257 urban and 382 rural will be selected using probability-proportional to-size allocation. We will use the 2018 Ethiopia Population and Housing Census enumeration areas sampling frame to select EAs (the Primary Sampling Units (PSUs)). The Central Statistical Agency (CSA) prepared the enumeration areas sampling frame. An EA typically contains 100-150 households. EA maps will be used to delineate the boundaries of the selected EA. In the second stage of sampling to identify eligible households, all households with the EA will be listed. A household will be eligible for selection if at least one of the study target groups are residents (de jure) or stayed at the household the night before the interview (de factor).

Twenty-six (26) eligible households will be selected within each EA using systematic random selection. All target groups will be eligible for the NSS interview in the selected households. All children aged 6-59 months will also be eligible for dietary assessment. Women residing in 13 households (Out of 26 households) who will be selected randomly will be eligible for dietary assessment. Biomarker samples will be collected for all children under 5 years of age, school-age children, and adolescents in the selected households. Similar to dietary assessment, biomarker samples will be collected for women residing in half of the selected households.

For peer review only - http://bmjopen.bmj.com/site/about/guidelines.xhtml

Outcomes

Coverage of direct and indirect nutrition interventions

A structured questionnaire will be used to determine the coverage of direct and indirect nutrition interventions provided to children aged 6-59 months, WRA, and adolescent girls. Direct nutrition interventions included vitamin A supplementation, iron supplementation, zinc supplementation, growth monitoring and promotion, nutrition counseling services, and food fortification. Water, sanitation and hygiene, coverage of food or cash assistance program, women empowerment, and mental health will be some nutrition-sensitive indicators considered in this study. We will use standard indicator definitions proposed by the Data for Decisions to Expand Nutrition Transformation project (DataDENT) to assess coverage of nutrition programs.

Anthropometric status

Using standardized procedures, anthropometric measurements, including weight, height/length, and mid-upper arm circumference, will be taken for all study target populations.[20] Anthropometric indices (weight-for-height z-scores, length/height-for-age z-scores, weight-for-age z-scores, BMI-for-age z-scores) will be calculated using the WHO 2006 child growth standards and the WHO 2007 child growth reference data. Stunting (length/height-for-age z-scores below -2 SD), wasting (weight-for-height z-scores below -2 SD), underweight (weight-for-age z-scores below -2 SD), thinness (BMI-for-age z-scores below -2 SD) and BMI will be the primary anthropometric outcomes of interest.

Infant and young child feeding practices

Infant and young child feeding practices will be assessed using the new World Health Organization (WHO) and United Nations Children's Fund (UNICEF) recommended 17 indicators to evaluate IYCF practices.[21]

Food insecurity

The Food Insecurity Experience Scale (FIES) will be used to assess household food security.[22] The FIES consists of eight questions that assess household experience related to adequate food access. Experience questions range from worrying about getting enough food to not eating for a whole day.

In addition to these outcome indicators, information on the sociodemographic characteristic of households, child health, maternal health, employment status, and household agricultural practices will be collected using structured questionnaires.

Mental health of women

Common mental health disorders will be assessed using the WHO Self-reporting questionnaire which consists of 20 questions. Women will be classified as having a common mental health disorder if row score was greater or equal to 6 out of 20.[23]

Assessment of dietary intakes of children and WRA

We will measure dietary intake for children aged 6-59 months and WRA. A one-day quantitative multiple-pass 24-hour recall will be conducted to assess dietary intakes. The interactive multiple-pass 24-hour recall interview consists of four steps designed to enhance memory.[24] All days of the week will be proportionately represented during the dietary survey to account for the day of the week effects on food

For peer review only - http://bmjopen.bmj.com/site/about/guidelines.xhtml

intake. To account for the day-to-day variability of dietary intake within individuals, a second non-consecutive day 24-hr recall (repeat) will be collected (within 2 to 10 days of the first recall) on a randomly selected sub-sample of WRA and children. The number of repeats needed is determined by allocating for each region 50 repeats, which is then multiplied by a design effect of 1.5 and a 10% non-response rate. The number of repeats was rounded up to 1244 recalls for each target group to ensure that the minimum number of repeats (n=83) needed from each region would be collected. Detailed non-standard recipe ingredient data will be collected for all mixed dishes that were prepared at home.

Dietary assessment pre-survey work: We carried out pre-survey work to aid dietary data collection following recommendation set by the Intake: Center for Dietary Assessment.[25 26] An initial step was developing a food and ingredient list containing a comprehensive list of food items, mixed dishes, and ingredients expected to be consumed by the study target groups. The food list was generated using data from the first 2011 Ethiopian National Food Consumption Survey.[19] Other common foods consumed across the regions in Ethiopia were derived from the 2016 Household Income and Expenditure Surveys,[27] the Ethiopian Food Composition Tables, and dietary intake data from other recent dietary assessment surveys conducted by the Ethiopian Public Health Institute (EPHI). Portion size estimation methods suitable for large-scale studies were pre-selected for use in the survey following Intake recommendations.[28] The selected methods were direct measurement of actual foods consumed, standard unit: size and number, proxy measurement using play dough, water, rice, and maize flour, and finally using food price to estimate the amount of food consumed. Portion size estimation methods were assigned for all foods included in the food list.

Assessment of micronutrient status:

Biological specimens will be collected from the study population to determine serum retinol, ferritin, soluble transferrin receptor (sTfR), zinc, folate, vitamin B_{12}, and red blood cell (RBC) folate. Additionally, markers of inflammation, alpha(1)-acid glycoprotein (AGP), high-sensitivity C-reactive protein (hsCRP) will also be measured. We will also analyze parasites from stool specimens. All laboratory analyses will be performed at the EPHI Clinical chemistry, and Food Science and Nutrition Laboratories. Both laboratories participate in an external quality assessment scheme and are accredited by the Ethiopian National Accreditation Office (ENAO). Collection, storage, and analytical procedures for blood, urine, stool, and salt samples are described below.

Blood sample collection and analysis: Venous blood samples (5-7 mL) will be collected using vacutainer tubes following standard operating procedures.[29] Trace mineral-free vacutainer tubes will be used to collect blood for trace metal analysis. After collection, blood samples will be allowed to clot for 30 minutes in cold boxes (<8 °c). Samples will then be centrifuged at 3000 rpm (revolution per minute) for 10 minutes. The separated serum will be aliquoted and stored in -20°c portable freezers in the field. Samples will then be transported to EPHI and stored at -80°c until analysis. Hemoglobin will be measured in the field using Hemocue® (Hb 301, Hemocue AB, Angelholm, Sweden)[30].[30] If the hemoglobin values are below WHO cutoff point(11g/dl), the phlebotomist will send whole blood samples to the EPHI laboratory to identify hemoglobinopathies using electrophoresis method.[31] Malaria test will be conducted on-site using Bioline™ Malaria Ag P.f rapid diagnostic test kits (RDT) for P. falciparum and P. vivax).[32] Serum

For peer review only - http://bmjopen.bmj.com/site/about/guidelines.xhtml

soluble transferrin receptor (sTfR), AGP, hsCRP, folate, red blood cell (RBC) folate, vitamin B_{12}, and ferritin will be measured using Cobas 6000 analyzer (Roche Diagnostics GmbH, Mannheim, Germany). Serum retinol will be measured using high-performance liquid chromatography (HPLC) method[32], and serum zinc and selenium will be measured using a microwave plasma atomic emission spectrometers (MP-AES) analyzer.

***Stool and urine sample collection and analysis**:* Stool samples will be collected using stool cups and stored in 10 % formalin to preserve the parasite until analysis.[33] A portion of each stool sample will be used to detect direct ova, larvae and cysts of intestine parasites using formal ether concentration technique.[34]Urine samples will be collected from WRA and school-age children using 60 ml urine cup containers. Samples will be stored at -20°c. Urinary iodine excretion will be assessed by Sandell Kolthoff reaction at EPHI Laboratory using Shimadzu 1800 UV-Vis spectroscopy.[35]

***Salt collection and analysis*:** Salt samples will be collected from households with WRA for whom dietary data will be collected. At least 25 grams (one coffee cup) of salt will be collected to determine iodine content using the iodometric titration method.[36]

Assessment of nutrients in the soil

Soil samples will be collected from three households in each EA. Zig-zag or cross sampling method will be used to collect 10 to 20 subsamples (0-30 cm depth) constituting one composite sample. Subsamples will be collected at a separation distance of five meters. After thoroughly mixing composite samples, 1 kg soil sample will be transferred to polyethylene bags. The collected soil samples will be air-dried in wooden trays and disaggregated using a ceramic mortar and pestle (soil grinder) at the EPHI soil laboratory. Samples will then pass through a 6 mm sieve of stainless-steel screens to remove debris and homogenize the soil sample. The sieved fraction will be further pulverized to pass through a 1 mm sieve for the micronutrient analysis. Soil zinc, iron, copper, and manganese will be determined following standard procedures.[37] Micronutrient content will be determined using inductively coupled plasma-optical emission spectroscopy (ICP-OES) after extraction with diethylene triamine penta acetic acid (DPTA). Additional variables that affect the mobility of micronutrients in the soil and their uptake into crops will also be measured. These variables include soil reaction (pH), electrical conductivity, organic matter, total nitrogen, and soil organic carbon content. Data collectors will also record topography, slope, cropping history, type, and fertilizer application information. Table 1 provides a summary of procedures for each of the four components of the survey by study target groups.

Data quality assurance and analysis

Training of trainers on components of the survey was held before training the data collectors and supervisors. After fifteen days of training on methodological procedures, questionnaires and quality assurance, the questionnaires were tested in a pilot group (in EAs not included in the actual survey), and adapted based on the received feedback from the survey team. The questionnaires (including the food list) were translated into local languages (Amharic, Oromifa, Tigrigna, Somali, and Afar) and back-translated to English to ensure the quality of the translation. The data collectors' measurements were standardized to ensure that the inter-observer variability was within tolerable limits. Supervisors received additional training on teamwork and on monitoring and supervising the data collection process. All data

For peer review only - http://bmjopen.bmj.com/site/about/guidelines.xhtml

collection tools are programmed using open-source software (ODK). Data quality checks were included during ODK programming to prevent data recording errors. These include restricted responses, filter insert choices, skip patterns, and defaults. During data collection daily data tracking forms will be completed to track completed surveys for each study components to prevent missing data. Before the start of the survey high frequency checks were identified and error tracking forms were designed to track data quality in real-time. These checks included completeness checks, target group tracking, and duplicate ID check. Random field-supervision visits will also be made to check data quality. Every day, collected data will be sent to the EPHI central server and imported into statistical software programs as comma-separated values (CSV) files. For laboratory analysis, a quality control chart will be used to ensure the internal and external quality control materials are in the acceptable range.

The primary data analysis will focus on computing frequencies and percentages for categorical variables and summary statistics (like means, medians SD, IQR) for summarizing continuous variables. Sample weights will be constructed based on the selection probabilities of EAs, eligible households, and non-response rates. All analyses will also be adjusted for the survey design. Additional subgroup analysis will be computed for variables with adequate sample sizes for each category. The Biomarkers Reflecting Inflammation and Nutrition Determinants of Anemia (BRINDA) Working Group's regression correction approach will be used to account for inflammation in the study of all micronutrients status using the biomarkers C-reactive protein (CRP) and AGP. Geostatistical analyses will be employed to determine the spatial patterns of micronutrient distribution in the soil and blood samples. The wealth index will be constructed using principal component analysis (PCA).[38] The Rasch model will be used to construct the Food Insecurity Experience Scale (FIES). [22] All analysis will be done using STATA 16 and ArcGIS/QGIS.

Patient and public involvement statement

No patient involved.

Dissemination

The study's findings will be disseminated through several communication channels, including stakeholder workshops, various local and International conferences and technical report. Additionally, the findings will be submitted as special issue to peer-reviewed journals.

DISCUSSION

This comprehensive, nationally representative survey will for the first time characterize simultaneously the dietary intake and micronutrient status of Ethiopian children, adolescent girls and WRA. Besides, the study assesses key drivers of malnutrition including soil nutrient composition, as well as coverage of direct and indirect nutrition interventions. The survey will provide key insights informing the implementation of Ethiopia's National Food and Nutrition Strategy.

High-quality and timely data is critical to assess the burden of nutritional problems, identify vulnerable populations and priority actions, track the implementation of nutrition programs, and assess impact.[39 40] Ethiopia conducted its first-ever food consumption survey in 2011.[19]and its micronutrient survey in 2015.[41] Both surveys were collected at different times, which made it difficult to link the two surveys. Besides, the causes and solutions of malnutrition are complex and multisectoral; hence, requiring data on multiple indicators from various sectors spanning from soil nutrient, diets,

For peer review only - http://bmjopen.bmj.com/site/about/guidelines.xhtml

micronutrient status, to access and exposure to direct and indirect nutrition interventions. In this regard, this survey is uniquely positioned to integrate data from multiple domains to support evidence-based decision making for improved diets, nutrition, and overall wellbeing.

This study will allow us to evaluate progress relative to the last food consumption and micronutrient surveys, but more importantly will serve as a baseline to which future progress related to the implementation of the food and nutrition strategy will be evaluated. Furthermore, the current survey will also serve as a baseline for the Ethiopian Food System Transformation Plan by capturing majority indicators used for monitoring food systems related progress. Thus, filling information gaps that could have impeded successful implementation of the National Food and Nutrition Strategy (FNS). By establishing 13 strategic objectives, the FNS is intended to be aligned with the strategic directions of the Food and Nutrition Policy (FNP). Each strategy direction includes initiatives, actions, and key performance indicators, as well as leading and collaborating sectors. The key performance indicators should be evaluated to determine the progress of each implementing sector's achievement. The current survey will provide up to date national and subnational information on the current food and nutrition situation in Ethiopia for different target populations as well as provide comprehensive list of indicators that are pertinent to the implementation of the policy.[39] In addition, this study will provide information on context-specific determinants for prioritizing direct and indirect actions that can be implemented across sectors taking into account the specific needs of different target populations.

Additionally, effective multisectoral interventions that address the immediate and underlying determinants of malnutrition must be implemented in order to accelerate the reduction of malnutrition in its all form.[39] These interventions need to address context-specific determinants to reduce malnutrition effectively.[39] The lack of timely and disaggregated information on the determinants of malnutrition is a bottleneck to preventing malnutrition, particularly among the most vulnerable target populations. This study will also provide information on the coverage and quality of interventions which can be used to contextualize National Food and Nutrition Strategy monitoring frameworks, monitor implementation and track progress towards global and local targets.

Although this study will provide regionally and nationally representative estimates for key indicators and critical life stages, it has several limitations. Inherent to the cross-sectional design of the study, the findings of this study cannot be used to establish cause and effect. Additionally, the design prevents us from considering seasonal differences in nutritional outcomes and determinants. This study also relies on self-reported data, which is subject to recall bias. Notwithstanding the above-mentioned limitations, this study is uniquely designed to combine the assessment of anthropometric status, 24hr recall quantitative dietary intakes and the determination of micronutrient status in the same participants, while at the same time capturing data on the food system. Additionally, the study will be evaluating micronutrients in the agricultural soil, which will expand our understanding of factors that influence nutrition. To the best of our knowledge, this will be among -if not-the first study to simultaneously collect these variables from the same household. This could contribute to a better understanding of nutritional problem across multiple facets—from soil to people to the environment. In the past, nutrition programs implemented in Ethiopia have relied on information provided from small-scale studies and population-based surveys such as the Ethiopia Demographic and Health Surveys.[5-7 42 43] Although these data sources

For peer review only - http://bmjopen.bmj.com/site/about/guidelines.xhtml

provide some information to track progress and tailor interventions, they only provide data on a limited number of nutrition indicators and do not measure dietary intakes and assess biomarker status. This study will fill these data gaps by providing information on comprehensive indicators that show the burden and spatial distribution of micronutrient deficiencies and shifts of dietary patterns. Additionally, this study will provide information on emerging determinants such as mental health and intake of nutrients such as folate and B_{12} that have not been included in previous studies. Finally, the inclusion of adolescent girls, and school-age children, will provide vital information on nutritional indicators for these target groups, which are often not included in other nationally representative surveys. This survey will also provide information on the coverage of direct interventions implemented in the health sectors and indirect interventions implemented in the agriculture, WASH, education and social protection sectors for whom scant data exists. Hence, this study will provide valuable information that will guide the implementation of strategic actions for the reduction of malnutrition in Ethiopia.

For peer review only

For peer review only - http://bmjopen.bmj.com/site/about/guidelines.xhtml

Acknowledgments:

Contributions: MT, AL, SC, MW, AP, AS, and MG conceived the study and drafted the original protocol. All authors participated in refining of the protocol. AH, MW, MG and MT played a major role in the statistical consideration. KB, AL, SC, GT, MH, LT and MZ supervised manuscript preparation. All authors took responsibility for reviewing, final editing and approval of the manuscript.

Funding: This work was supported by Power of Nutrition, BMGF and World Bank through UNICEF, NI, and GAIN

Competing interests: The authors declare that they have no conflicts of interest.

Patient consent for publication: Not required

Ethical approval:

The study protocol is approved by the Institutional Review Board of the Ethiopian Public Health Institute (protocol no: EPHI-IRB-317-2020). Written informed consent will be obtained from each respondent. Confidentiality of all collected data will be given high priority during each stage of data handling. Individual names and personal information of respondents will be kept confidential and data sets will be kept anonymous for analysis.

For peer review only

For peer review only - http://bmjopen.bmj.com/site/about/guidelines.xhtml

References

1. Fanzo J, Hawkes C, Udomkesmalee E, et al. 2018 Global Nutrition Report. 2019
2. Black RE, Victora CG, Walker SP, et al. Maternal and child undernutrition and overweight in low-income and middle-income countries. *The Lancet* 2013;382(9890):427-51. doi: 10.1016/s0140-6736(13)60937-x
3. Martorell R. Improved nutrition in the first 1000 days and adult human capital and health. *Am J Hum Biol* 2017;29(2) doi: 10.1002/ajhb.22952 [published Online First: 2017/01/25]
4. United Nations. Transforming our world: The 2030 agenda for sustainable development, 2015.
5. Central Statistical Agency [Ethiopia] and ICF. Ethiopia demographic and health survey 2016. Addis Ababa, Ethiopia and Calverton, Maryland, USA: CSA and ICF, 2016.
6. Central Statistical Agency [Ethiopia] and ORC Macro. Ethiopia demographic and health survey 2000. Addis Ababa, Ethiopia and Calverton, Maryland, USA: CSA and ORC Macro, 2001.
7. Ethiopian Public Health Institute (EPHI) [Ethiopia] and ICF. Ethiopia Mini Demographic and Health Survey 2019: Key Indicators. Rockville, Maryland, USA: EPHI and ICF, 2019.
8. Ethiopian Public Health Institute. Ethiopian national micronutrient survey report Ethiopian Public Health Institute, 2016.
9. Hassen I, Dereje M, Minten B, et al. Diet transformation in Africa: the case of Ethiopia. *Agricultural Economics* 2017
10. Ethiopian Public Health Institute, Federal Ministery of Health, World Health Organization. Ethiopia STEPS report in risk factors for non-communicable diaseses and prevalence of selected NCDs Addis Ababa,Ethiopi: Ethiopian Public Health Institute 2016.
11. World Health Organization. Global nutrition monitoring framework: Operational guidance for tracking progress in meeting targets for 2025. Geneva: WHO, 2017.
12. Federal Democratic Republic of Ethiopia. National Nutrtion Program: 2013-2015. Addis Ababa,Ethiopia, 2013.
13. Federal Democratic Republic of Ethiopia. National Nutrition Program: 2016-2020. Addis Ababa,Ethiopia., 2016.
14. Federal Democratic Republic of Ethiopia. Food and Nutrition Policy, 2018.
15. Federal Democratic Republic of Ethiopia. National Food and Nutrition Strategy (2021-2023), 2021.
16. Reinhardt K, Fanzo J. Addressing Chronic Malnutrition through Multi-Sectoral, Sustainable Approaches: A Review of the Causes and Consequences. *Front Nutr* 2014;1:13. doi: 10.3389/fnut.2014.00013 [published Online First: 2014/01/01]
17. Bank W. Population projections Ethiopia 2021 [
18. Bardosh KL, Hussein JW, Sadik EA, et al. Chicken eggs, childhood stunting and environmental hygiene: an ethnographic study from the Campylobacter genomics and environmental enteric dysfunction (CAGED) project in Ethiopia. *One Health Outlook* 2020;2(1) doi: 10.1186/s42522-020-00012-9
19. Ethiopian Public Health Institute. Ethiopian national food consumption survey Ethiopian Public Health Institute, 2013.
20. UNICEF Wa. Recommendations for data collection, analysis and reporting on anthropometric indicators in children under 5 years old. : World Health Organization and the United Nations Children's Fund (UNICEF), 2019.
21. WHO, UNICEF. Indicators for assessing infant and young child feeding practices: definitions and measurement methods. Geneva: World Health Organization and the United Nations Children's Fund (UNICEF), 2021.
22. Ville AS, Tsun Po JY, Sen A, et al. Food security and the Food Insecurity Experience Scale (FIES): ensuring progress by 2030. *Food Security* 2019;11(3):483-91. doi: 10.1007/s12571-019-00936-9
23. World Health Organization. Self-reported mental health questionnaires1994.

For peer review only - http://bmjopen.bmj.com/site/about/guidelines.xhtml

24. Gibson RS, Ferguson EL. An interactive 24-hour recall for assessing the adequacy of iron and zinc intakes in developing countries HarvestPlus 2008.

25. Vossenaar M, Arimond M, Deitchler M, et al. An overview of the main pre-survey tasks required for large-scale quantitative 24-Hour recall dietary surveys in low- and middle-income countries. Washington, DC: Intake – Center for Dietary Assessment/FHI Solutions., 2020.

26. Intake – Center for Dietary Assessment. Dietary Survey Protocol Template: An Outline to Assist with the Development of a Protocol for a Quantitative 24-Hour Dietary Recall Survey in a Low- or Middle-Income Country Washington, DC: Intake – Center for Dietary Assessment/FHI Solutions., 2020.

27. Central Statistical Agency[Ethiopia]. Household Consumption and Expenditure (HCE) Survey 2015/16: Analytical Report. Addis Ababa: CSA, 2016.

28. Vossenaar M, Lubowa A, Hotz C, et al. Considerations for the Selection of Portion Size Estimation Methods for Use in Quantitative 24-Hour Dietary Recall Surveys in Low- and Middle-Income Countries. Washington, DC: Intake – Center for Dietary Assessment/FHI Solutions, 2020.

29. Organization WH. Micronutrient survey manual. 2020

30. Jaggernath M, Naicker R, Madurai S, et al. Diagnostic Accuracy of the HemoCue Hb 301, STAT-Site MHgb and URIT-12 Point-of-Care Hemoglobin Meters in a Central Laboratory and a Community Based Clinic in Durban, South Africa. *PloS one* 2016;11(4):e0152184. doi: 10.1371/journal.pone.0152184 [published Online First: 2016/04/06]

31. Wieringa FT, Dahl M, Chamnan C, et al. The High Prevalence of Anemia in Cambodian Children and Women Cannot Be Satisfactorily Explained by Nutritional Deficiencies or Hemoglobin Disorders. *Nutrients* 2016;8(6) doi: 10.3390/nu8060348 [published Online First: 2016/06/25]

32. Lee GC, Jeon ES, Le DT, et al. Development and evaluation of a rapid diagnostic test for Plasmodium falciparum, P. vivax, and mixed-species malaria antigens. *Am J Trop Med Hyg* 2011;85(6):989-93. doi: 10.4269/ajtmh.2011.11-0265 [published Online First: 2011/12/07]

33. Demeke G, Fenta A, Dilnessa T. Evaluation of Wet Mount and Concentration Techniques of Stool Examination for Intestinal Parasites Identification at Debre Markos Comprehensive Specialized Hospital, Ethiopia. *Infect Drug Resist* 2021;14:1357-62. doi: 10.2147/IDR.S307683 [published Online First: 2021/04/17]

34. Kache R, Phasuk N, Viriyavejakul P, et al. Prevalence of soil-transmitted helminth infections and associated risk factors among elderly individuals living in rural areas of southern Thailand. *BMC public health* 2020;20(1):1882. doi: 10.1186/s12889-020-09986-7 [published Online First: 2020/12/09]

35. Machado A, Lima L, Mesquita RBR, et al. Improvement of the Sandell-Kolthoff reaction method (ammonium persulfate digestion) for the determination of iodine in urine samples. *Clin Chem Lab Med* 2017;55(9):e206-e08. doi: 10.1515/cclm-2016-1094 [published Online First: 2017/03/18]

36. Jooste PL, Strydom E. Methods for determination of iodine in urine and salt. *Best Pract Res Clin Endocrinol Metab* 2010;24(1):77-88. doi: 10.1016/j.beem.2009.08.006 [published Online First: 2010/02/23]

37. Lindsay WL, Norvell W. Development of a DTPA soil test for zinc, iron, manganese, and copper. *Soil Sci Soc Am J* 1978;42(3):421-28.

38. Karamizadeh S, Abdullah SM, Manaf AA, et al. An overview of principal component analysis. *Journal of Signal and Information Processing* 2020;4

39. Heidkamp RA, Piwoz E, Gillespie S, et al. Mobilising evidence, data, and resources to achieve global maternal and child undernutrition targets and the Sustainable Development Goals: an agenda for action. *The Lancet* 2021 doi: 10.1016/s0140-6736(21)00568-7

For peer review only

For peer review only - http://bmjopen.bmj.com/site/about/guidelines.xhtml

40. Victora CG, Christian P, Vidaletti LP, et al. Revisiting maternal and child undernutrition in low-income and middle-income countries: variable progress towards an unfinished agenda. *The Lancet* 2021 doi: 10.1016/s0140-6736(21)00394-9
41. Institute EPH. Ethiopian national micronutreint Survey. Addis Ababa, Ethiopia Ethiopian Public Health Institute

2016.
42. Central Statistical Agency [Ethiopia] and ICF. Ethiopia demographic and health survey 2011. Addis Ababa, Ethiopia and Calverton, Maryland, USA: CSA and ICF, 2011.
43. Central Statistical Agency [Ethiopia] and ORC Macro. Ethiopia demographic and health survey 2005. Addis Ababa, Ethiopia and Calverton, Maryland, USA: CSA and ORC Macro, 2006.

For peer review only

For peer review only - http://bmjopen.bmj.com/site/about/guidelines.xhtml

Figure 1.Map showing study enumeration areas across regions

For peer review only

For peer review only - http://bmjopen.bmj.com/site/about/guidelines.xhtml

For peer review only

For peer review only - http://bmjopen.bmj.com/site/about/guidelines.xhtml

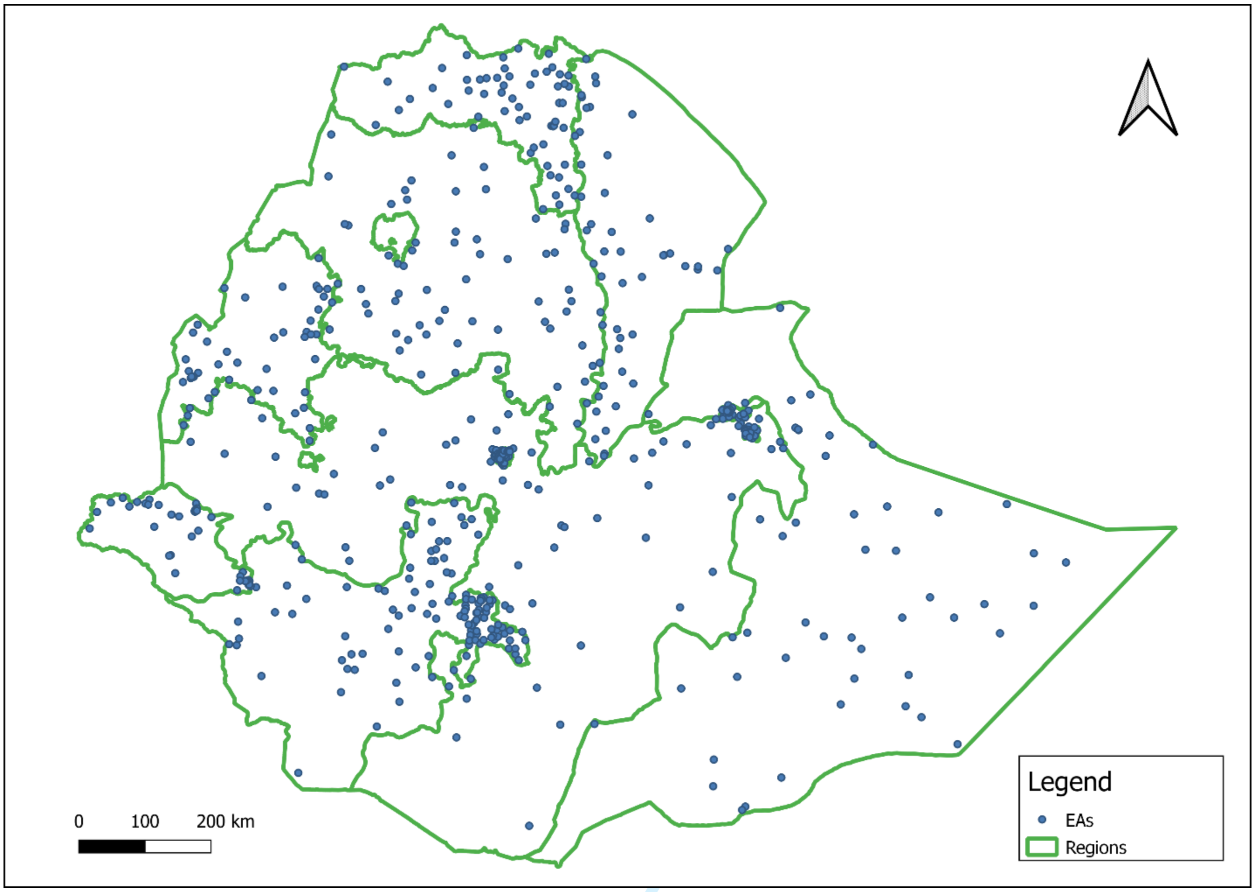

Figure 1. Map showing study enumeration areas across regions

For peer review only - http://bmjopen.bmj.com/site/about/guidelines.xhtml

Table 1. Summary of data collection procedures for each of the four components of the survey

	Child 0-5 months	Child 6-23 months	Child 24-59 months	School children 6-12 years	Adolescent girls 10-19 years	WRA 15-49 years	Household
**Nutrition specific and nutrition sensitive indicators (NSS)**							
Infant and young child feeding practices	X	X					
Nutritional information for adolescent girls					X		
Food insecurity							X
Water, sanitation and hygiene practices							X
Coverage of food fortification							X
Agricultural practices							X
Mental health						X	
Anthropometric status	X	X	X		X	X	
**Dietary assessment**							
24-hr recall quantitative dietary intake		X	X			X	
**Assessment of biomarker status**							
Blood sample		X	X	X	X	X	
Urine sample				X		X	
Stool sample		X	X	X	X	X	
Salt sample collection							X
**Assessment of micronutrients in the Soil**							
Soil micronutrient assessment							X

For peer review only

Table 1S. Key indicators used to estimate sample size for each target group

Target Group	Key indicators used to estimate sample size
Children under 5 years of age (0-59 months)	Vitamin A deficiency<br>Total goiter rate<br>Stunting<br>Any anemia<br>Zinc deficiency<br>Prevalence of inadequate intake of zinc
Women of reproductive age (15-49 years)	Vitamin A deficiency<br>Total goiter rate<br>Any anemia<br>Zinc deficiency<br>RBC folate<br>Serum folate<br>Vitamin B_{12}<br>Iodized salt use<br>Prevalence of inadequate intake of iron<br>Prevalence of inadequate intake of zinc<br>Prevalence of inadequate intake vitamin A
School age children (6 to 12 years)	Vitamin A deficiency<br>Total goiter rate<br>Any anemia<br>Iodine deficiency<br>Zinc deficiency
Adolescent girls (10 to 19 years)	Any anemia

For peer review only

Table 2S. Sample size determination and allocation

Region	Indicator used to estimate sample size	Number of EA	Number of HH	Expected number of Under 5 Children	Expected number of WRA	Expected number of 6-59 months children	Expected number of adolescent girls
Tigray	Any anemia	55	1,432	590	1,236	753	516
Afar	Stunting	51	1,328	539	1,096	695	406
Amhara	Stunting	61	1,585	619	1,253	843	531
Oromia	IDD	62	1,622	891	1,539	1,111	739
Somali	IDD	55	1,424	855	1,268	1,000	492
Benishangul-Gumuz	Stunting	49	1,282	555	1,127	732	475
SNNPR	Any anemia	59	1,528	818	1,492	1,000	692
Gambela	Any anemia	47	1,211	428	1,018	568	373
Harari	Any anemia	45	1,164	375	978	499	348
Addis Ababa	IDD/ TGR	54	1,411	413	1,274	405	262
Dire Dawa	Stunting	47	1,215	382	1,128	480	360
Sidama	Any anemia	54	1,395	747	1,363	914	632
Total sample size		639	16,596	7,213	14,772	9,001	5,824

For peer review only

STROBE Statement—checklist of items that should be included in reports of observational studies

	Item No	Recommendation	Reported on page #
**Title and abstract**	1	(*a*) Indicate the study's design with a commonly used term in the title or the abstract	Title p.1; Abstract p.2-3
		(*b*) Provide in the abstract an informative and balanced summary of what was done and what was found	
**Introduction**			
**Introduction** /rationale	4	Explain the scientific background and rationale for the investigation being reported	**Introduction** p.3-4
Objectives	5	State specific objectives, including any prespecified hypotheses	Background p.5 last statements
**Methods**			
Study design	5	Present key elements of study design early in the paper	Methods (data source) p.4
Setting	5	Describe the setting, locations, and relevant dates, including periods of recruitment, exposure, follow-up, and data collection	Methods (data source) p.5
Participants	5	(*a*) *Cohort study*—Give the eligibility criteria, and the sources and methods of selection of participants. Describe methods of follow-up<br>*Case-control study*—Give the eligibility criteria, and the sources and methods of case ascertainment and control selection. Give the rationale for the choice of cases and controls<br>*Cross-sectional study*—Give the eligibility criteria, and the sources and methods of selection of participants	Methods (participants) p.5-8
		(*b*) *Cohort study*—For matched studies, give matching criteria and number of exposed and unexposed<br>*Case-control study*—For matched studies, give matching criteria and the number of controls per case	
Variables	5	Clearly define all outcomes, exposures, predictors, potential confounders, and effect modifiers. Give diagnostic criteria, if applicable	Methods (all outcome and exposure variables are listed) p.6-8
Data sources/ measurement	NA	For each variable of interest, give sources of data and details of methods of assessment (measurement). Describe comparability of assessment methods if there is more than one group	Methods (methods of measurement indicated in) p.5-8
Bias	10	Describe any efforts to address potential sources of bias	Methods (data quality indicated) p.10-11
Study size	5-6	Explain how the study size was arrived at	Methods (data source,statistical

For peer review only

			analysis) p.5
Quantitative variables	10	Explain how quantitative variables were handled in the analyses. If applicable, describe which groupings were chosen and why	Methods (measurement and statistical analysis sections) p. 8-9
Statistical methods	10	(*a*) Describe all statistical methods, including those used to control for confounding	Methods (analysis section) p. 8-9
		(*b*) Describe any methods used to examine subgroups and interactions	
		(*c*) Explain how missing data were addressed	
		(*d*) *Cohort study*—If applicable, explain how loss to follow-up was addressed<br>*Case-control study*—If applicable, explain how matching of cases and controls was addressed<br>*Cross-sectional study*—If applicable, describe analytical methods taking account of sampling strategy	
		(*e*) Describe any sensitivity analyses	

Continued on next page

For peer review only

**Results**			
Participants	5*	(a) Report numbers of individuals at each stage of study—eg numbers potentially eligible, examined for eligibility, confirmed eligible, included in the study, completing follow-up, and analysed	NA
		(b) Give reasons for non-participation at each stage	
		(c) Consider use of a flow diagram	
Descriptive data	14*	(a) Give characteristics of study participants (eg demographic, clinical, social) and information on exposures and potential confounders	NA
		(b) Indicate number of participants with missing data for each variable of interest	
		(c) *Cohort study*—Summarise follow-up time (eg, average and total amount)	
Outcome data	15*	*Cohort study*—Report numbers of outcome events or summary measures over time	NA
		Case-control study—Report numbers in each exposure category, or summary measures of exposure	
		Cross-sectional study—Report numbers of outcome events or summary measures	
Main results	16	(*a*) Give unadjusted estimates and, if applicable, confounder-adjusted estimates and their precision (eg, 95% confidence interval). Make clear which confounders were adjusted for and why they were included	NA
		(*b*) Report category boundaries when continuous variables were categorized	
		(*c*) If relevant, consider translating estimates of relative risk into absolute risk for a meaningful time period	
Other analyses	17	Report other analyses done—eg analyses of subgroups and interactions, and sensitivity analyses	NA
**Discussion**			
Key results	18	Summarise key results with reference to study objectives	p. 9-10
Limitations	19	Discuss limitations of the study, taking into account sources of potential bias or imprecision. Discuss both direction and magnitude of any potential bias	Discussion (strengths and weaknesses of the study) p.10-11
Interpretation	20	Give a cautious overall interpretation of results considering objectives, limitations, multiplicity of analyses, results from similar studies, and other relevant evidence	Discussion (interpretation of findings in the context of existing research, meaning of the study:

For peer review only - http://bmjopen.bmj.com/site/about/guidelines.xhtml
For peer review only

			p.9-11 implication
Generalisability	21	Discuss the generalisability (external validity) of the study results	Discussion (strengths and weakness of the study) p.10-11
**Other information**			
Funding	22	Give the source of funding and the role of the funders for the present study and, if applicable, for the original study on which the present article is based	Funding p.12

*Give information separately for cases and controls in case-control studies and, if applicable, for exposed and unexposed groups in cohort and cross-sectional studies.

Note: An Explanation and Elaboration article discusses each checklist item and gives methodological background and published examples of transparent reporting. The STROBE checklist is best used in conjunction with this article (freely available on the Web sites of PLoS Medicine at http://www.plosmedicine.org/, Annals of Internal Medicine at http://www.annals.org/, and Epidemiology at http://www.epidem.com/). Information on the STROBE Initiative is available at www.strobe-statement.org.

For peer review only

የኢትዮጵያ የሕብረተሰብ ጤና ኢንስቲትዩት

Ethiopian Public Health Institute

አዲስ አበባ-ኢትዮጵያ Addis Ababa, Ehiopia

ስልክ-Tel: +251 11 2133499, +251 11 2751522, ፋክስ Fax: +251 11 2758634,

የመ.ሳ.ቁ - P. O. BOX: 1242/5654 e-mail: ephi@ethionet.et

www.ephi.gov.et

ቁጥር EPHI 6.13/233
Ref. No
ቀን 01-04-2021
Date

EPHI-IRB Certificate of Approval

EPHI-IRB MM No.: 076

Protocol Number: EPHI-IRB-317-2020

Protocol Title: Ethiopian Food and Nutrition Programs Baseline Survey.	
Primary Investigator	Dr Masresha Tessema
Institute:	EPHI
Study site/s	Ethiopia
Elements Reviewed (EPHI-IRB AF 01-008/02.0):	☐ Attached ✔ Not attached
Mode of Review	☐ Expedited ✔ Full Board
Decision of the meeting	✔ Approved

I. Elements approved:
1. Protocol Version No.: 03
2. Protocol Version Date: 19 Mar 2021
3. ICF Version No.: 03
4. ICF Version Date: 19 Mar 2021

II. . Obligations of the PI:
1. Should comply with the standard international & national scientific and ethical guidelines
2. All amendments and changes made in protocol and consent form needs IRB approval
3. The PI should report SAE within 48 hours of the event
4. This approval certificate is valid for only one year (specified below). The PI should Submit continuation request before expire date of approval, if project is to continue.
5. Final report/Thesis should be submitted to the IRB secretariat office (SERO) within two months following completion of the study, and Articles as soon as published

Institutional Review Board Approval Date: **16 Jan 2021**

Approval Period: **From 16 Jan 2021 to 15 Jan 2022**

Follow up report expected in:

6 months______ 9 months ______ one year ✔

EPHI-IRB Chairperson
Name &Signature Dr. Alemnesh H/mariam [illegible]
Date: 30/3/2021

EPHI Director General
Name &Signature [illegible]
Date: 31/03/21

Ebba Abate (Dr)
Director General

unicef | for every child

Ref: NUT/005/2021
Date: February 15, 2021

To: Ethiopian Public Health Institute (EPHI)

Addis Ababa

Subject: Notification of financial support for NNP II end line survey/the Ethiopian Food and Nutrition Strategy Baseline Survey

As part of the Investment Project Financing (IPF) support by World Bank and signed Memorandum of Understanding with MOH and UNICEF; the National Nutrition program II end line survey which will be served as a baseline for the National Food and Nutrition Strategy will be conducted by EPHI.

The objective of the baseline survey is to produce information on anthropometric status, dietary intakes, and micronutrient status of different population groups in Ethiopia, and assess the coverage of nutrition-sensitive and specific interventions.

UNICEF will be providing financial support ($ 917,335) to EPHI to conduct Ethiopian Food and Nutrition Strategy Baseline Survey in ten regions and two city administrations and considering of raising additional mobilization of funds for the purpose.

We look forward to continuing to work with your organization as this survey moves forward.

Sincerely Yours,

stanley chitekwe

Stanley Chitekwe,
Chief of Nutrition
UNICEF Ethiopia

Active in more than 190 countries and territories through country programmes and National Committees.
We are UNICEF, the United Nations Children's Fund.

UNICEF Ethiopia: Telephone +251 115 184000 **Fax** +251 115 511628 **Email** ethcommunication@unicef.org **Website** www.unicef.org/ethiopia

For peer review only

gain
Global Alliance for Improved Nutrition

Date: 15 February 2021
Ref. №: 01799/GAIN/ET

EPHI, IRB Office
Addis Ababa

Subject: Letter of confirmation

This letter is in response to your request on financial and technical support concerning Food and Nutrition program baseline survey. We would like to confirm that GAIN is happy to support this important initiative.

GAIN's support on Food and Nutrition program bassline survey includes the following:

- Financing field supplies for the biomarkers collection.
- Hiring a food consumption survey assistant and
- Assigning two experts from Global and local GAIN office to provide technical support.

Looking forward to a continued fruitful collaboration.

Kind regards,

Thomas Haverkort
Country Director

BETTER NUTRITION. FOR ALL.
Global Alliance for Improved Nutrition (GAIN), registered as a Foreign Charity in Ethiopia
Bole S/City, Woreda 13, H. No: New, Across Ethiopian Youth Sport Academy on the Ring Road Side Addis Ababa, Ethiopia
Telephone 251 116 610088/89 Fax 251 116 610193 e-mail: ethiopiaoffice@gainhealth.org website: www.gainhealth.org

For peer review only - http://bmjopen.bmj.com/site/about/guidelines.xhtml

BMJ Open

Ethiopia National Food and Nutrition Survey to inform the Ethiopian Food and Nutrition Strategy: A study protocol

Journal:	*BMJ Open*
Manuscript ID	bmjopen-2022-067641.R1
Article Type:	Protocol
Date Submitted by the Author:	23-Feb-2023
Complete List of Authors:	Woldeyohannes, Meseret ; Ethiopian Public Health Institute<br>Girma, Meron; Ethiopian Public Health Institute, National Information Platforms for Nutrition<br>Petros, Alemnesh; Ethiopian Public Health Institute<br>Hussen, Alemayehu ; Ethiopian Public Health Institute<br>Samuel, Aregash; Ethiopian Public Health Institute<br>Dinssa, Danial ; Ethiopian Public Health Institute, Environmental and Public Health Research<br>Challa, Feyissa; Ethiopian Public Health Institute<br>Laillou, Arnaud; UNICEF, Dakar, Senegal<br>Chitekwe, Stanley; UNICEF Ethiopia<br>Baye, Kaleab ; Addis Ababa University,<br>Noor , Ramadhani; UNICEF Ethiopia<br>Donze, Anne Sophie; UNICEF Ethiopia<br>Tollera, Getachew; Ethiopian Public Health Institute<br>Dangiso, Mesay; Ethiopian Public Health Institute<br>Tadesse, Lia; Ethiopia Ministry of Health<br>Zelalem, Meseret; Ethiopia Ministry of Health<br>Tessema, Masresha; Ethiopian Public Health Institute,
**Primary Subject Heading**:	Public health
Secondary Subject Heading:	Epidemiology
Keywords:	EPIDEMIOLOGY, NUTRITION & DIETETICS, PUBLIC HEALTH

SCHOLARONE™
Manuscripts

Ethiopia National Food and Nutrition Survey to Inform the Ethiopian Food and Nutrition Strategy: A Study Protocol

Meseret Woldeyohannes[1], Meron Girma[1], Alemnesh Petros[1], Alemayehu Hussen[1], Aregash Samuel[1], Daniel Abera Dinssa[1], Feyissa Challa[1], Arnaud Laillou[2], Stanley Chitekwe[3], Kaleab Baye[4], Ramadhani Noor[3], Anne Sophie Donze[3], Getachew Tollera[1], Mesay Hailu Dangiso[1], Lia Tadesse[5], Meseret Zelalem[5], Masresha Tessema[1*]

[1] Ethiopian Public Health Institute, Addis Ababa, Ethiopia
[2] Regional Nutrition Specialist, United Nations Children's Fund (UNICEF) Regional Office, Dakar, Senegal
[3] United Nations Children's Fund (UNICEF), Addis Ababa 12000, Ethiopia
[4] Center for Food Science and Nutrition, Addis Ababa University, Addis Ababa 12000, Ethiopia
[5] Ethiopian Ministry of Health, Addis Ababa, Ethiopia

*Correspondence: Masresha Tessema, P.O.Box 1242 Ethiopian Public Health Institute, dr.masresha.tessema@gmail.com

Running title: National Food and Nutrition Strategy Survey

For peer review only

For peer review only - http://bmjopen.bmj.com/site/about/guidelines.xhtml

ABSTRACT

Introduction Ethiopia has made significant progress in reducing malnutrition in the past two decades. Despite such improvements, a substantial segment of the country's population remains chronically undernourished and suffers from not only micronutrient deficiencies but also from increasing diet-related non-communicable diseases such as diabetics, hypertension and cancer. This survey aims to assess the anthropometric status, dietary intake and micronutrient status of Ethiopian children, women, and adolescent girls. The study will also assess the coverage of direct and indirect nutrition-related interventions, and map agricultural soil nutrients. The survey will serve as a baseline for the recently developed Ethiopian food system transformation plan and will inform the implementation of the National Food and Nutrition Strategy.

Methods and analysis A population-based cross-sectional survey, the study will collect data in the ten regions and two city administrations of Ethiopia. The study population will be women of reproductive age, children aged 0-59 months, school-aged children and adolescent girls. A total of 16,596 households will be surveyed, allowing us to generate national and regional estimates for selected indicators. A two-stage stratified cluster sampling procedure will be used to select households. In the first stage, 639 enumeration areas will be selected using probability-proportional to size allocation. In the second stage 26 eligible households will be selected within each enumeration area using systematic random selection. Primary outcomes include coverage of direct and indirect nutrition interventions, infant and young child feeding practices, food insecurity, dietary intakes, mental health of women and children, anthropometric status, micronutrient status, and soil nutrient status.

Ethics and dissemination A protocol was full reviewed and approved by the Institutional Review Board of the Ethiopian Public Health Institute (protocol no: EPHI-IRB-317-2020). The finding of this survey will be disseminated in scientific forums, national research conference, and will be submitted for publication in peer reviewed journals.

Strengths and limitations of this study

- The survey covered a large geographic area and collect data on anthropometric status, 24hr recall quantitative dietary intakes and the determination of micronutrient status in the same participants or household, while at the same time capturing data on the food system in Ethiopia.
- The study will help to improve understanding of nutritional problem across multiple facets—from agricultural soil to people to the environment in Ethiopia.
- Inherent to the cross-sectional design of the study, the findings of this study cannot be used to establish cause and effect.
- The study design prevents us from considering seasonal differences in nutritional outcomes and determinants.

For peer review only

For peer review only - http://bmjopen.bmj.com/site/about/guidelines.xhtml

INTRODUCTION

Globally, one in every three population are affected by one of more forms of malnutrition.[1] Women and children are particularly vulnerable to malnutrition due to increased physiological nutrient needs required to support fetal and child growth.[2] Nutritional deprivation during early life impairs growth and development, leading to poor school performance, reduced productivity, and loss of earnings in later life.[3] Consequently, the first 1000 days of life, from conception to the child's second year of life, was recognized as a critical window of opportunity to effectively prevent malnutrition.[3 4] Adolescence is also identified as a second window of opportunity to correct nutritional inadequacies and adversities faced in early life, but little is known about this life stage.

Despite significant progress over the past two decades, the burden of malnutrition in Ethiopia remains high.[5-7] Nationally, 37% of Ethiopian children under five years of age are stunted[7], and 22% of women of reproductive age (WRA) are chronically undernourished (Body Mass Index (BMI) < 18.5 Kg/m^2).[5] Only 14% of children under two years of age consumed the minimum number of recommended food groups.[5] Furthermore, micronutrient deficiencies co-exist with chronic energy deficiency.[8] This along with the ongoing nutrition transition, characterized by shifts in diets[9], is further complicating the nutrition landscape by increasing the prevalence of overweight and non-communicable diseases.[5] Nearly a fifth (16%) of Ethiopian adults are estimated to be hypertensive, and 3% are diabetic.[10] Therefore, addressing not only undernutrition but all forms of malnutrition is critical.

The Sustainable Development Goals (SDGs) recognize the importance of nutrition, primarily driven by the need to mitigate its detrimental consequences. Further, the 2012 World Health Assembly (WHA) identified global targets to be achieved by 2025 that aim to reduce stunting, anemia, low birth weight, and childhood obesity. These targets are used to track progress in SDG goal 2: Zero hunger.[11]Recognizing the importance of good nutrition, the Government of Ethiopia has made ending malnutrition a national priority. Ethiopia started implementing its first National Nutrition Program in 2008.[12] The second phase of this program (2011-2016) was a multisectoral program aimed at accelerating progress in reducing malnutrition.[13] Moreover, Ethiopia's first Food and Nutrition Policy was endorsed in 2018[14] , followed the National Food and Nutrition Strategy (FNS)[15] which was launched in 2021 to provide a framework for the operationalization of the policy. Acceleration of progress in the reduction of malnutrition requires the design and implementation of direct and indirect nutrition interventions that can be implemented at scale. To this end, understanding the various factors contributing to the different forms of malnutrition is critical.

Multiple factors operating at the immediate, underlying, and basic levels contribute to malnutrition.[2] Inadequate dietary intake and poor health are immediate determinants.[2] Household food security, child care practices, access to health services, and healthy environments are underlying determinants.[16] Structural and contextual factors such as economic structures, political, environmental, social and cultural factors are the basic determinants of malnutrition.[2] The contribution of these factors varies across different contexts, and target groups, but studies capturing all these factors in a single survey are scant. The lack of timely and comprehensive information on nutritional status across critical life stages and their determinants is a bottleneck that is preventing Ethiopia from designing effective interventions. Up to date and comprehensive data on the coverage of direct and indirect nutrition

For peer review only - http://bmjopen.bmj.com/site/about/guidelines.xhtml

interventions delivered across various implementing sectors of the food and nutrition strategy is not available yet. This is unfortunate as such data could inform the implementation of the food and nutrition strategy, but also serve as a baseline to which progress can be tracked against.

Therefore, this study aimed to provide the first ever comprehensive information on the nutritional status of different populations in Ethiopia to support evidence-based implementation of the national food and nutrition strategy.

Objectives of the survey

The overall goal of this study is to produce nationally and regionally representative estimates on anthropometric status, coverage of nutrition interventions, dietary intakes, and micronutrient status for children, adolescent girls, and women of reproductive age in Ethiopia.

Specific objectives include;

1. Assess the coverage of direct and indirect nutrition interventions.
2. Assess food consumption patterns and nutrients intakes of children aged 6–59 months, and women of reproductive age.
3. Assess the micronutrient status of children (vitamin A, anemia, iron, iodine and zinc), adolescent girls, and women of reproductive age (vitamin A, vitamin D, anemia, iron, iodine, zinc, folate, vitamin B_{12})
4. Assess the anthropometric status of under-5 children, school-age children (6-12 years), adolescent girls, and women of reproductive age.
5. Assess the geographical distribution of soil micronutrient status in Ethiopian agricultural soil.

METHODS AND ANALYSIS

Study design

This study is a nationally and sub-nationally (regionally) representative cross-sectional survey that will characterize dietary intake, micronutrient status, and access to nutrition-related services for different target populations. Given that soil nutrient content can influence micronutrient content of foods and hence affect nutrient intake, the soil nutrient composition will also be analyzed . The study will have four main components. The first component will assess nutrition-specific and nutrition-sensitive indicators (NSS) for all target groups (children aged 0-59 months and WRA, school-age children, adolescent girls)using semi-structured questionnaires. The second component will measure quantitative dietary intake for children aged 6-59 months and WRA (15-49 years). The third component of the survey will collect biomarker samples from all children (6-59 months), school-age children (6-12 years), adolescent girls (10-19 years), and WRA (15-49 years). The final component of the study will measure micronutrients in agricultural soils. The study data will be collected from July 2021 to December 2022.

Study setting

Ethiopia has an estimated population size of 120 million and is the second most populous country in Africa.[17] The majority of its population resides in rural areas (70%).[17] Agriculture accounts for 40% of the country's gross domestic product.[17] Children aged 15 years and younger make up 40% of the Ethiopian population in 2021.[18] Ethiopia is administratively divided into 10 regions and two city administrations. This study will be conducted in all of the regions and city administrations of the country. Figure 1 provides a geographic representation of the study areas.

For peer review only - http://bmjopen.bmj.com/site/about/guidelines.xhtml

Study participants

The target population of this study are i) women of reproductive age (WRA) aged 15-49 years ii) children aged 0-59 months iii) school age children aged 6-12 years, and iv) adolescent girls aged 10-19 years, and V) household head.

Sample size calculations

Sample size was estimated to guarantee adequate precision to generate national and regional estimates for selected indicators for each study target group. Indicators used for each target group are shown in Table 1S in supplemental materials. The required number of households and target groups was calculated using a single population proportion formula at the regional level. We used region-specific prevalence estimates for indicators, a 5% margin-of-error, a design effect of 1.5, a household response rate of 95%, and an individual response rate of 80%. The initial sample size was then adjusted for region-specific average household size and percentage of the target population from the total population. An indicator that provides the maximum number of households was used to estimate the final sample size for each region. Regional sample sizes were summed up to derive the total (national) sample size. Based on these calculations, the total sample size for the overall survey was 16,596 households (Supplemental table 2S).

For WRA, dietary and biomarker data will be collected in half of the selected households within each Enumeration Area (EA). This selection will yield a total sample size of 7,386 WRA (50% of the expected 14,772 WRA). The sample size needed to assess dietary intakes and micronutrient status of WRA was calculated using the prevalence of inadequate zinc intake, which yielded the largest sample size.[8 19]

Sampling procedures

A two-stage stratified cluster sampling procedure will be used to select households. In the first stage, 639 EAs, 257 urban and 382 rural will be selected using probability-proportional to-size allocation. We will use the 2018 Ethiopia Population and Housing Census enumeration areas sampling frame to select EAs (the Primary Sampling Units (PSUs)). The Central Statistical Agency (CSA) prepared the enumeration areas sampling frame. An EA typically contains 100-150 households. EA maps will be used to delineate the boundaries of the selected EA. In the second stage of sampling to identify eligible households, all households with the EA will be listed. A household will be eligible for selection if at least one of the study target groups are residents (de jure) or stayed at the household the night before the interview (de factor).

Twenty-six (26) eligible households will be selected within each EA using systematic random selection. All target groups will be eligible for the NSS interview in the selected households. All children aged 6-59 months will also be eligible for dietary assessment. Women residing in 13 households (Out of 26 households) who will be selected randomly will be eligible for dietary assessment. Biomarker samples will be collected for all children under 5 years of age, school-age children, and adolescents in the selected households. Similar to dietary assessment, biomarker samples will be collected for women residing in half of the selected households (figure 2).

For peer review only - http://bmjopen.bmj.com/site/about/guidelines.xhtml

Outcomes

Coverage of direct and indirect nutrition interventions

A structured questionnaire will be used to determine the coverage of direct and indirect nutrition interventions provided to children aged 6-59 months, WRA, and adolescent girls. Direct nutrition interventions included vitamin A supplementation, iron supplementation, zinc supplementation, growth monitoring and promotion, nutrition counseling services, and food fortification. Water, sanitation and hygiene, coverage of food or cash assistance program, women empowerment, and mental health will be some nutrition-sensitive indicators considered in this study (table 1). We will use standard indicator definitions proposed by the Data for Decisions to Expand Nutrition Transformation project (DataDENT) to assess coverage of nutrition programs.

Anthropometric status

Using standardized procedures, anthropometric measurements, including weight, height/length, and mid-upper arm circumference, will be taken for all study target populations.[20] Anthropometric indices (weight-for-height z-scores, length/height-for-age z-scores, weight-for-age z-scores, BMI-for-age z-scores) will be calculated using the WHO 2006 child growth standards and the WHO 2007 child growth reference data. Stunting (length/height-for-age z-scores below -2 SD), wasting (weight-for-height z-scores below -2 SD), underweight (weight-for-age z-scores below -2 SD), thinness (BMI-for-age z-scores below -2 SD) and BMI will be the primary anthropometric outcomes of interest.

Infant and young child feeding practices

Infant and young child feeding practices will be assessed using the new World Health Organization (WHO) and United Nations Children's Fund (UNICEF) recommended 17 indicators to evaluate IYCF practices.[21]

Food insecurity

The Food Insecurity Experience Scale (FIES) will be used to assess household food security.[22] The FIES consists of eight questions that assess household experience related to adequate food access. Experience questions range from worrying about getting enough food to not eating for a whole day.

In addition to these outcome indicators, information on the sociodemographic characteristic of households, child health, maternal health, employment status, and household agricultural practices will be collected using structured questionnaires.

Mental health of women

Common mental health disorders will be assessed using the WHO Self-reporting questionnaire which consists of 20 questions. Women will be classified as having a common mental health disorder if row score was greater or equal to 6 out of 20.[23]

Assessment of dietary intakes of children and WRA

We will measure dietary intake for children aged 6-59 months and WRA. A one-day quantitative multiple-pass 24-hour recall will be conducted to assess dietary intakes. The interactive multiple-pass 24-hour recall interview consists of four steps designed to enhance memory.[24] All days of the week will be proportionately represented during the dietary survey to account for the day of the week effects on food

For peer review only - http://bmjopen.bmj.com/site/about/guidelines.xhtml

intake. To account for the day-to-day variability of dietary intake within individuals, a second non-consecutive day 24-hr recall (repeat) will be collected (within 2 to 10 days of the first recall) on a randomly selected sub-sample of WRA and children. The number of repeats needed is determined by allocating for each region 50 repeats, which is then multiplied by a design effect of 1.5 and a 10% non-response rate. The number of repeats was rounded up to 1244 recalls for each target group to ensure that the minimum number of repeats (n=83) needed from each region would be collected. Detailed non-standard recipe ingredient data will be collected for all mixed dishes that were prepared at home.

We will use 15 food groups to assess dietary intakes of women (15-49 months) and children aged 24-59 months. These food groups were: 1) Cereals and their products, 2) Starchy Roots and tubers, and their products, 3) Pulses, and their products, 4) Vegetables and their products, 5) Fruits and their products, 6) Meat, and poultry their products, 7) Eggs and their products, 8) Fish, shellfish and their products, 9) Milk and milk products, 10) Fats and oils, 11) Nuts and seeds, 12) Sugar and sweetened products, 13) Beverages, 14) Spices and condiments, and 15) Miscellaneous. For children aged 6-23 months we will use the updated WHO, UNICEF food groups: 1) Breastmilk, 2) Grains, roots and tubers, 3) Pulses, nuts and seeds, 4) Dairy products, 5) Flesh foods (meats, fish, poultry, organ meats), 6) Eggs 7) Vitamin-A rich fruits and vegetables, and 8) other fruits and vegetables.

Dietary assessment pre-survey work: We carried out pre-survey work to aid dietary data collection following recommendation set by the Intake: Center for Dietary Assessment.[25 26] An initial step was developing a food and ingredient list containing a comprehensive list of food items, mixed dishes, and ingredients expected to be consumed by the study target groups. The food list was generated using data from the first 2011 Ethiopian National Food Consumption Survey.[19] Other common foods consumed across the regions in Ethiopia were derived from the 2016 Household Income and Expenditure Surveys,[27] the Ethiopian Food Composition Tables, and dietary intake data from other recent dietary assessment surveys conducted by the Ethiopian Public Health Institute (EPHI). Portion size estimation methods suitable for large-scale studies were pre-selected for use in the survey following Intake recommendations.[28] The selected methods were direct measurement of actual foods consumed, standard unit: size and number, proxy measurement using play dough, water, rice, and maize flour, and finally using food price to estimate the amount of food consumed. Portion size estimation methods were assigned for all foods included in the food list.

Table 1. Nutrition-direct and nutrition-indirect interventions coverage

No	Indicator	Target population
	**Nutrition indirect intervention coverage**	
	Child interventions	
1.	Children received iron tablets/syrup in the last 12 months	Children aged 6-59 months
2.	Children received vitamin A supplements in past 6 months	Children aged 6-59 months
3.	Children received deworming tablets in the past 6 months	Children aged 24-59 months
4.	All 8 basic vaccinations: one dose of BCG, three doses of DPT, three doses of the polio vaccine,	Children aged 9-59 months

For peer review only - http://bmjopen.bmj.com/site/about/guidelines.xhtml

	and one dose of the measles vaccine	
5.	No Vaccination	Children aged 0-59 months
	Growth monitoring	
6.	Weight measured in the last 3 months	Children aged 0-23 months
7.	Height measured in the last 3 months (Optional)	Children aged 0-23 months
8.	MUAC measured in the last 3 months (Optional)	Children aged 0-23 months
	Infant and young child feeding (IYCF) counselling	
9.	Mothers with children 6-23 months received any IYCF counseling	Children aged 6-23 months
10.	Mothers with children 6-23 months received age-appropriate IYCF counseling	Children aged 6-23 months
	Early breast-feeding counseling	
11.	Women *received breastfeeding counseling with observation during the first two days after birth*	Women aged 15-49 years with a live birth in the past 5 years for the most recent birth
12.	Women *received breastfeeding counseling during the first month after birth*	Women aged 15-49 years with a live birth in the past 5 years for the most recent birth
	Coverage of Nutritional Interventions during pregnancy/antenatal Care (ANC)	
13.	Percentage of women who had 4 or more ANC visits for the most recent birth	Women aged 15-49 years with a birth in the last 5 years
14.	Percentage of women who received counseling about healthy eating during pregnancy	Women aged 15-49 years who received antenatal care for their most recent birth
15.	Percentage of women whose weight gain was monitored during pregnancy	Women aged 15-49 years who received antenatal care for their most recent birth
16.	Women received food or cash assistance during pregnancy	Women aged 15-49 years with a birth in the last 5 years
17.	Women took 90+ iron/folate tablets during pregnancy	Women aged 15-49 years with a live birth in the past 5 years for the most recent birth
18.	Women received deworming tablets during pregnancy	Women aged 15-49 years with a live birth in the past 5 years for the most recent birth
	**Nutrition indirect intervention coverage**	
19.	Basic water services	Household
20.	Basic hygiene services	Household
21.	Basic Sanitation services	Household
22.	Food insecurity (not a service hence no coverage)	Household
23.	Women received food or cash assistance during pregnancy	Women aged 15-49 years with a live birth in the past 5 years for the most recent birth
24.	Basic water services	Household
25.	Basic hygiene services	Household
26.	Basic Sanitation services	Household
27.	Presence of common mental health disorders in	Women aged 15-49 years

For peer review only - http://bmjopen.bmj.com/site/about/guidelines.xhtml

	the past month	
28.	Women empowerment	Women aged 15-49 years
29.	Livestock ownership	Household
30	Agricultural productivity by food group	Household

Assessment of micronutrient status:

Blood specimens will be collected from the study population to determine serum retinol, ferritin, soluble transferrin receptor (sTfR), zinc, folate, vitamin B12, red blood cell (RBC) folate and 25-hydroxyvitamin D. Additionally, markers of inflammation, alpha(1)-acid glycoprotein (AGP), high-sensitivity C-reactive protein (hsCRP) will also be measured. We will also analyze parasites from stool specimens. All laboratory analyses will be performed at the EPHI Clinical chemistry, and Food Science and Nutrition Laboratories. Both laboratories participate in an external quality assessment scheme and are accredited by the Ethiopian National Accreditation Office (ENAO). Collection, storage, and analytical procedures for blood, urine, stool, and salt samples are described below. The details of each biomarker analysis are described in Supplemental Material 1-11.

Blood sample collection and analysis: Venous blood samples (5-7 mL) will be collected using vacutainer tubes following standard operating procedures.[29] Trace mineral-free vacutainer tubes will be used to collect blood for trace metal analysis. After collection, blood samples will be allowed to clot for 30 minutes in cold boxes (<8 °c). Samples will then be centrifuged at 3000 rpm (revolution per minute) for 10 minutes. The separated serum will be aliquoted and stored in -20°c portable freezers in the field. Samples will then be transported to EPHI and stored at -80°c until analysis. Hemoglobin will be measured in the field using Hemocue® (Hb 301, Hemocue AB, Angelholm, Sweden)[30].[30] If the hemoglobin values are below WHO cutoff point(11g/dl), the phlebotomist will send whole blood samples to the EPHI laboratory to identify hemoglobinopathies using electrophoresis method.[31] Malaria test will be conducted on-site using Bioline™ Malaria Ag P.f rapid diagnostic test kits (RDT) for P. falciparum and P. vivax).[32] Serum soluble transferrin receptor (sTfR), AGP, hsCRP, folate, red blood cell (RBC) folate, vitamin B_{12}, and ferritin will be measured using Cobas 6000 analyzer (Roche Diagnostics GmbH, Mannheim, Germany). Serum retinol will be measured using high-performance liquid chromatography (HPLC) method[32], and serum zinc and selenium will be measured using a microwave plasma atomic emission spectrometers (MP-AES) analyzer.

***Stool and urine sample collection and analysis**:* Stool samples will be collected using stool cups and stored in 10 % formalin to preserve the parasite until analysis.[33] A portion of each stool sample will be used to detect direct ova, larvae and cysts of intestine parasites using formal ether concentration technique.[34]Urine samples will be collected from WRA and school-age children using 60 ml urine cup containers. Samples will be stored at -20°c. Urinary iodine excretion will be assessed by Sandell Kolthoff reaction at EPHI Laboratory using Shimadzu 1800 UV-Vis spectroscopy.[35]

***Salt collection and analysis*:** Salt samples will be collected from households with WRA for whom dietary data will be collected. At least 25 grams (one coffee cup) of salt will be collected to determine iodine content using the iodometric titration method.[36]

For peer review only - http://bmjopen.bmj.com/site/about/guidelines.xhtml

Assessment of nutrients in the soil

Soil samples will be collected from three households in each EA. Zig-zag or cross sampling method will be used to collect 10 to 20 subsamples (0-30 cm depth) constituting one composite sample. Subsamples will be collected at a separation distance of five meters. After thoroughly mixing composite samples, 1 kg soil sample will be transferred to polyethylene bags. The collected soil samples will be air-dried in wooden trays and disaggregated using a ceramic mortar and pestle (soil grinder) at the EPHI soil laboratory. Samples will then pass through a 6 mm sieve of stainless-steel screens to remove debris and homogenize the soil sample. The sieved fraction will be further pulverized to pass through a 1 mm sieve for the micronutrient analysis. Soil zinc, iron, copper, and manganese will be determined following standard procedures.[37] Micronutrient content will be determined using inductively coupled plasma-optical emission spectroscopy (ICP-OES) after extraction with diethylene triamine penta acetic acid (DPTA). Additional variables that affect the mobility of micronutrients in the soil and their uptake into crops will also be measured. These variables include soil reaction (pH), electrical conductivity, organic matter, total nitrogen, and soil organic carbon content. Data collectors will also record topography, slope, cropping history, type, and fertilizer application information. Table 2 provides a summary of procedures for each of the four components of the survey by study target groups.

For peer review only - http://bmjopen.bmj.com/site/about/guidelines.xhtml

Table 2. Summary of data collection procedures for each of the four components of the survey

	Child 0-5 months	Child 6-23 months	Child 24-59 months	School children 6-12 years	Adolescent girls 10-19 years	WRA 15-49 years	Household
**Nutrition direct and nutrition indirect interventions indicators**							
Infant and young child feeding practices	X	X					
Nutritional information for adolescent girls					X		
Food insecurity							X
Water, sanitation and hygiene practices							X
Coverage of food fortification							X
Agricultural practices							X
Mental health						X	
Anthropometric status	X	X	X		X	X	
**Dietary assessment**							
24-hr recall quantitative dietary intake		X	X			X	
**Assessment of biomarker status**							
Blood sample		X	X	X	X	X	
Urine sample				X		X	
Stool sample		X	X	X	X	X	
Salt sample collection							X
**Assessment of micronutrients in the Soil**							
Soil micronutrient assessment							X

For peer review only

For peer review only - http://bmjopen.bmj.com/site/about/guidelines.xhtml

Data quality assurance and analysis

Training of trainers on components of the survey was held before training the data collectors and supervisors. After fifteen days of training on methodological procedures, questionnaires and quality assurance, the questionnaires were tested in a pilot group (in EAs not included in the actual survey), and adapted based on the received feedback from the survey team. The questionnaires (including the food list) were translated into local languages (Amharic, Oromifa, Tigrigna, Somali, and Afar) and back-translated to English to ensure the quality of the translation. The data collectors' measurements were standardized to ensure that the inter-observer variability was within tolerable limits. Supervisors received additional training on teamwork and on monitoring and supervising the data collection process. All data collection tools are programmed using open-source software (ODK) (Supplemental Material 12). Data quality checks were included during ODK programming to prevent data recording errors. These include restricted responses, filter insert choices, skip patterns, and defaults. During data collection daily data tracking forms will be completed to track completed surveys for each study components to prevent missing data. Before the start of the survey high frequency checks were identified and error tracking forms were designed to track data quality in real-time. These checks included completeness checks, target group tracking, and duplicate ID check. Random field-supervision visits will also be made to check data quality. Every day, collected data will be sent to the EPHI central server and imported into statistical software programs as comma-separated values (CSV) files. For laboratory analysis, a quality control chart will be used to ensure the internal and external quality control materials are in the acceptable range.

The primary data analysis will focus on computing frequencies and percentages for categorical variables and summary statistics (like means, medians SD, IQR) for summarizing continuous variables. Sample weights will be constructed based on the selection probabilities of EAs, eligible households, and non-response rates. All analyses will also be adjusted for the survey design. Additional subgroup analysis will be computed for variables with adequate sample sizes for each category. The Biomarkers Reflecting Inflammation and Nutrition Determinants of Anemia (BRINDA) Working Group's regression correction approach will be used to account for inflammation in the study of all micronutrients status using the biomarkers C-reactive protein (CRP) and AGP. Geostatistical analyses will be employed to determine the spatial patterns of micronutrient distribution in the soil and blood samples. The wealth index will be constructed using principal component analysis (PCA).[38] The Rasch model will be used to construct the Food Insecurity Experience Scale (FIES). [22] All analysis will be done using STATA 16 and ArcGIS/QGIS. Anthropometric indices will be calculated using the WHO Anthro software for under five children and WHO AnthroPlus software for adolescent.

Patient and public involvement statement

None

Ethics and dissemination

The study protocol is approved by the Institutional Review Board of the Ethiopian Public Health Institute (protocol no: EPHI-IRB-317-2020). Written informed consent will be obtained from each respondent and participants may withdraw at any time (supplemental Material 13).Confidentiality of all

For peer review only - http://bmjopen.bmj.com/site/about/guidelines.xhtml

collected data will be given high priority during each stage of data handling. Individual names and personal information of respondents will be kept confidential and data sets will be kept anonymous for analysis.

The study's findings will be disseminated through several communication channels, including stakeholder workshops, various local and International conferences and technical report. Additionally, the findings will be submitted as special issue to peer-reviewed journals.

DISCUSSION

This comprehensive, nationally representative survey will for the first time characterize simultaneously the dietary intake and micronutrient status of Ethiopian children, adolescent girls and WRA. Besides, the study assesses key drivers of malnutrition including soil nutrient composition, as well as coverage of direct and indirect nutrition interventions. The survey will provide key insights informing the implementation of Ethiopia's National Food and Nutrition Strategy.

High-quality and timely data is critical to assess the burden of nutritional problems, identify vulnerable populations and priority actions, track the implementation of nutrition programs, and assess impact.[39 40] Ethiopia conducted its first-ever food consumption survey in 2011.[19]and its micronutrient survey in 2015.[41] Both surveys were collected at different times, which made it difficult to link the two surveys. Besides, the causes and solutions of malnutrition are complex and multisectoral; hence, requiring data on multiple indicators from various sectors spanning from soil nutrient, diets, micronutrient status, to access and exposure to direct and indirect nutrition interventions. In this regard, this survey is uniquely positioned to integrate data from multiple domains to support evidence-based decision making for improved diets, nutrition, and overall wellbeing.

This study will allow us to evaluate progress relative to the last food consumption and micronutrient surveys, but more importantly will serve as a baseline to which future progress related to the implementation of the food and nutrition strategy will be evaluated. Furthermore, the current survey will also serve as a baseline for the Ethiopian Food System Transformation Plan by capturing majority indicators used for monitoring food systems related progress. Thus, filling information gaps that could have impeded successful implementation of the National Food and Nutrition Strategy (FNS). By establishing 13 strategic objectives, the FNS is intended to be aligned with the strategic directions of the Food and Nutrition Policy (FNP). Each strategy direction includes initiatives, actions, and key performance indicators, as well as leading and collaborating sectors. The key performance indicators should be evaluated to determine the progress of each implementing sector's achievement. The current survey will provide up to date national and subnational information on the current food and nutrition situation in Ethiopia for different target populations as well as provide comprehensive list of indicators that are pertinent to the implementation of the policy.[39] In addition, this study will provide information on context-specific determinants for prioritizing direct and indirect actions that can be implemented across sectors taking into account the specific needs of different target populations.

Additionally, effective multisectoral interventions that address the immediate and underlying determinants of malnutrition must be implemented in order to accelerate the reduction of malnutrition in its all form.[39] These interventions need to address context-specific determinants to reduce

For peer review only - http://bmjopen.bmj.com/site/about/guidelines.xhtml

malnutrition effectively.[39] The lack of timely and disaggregated information on the determinants of malnutrition is a bottleneck to preventing malnutrition, particularly among the most vulnerable target populations. This study will also provide information on the coverage and quality of interventions which can be used to contextualize National Food and Nutrition Strategy monitoring frameworks, monitor implementation and track progress towards global and local targets.

Although this study will provide regionally and nationally representative estimates for key indicators and critical life stages, it has several limitations. Inherent to the cross-sectional design of the study, the findings of this study cannot be used to establish cause and effect. Additionally, the design prevents us from considering seasonal differences in nutritional outcomes and determinants. This study also relies on self-reported data, which is subject to recall bias. Notwithstanding the above-mentioned limitations, this study is uniquely designed to combine the assessment of anthropometric status, 24hr recall quantitative dietary intakes and the determination of micronutrient status in the same participants, while at the same time capturing data on the food system. Additionally, the study will be evaluating micronutrients in the agricultural soil, which will expand our understanding of factors that influence nutrition. To the best of our knowledge, this will be among -if not-the first study to simultaneously collect these variables from the same household. This could contribute to a better understanding of nutritional problem across multiple facets—from soil to people to the environment. In the past, nutrition programs implemented in Ethiopia have relied on information provided from small-scale studies and population-based surveys such as the Ethiopia Demographic and Health Surveys.[5-7 42 43] Although these data sources provide some information to track progress and tailor interventions, they only provide data on a limited number of nutrition indicators and do not measure dietary intakes and assess biomarker status. This study will fill these data gaps by providing information on comprehensive indicators that show the burden and spatial distribution of micronutrient deficiencies and shifts of dietary patterns. Additionally, this study will provide information on emerging determinants such as mental health and intake of nutrients such as folate and B_{12} that have not been included in previous studies. Finally, the inclusion of adolescent girls, and school-age children, will provide vital information on nutritional indicators for these target groups, which are often not included in other nationally representative surveys. This survey will also provide information on the coverage of direct interventions implemented in the health sectors and indirect interventions implemented in the agriculture, WASH, education and social protection sectors for whom scant data exists. Hence, this study will provide valuable information that will guide the implementation of strategic actions for the reduction of malnutrition in Ethiopia.

For peer review only - http://bmjopen.bmj.com/site/about/guidelines.xhtml

Acknowledgments:

Contributions: MT, AL, SC, MW, AP, AS, and MG conceived the study and drafted the original protocol. All authors participated in refining of the protocol. AH, MW, MG and MT played a major role in the statistical consideration. DAD, FC, MG, RN, and ASD helped to write the draft protocol and made a critical contribution to the content. KB, AL, GT, MHD, LT and MZ supervised manuscript preparation. All authors took responsibility for reviewing, final editing and approval of the manuscript.

Funding: This work was supported by Power of Nutrition, BMGF and World Bank through UNICEF; Nutrition International, and Global Alliance for Improved Nutrition.

Competing interests: The authors declare that they have no conflicts of interest.

Patient consent for publication: Not required

For peer review only

References

1. Fanzo J, Hawkes C, Udomkesmalee E, et al. 2018 Global Nutrition Report. 2019
2. Black RE, Victora CG, Walker SP, et al. Maternal and child undernutrition and overweight in low-income and middle-income countries. *The Lancet* 2013;382(9890):427-51. doi: 10.1016/s0140-6736(13)60937-x
3. Martorell R. Improved nutrition in the first 1000 days and adult human capital and health. *Am J Hum Biol* 2017;29(2) doi: 10.1002/ajhb.22952 [published Online First: 2017/01/25]
4. United Nations. Transforming our world: The 2030 agenda for sustainable development, 2015.
5. Central Statistical Agency [Ethiopia] and ICF. Ethiopia demographic and health survey 2016. Addis Ababa, Ethiopia and Calverton, Maryland, USA: CSA and ICF, 2016.
6. Central Statistical Agency [Ethiopia] and ORC Macro. Ethiopia demographic and health survey 2000. Addis Ababa, Ethiopia and Calverton, Maryland, USA: CSA and ORC Macro, 2001.
7. Ethiopian Public Health Institute (EPHI) [Ethiopia] and ICF. Ethiopia Mini Demographic and Health Survey 2019: Key Indicators. Rockville, Maryland, USA: EPHI and ICF, 2019.
8. Ethiopian Public Health Institute. Ethiopian national micronutrient survey report Ethiopian Public Health Institute, 2016.
9. Hassen I, Dereje M, Minten B, et al. Diet transformation in Africa: the case of Ethiopia. *Agricultural Economics* 2017
10. Ethiopian Public Health Institute, Federal Ministery of Health, World Health Organization. Ethiopia STEPS report in risk factors for non-communicable diaseses and prevalence of selected NCDs Addis Ababa,Ethiopi: Ethiopian Public Health Institute 2016.
11. World Health Organization. Global nutrition monitoring framework: Operational guidance for tracking progress in meeting targets for 2025. Geneva: WHO, 2017.
12. Federal Democratic Republic of Ethiopia. National Nutrtion Program: 2013-2015. Addis Ababa,Ethiopia, 2013.
13. Federal Democratic Republic of Ethiopia. National Nutrition Program: 2016-2020. Addis Ababa,Ethiopia., 2016.
14. Federal Democratic Republic of Ethiopia. Food and Nutrition Policy, 2018.
15. Federal Democratic Republic of Ethiopia. National Food and Nutrition Strategy (2021-2023), 2021.
16. Reinhardt K, Fanzo J. Addressing Chronic Malnutrition through Multi-Sectoral, Sustainable Approaches: A Review of the Causes and Consequences. *Front Nutr* 2014;1:13. doi: 10.3389/fnut.2014.00013 [published Online First: 2014/01/01]
17. Bank W. Population projections Ethiopia 2021 [
18. Bardosh KL, Hussein JW, Sadik EA, et al. Chicken eggs, childhood stunting and environmental hygiene: an ethnographic study from the Campylobacter genomics and environmental enteric dysfunction (CAGED) project in Ethiopia. *One Health Outlook* 2020;2(1) doi: 10.1186/s42522-020-00012-9
19. Ethiopian Public Health Institute. Ethiopian national food consumption survey Ethiopian Public Health Institute, 2013.
20. UNICEF Wa. Recommendations for data collection, analysis and reporting on anthropometric indicators in children under 5 years old. : World Health Organization and the United Nations Children's Fund (UNICEF), 2019.
21. WHO, UNICEF. Indicators for assessing infant and young child feeding practices: definitions and measurement methods. Geneva: World Health Organization and the United Nations Children's Fund (UNICEF), 2021.
22. Ville AS, Tsun Po JY, Sen A, et al. Food security and the Food Insecurity Experience Scale (FIES): ensuring progress by 2030. *Food Security* 2019;11(3):483-91. doi: 10.1007/s12571-019-00936-9

For peer review only - http://bmjopen.bmj.com/site/about/guidelines.xhtml

23. World Health Organization. Self-reported mental health questionnaires1994.
24. Gibson RS, Ferguson EL. An interactive 24-hour recall for assessing the adequacy of iron and zinc intakes in developing countries HarvestPlus 2008.
25. Vossenaar M, Arimond M, Deitchler M, et al. An overview of the main pre-survey tasks required for large-scale quantitative 24-Hour recall dietary surveys in low- and middle-income countries. Washington, DC: Intake – Center for Dietary Assessment/FHI Solutions., 2020.
26. Intake – Center for Dietary Assessment. Dietary Survey Protocol Template: An Outline to Assist with the Development of a Protocol for a Quantitative 24-Hour Dietary Recall Survey in a Low- or Middle-Income Country Washington, DC: Intake – Center for Dietary Assessment/FHI Solutions., 2020.
27. Central Statistical Agency[Ethiopia]. Household Consumption and Expenditure (HCE) Survey 2015/16: Analytical Report. Addis Ababa: CSA, 2016.
28. Vossenaar M, Lubowa A, Hotz C, et al. Considerations for the Selection of Portion Size Estimation Methods for Use in Quantitative 24-Hour Dietary Recall Surveys in Low- and Middle-Income Countries. Washington, DC: Intake – Center for Dietary Assessment/FHI Solutions, 2020.
29. Organization WH. Micronutrient survey manual. 2020
30. Jaggernath M, Naicker R, Madurai S, et al. Diagnostic Accuracy of the HemoCue Hb 301, STAT-Site MHgb and URIT-12 Point-of-Care Hemoglobin Meters in a Central Laboratory and a Community Based Clinic in Durban, South Africa. *PloS one* 2016;11(4):e0152184. doi: 10.1371/journal.pone.0152184 [published Online First: 2016/04/06]
31. Wieringa FT, Dahl M, Chamnan C, et al. The High Prevalence of Anemia in Cambodian Children and Women Cannot Be Satisfactorily Explained by Nutritional Deficiencies or Hemoglobin Disorders. *Nutrients* 2016;8(6) doi: 10.3390/nu8060348 [published Online First: 2016/06/25]
32. Lee GC, Jeon ES, Le DT, et al. Development and evaluation of a rapid diagnostic test for Plasmodium falciparum, P. vivax, and mixed-species malaria antigens. *Am J Trop Med Hyg* 2011;85(6):989-93. doi: 10.4269/ajtmh.2011.11-0265 [published Online First: 2011/12/07]
33. Demeke G, Fenta A, Dilnessa T. Evaluation of Wet Mount and Concentration Techniques of Stool Examination for Intestinal Parasites Identification at Debre Markos Comprehensive Specialized Hospital, Ethiopia. *Infect Drug Resist* 2021;14:1357-62. doi: 10.2147/IDR.S307683 [published Online First: 2021/04/17]
34. Kache R, Phasuk N, Viriyavejakul P, et al. Prevalence of soil-transmitted helminth infections and associated risk factors among elderly individuals living in rural areas of southern Thailand. *BMC public health* 2020;20(1):1882. doi: 10.1186/s12889-020-09986-7 [published Online First: 2020/12/09]
35. Machado A, Lima L, Mesquita RBR, et al. Improvement of the Sandell-Kolthoff reaction method (ammonium persulfate digestion) for the determination of iodine in urine samples. *Clin Chem Lab Med* 2017;55(9):e206-e08. doi: 10.1515/cclm-2016-1094 [published Online First: 2017/03/18]
36. Jooste PL, Strydom E. Methods for determination of iodine in urine and salt. *Best Pract Res Clin Endocrinol Metab* 2010;24(1):77-88. doi: 10.1016/j.beem.2009.08.006 [published Online First: 2010/02/23]
37. Lindsay WL, Norvell W. Development of a DTPA soil test for zinc, iron, manganese, and copper. *Soil Sci Soc Am J* 1978;42(3):421-28.
38. Karamizadeh S, Abdullah SM, Manaf AA, et al. An overview of principal component analysis. *Journal of Signal and Information Processing* 2020;4
39. Heidkamp RA, Piwoz E, Gillespie S, et al. Mobilising evidence, data, and resources to achieve global maternal and child undernutrition targets and the Sustainable Development Goals: an agenda for action. *The Lancet* 2021 doi: 10.1016/s0140-6736(21)00568-7

For peer review only

For peer review only - http://bmjopen.bmj.com/site/about/guidelines.xhtml

40. Victora CG, Christian P, Vidaletti LP, et al. Revisiting maternal and child undernutrition in low-income and middle-income countries: variable progress towards an unfinished agenda. *The Lancet* 2021 doi: 10.1016/s0140-6736(21)00394-9
41. Institute EPH. Ethiopian national micronutreint Survey. Addis Ababa, Ethiopia Ethiopian Public Health Institute 2016.
42. Central Statistical Agency [Ethiopia] and ICF. Ethiopia demographic and health survey 2011. Addis Ababa, Ethiopia and Calverton, Maryland, USA: CSA and ICF, 2011.
43. Central Statistical Agency [Ethiopia] and ORC Macro. Ethiopia demographic and health survey 2005. Addis Ababa, Ethiopia and Calverton, Maryland, USA: CSA and ORC Macro, 2006.

For peer review only

For peer review only - http://bmjopen.bmj.com/site/about/guidelines.xhtml

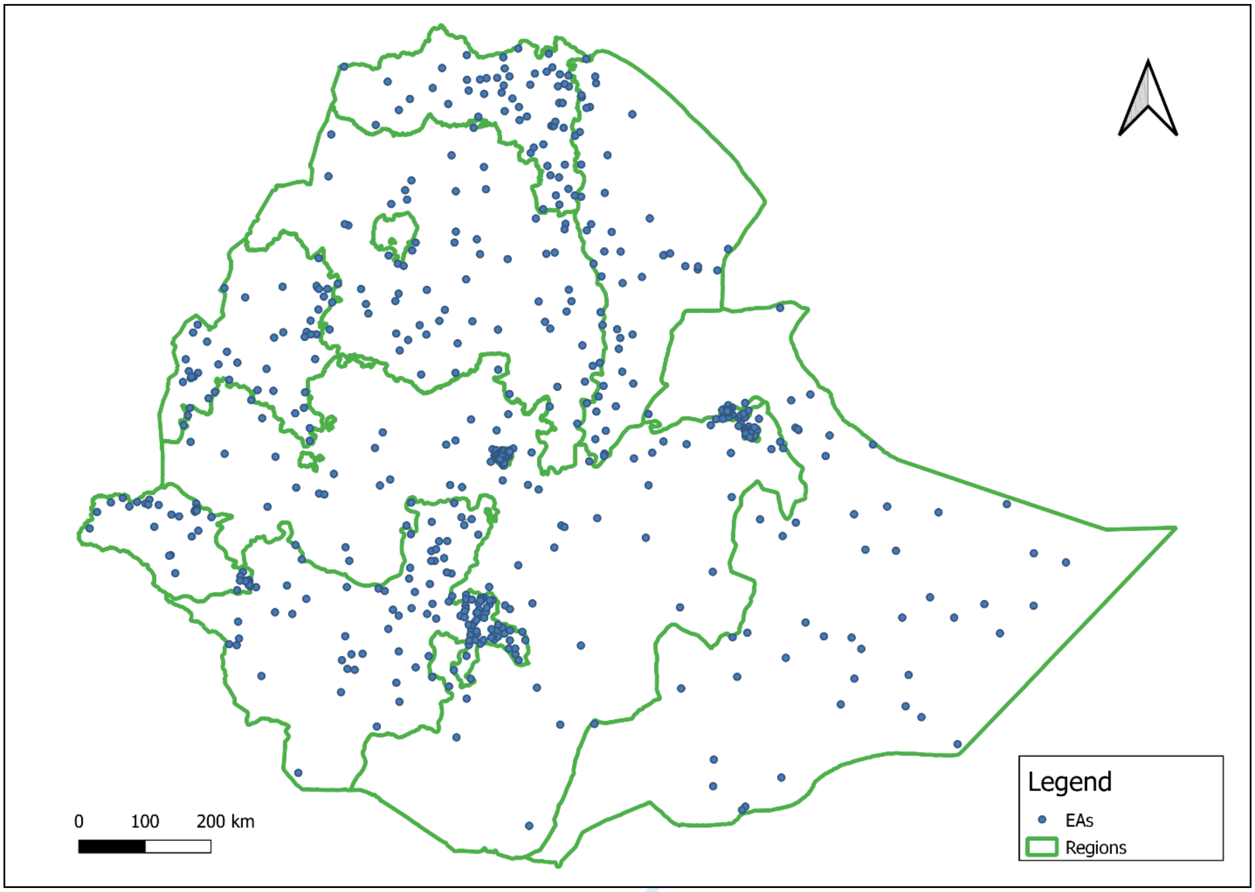

Figure 1. Map showing study enumeration areas across regions

view only

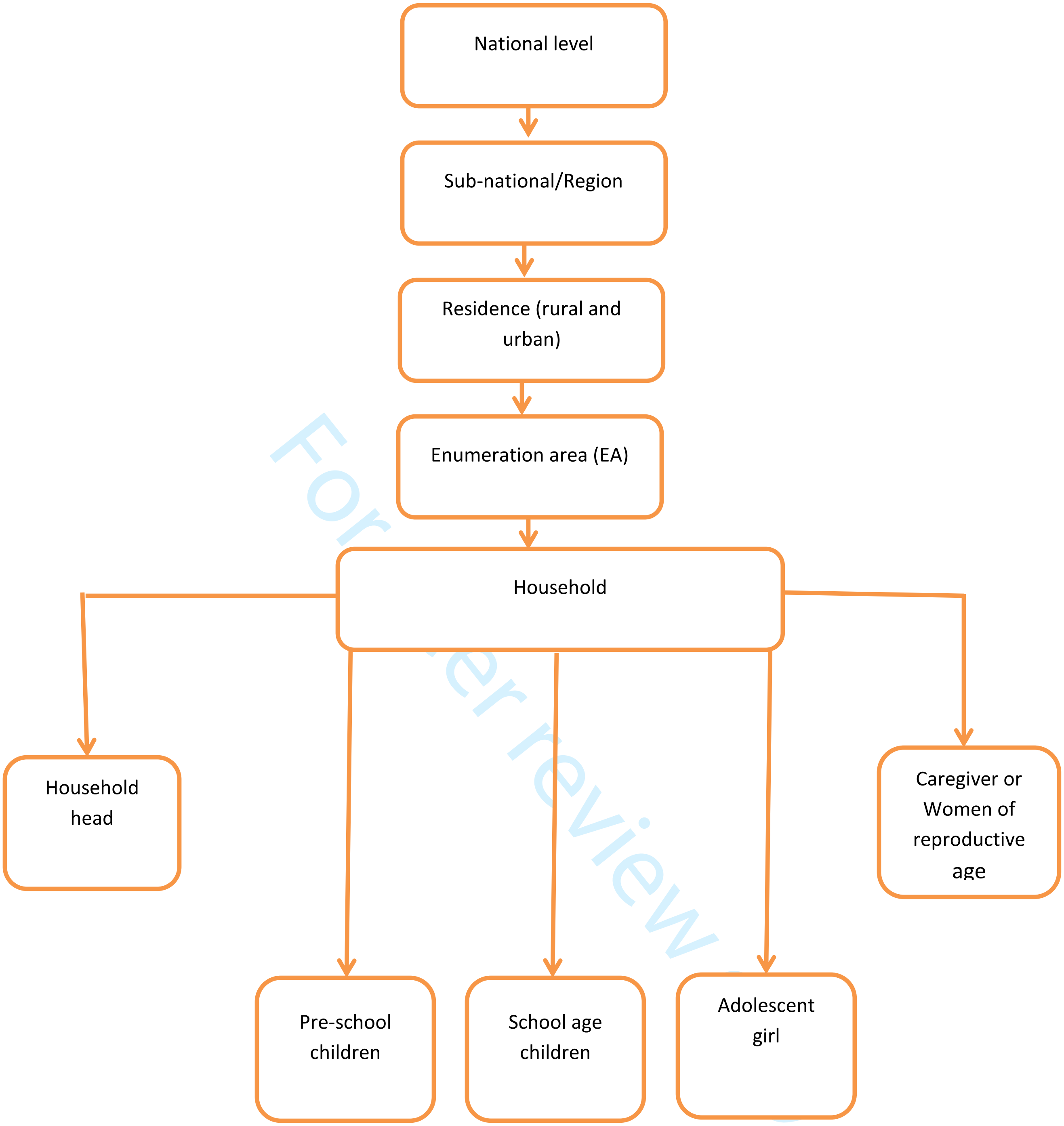

Figure 2. Sampling frame for Ethiopia National Food and Nutrition Survey to Inform the Ethiopian Food and Nutrition Strategy

Table 1S. Key indicators used to estimate sample size for each target group

Target Group	Key indicators used to estimate sample size
Children under 5 years of age (0-59 months)	Vitamin A deficiency<br>Total goiter prevalence<br>Stunting<br>Any anemia<br>Zinc deficiency<br>Prevalence of inadequate intake of zinc
Women of reproductive age (15-49 years)	Vitamin A deficiency<br>Total goiter prevalence<br>Any anemia<br>Zinc deficiency<br>RBC folate deficiency<br>Serum folate deficiency<br>Vitamin B_{12} deficiency<br>Iodized salt coverage<br>Prevalence of inadequate intake of iron<br>Prevalence of inadequate intake of zinc<br>Prevalence of inadequate intake vitamin A
School age children (6 to 12 years)	Vitamin A deficiency<br>Total goiter prevalence<br>Any anemia<br>Iodine deficiency<br>Zinc deficiency
Adolescent girls (10 to 19 years)	Any anemia

For peer review only

Table 2S. Sample size determination and allocation

Region	Indicator used to estimate sample size	Number of EA	Number of HH	Expected number pre-school children (0-59 months)	Expected number of WRA (15-49 years)	Expected number school age children (6-12 years)	Expected number of adolescent girls (10-19 years)	Total population (2019 year projection)
Tigray	Any anemia	55	1,432	590	1,236	753	516	5,443,095
Afar	Stunting	51	1,328	539	1,096	695	406	1,901,863
Amhara	Stunting	61	1,585	619	1,253	843	531	21,842,548
Oromia	IDD	62	1,622	891	1,539	1,111	739	37,267,225
Somali	IDD	55	1,424	855	1,268	1,000	492	6,050,851
Benishangul-Gumuz	Stunting	49	1,282	555	1,127	732	475	1,126,656
SNNPR	Any anemia	59	1,528	818	1,492	1,000	692	15,763,484
Gambela	Any anemia	47	1,211	428	1,018	568	373	463,203
Harari	Any anemia	45	1,164	375	978	499	348	257,362
Addis Ababa	IDD/ TGR	54	1,411	413	1,274	405	262	3,685,684
Dire Dawa	Stunting	47	1,215	382	1,128	480	360	492,819
Sidama	Any anemia	54	1,395	747	1,363	914	632	4,322,685
Total sample size		639	16,596	7,213	14,772	9,001	5,824	**98,617,475**

For peer review only

Name of institute	Doc. number:	Version No: 3
**ETHIOPIAN PUBLIC HEALTH INSTITUTE**	EPHI/ TL/ FNL/TM/7.2.11	Copy no_____
		Page 1 of 7
Document Title: **DETERMIANTION OF IODINE FROM SALT SAMPLE**	Effective date: November 20, 2020	

1. Purpose

This procedure used to determine the amount of iodine content in the form of potassium iodate (KIO_3) by iodometric titration from iodized salt.

2. Abbreviations:

**g**	gram	**KI**	Potassium Iodide
**ppm**	Parts Per million	**H_2SO_4**	sulfuric acid
**M**	Molarity	**KIO_3**	potassium iodate
**ml**	mili liter	**IDD**	Iodine deficiency disorder
**N**	Normality	**$Na_2S_2O_3.5H_2O$**	sodium thiosulfate penta hydrate

3. Principle:

Iodine released from potassium iodate by the action of sulphuric acid and the released iodine trapped with potassium iodide and titrate with sodium thiosulphate.

4. Material and methods

4.1 Reagents

- 0.005M $Na_2S_2O_3.5H_2O$
- 2N H_2SO_4
- 10% KI
- 1% Starch

4.2 Reagents preparation:

- **1%starch:** Dissolve 1 g of soluble starch in 100ml boiled distilled water heat the solution till starch dissolve completely.
- **10% KI;** Dissolve 10gm of KI in 100ml deionized water.

Prepared by: Addisu Legesse and Teshome Assefa	Authority: EPHI

NOTE: This is a CONTROLLED document for internal use only. Any documents appearing in paper form are not controlled and should be checked against the electronic version prior to use.

For peer review only

- **2N H_2SO_4;** Slowly add 6 ml of concentrated H_2SO_4 to 90 ml of deionized water make the final volume 100 ml.
- **0.005M $Na_2S_2O_3$;** Dissolve 1.24gm of $Na_2S_2O_3.5H_2O$ in 1000 ml of deionized water

4.3 Reagents stability and storage:

- $Na_2S_2O3.5H_2O$ & 10% KI reagents store in a cool & dark place for six months.
- H_2SO_4 store at room temperature it stable indefinitely.
- Starch should be prepared daily.

5. Supplies and Equipment

- Balance (Four-beam pan): Sensitivity = 0.01g, Capacity = 410g
- Flask, volumetric, 1000mL, 100mL
- Measuring cylinder, 10mL, 100mL
- Beakers (Pyrex)
- Flasks, Erlenmeyer (conical) with stopper, 250mL
- Pipette, volumetric, 1mL, 5mL
- Burette w/straight stopcock 10mL
- Burette stand
- Laboratory safety glasses
- Parafilm, for covering beakers
- Glass bottles with stoppers, for reagents, 250mL
- Spatula Lab single blade 150mm SS length
- Dropper bottle, glass 25-60mL
- Hot plate

6. Sample

- Sample type: salt
- Amount required: 50-100g
- Transport and storage: At room temperature avoid exposure to direct sunlight

NOTE: This is a CONTROLLED document for internal use only. Any documents appearing in paper form are not controlled and should be checked against the electronic version prior to use.

For peer review only - http://bmjopen.bmj.com/site/about/guidelines.xhtml

- Stability: At room temperature for 3 months.

7. Special Safety and Precautions

- While titrate the sample wear eye google.
- The reaction mixture should be kept in the dark before titration because a side reaction can occur when the solution is exposed to light that causes iodide ions to be oxidized to iodine.
- Inaccurate results may occur if starch solution is used while still warm.
- If starch indicator is added too early, a strong iodine-starch complex is formed, which reacts slowly, and gives falsely elevated results.
- The reaction should be performed at mild room temperature (<30 ^{0}C), since the iodine is volatile, and the indicator solution loses sensitivity when exposed to high temperatures.

8. Procedure

8.1 Weigh 10g of the salt sample into a 250mL Erlenmeyer flask with a stopper.

8.2 Add approximately 50 mL water, swirl to dissolve salt sample.

8.3 Add 1 ml 2N H_2SO_4

8.4 Add 5mL 10% KI. The solution should turn yellow if iodine is present.

8.5 Close the flask with stopper & put it in the dark place for 10 minutes in closed box (cupboard or drawer).

8.6 Rinse and fill burette with 0.005M $Na_2S_2O_3$, and adjust level to zero.

8.7 After 10 minutes take the flask out from drawer, and add some $Na_2S_2O_3$ from the titration burette Until the solution turns pale yellow.

8.8 Add approximately 2mL of starch indicator solution (the solution should turn dark purple) and continue titrating with 0.005M $Na_2S_2O_3$ until the solution becomes pink, and finally colorless.

8.9 Record the level of thiosulfate in the burette and convert to parts per million (ppm)

NOTE: This is a CONTROLLED document for internal use only. Any documents appearing in paper form are not controlled and should be checked against the electronic version prior to use.

For peer review only - http://bmjopen.bmj.com/site/about/guidelines.xhtml

9. Quality control:

- **Control material**: KIO_3
- **Level of iodine**: 59.3-60.3ppm
- **Stability**: stable at room temperature for long period of time.
- **Frequency**: per batch

10. Quality control preparation: Prepare 0.0047M KIO_3 in 100ml. By weighing 0.10058g in 100ml deionized water. From this solution Pipette out 1 ml into conical flask and follow the procedure proceed from step 2 as described above.

11. Calculation

Iodine(ppm) = **10.6* V $Na_2S_2O_3$(ml)**

Where: **V**$Na_2S_2O_3$: Volume of sodium thiosulphate takes to titrate iodine in salt

12. Result Interpretation

- **<5ppm** to indicate salt with no added iodine
- **5-14.9** ppm to indicate inadequately iodized salt
- **15-39.9** ppm to indicate salt is adequately iodized
- **>40** ppm of iodine is not recommended.

13. Other Records

- Data Log sheet
- QC chart

14. References

- De Maeyer, Lowenstein & Thilly, 1979; World Health Organization (WHO), United Nations Children Fund (UNICEF) & ICCIDD, 2007.
- AOAC 925.56 2016

NOTE: This is a CONTROLLED document for internal use only. Any documents appearing in paper form are not controlled and should be checked against the electronic version prior to use.

For peer review only

Name of institute	Doc. number:	Version No: 3
**ETHIOPIAN PUBLIC HEALTH INSTITUTE**	EPHI/ TL/ FNL/TM/7.2.11	Copy no_____
		Page 5 of 7
Document Title: **DETERMIANTION OF IODINE FROM SALT SAMPLE**	Effective date: November 20, 2020	

15. Annex

Conversion chart for iodine in fortified salt in the form of KIO_3 (PPM)

Volume Thiosulphate (mL)	Iodine (ppm)	Volume Thiosulphate (mL)	Iodine (ppm)	Volume Thiosulphate (mL)	Iodine (ppm)	Volume Thiosulphate (mL)	Iodine (ppm)	Volume Thiosulphate (mL)	Iodine (ppm)	Volume Thiosulphate (mL)	Iodine (ppm)
0.1	1.1	2.0	21.2	3.9	41.3	5.8	61.5	7.7	81.6	9.6	101.8
0.2	2.1	2.1	22.2	4.0	42.4	5.9	62.5	7.8	82.7	9.7	102.8
0.3	3.2	2.2	23.3	4.1	43.5	6.0	63.6	7.9	83.4	9.8	103.9
0.4	4.2	2.3	24.4	4.2	44.5	6.1	64.7	8.0	84.8	9.9	104.9
0.5	5.3	2.4	25.4	4.3	45.6	6.2	65.7	8.1	85.9	10.0	106.0
0.6	6.4	2.5	26.5	4.4	46.4	6.3	66.8	8.2	86.9	10.1	107.1
0.7	7.4	2.6	27.6	4.5	47.7	6.4	67.8	8.3	88.0	10.2	108.1
0.8	8.5	2.7	28.6	4.6	48.8	6.5	68.9	8.4	89.0	10.3	109.2
0.9	9.4	2.8	29.7	4.7	49.8	6.6	70.0	8.5	90.1	10.4	110.2
1.0	10.6	2.9	30.7	4.8	50.9	6.7	71.0	8.6	91.2	10.5	111.3
1.1	11.7	3.0	31.8	4.9	51.9	6.8	72.1	8.7	92.2	10.6	112.4
1.2	12.2	3.1	32.9	5.0	53.0	6.9	73.1	8.8	93.3	10.7	113.4
1.3	13.8	3.2	33.9	5.1	54.1	7.0	74.2	8.9	94.3	10.8	114.5
1.4	14.8	3.3	35.0	5.2	55.1	7.1	75.3	9.0	95.4	10.9	115.5
1.5	15.9	3.4	36.0	5.3	56.2	7.2	76.3	9.1	96.5	11.0	116.6
1.6	17.0	3.5	37.1	5.4	57.2	7.3	77.4	9.2	97.5	11.1	117.7
1.7	18.0	3.6	38.2	5.5	58.3	7.4	78.4	9.3	98.6	11.2	118.7
1.8	19.1	3.7	39.2	5.6	59.4	7.5	79.5	9.4	99.7	11.3	119.8
1.9	20.1	3.8	40.3	5.7	60.4	7.6	80.6	9.5	100.7	11.4	120.8

Prepared by: Addisu Legesse and Teshome Assefa	Authority: EPHI

NOTE: This is a CONTROLLED document for internal use only. Any documents appearing in paper form are not controlled and should be checked against the electronic version prior to use.

Name of institute<br>**ETHIOPIAN PUBLIC HEALTH INSTITUTE**	Doc. number:<br>EPHI/ TL/ FNL/TM/7.2.11	Version No: 3<br>Copy no_____<br>Page 6 of 7
Document Title: **DETERMIANTION OF IODINE FROM SALT SAMPLE**	Effective date: November 20, 2020	

Name Signature

Prepared by: -------------------------- ----------------------------

Reviewed by: ------------------------- ----------------------------

Approved by: -------------------------- -----------------------------

(Director of Food science and Nutrition research directorate)

Declaration

I, the undersigned laboratory personnel, certify that I am conducting every steps of the procedures incorporated in this SOP after a prior reading.

**Name**	**Signature and Date**
………………………………..	………………………………..
………………………………..	………………………………..
………………………………..	………………………………..
………………………………..	………………………………..
………………………………..	………………………………..

Prepared by: Addisu Legesse and Teshome Assefa	Authority: EPHI

NOTE: This is a CONTROLLED document for internal use only. Any documents appearing in paper form are not controlled and should be checked against the electronic version prior to use.

For peer review only

Name of institute	Doc. number:	Version No: 3
**ETHIOPIAN PUBLIC HEALTH INSTITUTE**	EPHI/ TL/ FNL/TM/7.2.11	Copy no_____
		Page 7 of 7
Document Title: **DETERMIANTION OF IODINE FROM SALT SAMPLE**	Effective date: November 20, 2020	

Document Change History

**Revision No.**	**Date approved**	**Nature of revision**
1	February, 2019	Initial release
2	November, 2020	Typographical adjustment<br>Document revision table is added

Prepared by: Addisu Legesse and Teshome Assefa	Authority: EPHI

NOTE: This is a CONTROLLED document for internal use only. Any documents appearing in paper form are not controlled and should be checked against the electronic version prior to use.

For peer review only

Determination of serum retinol using hexane as the serum sample extraction procedure

For peer review only

Contents

1. Reagents and materials 2

2. Instrument (HPLC) parameters – we have Shimadzu prominence HPLC. 2

3. Sample extraction procedure 2

4. Calculation 3

5. Reference 3

For peer review only

For peer review only - http://bmjopen.bmj.com/site/about/guidelines.xhtml

1. Reagents and materials

- Methanol (HPLC grade)
- Hexane (HPLC grade)
- Ethanol (HPLC grade/AR grade)
- Normal saline solution (0.9% NaCl)
- Retinol acetate (as internal standard)
- Retinol (as external standard)
- CRM (Certified Reference Material) for serum retinol
- 15 ml centrifuge tube
- Vortex mixer
- Centrifuge (up to 4000 rpm capacity)
- Centrifuge tube rack
- Micropipettes (10-100µl and 100-1000µl)
- Micropipettes tips for both types
- Stopwatch
- HPLC Vials (1ml)

2. Instrument (HPLC) parameters – we have Shimadzu prominence HPLC.

- Detector - SPD-10A UV/VIS at 325 nm
- Column – SUPELCOSIL™LC-NH_2-NP, 25cm × 4.6mm, 5µm
- Mobile phase – HPLC grade methanol
- Elution system – isocratic

3. Sample extraction procedure

- Add 200 µl serum sample to 15ml plastic test tube.
- Add an equal volume of 50 µg/dl retinol acetate and ethanol.
- Take 200 µl of a serious of standards (10, 20, 40, 60, 75 µg/dl) to the 15 ml centrifuge tube and add the same volume of retinol acetate and normal saline solution.
- Mix the standard and sample solutions with a vortex mixer
- Add 1ml of HPLC grade hexane and mix for 45 seconds
- Centrifuge the solution at 4000rpm for 7 minutes and transfer the supernatant to other test tubes
- The solution has to be extract twice by adding 1 ml hexane.

For peer review only - http://bmjopen.bmj.com/site/about/guidelines.xhtml

- Dry the hexane using nitrogen gas and reconstitute by 500µl HPLC grade methanol
- It has to be mix for 30 minutes using a vortex mixer and transfer to 1 ml HPLC vials
- Analyze the extracted solutions using reversed-phase HPLC by isocratic elution system, at the flow rate of 1.5 ml/min, and injection volume 30µl

N: B: - Extract and analyse the CRM using the sample extraction procedure.

4. Calculation

- Plot the calibration curve using area ratio of retinol (standard) and retinol acetate (internal standard) vs concentration ratio (retinol: retinol acetate).
- From the linear equation formula, Y = mx + b, the concentration of serum retinol can be calculated.

  Where Y – is area ratio (retinol: retinol acetate) of sample

  M – is slope and b – is Y intercept

  X – is the concentration of serum retinol in µg/dl

5. Reference

Quadro, Y.-K. K. and L. (2013). Reverse-Phase High-Performance Liquid Chromatography (HPLC) Analysis of Retinol and Retinyl Esters in Mouse Serum and Tissues. *Methods Mol Biol. 2010 ; 652: 263–275. Doi:10.1007/978-1-60327-325-1_15.*, (12), 1–10. https://doi.org/10.1007/978-1-60327-325-1

von Lucke, A., Russell, R. M., Stephensen, C. B., Gannon, B. M., Craft, N. E., Haskell, M. J., … Raiten, D. J. (2016). Biomarkers of Nutrition for Development (BOND)—Vitamin A Review. *The Journal of Nutrition*, 1816s-1848s. https://doi.org/10.3945/jn.115.229708.FIGURE

Prepared by: Nahom Tefera (September 2020)

Reviewed by: Meseret W/yohannis

Approved by: Dr. Masresha Tessema

For peer review only - http://bmjopen.bmj.com/site/about/guidelines.xhtml

	Name of institute<br>**ETHIOPIAN PUBLIC HEALTH INSTITUTE**	Doc. number:<br>**EPHI/TL/FNL/TM7.2-17**	Version No: 3<br>Copy No. _____<br>Page 1 of 5
Document Title: DETERMINATION OF IODINE IN URINE		Effective date: December, 2020	

1 Scope

This test method is applicable for the determination of iodine from a urine sample.

2 Purpose

This procedure provides methods to control the amount of iodine and it's toxicity by ammonium persulfate digestion with spectrophotometric detection of the Sandell-Kolthoff Reaction method from urine samples.

3 Principle

Urine is digested with ammonium persulfate. Iodide is the catalyst in the reduction of ceric ammonium sulfate (yellow) to cerous form (colorless) and is detected by the rate of color disappearance (Sandell-Kolthoff reaction).

4 Chemicals and Apparatus

a) Chemicals

- Ammonium persulphate
- Arsenic trioxide
- Ceric ammonium sulphate
- Potassium iodate
- Deionized water
- Sodium chloride

b) Apparatus

- Hot plate
- Oven
- UV-Vis spectrophotometer
- Volumetric flasks (100 – 2000 ml)
- Beakers (100- 1000 ml)

For peer review only

Name of institute	Doc. number:	Version No: 3
**ETHIOPIAN PUBLIC HEALTH INSTITUTE**	**EPHI/TL/FNL/TM7.2-17**	Copy No. _____
		Page 2 of 5
Document Title: DETERMINATION OF IODINE IN URINE	Effective date: December, 2020	

- Micropipette (100 – 1000 µl)
- Pipette (5 – 10 ml)
- Vortex mixer
- Measuring cylinder (100 – 1000 ml)
- Glass test tubes (13*100 mm)
- Analytical balance (nearest to the 0.0001 g)
- Erlenmeyer flask (2000 ml)

5 Reagents preparation

a) 1 M Ammonium persulphate

- Dissolve 114.1 g $H_2 N_2 O_8 S_2$ in deionized water and makeup to 500 ml with H_2O. Store away from light. Stable for at least one month.

b) 5 N H_2SO_4

- Slowly add 139 ml concentrated (36 N) H_2SO_4 to about 700 ml deionized water (careful - this generates heat!). When cool, adjust with deionized water to a final volume of 1 liter.

c) 3.5 N H_2SO_4

- Slowly add 97 ml concentrated (36 N) H_2SO_4 to about 800 ml deionized water (careful - this generates heat!), and when cool, adjusting with deionized water to a final volume of 1 liter

d) Ceric ammonium sulphate solution

- Dissolve 48 g ceric ammonium sulphate in 1 liter 3.5 N H_2SO_4. Store in a dark bottle away from light at room temperature. The solution is stable for months.

e) Arsenious acid solution

- In a 2000 ml Erlenmeyer flask, place 20 g As_2O_3 and 50 g NaCl, then slowly add 400 ml 5 N H_2SO_4. Add water to about 1 liter, heat gently to dissolve, cool to room temperature, dilute with water to 2 liters, filter, store in a dark bottle away from light at room temperature. The solution is stable for months.

For peer review only

Name of institute		Doc. number:	Version No: 3
	**ETHIOPIAN PUBLIC HEALTH INSTITUTE**	**EPHI/TL/FNL/TM7.2-17**	Copy No. _____
			Page 3 of 5
Document Title:	DETERMINATION OF IODINE IN URINE	Effective date: December, 2020	

6 Standards preparation

a) Stock standard solution (1 mg/ml)

- Dissolve 0.1685 g KIO_3 in deionized water to a final volume of 100 ml (1.68 g KIO_3 contains 1.0 g iodine). KIO_3 is preferred over KI because it is more stable. It may be more convenient to make a more concentrated solution, e.g., 10 or 100 mg iodine/ml.

b) Intermediate standard solution (1 µg/ml)

- Dilute 100 µl stock iodine standard (1mg/ml) to 100 ml deionized water. Store in a dark bottle. The solution is stable for months. Useful standards are 20, 50, 100, 150, 200, and 300 µg/l.

c) Serial standard dilutions

- From intermediate standards, prepare 50, 100, 150, 200, and 250 µg/L useful standards for calibration curve purposes.

Note: All standard solutions should store in a dark place. The solutions are stable for months.

7 Procedures

- Mix urine to suspend sediment using a vortex mixer.
- Pipette 250 µl of each urine sample into a 13 x 100 mm test tube.
- Pipette 250 µl each iodine serial standards also into a test tube.
- Add 1 ml 1.0 M ammonium persulfate solution to each tube.
- Vortex all tubes using a vortex mixer
- Heat all tubes for 60 minutes at 100° C in the oven.
- Cool tubes to room temperature in a fume hood.
- Add 2.5 ml arsenious acid solution. Mix by a vortex. Let stand for 15 minutes.
- Add 300 µl of ceric ammonium sulfate solution to each tube (quickly mixing) at 15-30 second intervals between successive tubes. A stopwatch should be used for this. With practice, a 20-second interval is convenient.

For peer review only

Name of institute		Doc. number:	Version No: 3
	**ETHIOPIAN PUBLIC HEALTH INSTITUTE**	**EPHI/TL/FNL/T M7.2-17**	Copy No. _____
			Page 4 of 5
Document Title: DETERMINATION OF IODINE IN URINE		Effective date: December, 2020	

- Allow sitting at room temperature. Exactly 30 minutes after the addition of ceric ammonium sulfate to the first tube, read its absorbance by using UV-Vis spectrophotometer at 420 nm. Read successive tubes at the same interval as when adding the ceric ammonium sulfate.

8 Calculation

- Construct a standard curve on graph paper by plotting log absorbance of each concentration versus iodine concentration of each standard.
- Urinary Iodine in µg/l = ((log A-b)/(m)) * 10
  Where log A is log absorbance of sample, b is Y-intercept of the calibration graph, m is the slope of the graph, and 10 is used as a conversion factor when we prepare serials of standards in µg/dl (in this case we multiply the final concentration (µg/ dl) by 10 to get the concentration in µg /l).

9 Quality control and safety precautions

- In each batch, the urine quality control sample (CRM or in-house prepared) should run together with sample and standards.
- While working the urine analysis and reagent preparation wear gloves, lab coat, eye goggle, lab shoe, and mouth cover.

Remember: During the analysis of iodine from urine, you should ensure that the laboratory working environment should free from salt samples (especially iodized salt samples) to avoid contamination.

Limitations: If the urine sample is analyzed at a high temperature the loss of iodine occurred and the method has to detect very low iodine concentration in urine.

10 References

- ICCIDD, UNICEF, WHO. Dunn JT et al. Methods for measuring iodine in urine. The Netherlands, ICCIDD, 1993.

For peer review only

Name of institute	Doc. number:	Version No: 3
**ETHIOPIAN PUBLIC HEALTH INSTITUTE**	**EPHI/TL/FNL/TM7.2-17**	Copy No. ______
		Page 5 of 5
Document Title: DETERMINATION OF IODINE IN URINE	Effective date: December, 2020	

Prepared by	Signature	Date
Prepared by: Nahom Tefera	____________	___________________
Reviewed by: Meseret W/yohannes	____________	____________________
Approved by: Dr. Masresha Tessema	____________	____________________

For peer review only

0003333795190c501V10.0

AAGP2

cobas®

Tina-quant α1-Acid Glycoprotein Gen.2

Order information

REF	CONTENT		Analyzer(s) on which **cobas c** pack(s) can be used
**03333795** 190	Tina-quant α1-Acid Glycoprotein Gen.2 100 tests	System-ID 07 6758 1	Roche/Hitachi **cobas c** 311, **cobas c** 501/502
**11355279** 216	Calibrator f.a.s. Proteins (5 x 1 mL)	Code 656	
**11355279** 160	Calibrator f.a.s. Proteins (5 x 1 mL, for USA)	Code 656	
**10557897** 122	Precinorm Protein (3 x 1 mL)	Code 302	
**10557897** 160	Precinorm Protein (3 x 1 mL, for USA)	Code 302	
**11333127** 122	Precipath Protein (3 x 1 mL)	Code 303	
**11333127** 160	Precipath Protein (3 x 1 mL, for USA)	Code 303	
**05117003** 190	PreciControl ClinChem Multi 1 (20 x 5 mL)	Code 391	
**05947626** 190	PreciControl ClinChem Multi 1 (4 x 5 mL)	Code 391	
**05947626** 160	PreciControl ClinChem Multi 1 (4 x 5 mL, for USA)	Code 391	
**05117216** 190	PreciControl ClinChem Multi 2 (20 x 5 mL)	Code 392	
**05947774** 190	PreciControl ClinChem Multi 2 (4 x 5 mL)	Code 392	
**05947774** 160	PreciControl ClinChem Multi 2 (4 x 5 mL, for USA)	Code 392	
**04489357** 190	Diluent NaCl 9 % (50 mL)	System-ID 07 6869 3	

English

System information

For **cobas c** 311/501 analyzers:

AAGP2: ACN 229

For **cobas c** 502 analyzer:

AAGP2: ACN 8229

Intended use

In vitro test for the quantitative determination of α_1-acid glycoprotein in human serum and plasma on Roche/Hitachi **cobas c** systems.

Summary[1,2,3,4,5]

α_1-Acid glycoprotein is synthesized in hepatocytes and consists of a polypeptide chain having 5 carbohydrate chains N-glycosidically bonded to it (molar mass 41000 daltons). Structurally, it belongs to the lipocalin superfamily of secretory proteins (such as α_1-microglobulin and retinol-binding protein). α_1-Acid glycoprotein promotes fibroblast growth and interacts with collagen.

It is a sensitive acute phase reactant whose concentration can increase by a factor of 3 within 24-48 hours when inflammation occurs. α_1-Acid glycoprotein can also be used to differentiate between acute phase reactions (elevated serum level) and estrogen effects (normal or decreased serum level) whereas the serum level of other positive reactants such as ceruloplasmin and haptoglobin increases during such reactions. Along with haptoglobin it is perhaps the best protein for identifying slight in vivo hemolysis. An increased α_1-acid glycoprotein level and normal haptoglobin values indicate an acute phase reaction with concomitant slight in vivo hemolysis. Moderate and isolated increases occur when glomerular filtration is inhibited in the early stages of uremia. The determination is used in the assessment of the activity of acute and recurring inflammations as well as of tumors with cell necrosis.

Various assay methods for α_1-acid glycoprotein determination are available such as kinetic nephelometry, radial immunodiffusion (RID) and turbidimetry. The Roche α_1-acid glycoprotein assay is based on the principle of immunological agglutination.

Test principle[2]

Immunoturbidimetric assay.

Anti-α_1-acid glycoprotein antibodies react with antigen in the sample to form an antigen/antibody complex. Following agglutination, this is measured turbidimetrically.

Reagents - working solutions

R1 TRIS buffer: 50 mmol/L, pH 8.0; NaCl: 300 mmol/L; PEG: 7 %; preservative; stabilizer

R2 Polyclonal anti-human α_1-acid glycoprotein antibody (goat): dependent on titer; TRIS buffer: 13 mmol/L, pH 7.5; NaCl: 198 mmol/L; preservative

R1 is in position B and R2 is in position C.

Precautions and warnings

For in vitro diagnostic use.
Exercise the normal precautions required for handling all laboratory reagents.
Disposal of all waste material should be in accordance with local guidelines.
Safety data sheet available for professional user on request.

For USA: Caution: Federal law restricts this device to sale by or on the order of a physician.

Reagent handling

Ready for use

Storage and stability

AAGP2

Shelf life at 2-8 °C:	See expiration date on **cobas c** pack label.
On-board in use and refrigerated on the analyzer:	12 weeks

Diluent NaCl 9 %

Shelf life at 2-8 °C:	See expiration date on **cobas c** pack label.
On-board in use and refrigerated on the analyzer:	12 weeks

Specimen collection and preparation

For specimen collection and preparation only use suitable tubes or collection containers.

Only the specimens listed below were tested and found acceptable.
Serum
Plasma: Li-heparin and K_2-EDTA plasma.

The sample types listed were tested with a selection of sample collection tubes that were commercially available at the time of testing, i.e. not all available tubes of all manufacturers were tested. Sample collection systems from various manufacturers may contain differing materials which could affect the test results in some cases. When processing samples in primary tubes (sample collection systems), follow the instructions of the tube manufacturer.

Centrifuge samples containing precipitates before performing the assay.

Stability:[6]	< 72 hours at 4 °C
	6 months at -20 °C

For peer review only
For peer review only - http://bmjopen.bmj.com/site/about/guidelines.xhtml

0003333795190c501V10.0

AAGP2

cobas®

Tina-quant α1-Acid Glycoprotein Gen.2

Materials provided

See “Reagents – working solutions” section for reagents.

Materials required (but not provided)

- See “Order information” section
- General laboratory equipment

Assay

For optimum performance of the assay follow the directions given in this document for the analyzer concerned. Refer to the appropriate operator’s manual for analyzer-specific assay instructions.

The performance of applications not validated by Roche is not warranted and must be defined by the user.

Application for serum and plasma

cobas c 311 **test definition**

Assay type	2-Point End		
Reaction time/Assay points	10/6-32		
Wavelength (sub/main)	660/340 nm		
Reaction direction	Increase		
Units	g/L (µmol/L, mg/dL)		
Reagent pipetting		Diluent (H_2O)	
R1	120 µL	–	
R2	40 µL	–	
Sample volumes	*Sample*	*Sample dilution*	
		Sample	*Diluent (NaCl)*
Normal	12 µL	9 µL	180 µL
Decreased	12 µL	4 µL	122 µL
Increased	12 µL	9 µL	180 µL

cobas c 501 **test definition**

Assay type	2-Point End		
Reaction time/Assay points	10/10-48		
Wavelength (sub/main)	660/340 nm		
Reaction direction	Increase		
Units	g/L (µmol/L, mg/dL)		
Reagent pipetting		Diluent (H_2O)	
R1	120 µL	–	
R2	40 µL	–	
Sample volumes	*Sample*	*Sample dilution*	
		Sample	*Diluent (NaCl)*
Normal	12 µL	9 µL	180 µL
Decreased	12 µL	4 µL	122 µL
Increased	12 µL	9 µL	180 µL

cobas c 502 **test definition**

Assay type	2-Point End		
Reaction time/Assay points	10/10-48		
Wavelength (sub/main)	660/340 nm		
Reaction direction	Increase		
Units	g/L (µmol/L, mg/dL)		
Reagent pipetting		Diluent (H_2O)	
R1	120 µL	–	
R2	40 µL	–	
Sample volumes	*Sample*	*Sample dilution*	
		Sample	*Diluent (NaCl)*
Normal	12 µL	9 µL	180 µL
Decreased	12 µL	4 µL	122 µL
Increased	12 µL	18 µL	180 µL

Calibration

Calibrators	S1: H_2O	
	S2-S6: C.f.a.s. Proteins	
	Multiply the lot-specific C.f.a.s. Proteins calibrator value by the factors below to determine the standard concentrations for the 6-point calibration curve:	
	S2: 0.140	S5: 1.40
	S3: 0.280	S6: 2.81
	S4: 0.700	
Calibration mode	RCM2	
Calibration frequency	Full calibration<br>- after reagent lot change<br>- as required following quality control procedures	

Calibration interval may be extended based on acceptable verification of calibration by the laboratory.

Traceability: This method has been standardized against the reference preparation of the IRMM (Institute for Reference Materials and Measurements) BCR470/CRM470 (RPPHS - Reference Preparation for Proteins in Human Serum).[7]

Quality control

For quality control, use control materials as listed in the "Order information" section.

In addition, other suitable control material can be used.

The control intervals and limits should be adapted to each laboratory’s individual requirements. Values obtained should fall within the defined limits. Each laboratory should establish corrective measures to be taken if values fall outside the defined limits.

Follow the applicable government regulations and local guidelines for quality control.

Calculation

Roche/Hitachi **cobas c** systems automatically calculate the analyte concentration of each sample.

Conversion factors:	g/L x 25 = µmol/L	mg/dL x 0.01 = g/L
	mg/dL x 0.25 = µmol/L	g/L x 100 = mg/dL

Limitations - interference

Criterion: Recovery within ± 10 % of initial value at an α_1-acid glycoprotein concentration of 0.5 g/L (12.5 µmol/L, 50 mg/dL).

Icterus:[8] No significant interference up to an I index of 60 for conjugated and unconjugated bilirubin (approximate conjugated and unconjugated bilirubin concentration: 1026 µmol/L or 60 mg/dL).

Hemolysis:[8] No significant interference up to an H index of 1000 (approximate hemoglobin concentration: 621 µmol/L or 1000 mg/dL).

Lipemia (Intralipid):[8] No significant interference up to an L index of 650. There is poor correlation between the L index (corresponds to turbidity) and triglycerides concentration.

Rheumatoid factors up to 1200 IU/mL do not interfere.

High dose hook-effect: No false result occurs up to an α_1-acid glycoprotein concentration of 11 g/L (275 µmol/L, 1100 mg/dL).

Drugs: No interference was found at therapeutic concentrations using common drug panels.[9, 10]

In very rare cases, gammopathy, in particular type IgM (Waldenström’s macroglobulinemia), may cause unreliable results.[11]

For peer review only

For peer review only - http://bmjopen.bmj.com/site/about/guidelines.xhtml

0003333795190c501V10.0

AAGP2

cobas®

Tina-quant α1-Acid Glycoprotein Gen.2

For diagnostic purposes, the results should always be assessed in conjunction with the patient's medical history, clinical examination and other findings.

ACTION REQUIRED

Special Wash Programming: The use of special wash steps is mandatory when certain test combinations are run together on Roche/Hitachi **cobas c** systems. The latest version of the carry-over evasion list can be found with the NaOHD-SMS-SmpCln1+2-SCCS Method Sheets. For further instructions refer to the operator's manual. **cobas c** 502 analyzer: All special wash programming necessary for avoiding carry-over is available via the **cobas** link, manual input is not required.

Where required, special wash/carry-over evasion programming must be implemented prior to reporting results with this test.

Limits and ranges

Measuring range

0.1-4.0 g/L (2.5-100 µmol/L, 10-400 mg/dL)

Determine samples having higher concentrations via the rerun function. Dilution of samples via the rerun function is a 1:1.5 dilution. Results from samples diluted using the rerun function are automatically multiplied by a factor of 1.5.

Lower limits of measurement

Lower detection limit of the test

0.1 g/L (2.5 µmol/L, 10 mg/dL)

The lower detection limit represents the lowest measurable analyte level that can be distinguished from zero. It is calculated as the value lying 3 standard deviations above that of the lowest standard (standard 1 + 3 SD, repeatability, n = 21).

Expected values[12]

0.5-1.2 g/L (12.5-30 µmol/L, 50-120 mg/dL)

Each laboratory should investigate the transferability of the expected values to its own patient population and if necessary determine its own reference ranges.

Specific performance data

Representative performance data on the analyzers are given below. Results obtained in individual laboratories may differ.

Precision

Precision was determined using human samples and controls in an internal protocol with repeatability (n = 21) and intermediate precision (3 aliquots per run, 1 run per day, 21 days). The following results were obtained:

Repeatability	*Mean*	*SD*	*CV*
	g/L (µmol/L, mg/dL)	*g/L (µmol/L, mg/dL)*	*%*
Precinorm Protein	0.724 (18.1, 72.4)	0.00 (0.0, 0.0)	0.6
Precipath Protein	1.21 (30.3, 121)	0.01 (0.3, 1)	0.5
Human serum 1	0.642 (16.1, 64.2)	0.00 (0.0, 0.0)	0.7
Human serum 2	1.07 (26.8, 107)	0.01 (0.3, 1)	0.7

Intermediate precision	*Mean*	*SD*	*CV*
	g/L (µmol/L, mg/dL)	*g/L (µmol/L, mg/dL)*	*%*
Precinorm Protein	0.710 (17.8, 71.0)	0.007 (0.2, 1.0)	0.9
Precipath Protein	1.19 (30.0, 119)	0.01 (0.3, 1)	0.9
Human serum 3	0.660 (16.5, 66.0)	0.010 (0.3, 1.0)	1.5
Human serum 4	1.21 (30.3, 121)	0.02 (0.5, 2)	1.5

Method comparison

α_1-Acid glycoprotein values for human serum and plasma samples obtained on a Roche/Hitachi **cobas c** 501 analyzer (y) were compared with those determined using the corresponding reagent on a Roche/Hitachi 917 analyzer (x).

Sample size (n) = 119

Passing/Bablok[13]	Linear regression
y = 1.012x - 0.070 g/L	y = 0.998x - 0.056 g/L
τ = 0.973	r = 0.999

The sample concentrations were between 0.489 and 3.25 g/L (12.2 and 81.3 µmol/L, 48.9 and 325 mg/dL).

References

1 Schmid K. α1-Acid glycoprotein. In: The Plasma Proteins, 2nd ed. Putnam FW, ed. New York: Academic Press 1975;183-228.

2 Greiling H, Gressner AM, eds. Lehrbuch der Klinischen Chemie und Pathobiochemie, 3rd ed. Stuttgart/New York: Schattauer Verlag 1995:236.

3 Tietz NW, ed. Clinical Guide to Laboratory Tests, 3rd ed. Philadelphia, PA: WB Saunders Company 1995;66-67.

4 Ganrot K. Plasma protein pattern in acute infectious disease. Scand J Clin Lab Invest 1974;34:75-81.

5 Lievens M, Bienvenu J, Buitrago JMG, et al. Evaluation of four new Tina-quant assays for determination of α1-acid glycoprotein, α1-antitrypsin, haptoglobin and prealbumin. Clin Lab 1996;42:515-520.

6 Wu AHB, ed. Tietz Clinical Guide to Laboratory Tests, 4th ed. St. Louis (MO): Saunders Elsevier 2006:42.

7 Baudner S, Bienvenu J, Blirup-Jensen S, et al. The certification of a matrix reference material for immunochemical measurement of 14 human serum proteins CRM470. Report EUR 15243 EN 1993;1-186.

8 Glick MR, Ryder KW, Jackson SA. Graphical Comparisons of Interferences in Clinical Chemistry Instrumentation. Clin Chem 1986;32:470-475.

9 Breuer J. Report on the Symposium "Drug effects in Clinical Chemistry Methods". Eur J Clin Chem Clin Biochem 1996;34:385-386.

10 Sonntag O, Scholer A. Drug interference in clinical chemistry: recommendation of drugs and their concentrations to be used in drug interference studies. Ann Clin Biochem 2001;38:376-385.

11 Bakker AJ, Mücke M. Gammopathy interference in clinical chemistry assays: mechanisms, detection and prevention. Clin Chem Lab Med 2007;45(9):1240-1243.

12 Dati F, Schumann G, Thomas L, et al. Consensus of a group of professional societies and diagnostic companies on guidelines for interim reference ranges for 14 proteins in serum based on the standardization against the IFCC/BCR/CAP reference material (CRM 470). Eur J Clin Chem Clin Biochem 1996;34:517-520.

13 Bablok W, Passing H, Bender R, et al. A general regression procedure for method transformation. Application of linear regression procedures for method comparison studies in clinical chemistry, Part III. J Clin Chem Clin Biochem 1988 Nov;26(11):783-790.

A point (period/stop) is always used in this Method Sheet as the decimal separator to mark the border between the integral and the fractional parts of a decimal numeral. Separators for thousands are not used.

Symbols

Roche Diagnostics uses the following symbols and signs in addition to those listed in the ISO 15223-1 standard (for USA: see https://usdiagnostics.roche.com for definition of symbols used):

CONTENT	Contents of kit
→	Volume after reconstitution or mixing
GTIN	Global Trade Item Number

For peer review only - http://bmjopen.bmj.com/site/about/guidelines.xhtml

0003333795190c501V10.0

AAGP2

cobas®

Tina-quant α1-Acid Glycoprotein Gen.2

FOR US CUSTOMERS ONLY: LIMITED WARRANTY

Roche Diagnostics warrants that this product will meet the specifications stated in the labeling when used in accordance with such labeling and will be free from defects in material and workmanship until the expiration date printed on the label. THIS LIMITED WARRANTY IS IN LIEU OF ANY OTHER WARRANTY, EXPRESS OR IMPLIED, INCLUDING ANY IMPLIED WARRANTY OF MERCHANTABILITY OR FITNESS FOR PARTICULAR PURPOSE. IN NO EVENT SHALL ROCHE DIAGNOSTICS BE LIABLE FOR INCIDENTAL, INDIRECT, SPECIAL OR CONSEQUENTIAL DAMAGES.

COBAS, COBAS C, PRECICONTROL, PRECINORM, PRECIPATH and TINA-QUANT are trademarks of Roche.
All other product names and trademarks are the property of their respective owners.

Additions, deletions or changes are indicated by a change bar in the margin.

© 2017, Roche Diagnostics

CE

Roche Diagnostics GmbH, Sandhofer Strasse 116, D-68305 Mannheim
www.roche.com

Distribution in USA by:
Roche Diagnostics, Indianapolis, IN
US Customer Technical Support 1-800-428-2336

Roche

For peer review only

ms_05894913190V7.0

Vitamin D total

cobas®

25-Hydroxyvitamin D

REF	Σ	SYSTEM
**05894913** 190	100	Elecsys 2010<br>MODULAR ANALYTICS E170<br>**cobas e** 411<br>**cobas e** 601<br>**cobas e** 602

English

Intended use

This assay is intended for the quantitative determination of total 25-hydroxyvitamin D in human serum and plasma. This assay is to be used as an aid in the assessment of vitamin D sufficiency.

The electrochemiluminescence binding assay is intended for use on Elecsys and **cobas e** immunoassay analyzers.

Summary

Vitamin D is a fat-soluble steroid hormone precursor that is mainly produced in the skin by exposure to sunlight. Vitamin D is biologically inert and must undergo two successive hydroxylations in the liver and kidney to become the biologically active 1,25-dihydroxyvitamin D.[1]

The two most important forms of vitamin D are vitamin D_3 (cholecalciferol) and vitamin D_2 (ergocalciferol). In contrast to vitamin D_3, the human body cannot produce vitamin D_2 which is taken up with fortified food or given by supplements. In human plasma vitamin D_3 and D_2 are bound to the vitamin D binding protein and transported to the liver where both are hydroxylated to form vitamin D (25-OH), i.e. 25-hydroxyvitamin D. It is commonly agreed that vitamin D (25-OH) is the metabolite to determine the overall vitamin D status as it is the major storage form of vitamin D in the human body. This primary circulating form of vitamin D is biologically inactive with levels approximately 1000-fold greater than the circulating 1,25-dihydroxyvitamin D. The half-life of circulating vitamin D (25-OH) is 2-3 weeks.

Most of the vitamin D (25-OH), measurable in serum, is vitamin D_3 (25-OH) whereas vitamin D_2 (25-OH) reaches measurable levels only in patients taking vitamin D_2 supplements.[2,3,4] Vitamin D_2 is considered to be less effective.[5]

Vitamin D is essential for bone health. In children, severe deficiency leads to bone-malformation, known as rickets. Milder degrees of insufficiency are believed to cause reduced efficiency in the utilization of dietary calcium.[6] Vitamin D deficiency causes muscle weakness; in elderly, the risk of falling has been attributed to the effect of vitamin D on muscle function.[7] Vitamin D deficiency is a common cause of secondary hyperparathyroidism.[8,9] Elevations of PTH levels, especially in elderly vitamin D deficient adults can result in osteomalacia, increased bone turnover, reduced bone mass and risk of bone fractures.[10] Low vitamin D (25-OH) concentrations are also associated with lower bone mineral density.[11] In conjunction with other clinical data, the results may be used as an aid in the assessment of bone metabolism.

So far, vitamin D has been shown to affect expression of over 200 different genes. Insufficiency has been linked to diabetes, different forms of cancer, cardiovascular disease, autoimmune diseases and innate immunity.[2]

The Elecsys Vitamin D total assay employs a vitamin D binding protein (VDBP) as capture protein to bind vitamin D_3 (25-OH) and vitamin D_2 (25-OH).

Test principle

Competition principle. Total duration of assay: 27 minutes.

- 1st incubation: By incubating the sample (15 µL) with pretreatment reagent 1 and 2, bound vitamin D (25-OH) is released from the vitamin D binding protein.
- 2nd incubation: By incubating the pretreated sample with the ruthenium labeled vitamin D binding protein, a complex between the vitamin D (25-OH) and the ruthenylated vitamin D binding protein is formed.
- 3rd incubation: After addition of streptavidin-coated microparticles and vitamin D (25-OH) labeled with biotin, unbound ruthenium labeled vitamin D binding proteins become occupied. A complex consisting of the ruthenylated vitamin D binding protein and the biotinylated vitamin D (25-OH) is formed and becomes bound to the solid phase via interaction of biotin and streptavidin.
- The reaction mixture is aspirated into the measuring cell where the microparticles are magnetically captured onto the surface of the electrode. Unbound substances are then removed with ProCell/ProCell M. Application of a voltage to the electrode then induces chemiluminescent emission which is measured by a photomultiplier.
- Results are determined via a calibration curve which is instrument-specifically generated by 2-point calibration and a master curve provided via the reagent barcode.

Reagents - working solutions

The reagent rackpack (M, R1, R2) and the pretreatment reagents (PT1, PT2) are labeled as VITD-T.

PT1	Pretreatment reagent 1 (white cap), 1 bottle, 4 mL:
	Dithiothreitol 1 g/L, pH 5.5.
PT2	Pretreatment reagent 2 (gray cap), 1 bottle, 4 mL:
	Sodium hydroxide 55 g/L.
M	Streptavidin-coated microparticles (transparent cap), 1 bottle, 6.5 mL:
	Streptavidin-coated microparticles 0.72 mg/mL; preservative.
R1	Vitamin D binding protein-BPRu (gray cap), 1 bottle, 9 mL:
	Ruthenium labeled vitamin D binding protein 150 µg/L; bis-tris propane buffer 200 mmol/L; albumin (human) 25 g/L; pH 7.5; preservative.
R2	25-hydroxyvitamin D~biotin (black cap), 1 bottle, 8.5 mL:
	Biotinylated vitamin D (25-OH) 14 µg/L; bis-tris propane buffer 200 mmol/L; pH 8.6; preservative.

Precautions and warnings

For in vitro diagnostic use.
Exercise the normal precautions required for handling all laboratory reagents.
Disposal of all waste material should be in accordance with local guidelines.
Safety data sheet available for professional user on request.

This kit contains components classified as follows in accordance with the Regulation (EC) No. 1272/2008:

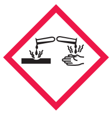

Danger

H290	May be corrosive to metals.
H314	Causes severe skin burns and eye damage.

Prevention:

P280	Wear protective gloves/ protective clothing/ eye protection/ face protection.

Response:

For peer review only - http://bmjopen.bmj.com/site/about/guidelines.xhtml

ms_05894913190V7.0

Vitamin D total

cobas®

25-Hydroxyvitamin D

P303 + P361 + P353	IF ON SKIN (or hair): Remove/Take off immediately all contaminated clothing. Rinse skin with water/shower.
P304 + P340 + P310	IF INHALED: Remove person to fresh air and keep comfortable for breathing. Immediately call a POISON CENTER or doctor/physician.
P305 + P351 + P338 + P310	IF IN EYES: Rinse cautiously with water for several minutes. Remove contact lenses, if present and easy to do. Continue rinsing. Immediately call a POISON CENTER or doctor/ physician.

Product safety labeling primarily follows EU GHS guidance.

Contact phone: all countries: +49-621-7590

All human material should be considered potentially infectious. All products derived from human blood are prepared exclusively from the blood of donors tested individually and shown to be free from HBsAg and antibodies to HCV and HIV. The testing methods applied were FDA-approved or cleared in compliance with the European Directive 98/79/EC, Annex II, List A.

However, as no testing method can rule out the potential risk of infection with absolute certainty, the material should be handled with the same level of care as a patient specimen. In the event of exposure, the directives of the responsible health authorities should be followed.[12,13]

Avoid foam formation in all reagents and sample types (specimens, calibrators and controls).

Reagent handling

The reagents in the kit have been assembled into a ready-for-use unit that cannot be separated.

All information required for correct operation is read in from the respective reagent barcodes.

Storage and stability

Store at 2-8 °C.

Do not freeze.

Store the Elecsys reagent kit **upright** in order to ensure complete availability of the microparticles during automatic mixing prior to use.

Stability:	
unopened at 2-8 °C	up to the stated expiration date
after opening at 2-8 °C	56 days (8 weeks)
on Elecsys 2010 and **cobas e** 411	21 days (3 weeks)
on MODULAR ANALYTICS E170, **cobas e** 601 and **cobas e** 602	28 days (4 weeks)

Specimen collection and preparation

Only the specimens listed below were tested and found acceptable.

Serum collected using standard sampling tubes or tubes containing separating gel.

Li-heparin, K_2- and K_3-EDTA plasma as well as Li-heparin plasma tubes containing separating gel.

Criterion: Method comparison serum versus plasma, slope 0.9-1.1 + intercept within < ± 2 x LoB + coefficient of correlation > 0.9.

Serum, Li-heparin, K_2- and K_3-EDTA plasma: Vitamin D (25-OH) is stable for 8 hours at 18-25 °C, 4 days at 2-8 °C, 24 weeks at -20 °C.

The stability of vitamin D (25-OH) found with the Elecsys Vitamin D total assay is in line with earlier studies using a vitamin D binding protein assay and mass spectrometry.[14]

The sample types listed were tested with a selection of sample collection tubes that were commercially available at the time of testing, i.e. not all available tubes of all manufacturers were tested. Sample collection systems from various manufacturers may contain differing materials which could affect the test results in some cases. When processing samples in primary tubes (sample collection systems), follow the instructions of the tube manufacturer.

Centrifuge samples containing precipitates before performing the assay.

Do not use heat-inactivated samples.

Do not use samples and controls stabilized with azide.

Ensure the samples, calibrators and controls are at 20-25 °C prior to measurement.

Due to possible evaporation effects, samples, calibrators and controls on the analyzers should be analyzed/measured within 2 hours.

Materials provided

See “Reagents – working solutions” section for reagents.

Materials required (but not provided)

- REF 05894921190, Vitamin D total CalSet, for 4 x 1 mL
- REF 05618860190, PreciControl Varia, for 2 x 3 mL each of PreciControl Varia 1 and 2
- REF 11732277122, Diluent Universal, 2 x 16 mL sample diluent or
  REF 03183971122, Diluent Universal, 2 x 36 mL sample diluent
- General laboratory equipment
- Elecsys 2010, MODULAR ANALYTICS E170 or **cobas e** analyzer

Accessories for Elecsys 2010 and **cobas e** 411 analyzers:

- REF 11662988122, ProCell, 6 x 380 mL system buffer
- REF 11662970122, CleanCell, 6 x 380 mL measuring cell cleaning solution
- REF 11930346122, Elecsys SysWash, 1 x 500 mL washwater additive
- REF 11933159001, Adapter for SysClean
- REF 11706802001, Elecsys 2010 AssayCup, 60 x 60 reaction vessels
- REF 11706799001, Elecsys 2010 AssayTip, 30 x 120 pipette tips

Accessories for MODULAR ANALYTICS E170, **cobas e** 601 and **cobas e** 602 analyzers:

- REF 04880340190, ProCell M, 2 x 2 L system buffer
- REF 04880293190, CleanCell M, 2 x 2 L measuring cell cleaning solution
- REF 03023141001, PC/CC-Cups, 12 cups to prewarm ProCell M and CleanCell M before use
- REF 03005712190, ProbeWash M, 12 x 70 mL cleaning solution for run finalization and rinsing during reagent change
- REF 03004899190, PreClean M, 5 x 600 mL detection cleaning solution
- REF 12102137001, AssayTip/AssayCup Combimagazine M, 48 magazines x 84 reaction vessels or pipette tips, waste bags
- REF 03023150001, WasteLiner, waste bags
- REF 03027651001, SysClean Adapter M

Accessories for all analyzers:

- REF 11298500316, ISE Cleaning Solution/Elecsys SysClean, 5 x 100 mL system cleaning solution

Assay

For optimum performance of the assay follow the directions given in this document for the analyzer concerned. Refer to the appropriate operator’s manual for analyzer-specific assay instructions.

Resuspension of the microparticles takes place automatically prior to use. Read in the test-specific parameters via the reagent barcode. If in exceptional cases the barcode cannot be read, enter the 15-digit sequence of numbers.

MODULAR ANALYTICS E170, **cobas e** 601 and **cobas e** 602 analyzers: PreClean M solution is necessary.

Bring the cooled reagents to approximately 20 °C and place on the reagent disk (20 °C) of the analyzer. Avoid foam formation. The system automatically regulates the temperature of the reagents and the opening/closing of the bottles.

Calibration

Traceability: This method has been standardized against LC-MS/MS[15] which in turn has been standardized to the NIST standard.[16]

Every Elecsys reagent set has a barcoded label containing specific information for calibration of the particular reagent lot. The predefined master curve is adapted to the analyzer using the relevant CalSet.

For peer review only - http://bmjopen.bmj.com/site/about/guidelines.xhtml

ms_05894913190V7.0

Vitamin D total

cobas®

25-Hydroxyvitamin D

Calibration frequency: Calibration must be performed once per reagent lot using fresh reagent (i.e. not more than 24 hours since the reagent kit was registered on the analyzer). Renewed calibration is recommended as follows:

- after 1 month (28 days) when using the same reagent lot
- after 7 days (when using the same reagent kit on the analyzer)
- as required: e.g. quality control findings outside the defined limits

Quality control

For quality control, use PreciControl Varia.

In addition, other suitable control material can be used.

Controls for the various concentration ranges should be run individually at least once every 24 hours when the test is in use, once per reagent kit, and following each calibration.

The control intervals and limits should be adapted to each laboratory's individual requirements. Values obtained should fall within the defined limits. Each laboratory should establish corrective measures to be taken if values fall outside the defined limits.

Follow the applicable government regulations and local guidelines for quality control.

Calculation

The analyzer automatically calculates the analyte concentration of each sample (either in ng/mL or nmol/L).

Conversion factors: nmol/L x 0.40 = ng/mL
ng/mL x 2.50 = nmol/L

Limitations - interference

Samples showing visible signs of hemolysis may cause interference.

Hemoglobin concentrations > 2 g/L (> 0.124 mmol/L) may lead to elevated results.

The assay is unaffected by icterus (bilirubin < 1129 µmol/L or < 66 mg/dL), lipemia (Intralipid < 400 mg/dL) and biotin (< 287 nmol/L or < 70 ng/mL).

Criterion: For concentrations from LoQ up to 15 ng/mL, deviation is ≤ 1.5 ng/mL; for concentrations > 15 ng/mL, deviation is ≤ 10 %.

Samples should not be taken from patients receiving therapy with high biotin doses (i.e. > 5 mg/day) until at least 8 hours following the last biotin administration.

In vitro tests were performed on 17 commonly used pharmaceuticals and 5 special therapeutic drugs (Bonviva (Ibandronate), EinsAlpha (Alfacalcidol), Fosamax (Alendronate), Pamidron HEXAL (Pamidronate) and Zometa (Zoledronate)). No interference with the assay was found.

In rare cases, interference due to extremely high titers of antibodies to analyte-specific antibodies, streptavidin or ruthenium can occur. These effects are minimized by suitable test design.

For diagnostic purposes, the results should always be assessed in conjunction with the patient's medical history, clinical examination and other findings.

Limits and ranges

Measuring range

3.00-70.0 ng/mL or 7.50-175 nmol/L (defined by the Limit of Detection and the maximum of the master curve). Values below the Limit of Detection are reported as < 3.00 ng/mL (< 7.50 nmol/L). Values above the measuring range are reported as > 70.0 ng/mL (> 175 nmol/L).

Lower limits of measurement

Limit of Blank (LoB), Limit of Detection (LoD) and Limit of Quantitation (LoQ)

Limit of Blank = 2.00 ng/mL (5.00 nmol/L)

Limit of Detection = 3.00 ng/mL (7.50 nmol/L)

Limit of Quantitation = 5.00 ng/mL (12.5 nmol/L) with a total allowable relative error of ≤ 30 %

The Limit of Blank, Limit of Detection and Limit of Quantitation were determined in accordance with the CLSI (Clinical and Laboratory Standards Institute) EP17-A requirements.

The Limit of Blank is the 95th percentile value from n ≥ 60 measurements of analyte-free samples over several independent series. The Limit of Blank corresponds to the concentration below which analyte-free samples are found with a probability of 95 %.

The Limit of Detection is determined based on the Limit of Blank and the standard deviation of low concentration samples. The Limit of Detection corresponds to the lowest analyte concentration which can be detected (value above the Limit of Blank with a probability of 95 %).

The Limit of Quantitation is defined as the lowest amount of analyte in a sample that can be accurately quantitated with a total allowable relative error of ≤ 30 %.

The total error concept describes the maximum possible error of a test result taking into account the imprecision (SD) and inaccuracy (bias) of the test system. The Total Error (TE) was determined using the RMS (Root Mean Square) model (CLSI EP17-A2). The relative allowable total error refers to the respective concentration of the sample.

Dilution

Samples with vitamin D (25-OH) concentrations above the measuring range can be manually diluted with Diluent Universal or a suitable human serum with a low analyte concentration. The recommended dilution is 1:2. The concentration of the diluted sample must be > 30.0 ng/mL (> 75.0 nmol/L). After manual dilution, multiply the results by the dilution factor 2. The endogenous analyte concentration of the human serum used for dilution has to be taken into account.

Expected values

Due to different standardizations between methods, result variation may arise. Clinical assessment should be taken into consideration when interpreting results.

Health based reference values (recommended for use):

Currently there is no standard definition of the optimal vitamin D status. Many specialists consider the commonly used population based reference values too low. Health based reference values are recommended to replace population based reference values.[17]

Most experts agree that vitamin D deficiency should be defined as vitamin D (25-OH) of ≤ 20 ng/mL (≤ 50 nmol/L).[18] Vitamin D insufficiency is recognized as 21-29 ng/mL.[18] Similarly, the US National Kidney Foundation considers levels < 30 ng/mL to be insufficient or deficient.[19]

The preferred level for vitamin D (25-OH) by many experts is now recommended to be ≥ 30 ng/mL (≥ 75 nmol/L).[18,20,21,22]

Reference values measured in an apparently healthy population:

It should be taken into consideration that differences in vitamin D (25-OH) levels may exist with respect to gender, age, season, geographical latitude and ethnic groups.[18,20]

Each laboratory should investigate the transferability of the expected values to its own patient population and if necessary determine its own reference ranges.

Population based reference ranges should not be taken as clinical cutoff to recommend or dissuade from vitamin D supplementation. Guidance for supplementation should be taken from recent literature.[18,19]

A reference range study was conducted with samples from apparently healthy individuals of Caucasian heritage. The age range was 20-77 years. Samples were collected between November and July in northern Germany.

The values given are for information only and may vary from other published data.

			Gender			
	All (n = 453)		Female (n = 252)		Male (n = 201)	
Unit	ng/mL	nmol/L	ng/mL	nmol/L	ng/mL	nmol/L
Mean	20.6	51.5	21.6	54.0	19.4	48.5
2.5th percentile	5.26	13.2	6.23	15.6	4.92	12.3
97.5th percentile	47.0	118	49.9	125	42.7	107

A lower recovery may be found in particular clinical cohorts, for example dialysis patients.[23]

Specific performance data

Representative performance data on the analyzers are given below. Results obtained in individual laboratories may differ.

For peer review only - http://bmjopen.bmj.com/site/about/guidelines.xhtml

ms_05894913190V7.0

Vitamin D total

cobas®

25-Hydroxyvitamin D

Precision

Precision was determined using Elecsys reagents, pooled human sera and controls in a protocol (EP5-A2) of the CLSI (Clinical and Laboratory Standards Institute): 2 runs per day in duplication each for 21 days (n = 84). The following results were obtained:

Elecsys 2010 and **cobas e** 411 analyzers					
			**Repeatability**		
Sample	Mean		SD		CV
	ng/mL	nmol/L	ng/mL	nmol/L	%
HS[a)] 1	6.76	16.9	0.525	1.31	7.8
HS 2	15.0	37.5	0.770	1.93	5.1
HS 3	28.0	70.0	0.860	2.15	3.1
HS 4	67.0	168	1.15	2.88	1.7
PC[b)] Varia 1	19.9	49.8	0.948	2.37	4.8
PC Varia 2	38.3	95.8	1.05	2.63	2.7

a) HS = human serum

b) PC = PreciControl

Elecsys 2010 and **cobas e** 411 analyzers					
			**Intermediate precision**		
Sample	Mean		SD		CV
	ng/mL	nmol/L	ng/mL	nmol/L	%
HS 1	6.76	16.9	0.724	1.81	10.7
HS 2	15.0	37.5	1.28	3.20	8.5
HS 3	28.0	70.0	1.46	3.65	5.2
HS 4	67.0	168	1.46	3.65	2.2
PC Varia 1	19.9	49.8	1.23	3.08	6.2
PC Varia 2	38.3	95.8	1.41	3.53	3.7

MODULAR ANALYTICS E170, **cobas e** 601 and **cobas e** 602 analyzers					
			**Repeatability**		
Sample	Mean		SD		CV
	ng/mL	nmol/L	ng/mL	nmol/L	%
HS 1	8.35	20.9	0.567	1.42	6.8
HS 2	15.8	39.5	0.824	2.06	5.2
HS 3	28.3	70.8	1.11	2.78	3.9
HS 4	69.6	174	1.50	3.75	2.2
PC Varia 1	20.2	50.5	0.924	2.31	4.6
PC Varia 2	39.6	99.0	1.06	2.65	2.7

MODULAR ANALYTICS E170, **cobas e** 601 and **cobas e** 602 analyzers					
			**Intermediate precision**		
Sample	Mean		SD		CV
	ng/mL	nmol/L	ng/mL	nmol/L	%
HS 1	8.35	20.9	1.10	2.75	13.1
HS 2	15.8	39.5	1.18	2.95	7.5
HS 3	28.3	70.8	1.83	4.58	6.5
HS 4	69.6	174	2.37	5.93	3.4
PC Varia 1	20.2	50.5	0.954	2.39	4.7
PC Varia 2	39.6	99.0	1.38	3.45	3.5

Method comparison

1) A comparison of the Elecsys Vitamin D total assay (y) using samples measured with LC-MS/MS (x) gave the following correlations (ng/mL):

Number of samples measured: 903

Passing/Bablok[24] y = 1.09x - 0.510

Pearson r = 0.894

The sample concentrations were between approximately 3 ng/mL (7.5 nmol/L) and 81 ng/mL (203 nmol/L).

2) A comparison of the Elecsys Vitamin D total assay (y) using samples measured with a commercially available vitamin D (25-OH) immunoassay (x) gave the following correlations (ng/mL):

Number of samples measured: 451

Passing/Bablok[24] y = 1.29x + 1.71

Pearson r = 0.803

The sample concentrations were between approximately 5 ng/mL (12.5 nmol/L) and 81 ng/mL (203 nmol/L).

Analytical specificity

The specificity was assessed at 50 % B_0 and the results are summarized in the following table:

Cross-reactant	Cross-reactivity (%)
25-hydroxyvitamin D_3	100
25-hydroxyvitamin D_2	92
24,25-dihydroxyvitamin D_3	149
C3-epimer of 25-hydroxyvitamin D_3	91
1,25-dihydroxyvitamin D_3	non detectable
1,25-dihydroxyvitamin D_2	non detectable
Vitamin D_3	non detectable
Vitamin D_2	non detectable

Functional sensitivity

The functional sensitivity is the lowest analyte concentration that can be reproducibly measured with an intermediate precision CV of ≤ 20 %.
8 samples with concentrations between 0.722 ng/mL and 10.1 ng/mL were measured on several days. The functional sensitivity was determined to be 4.01 ng/mL (CV 18.5 %).

References

1 Holick M. Vitamin D: the underappreciated D-lightful hormone that is important for skeletal and cellular health. Curr Opin Endocrinol Diabetes 2002;9(1)87-98.

2 Holick MF. Vitamin D deficiency. N Engl J Med 2007;357:266-281.

3 Houghton LA, Vieth R. The case against ergocalciferol (vitamin D2) as a vitamin supplement. Am J Clin Nutr 2006;84:694-697.

4 Hart GR, Furniss JL, Laurie D, et al. Measurement of vitamin D Status: background, clinical use and methodologies. Clin Lab 2006;52(7-8):335-343.

5 Armas LAG, Hollis BW, Heaney RP. Vitamin D2 is much less effective than Vitamin D3 in humans. J Clin Endocrinol Metab 2004;89(11):5387-5391.

6 Steingrimsdottir L, Gunnarsson O, Indridason OS, et al. Relationship between serum parathyroid hormone levels, vitamin D sufficiency, and calcium intake. JAMA 2005 Nov 9;294(18):2336-2341.

7 Venning G. Recent developments in vitamin D deficiency and muscle weakness among elderly people. BMJ 2005;330:524-526.

8 Lips P. Vitamin D deficiency and secondary hyperparathyroidism in the elderly: consequences for bone loss and fractures and therapeutic implications. Endocr Rev 2001 Aug;22(4):447-501.

9 Souberbielle JC, Lawson-Body E, Hammadi B, et al. The use in clinical practice of parathyroid hormone normative values established in vitamin D-sufficient subjects. J Clin Endocrinol Metab 2003 Aug;88(8):3501-3504.

10 Willett AM. Vitamin D status and its relationship with parathyroid hormone and bone mineral status in older adolescents. Proceeding of the Nutrition Society 2005;64:193-203.

For peer review only - http://bmjopen.bmj.com/site/about/guidelines.xhtml

ms_05894913190V7.0

Vitamin D total

cobas®

25-Hydroxyvitamin D

11 Kuchukk NO, van Schoor NM, Pluijm SM, et al. Vitamin D status, parathyroid function, bone turnover, and BMD in postmenopausal women with osteoporosis: global perspective. J Bone Miner Res 2009;24:693-701.

12 Occupational Safety and Health Standards: Bloodborne pathogens. (29 CFR Part 1910.1030). Fed. Register.

13 Directive 2000/54/EC of the European Parliament and Council of 18 September 2000 on the protection of workers from risks related to exposure to biological agents at work.

14 Lewis JG, Elder PA. Serum 25-OH Vitamin D2 and D3 are Stable under Exaggerated Conditions. Clin Chem 2008;54:1931-1932.

15 Vogeser M, Kyriatsoulis A, Huber E, et al. Candidate Reference Method for the Quantification of Circulating 25-Hydroxyvitamin D3 by Liquid Chromatography-Tandem Mass Spectrometry. Clin Chem 2004;50:1415-1417.

16 Phinney KW. Development of a standard reference material for vitamin D in serum. Am J Clin Nutr 2008;88(suppl):511-512.

17 Bischoff-Ferrari HA, Giovannucci E, Willett WC, et al. Estimation of optimal serum concentrations of 25-hydroxyvitamin D for multiple health outcomes. Am J Clin Nutr 2006;84:18-28.

18 Holick MF. Vitamin D status: measurement, interpretation, and clinical application. Ann Epidemiol 2009;19(2):73-78.

19 KDOQI Clinical Practice Guidelines for Bone Metabolism and Disease in Children With Chronic Kidney Disease. http://www.kidney.org/PROFESSIONALS/kdoqi/guidelines_pedbone/guide8.htm

20 Souberbielle JC, Body JJ, Lappe JM, et al. Vitamin D and musculoskeletal health, cardiocascular disease, autoimmunity and cancer: Recommendations for clinical practice. Autoimmun Rev 2010;9:709-715.

21 Dawson-Hughes B, Heaney RP, Holick MF, et al. Estimates of optimal vitamin D status. Osteoporos Int 2005;16:713-716.

22 Vieth R. Why the minimum desirable serum 25-hydroxyvitamin D level should be 75 nmol/L (30 ng/mL). Best Pract Res Clin Endocrinol Metab 2011;25(4):681-691.

23 Heijboer AC, Blankenstein MA, Kema IP, et al. Accuracy of 6 routine 25-hydroxyvitamin D assays: influence of vitamin D binding protein concentration. Clin Chem 2012;58:543-548.

24 Bablok W, Passing H, Bender R, et al. A general regression procedure for method transformation. Application of linear regression procedures for method comparison studies in clinical chemistry, Part III. J Clin Chem Clin Biochem 1988 Nov;26(11):783-790.

For further information, please refer to the appropriate operator's manual for the analyzer concerned, the respective application sheets, the product information and the Method Sheets of all necessary components (if available in your country).

A point (period/stop) is always used in this Method Sheet as the decimal separator to mark the border between the integral and the fractional parts of a decimal numeral. Separators for thousands are not used.

Symbols

Roche Diagnostics uses the following symbols and signs in addition to those listed in the ISO 15223-1 standard.

CONTENT	Contents of kit
SYSTEM	Analyzers/Instruments on which reagents can be used
REAGENT	Reagent
CALIBRATOR	Calibrator
→	Volume after reconstitution or mixing
GTIN	Global Trade Item Number

COBAS, COBAS E, ELECSYS and PRECICONTROL are trademarks of Roche. INTRALIPID is a trademark of Fresenius Kabi AB.

All other product names and trademarks are the property of their respective owners.

Additions, deletions or changes are indicated by a change bar in the margin.

© 2015, Roche Diagnostics

CE

Roche Diagnostics GmbH, Sandhofer Strasse 116, D-68305 Mannheim
www.roche.com

Roche

For peer review only

For peer review only - http://bmjopen.bmj.com/site/about/guidelines.xhtml

ms_07212771190V1.0

Vitamin B12 II

cobas®

Vitamin B12

REF	Σ	SYSTEM
**07212771** 190	100	Elecsys 2010<br>MODULAR ANALYTICS E170<br>**cobas e** 411<br>**cobas e** 601<br>**cobas e** 602

English

Intended use

Binding assay for the in vitro quantitative determination of vitamin B12 in human serum and plasma.

The **e**lectro**c**hemi**l**uminescence **i**mmuno**a**ssay “ECLIA” is intended for use on Elecsys and **cobas e** immunoassay analyzers.

Summary

Vitamin B12, also referred to as cobalamin, is a complex organometallic compound in which a cobalt atom is situated within a corrin ring. It is a water-soluble vitamin which is synthesized by microorganisms. It cannot be synthesized in the human body and is seldom found in products of plant origin. Main sources of vitamin B12 are meat, fish, eggs and dairy products.[1] The uptake in the gastrointestinal tract depends on intrinsic factor, which is synthesized by the gastric parietal cells, and on the “cubam receptor” in the distal ileum. The most frequent cause of severe vitamin B12 deficiency is a lack of intrinsic factor due to autoimmune atrophic gastritis. The disease is historically called “pernicious anemia”, even though many patients present with mainly neurologic manifestations. Examples of other causes for vitamin B12 deficiency are malabsorption due to gastrectomy, inflammatory bowel disease or dietary deficiency, e.g. in strict vegetarians (vegans).[2]

Vitamin B12 is the cofactor for two enzymes, methionine synthase and methylmalonyl CoA mutase.[2,3] Methionine synthase, located in the cytoplasm, requires vitamin B12 in the form of methylcobalamin and catalyzes the conversion of homocysteine to methionine, an essential amino acid. During this step a methyl group is transferred from methyltetrahydrofolate to the amino acid.[3] This enzyme links the methylation pathway through synthesis of the methyl donor S-Adenosyl methionine and the pathway in which purine and pyrimidine are synthesized via generation of tetrahydrofolate.[3] In the form of 5’-deoxyadenosylcobalamin, vitamin B12 is also required for the mitochondrial enzyme methylmalonyl CoA mutase, which converts methylmalonyl CoA to succinyl CoA. This is a step in the oxidation of odd-chain fatty acids and catabolism of ketogenic amino acids.[3] Thus, vitamin B12 is important for DNA synthesis, regenerating methionine for protein synthesis and methylation, as well as for the development and initial myelination of the central nervous system (CNS) and for the maintenance of normal CNS function.[2,3]

Vitamin B12 deficiencies are common in wealthier countries principally among the elderly and are most prevalent in poorer populations. In general the prevalence increases with age.[4,5]

Vitamin B12 deficiency impacts red blood cell synthesis, resulting in megaloblastic anemia due to abnormal DNA synthesis.[3] In addition it impairs neurological function, in particular demyelination of nerves in part due to abnormal methylation, leading to peripheral neuropathy, dementia, poor cognitive performance, and depression.[3] Other effects of vitamin B12 deficiency or depletion are increased risk of neural tube defects, osteoporosis, cerebrovascular and cardiovascular diseases.[3] Early diagnosis is essential, because of the latent nature of this disorder and the risk of permanent neurological damage.[3,5]

Generally, the primary test performed to confirm the diagnosis of vitamin B12 deficiency is measurement of serum vitamin B12 level.[2] Recent publications suggest that in addition the following biomarkers should be measured to improve the specificity of diagnosis: folate, methylmalonic acid (MMA), homocysteine and holotranscobalamin.[2,5,6,7]

The Elecsys Vitamin B12 II assay employs a competitive test principle using intrinsic factor specific for vitamin B12. Vitamin B12 in the sample competes with the added vitamin B12 labeled with biotin for the binding sites on the ruthenium-labeled intrinsic factor complex[a)].

a) Tris(2,2'-bipyridyl)ruthenium(II)-complex (Ru(bpy)$_3^{2+}$)

Test principle

Competition principle. Total duration of assay: 27 minutes.

- 1st incubation: By incubating the sample (15 µL) with the vitamin B12 pretreatment 1 and pretreatment 2, bound vitamin B12 is released.
- 2nd incubation: By incubating the pretreated sample with the ruthenium labeled intrinsic factor, a vitamin B12-binding protein complex is formed, the amount of which is dependent upon the analyte concentration in the sample.
- 3rd incubation: After addition of streptavidin-coated microparticles and vitamin B12 labeled with biotin, the still-vacant sites of the ruthenium labeled intrinsic factor become occupied, with formation of a ruthenium labeled intrinsic factor vitamin B12 biotin complex. The entire complex becomes bound to the solid phase via interaction of biotin and streptavidin.
- The reaction mixture is aspirated into the measuring cell where the microparticles are magnetically captured onto the surface of the electrode. Unbound substances are then removed with ProCell/ProCell M. Application of a voltage to the electrode then induces chemiluminescent emission which is measured by a photomultiplier.
- Results are determined via a calibration curve which is instrument-specifically generated by 2-point calibration and a master curve provided via the reagent barcode.

Reagents - working solutions

The reagent rackpack (M, R1, R2) and the pretreatment reagents (PT1, PT2) are labeled as B12 II.

PT1 Pretreatment reagent 1 (white cap), 1 bottle, 4 mL:
Dithiothreitol 1.028 g/L; stabilizer, pH 5.5.

PT2 Pretreatment reagent 2 (gray cap), 1 bottle, 4 mL:
Sodium hydroxide 40 g/L; sodium cyanide 2.205 g/L.

M Streptavidin-coated microparticles (transparent cap), 1 bottle, 6.5 mL:
Streptavidin-coated microparticles 0.72 mg/mL; preservative.

R1 Intrinsic factor~Ru(bpy)$_3^{2+}$ (gray cap), 1 bottle, 10 mL:
Ruthenium labeled recombinant porcine intrinsic factor 4 µg/L; cobinamide dicyanide 15 µg/L; stabilizer; human serum albumin; phosphate buffer, pH 5.5; preservative.

R2 Vitamin B12~biotin (black cap), 1 bottle, 8.5 mL:
Biotinylated vitamin B12 25 µg/L; biotin 3 µg/L; phosphate buffer, pH 7.0; preservative.

Precautions and warnings

For in vitro diagnostic use.
Exercise the normal precautions required for handling all laboratory reagents.
Disposal of all waste material should be in accordance with local guidelines.
Safety data sheet available for professional user on request.

This kit contains components classified as follows in accordance with the Regulation (EC) No. 1272/2008:

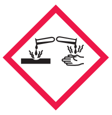

Danger

For peer review only - http://bmjopen.bmj.com/site/about/guidelines.xhtml

ms_07212771190V1.0

Vitamin B12 II

cobas®

Vitamin B12

H290 May be corrosive to metals.

H314 Causes severe skin burns and eye damage.

H412 Harmful to aquatic life with long lasting effects.

Prevention:

P234 Keep only in original container.

P264 Wash skin thoroughly after handling.

P273 Avoid release to the environment.

P280 Wear protective gloves/ protective clothing/ eye protection/ face protection.

Response:

P301 + P330 + P331 IF SWALLOWED: Rinse mouth. Do NOT induce vomiting.

P303 + P361 + P353 IF ON SKIN (or hair): Remove/Take off immediately all contaminated clothing. Rinse skin with water/shower.

P304 + P340 IF INHALED: Remove person to fresh air and keep comfortable for breathing.

P305 + P351 + P338 IF IN EYES: Rinse cautiously with water for several minutes. Remove contact lenses, if present and easy to do. Continue rinsing.

P310 Immediately call a POISON CENTER or doctor/physician.

P363 Wash contaminated clothing before reuse.

P390 Absorb spillage to prevent material damage.

Storage:

P405 Store locked up.

P406 Store in corrosive resistant stainless steel container with a resistant inner liner.

Disposal:

P501 Dispose of contents/container to an approved waste disposal plant.

Product safety labeling primarily follows EU GHS guidance.

Contact phone: all countries: +49-621-7590

All human material should be considered potentially infectious. All products derived from human blood are prepared exclusively from the blood of donors tested individually and shown to be free from HBsAg and antibodies to HCV and HIV. The testing methods applied were FDA-approved or cleared in compliance with the European Directive 98/79/EC, Annex II, List A.

However, as no testing method can rule out the potential risk of infection with absolute certainty, the material should be handled with the same level of care as a patient specimen. In the event of exposure, the directives of the responsible health authorities should be followed.[8,9]

Avoid foam formation in all reagents and sample types (specimens, calibrators and controls).

Reagent handling

The reagents in the kit have been assembled into a ready-for-use unit that cannot be separated.

All information required for correct operation is read in from the respective reagent barcodes.

Storage and stability

Store at 2-8 °C.

Do not freeze.

Store the Elecsys reagent kit **upright** in order to ensure complete availability of the microparticles during automatic mixing prior to use.

Stability:	
unopened at 2-8 °C	up to the stated expiration date
after opening at 2-8 °C	84 days (12 weeks)
on the analyzers	35 days (5 weeks) onboard<br>or<br>60 days when stored alternatively in the refrigerator and on the analyzer, with the total time onboard on the analyzer not exceeding<br>10 x 8 hours

Specimen collection and preparation

Only the specimens listed below were tested and found acceptable.

Serum collected using standard sampling tubes or tubes containing separating gel.

Na-heparin, Li-heparin, K_2-EDTA and K_3-EDTA plasma. Li-heparin plasma tubes containing separating gel can be used.

Criterion: Slope 0.9-1.1 + intercept within < ± 2x Limit of Blank (LoB) + coefficient of correlation ≥ 0.95.

Stable for 2 hours at 15-25 °C, 48 hours at 2-8 °C, 56 days at (-15)-(-25) °C. Freeze once only.

Stability of serum obtained with separating tubes: 24 hours at 2-8 °C (note the data provided by the tube manufacturer).

The sample types listed were tested with a selection of sample collection tubes that were commercially available at the time of testing, i.e. not all available tubes of all manufacturers were tested. Sample collection systems from various manufacturers may contain differing materials which could affect the test results in some cases. When processing samples in primary tubes (sample collection systems), follow the instructions of the tube manufacturer.

Centrifuge samples containing precipitates before performing the assay.

Do not use heat-inactivated samples.

Avoid hemolysis.

Do not use samples and controls stabilized with azide.

Vitamin B12 determinations should be performed on serum or plasma samples from fasting patients.

Note: Samples with extremely high total protein concentrations (e.g. patients suffering from Waldenström's macroglobulinemia) are not suitable for use in this assay, since they may lead to the formation of protein gel in the assay cup. Processing protein gel may cause a run abort. The critical protein concentration is dependent upon the individual sample composition. The formation of protein gel was seen in samples (spiked with human IgG or human serum albumin) having a total protein concentration > 160 g/L.

Ensure the samples, calibrators and controls are at 20-25 °C prior to measurement.

Due to possible evaporation effects, samples, calibrators and controls on the analyzers should be analyzed/measured within 2 hours.

Materials provided

See “Reagents – working solutions” section for reagents.

Materials required (but not provided)

- REF 07212780190, Vitamin B12 II CalSet, for 4 x 1 mL
- REF 05618860190, PreciControl Varia, for 2 x 3 mL each of PreciControl Varia 1 and 2
- REF 11732277122, Diluent Universal, 2 x 16 mL sample diluent or REF 03183971122, Diluent Universal, 2 x 36 mL sample diluent
- General laboratory equipment
- Elecsys 2010, MODULAR ANALYTICS E170 or **cobas e** analyzer

Accessories for Elecsys 2010 and **cobas e** 411 analyzers:

- REF 11662988122, ProCell, 6 x 380 mL system buffer
- REF 11662970122, CleanCell, 6 x 380 mL measuring cell cleaning solution

For peer review only - http://bmjopen.bmj.com/site/about/guidelines.xhtml

ms_07212771190V1.0

Vitamin B12 II

cobas®

Vitamin B12

- REF 11930346122, Elecsys SysWash, 1 x 500 mL washwater additive
- REF 11933159001, Adapter for SysClean
- REF 11706802001, Elecsys 2010 AssayCup, 60 x 60 reaction vessels
- REF 11706799001, Elecsys 2010 AssayTip, 30 x 120 pipette tips

Accessories for MODULAR ANALYTICS E170, **cobas e** 601 and **cobas e** 602 analyzers:

- REF 04880340190, ProCell M, 2 x 2 L system buffer
- REF 04880293190, CleanCell M, 2 x 2 L measuring cell cleaning solution
- REF 03023141001, PC/CC-Cups, 12 cups to prewarm ProCell M and CleanCell M before use
- REF 03005712190, ProbeWash M, 12 x 70 mL cleaning solution for run finalization and rinsing during reagent change
- REF 03004899190, PreClean M, 5 x 600 mL detection cleaning solution
- REF 12102137001, AssayTip/AssayCup Combimagazine M, 48 magazines x 84 reaction vessels or pipette tips, waste bags
- REF 03023150001, WasteLiner, waste bags
- REF 03027651001, SysClean Adapter M

Accessories for all analyzers:

- REF 11298500316, ISE Cleaning Solution/Elecsys SysClean, 5 x 100 mL system cleaning solution

Assay

For optimum performance of the assay follow the directions given in this document for the analyzer concerned. Refer to the appropriate operator's manual for analyzer-specific assay instructions.

Resuspension of the microparticles takes place automatically prior to use. Read in the test-specific parameters via the reagent barcode. If in exceptional cases the barcode cannot be read, enter the 15-digit sequence of numbers.

MODULAR ANALYTICS E170, **cobas e** 601 and **cobas e** 602 analyzers: PreClean M solution is necessary.

Bring the cooled reagents to approximately 20 °C and place on the reagent disk (20 °C) of the analyzer. Avoid foam formation. The system automatically regulates the temperature of the reagents and the opening/closing of the bottles.

Calibration

Traceability: This method has been standardized against the Vitamin B12 assay (REF 04745736).

Every Elecsys reagent set has a barcoded label containing specific information for calibration of the particular reagent lot. The predefined master curve is adapted to the analyzer using the relevant CalSet.

Calibration frequency: Calibration must be performed once per reagent lot using fresh reagent (i.e. not more than 24 hours since the reagent kit was registered on the analyzer). Renewed calibration is recommended as follows:

- after 1 month (28 days) when using the same reagent lot
- after 7 days (when using the same reagent kit on the analyzer)
- as required: e.g. quality control findings outside the defined limits

Quality control

For quality control, use PreciControl Varia.

In addition, other suitable control material can be used.

Controls for the various concentration ranges should be run individually at least once every 24 hours when the test is in use, once per reagent kit, and following each calibration.

The control intervals and limits should be adapted to each laboratory's individual requirements. Values obtained should fall within the defined limits. Each laboratory should establish corrective measures to be taken if values fall outside the defined limits.

Follow the applicable government regulations and local guidelines for quality control.

Calculation

The analyzer automatically calculates the analyte concentration of each sample (either in pmol/L or pg/mL).

Conversion factors:	pmol/L x 1.36 = pg/mL
	pg/mL x 0.738 = pmol/L

Limitations - interference

The assay is unaffected by icterus (bilirubin ≤ 1112 µmol/L or ≤ 65 mg/dL), hemolysis (Hb ≤ 0.025 mmol/L or ≤ 0.04 g/dL), lipemia (triglycerides ≤ 17.1 mmol/L or ≤ 1500 mg/dL), biotin (≤ 205 nmol/L or ≤ 50 ng/mL), IgG ≤ 28 g/L, IgA ≤ 16 g/L and IgM ≤ 10 g/L.

Criterion: Recovery within ± 10 % of initial value with samples > 200 pg/mL and ≤ ± 20 pg/mL with samples ≤ 200 pg/mL.

Samples should not be taken from patients receiving therapy with high biotin doses (i.e. > 5 mg/day) until at least 8 hours following the last biotin administration.

No interference was observed from rheumatoid factors up to a concentration of 1500 IU/mL.

In vitro tests were performed on 16 commonly used pharmaceuticals. No interference with the assay was found.

In rare cases, interference due to extremely high titers of antibodies to analyte-specific antibodies, streptavidin or ruthenium can occur. These effects are minimized by suitable test design.

Because intrinsic factor is typically used as the binding protein in serum vitamin B12 assays, anti-intrinsic factor antibodies (which are common in pernicious anemia) can lead to elevated vitamin B12 measurement values.[2,10,11] The Elecsys Vitamin B12 II assay is designed to avoid interference due to anti-intrinsic factor antibodies.[12]

For diagnostic purposes, the results should always be assessed in conjunction with the patient's medical history, clinical examination and other findings.

Note: The presence of immunoglobulin-vitamin B12 complexes may cause unexpectedly high values of vitamin B12.[13,14]

Limits and ranges

Measuring range

50.0-2000 pg/mL or 36.9-1476 pmol/L (defined by the Limit of Blank and the maximum of the master curve). Values below the Limit of Blank are reported as < 50.0 pg/mL or < 36.9 pmol/L. Values above the measuring range are reported as > 2000 pg/mL or > 1476 pmol/L.

Lower limits of measurement

Limit of Blank (LoB), Limit of Detection (LoD) and Limit of Quantitation (LoQ)

Limit of Blank = 50 pg/mL (36.9 pmol/L)

Limit of Detection = 100 pg/mL (73.8 pmol/L)

Limit of Quantitation = 150 pg/mL (111 pmol/L) with a allowable imprecision of ≤ 20 %

The Limit of Blank, Limit of Detection and Limit of Quantitation were determined in accordance with the CLSI (Clinical and Laboratory Standards Institute) EP17-A requirements.

The Limit of Blank is the 95th percentile value from n ≥ 60 measurements of analyte-free samples over several independent series. The Limit of Blank corresponds to the concentration below which analyte-free samples are found with a probability of 95 %.

The Limit of Detection is determined based on the Limit of Blank and the standard deviation of low concentration samples. The Limit of Detection corresponds to the lowest analyte concentration which can be detected (value above the Limit of Blank with a probability of 95 %).

The Limit of Quantitation is defined as the lowest amount of analyte in a sample that can be accurately quantitated with a allowable imprecision of ≤ 20 %.

It has been determined using low concentration vitamin B12 samples.

Dilution

Samples with vitamin B12 concentrations above the measuring range can be manually diluted 1:2 with Diluent Universal. The concentration of the diluted sample must be > 738 pmol/L or > 1000 pg/mL. After manual dilution, multiply the results by the dilution factor 2.

Note: Sample-dependent non-linearity upon dilution is seen with samples having analyte levels beyond the measuring range. As Diluent Universal may contain low levels of endogenous vitamin B12, it is recommended that linearity studies be performed using a known low analyte-containing serum

For peer review only - http://bmjopen.bmj.com/site/about/guidelines.xhtml

ms_07212771190V1.0

Vitamin B12 II

Vitamin B12

cobas®

pool. Samples outside the measuring range can be diluted 1:2 with Diluent Universal; the effect of endogenous vitamin B12 concentration is insignificant at these levels.

Expected values

Because differences may exist with respect to population and dietary status, it is recommended that normal ranges be determined by each laboratory over a suitable period of time and in a statistically significant number of assays before clinical significance is attached to the results of these tests.

The values shown below were performed on samples from an apparently healthy population, using the Elecsys Vitamin B12 II assay. The calculation is based on 135 sera (68 men, 67 women). The age range was between 20 and 78 years. Pregnant women were excluded. The reference population was selected according to normal homocysteine values.

N	Median		Range (2.5^{th}-97.5^{th} percentile)	
	pg/mL	pmol/L	pg/mL	pmol/L
135	425	314	197-771	145-569

These values should only be used as guidelines.

Each laboratory should investigate the transferability of the expected values to its own patient population and if necessary determine its own reference ranges.

Specific performance data

Representative performance data on the analyzers are given below. Results obtained in individual laboratories may differ.

Precision

Precision was determined using Elecsys reagents, pooled human sera and controls in a protocol (EP5-A2) of the CLSI (Clinical and Laboratory Standards Institute): 2 runs per day in duplication each for 21 days (n = 84). The following results were obtained:

Elecsys 2010 and **cobas e** 411 analyzers					
		**Repeatability**		**Intermediate precision**	
Sample	Mean	SD	CV	SD	CV
	pg/mL	pg/mL	%	pg/mL	%
Human serum 1	176	8.86	5.0	12.7	7.2
Human serum 2	405	13.0	3.2	17.5	4.3
Human serum 3	960	19.7	2.1	31.0	3.2
Human serum 4	1230	27.4	2.2	46.4	3.8
Human serum 5	1940	40.9	2.1	72.6	3.7
PreciControl Varia1	447	12.2	2.7	18.6	4.2
PreciControl Varia2	934	20.2	2.2	38.4	4.1

Elecsys 2010 and **cobas e** 411 analyzers					
		**Repeatability**		**Intermediate precision**	
Sample	Mean	SD	CV	SD	CV
	pmol/L	pmol/L	%	pmol/L	%
Human serum 1	130	6.54	5.0	9.37	7.2
Human serum 2	299	9.59	3.2	12.9	4.3
Human serum 3	708	14.5	2.1	22.9	3.2
Human serum 4	908	20.2	2.2	34.2	3.8
Human serum 5	1432	30.2	2.1	53.6	3.7
PreciControl Varia1	330	9.00	2.7	13.7	4.2
PreciControl Varia2	689	14.9	2.2	28.3	4.1

MODULAR ANALYTICS E170, **cobas e** 601 and **cobas e** 602 analyzers					
		**Repeatability**		**Intermediate precision**	
Sample	Mean	SD	CV	SD	CV
	pg/mL	pg/mL	%	pg/mL	%
Human serum 1	176	5.84	3.3	9.14	5.2
Human serum 2	407	8.24	2.0	12.7	3.1
Human serum 3	1010	13.2	1.3	21.1	2.1
Human serum 4	1230	19.8	1.6	28.8	2.3
Human serum 5	1890	29.8	1.6	41.5	2.2
PreciControl Varia1	448	7.16	1.6	15.3	3.4
PreciControl Varia2	917	12.0	1.3	27.8	3.0

MODULAR ANALYTICS E170, **cobas e** 601 and **cobas e** 602 analyzers					
		**Repeatability**		**Intermediate precision**	
Sample	Mean	SD	CV	SD	CV
	pmol/L	pmol/L	%	pmol/L	%
Human serum 1	130	4.31	3.3	6.75	5.2
Human serum 2	300	6.08	2.0	9.37	3.1
Human serum 3	745	9.74	1.3	15.6	2.1
Human serum 4	908	14.6	1.6	21.3	2.3
Human serum 5	1395	22.0	1.6	30.6	2.2
PreciControl Varia1	331	5.28	1.6	11.3	3.4
PreciControl Varia2	677	8.86	1.3	20.5	3.0

Method comparison

a) A comparison of the Elecsys Vitamin B12 assay (calibrated with Vitamin B12 CalSet II; x) and the Elecsys Vitamin B12 II assay (calibrated with Vitamin B12 II CalSet; y) using clinical samples gave the following correlations (pg/mL):

Number of samples measured: 100

Passing/Bablok[15]	Linear regression
y = 0.952x + 15.1	y = 0.957x + 11.6
τ = 0.977	r = 0.999

The sample concentrations were between 69 and 1890 pg/mL (51 and 1395 pmol/L).

b) A comparison of the Elecsys Vitamin B12 II assay (y) and a commercially available method (x) using clinical samples gave the following correlations (pg/mL):

Number of samples measured: 106

Passing/Bablok[15]	Linear regression
y = 0.923x + 4.90	y = 0.881x + 27.6
τ = 0.952	r = 0.993

The sample concentrations were between 182 and 1797 pg/mL (134 and 1326 pmol/L).

c) A comparison of the Elecsys Vitamin B12 II assay on the **cobas e** 601 analyzer (y) and the Elecsys Vitamin B12 II assay on the **cobas e** 411 analyzer (x) using clinical samples gave the following correlations (pg/mL):

Number of samples measured: 117

Passing/Bablok[15]	Linear regression
y = 1.01x - 2.77	y = 1.01x + 3.22
τ = 0.933	r = 0.995

The sample concentrations were between 56 and 1887 pg/mL (41 and 1393 pmol/L).

For peer review only - http://bmjopen.bmj.com/site/about/guidelines.xhtml

ms_07212771190V1.0

Vitamin B12 II

cobas®

Vitamin B12

Analytical specificity

The following cross-reactivities were found, tested with vitamin B12 concentrations of 129 pg/mL and 550 pg/mL.

Cross-reactant	Maximum concentration tested ng/mL	Cross-reactivity %
Cobinamide dicyanide	210	0.003

References

1 Thomas L. Clinical Laboratory Diagnostics: Use and Assessment of Clinical Laboratory Results; 1st Edition, Frankfurt/Main: TH-Books-Verl.-Ges.,1998:424-431.

2 Stabler SP. Vitamin B12 deficiency. N Engl J Med 2013;368:149-160.

3 Allen LH. Vitamin B-12. Adv Nutr 2012;3(1):54-55. doi: 10.3945/an.111.001370. Epub 2012 Jan 5.

4 Allen LH. How common is vitamin B-12 deficiency? Am J Clin Nutr 2009;89(2):693S-696S. Epub 2008 Dec 30.

5 Chatthanawaree W. Biomarkers of cobalamin (vitamin B12) deficiency and its application. J Nutr Health Aging 2011 Mar;15(3):227-313.

6 Yetley EA, Pfeiffer CM, Phinney KW, et al., Biomarkers of vitamin B-12 status in NHANES: a roundtable summary. Am J Clin Nutr 2011 Jul;94(1):313S-321S.

7 Hvas AM, Nexo E. Diagnosis and treatment of vitamin B12 deficiency - an update. Haematologica 2006;91(11):1506-1512.

8 Occupational Safety and Health Standards: bloodborne pathogens. (29 CFR Part 1910.1030). Fed. Register.

9 Directive 2000/54/EC of the European Parliament and Council of 18 September 2000 on the protection of workers from risks related to exposure to biological agents at work.

10 Yang DT, Cook RJ. Spurious elevations of vitamin B12 with pernicious anemia. N Engl J Med 2012;366:1742-1743.

11 Carmel R, Agrawal YP. Failures of cobalamin assays in pernicious anemia. N Engl J Med 2012;367:385-386. [Erratum, N Engl J Med 2012;367:976.]

12 Schilling KA, Wiesgigl M. The Elecsys® Vitamin B12 assay is not affected by anti-intrinsic factor antibodies. Clin Chem Lab Med 2013 Jun 29;51(11):e251-e252.

13 Jeffery J, Millar H, MacKenzie P, et al. An IgG complexed form of vitamin B12 is a common cause of elevated serum concentrations. Clin Biochem 2010 Jan;43(1-2):82-88. doi: 10.1016/j.clinbiochem.2009.08.022. Epub 2009 Sep 8.

14 Bowen RA, Drake SK, Vanjani R, et al. Markedly increased vitamin B12 concentrations attributable to IgG-IgM-vitamin B12 immune complexes. Clin Chem 2006;52(11):2107-2114.

15 Bablok W, Passing H, Bender R, et al. A general regression procedure for method transformation. Application of linear regression procedures for method comparison studies in clinical chemistry, Part III. J Clin Chem Clin Biochem 1988 Nov;26(11):783-790.

For further information, please refer to the appropriate operator's manual for the analyzer concerned, the respective application sheets, the product information and the Method Sheets of all necessary components (if available in your country).

A point (period/stop) is always used in this Method Sheet as the decimal separator to mark the border between the integral and the fractional parts of a decimal numeral. Separators for thousands are not used.

Symbols

Roche Diagnostics uses the following symbols and signs in addition to those listed in the ISO 15223-1 standard.

CONTENT	Contents of kit
SYSTEM	Analyzers/Instruments on which reagents can be used
REAGENT	Reagent
CALIBRATOR	Calibrator
→	Volume after reconstitution or mixing
GTIN	Global Trade Item Number

COBAS, COBAS E, ELECSYS and PRECICONTROL are trademarks of Roche. INTRALIPID is a trademark of Fresenius Kabi AB.

All other product names and trademarks are the property of their respective owners.

Additions, deletions or changes are indicated by a change bar in the margin.

© 2015, Roche Diagnostics

CE

Roche Diagnostics GmbH, Sandhofer Strasse 116, D-68305 Mannheim
www.roche.com

Roche

For peer review only

For peer review only - http://bmjopen.bmj.com/site/about/guidelines.xhtml

0107227841190c501V1.0

STFR2

cobas®

Tina-quant Soluble Transferrin Receptor II

Order information

REF	CONTENT		Analyzer(s) on which **cobas c** pack(s) can be used
07227841190	Tina-quant Soluble Transferrin Receptor II(100 tests)	System-ID 07 7473 1	**cobas c** 311, **cobas c** 501/502
08753776190	Calibrator sTfR II (3 x 1 mL)	Codes 697	
08278202190	ControlSet sTfR II		
	Level I (3 x 1 mL)	Level I Code 153	
	Level II (3 x 1 mL)	Level II Code 154	
04489357190	Diluent NaCl 9 % (50 mL)	System-ID 07 6869 3	

English

System information

For **cobas c** 311/501 analyzers:

STFR2: ACN 439

For **cobas c** 502 analyzer:

STFR2: ACN 8439

Intended use

In vitro test for the quantitative determination of soluble transferrin receptor (sTfR) in human serum and plasma on **cobas c** systems.

Summary[1,2,3,4,5,6,7]

The transferrin receptor is an integral membrane glycoprotein having a molecular weight of 190 kilodalton (kDa). It consists of two identical subunits linked by disulfide bridges. Each of the monomers has an 85 kDa C-terminal component which can bind an iron-laden transferrin molecule. Proteolysis leads to the soluble form of the transferrin receptor (sTfR). In plasma, the soluble transferrin receptor is present in the form of a complex with transferrin having a molecular weight of approximately 320 kD. The serum concentration of sTfR is directly proportional to the concentration of the receptor on the membrane.

The uptake of iron by the body's cells is controlled by expression of the transferrin receptor (TfR). If the intracellular iron stores are exhausted - corresponding to a ferritin concentration of less than 12 µg/L - then more TfR is expressed. The affinity of the transferrin receptor to transferrin depends on the latter's loading state. As 80-95 % of the transferrin receptor molecules are localized on erythropoietic cells, the TfR concentration (and hence also the sTfR concentration) reflects the iron requirement of these cells. When iron deficiency exists, the sTfR concentration in serum rises even before the hemoglobin concentration is significantly depressed. The sTfR concentration can therefore describe the functional iron status while ferritin reflects the iron storage status. A precise assessment of the iron status can be obtained by determining the sTfR index
(= sTfR concentration/log ferritin concentration).

As - in contrast to ferritin - the concentration of sTfR is not affected by acute-phase reactions, acute liver function disorders or malignant tumors, it is possible to differentiate between anemia of chronic disease (ACD) and iron deficiency anemia (IDA). Elevated sTfR values are also found in polycythemia, hemolytic anemia, thalassemia, hereditary spherocytosis, sickle cell anemia, megaloblastic anemia, myelodysplastic syndrome and vitamin B_{12} deficiency. Elevated sTfR concentrations occur during pregnancy when there is a deficiency of functional iron.

Parameter	Change	IDA	ACD	IDA + ACD
Ferritin	iron stores	↓	↑	— or ↑
TIBC/TRSF	iron status	↑	↓	↑ or —
Serum iron	iron status	↓	↓	↓
sTfR	functional iron deficiency	↑	—	↑

↓ decreased, ↑ increased, — unchanged

Test principle[8]

Particle enhanced immunoturbidimetric assay.

Human soluble transferrin receptor agglutinates with latex particles coated with anti-soluble transferrin receptor antibodies. The precipitate is determined photometrically.

Reagents - working solutions

R1 TRIS buffer: with bovine serum albumin; preservatives

R2 Latex particles coated with monoclonal anti-human sTfR antibodies (mouse) in glycine buffer; preservative

R1 is in position B and R2 is in position C.

Precautions and warnings

For in vitro diagnostic use.
Exercise the normal precautions required for handling all laboratory reagents.
Disposal of all waste material should be in accordance with local guidelines.
Safety data sheet available for professional user on request.

This kit contains components classified as follows in accordance with the Regulation (EC) No. 1272/2008:

Contains 2-methyl-2H-isothiazol-3-one hydrochloride

EUH 208 May produce an allergic reaction.

Product safety labeling follows EU GHS guidance.

Reagent handling

Ready for use

Carefully invert reagent container several times prior to use to ensure that the reagent components are mixed.

Storage and stability

STFR2

Shelf life at 2-8 °C:	See expiration date on **cobas c** pack label.
On-board in use and refrigerated on the analyzer:	26 weeks

Diluent NaCl 9 %

Shelf life at 2-8 °C:	See expiration date on **cobas c** pack label.
On-board in use and refrigerated on the analyzer:	12 weeks

Specimen collection and preparation

For specimen collection and preparation only use suitable tubes or collection containers.

Only the specimens listed below were tested and found acceptable.
Serum.
Plasma: Heparin (Li-, Na-, $NH4^{+}$-) plasma

The sample types listed were tested with a selection of sample collection tubes that were commercially available at the time of testing, i.e. not all available tubes of all manufacturers were tested. Sample collection systems from various manufacturers may contain differing materials which could affect the test results in some cases. When processing samples in primary tubes (sample collection systems), follow the instructions of the tube manufacturer.

Centrifuge samples containing precipitates before performing the assay.

See the limitations and interferences section for details about possible sample interferences.

Stability:	6 days at 15-25 °C
	15 days at 2-8 °C
	13 weeks at -20 °C (±5 °C) (freeze only once)

For peer review only - http://bmjopen.bmj.com/site/about/guidelines.xhtml

0107227841190c501V1.0

STFR2

Tina-quant Soluble Transferrin Receptor II

cobas®

Sample stability claims were established by experimental data by the manufacturer or based on reference literature and only for the temperatures/time frames as stated in the method sheet. It is the responsibility of the individual laboratory to use all available references and/or its own studies to determine specific stability criteria for its laboratory.

Materials provided

See “Reagents – working solutions” section for reagents.

Materials required (but not provided)

- See “Order information” section
- General laboratory equipment

Assay

For optimum performance of the assay follow the directions given in this document for the analyzer concerned. Refer to the appropriate operator’s manual for analyzer-specific assay instructions.

The performance of applications not validated by Roche is not warranted and must be defined by the user.

Application for serum and plasma

cobas c 311 **test definition**

Assay type	2-Point End		
Reaction time/Assay points	10 / 8-21		
Wavelength (sub/main)	800/570 nm		
Reaction direction	Increase		
Unit	mg/L (mg/dL, nmol/L)		
Reagent pipetting		Diluent (H_2O)	
R1	100 µL	-	
R2	40 µL	-	
Sample volumes	*Sample*	*Sample dilution*	
		Sample	*Diluent (NaCl)*
Normal	2 µL	-	-
Decreased	2 µL	25 µL	75 µL
Increased	2 µL		

cobas c 501 **test definition**

Assay type	2-Point End		
Reaction time/Assay points	10 / 13-30		
Wavelength (sub/main)	800/570 nm		
Reaction direction	Increase		
Unit	mg/L (mg/dL, nmol/L)		
Reagent pipetting		Diluent (H_2O)	
R1	100 µL	-	
R2	40 µL	-	
Sample volumes	*Sample*	*Sample dilution*	
		Sample	*Diluent (NaCl)*
Normal	2 µL	-	-
Decreased	2 µL	25 µL	75 µL
Increased	2 µL		

cobas c 502 **test definition**

Assay type	2-Point End		
Reaction time/Assay points	10 / 13-30		
Wavelength (sub/main)	800/570 nm		
Reaction direction	Increase		
Unit	mg/L (mg/dL, nmol/L)		
Reagent pipetting		Diluent (H_2O)	
R1	100 µL	-	
R2	40 µL	-	
Sample volumes	*Sample*	*Sample dilution*	
		Sample	*Diluent (NaCl)*
Normal	2 µL	-	-
Decreased	2 µL	25 µL	75 µL
Increased	2 µL		

Calibration

Calibrators	S1: H_2O
	S2-S6: Calibrator sTfR II
Calibration mode	Non-linear
Calibration frequency	Full calibration<br>• after reagent lot change<br>• after 12 weeks on-board the analyzer<br>• after 6 months when using a single reagent lot<br>• as required following quality control procedures

Calibration interval may be extended based on acceptable verification of calibration by the laboratory.

Traceability: This method has been standardized against an in-house reference preparation.

Quality control

For quality control, use control materials as listed in the "Order information" section.

In addition, other suitable control material can be used.

The control intervals and limits should be adapted to each laboratory’s individual requirements. Values obtained should fall within the defined limits. Each laboratory should establish corrective measures to be taken if values fall outside the defined limits.

Follow the applicable government regulations and local guidelines for quality control.

Calculation

cobas c systems automatically calculate the analyte concentration of each sample in the unit mg/L (mg/dL, nmol/L)

Conversion factors:	mg/L x 11.8 = nmol/L[9,a)]
	mg/L x 0.1 = mg/dL

a) Based on a molecular mass of 85 kDa for circulating transferrin receptor.

Limitations – interference

Criterion: Recovery within ± 0.2 mg/L (2.36 nmol/L) of initial values of samples ≤ 2 mg/L (23.6 nmol/L) and within ± 10 % for samples > 2 mg/L.

Icterus:[10] No significant interference up to an I index of 60 for conjugated and unconjugated bilirubin (approximate conjugated and unconjugated bilirubin concentration: 1026 µmol/L or 60 mg/dL).

Hemolysis:[10] No significant interference up to an H index of 1000 (approximate hemoglobin concentration: 622 µmol/L or 1000 mg/dL).

Lipemia (Intralipid):[10] No significant interference up to an L index of 2000. There is poor correlation between the L index (corresponds to turbidity) and triglycerides concentration.

Rheumatoid factors: No significant interference from rheumatoid factors up to a concentration of 1200 IU/mL.

High dose hook-effect: No false result occurs up to an sTfR concentration of 80 mg/L (944 nmol/L).

The antibodies are specific for sTfR. There is no cross-reactivity with diferrotransferrin, apotransferrin or ferritin under the assay conditions.

Drugs: No interference was found at therapeutic concentrations using common drug panels.[11,12]

For peer review only

For peer review only - http://bmjopen.bmj.com/site/about/guidelines.xhtml

0107227841190c501V1.0

STFR2

Tina-quant Soluble Transferrin Receptor II

cobas®

In very rare cases, gammopathy, in particular type IgM (Waldenström's macroglobulinemia), may cause unreliable results.[13]

In very rare cases, patient samples may contain particle agglutinating proteins (e.g. heterophilic antibodies or antibodies due to abnormal immunoglobulin synthesis, such as gammopathies like MGUS* or Waldenström's macroglobulinemia) which may lead to incorrect low or high results with this assay. Correct results cannot be obtained by sample dilution and these samples should be analyzed by an alternative method.
*Monoclonal Gammopathy of unknown significance

For diagnostic purposes, the results should always be assessed in conjunction with the patient's medical history, clinical examination and other findings.

ACTION REQUIRED
Special Wash Programming: The use of special wash steps is mandatory when certain test combinations are run together on **cobas c** systems. The latest version of the carry-over evasion list can be found with the NaOHD-SMS-SmpCln1+2-SCCS Method Sheets. For further instructions refer to the operator's manual. **cobas c** 502 analyzer: All special wash programming necessary for avoiding carry-over is available via the **cobas** link, manual input is required in certain cases.

Where required, special wash/carry-over evasion programming must be implemented prior to reporting results with this test.

Limits and ranges

Measuring range

0.50-20.0 mg/L (5.9-236 nmol/L)

Determine samples having higher concentrations via the rerun function. Dilution of samples via the rerun function is a 1:4 dilution. Results from samples diluted using the rerun function are automatically multiplied by a factor of 4.

Lower limits of measurement

Limit of Blank, Limit of Detection and Limit of Quantitation

Limit of Blank	= 0.25 mg/L (2.95 nmol/L)
Limit of Detection	= 0.40 mg/L (4.72 nmol/L)
Limit of Quantitation	= 0.50 mg/L (5.90 nmol/L)

The Limit of Blank and Limit of Detection were determined in accordance with the CLSI (Clinical and Laboratory Standards Institute) EP17-A2 requirements.

The Limit of Blank is the 95th percentile value from n ≥ 60 measurements of analyte-free samples over several independent series. The Limit of Blank corresponds to the concentration below which analyte-free samples are found with a probability of 95 %.

The Limit of Detection is determined based on the Limit of Blank and the standard deviation of low concentration samples.

The Limit of Detection corresponds to the lowest analyte concentration which can be detected (value above the Limit of Blank with a probability of 95 %).

The Limit of Quantitation is the lowest analyte concentration that can be reproducibly measured with a total error of 20 %. It has been determined using low concentration sTfR samples.

Expected values

The values shown below were performed on samples from an apparently healthy population, using the Tina-quant Soluble Transferrin Receptor II assay (STFR2). The calculation is based on 165 sera (101 men, 64 women). The age range was between 22 and 83 years. The analysis of the data with the 2.5 % and the 97.5 % percentile gave a soluble transferrin receptor (sTfR) range from 1.71 mg/L (20.2 nmol/L) to 4.13 mg/L (48.7 nmol/L).

Each laboratory should investigate the transferability of the expected values to its own patient population and if necessary determine its own reference ranges.

Specific performance data

Representative performance data on the analyzers are given below. Results obtained in individual laboratories may differ.

Precision

Precision was determined using human samples and controls in accordance with the CLSI (Clinical and Laboratory Standards Institute) EP5-A3 requirements with repeatability (n = 84) and intermediate precision (2 aliquots per run, 2 runs per day, 21 days)

The following results were obtained:

Repeatability	*Mean*	*SD*	*CV*
	mg/L	*mg/L*	*%*
Control Set sTfR II L 1	2.56	0.0389	1.5
Control Set sTfR II L 2	6.91	0.0626	0.9
Human serum 1	1.21	0.0375	3.1
Human serum 2	2.00	0.0438	2.2
Human serum 3	5.27	0.0526	1.0
Human serum 4	9.23	0.108	1.2
Human serum 5	17.7	0.157	0.9

Intermediate precision	*Mean*	*SD*	*CV*
	mg/L	*mg/L*	*%*
Control Set sTfR II L 1	2.56	0.0444	1.7
Control Set sTfR II L 2	6.91	0.0732	1.1
Human serum 1	1.21	0.0388	3.2
Human serum 2	2.00	0.0475	2.4
Human serum 3	5.27	0.0675	1.3
Human serum 4	9.31	0.118	1.3
Human serum 5	17.7	0.192	1.1

Method comparison

sTfR values for human serum and plasma samples obtained on a **cobas c** 501 analyzer (y) were compared with those determined using the Soluble Transferrin Receptor assay (STFR) on a **cobas c** 501 analyzer(x).

Sample size (n) = 87

Passing/Bablok[14]	Linear regression
y = 0.987x + 0.0347 mg/L	y = 0.989x + 0.0264 mg/L
τ = 0.939	r = 0.996

The sample concentrations were between 0.660 and 19.1 mg/L.

References

1 Wick M, Pinggera W, Lehmann P, eds. Iron Metabolism, Diagnosis and Therapy of Anemias. 4th ed. Vienna/New York: Springer Verlag 1999.

2 Ferguson BJ, Skikne BS, Simpson KM, et al. Serum transferrin receptor distinguishes the anemia of chronic diseases from iron deficiency anemia. J Lab Clin Med 1992;19:385-390.

3 Baynes RD, Skikne BS, Cook JD. Circulating transferrin receptors and assessment of iron status. J Nutr Biochem 1994;5:322-330.

4 Kuiper-Kramer EPA, Huisman CMS, van Raan J, et al. Analytical and clinical implications of soluble transferrin receptors. Eur J Clin Chem Clin Biochem 1996;34:645-649.

5 Baynes RD. Assessment of iron status. Clin Biochem 1996;29:209-215.

6 Punnonen K, Irjala K, Rajamäki A. Serum transferrin receptor and its ratio to serum ferritin in the diagnosis of iron deficiency. Blood 1997;89:1052-1057.

7 Suominen P, Punnonen K, Rajamäki A, et al. Serum transferrin receptor and transferrin receptor-ferritin index identify healthy subjects with subclinical iron deficits. Blood 1998;92:2934-2939.

8 Haeckel R, ed. Evaluation methods in laboratory medicine. VCH Verlagsgesellschaft, Weinheim 1993.

9 Cook JD, Skikne BS, Baynes RD. Serum transferrin receptor. Annu Rev Med 1993;44:63-74.

10 Glick MR, Ryder KW, Jackson SA. Graphical Comparisons of Interferences in Clinical Chemistry Instrumentation. Clin Chem 1986;32:470-475.

For peer review only - http://bmjopen.bmj.com/site/about/guidelines.xhtml

0107227841190c501V1.0

STFR2

Tina-quant Soluble Transferrin Receptor II

11 Breuer J. Report on the Symposium “Drug effects in Clinical Chemistry Methods”. Eur J Clin Chem Clin Biochem 1996;34:385-386.

12 Sonntag O, Scholer A. Drug interference in clinical chemistry: recommendation of drugs and their concentrations to be used in drug interference studies. Ann Clin Biochem 2001;38:376-385.

13 Bakker AJ, Mücke M. Gammopathy interference in clinical chemistry assays: mechanisms, detection and prevention. Clin Chem Lab Med 2007;45(9):1240-1243.

14 Bablok W, Passing H, Bender R, et al. A general regression procedure for method transformation. Application of linear regression procedures for method comparison studies in clinical chemistry, Part III. J Clin Chem Clin Biochem 1988 Nov;26(11):783-790.

A point (period/stop) is always used in this Method Sheet as the decimal separator to mark the border between the integral and the fractional parts of a decimal numeral. Separators for thousands are not used.

Symbols

Roche Diagnostics uses the following symbols and signs in addition to those listed in the ISO 15223-1 standard (for USA: see dialog.roche.com for definition of symbols used):

CONTENT	Contents of kit
→	Volume for reconstitution
GTIN	Global Trade Item Number

COBAS, COBAS C and TINA-QUANT are trademarks of Roche.

All other product names and trademarks are the property of their respective owners.

Additions, deletions or changes are indicated by a change bar in the margin.

© 2020, Roche Diagnostics

CE

Roche Diagnostics GmbH, Sandhofer Strasse 116, D-68305 Mannheim
www.roche.com

For peer review only - http://bmjopen.bmj.com/site/about/guidelines.xhtml

ms_03737551190V14.0

Ferritin

Ferritin

cobas®

REF	Σ	SYSTEM
**03737551** 190	100	Elecsys 2010<br>MODULAR ANALYTICS E170<br>**cobas e** 411<br>**cobas e** 601<br>**cobas e** 602

English

Intended use

Immunoassay for the in vitro quantitative determination of ferritin in human serum and plasma.

The **e**lectro**c**hemi**l**uminescence **i**mmuno**a**ssay "ECLIA" is intended for use on Elecsys and **cobas e** immunoassay analyzers.

Summary

Ferritin is a macromolecule with a molecular weight of at least 440 kDa (depending on the iron content) and consists of a protein shell (apoferritin) of 24 subunits and an iron core containing an average of approx. 2500 Fe^{3+} ions (in liver and spleen ferritin).[1]

Ferritin tends to form oligomers, and when it is present in excess in the cells of the storage organs there is a tendency for condensation to semicrystalline hemosiderin to occur in the lysosomes.

At least 20 isoferritins can be distinguished with the aid of isoelectric focusing.[2] This microheterogeneity is due to differences in the contents of the acidic H and weakly basic L subunits. The basic isoferritins are responsible for the long-term iron storage function, and are found mainly in the liver, spleen, and bone marrow.[1,3]

Acidic isoferritins are found mainly in the myocardium, placenta, and tumor tissue. They have a lower iron content and presumably function as intermediaries for the transfer of iron in various syntheses.[4,5,6]

The determination of ferritin is a suitable method for ascertaining the iron metabolism situation. Determination of ferritin at the beginning of therapy provides a representative measure of the body's iron reserves. A storage deficiency in the reticulo-endothelial system (RES) can be detected at a very early stage.[7]

Clinically, a threshold value of 20 µg/L (ng/mL) has proved useful in the detection of prelatent iron deficiency. This value provides a reliable indication of exhaustion of the iron reserves that can be mobilized for hemoglobin synthesis. Latent iron deficiency is defined as a fall below the 12 µg/L (ng/mL) ferritin threshold. These two values necessitate no further laboratory elucidation, even when the blood picture is still morphologically normal. If the depressed ferritin level is accompanied by hypochromic, microcytal anemia, then manifest iron deficiency is present.[1]

When the ferritin level is elevated and the possibility of a distribution disorder can be ruled out, this is a manifestation of iron overloading in the body. 400 µg/L (ng/mL) ferritin is used as the threshold value. Elevated ferritin values are also encountered with the following tumors: acute leukemia, Hodgkin's disease and carcinoma of the lung, colon, liver and prostate. The determination of ferritin has proved to be of value in liver metastasis. Studies indicate that 76 % of all patients with liver metastasis have ferritin values above 400 µg/L (ng/mL). Reasons for the elevated values could be cell necrosis, blocked erythropoiesis or increased synthesis in tumor tissue.

Two monoclonal mouse antibodies - M-4.184 and M-3.170 - are used to form the sandwich complex in the assay.

Test principle

Sandwich principle. Total duration of assay: 18 minutes.

- 1st incubation: 10 µL of sample, a biotinylated monoclonal ferritin-specific antibody, and a monoclonal ferritin-specific antibody labeled with a ruthenium complex[a] form a sandwich complex.
- 2nd incubation: After addition of streptavidin-coated microparticles, the complex becomes bound to the solid phase via interaction of biotin and streptavidin.
- The reaction mixture is aspirated into the measuring cell where the microparticles are magnetically captured onto the surface of the electrode. Unbound substances are then removed with ProCell/ProCell M. Application of a voltage to the electrode then induces chemiluminescent emission which is measured by a photomultiplier.
- Results are determined via a calibration curve which is instrument-specifically generated by 2-point calibration and a master curve provided via the reagent barcode.

a) Tris(2,2'-bipyridyl)ruthenium(II)-complex ($Ru(bpy)_3^{2+}$)

Reagents - working solutions

The reagent rackpack is labeled as FERR.

M Streptavidin-coated microparticles (transparent cap), 1 bottle, 6.5 mL:
Streptavidin-coated microparticles 0.72 mg/mL; preservative.

R1 Anti-Ferritin-Ab~biotin (gray cap), 1 bottle, 10 mL:
Biotinylated monoclonal anti-ferritin antibody (mouse) 3.0 mg/L; phosphate buffer 100 mmol/L, pH 7.2; preservative.

R2 Anti-ferritin-Ab~$Ru(bpy)_3^{2+}$ (black cap), 1 bottle, 10 mL:
Monoclonal anti-ferritin antibody (mouse) labeled with ruthenium complex 6.0 mg/L; phosphate buffer 100 mmol/L, pH 7.2; preservative.

Precautions and warnings

For in vitro diagnostic use.
Exercise the normal precautions required for handling all laboratory reagents.
Disposal of all waste material should be in accordance with local guidelines.
Safety data sheet available for professional user on request.

Avoid foam formation in all reagents and sample types (specimens, calibrators and controls).

Reagent handling

The reagents in the kit have been assembled into a ready-for-use unit that cannot be separated.

All information required for correct operation is read in from the respective reagent barcodes.

Storage and stability

Store at 2-8 °C.

Do not freeze.

Store the Elecsys reagent kit **upright** in order to ensure complete availability of the microparticles during automatic mixing prior to use.

Stability:	
unopened at 2-8 °C	up to the stated expiration date
after opening at 2-8 °C	12 weeks
on the analyzers	6 weeks

Specimen collection and preparation

Only the specimens listed below were tested and found acceptable.

Serum collected using standard sampling tubes.

Li-, Na-heparin, K_3-EDTA and sodium citrate plasma.

When sodium citrate is used, the results must be corrected by + 10 %.

Criterion: Recovery within 90-110 % of serum value or slope 0.9-1.1 + intercept within < ± 2x analytical sensitivity (LDL) + coefficient of correlation > 0.95.

Stable for 7 days at 2-8 °C, 12 months at -20 °C.[8]

For peer review only - http://bmjopen.bmj.com/site/about/guidelines.xhtml

ms_03737551190V14.0

Ferritin

cobas®

Ferritin

The sample types listed were tested with a selection of sample collection tubes that were commercially available at the time of testing, i.e. not all available tubes of all manufacturers were tested. Sample collection systems from various manufacturers may contain differing materials which could affect the test results in some cases. When processing samples in primary tubes (sample collection systems), follow the instructions of the tube manufacturer.

Centrifuge samples containing precipitates before performing the assay.

Do not use heat-inactivated samples.

Do not use samples and controls stabilized with azide.

Ensure the samples, calibrators and controls are at 20-25 °C prior to measurement.

Due to possible evaporation effects, samples, calibrators and controls on the analyzers should be analyzed/measured within 2 hours.

Materials provided

See "Reagents – working solutions" section for reagents.

Materials required (but not provided)

- REF 03737586190, Ferritin CalSet, 4 x 1 mL
- REF 11776452122, PreciControl Tumor Marker, for 2 x 3 mL each of PreciControl Tumor Marker 1 and 2 or
  REF 05618860190, PreciControl Varia, for 2 x 3 mL each of PreciControl Varia 1 and 2
- REF 11732277122, Diluent Universal, 2 x 16 mL sample diluent or
  REF 03183971122, Diluent Universal, 2 x 36 mL sample diluent
- General laboratory equipment
- Elecsys 2010, MODULAR ANALYTICS E170 or **cobas e** analyzer

Accessories for Elecsys 2010 and **cobas e** 411 analyzers:

- REF 11662988122, ProCell, 6 x 380 mL system buffer
- REF 11662970122, CleanCell, 6 x 380 mL measuring cell cleaning solution
- REF 11930346122, Elecsys SysWash, 1 x 500 mL washwater additive
- REF 11933159001, Adapter for SysClean
- REF 11706802001, Elecsys 2010 AssayCup, 60 x 60 reaction vessels
- REF 11706799001, Elecsys 2010 AssayTip, 30 x 120 pipette tips

Accessories for MODULAR ANALYTICS E170, **cobas e** 601 and **cobas e** 602 analyzers:

- REF 04880340190, ProCell M, 2 x 2 L system buffer
- REF 04880293190, CleanCell M, 2 x 2 L measuring cell cleaning solution
- REF 03023141001, PC/CC-Cups, 12 cups to prewarm ProCell M and CleanCell M before use
- REF 03005712190, ProbeWash M, 12 x 70 mL cleaning solution for run finalization and rinsing during reagent change
- REF 12102137001, AssayTip/AssayCup Combimagazine M, 48 magazines x 84 reaction vessels or pipette tips, waste bags
- REF 03023150001, WasteLiner, waste bags
- REF 03027651001, SysClean Adapter M

Accessories for all analyzers:

- REF 11298500316, Elecsys SysClean, 5 x 100 mL system cleaning solution

Assay

For optimum performance of the assay follow the directions given in this document for the analyzer concerned. Refer to the appropriate operator's manual for analyzer-specific assay instructions.

Resuspension of the microparticles takes place automatically prior to use. Read in the test-specific parameters via the reagent barcode. If in exceptional cases the barcode cannot be read, enter the 15-digit sequence of numbers.

Bring the cooled reagents to approx. 20 °C and place on the reagent disk (20 °C) of the analyzer. Avoid foam formation. The system automatically regulates the temperature of the reagents and the opening/closing of the bottles.

Calibration

Traceability: The Ferritin assay (REF 03737551) has been standardized against the Ferritin assay (REF 11820982). The Ferritin assay (REF 11820982) has been standardized against the Enzymun-Test Ferritin method. This in turn has been standardized against the 1st International Standard (IS) NIBSC (National Institute for Biological Standards and Control) "Reagent for Ferritin (human liver)" 80/602.

Recovery studies, including a published study,[9] to assess traceability of the Elecsys Ferritin assay to more recent international standards (2nd IS 80/578 and 3rd IS 94/572) have been conducted, with results showing very good agreement.

Every Elecsys reagent set has a barcoded label containing specific information for calibration of the particular reagent lot. The predefined master curve is adapted to the analyzer using the relevant CalSet.

Calibration frequency: Calibration must be performed once per reagent lot using fresh reagent (i.e. not more than 24 hours since the reagent kit was registered on the analyzer). Renewed calibration is recommended as follows:

- after 1 month (28 days) when using the same reagent lot
- after 7 days (when using the same reagent kit on the analyzer)
- as required: e.g. quality control findings outside the defined limits

Quality control

For quality control, use PreciControl Tumor Marker or PreciControl Varia.

In addition, other suitable control material can be used.

Controls for the various concentration ranges should be run individually at least once every 24 hours when the test is in use, once per reagent kit, and following each calibration.

The control intervals and limits should be adapted to each laboratory's individual requirements. Values obtained should fall within the defined limits. Each laboratory should establish corrective measures to be taken if values fall outside the defined limits.

Follow the applicable government regulations and local guidelines for quality control.

Calculation

The analyzer automatically calculates the analyte concentration of each sample (either in µg/L or ng/mL).

Limitations - interference

The assay is unaffected by icterus (bilirubin < 1112 µmol/L or < 65 mg/dL), hemolysis (Hb < 0.31 mmol/L or < 0.5 g/dL), lipemia (Intralipid < 3300 mg/dL) and biotin (< 205 nmol/L or < 50 ng/mL).

Criterion: Recovery within ± 10 % of initial value.

Samples should not be taken from patients receiving therapy with high biotin doses (i.e. > 5 mg/day) until at least 8 hours following the last biotin administration.

No interference was observed from rheumatoid factors up to a concentration of 2500 IU/mL.

There is no high-dose hook effect at ferritin concentrations up to 100000 µg/L (ng/mL).

In vitro tests were performed on 19 commonly used pharmaceuticals. No interference with the assay was found.

Iron^{2+}- and iron^{3+}-ions at therapeutic concentrations do not interfere with the Elecsys Ferritin assay.

In rare cases, interference due to extremely high titers of antibodies to analyte-specific antibodies, streptavidin or ruthenium can occur. These effects are minimized by suitable test design.

For diagnostic purposes, the results should always be assessed in conjunction with the patient's medical history, clinical examination and other findings.

Limits and ranges

Measuring range

0.500-2000 µg/L (ng/mL) (defined by the lower detection limit and the maximum of the master curve). Values below the lower detection limit are reported as < 0.500 µg/L (ng/mL). Values above the measuring range are reported as > 2000 µg/L (ng/mL) (or up to 100000 µg/L (ng/mL) for 50-fold diluted samples).

For peer review only - http://bmjopen.bmj.com/site/about/guidelines.xhtml

ms_03737551190V14.0

Ferritin

Ferritin

Lower limits of measurement

Lower detection limit of the test

Lower detection limit: 0.50 µg/L (ng/mL)

The lower detection limit represents the lowest measurable analyte level that can be distinguished from zero. It is calculated as the value lying two standard deviations above that of the lowest standard (master calibrator, standard 1 + 2 SD, repeatability study, n = 21).

Dilution

Samples with ferritin concentrations above the measuring range can be diluted with Diluent Universal. The recommended dilution is 1:50 (either automatically by the MODULAR ANALYTICS E170, Elecsys 2010 or **cobas e** analyzers or manually). The concentration of the diluted sample must be > 40 µg/L (ng/mL).

After manual dilution, multiply the result by the dilution factor.

After dilution by the analyzers, the MODULAR ANALYTICS E170, Elecsys 2010 and **cobas e** software automatically takes the dilution into account when calculating the sample concentration.

Expected values

Results of a study with the Enzymun-Test Ferritin method on samples from 224 healthy test subjects (104 women - mainly premenopausal - and 120 men) are given below. The values correspond to the 5th and 95th percentiles.[10]

Men, 20-60 years: 30-400 µg/L (ng/mL)

Women, 17-60 years: 13-150 µg/L (ng/mL)

Each laboratory should investigate the transferability of the expected values to its own patient population and if necessary determine its own reference ranges.

Specific performance data

Representative performance data on the analyzers are given below. Results obtained in individual laboratories may differ.

Precision

Precision was determined using Elecsys reagents, pooled human sera and controls in a protocol (EP5-A2) of the CLSI (Clinical and Laboratory Standards Institute): 2 runs per day in duplication each for 21 days (n = 84). The following results were obtained:

Elecsys 2010 and **cobas e** 411 analyzers					
		**Repeatability**		**Intermediate precision**	
Sample	Mean µg/L (ng/mL)	SD µg/L (ng/mL)	CV %	SD µg/L (ng/mL)	CV %
Human serum 1	1.45	0.101	7.0	0.168	11.6
Human serum 2	11.9	0.411	3.5	0.798	6.7
Human serum 3	19.2	0.780	4.1	1.47	7.7
Human serum 4	376	10.8	2.9	17.2	4.6
Human serum 5	1361	26.5	1.9	84.4	6.2
PreciControl Varia 1	134	1.96	1.5	2.75	2.1
PreciControl Varia 2	858	15.1	1.8	21.7	2.5

MODULAR ANALYTICS E170, **cobas e** 601 and **cobas e** 602 analyzers					
		**Repeatability**		**Intermediate precision**	
Sample	Mean µg/L (ng/mL)	SD µg/L (ng/mL)	CV %	SD µg/L (ng/mL)	CV %
Human serum 1	1.12	0.139	12.4	0.263	23.4
Human serum 2	12.3	0.467	3.8	0.789	6.4
Human serum 3	20.5	0.837	4.1	1.67	8.1
Human serum 4	392	8.14	2.1	16.9	4.3
Human serum 5	1449	35.6	2.5	92.8	6.4
PreciControl Varia 1	140	2.31	1.7	3.53	2.5
PreciControl Varia 2	900	14.4	1.6	25.0	2.8

Method comparison

A comparison of the Ferritin assay, REF 03737551 (y) with the Ferritin assay, REF 11820982 (x) using clinical samples gave the following correlations:

Number of samples measured: 134

Passing/Bablok[11]	Linear regression
y = 1.00x + 0.72	y = 0.99x + 4.11
τ = 0.984	r = 0.999

The sample concentrations were between approximately 2.68 and 1891 µg/L (ng/mL).

Analytical specificity

Human liver ferritin: 100 % recovery

Human spleen ferritin: 85 % recovery

Human heart ferritin: 1 % recovery

References

1 Wick M, Pinggera W, Lehmann P. Ferritin in Iron Metabolism - Diagnosis of Anemieas (second edition). Springer-Verlag 1995;ISBN 3-211-82525-8 and ISBN 0-387-82525-8.

2 Arosio P, Levi S, Gabri E, et al. Heterogeneity of ferritin II: Immunological aspects. In: Albertini A, Arosio P, Chiancone E, Drysdale J (eds). Ferritins and isoferritins as biochemical markers. Elsevier, Amsterdam 1984;33-47.

3 Kaltwasser JP, Werner E. Serumferritin: Methodische und Klinische Aspekte. Springer Verlag (1980).

4 Morikawa K, Oseko F, Morikawa S. A role for ferritin in hematopoiesis and the immune system. Leuk-Lymphoma 1995;18(5-6):429-433.

5 Borch-Iohnson B. Determination of Iron status: brief review of physiological effects on iron measures. Analyst 1995;120(3):891-903.

6 Cook JD, Skikne BS, Baynes RD. Iron deficiency: the global perspective. Adv-Exp-Med-Biol 1994;356:219-228.

7 Albertini A, Arosio P, Chiancone E, et al. (eds). Functional aspects of isoferritins. In: Jacobs A, Hodgetts J, Hoy TG. Ferritins and isoferritins as biochemical markers. Elsevier, Amsterdam 1984;113-127.

8 Guder WG, Narayanan S, Wisser H, et al. List of Analytes; Preanalytical Variables. Brochure in: Samples: From the Patient to the Laboratory. GIT-Verlag, Darmstadt 1996:14. ISBN 3-928865-22-6.

9 Blackmore S, Hamilton M, Lee A, et al. Automated immunoassay methods for ferritin: recovery studies to assess traceability to an international standard. Clin Chem Lab Med 2008;46(10):1450-1457.

10 Lotz J, Hafner G, Prellwitz W. Reference Study for Ferritin Assays. Kurzmitteilung Clin Lab 1997;43(11):993-994.

11 Bablok W, Passing H, Bender R, et al. A general regression procedure for method transformation. Application of linear regression procedures for method comparison studies in clinical chemistry, Part III. J Clin Chem Clin Biochem 1988 Nov;26(11):783-790.

For further information, please refer to the appropriate operator's manual for the analyzer concerned, the respective application sheets, the product information and the Method Sheets of all necessary components (if available in your country).

A point (period/stop) is always used in this Method Sheet as the decimal separator to mark the border between the integral and the fractional parts of a decimal numeral. Separators for thousands are not used.

For peer review only - http://bmjopen.bmj.com/site/about/guidelines.xhtml

ms_03737551190V14.0

Ferritin

cobas®

Ferritin

Symbols

Roche Diagnostics uses the following symbols and signs in addition to those listed in the ISO 15223-1 standard.

CONTENT	Contents of kit
SYSTEM	Analyzers/Instruments on which reagents can be used
REAGENT	Reagent
CALIBRATOR	Calibrator
→	Volume after reconstitution or mixing

COBAS, COBAS E, ELECSYS, MODULAR and PRECICONTROL are trademarks of Roche. INTRALIPID is a trademark of Fresenius Kabi AB.

All other product names and trademarks are the property of their respective owners.

Significant additions or changes are indicated by a change bar in the margin.

© 2013, Roche Diagnostics

CE

Roche Diagnostics GmbH, Sandhofer Strasse 116, D-68305 Mannheim
www.roche.com

Roche

For peer review only

For peer review only - http://bmjopen.bmj.com/site/about/guidelines.xhtml

05944295500V8.0

Elecsys Folate RBC

cobas®

REF	(book icon)	Σ	SYSTEM
05944295190	05944295500	100	**cobas e** 411<br>**cobas e** 601<br>**cobas e** 602

English

System information

For **cobas e** 411 analyzer: test number 1210
For **cobas e** 601 and **cobas e** 602 analyzers: Application Code Number 272

Intended use

This assay is used for the in vitro quantitative determination of folate in erythrocytes (red blood cells, RBC). The electrochemiluminescence binding assay is intended for use on the Elecsys and **cobas e** immunoassay analyzers.

Summary

Folate belongs to the family of B-group vitamins composed of an aromatic pteridine ring linked through a methylene group to p-aminobenzoic acid and a glutamate residue. Folate (folic acid) is vital for normal cellular functions and plays an essential role in nucleic acid synthesis, methionine regeneration, shuttling and redox reactions of one-carbon units required for normal metabolism and regulation.[1,2]

The folate metabolism can be exemplified as a cycle, where folate facilitates the transfer of one-carbon units from one molecule to another required in various biochemical reactions: for example, tetrahydrofolate (THF) accepts a single carbon unit from serine, which is reduced in a number of steps to 5-methyltetrahydrofolate (5-MTHF). 5-MTHF gives its methyl group to homocysteine, which is - with involvement of methionine synthase and vitamin B12 - enzymatically converted to methionine. The resulting THF starts again the cycle of methyl group synthesis. From methionine, the methyl groups are transferred to S-adenosylmethionine (SAM).[3] SAM serves as a methyl group donor in several methylation reactions, like DNA, RNA and protein methylation.[1]

The methionine cycle is highly sensitive to folate deficiency: with a low folate status, the ability of the cell to re-methylate homocysteine is impaired and this results in increased homocysteine concentrations in plasma.[2]

Folate also plays an essential role in the synthesis of purine and pyrimidine precursors of nucleic acids. Altered distribution of methyl groups and impaired DNA synthesis play an essential role in the development of cancers. Abnormal folate status has also been linked with the development of diseases like cardiovascular diseases, neural tube defects, cleft lip and palate, late pregnancy complications, neurodegenerative and psychiatric disorders.[1,2]

Folate belongs to the group of essential vitamins, i.e. it cannot be synthesized by the human organism and therefore must be absorbed from diet. Primary sources of folates are green and leafy vegetables, sprouts, fruits, brewer's yeast and liver.[1,2]

Folate deficiency can be caused by decreased nutritional intake, poor absorption of ingested folate in the intestine or increased demand of folate, for example during physical activity or pregnancy. Deficiency of folate can also be a result of liver diseases or impaired folate metabolism due to genetic defects or drug interactions.[2]

A clinical manifestation of both folate and vitamin B12 deficiency is the so called megaloblastic (macrocytic) anemia: due to the affected DNA synthesis and cell maturation, especially involving the cells of erythropoiesis, the total count of erythrocytes is significantly reduced. The hemoglobin synthesis capacity however is normal, which leads to abnormally large erythrocyte precursors ("macrocytes" or "megaloblasts"), which have an elevated hemoglobin content ("hyperchromic anemia").[3,4]

Serum folate concentrations may be affected by recent folate intakes, whereas red blood cell (RBC) folate is a measure of the folate intake across the 90-120 days lifespan of erythrocytes. Thus, folate concentrations in RBC give a more accurate picture of a patient's underlying folate status than serum folate concentrations, and are considered by experts as the better measure for folate status.[5]

Because vitamin B12 and folate are closely interrelated in the cellular one-carbon unit metabolism, and also hematologic and clinical consequences of the two vitamin deficiency states might be similar, it is advisable to determine both parameters simultaneously in patients with the relevant symptoms of vitamin deficiency.[3,4]

Test principle

Competition principle. Total duration of assay: 27 minutes.

Whole blood treated with anticoagulants (heparin or EDTA) is mixed with ascorbic acid solution and incubated for approximately 90 minutes at 20-25 °C. Lysis of the erythrocytes takes place, with liberation and stabilization of the intracellular folate. The resulting hemolysate sample is then used for subsequent measurement.

- 1st incubation: By incubating 25 µL of hemolysate sample with the folate pretreatment reagents 1 and 2, bound folate is released from endogenous folate binding proteins.
- 2nd incubation: By incubating the pretreated sample with the ruthenium labeled folate binding protein, a folate complex is formed, the amount of which is dependent upon the analyte concentration in the sample.
- 3rd incubation: After addition of streptavidin-coated microparticles and folate labeled with biotin, the unbound sites of the ruthenium labeled folate binding protein become occupied, with formation of a ruthenium labeled folate binding protein-folate biotin complex. The entire complex becomes bound to the solid phase via interaction of biotin and streptavidin.
- The reaction mixture is aspirated into the measuring cell where the microparticles are magnetically captured onto the surface of the electrode. Unbound substances are then removed with ProCell/ProCell M. Application of a voltage to the electrode then induces chemiluminescent emission which is measured by a photomultiplier.
- Results are determined via a calibration curve which is instrument-specifically generated by 2-point calibration and a master curve provided via the reagent barcode or e-barcode.

Reagents - working solutions

The reagent rackpack (M, R1, R2) is labeled as RBC-FOL.

PT1 Pretreatment reagent 1 (white cap), 1 bottle, 4 mL:
Sodium 2-mercaptoethanesulfonate (MESNA) 40 g/L, pH 5.5.

PT2 Pretreatment reagent 2 (gray cap), 1 bottle, 5 mL:
Sodium hydroxide 25 g/L.

M Streptavidin-coated microparticles (transparent cap), 1 bottle, 6.5 mL:
Streptavidin-coated microparticles 0.72 mg/mL; preservative.

R1 Folate binding protein~Ru(bpy)$_3^{2+}$ (gray cap), 1 bottle, 9 mL:
Ruthenium labeled folate binding protein 75 µg/L; human serum albumin (stabilizer); borate/phosphate/citrate buffer 70 mmol/L, pH 5.5; preservative.

R2 Folate~biotin (black cap), 1 bottle, 8 mL:
Biotinylated folate 17 µg/L; biotin 120 µg/L; human serum albumin (stabilizer); borate buffer 100 mmol/L, pH 9.0; preservative.

Precautions and warnings

For in vitro diagnostic use.
Exercise the normal precautions required for handling all laboratory reagents.
Disposal of all waste material should be in accordance with local guidelines.
Safety data sheet available for professional user on request.

This kit contains components classified as follows in accordance with the Regulation (EC) No. 1272/2008:

2-methyl-2H-isothiazol-3-one hydrochloride

EUH 208 May produce an allergic reaction.

For peer review only - http://bmjopen.bmj.com/site/about/guidelines.xhtml

05944295500V8.0

Elecsys Folate RBC

cobas®

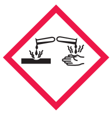

Danger

H290	May be corrosive to metals.
H314	Causes severe skin burns and eye damage.

Prevention:

P280	Wear protective gloves/ protective clothing/ eye protection/ face protection.

Response:

P301 + P330 + P331	IF SWALLOWED: Rinse mouth. Do NOT induce vomiting.
P303 + P361 + P353	IF ON SKIN (or hair): Take off immediately all contaminated clothing. Rinse skin with water.
P304 + P340 + P310	IF INHALED: Remove person to fresh air and keep comfortable for breathing. Immediately call a POISON CENTER/ doctor.
P305 + P351 + P338 + P310	IF IN EYES: Rinse cautiously with water for several minutes. Remove contact lenses, if present and easy to do. Continue rinsing. Immediately call a POISON CENTER/ doctor.
P390	Absorb spillage to prevent material damage.

Product safety labeling follows EU GHS guidance.

Contact phone: all countries: +49-621-7590

All human material should be considered potentially infectious. All products derived from human blood are prepared exclusively from the blood of donors tested individually and shown to be free from HBsAg and antibodies to HCV and HIV. The testing methods used assays approved by the FDA or cleared in compliance with the European Directive 98/79/EC, Annex II, List A.

However, as no testing method can rule out the potential risk of infection with absolute certainty, the material should be handled with the same level of care as a patient specimen. In the event of exposure, the directives of the responsible health authorities should be followed.[6,7]

Avoid foam formation in all reagents and sample types (specimens, calibrators and controls).

Reagent handling

The reagents in the kit have been assembled into a ready-for-use unit that cannot be separated.

All information required for correct operation is read in from the respective reagent barcodes.

Storage and stability

Store at 2-8 °C.

Do not freeze.

Store the Elecsys reagent kit **upright** in order to ensure complete availability of the microparticles during automatic mixing prior to use.

Stability:	
unopened at 2-8 °C	up to the stated expiration date
after opening at 2-8 °C	8 weeks
on the analyzers	2 weeks

Specimen collection and preparation

Only the specimens listed below were tested and found acceptable.

Hemolysate prepared from whole blood treated with anticoagulants (Na-heparin or K_3-EDTA).

- *For the determination of folate in RBC:* Determine hematocrit in whole blood samples and record the value.
- *Preparation of the hemolysate sample*

  Mix 3.0 mL of Folate RBC Hemolyzing Reagent (ascorbic acid solution, 0.2 %) and 100 µL of well-mixed whole blood, avoiding foam formation. Incubate with closed caps for 90 ± 15 minutes at 20-25 °C.

Stability:

Whole blood: 2 hours at 20-25 °C[8], 24 hours at 2-8 °C, 1 month at -20 °C (± 5 °C) (only EDTA-blood).

Hemolysate sample: 1 month at -20 °C (± 5 °C), freeze only once.

The sample types listed were tested with a selection of sample collection tubes that were commercially available at the time of testing, i.e. not all available tubes of all manufacturers were tested. Sample collection systems from various manufacturers may contain differing materials which could affect the test results in some cases. When processing samples in primary tubes (sample collection systems), follow the instructions of the tube manufacturer.

Samples should not subsequently be altered with additives (biocides, anti-oxidants or substances possibly changing the pH of the sample) in order to avoid erroneous folate recovery.

Ensure the samples, calibrators and controls are at 20-25 °C prior to measurement.

Due to possible evaporation effects, samples, calibrators and controls on the analyzers should be analyzed/measured within 2 hours.

If measurements cannot be carried out within 2 hours please store the hemolysate sample at -20 °C (± 5 °C).

Materials provided

See "Reagents – working solutions" section for reagents.

Materials required (but not provided)

- REF 05944309190, Folate RBC CalSet, for 4 x 1.0 mL
- REF 05944317190, Folate RBC Hemolyzing Reagent kit for 4 x 200 mL, contains ascorbic acid
- General laboratory equipment
- **cobas e** analyzer

Additional materials for the **cobas e** 411 analyzer:

- REF 11662988122, ProCell, 6 x 380 mL system buffer
- REF 11662970122, CleanCell, 6 x 380 mL measuring cell cleaning solution
- REF 11930346122, Elecsys SysWash, 1 x 500 mL washwater additive
- REF 11933159001, Adapter for SysClean
- REF 11706802001, AssayCup, 60 x 60 reaction cups
- REF 11706799001, AssayTip, 30 x 120 pipette tips
- REF 11800507001, Clean-Liner

Additional materials for **cobas e** 601 and **cobas e** 602 analyzers:

- REF 04880340190, ProCell M, 2 x 2 L system buffer
- REF 04880293190, CleanCell M, 2 x 2 L measuring cell cleaning solution
- REF 03023141001, PC/CC-Cups, 12 cups to prewarm ProCell M and CleanCell M before use
- REF 03005712190, ProbeWash M, 12 x 70 mL cleaning solution for run finalization and rinsing during reagent change
- REF 03004899190, PreClean M, 5 x 600 mL detection cleaning solution
- REF 12102137001, AssayTip/AssayCup, 48 magazines x 84 reaction cups or pipette tips, waste bags
- REF 03023150001, WasteLiner, waste bags
- REF 03027651001, SysClean Adapter M

Additional materials for all analyzers:

- REF 11298500316, ISE Cleaning Solution/Elecsys SysClean, 5 x 100 mL system cleaning solution

For peer review only - http://bmjopen.bmj.com/site/about/guidelines.xhtml

05944295500V8.0

Elecsys Folate RBC

cobas®

Assay

The well-mixed hemolysate sample is placed in the sample zone of the analyzer and recorded by entering the sample identification data. Complete determinations on the analyzer within 2 hours after finalizing the preparation of the hemolysate sample.

For optimum performance of the assay follow the directions given in this document for the analyzer concerned. Refer to the appropriate operator's manual for analyzer-specific assay instructions.

Resuspension of the microparticles takes place automatically prior to use. Read in the test-specific parameters via the reagent barcode. If in exceptional cases the barcode cannot be read, enter the 15-digit sequence of numbers.

cobas e 601 and **cobas e** 602 analyzers: PreClean M solution is necessary.

Bring the cooled reagents to approximately 20 °C and place on the reagent disk (20 °C) of the analyzer. Avoid foam formation. The system automatically regulates the temperature of the reagents and the opening/closing of the bottles.

Calibration

Traceability: This method has been standardized against the Elecsys Folate III assay (REF 04476433190)/RBC application.

The standardization of the Elecsys Folate RBC assay includes the volume correction to account for the preparation of hemolysate sample (1:31 vol/vol).

Every Elecsys reagent set has a barcoded label containing specific information for calibration of the particular reagent lot. The predefined master curve is adapted to the analyzer using the relevant CalSet.

Calibration frequency: Calibration must be performed once per reagent lot using fresh reagent (i.e. not more than 24 hours since the reagent kit was registered on the analyzer).

Calibration interval may be extended based on acceptable verification of calibration by the laboratory.

Renewed calibration is recommended as follows:

- after 1 month (28 days) when using the same reagent lot
- after 7 days (when using the same reagent kit on the analyzer)
- as required: e.g. quality control findings outside the defined limits

Quality control

For quality control, use commercially available whole blood control material.

Controls for the various concentration ranges should be run individually at least once every 24 hours when the test is in use, once per reagent kit, and following each calibration.

The control intervals and limits should be adapted to each laboratory's individual requirements. Values obtained should fall within the defined limits. Each laboratory should establish corrective measures to be taken if values fall outside the defined limits.

If necessary, repeat the measurement of the samples concerned.

Follow the applicable government regulations and local guidelines for quality control.

Calculation

1. Whole blood folate (from hemolysate sample)

The standardization of the Elecsys Folate RBC assay includes the volume correction to account for the preparation of hemolysate sample (1:31 vol/vol).

The analyzer automatically calculates the analyte concentration of each sample in nmol/L or ng/mL.

Conversion factors:	nmol/L x 0.44 = ng/mL
	ng/mL x 2.27 = nmol/L

2. RBC folate

To calculate the folate concentration in the erythrocyte fraction of the sample **(RBC folate)**, the predetermined sample specific hematocrit value must be taken into account using the following equation:

$$\text{RBC folate} = \frac{\text{analyzer result}}{\%\ \text{hematocrit}} \times 100$$

Limitations - interference

The assay is unaffected by icterus (bilirubin < 564 µmol/L or < 33 mg/dL), lipemia (Intralipid < 1500 mg/dL), biotin (< 86.1 nmol/L or < 21 ng/mL), IgG < 16 g/L and IgA < 4.0 g/L.

Criterion: Recovery within ± 10 % of initial value with samples > 155 ng/mL and ≤ ± 15.5 ng/mL with samples ≤ 155 ng/mL.

Samples should not be taken from patients receiving therapy with high biotin doses (i.e. > 5 mg/day) until at least 8 hours following the last biotin administration.

No interference was observed from rheumatoid factors up to a concentration of 1000 IU/mL.

In vitro tests were performed on 16 commonly used pharmaceuticals and in addition on human erythropoietin. No interference with the assay was found.

It is contraindicated to measure samples of patients receiving therapy with certain pharmaceuticals, e.g. methotrexate or leucovorin, because of the cross-reactivity of folate binding protein with these compounds.

In rare cases, interference due to extremely high titers of antibodies to analyte-specific antibodies, streptavidin or ruthenium can occur. These effects are minimized by suitable test design.

For diagnostic purposes, the results should always be assessed in conjunction with the patient's medical history, clinical examination and other findings.

In rare cases, samples with low erythrocyte folate concentration, but high serum folate concentration can occur. In these cases, a correction of the folate concentration in erythrocytes by the serum folate concentration with the following equation is recommended*:

* expected values can be used as an indicator for high serum folate concentration

Corrected RBC folate concentration =

$$\text{RBC folate concentration} - \left(\text{serum folate concentration} \times \frac{100 - \%\ \text{hematocrit}}{\%\ \text{hematocrit}}\right)$$

Example

RBC folate concentration: 241 (ng/mL RBC);

serum folate concentration: 10.5 (ng/mL S);

hematocrit measured (%) = 45

Corrected RBC folate concentration =

$$241\ \text{ng/mL RBC} - \left(10.5\ \text{ng/mL S} \times \frac{100 - 45}{45}\right) = 228\ \text{ng/mL RBC}$$

Limits and ranges

Measuring range

120-620 ng/mL or 272-1407 nmol/L (defined by the Limit of Quantitation and the maximum of the master curve). Values below the Limit of Quantitation are reported as < 120 ng/mL (< 272 nmol/L). Values above the measuring range are reported as > 620 ng/mL (> 1407 nmol/L). Values are not corrected for the sample hematocrit.

Lower limits of measurement

Limit of Blank, Limit of Detection and Limit of Quantitation:

Limit of Blank = 20.0 ng/mL

Limit of Detection = 46.5 ng/mL

Limit of Quantitation = 120 ng/mL

The Limit of Blank, Limit of Detection and Limit of Quantitation were determined in accordance with the CLSI (Clinical and Laboratory Standards Institute) EP17-A requirements.

The Limit of Blank is the 95th percentile value from n ≥ 60 measurements of analyte-free samples over several independent series. The Limit of Blank corresponds to the concentration below which analyte-free samples are found with a probability of 95 %.

The Limit of Detection is determined based on the Limit of Blank and the standard deviation of low concentration samples. The Limit of Detection corresponds to the lowest analyte concentration which can be detected (value above the Limit of Blank with a probability of 95 %).

The Limit of Quantitation is defined as the lowest amount of analyte in a sample that can be accurately quantitated with a total allowable relative

For peer review only - http://bmjopen.bmj.com/site/about/guidelines.xhtml

05944295500V8.0

Elecsys Folate RBC

cobas®

error of ≤ 30 %. It has been determined using low concentration folate samples.

Dilution

Hemolysate samples with folate concentrations above the measuring range can be diluted manually with Elecsys Folate RBC Hemolyzing Reagent (ascorbic acid solution, 0.2 %). The recommended dilution is 1:2. The concentration of the diluted sample must be > 265 ng/mL or > 602 nmol/L. After manual dilution, multiply the results by the dilution factor 2.

Expected values

The values shown below were measured on samples from an apparently healthy population, using the Elecsys Folate III/RBC application. The values can be applied for the Elecsys Folate RBC assay on all Elecsys and **cobas e** analyzers. The calculation is based on 290 sera (96 men, 194 women) from an European population. The age range was between 18 and 65 years. Pregnant or lactating women were excluded. The reference population was selected according to normal homocysteine values. The following values were obtained:

Whole blood folate (from hemolysate samples)					
	N	Median		2.5^{th}-97.5^{th} percentile	
		nmol/L	ng/mL	nmol/L	ng/mL
Europe	290	673	296	481-1212	212-534

The measured hematocrit value in this study showed a range from 37.1-46.1 %.

RBC folate (folate in erythrocyte fraction)					
	N	Median		2.5^{th}-97.5^{th} percentile	
		nmol/L	ng/mL	nmol/L	ng/mL
Europe	290	1657	730	1187-2854	523-1257

If pathologically low hematocrit values are considered for calculation of RBC folate in the erythrocyte fraction, elevated RBC folate concentrations may be observed. No medical conclusion should be based on the calculation considering hematocrit values in such cases. Instead, whole blood folate results (from hemolysate samples) and suitable expected values may be used.

Each laboratory should investigate the transferability of the expected values to its own patient population and if necessary determine its own reference ranges.

Specific performance data

Representative performance data on the analyzers are given below. Results obtained in individual laboratories may differ.

Precision

Precision was determined using Elecsys reagents and hemolysate samples in a protocol (EP5-A2) of the CLSI (Clinical and Laboratory Standards Institute): 2 runs per day in duplicate each for 21 days (n = 84). Results are given as whole blood folate (from hemolysate sample). The following results were obtained:

**cobas e** 411 analyzer								
			**Repeatability**			**Intermediate precision**		
Sample	Mean		SD		CV	SD		CV
	nmol/L	ng/mL	nmol/L	ng/mL	%	nmol/L	ng/mL	%
HL[a] 1	154	68.0	11.7	5.17	7.6	21.9	9.65	14.2
HL 2	352	155	17.5	7.73	5.0	27.7	12.2	7.9
HL 3	618	272	25.4	11.2	4.1	38.4	16.9	6.2
HL 4	1195	527	38.8	17.1	3.3	56.3	24.8	4.7

a) HL = Hemolysate

**cobas e** 601 and **cobas e** 602 analyzers								
			**Repeatability**			**Intermediate precision**		
Sample	Mean		SD		CV	SD		CV
	nmol/L	ng/mL	nmol/L	ng/mL	%	nmol/L	ng/mL	%
HL 1	138	61.0	12.1	5.31	8.8	14.3	6.32	10.4
HL 2	434	191	26.1	11.5	6.0	28.4	12.5	6.5
HL 3	586	258	32.0	14.1	5.5	34.3	15.1	5.9
HL 4	1317	580	29.1	12.8	2.2	44.7	19.7	3.4

Method comparison

a) A comparison of the Elecsys Folate RBC assay (calibrated with Folate RBC CalSet; y) and the Elecsys Folate III/RBC application (calibrated with Folate III CalSet; x) using hemolyzed clinical samples gave the following correlations (ng/mL). Results are given as whole blood folate (from hemolysate sample).

Number of samples measured: 187

Passing/Bablok[9]	Linear regression
y = 1.02x - 14.1	y = 1.00x - 12.0
τ = 0.869	r = 0.985

The sample concentrations were between 151 and 551 ng/mL (343 and 1251 nmol/L).

b) A comparison of the Elecsys Folate RBC assay on the MODULAR ANALYTICS E170 analyzer (y) with the Elecsys Folate RBC assay on the Elecsys 2010 analyzer (x) (both tests have been calibrated with Folate RBC CalSet) using hemolyzed clinical samples gave the following correlations (ng/mL). Results are given as whole blood folate (from hemolysate sample).

Number of samples measured: 187

Passing/Bablok	Linear regression
y = 1.04x + 1.94	y = 1.02x + 8.07
τ = 0.814	r = 0.970

The sample concentrations were between 137 and 557 ng/mL (311 and 1264 nmol/L).

References

1 Nazki FH, Sameer AS, Ganaie BA. Folate: Metabolism, genes, polymorphisms and the associated diseases. Gene 2014;533(1):11-20.

2 Scaglione F, Panzavolta G. Folate, folic acid and 5-methyltetrahydrofolate are not the same thing. Xenobiotica 2014;44(5):480-488.

3 Reynolds EH. The neurology of folic acid deficiency. Handb Clin Neurol 2014;120:927-43.

4 Wick M, Pinggera W, Lehmann P. Clinical Aspects and Laboratory. Iron metabolism, Anemias. Springer Verlag, Wien, New York, 6th edition 2011:41-42.

5 Yetley EA, Pfeiffer CM, Phinney KW, et al. Biomarkers of folate status in NHANES: a roundtable summary. Am J Clin Nutr 2011;94(1):303S-312S.

6 Occupational Safety and Health Standards: Bloodborne pathogens. (29 CFR Part 1910.1030). Fed. Register.

7 Directive 2000/54/EC of the European Parliament and Council of 18 September 2000 on the protection of workers from risks related to exposure to biological agents at work.

8 Eijsden M, van der Wal MF, Hornstra G, et al. Can whole blood samples be stored over 24 hours without compromising stability of C-Reactive Protein, Retinol, Ferritin, Folic Acid and Fatty Acids in Epidemiology Research? Clin Chem 2005;51(1):230-232.

9 Bablok W, Passing H, Bender R, et al. A general regression procedure for method transformation. Application of linear regression procedures for method comparison studies in clinical chemistry, Part III. J Clin Chem Clin Biochem 1988 Nov;26(11):783-790.

For peer review only - http://bmjopen.bmj.com/site/about/guidelines.xhtml

05944295500V8.0

Elecsys Folate RBC

cobas®

For further information, please refer to the appropriate operator's manual for the analyzer concerned, the respective application sheets, the product information and the Method Sheets of all necessary components (if available in your country).

A point (period/stop) is always used in this Method Sheet as the decimal separator to mark the border between the integral and the fractional parts of a decimal numeral. Separators for thousands are not used.

Symbols

Roche Diagnostics uses the following symbols and signs in addition to those listed in the ISO 15223-1 standard (for USA: see dialog.roche.com for definition of symbols used):

CONTENT	Contents of kit
SYSTEM	Analyzers/Instruments on which reagents can be used
REAGENT	Reagent
CALIBRATOR	Calibrator
→	Volume after reconstitution or mixing
GTIN	Global Trade Item Number

COBAS, COBAS E, ELECSYS and PRECICONTROL are trademarks of Roche. INTRALIPID is a trademark of Fresenius Kabi AB.

All other product names and trademarks are the property of their respective owners.

Additions, deletions or changes are indicated by a change bar in the margin.

© 2020, Roche Diagnostics

CE

Roche Diagnostics GmbH, Sandhofer Strasse 116, D-68305 Mannheim
www.roche.com

Roche

For peer review only

For peer review only - http://bmjopen.bmj.com/site/about/guidelines.xhtml

0004628918190c501V12.0

CRPHS

cobas®

Cardiac C-Reactive Protein (Latex) High Sensitive

Order information

REF	CONTENT		Analyzer(s) on which **cobas c** pack(s) can be used
**04628918** 190	Cardiac C-Reactive Protein (Latex) High Sensitive (300 tests)	System-ID 07 6866 9	Roche/Hitachi **cobas c** 311, **cobas c** 501/502
**11355279** 216	Calibrator f.a.s. Proteins (5 x 1 mL)	Code 656	
**11355279** 160	Calibrator f.a.s. Proteins (5 x 1 mL, for USA)	Code 656	
**20766321** 322	CRP T Control N (5 x 0.5 mL)	Code 235	
**10557897** 122	Precinorm Protein (3 x 1 mL)	Code 302	
**10557897** 160	Precinorm Protein (3 x 1 mL, for USA)	Code 302	
**05117003** 190	PreciControl ClinChem Multi 1 (20 x 5 mL)	Code 391	
**05947626** 190	PreciControl ClinChem Multi 1 (4 x 5 mL)	Code 391	
**05947626** 160	PreciControl ClinChem Multi 1 (4 x 5 mL, for USA)	Code 391	
**04489357** 190	Diluent NaCl 9 % (50 mL)	System-ID 07 6869 3	

English

System information

For **cobas c** 311/501 analyzers:

CRPHS: ACN 217

For **cobas c** 502 analyzer:

CRPHS: ACN 8217

Intended use

In vitro test for the quantitative determination of C-reactive protein (CRP) in human serum and plasma on Roche/Hitachi **cobas c** systems.
Measurement of CRP is of use for the detection and evaluation of inflammatory disorders and associated diseases, infection and tissue injury. Highly sensitive measurement of CRP may also be used as an aid in the assessment of the risk of future coronary heart disease. When used as an adjunct to other laboratory evaluation methods of acute coronary syndromes, it may also be an additional independent indicator of recurrent event prognosis in patients with stable coronary disease or acute coronary syndrome.

Summary[1,2,3,4,5,6,7,8,9,10,11,12,13,14,15,16,17,18,19,20,21]

C-reactive protein is the classic acute phase protein in inflammatory reactions. It is synthesized by the liver and consists of five identical polypeptide chains that form a five-member ring having a molecular weight of 105000 daltons. CRP is the most sensitive of the acute phase reactants and its concentration increases rapidly during inflammatory processes. Complexed CRP activates the complement system beginning with C1q. CRP then initiates opsonization and phagocytosis of invading cells, but its main function is to bind and detoxify endogenous toxic substances produced as a result of tissue damage.

CRP assays are used to detect systemic inflammatory processes (apart from certain types of inflammation such as SLE and Colitis ulcerosa); to assess treatment of bacterial infections with antibiotics; to detect intrauterine infections with concomitant premature amniorrhexis; to differentiate between active and inactive forms of disease with concurrent infection, e.g. in patients suffering from SLE or Colitis ulcerosa; to therapeutically monitor rheumatic disease and assess anti-inflammatory therapy; to determine the presence of post-operative complications at an early stage, such as infected wounds, thrombosis and pneumonia, and to distinguish between infection and bone marrow transplant rejection.

Sensitive CRP measurements have been used and discussed for early detection of infection in pediatrics and risk assessment of coronary heart disease. Several studies came to the conclusion that the highly sensitive measurement of CRP could be used as a marker to predict the risk of coronary heart disease in apparently healthy persons and as an indicator of recurrent event prognosis. Increases in CRP values are non-specific and should not be interpreted without a complete clinical history. The American Heart Association and the Centers for Disease Control and Prevention have made several recommendations concerning the use of high sensitivity C-Reactive Protein (hsCRP) in cardiovascular risk assessment.[21] Testing for any risk assessment should not be performed while there is an indication of infection, systemic inflammation or trauma. Patients with persistently unexplained hsCRP levels above 10 mg/L (95.2 nmol/L) should be evaluated for non-cardiovascular etiologies. When using hsCRP to assess the risk of coronary heart disease, measurements should be made on metabolically stable patients and compared to previous values. Optimally, the average of hsCRP results repeated two weeks apart should be used for risk assessment. Screening the entire adult population for hsCRP is not recommended, and hsCRP is not a substitute for traditional cardiovascular risk factors. Acute coronary syndrome management should not depend solely on hsCRP measurements. Similarly, application of secondary prevention measures should be based on global risk assessment and not solely on hsCRP measurements. Serial measurements of hsCRP should not be used to monitor treatment.

Various assay methods are available for CRP determination, such as nephelometry and turbidimetry. The Roche CRP assay is based on the principle of particle-enhanced immunological agglutination.

Test principle[22,23]

Particle enhanced immunoturbidimetric assay.

Human CRP agglutinates with latex particles coated with monoclonal anti-CRP antibodies. The precipitate is determined turbidimetrically.

Reagents - working solutions

R1 TRIS buffer with bovine serum albumin and immunoglobulins (mouse); preservative; stabilizers

R2 Latex particles coated with anti-CRP (mouse) in glycine buffer; preservative; stabilizers

R1 is in position B and R2 is in position C.

Precautions and warnings

For in vitro diagnostic use.
Exercise the normal precautions required for handling all laboratory reagents.
Disposal of all waste material should be in accordance with local guidelines. Safety data sheet available for professional user on request.

For USA: Caution: Federal law restricts this device to sale by or on the order of a physician.

Reagent handling

Ready for use

Mix **cobas c** pack well before placing on the analyzer.

Carefully invert reagent container several times prior to use to ensure that the reagent components are mixed.

Storage and stability

CRPHS

Shelf life at 2-8 °C:	See expiration date on **cobas c** pack label.
On-board in use and refrigerated on the analyzer:	12 weeks

Diluent NaCl 9 %

Shelf life at 2-8 °C:	See expiration date on **cobas c** pack label.
On-board in use and refrigerated on the analyzer:	12 weeks

For peer review only - http://bmjopen.bmj.com/site/about/guidelines.xhtml

0004628918190c501V12.0

CRPHS

Cardiac C-Reactive Protein (Latex) High Sensitive

cobas®

Specimen collection and preparation

For specimen collection and preparation only use suitable tubes or collection containers.

Only the specimens listed below were tested and found acceptable.
Serum.
Plasma: Li-heparin and K_2-EDTA plasma

The sample types listed were tested with a selection of sample collection tubes that were commercially available at the time of testing, i.e. not all available tubes of all manufacturers were tested. Sample collection systems from various manufacturers may contain differing materials which could affect the test results in some cases. When processing samples in primary tubes (sample collection systems), follow the instructions of the tube manufacturer.

Centrifuge samples containing precipitates before performing the assay.

See the limitations and interferences section for details about possible sample interferences.

Sample stability claims were established by experimental data by the manufacturer or based on reference literature and only for the temperatures/time frames as stated in the method sheet. It is the responsibility of the individual laboratory to use all available references and/or its own studies to determine specific stability criteria for its laboratory.

Stability:[24]	11 days at 15‑25 °C
	2 months at 2‑8 °C
	3 years at (-15)-(-25) °C

Materials provided

See “Reagents – working solutions” section for reagents.

Materials required (but not provided)

- See “Order information” section
- General laboratory equipment

Assay

For optimum performance of the assay follow the directions given in this document for the analyzer concerned. Refer to the appropriate operator’s manual for analyzer-specific assay instructions.

The performance of applications not validated by Roche is not warranted and must be defined by the user.

Application for serum and plasma

cobas c 311 test definition

Assay type	Rate A	
Reaction time / Assay points	10/7-57	
Wavelength (sub/main)	– /546 nm	
Reaction direction	Increase	
Units	mg/L (nmol/L, mg/dL)	
Reagent pipetting		Diluent (H_2O)
R1	82 µL	42 µL
R2	28 µL	20 µL

Sample volumes	*Sample*	*Sample dilution*	
		Sample	*Diluent (NaCl)*
Normal	6 µL	–	–
Decreased	6 µL	10 µL	140 µL
Increased	6 µL	–	–

cobas c 501 test definition

Assay type	Rate A	
Reaction time / Assay points	10/12-70	
Wavelength (sub/main)	– /546 nm	
Reaction direction	Increase	
Units	mg/L (nmol/L, mg/dL)	
Reagent pipetting		Diluent (H_2O)
R1	82 µL	42 µL
R2	28 µL	20 µL

Sample volumes	*Sample*	*Sample dilution*	
		Sample	*Diluent (NaCl)*
Normal	6 µL	–	–
Decreased	6 µL	10 µL	140 µL
Increased	6 µL	–	–

cobas c 502 test definition

Assay type	Rate A	
Reaction time / Assay points	10/12-70	
Wavelength (sub/main)	– /546 nm	
Reaction direction	Increase	
Units	mg/L (nmol/L, mg/dL)	
Reagent pipetting		Diluent (H_2O)
R1	82 µL	42 µL
R2	28 µL	20 µL

Sample volumes	*Sample*	*Sample dilution*	
		Sample	*Diluent (NaCl)*
Normal	6 µL	–	–
Decreased	6 µL	10 µL	140 µL
Increased	12 µL	–	–

Calibration

Calibrators	S1: H_2O	
	S2: C.f.a.s. Proteins	
	Multiply the lot-specific C.f.a.s. Proteins calibrator value by the factors below to determine the standard concentrations for the 6-point calibration curve:	
	S2: 0.0125	S5: 0.100
	S3: 0.0250	S6: 0.200
	S4: 0.0500	
Calibration mode	Line Graph	
Calibration frequency	Full calibration<br>• after reagent lot change<br>• as required following quality control procedures	

Calibration interval may be extended based on acceptable verification of calibration by the laboratory.

Traceability: This method has been standardized against the reference preparation of the IRMM (Institute for Reference Materials and Measurements) BCR470/CRM470 (RPPHS - Reference Preparation for Proteins in Human Serum).[25]

Quality control

For quality control, use control materials as listed in the "Order information" section.

In addition, other suitable control material can be used.

The control intervals and limits should be adapted to each laboratory’s individual requirements. Values obtained should fall within the defined

For peer review only - http://bmjopen.bmj.com/site/about/guidelines.xhtml

0004628918190c501V12.0

CRPHS

cobas®

Cardiac C-Reactive Protein (Latex) High Sensitive

limits. Each laboratory should establish corrective measures to be taken if values fall outside the defined limits.

Follow the applicable government regulations and local guidelines for quality control.

Calculation

Roche/Hitachi **cobas c** systems automatically calculate the analyte concentration of each sample.

Conversion factors:	mg/L x 9.52 = nmol/L
	mg/L x 0.1 = mg/dL

Limitations - interference

Criterion: Recovery within ± 10 % of initial values at CRP levels of 1.0 mg/L.

Icterus:[26] No significant interference up to an I index of 60 for conjugated bilirubin and unconjugated bilirubin (approximate conjugated and unconjugated bilirubin concentration: 60 mg/dL or 1026 µmol/L).

Hemolysis:[26] No significant interference up to an H index of 1000 (approximate hemoglobin concentration: 622 µmol/L or 1000 mg/dL).

Lipemia (Intralipid):[26] No significant interference up to an L index of 600. There is poor correlation between the L index (corresponds to turbidity) and triglycerides concentration.

Rheumatoid factors: No significant interference from rheumatoid factors up to a concentration of 1200 IU/mL.

Drugs: No interference was found at therapeutic concentrations using common drug panels.[27,28]

Therapeutic drugs: Significantly decreased CRP values may be obtained from samples taken from patients who have been treated with carboxypenicillins.

High dose hook-effect: No false result occurs up to a CRP concentration of 1000 mg/L.

In very rare cases, gammopathy, in particular type IgM (Waldenström's macroglobulinemia), may cause unreliable results.[29]

Although measures were taken to minimize interference caused by human anti-mouse antibodies, erroneous findings may be obtained from samples taken from patients who have been treated with monoclonal mouse antibodies or have received them for diagnostic purposes.

For diagnostic purposes, the results should always be assessed in conjunction with the patient's medical history, clinical examination and other findings.

ACTION REQUIRED
Special Wash Programming: The use of special wash steps is mandatory when certain test combinations are run together on Roche/Hitachi **cobas c** systems. The latest version of the carry-over evasion list can be found with the NaOHD-SMS-SmpCln1+2-SCCS Method Sheets. For further instructions refer to the operator's manual. **cobas c** 502 analyzer: All special wash programming necessary for avoiding carry-over is available via the **cobas** link, manual input is required in certain cases.

Where required, special wash/carry-over evasion programming must be implemented prior to reporting results with this test.

Limits and ranges

Measuring range

0.15-20.0 mg/L (1.43-190 nmol/L, 0.015-2.0 mg/dL)

Determine samples having higher concentrations via the rerun function. Dilution of samples via the rerun function is a 1:15 dilution. Results from samples diluted using the rerun function are automatically multiplied by a factor of 15.

Lower limits of measurement

Lower detection limit of the test

0.15 mg/L (1.43 nmol/L, 0.015 mg/dL)

The lower detection limit represents the lowest measurable analyte level that can be distinguished from zero. It is calculated as the value lying 3 standard deviations above that of the lowest standard (standard 1 + 3 SD, repeatability, n = 21).

Functional sensitivity

0.3 mg/L (2.96 nmol/L, 0.03 mg/dL)

The functional sensitivity is the lowest CRP concentration that can be reproducibly measured with an inter-assay coefficient of variation of < 10 %.

Expected values

Consensus reference interval for adults:[30]

IFCC/CRM 470

mg/dL	mg/L	nmol/L
< 0.5	< 5.0	< 47.6

The CDC/AHA recommended the following hsCRP cut-off points (tertiles) for CVD risk assessment:[21,31]

hsCRP level (mg/L)	hsCRP level (nmol/L)	Relative risk
< 1.0	< 9.52	low
1.0-3.0	9.52-28.6	average
> 3.0	> 28.6	high

Patients with higher hsCRP concentrations are more likely to develop myocardial infarction and severe peripheral vascular disease.

5-95 % reference intervals of neonates and children:[32]

Neonates (0-3 weeks): 0.1-4.1 mg/L (0.95-39.0 nmol/L)

Children (2 months-15 years): 0.1-2.8 mg/L (0.95-26.7 nmol/L)

It is important to monitor the CRP concentration during the acute phase of the illness.

Roche has not evaluated reference ranges in a pediatric population.

Each laboratory should investigate the transferability of the expected values to its own patient population and if necessary determine its own reference ranges.

Increases in CRP values are non-specific and should not be interpreted without a complete clinical history.

When using hsCRP to assess the risk of coronary heart disease, measurements should be made on metabolically stable patients and compared to previous values. Optimally, the average of hsCRP results repeated two weeks apart should be used for risk assessment. Measurements should be compared to previous values. When the results are being used for risk assessment, patients with persistently unexplained hsCRP levels of above 10 mg/L (95.2 nmol/L) should be evaluated for non-cardiovascular origins. Testing for any risk assessment should not be performed while there is indication of infection, systemic inflammation or trauma.[21]

Specific performance data

Representative performance data on the analyzers are given below. Results obtained in individual laboratories may differ.

Precision

Precision was determined using human samples and controls in an internal protocol with repeatability (n = 21) and intermediate precision (3 aliquots per run, 1 run per day, 21 days). The following results were obtained:

Repeatability	*Mean*	*SD*	*CV*
	mg/L (nmol/L, mg/dL)	*mg/L (nmol/L, mg/dL)*	*%*
Precinorm Protein	9.00 (85.7, 0.900)	0.10 (1.0, 0.010)	1.2
CRP T Control N	4.34 (41.3, 0.434)	0.04 (0.4, 0.004)	1.0
Human serum 1	15.9 (151, 1.59)	0.1 (1, 0.01)	0.4
Human serum 2	0.54 (5.14, 0.054)	0.01 (0.10, 0.001)	1.6

Intermediate precision	*Mean*	*SD*	*CV*
	mg/L (nmol/L, mg/dL)	*mg/L (nmol/L, mg/dL)*	*%*
Precinorm Protein	9.06 (86.3, 0.906)	0.11 (1.1, 0.011)	1.3
CRP T Control N	4.28 (40.8, 0.428)	0.11 (1.1, 0.011)	2.6
Human serum 3	13.3 (126, 1.33)	0.3 (3, 0.03)	2.1
Human serum 4	0.53 (5.05, 0.053)	0.05 (0.48, 0.005)	8.4

Method comparison

CRP values for human serum and plasma samples obtained on a Roche/Hitachi **cobas c** 501 analyzer (y) were compared with those

For peer review only - http://bmjopen.bmj.com/site/about/guidelines.xhtml

0004628918190c501V12.0

CRPHS

cobas®

Cardiac C-Reactive Protein (Latex) High Sensitive

determined using the corresponding reagent on a Roche/Hitachi 917 analyzer (x).

Sample size (n) = 192

Passing/Bablok[33]	Linear regression
y = 0.992x + 0.254 mg/L	y = 0.946x + 0.514 mg/L
τ = 0.944	r = 0.996

The sample concentrations were between 0.500 and 19.7 mg/L (4.76 and 188 nmol/L, 0.050 and 1.97 mg/dL).

References

1 Henry JB, ed. Clinical Diagnosis and Management by Laboratory Methods. Vol II. Philadelphia, Pa: WB Saunders 1979.

2 Greiling H, Gressner AM, eds. Lehrbuch der Klinischen Chemie und Pathobiochemie, 3rd ed. Stuttgart/New York: Schattauer Verlag 1995:234-236.

3 Thomas L, Messenger M. Pathobiochemie und Labordiagnostik der Entzündung. Lab med 1993;17:179–194.

4 Young B, Gleeson M, Cripps AW. C-reactive protein: A critical review. Pathology 1991;23:118-124.

5 Tietz NW. Clinical Guide to Laboratory Tests. 3rd ed. Philadelphia, Pa: WB Saunders Co; 1995.

6 Wasunna A, Whitelaw A, Gallimore R, et al. C-reactive protein and bacterial infection in preterm infants. Eur J Pediatr 1990 Mar;149(6):424-427.

7 Liuzzo G, Biasucci LM, Gallimore JR, et al. The prognostic value of C-reactive protein and serum amyloid A protein in severe unstable angina. N Engl J Med 1994;331:417-424.

8 Kuller LH, Tracy RP, Shaten J, et al. Relation of c-reactive protein and coronary heart disease in the MRFIT nested case control study. Am J Epidem 1996;144:537-547.

9 Ridker PM, Glynn RJ, Hennekens CH, et al. C-Reactive Protein Adds to the Predictive Value of Total and HDL Cholesterol in Determining Risk of First Myocardial Infarction. Circulation 1998;97:2007-2011.

10 Ridker PM, Cushman M, Stampfer MJ, et al. Plasma Concentration of C-Reactive Protein and Risk of Developing Peripheral Vascular Disease. Circulation 1998;97:425-428.

11 Ridker PM, Cushman M, Stampfer MJ, et al. Inflammation, Aspirin, and the Risk of Cardiovascular Disease in Apparently Healthy Men. N Eng J Med 1997;336(14):973-979.

12 Danesh J, Wheeler JG, Hirschfield GM, et al. C-Reactive Protein and Other Circulating Markers of Inflammation in the Prediction of Coronary Heart Disease. N Eng J Med 2004;350(14):1387-1397.

13 Ridker PM, Hennekens CH, Buring JE, et al. C-Reactive Protein and Other Markers of Inflammation in the Prediction of Cardiovascular Disease in Women. N Engl J Med 2000;342(12):836-843.

14 Tracy RP, Lemaitre RN, Psaty BM, et al. Relationship of C-Reactive Protein to Risk of Cardiovascular Disease in the Elderly. Arterioscler Thromb Vasc Biol 1997;17:1121-1127.

15 Järvisalo MJ, Harmoinen A, Hakanen M, et al. Elevated Serum C-Reactive Protein Levels and Early Arterial Changes in Healthy Children. Arterioscler Thromb Vasc Biol, (August) 2002;1323-1328.

16 Almagor M, Keren A, Banai S. Increased C-Reactive Protein Level after Coronary Stent Implantation in Patients with Stable Coronary Artery Disease. American Heart Journal 2003;145 (2):248-253.

17 Katritsis D, Korovesis S, Giazitzoglou E, et al. C-Reactive Protein Concentrations and Angiographic Characteristics of Coronary Lesions. Clin Chem 2001;47(5):882-886.

18 Beattie MS, Shlipak, MG, Liu H, et al. C-Reactive Protein and Ischemia in Users and Nonusers of β-Blockers and Statins. Circulation 2003;107:245-250.

19 Plenge JK, Hernandez TL, Weil KM, et al. Simvastatin Lowers C-Reactive Protein Within 14 Days. An Effect Independent of Low-Density Lipoprotein Cholesterol Reduction. Circulation 2002;106:1447-1452.

20 Lindahl B, Toss H, Siegbahn A, et al. Markers of Myocardial Damage and Inflammation in Relation to Long-Term Mortality in Unstable Coronary Artery Disease. N Eng J Med 2000;343(16):1139-1147.

21 Pearson TA, Mensah GA, Alexander RW, et al. Markers of Inflammation and Cardiovascular Disease. Application to Clinical and Public Health Practice. A Statement for Healthcare Professionals From the Centers for Disease Control and Prevention and the American Heart Association. Circulation 2003;107:499-511.

22 Price CP, Trull AK, Berry D, et al. Development and validation of a particle-enhanced turbidimetric immunoassay for C-reactive protein. J Immunol Methods 1987;99:205-211.

23 Eda S, Kaufmann J, Roos W, et al. Development of a New Microparticle-Enhanced Turbidimetric Assay for C-reactive Protein with Superior Features in Analytical Sensitivity and Dynamic Range. J Clin Lab Anal 1998;12:137-144.

24 Use of Anticoagulants in Diagnostic Laboratory Investigations. WHO Publication WHO/DIL/LAB/99.1 Rev. 2: Jan 2002.

25 Baudner S, Bienvenu J, Blirup-Jensen S, et al. The certification of a matrix reference material for immunochemical measurement of 14 human serum proteins CRM470. Report EUR 15243 EN 1993;1-186.

26 Glick MR, Ryder KW, Jackson SA. Graphical Comparisons of Interferences in Clinical Chemistry Instrumentation. Clin Chem 1986;32:470-475.

27 Breuer J. Report on the Symposium "Drug effects in Clinical Chemistry Methods". Eur J Clin Chem Clin Biochem 1996;34:385-386.

28 Sonntag O, Scholer A. Drug interference in clinical chemistry: recommendation of drugs and their concentrations to be used in drug interference studies. Ann Clin Biochem 2001;38:376-385.

29 Bakker AJ, Mücke M. Gammopathy interference in clinical chemistry assays: mechanisms, detection and prevention. Clin Chem Lab Med 2007;45(9):1240-1243.

30 Dati F, Schumann G, Thomas L, et al. Consensus of a group of professional societies and diagnostic companies on guidelines for interim reference ranges for 14 proteins in serum based on the standardization against the IFCC/BCR/CAP reference material (CRM 470). Eur J Clin Chem Clin Biochem 1996;34:517-520.

31 Ridker PM. Clinical Application of C-Reactive Protein for Cardiovascular Disease Detection and Prevention. Circulation 2003;107:363-369.

32 Schlebusch H, Liappis N, Kalina E, et al. High Sensitive CRP and Creatinine: Reference Intervals from Infancy to Childhood. J Lab Med 2002;26:341-346.

33 Bablok W, Passing H, Bender R, et al. A general regression procedure for method transformation. Application of linear regression procedures for method comparison studies in clinical chemistry, Part III. J Clin Chem Clin Biochem 1988 Nov;26(11):783-790.

A point (period/stop) is always used in this Method Sheet as the decimal separator to mark the border between the integral and the fractional parts of a decimal numeral. Separators for thousands are not used.

Symbols

Roche Diagnostics uses the following symbols and signs in addition to those listed in the ISO 15223-1 standard (for USA: see https://usdiagnostics.roche.com for definition of symbols used):

CONTENT	Contents of kit
→	Volume after reconstitution or mixing
GTIN	Global Trade Item Number

For peer review only

For peer review only - http://bmjopen.bmj.com/site/about/guidelines.xhtml

0004628918190c501V12.0

CRPHS

Cardiac C-Reactive Protein (Latex) High Sensitive

FOR US CUSTOMERS ONLY: LIMITED WARRANTY

Roche Diagnostics warrants that this product will meet the specifications stated in the labeling when used in accordance with such labeling and will be free from defects in material and workmanship until the expiration date printed on the label. THIS LIMITED WARRANTY IS IN LIEU OF ANY OTHER WARRANTY, EXPRESS OR IMPLIED, INCLUDING ANY IMPLIED WARRANTY OF MERCHANTABILITY OR FITNESS FOR PARTICULAR PURPOSE. IN NO EVENT SHALL ROCHE DIAGNOSTICS BE LIABLE FOR INCIDENTAL, INDIRECT, SPECIAL OR CONSEQUENTIAL DAMAGES.

COBAS, COBAS C, PRECICONTROL and PRECINORM are trademarks of Roche.
All other product names and trademarks are the property of their respective owners.
Additions, deletions or changes are indicated by a change bar in the margin.
© 2019, Roche Diagnostics

Roche Diagnostics GmbH, Sandhofer Strasse 116, D-68305 Mannheim
www.roche.com

Distribution in USA by:
Roche Diagnostics, Indianapolis, IN
US Customer Technical Support 1-800-428-2336

For peer review only

For peer review only - http://bmjopen.bmj.com/site/about/guidelines.xhtml

07559992500V4.0

Elecsys Folate III

cobas®

REF	[instructions for use symbol]	Σ	SYSTEM
07559992190	07559992500	100	**cobas e** 411<br>**cobas e** 601<br>**cobas e** 602

English

System information

For **cobas e** 411 analyzer: test number 1520
For **cobas e** 601 and **cobas e** 602 analyzers: Application Code Number 721

Intended use

Binding assay for the in vitro quantitative determination of folate in human serum and plasma.

The binding assay is intended for use on Elecsys and **cobas e** immunoassay analyzers.

Summary

Folate belongs to the family of B-group vitamins composed of an aromatic pteridine ring linked through a methylene group to p-aminobenzoic acid and a glutamate residue. Folate (folic acid) is vital for normal cellular functions and plays an essential role in nucleic acid synthesis, methionine regeneration, shuttling and redox reactions of one-carbon units required for normal metabolism and regulation.[1,2]

The folate metabolism can be exemplified as a cycle, where folate facilitates the transfer of one-carbon units from one molecule to another required in various biochemical reactions: for example, tetrahydrofolate (THF) accepts a single carbon unit from serine, which is reduced in a number of steps to 5-methyltetrahydrofolate (5-MTHF). 5-MTHF gives its methyl group to homocysteine, which is - with involvement of methionine synthase and vitamin B12 - enzymatically converted to methionine. The resulting THF starts again the cycle of methyl group synthesis. From methionine, the methyl groups are transferred to S-adenosylmethionine (SAM).[3] SAM serves as a methyl group donor in several methylation reactions, like DNA, RNA and protein methylation.[1]

The methionine cycle is highly sensitive to folate deficiency: with a low folate status, the ability of the cell to re-methylate homocysteine is impaired and this results in increased homocysteine concentrations in plasma.[2]

Folate also plays an essential role in the synthesis of purine and pyrimidine precursors of nucleic acids. Altered distribution of methyl groups and impaired DNA synthesis play an essential role in the development of cancers. Abnormal folate status has also been linked with the development of diseases like cardiovascular diseases, neural tube defects, cleft lip and palate, late pregnancy complications, neurodegenerative and psychiatric disorders.[1,2]

Folate belongs to the group of essential vitamins, i.e. it cannot be synthesized by the human organism and therefore must be absorbed from diet. Primary sources of folates are green and leafy vegetables, sprouts, fruits, brewer's yeast and liver.[1,2]

Folate deficiency can be caused by decreased nutritional intake, poor absorption of ingested folate in the intestine or increased demand of folate, for example during physical activity or pregnancy. Deficiency of folate can also be a result of liver diseases or impaired folate metabolism due to genetic defects or drug interactions.[2]

A clinical manifestation of both folate and vitamin B12 deficiency is the so called megaloblastic (macrocytic) anemia: due to the affected DNA synthesis and cell maturation, especially involving the cells of erythropoiesis, the total count of erythrocytes is significantly reduced. The hemoglobin synthesis capacity however is normal, which leads to abnormally large erythrocyte precursors ("macrocytes" or "megaloblasts"), which have an elevated hemoglobin content ("hyperchromic anemia").[3,4]

Because vitamin B12 and folate are closely interrelated in the cellular one-carbon unit metabolism, and also hematologic and clinical consequences of the two vitamin deficiency states might be similar, it is advisable to determine both parameters simultaneously in patients with the relevant symptoms of vitamin deficiency.[3,4]

Test principle

Competition principle. Total duration of assay: 27 minutes.

- 1st incubation: By incubating 25 µL of sample with the folate pretreatment reagents 1 and 2, bound folate is released from endogenous folate binding proteins.
- 2nd incubation: By incubating the pretreated sample with the ruthenium labeled folate binding protein, a folate complex is formed, the amount of which is dependent upon the analyte concentration in the sample.
- 3rd incubation: After addition of streptavidin-coated microparticles and folate labeled with biotin, the unbound sites of the ruthenium labeled folate binding protein become occupied, with formation of a ruthenium labeled folate binding protein-folate biotin complex. The entire complex becomes bound to the solid phase via interaction of biotin and streptavidin.
- The reaction mixture is aspirated into the measuring cell where the microparticles are magnetically captured onto the surface of the electrode. Unbound substances are then removed with ProCell/ProCell M. Application of a voltage to the electrode then induces chemiluminescent emission which is measured by a photomultiplier.
- Results are determined via a calibration curve which is instrument-specifically generated by 2-point calibration and a master curve provided via the reagent barcode or e-barcode.

Reagents - working solutions

The reagent rackpack (M, R1, R2) and the pretreatment reagents (PT1, PT2) are labeled as Fol III.

PT1 Pretreatment reagent 1 (white cap), 1 bottle, 4 mL:
Sodium 2-mercaptoethanesulfonate (MESNA) 40 g/L, pH 5.5.

PT2 Pretreatment reagent 2 (gray cap), 1 bottle, 5 mL:
Sodium hydroxide 25 g/L.

M Streptavidin-coated microparticles (transparent cap), 1 bottle, 6.5 mL:
Streptavidin-coated microparticles 0.72 mg/mL; preservative.

R1 Folate binding protein~$Ru(bpy)_3^{2+}$ (gray cap), 1 bottle, 9 mL:
Ruthenium labeled folate binding protein 75 µg/L; human serum albumin (stabilizer); borate/phosphate/citrate buffer 70 mmol/L, pH 5.5; preservative.

R2 Folate~biotin (black cap), 1 bottle, 8 mL:
Biotinylated folate 17 µg/L; biotin 120 µg/L; human serum albumin (stabilizer); borate buffer 100 mmol/L, pH 9.0; preservative.

Precautions and warnings

For in vitro diagnostic use.
Exercise the normal precautions required for handling all laboratory reagents.
Disposal of all waste material should be in accordance with local guidelines.
Safety data sheet available for professional user on request.

This kit contains components classified as follows in accordance with the Regulation (EC) No. 1272/2008:

2-methyl-2H-isothiazol-3-one hydrochloride

EUH 208 May produce an allergic reaction.

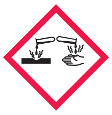

Danger

H290 May be corrosive to metals.

For peer review only

For peer review only - http://bmjopen.bmj.com/site/about/guidelines.xhtml

07559992500V4.0

Elecsys Folate III

cobas®

H314	Causes severe skin burns and eye damage.

Prevention:

P280	Wear protective gloves/ protective clothing/ eye protection/ face protection.

Response:

P301 + P330 + P331	IF SWALLOWED: Rinse mouth. Do NOT induce vomiting.
P303 + P361 + P353	IF ON SKIN (or hair): Take off immediately all contaminated clothing. Rinse skin with water.
P304 + P340 + P310	IF INHALED: Remove person to fresh air and keep comfortable for breathing. Immediately call a POISON CENTER/ doctor.
P305 + P351 + P338 + P310	IF IN EYES: Rinse cautiously with water for several minutes. Remove contact lenses, if present and easy to do. Continue rinsing. Immediately call a POISON CENTER/ doctor.
P390	Absorb spillage to prevent material damage.

Product safety labeling follows EU GHS guidance.

Contact phone: all countries: +49-621-7590

All human material should be considered potentially infectious. All products derived from human blood are prepared exclusively from the blood of donors tested individually and shown to be free from HBsAg and antibodies to HCV and HIV. The testing methods used assays approved by the FDA or cleared in compliance with the European Directive 98/79/EC, Annex II, List A.

However, as no testing method can rule out the potential risk of infection with absolute certainty, the material should be handled with the same level of care as a patient specimen. In the event of exposure, the directives of the responsible health authorities should be followed.[5,6]

Avoid foam formation in all reagents and sample types (specimens, calibrators and controls).

Reagent handling

The reagents in the kit have been assembled into a ready-for-use unit that cannot be separated.

All information required for correct operation is read in from the respective reagent barcodes.

Storage and stability

Store at 2-8 °C.

Do not freeze.

Store the Elecsys reagent kit **upright** in order to ensure complete availability of the microparticles during automatic mixing prior to use.

Stability:	
unopened at 2-8 °C	up to the stated expiration date
after opening at 2-8 °C	56 days (8 weeks)
on the analyzers	14 days (2 weeks) onboard<br>or<br>28 days (4 weeks) when stored alternatively in the refrigerator and on the analyzer, with the total time onboard on the analyzer not exceeding 10 x 8 hours

Specimen collection and preparation

Only the specimens listed below were tested and found acceptable.

Serum collected using standard sampling tubes or tubes containing separating gel.

Li-heparin plasma. Li-heparin plasma tubes containing separating gel can be used.

Criterion: Method comparison serum versus Li-heparin plasma, slope 0.9-1.1 + intercept within < ± 2x Limit of Blank (LoB), coefficient of correlation ≥ 0.95.

Serum: Stable for 2 hours at 15-25 °C, 48 hours at 2-8 °C, 28 days at -20 °C (± 5 °C). Freeze only once. Protect from light. Store the samples at 2-8 °C if they cannot be measured immediately.

Li-heparin plasma: Stable for 2 hours at 15-25 °C, 48 hours at 2-8 °C, 28 days at -20 °C (± 5 °C). Freeze only once. Protect from light. Store the samples at 2-8 °C if they cannot be measured immediately.

The sample types listed were tested with a selection of sample collection tubes that were commercially available at the time of testing, i.e. not all available tubes of all manufacturers were tested. Sample collection systems from various manufacturers may contain differing materials which could affect the test results in some cases. When processing samples in primary tubes (sample collection systems), follow the instructions of the tube manufacturer.

Samples should not subsequently be altered with additives (biocides, anti-oxidants or substances possibly changing the pH of the sample) in order to avoid erroneous folate recovery.

Centrifuge samples containing precipitates before performing the assay.

Do not use heat-inactivated samples.

Ensure the samples, calibrators and controls are at 20-25 °C prior to measurement.

Due to possible evaporation effects, samples, calibrators and controls on the analyzers should be analyzed/measured within 2 hours.

Note: Hemolysis may significantly increase folate values due to high concentrations of folate in red blood cells. Therefore, hemolyzed samples are not suitable for use in this assay. Samples for folate determinations should be collected from fasting persons.

Materials provided

See "Reagents – working solutions" section for reagents.

Materials required (but not provided)

- REF 07560001190, Folate III CalSet, for 4 x 1.0 mL
- REF 05618860190, PreciControl Varia, for 4 x 3.0 mL
- REF 11732277122, Diluent Universal, 2 x 16 mL sample diluent or REF 03183971122, Diluent Universal, 2 x 36 mL sample diluent
- General laboratory equipment
- **cobas e** analyzer

Additional materials for the **cobas e** 411 analyzer:

- REF 11662988122, ProCell, 6 x 380 mL system buffer
- REF 11662970122, CleanCell, 6 x 380 mL measuring cell cleaning solution
- REF 11930346122, Elecsys SysWash, 1 x 500 mL washwater additive
- REF 11933159001, Adapter for SysClean
- REF 11706802001, AssayCup, 60 x 60 reaction cups
- REF 11706799001, AssayTip, 30 x 120 pipette tips
- REF 11800507001, Clean-Liner

Additional materials for **cobas e** 601 and **cobas e** 602 analyzers:

- REF 04880340190, ProCell M, 2 x 2 L system buffer
- REF 04880293190, CleanCell M, 2 x 2 L measuring cell cleaning solution
- REF 03023141001, PC/CC-Cups, 12 cups to prewarm ProCell M and CleanCell M before use
- REF 03005712190, ProbeWash M, 12 x 70 mL cleaning solution for run finalization and rinsing during reagent change
- REF 03004899190, PreClean M, 5 x 600 mL detection cleaning solution
- REF 12102137001, AssayTip/AssayCup, 48 magazines x 84 reaction cups or pipette tips, waste bags
- REF 03023150001, WasteLiner, waste bags
- REF 03027651001, SysClean Adapter M

Additional materials for all analyzers:

For peer review only - http://bmjopen.bmj.com/site/about/guidelines.xhtml

07559992500V4.0

Elecsys Folate III

cobas®

- REF 11298500316, ISE Cleaning Solution/Elecsys SysClean, 5 x 100 mL system cleaning solution

Assay

For optimum performance of the assay follow the directions given in this document for the analyzer concerned. Refer to the appropriate operator's manual for analyzer-specific assay instructions.

Resuspension of the microparticles takes place automatically prior to use. Read in the test-specific parameters via the reagent barcode. If in exceptional cases the barcode cannot be read, enter the 15-digit sequence of numbers.

cobas e 601 and **cobas e** 602 analyzers: PreClean M solution is necessary.

Bring the cooled reagents to approximately 20 °C and place on the reagent disk (20 °C) of the analyzer. Avoid foam formation. The system automatically regulates the temperature of the reagents and the opening/closing of the bottles.

Calibration

Traceability: This method has been standardized against the WHO International Standard NIBSC code: 03/178.

Every Elecsys reagent set has a barcoded label containing specific information for calibration of the particular reagent lot. The predefined master curve is adapted to the analyzer using the relevant CalSet.

Calibration frequency: Calibration must be performed once per reagent lot using fresh reagent (i.e. not more than 24 hours since the reagent kit was registered on the analyzer).

Calibration interval may be extended based on acceptable verification of calibration by the laboratory.

Renewed calibration is recommended as follows:

- after 1 month (28 days) when using the same reagent lot
- after 7 days (when using the same reagent kit on the analyzer)
- as required: e.g. quality control findings outside the defined limits

Quality control

For quality control, use PreciControl Varia.

In addition, other suitable control material can be used.

Controls for the various concentration ranges should be run individually at least once every 24 hours when the test is in use, once per reagent kit, and following each calibration.

The control intervals and limits should be adapted to each laboratory's individual requirements. Values obtained should fall within the defined limits. Each laboratory should establish corrective measures to be taken if values fall outside the defined limits.

If necessary, repeat the measurement of the samples concerned.

Follow the applicable government regulations and local guidelines for quality control.

Calculation

The analyzer automatically calculates the analyte concentration of each sample (either in nmol/L or ng/mL).

Conversion factors: nmol/L x 0.44 = ng/mL
ng/mL x 2.27 = nmol/L

Limitations - interference

The assay is unaffected by icterus (bilirubin ≤ 496 µmol/L or ≤ 29 mg/dL), lipemia (Intralipid ≤ 1500 mg/dL), biotin (≤ 86.1 nmol/L or ≤ 21 ng/mL), IgG ≤ 16 g/L, IgA ≤ 4.0 g/L and IgM ≤ 10 g/L.

Criterion: Recovery within ± 10 % of initial value with samples > 4 ng/mL and ≤ ± 0.4 ng/mL with samples ≤ 4 ng/mL.

Hemolysis may significantly increase folate values due to high concentrations of folate in red blood cells. Therefore, hemolyzed samples are not suitable for use in this assay.

Samples should not be taken from patients receiving therapy with high biotin doses (i.e. > 5 mg/day) until at least 8 hours following the last biotin administration.

No interference was observed from rheumatoid factors up to a concentration of 1000 IU/mL.

In vitro tests were performed on 16 commonly used pharmaceuticals and in addition on human erythropoietin. No interference with the assay was found.

It is contraindicated to measure samples of patients receiving therapy with certain pharmaceuticals, e.g. methotrexate or leucovorin, because of the cross-reactivity of folate binding protein with these compounds.

Samples with extremely high total protein concentrations (hyperproteinemia) are not suitable for use in this assay. Hyperproteinemia may be caused by, but not limited to, the following conditions: Lymphoma[7,8], bone marrow disorders such as multiple myeloma, monoclonal gammopathy of undetermined significance (MGUS), Waldenström macroglobulinemia, plasmocytoma[7,8,9,10,11,12,13], Amyloidosis[13,14]. Respective samples may lead to the formation of protein gel in the assay cup, which may cause a run abort. The critical total protein concentration is dependent upon the individual sample composition.

In rare cases, interference due to extremely high titers of antibodies to streptavidin and ruthenium can occur. These effects are minimized by suitable test design.

For diagnostic purposes, the results should always be assessed in conjunction with RBC folate, the patient's medical history, clinical examination, and other findings.

Limits and ranges

Measuring range

0.6-20.0 ng/mL or 1.36-45.4 nmol/L (defined by the Limit of Blank and the maximum of the master curve). Values below the Limit of Blank are reported as < 0.6 ng/mL (< 1.36 nmol/L). Values above the measuring range are reported as > 20.0 ng/mL (> 45.4 nmol/L).

Lower limits of measurement

Limit of Blank, Limit of Detection and Limit of Quantitation

Limit of Blank = 0.6 ng/mL (1.36 nmol/L)

Limit of Detection = 1.2 ng/mL (2.72 nmol/L)

Limit of Quantitation = 2.0 ng/mL (4.54 nmol/L)

The Limit of Blank, Limit of Detection and Limit of Quantitation were determined in accordance with the CLSI (Clinical and Laboratory Standards Institute) EP17-A requirements.

The Limit of Blank is the 95th percentile value from n ≥ 60 measurements of analyte-free samples over several independent series. The Limit of Blank corresponds to the concentration below which analyte-free samples are found with a probability of 95 %.

The Limit of Detection is determined based on the Limit of Blank and the standard deviation of low concentration samples. The Limit of Detection corresponds to the lowest analyte concentration which can be detected (value above the Limit of Blank with a probability of 95 %).

The Limit of Quantitation is defined as the lowest amount of analyte in a sample that can be accurately quantitated with a total allowable relative error of ≤ 20 %.

It has been determined using low concentration folate samples.

Dilution

Samples with folate concentrations above the measuring range can be diluted manually with Diluent Universal. The recommended dilution is 1:2. The concentration of the diluted sample must be > 8.5 ng/mL or > 19.3 nmol/L. After manual dilution, multiply the results by the dilution factor 2.

Expected values

Referring to "The American Journal of Clinical Nutrition"[15] serum folate (folic acid) values were found as follows:

Sex	Age	N	Median		2.5th-97.5th percentile	
	years		ng/mL	nmol/L	ng/mL	nmol/L
Both	all	23345	13.0	29.5	4.6-34.8	10.4-78.9
Male	all	11387	12.3	27.9	4.5-32.2	10.2-73.0
Female	all	11958	13.6	30.1	4.8-37.3	10.9-84.5
Both	4-11	3595	17.2	39.0	8.6-37.7	19.5-85.4
Both	12-19	6390	12.1	27.4	5.0-27.2	11.3-61.6
Both	20-59	8689	11.6	26.3	4.4-31.0	10.0-70.2

For peer review only - http://bmjopen.bmj.com/site/about/guidelines.xhtml

07559992500V4.0

Elecsys Folate III

Sex	Age	N	Median		2.5th-97.5th percentile	
	years		ng/mL	nmol/L	ng/mL	nmol/L
Both	≥ 60	4671	16.6	37.6	5.6-45.8	12.7-103.8

These values were obtained in the USA during the National Health and Nutrition Examination Survey (NHANES), 1999-2004.

The values shown below were performed on samples from an apparently healthy population, using the Elecsys Folate III assay.

The calculation is based on 404 sera (177 men, 227 women). The age range was between 20 and 65 years. Pregnant or lactating women were excluded. The reference population was selected according to normal homocysteine values.

N	Median		2.5th-97.5th percentile	
	ng/mL	nmol/L	ng/mL	nmol/L
404	8.94	20.3	3.89-26.8	8.83-60.8

Please note: These values should only be used as a guideline.

It should be taken into consideration that differences in the expected values may exist with respect to population and dietary status.

Each laboratory should investigate the transferability of the expected values to its own patient population and if necessary determine its own reference ranges.

Folate deficient sample values

25 samples considered to be deficient[a)] in serum folate concentration were assessed using the Elecsys Folate III assay. All samples were found to be below the 2.5th percentile as given in the table above.

a) Folate deficiency was assessed by measurement of serum folate by two commercially available folate assays.

Specific performance data

Representative performance data on the analyzers are given below. Results obtained in individual laboratories may differ.

Precision

Precision was determined using Elecsys reagents, pooled human sera and controls in a protocol (EP5-A2) of the CLSI (Clinical and Laboratory Standards Institute): 2 runs per day in duplicate each for 21 days (n = 84). The following results were obtained:

**cobas e** 411 analyzer					
		**Repeatability**		**Intermediate precision**	
Sample	Mean	SD	CV	SD	CV
	ng/mL	ng/mL	%	ng/mL	%
Human serum 1	1.88	0.150	8.0	0.205	10.9
Human serum 2	3.92	0.200	5.1	0.318	8.1
Human serum 3	11.9	0.346	2.9	0.571	4.8
Human serum 4	13.4	0.301	2.2	0.574	4.3
Human serum 5	17.8	0.440	2.5	0.666	3.7
PreciControl Varia1	3.24	0.215	6.6	0.309	9.5
PreciControl Varia2	11.6	0.314	2.7	0.566	4.9

**cobas e** 411 analyzer					
		**Repeatability**		**Intermediate precision**	
Sample	Mean	SD	CV	SD	CV
	nmol/L	nmol/L	%	nmol/L	%
Human serum 1	4.27	0.341	8.0	0.465	10.9
Human serum 2	8.90	0.454	5.1	0.722	8.1
Human serum 3	27.0	0.785	2.9	1.30	4.8
Human serum 4	30.4	0.683	2.2	1.30	4.3
Human serum 5	40.4	0.999	2.5	1.51	3.7
PreciControl Varia1	7.35	0.488	6.6	0.701	9.5
PreciControl Varia2	26.3	0.713	2.7	1.28	4.9

**cobas e** 601 and **cobas e** 602 analyzers					
		**Repeatability**		**Intermediate precision**	
Sample	Mean	SD	CV	SD	CV
	ng/mL	ng/mL	%	ng/mL	%
Human serum 1	1.66	0.255	15.4	0.268	16.1
Human serum 2	4.10	0.219	5.4	0.303	7.4
Human serum 3	11.1	0.449	4.1	0.503	4.6
Human serum 4	12.2	0.454	3.7	0.467	3.8
Human serum 5	16.4	0.502	3.1	0.625	3.8
PreciControl Varia1	2.34	0.189	8.1	0.228	9.8
PreciControl Varia2	10.1	0.443	4.4	0.489	4.9

**cobas e** 601 and **cobas e** 602 analyzers					
		**Repeatability**		**Intermediate precision**	
Sample	Mean	SD	CV	SD	CV
	nmol/L	nmol/L	%	nmol/L	%
Human serum 1	3.77	0.579	15.4	0.608	16.1
Human serum 2	9.31	0.497	5.4	0.688	7.4
Human serum 3	25.2	1.02	4.1	1.14	4.6
Human serum 4	27.7	1.03	3.7	1.06	3.8
Human serum 5	37.2	1.14	3.1	1.42	3.8
PreciControl Varia1	5.31	0.429	8.1	0.518	9.8
PreciControl Varia2	22.9	1.01	4.4	1.11	4.9

Method comparison

a) A comparison of the Elecsys Folate III assay (traceable to WHO IS 03/178; y) and the Elecsys Folate III assay prior to standardization against WHO IS 03/178 (x) using clinical samples gave the following correlations (ng/mL):

Number of samples measured: 113

Passing/Bablok[16]	Linear regression
y = 1.14x - 1.97	y = 1.11x - 1.77
τ = 0.939	r = 0.994

The sample concentrations were between 2.1 and 18 ng/mL (4.8 and 41 nmol/L).

b) A comparison of the Elecsys Folate III assay (y) with a commercially available method (x) using clinical samples gave the following correlations (ng/mL):

Number of samples measured: 106

Passing/Bablok[16]	Linear regression
y = 0.980x - 0.095	y = 1.09x - 0.659
τ = 0.924	r = 0.984

The sample concentrations were between 1.9 and 17 ng/mL (4.3 and 39 nmol/L).

For peer review only - http://bmjopen.bmj.com/site/about/guidelines.xhtml

07559992500V4.0

Elecsys Folate III

cobas®

c) A comparison of the Elecsys Folate III assay on the **cobas e** 601 analyzer (y) with the Elecsys Folate III assay on the **cobas e** 411 analyzer (x) using clinical samples gave the following correlations (ng/mL):

Number of samples measured: 105

Passing/Bablok[16]	Linear regression
y = 1.05x - 0.303	y = 0.981x + 0.143
τ = 0.868	r = 0.982

The sample concentrations were between 1.6 and 19 ng/mL (3.6 and 43 nmol/L).

Analytical specificity

The following cross-reactivities were found, tested with folate concentrations of approximately 3.5 ng/mL, 10 ng/mL and 19 ng/mL.

Cross-reactant	Concentration tested ng/mL	Cross-reactivity %
Amethopterin	750	2.5
Aminopterin	750	4.4
Folinic acid	750	0.7

References

1 Nazki FH, Sameer AS, Ganaie BA. Folate: Metabolism, genes, polymorphisms and the associated diseases. Gene 2014;533(1):11-20.

2 Scaglione F, Panzavolta G. Folate, folic acid and 5-methyltetrahydrofolate are not the same thing. Xenobiotica 2014;44(5):480-488.

3 Reynolds EH. The neurology of folic acid deficiency. Handb Clin Neurol 2014;120:927-43.

4 Wick M, Pinggera W, Lehmann P. Clinical Aspects and Laboratory. Iron metabolism, Anemias. Springer Verlag, Wien, New York, 6th edition 2011:41-42.

5 Occupational Safety and Health Standards: Bloodborne pathogens. (29 CFR Part 1910.1030). Fed. Register.

6 Directive 2000/54/EC of the European Parliament and Council of 18 September 2000 on the protection of workers from risks related to exposure to biological agents at work.

7 Wu AHB. Tietz clinical guide to laboratory tests, 4th ed. St. Louis, Saunders/Elsevier 2006:608-609, 916-917.

8 Paricaud K, Moulis G, Combis MS, et al. Causes of protidemia above 100 g/L. Eur J Intern Med 2014;25:e123.

9 Filippatos TD, Liamis G, Christopoulou F, et al. Ten common pitfalls in the evaluation of patients with hyponatremia. Eur J Intern Med 2016;29:22-25.

10 Mailankody S, Landgren O. Monoclonal gammopathy of undetermined significance and Waldenström's macroglobulinemia. Best Pract Res Clin Haematol 2016;29:187-193.

11 Morel P, Duhamel A, Gobbi P, et al. International prognostic scoring system for Waldenström macroglobulinemia. Blood 2009;113:4163-4170.

12 Rajkumar SV. Multiple Myeloma. Curr Probl Cancer 2009;33:7-64.

13 Gertz MA. Immunoglobulin light chain amyloidosis: 2016 update on diagnosis, prognosis, and treatment. Am J Hematol 2016;91:947-956.

14 Wu AHB. Tietz clinical guide to laboratory tests, 4th ed. St. Louis, Saunders/Elsevier 2006: 916-917, 925.

15 Pfeiffer CM, Johnson CL, Jain RB, et al. Trends in blood folate and vitamin B-12 concentrations in the United States, 1988-2004. Am J Clin Nutr 2007;86:718-727.

16 Bablok W, Passing H, Bender R, et al. A general regression procedure for method transformation. Application of linear regression procedures for method comparison studies in clinical chemistry, Part III. J Clin Chem Clin Biochem 1988 Nov;26(11):783-790.

For further information, please refer to the appropriate operator's manual for the analyzer concerned, the respective application sheets, the product information and the Method Sheets of all necessary components (if available in your country).

A point (period/stop) is always used in this Method Sheet as the decimal separator to mark the border between the integral and the fractional parts of a decimal numeral. Separators for thousands are not used.

Symbols

Roche Diagnostics uses the following symbols and signs in addition to those listed in the ISO 15223-1 standard (for USA: see dialog.roche.com for definition of symbols used):

CONTENT	Contents of kit
SYSTEM	Analyzers/Instruments on which reagents can be used
REAGENT	Reagent
CALIBRATOR	Calibrator
→	Volume after reconstitution or mixing
GTIN	Global Trade Item Number

COBAS, COBAS E, ELECSYS and PRECICONTROL are trademarks of Roche. INTRALIPID is a trademark of Fresenius Kabi AB.

All other product names and trademarks are the property of their respective owners.

Additions, deletions or changes are indicated by a change bar in the margin.

© 2019, Roche Diagnostics

CE

Roche Diagnostics GmbH, Sandhofer Strasse 116, D-68305 Mannheim
www.roche.com

Roche

For peer review only - http://bmjopen.bmj.com/site/about/guidelines.xhtml

Data collection tool
Household Listing Form

FNS/1
FNS EA no_______

Serial no of structure	Address description of structure	Residence (Y/N)	Serial no House/HH in the structure	Name of HH head	Occupied (Y/N)	Serial no occupied EA	Remark(mark "X" for selected HH)

For peer review only

For peer review only - http://bmjopen.bmj.com/site/about/guidelines.xhtml

Annex 2: Nutrition direct and indirect interventions/indicators assessment questionnaire

Nutrition direct and indirect Interventions Questionnaire

Module 1: Household identifier, characteristics and socio-demographic status

<table>
<tr><th colspan="7">Household identifier and characteristics</th></tr>
<tr><td>101</td><td>Region Code</td><td colspan="5">|__||__|</td></tr>
<tr><td>102</td><td>Woreda Code</td><td colspan="5">|__||__|</td></tr>
<tr><td>103</td><td>Kebele Code</td><td colspan="5">|__||__|</td></tr>
<tr><td>104</td><td>Gote Code</td><td colspan="5">|__||__|</td></tr>
<tr><td>105</td><td>Household Code</td><td colspan="5">|__||__| GPS Coordinates
|__||__||__||__||__||__|</td></tr>
<tr><td rowspan="2">106</td><td rowspan="2">Unique Household Code</td><td>|__||__||__|</td><td>|__||__|</td><td>|__||__|</td><td>|__||__|</td><td>|__||__||__|</td></tr>
<tr><td>Region Code</td><td>Woreda Code</td><td>Kebele Code</td><td>EA Code</td><td>Household Code</td></tr>
<tr><td>107</td><td>Residence</td><td colspan="4">1 = Urban
2 = Rural</td><td>|__|</td></tr>
<tr><td>108</td><td>Do you own this house?</td><td colspan="4">1 = Yes
0 = No</td><td>|__|</td></tr>
<tr><td>109</td><td>What is the main material of the walls?

Observe</td><td colspan="4">1 = No walls
2 = Natural materials (cane, wood, mud, straw)
3 = Stone with mud
4 = Stone/bricks with cement
99 = Other</td><td>|__||__|</td></tr>
<tr><td>110</td><td>What is the main floor material?

Observe</td><td colspan="4">1 = Natural floor (earth/sand/dung)
2 = Rudimentary floor (wood/palm/bamboo)
3=Finished floor (polished wood/ vinyl/ tiles/cement/carpet)
99 = Other</td><td>|__||__|</td></tr>
<tr><td>111</td><td>What is the main material of the roof?
Observe</td><td colspan="4">1 = Thatch/grass or leaves
2 = Iron sheets or tiles
99 = Other</td><td>|__||__|</td></tr>
<tr><td>112</td><td>What type of fuel does your household mostly use for cooking?

Do not read list</td><td colspan="4">1 = Dung
2 = Firewood/straw
3 = Charcoal
4 = Kerosene
5 = Gas (methane/biogas)
6 = Electricity
99 = Other</td><td>|__||__|</td></tr>
<tr><td>113</td><td>Is the house connected to electricity?</td><td colspan="4">1 = Yes
0 = No</td><td>|__|</td></tr>
<tr><td>114</td><td rowspan="7">In total, how many of the following items are owned by residents of this household?

Add the household total for each item</td><td colspan="4">A kerosene lamp/pressure lamp</td><td>|__|</td></tr>
<tr><td>115</td><td colspan="4">Mobile phone</td><td>|__|</td></tr>
<tr><td>116</td><td colspan="4">Cart</td><td>|__|</td></tr>
<tr><td>117</td><td colspan="4">Bicycle</td><td>|__|</td></tr>
<tr><td>118</td><td colspan="4">Motorcycle</td><td>|__|</td></tr>
<tr><td>119</td><td colspan="4">Radio</td><td>|__|</td></tr>
<tr><td>120</td><td colspan="4">Television</td><td>|__|</td></tr>
</table>

For peer review only - http://bmjopen.bmj.com/site/about/guidelines.xhtml

121		Car/tractor/Bajaj	|__|
122	Does this household own any livestock, herds, other farm animals, or poultry?	1 = Yes<br>0 = No **(Go to→131)**	
123	In total, how many of the following animals are owned by residents of this household? **Add the household total for each item**	Milk cows, oxen or bulls?	|__|
124		Other cattle?	|__|
125		Horses, donkeys, or mules?	|__|
126		Camels	|__|
127		Goats?	|__|
128		Sheep?	|__|
129		Chickens or other poultry?	|__|
130		Beehives?	|__|
131	Does any member of this household own any agricultural land?	1 = Yes<br>0 = No	|__|
132	How often does anyone smoke inside your house?<br><br>*Would you say daily, weekly, monthly, less often than once a month, or never?*	1 = Daily<br>2 = Weekly<br>3 = Monthly<br>4 = Less once monthly<br>5 = Never	|__|
**Household head socio-demographic status**			
133	Age in years		|__||__|
134	Marital status	1. Single<br>2. Married<br>3. Divorced<br>4. Separated<br>5. Widowed	|__|
135	What is the highest level of school the head of the household completed?	1. None<br>2. Primary<br>3. Secondary<br>4. Technical/vocational certificate<br>5. Higher / university/ college<br>98.Don't know<br>99.Other (specify)_______	|__||__|
136	What is the religion of the head of the HH?	1. Orthodox<br>2. Protestant<br>3. Catholic/ other Christian<br>4. Muslim<br>5. No religion<br>98.Don't know<br>99.Other religion (specify)___________	|__||__|
137	Ethnicity	Specify	

For peer review only - http://bmjopen.bmj.com/site/about/guidelines.xhtml

For peer review only

Module 2: Child health

Now I would like to ask some questions about the health of your children born in the last 5 years. We will talk about each separately, starting with the youngest.			
201	Child's code		|__||__|
202	Mother's name	Mother's given name	
203	Mother's age		
204	Mother's education level	1=None<br>2=Primary<br>3=Secondary<br>4=Technical/vocational certificate<br>5=Higher / university/ college<br>98=Don't know<br>99. Other (specify)_________	
205	Mother's marital status	1=Single<br>2 =Married<br>3=Divorced<br>4 =Separated<br>5=Widowed	
206	Mother's religion	1=Orthodox<br>2=Protestant<br>3=Catholic/ other Christian<br>4=Muslim<br>5=No religion<br>98=Don't know<br>99=Other religion (specify)______	
207	Mother's ethnicity	Specify	
208	Child's name	Child's given name	
209	Child (NAME) sex	1 = Boy<br>2 = Girl	|__|
210	Child (NAME) age?	Age in months or age at the time of the child's death	|__||__|
211	In the last six months, was (NAME) given any vitamin A supplement?	1 = Yes<br>0 = No<br>98 = Don't know	|__||__|
212	When was the child (NAME) given the vitamin A supplement?	Specify	
213	In the last 12 months, was (NAME) given any iron tablet or syrup or supplement?	1 = Yes<br>0 = No<br>98 = Don't know	|__||__|
214	In the last 6 months, was (name) given any medicine for intestinal worms?	1 = Yes<br>0 = No<br>98 = Don't know	|__||__|
215	In the last 3 months, has any healthcare provider measured?	1 = Yes, 0 = No, 98 = Don't know	
		Weight	|__||__|
		Height/length	|__||__|
		MUAC	|__||__|
216	Has (name) had diarrhea in the last 2 weeks?	1 = Yes<br>0 = No **(Go to 224)**	|__||__|

For peer review only - http://bmjopen.bmj.com/site/about/guidelines.xhtml

		98 = Don't know	
217	Now I would like to know how much was the child given to drink during diarrhea, including breast milk. Was the child given less than usual to drink, about the same amount, or more than usual to drink?	1 = Much less<br>2 = Somewhat less<br>3 = About the same<br>4 = More<br>5 = Nothing to drink<br>98 = Don't know	|__|
218	When the child had diarrhea, was he/she given less than usual to eat, about the same amount, more than usual, or nothing to eat?	1 = Much less<br>2 = Somewhat less<br>3 = About the same<br>4 = More<br>5 = Nothing to drink<br>98 = Don't know	|__|
219	Did you seek advice or treatment for the diarrhea from any source?	1 = Yes<br>0 = No **(Go to 221)**	|__|
220	Where did you seek advice or treatment? Anywhere else?<br><br>*Probe to identify the type of source.*<br>*If unable to determine if public, private, or NGO sector, record '21' and write the name Of the place(s).*	1= Government hospital<br>2= Government health center<br>3= Government health post<br>4 = Mobile clinic<br>5 = Community health worker/ fieldworker<br>6 = Other public sector (specify)<br>7 = Private hospital<br>8 = Private clinic<br>9 = Pharmacy<br>10 = Private doctor<br>11 = Mobile clinic<br>12 = Community health worker/fieldworker<br>13 = Other private medical sector (specify)<br>14 = NGO hospital<br>15 = NGO clinic<br>16 = Other NGO medical sector (specify)<br>17 = Shop<br>18 = Traditional practitioner<br>19 = Market<br>20 = Itinerant drug seller<br>99 = Other (specify)________	|__||__|
	Was (name) given any of the following at any time since (name) started having diarrhea:	1 = Yes, 0 = No, 98 = Don't know	
221		Fluid from ORS packet	|__||__|
222		Zinc	|__||__|
223		Homemade fluid	|__||__|
224	Has (name) been ill with a fever at any time in the last 2 weeks?	1 = Yes<br>0 = No (**Go to 226**)<br>98 = Don't know	|__||__|
225	Where did you seek advice or treatment for	1= Government hospital	

For peer review only - http://bmjopen.bmj.com/site/about/guidelines.xhtml

	fever?<br>Anywhere else?<br><br>*Probe to identify the type of source.*<br>*If unable to determine if public, private, or NGO sector, record '21' and write the name Of the place(s).*	2= Government health center<br>3= Government health post<br>4 = Mobile clinic<br>5 = Community health worker/ fieldworker<br>6 = Other public sector (specify)<br>7 = Private hospital<br>8 = Private clinic<br>9 = Pharmacy<br>10 = Private doctor<br>11 = Mobile clinic<br>12 = Community health worker/fieldworker<br>13 = Other private medical sector (specify)<br>14 = NGO hospital<br>15 = NGO clinic<br>16 = Other NGO medical sector (specify)<br>17 = Shop<br>18 = Traditional practitioner<br>19 = Market<br>20 = Itinerant drug seller<br>99 = Other (specify)______	|__||__|
226	Was Child (Name) ever breastfed?	1 = Yes<br>0 = No (**Go 228**)	|__|
227	How many months the child (NAME) was breastfed?		|__|
**Anthropometric and clinical nutrition assessment**			
228	Weight		|__||__|
229	Height/length		|__||__|
230	MUAC		|__||__|
231	Presence of bilateral oedema for children 6-59 months	1 = Yes<br>0 = No	|__|
232	Bitot spot	1 = Yes<br>0 = No	

For peer review only

For peer review only - http://bmjopen.bmj.com/site/about/guidelines.xhtml

Module 3: Infant and young child feeding practices

**For children 0-23 months**			
This module is to be administered to the mother/caregiver of children born 0-23 months before the survey living with respondents. Verify that the respondent you are speaking to is the mother/caregiver of the child.			
301	Was Child (Name) ever breastfed?	1 = Yes<br>0 = No (**Go to 304)**<br>98 = Don't know	|__||__|
302	Was Child (NAME) given the first milk (colostrum) after birth?	1 = Yes<br>0 = No<br>98 = Don't know	|__||__|
303	How long after birth did you first put (NAME) to the breast, even if your breast milk did not arrive?	1 = Immediately after birth, or within 1 hour<br>2 = Between 1 and 24 hours<br>3 = More than 24 hours after delivery<br>98 = Don't know	|__||__|
304	Child (NAME) alive now?	1 = Yes<br>0 = No (**Go to 401**)	|__||__|
305	Was *(NAME)* breastfed yesterday from sunrise until today sunrise?<br>NB: Breastfeeding could be by the mother herself or by wet mother.	1 = Yes<br>0 = No (**Go to 307**)<br>98 = Don't know	|__||__|
306	*Sometimes babies are fed breast milk in different ways, for example by spoon, cup or bottle. This can happen when the mother cannot always be with her baby. Sometimes babies are breastfed by another woman, or given breast milk from another woman by spoon, cup or bottle or some other way. This can happen if a mother cannot breastfeed her own baby.*<br><br>Did *(NAME)* consume breast milk in any of these ways yesterday from sunrise until today sunrise?	1 = Yes<br>0 = No<br>98 = Don't know	|__||__|
307	Now I would like to ask you about some medicines and vitamins that are sometimes given to infants.<br><br>Was *(NAME)* given any vitamin drops or other medicines as drops yesterday from sunrise until today sunrise?	1 = Yes<br>0 = No<br>98 = Don't know	|__||__|
308	Was *(NAME)* given Lemlem or ORS in the last two weeks?	1 = Yes<br>0 = No<br>98 = Don't know	|__||__|

For peer review only - http://bmjopen.bmj.com/site/about/guidelines.xhtml

309	In the last 6 months, did any healthcare provider or community health worker talk with you about how and what to feed your child?	1 = Yes<br>0 = No<br>98 = Don't know	|__||__|
310	Now, I would like to ask you about some liquids that *(NAME)* may have had yesterday from sunrise until today sunrise?<br><br>**If yes to Q310, read the list of liquids starting with 'plain water'.**	Did *(NAME)* have any *(item from list)*?<br>1 = Yes<br>0 = No **(Go to 321)**<br>98 = Don't know	|__||__|
311	Plain water	1 = Yes<br>0 = No<br>98 = Don't know	|__||__|
312	Infant formula such as S-26?	1 = Yes<br>0 = No **(Go to 314)**<br>98 = Don't know	|__||__|
313	How many times infant formula such as S-26?		|__||__|
314	Milk such as tinned, powdered, or fresh animal milk?	1 = Yes<br>0 = No **(Go to 316)**<br>98 = Don't know	|__||__|
315	How many times milk drink?		|__||__|
316	Yogurt drink?	1 = Yes<br>0 = No<br>98 = Don't know	|__||__|
317	Chocolate flavored drink?	1 = Yes<br>0 = No<br>98 = Don't know	|__||__|
318	Sodas, malt drinks or energy drinks?	1 = Yes<br>0 = No<br>98 = Don't know	|__||__|
319	Clear broth or clear soup?	1 = Yes<br>0 = No<br>98 = Don't know	|__||__|
320	Any other liquids?	1 = Yes<br>0 = No<br>98 = Don't know	|__||__|
Now I would like to ask you about foods that (NAME) had yesterday during the day or night. I am interested in foods your child ate whether at home or somewhere else. I will ask you about different types of foods, and I would like to know whether your child ate the food even if it was combined with other foods. Please do not answer 'yes' for any food or ingredient used in a small amount to add flavor to a dish.<br><br>OTHER FOODS: Please write down other foods in this box that respondent mentioned but are not in the list below<br><br>Yesterday during the day or at night, did (NAME) eat:			
321	Did the child ate any solid or semi-solid food yesterday?		Eaten?<br>1 = Yes

For peer review only

For peer review only - http://bmjopen.bmj.com/site/about/guidelines.xhtml

			0 = No **(Go to 342)**<br>98 = Don't know
322	Yogurt, other than yogurt drink?		|__|
323	How many times did child (NAME) eat yogurt?		|__|
324	Injera, bread, rice, noodles, pasta, macaroni, porridge, or other foods made from grains such as tef, oats, maize, barley?		|__|
325	Any commercially fortified baby food like Fafa, Hilina, Cerilak, Cerifam, Mother Choice?		
326	Pumpkin, carrots, squash, or sweet potatoes that are yellow or orange inside?		|__|
327	White potatoes, white yams, bulla, kocho, manioc, cassava or any other foods made from roots?		|__|
328	Any dark green leafy vegetables (kale, dark green lettuce, moringa …)?		|__|
329	Any other vegetable?		
330	Ripe mangoes, ripe papayas (insert other local vitamin a-rich fruits)?		|__|
331	Any other fruit?		|__|
332	Liver, kidney, heart, or other organ meats?		|__|
333	Any meat, such as beef, pork, lamb, goat, chicken?		|__|
334	Egg?		|__|
335	Fresh or dried fish, shellfish, or seafood?		|__|
336	Any foods made from beans, peas, lentils, nuts, or seeds?		|__|
337	Cheese or other food made from milk?		|__|
338	Any sugary foods such as chocolates, sweets, candies, pastries, cakes, or biscuits		|__|
339	Any savory junk foods, such as crisps/chips/salted biscuits/instant noodles?		|__|
340	Any other solid, semi-solid, or soft food?		|__|
341	How many times did *(NAME)* eat solid, semi-solid, or soft foods other than liquids yesterday during the day or at night?	Fill in the number of times.<br>98 = Don't know	|__||__|
342	Did *(NAME)* drink anything from a bottle with a nipple yesterday during the day or night?	1 = Yes<br>0 = No<br>98 = Don't know	|__||__|

For peer review only - http://bmjopen.bmj.com/site/about/guidelines.xhtml

Module 4: KAP of mothers or caregivers on children's care and feeding

I am going to read you some knowledge questions about breastfeeding. Please tell me your answers on these questions.			
401	How long after birth should a baby start breastfeeding?	1 = Immediately, within 1 hour of delivery<br>2 = Some hours later but within 24 hours<br>3 = After 1 day<br>4 = After 2 days<br>5 = After >3 days<br>6 = Does not think a baby should be breastfed<br>98 = Don't know	|__||__|
402	How long should a baby receive nothing other than breast milk?	1 = From birth to six months<br>2 = Other<br>98 = Don't know	|__||__|
403	How often should a baby younger than six months be breastfed or fed with breast milk?	1 = On-demand, whenever the baby wants<br>2 = Other<br>98 = Don't know	|__||__|
404	How much should a child be fed when he/she is sick?	1 = Less frequent than usual<br>2 = Same as usual<br>3 = More than usual<br>98 = Don't know	|__||__|
405	How often should a child be fed when he/she is sick?	1 = Less frequently than usual<br>2 = Same as usual<br>3 = More frequently than usual<br>98 = Don't know	|__||__|
406	At what age should a baby first start to receive foods in addition to breast milk?	Months of age (Specify)<br>98= Don't know	|__||__|
407	At what age should children begin observing fasting days if that is their culture or religion?<br>(If <2 years, enter age in months.)	Years of age (Specify)<br>98=Don't know/remember	|__||__|
408	Have you ever heard of child stunting?	1 = Yes<br>0 = No<br>98 = Don't know	|__||__|
409	What age are children at the highest risk of becoming stunted?	Years of age (Specify)<br>Months of age (Specify)<br>98 = Don't know/remember	|__||__|
410	What are the consequences of stunting for young children?<br>Mark all that are mentioned by the respondent	1 = Higher risk of severe infectious diseases<br>2 = Poor educational performance<br>3 = Weaker immune system<br>4 = Low adult wages<br>5 = Lost productivity<br>6 = Excessive weight gain in later life	|__||__|

For peer review only

For peer review only - http://bmjopen.bmj.com/site/about/guidelines.xhtml

		7 = Increased risk of nutrition-related chronic diseases in adult life<br>8 = Increased mortality rate<br>98= Don't know<br>99 = Other	
411	Poor diet during pregnancy and the first two years of child age can cause child stunting	1 = Agree<br>2 = Do not agree<br>98 = Don't know	|__||__|
I am going to read you some statements about breastfeeding and complementary feeding made by other mothers who live in a community like yours. Please tell me if you agree with these statements. Remember, there are no correct answers! I would like to know your opinion.			
412	The colostrum (the "first yellowish milk") is not good for the baby and should be discarded	1 = Strongly disagree<br>2 = Disagree<br>3 = Agree somewhat<br>4 = Agree<br>5 = Strongly agree<br>98 = Don't know	|__||__|
413	It is good to exclusively breastfeed give a baby only breast milk and no other foods or liquids for the first six months	1 = Strongly disagree<br>2 = Disagree<br>3 = Agree somewhat<br>4 = Agree<br>5 = Strongly agree<br>98 = Don't know	|__||__|
414	If a child is sick (for example has fever/diarrhea) breastfeeding must be continued	1 = Strongly disagree<br>2 = Disagree<br>3 = Agree somewhat<br>4 = Agree<br>5 = Strongly agree<br>98 = Don't know	|__||__|
415	A child should eat eggs, cow milk, or meat even on fasting days	1 = Strongly disagree<br>2 = Disagree<br>3 = Agree somewhat<br>4 = Agree<br>5 = Strongly agree<br>98 = Don't know	|__||__|
416	Eating a meal from different food groups is not necessary until children are old enough to go to school	1 = Strongly disagree<br>2 = Disagree<br>3 = Agree somewhat<br>4 = Agree<br>5 = Strongly agree<br>98 = Don't know	|__||__|
417	It is good to feed a two years child at least four times each day	1 = Strongly disagree<br>2 = Disagree<br>3 = Agree somewhat<br>4 = Agree<br>5 = Strongly agree<br>98 = Don't know	|__||__|
418	A mother should eat nutritious food	1 = Strongly disagree	|__||__|

For peer review only - http://bmjopen.bmj.com/site/about/guidelines.xhtml

	(four) times daily from the time of pregnancy	2 = Disagree<br>3 = Agree somewhat<br>4 = Agree<br>5 = Strongly agree<br>98 = Don't know	
419	A mother should take iron folic acid tablets during pregnancy	1 = Strongly disagree<br>2 = Disagree<br>3 = Agree somewhat<br>4 = Agree<br>5 = Strongly agree<br>98 = Don't know	I__II__I
420	A mother should take iodized salt during pregnancy	1 = Strongly disagree<br>2 = Disagree<br>3 = Agree somewhat<br>4 = Agree<br>5 = Strongly agree<br>98 = Don't know	I__II__I
421	A mother should take de-worming medicines during pregnancy	1 = Strongly disagree<br>2 = Disagree<br>3 = Agree somewhat<br>4 = Agree<br>5 = Strongly agree<br>98 = Don't know	I__II__I

For peer review only

For peer review only - http://bmjopen.bmj.com/site/about/guidelines.xhtml

Module 5: Adolescent girls (10-19 Years)

Hint; This section is administered for Adolescent girls 10-19 years old. Provide a paper copy of both the informed consent and Assent Form to the respondent Read the consent (for mothers of adolescent girls) and Assent (adolescent girls) form			
501	Girl's code		|__||__|
502	Girl's name	Given name	
503	Girl's age		
504	Girl's education level	1=None<br>1=Primary<br>2=Secondary<br>3=Technical/vocational certificate<br>98=Don't know<br>99=Other (specify)	
505	Girl's marital status	1 = Single<br>2 = Married<br>3 = Divorced<br>4 = Separated<br>5 = Widowed	
506	Girl's religion	1=Orthodox<br>2=Protestant<br>3=Catholic/ other Christian<br>4=Muslim<br>5=No religion<br>98=Don't know<br>Other religion (specify)	
507	Girl's ethnicity	specify	
508	Are you currently a student	1 = Yes<br>0 = No	|__|
509	Were you given any iron/foliate tablets at school or out of school? (***show the tablet)***	1 = Yes<br>0 = No (**Go to 511)**	|__|
510	How many weeks per month have you taken the iron tablets?	Weeks per month (specify)<br>98 = don't know	|__||__|
511	Were you given any drug for intestinal worms at school or out of school in the last six months?	1 = Yes<br>0 = No	|__|
512	Have you received any nutrition counseling in the last six months?	1 = Yes<br>0 = No	|__|
513	Did you receive nutritional assessment services in health facilities when you went for any kind of health service?	1 = Yes<br>0 = No	|__|
514	Is there any food taboo for adolescent girls in your community?	1 = Yes<br>0 = No (**Go to 516**)	|__|
515	Mention types of food taboo?		
Anthropometry and clinical nutrition assessment			
516	Weight (in kg)		
517	Height (in CM)		
518	Waist circumference (in CM)		

For peer review only - http://bmjopen.bmj.com/site/about/guidelines.xhtml

519	Goiter	1 = Yes, 0 = No

For peer review only

For peer review only - http://bmjopen.bmj.com/site/about/guidelines.xhtml

Module 6: Reproductive age women (15-49 Years)

For currently pregnant women			
601	Woman's code		
602	Woman's name	Given name	
603	Woman's age		
604	Woman's education level	1=None<br>2=Primary<br>3=Secondary<br>4=Technical/vocational certificate<br>5=Higher / university/ college<br>98=Don't know<br>99=Other (specify)	
605	Woman's marital status	1 = Single<br>2 = Married<br>3 = Divorced<br>4 = Separated<br>5 = Widowed	
606	Woman's religion	1=Orthodox<br>2=Protestant<br>3=Catholic/ other Christian<br>4=Muslim<br>5=No religion<br>98=Don't know<br>99=Other religion (specify)	
607	Woman's ethnicity	Specify	
608	What was your age at first marriage	Year (specify)<br>96=Not applicable<br>98=Don't know	|__||__|
609	What was your age at your first pregnancy	Year (specify)<br>96=Not applicable<br>98=Don't know	|__||__|
610	Were you pregnant in the last 3 years?	1= Yes<br>0=No **(Go to 629)**	
611	Did you see anyone for antenatal care for the pregnancy?	1 = Yes<br>0 = No (**Go to 629**)	|__|
612	Whom did you see for antenatal care?<br>Probe to identify each type of person and record all	1=Health personnel<br>2 = Doctor<br>3 = Nurse<br>4 = Midwife<br>5= Health officer<br>6 = Health extension worker<br>7 = Other person<br>8 = Traditional birth Attendant<br>99 = Other (specify)	|__||__|
613	Where did you receive antenatal care for this pregnancy?<br>Anywhere else?	1= My home<br>2 = Her home<br>3 = Other home<br>4 = Health center	|__||__|

For peer review only - http://bmjopen.bmj.com/site/about/guidelines.xhtml

		5 = Government Hospital<br>6 = Private Clinic or Hospital<br>7 = Health post<br>8 = NGO clinic or hospital<br>99 = Other SPECIFY	
614	How many months pregnant were you when you first received antenatal care for this pregnancy?	Months (Specify)<br>98 = Don't know	|__||__|
615	How many times did you receive antenatal care during this pregnancy?	Number of times (specify)<br>98 = Don't know	|__||__|
616	As part of your antenatal care during this pregnancy, were any of the following done at least once:	1 = Yes 0 = No	
		Was your blood pressure measured?	|__|
		Did you give a urine sample?	|__|
		Did you give a blood sample?	|__|
		Did a health care provider talk with you about which foods to eat while pregnant?	|__|
		Did a health care provider weigh you?	|__|
		Did a health care provider talk with you about your weight?	|__|
		Did a health care provider talk with you about breastfeeding?	|__|
617	During this pregnancy, did you ever receive food or cash assistance from government, an NGO, religious institution or other group?	1 = Yes<br>0 = No (**Go to 619**)	|__|
618	What type of assistance did you receive?	1 = Cash only<br>2 = Food only<br>3 = Cash and food mix<br>99 = Other (specify)	|__||__|
619	During this pregnancy, were you given or did you buy any iron tablets or iron syrup?<br><br>***show tablets/syrup/multiple micronutrient supplement***	1 = Yes<br>0 = No **(Go to 622)**<br>98 = Don't Know **(Go to 622)**	|__||__|
620	Where did you get the iron tablet or syrup from?	1 = Govt. Health Facility<br>2 = Private Health Facility<br>3 = Mobile Clinic<br>4 = CHW<br>5 = [Mass Distribution Campaign – Add Local Name]<br>6 = Pharmacy<br>7 = Shop/Market<br>8 = School<br>98 = Don't Know<br>99= Other	|__||__|
621	During this pregnancy, for how many days did you take the iron tablets?<br>***If answer is not numeric, probe for***	Number of days (specify)<br>98 = Don't Know	|__||__|

For peer review only - http://bmjopen.bmj.com/site/about/guidelines.xhtml

	approximate number of days.		
622	During this pregnancy, did you take any drug for intestinal worms?	1 = Yes<br>0 = No<br>98 = Don't Know	|__||__|
623	During this pregnancy, did any health care provider talk with you about breastfeeding?	1 = Yes<br>0 = No<br>98 = Don't Know	|__||__|
624	During this pregnancy, did you practice fasting?	1 = Yes<br>0 = No<br>98 = Don't Know	|__||__|
625	At your last ANC visit, did the health provider weigh you?	1 = Yes<br>0 = No<br>98 = Don't Know	|__||__|
626	During this pregnancy has your health provider given you information about your weight gain?	1 = Yes<br>0 = No (**Go to 629**)<br>98 = Don't Know	|__||__|
627	During your pregnancy have you been thin for your height?	1 = Yes<br>0 = No<br>98 = Don't Know	|__||__|
628	Did you received treatment for malnutrition?	1 = Yes<br>0 = No	|__||__|
**Anthropometry**			
629	Weight (in kg)		
630	Height (in CM)		
631	MUAC		
632	Waist circumference (in cm)		
633	Goiter	1 = Yes<br>0 = No	

For peer review only

For peer review only - http://bmjopen.bmj.com/site/about/guidelines.xhtml

Module 7: Women Dietary Diversity

"Now I'd like to ask you about foods and drinks that you ate or drank yesterday during the day or night, whether you ate it at home or anywhere else.		
Yesterday, during the day or at night did you eat or drink:		
S.N	Question	Response<br>1 = Yes<br>0 = No
701	Woman's code	
702	Woman's name	Given name
703	Woman's age	
704	Woman's education level	0=None<br>1=Primary<br>2=Secondary<br>3=Technical/vocational certificate<br>4=Higher / university/ college<br>98=Don't know<br>99=Other (specify)
705	Woman's marital status	1= Single<br>2= Married<br>3= Divorced<br>4= Separated<br>5= Widowed
706	Woman's religion	1=Orthodox<br>2=Protestant<br>3=Catholic/other Christian<br>4=Muslim<br>5=No religion<br>98=Don't know<br>99=Other religion (specify)
707	Woman's ethnicity	Specify
708	Any vegetables or roots that are orange-colored inside, like: pumpkin, carrots, squash, or sweet potatoes that are yellow or orange inside	|__|
709	Any white roots and tubers or plantains, such as: white potatoes, white yams, manioc/cassava/yucca, cocoyam, taro or any other foods made from white-fleshed roots or tubers, or plantains	|__|
710	Any dark green leafy vegetables, such as: [list examples of any medium-to-dark green leafy vegetables including wild/foraged leaves]	|__|
711	Any fruits that are dark yellow or orange inside, like: ripe mango, ripe papaya	|__|
712	Any other fruits	|__|
713	Any other vegetables	|__|
714	Any meat made from animal organs, such as: liver, kidney, heart or other organ meats or blood-based foods, including from wild game	|__|
715	Any other types of meat or poultry, like: beef, pork, lamb, goat, rabbit, wild game meat, chicken, duck, other birds	|__|

For peer review only - http://bmjopen.bmj.com/site/about/guidelines.xhtml

716	Any eggs	|__|
717	Any fish or seafood, whether fresh or dried	|__|
718	Any beans or peas, such as: mature beans or peas (fresh or dried seed), lentils, or bean/pea products, including hummus, tofu and tempeh	|__|
719	Any nuts or seeds, like tree nut, groundnut/peanut, or certain seeds or nut/seed "butters" or pastes	|__|
720	Any milk or milk products, such as: milk, cheese, yoghurt or other milk products, but not including butter, ice cream, cream or sour cream	|__|
721	Any oils and fats	|__|
722	Any savory and fried snacks, such as: crisps and chips, fried dough, other fried snacks	|__|
723	Any sweets, such as: sugary foods, such as chocolates, candies, cookies/sweet biscuits and cakes, sweet pastries or ice cream	|__|
724	Any sugar-sweetened beverages, like: sweetened fruit juices and "juice drinks", soft drinks/fizzy drinks, chocolate drinks, malt drinks, yoghurt drinks, sweet tea or coffee with sugar	|__|
725	Any condiments and seasonings, such as: ingredients used in small quantities for flavour, such as chilies, spices, herbs, fish powder, tomato paste, flavor cubes or seeds	|__|
726	Any other beverages and foods like tela, tej, bordea, arkea, cheka, tselo	|__|
727	Did you eat anything (meal or snack) OUTSIDE of the home yesterday?	|__|
728	Did you fast yesterday during the day or night?	|__|

For peer review only

For peer review only - http://bmjopen.bmj.com/site/about/guidelines.xhtml

Module 8: SELF-REPORTING QUESTIONNAIRE (SRQ-20)

No	Question	Answer<br>1 = Yes<br>0 = No	
801	Do you often have headaches?		|__|
802	Is your appetite poor?		|__|
803	Do you sleep badly?		|__|
804	Are you easily frightened?		|__|
805	Do your hands shake?		|__|
806	Do you feel nervous, tense or worried?		|__|
807	Is your digestion poor?		|__|
808	Do you have trouble thinking clearly?		|__|
809	Do you feel unhappy?		|__|
810	Do you cry more than usual?		|__|
811	Do you find it difficult to enjoy your daily activities?		|__|
812	Do you find it difficult to make decisions?		|__|
813	Is your daily work suffering?		|__|
814	Are you unable to play a useful part in life?		|__|
815	Have you lost interest in things?		|__|
816	Do you feel that you are a worthless person?		|__|
817	Has the thought of ending your life been on your mind?		|__|
818	Do you feel tired all the time?		|__|
819	Do you have uncomfortable feelings in your stomach?		|__|
820	Are you easily tired?		|__|

For peer review only

For peer review only - http://bmjopen.bmj.com/site/about/guidelines.xhtml

Module 9: Women empowerment

901	Identify the most senior mother of the mothers who have a selected child. She is the mother who should respond to the rest of this interview from this point.		|__||__|
902	In the past major growing season (Meher) and minor growing (Belg) season, not including the current season, did you work on the family farm?	1 = Yes<br>0 = No → **Go to 913)**	|__|
903	What sort of work you did on the family farm?	1 = Home (kitchen) gardening	|__|
904		2 = Fieldwork	|__|
905		3 = Cash crop farming	|__|
906		4 = Dairy processing	|__|
907		5 = Poultry rearing	
908		6 = Raising livestock	|__|
909		7 = Fishpond/ aquaculture	|__|
910		99 = Other (specify)	|__|
911	From the work that you did on the farm did your household earn any money?	1 = Yes<br>0 = No<br>98 = Don't know	|__|
912	Who usually decides how the money you earn will be used?<br><br>**READ THE LIST.**	1 = Self<br>2 = Husband<br>3 = Self and husband jointly<br>4 = Someone else	|__|
913	Who usually makes decisions about major household purchases/sell such as cattle or livestock?<br><br>**READ THE LIST.**	1 = Self<br>2 = Husband<br>3 = Self and husband jointly<br>4 = Someone else	|__|
914	Who usually makes decisions about minor household purchases/sell such as spices/oils, soap, utensils, or daily household needs?<br><br>**READ THE LIST.**	1 = Self<br>2 = Husband<br>3 = Self and husband jointly<br>4 = Someone else	|__|
915	Who usually makes decisions about health care for your children?<br><br>**READ THE LIST.**	1 = Self<br>2 = Husband<br>3 = Self and husband jointly<br>4 = Someone else	|__|
916	Do you have husband?	1 = Yes<br>0 = No (**Go to 918**)	|__|
917	Who usually decides how the money your husband earns will be used?<br><br>**READ THE LIST.**	1 = Self<br>2 = Husband<br>3 = Self and husband jointly<br>4 = Someone else	

For peer review only

For peer review only - http://bmjopen.bmj.com/site/about/guidelines.xhtml

918	Do you have children?	1 = Yes<br>0 = No **(Go to 923)**	
919	Does your husband help you care for the children?	1 = Yes<br>0 = No **(Go to 923)**	|__|
920	Does he help care for the children almost every day, at least once a week, or rarely?	1 = Every day<br>2 = At least once a week<br>3 = Rarely	|__|
921	Does your husband help you with household chores like cooking, cleaning the house, fetching water, collecting firewood or other domestic work?	1 = Yes<br>0 = No **(Go to 923)**	|__|
922	Does he help almost every day, at least once a week, or rarely?	1 = Every day<br>2 = At least once a week<br>3 = Rarely	|__|

923	At any time during the previous major seasons (Meher) and minor season (Belg) not including the current season,<br><br>Did you:	Use improved seed varieties for any of your crops?	1 = Yes<br>0 = No<br>98 = Don't know	|__||__|
924		Keep improved varieties of livestock?	1 = Yes<br>0 = No<br>98 = Don't know	|__||__|
925		Use animal manure to improve you crops yield?	1 = Yes<br>0 = No<br>98 = Don't know	|__||__|
926		Use any other source of fertilizer on your crops?	1 = Yes<br>0 = No<br>98 = Don't know	|__||__|
927		Irrigate your crops?	1 = Yes<br>0 = No<br>98 = Don't know	|__||__|
928		Rotate your crops from one field to another when planting?	1 = Yes<br>0 = No<br>98 = Don't know	|__||__|
929		Harvest water during the rains?	1 = Yes<br>0 = No<br>98 = Don't know	|__||__|
930		Practice intercropping?	1 = Yes<br>0 = No<br>98 = Don't know	|__||__|
931	Have you ever taken any steps to reduce soil erosion on your farm?		1 = Yes<br>0 = No **(Go to 936)**<br>98 = Don't know **(Go to 936)**	|__||__|
	What steps did you take to reduce soil erosion?		For each mentioned: 1=Yes 0=No	
932			Plant trees or shrubs	|__|
933			Terracing	|__|

For peer review only - http://bmjopen.bmj.com/site/about/guidelines.xhtml

934		Use drainage system	|__|
935		Other	|__|
936	Have you received any inputs for your farm from a social/government program?	1 = Yes<br>0 = No (**Go to 1001)**	|__|
937	What farm inputs have you received?	For each mentioned: 1=Yes 0=No	
938		Seeds	|__|
939		Improved seeds	|__|
940		Livestock or poultry	|__|
941		Improved varieties of livestock/ poultry	|__|
942		Aquaculture (fish)	|__|
943		Fertilizer	|__|
944		Other	|__|

For peer review only

For peer review only - http://bmjopen.bmj.com/site/about/guidelines.xhtml

Module 10: WASH

No	Question	choices	
1001	What is the main source of drinking water for the household?<br><br>**Do not read list**	1 = Piped into dwelling<br>2 = Piped to yard/plot<br>3 = Piped to neighbour<br>4 = Public tap/standpipe<br>5 = Tube well or borehole<br>6 = Protected well<br>7 = Unprotected well<br>8 = Protected spring<br>9 = Unprotected spring<br>10 = Rainwater<br>11 = Tanker truck<br>12 = Cart with small tank<br>13 = Surface water (river/dam/ Lake/pond/stream/canal/ irrigation channel)<br>14 = Bottled water<br>99 = Other	|__||__|
1002	Do you do anything to your household water to make it safer to drink?	1 = Yes<br>0 = No (**Go to 1004)**<br>98 = Don't know (**Go to 1004)**	|__||__|
1003	What is the main thing you do to make the water safer?	1 = Boil<br>2 = Add bleach/chlorine<br>3 = Strain through a cloth<br>4 = Use water filter (ceramic/ Sand/composite/etc)<br>5 = Solar disinfection<br>6 = Let it stand and settle<br>7 = Other(specify)<br>98 = Don't know	|__||__|
1004	What kind of toilet facility do members of your household usually use? If not possible to determine, ask for Permission to observe the facility.	1 = Flush to piped sewer system<br>2 = Flush to septic tank<br>3 = Flush to pit latrine<br>4 = Flush to somewhere else<br>5 = Flush, don't know where<br>6 = Ventilated improved pit latrine<br>7 = Pit latrine with slab<br>8 = Pit latrine without slab/open pit<br>9 = Composting toilet<br>10 = Bucket toilet<br>11 = Hanging toilet/hanging latrine<br>12 = No facility/bush/field<br>99 = Other (specify)________	|__||__|
1005	How does your HH primarily dispose of HH waste?	1 = Collected by municipality<br>2 = Buried<br>3 = Collected by private establishment<br>4 = Dumped in street/open space	|__||__|

For peer review only - http://bmjopen.bmj.com/site/about/guidelines.xhtml

		5 = Disposed in the compound<br>6 = Dumped in river<br>7 = Burned<br>98 = Other	
1006	Do you have separate cooking room?	1 = Yes<br>2 = No **(Go to 1008)**	
1007	Does the stove or cooking room have a chimney?	1 = yes<br>2 = No	|__||__|
1008	What type of fuel or energy source is used in this cook stove?	1 = Alcohol/ethanol<br>2 = Gasoline/diesel<br>3 = Kerosene/paraffin<br>4 = Coal/lignite<br>5 = Charcoal<br>6 = Wood<br>7 = Straw/shrubs/grass<br>8 = Agricultural crop<br>9 = Animal dung/waste<br>10 = Processed biomass (pellets) or woodchips<br>11 = Garbage/plastic<br>12 = Sawdust<br>96 = Other (specify)	|__||__|
1009	Do you have a confined space (beret/gata) to keep livestock?	1 = Yes<br>0 = No<br>96 = Not applicable	|__|
1010	Do you keep poultry in cages/confined systems (kote)?	1 = Yes<br>0 = No<br>96 = Not applicable	|__|
1011	What do you think are the activities before which you should wash your hands with soap?<br><br>**DO NOT PROMPT.**	For each mentioned: 1=**Yes 0=No**	
1012		Before preparing food	|__|
1013		Before touching or eating food	|__|
1014		Before feeding a child or other person	|__|
1015		Praying	|__|
1016		Don't know	|__|
1017	What do you think are the activities after which you should wash your hands with soap?<br><br>**DO NOT PROMPT.**	For each mentioned: **1=Yes 0=No**	
1018		After defecation or urinating	|__|
1019		After handling animals and their waste	|__|
1020		After housework or fieldwork	|__|
1021		After touching pets or handling animals and their waste	|__|
1022		After blowing nose or coughing	|__|
1023		After cleaning a child's bottom	|__|
1024		None	|__|
1025	What do you think are the reasons to keep poultry and livestock in a confined space? For each mentioned: **1=Yes 0=No**	To keep out of house |__|	
1026		To keep away from water source	|__|
1027		To reduce infectious disease	|__|

For peer review only - http://bmjopen.bmj.com/site/about/guidelines.xhtml

1028	**DO NOT PROMPT.**	To protect livestock/poultry	**|__|**
1029		Other	

For peer review only

Module 11: Food fortification (household coverage of fortifiable foods)

SN	Question	Response	
1101	Does your household use cooking oil to prepare foods or add to foods at home?	1 = Yes<br>0 = No →**Go to 1103**	|__|
1102	The last time your household get cooking oil, where did you get it from?	1=Purchased from market/shop/kiosk/wholesaler/street vendor<br>2= Homemade or obtained from local farm or local small factory/processor….<br><br>3= Received from food aid/social protection program<br>4 = Other (specify): _____________<br><br>98= Don't know/remember	|__||__|
1103	Does your household prepare foods using wheat flour at home, such as bread, kita, injera?	1 = Yes<br>0 = No →**Go to 1105**	|__|
1104	The last time your household get wheat flour, where did you get it from?	1=Purchased from market/shop/kiosk/wholesaler/street vendor<br>2= Homemade or obtained from local farm or local small factory/processor….<br><br>3= Received from food aid/social protection program<br>4 = Other (specify): _____________<br><br>98= Don't know/remember	|__||__|
1105	I would like to check whether the salt used in your household is iodized. May I have a sample of the salt used to cook meals in your household? Test salt for iodine	1=Iodine present<br>2= No iodine<br>3= Household uses salt but there is no salt in household<br>4= Household does not use salt<br>5= Salt not tested, specify reason_________	|__|
1106	The last time your household get salt, where did you get it from?	1=Purchased from market/shop/kiosk/wholesaler/street vendor<br>2= Homemade or obtained from local farm or local small factory/processor….<br><br>3= Received from food aid/social protection program<br>98= Don't know/remember	|__||__|

For peer review only - http://bmjopen.bmj.com/site/about/guidelines.xhtml

		99 = Other (specify): _____________	

For peer review only

For peer review only - http://bmjopen.bmj.com/site/about/guidelines.xhtml

Module 12 – Agriculture practices

**About the household**			
1201	Does any member of the household own any agricultural land (purchased or own?	1 = Yes<br>0 = No (**Go to 1212)**	|__|
1202	How many hectares of agricultural land do members of this household own?<br><br>*Note: Convert local land measurement unit into hector after discussing with agriculture focal person/AEW.*	Enter total number of hectares (If less than 1, Enter in decimals (example 0.5)<br>Enter 9999 if hectares are not known	|__|__|__|__|
1203	In the past major growing season (Meher) and minor growing (Belg) season, not including the current season, did you work on the family farm?	1 = Yes<br>0 = No (**Go to 1211)**	|__|
	What sort of work did you do on the family farm?<br><br>READ THE LIST	1 = Yes 0 = No	
		1 = Home (kitchen) gardening	|__|
1204		2 = Fieldwork	|__|
1205		3 = Cash crop farming	|__|
1206		4 = Producing dairy	|__|
1207		5 = Rearing poultry	|__|
1208		6 = Raising livestock	|__|
1209		7 = Fishpond/ aquaculture	|__|
1210		99 = Other	|__|
1211	Does this household own any livestock, herds, other farm animals, or poultry?	1 = Yes<br>0 = No → **Go to 1221**	|__|
	How many of the following animals do this household own?	For each: Enter number. **If none, enter 0**	
1212		Chickens	|__|__|__|
1213		Goats	|__|__|__|
1214		Sheep	|__|__|__|
1215		Donkeys	|__|__|__|
1216		Horses	|__|__|__|
1217		Mules	|__|__|__|
1218		Camels	|__|__|__|
1219		Milk cows	|__|__|__|
1220		Oxen	|__|__|__|

For peer review only - http://bmjopen.bmj.com/site/about/guidelines.xhtml

In the past 2 growing seasons (Meher and Belg), not including the current season, please describe all the crops (cereals, legumes, vegetables, fruits, seeds, and other crops) grown on your household farm.

Please also describe all animal source foods (meat, eggs, milk, dairy, fish, other) that you have produced on your household farm in same period.

Write down all crops and animals mentioned by the respondent. When the respondent has finished, probe for crops and animal source foods not mentioned. Then ask about production/yields in the relevant units. Ask the respondent to estimate the amount of the total production that went to sales, food consumption, and storage/losses/other uses.

For peer review only

	Group	Crop	Did HH cultivate crop? 1 = yes 0 = No	Season 1=Meher 2=Belg 3=Both	Amount	During the previous Major seasons (Meher) and Minor season (Belg) not including the current season		
						Sold	Consumed	Storage, losses, animal feed or other uses
1221	Staples	Maize	|__|		|__|	|__||__|	|__||__|	|__||__|
1222		Teff	|__|		|__|	|__||__|	|__||__|	|__||__|
1223		Wheat	|__|		|__|	|__||__|	|__||__|	|__||__|
1224		Barley	|__|		|__|	|__||__|	|__||__|	|__||__|
1225		Sorghum	|__|		|__|	|__||__|	|__||__|	|__||__|
1226		Millet	|__|		|__|	|__||__|	|__||__|	|__||__|
1227		Rice	|__|		|__|	|__||__|	|__||__|	|__||__|
1228		Emmer wheat (oaths)	|__|		|__|	|__||__|	|__||__|	|__||__|
1229		Other cereals	|__|		|__|	|__||__|	|__||__|	|__||__|
1230	Pulses (legumes)	Bean	|__|		|__|	|__||__|	|__||__|	|__||__|
1231		Haricot bean	|__|		|__|	|__||__|	|__||__|	|__||__|
1232		Lentil (Miser)	|__|		|__|	|__||__|	|__||__|	|__||__|
1233		Grass pea (guaya)	|__|		|__|	|__||__|	|__||__|	|__||__|
1234		Chickpea	|__|		|__|	|__||__|	|__||__|	|__||__|
1235		Field pea (Ater)	|__|		|__|	|__||__|	|__||__|	|__||__|
1236		Soya bean	|__|		|__|	|__||__|	|__||__|	|__||__|

For peer review only - http://bmjopen.bmj.com/site/about/guidelines.xhtml

<table>
<tr><th></th><th></th><th></th><th></th><th></th><th></th><th></th><th></th><th></th></tr>
<tr><td>1237</td><td></td><td>Other legumes</td><td>|__|</td><td></td><td>|__|</td><td>|__||__|</td><td>|__||__|</td><td>|__||__|</td></tr>
<tr><td>1238</td><td>Oil Crops</td><td>Niger seed (Nug)</td><td>|__|</td><td></td><td>|__|</td><td>|__||__|</td><td>|__||__|</td><td>|__||__|</td></tr>
<tr><td>1239</td><td></td><td>Sunflower</td><td>|__|</td><td></td><td>|__|</td><td>|__||__|</td><td>|__||__|</td><td>|__||__|</td></tr>
<tr><td>1240</td><td></td><td>Sesame</td><td>|__|</td><td></td><td>|__|</td><td>|__||__|</td><td>|__||__|</td><td>|__||__|</td></tr>
<tr><td>1241</td><td></td><td>Linseed</td><td>|__|</td><td></td><td>|__|</td><td>|__||__|</td><td>|__||__|</td><td>|__||__|</td></tr>
<tr><td>1242</td><td></td><td>Rapeseed (Gomenzer)</td><td>|__|</td><td></td><td>|__|</td><td>|__||__|</td><td>|__||__|</td><td>|__||__|</td></tr>
<tr><td>1243</td><td></td><td>Lupine (Gibto)</td><td>|__|</td><td></td><td>|__|</td><td>|__||__|</td><td>|__||__|</td><td>|__||__|</td></tr>
<tr><td>1244</td><td></td><td>Nuts</td><td></td><td></td><td></td><td>|__||__|</td><td>|__||__|</td><td>|__||__|</td></tr>
<tr><td>1245</td><td></td><td>Other oil crops</td><td>|__|</td><td></td><td>|__|</td><td>|__||__|</td><td>|__||__|</td><td>|__||__|</td></tr>
<tr><td>1246</td><td>Root crops/ tubers/ vegetables</td><td>Cassava</td><td>|__|</td><td></td><td>|__|</td><td>|__||__|</td><td>|__||__|</td><td>|__||__|</td></tr>
<tr><td>1247</td><td></td><td>Enset</td><td>|__|</td><td></td><td>|__|</td><td>|__||__|</td><td>|__||__|</td><td>|__||__|</td></tr>
<tr><td>1248</td><td></td><td>Irish potato</td><td>|__|</td><td></td><td>|__|</td><td>|__||__|</td><td>|__||__|</td><td>|__||__|</td></tr>
<tr><td>1249</td><td></td><td>Sweet potato</td><td>|__|</td><td></td><td>|__|</td><td>|__||__|</td><td>|__||__|</td><td>|__||__|</td></tr>
<tr><td>1250</td><td></td><td>Sweet potato - orange flesh</td><td>|__|</td><td></td><td>|__|</td><td>|__||__|</td><td>|__||__|</td><td>|__||__|</td></tr>
<tr><td>1251</td><td></td><td>Onion</td><td>|__|</td><td></td><td>|__|</td><td>|__||__|</td><td>|__||__|</td><td>|__||__|</td></tr>
<tr><td>1252</td><td></td><td>Pepper</td><td>|__|</td><td></td><td>|__|</td><td>|__||__|</td><td>|__||__|</td><td>|__||__|</td></tr>
<tr><td>1253</td><td></td><td>Tomato</td><td>|__|</td><td></td><td>|__|</td><td>|__||__|</td><td>|__||__|</td><td>|__||__|</td></tr>
<tr><td>1254</td><td></td><td>Cabbage</td><td>|__|</td><td></td><td>|__|</td><td>|__||__|</td><td>|__||__|</td><td>|__||__|</td></tr>
<tr><td>1255</td><td></td><td>Other light green leafy vegetables</td><td>|__|</td><td></td><td>|__|</td><td>|__||__|</td><td>|__||__|</td><td>|__||__|</td></tr>
<tr><td>1256</td><td></td><td>Kale</td><td>|__|</td><td></td><td>|__|</td><td>|__||__|</td><td>|__||__|</td><td>|__||__|</td></tr>
<tr><td>1257</td><td></td><td>Other dark green leafy vegetables</td><td>|__|</td><td></td><td>|__|</td><td>|__||__|</td><td>|__||__|</td><td>|__||__|</td></tr>
<tr><td>1258</td><td></td><td>Carrot</td><td>|__|</td><td></td><td>|__|</td><td>|__||__|</td><td>|__||__|</td><td>|__||__|</td></tr>
<tr><td>1259</td><td></td><td>Other roots or tubers</td><td>|__|</td><td></td><td>|__|</td><td>|__||__|</td><td>|__||__|</td><td>|__||__|</td></tr>
<tr><td>1260</td><td></td><td>Other vegetables</td><td>|__|</td><td></td><td>|__|</td><td>|__||__|</td><td>|__||__|</td><td>|__||__|</td></tr>
<tr><td>1261</td><td>Perennial crops/ fruits</td><td>Coffee</td><td>|__|</td><td></td><td>|__|</td><td>|__||__|</td><td>|__||__|</td><td>|__||__|</td></tr>
<tr><td>1262</td><td></td><td>Chat (khat)</td><td>|__|</td><td></td><td>|__|</td><td>|__||__|</td><td>|__||__|</td><td>|__||__|</td></tr>
<tr><td>1263</td><td></td><td>Banana</td><td>|__|</td><td></td><td>|__|</td><td>|__||__|</td><td>|__||__|</td><td>|__||__|</td></tr>
<tr><td>1264</td><td></td><td>Orange</td><td>|__|</td><td></td><td>|__|</td><td>|__||__|</td><td>|__||__|</td><td>|__||__|</td></tr>
<tr><td>1265</td><td></td><td>Mango</td><td>|__|</td><td></td><td>|__|</td><td>|__||__|</td><td>|__||__|</td><td>|__||__|</td></tr>
<tr><td>1266</td><td></td><td>Hop</td><td>|__|</td><td></td><td>|__|</td><td>|__||__|</td><td>|__||__|</td><td>|__||__|</td></tr>
</table>

For peer review only

<table>
<tr><td></td><td></td><td>(Gesho)</td><td></td><td></td><td></td><td></td><td></td><td></td></tr>
<tr><td>1267</td><td></td><td>Avocado</td><td>|__|</td><td></td><td>|__|</td><td>|__||__|</td><td>|__||__|</td><td>|__||__|</td></tr>
<tr><td>1268</td><td></td><td>Lemon</td><td>|__|</td><td></td><td>|__|</td><td>|__||__|</td><td>|__||__|</td><td>|__||__|</td></tr>
<tr><td>1269</td><td></td><td>Papaya</td><td>|__|</td><td></td><td>|__|</td><td>|__||__|</td><td>|__||__|</td><td>|__||__|</td></tr>
<tr><td>1270</td><td></td><td>Guava</td><td>|__|</td><td></td><td>|__|</td><td>|__||__|</td><td>|__||__|</td><td>|__||__|</td></tr>
<tr><td>1271</td><td></td><td>Water Melon</td><td>|__|</td><td></td><td>|__|</td><td>|__||__|</td><td>|__||__|</td><td>|__||__|</td></tr>
<tr><td>1272</td><td></td><td>Tirngo fruit</td><td>|__|</td><td></td><td>|__|</td><td>|__||__|</td><td>|__||__|</td><td>|__||__|</td></tr>
<tr><td>1273</td><td></td><td>Other perennial crops</td><td>|__|</td><td></td><td>|__|</td><td>|__||__|</td><td>|__||__|</td><td>|__||__|</td></tr>
<tr><td>1274</td><td></td><td>Other fruits</td><td>|__|</td><td></td><td>|__|</td><td>|__||__|</td><td>|__||__|</td><td>|__||__|</td></tr>
</table>

For peer review only

	Group	Animal source food (unit)	Does HH produce? **1=yes** **0=no** *(If no, skip to the next item)*	**Amount**	**During the previous Major seasons (Meher) and Minor season (Belg) not including the current season**			
					Season 1=Meher 2=Belg 3=Both	**How much?**		
						Sold	Consumed	Storage, losses, animal feed or others
1275	All	Chicken eggs		|__|	|__|	|__|__|	|__|__|	|__|__|
1276		Chicken meat		|__|	|__|	|__|__|	|__|__|	|__|__|
1277		Goat milk		|__|	|__|	|__|__|	|__|__|	|__|__|
1278		Goat meat		|__|	|__|	|__|__|	|__|__|	|__|__|
1279		Camel milk		|__|	|__|	|__|__|	|__|__|	/__/__/
1280		Sheep meat		|__|	|__|	|__|__|	|__|__|	/__/__/
1281		Cow milk		|__|	|__|	|__|__|	|__|__|	/__/__/
1282		Cow other dairy		|__|	|__|	|__|__|	|__|__|	/__/__/
1283		Beef		|__|	|__|	|__|__|	|__|__|	/__/__/
1284		Other meat (e.g. camel, horse)		|__|	|__|	|__|__|	|__|__|	/__/__/
1285		Farmed fish		|__|	|__|	|__|__|	|__|__|	/__/__/

For peer review only

For peer review only - http://bmjopen.bmj.com/site/about/guidelines.xhtml

Module 13: Household food insecurity

Now I would like to ask you some questions about food. During the last 12 MONTHS, was there a time when:			
SN	Questions	Answers	
1301	You or others in your household worried about not having enough food to eat because of a lack of money or other resources?	1=Yes<br>0= No<br>98=Don't know<br>97=Refused	|__||__|
1302	Still thinking about the last 12 MONTHS, was there a time when you or others in your household were unable to eat healthy and nutritious food because of a lack of money or other resources?	1=Yes<br>0= No<br>98=Don't know<br>97=Refused	|__||__|
1303	Was there a time when you or others in your household ate only a few kinds of foods because of a lack of money or other resources?	1=Yes<br>0= No<br>98=Don't know<br>97=Refused	|__||__|
1304	Was there a time when you or others in your household had to skip a<br>meal because there was not enough money or other resources to get<br>food?	1=Yes<br>0= No<br>98=Don't know<br>97=Refused	|__||__|
1305	Still thinking about the last 12 MONTHS, was there a time when you or others in your household ate less than you thought you should because of a lack of money or other resources?	1=Yes<br>0= No<br>98=Don't know<br>97=Refused	|__||__|
1306	Was there a time when your household ran out of food because of a lack of money or other resources?	1=Yes<br>0= No<br>98=Don't know<br>97=Refused	|__||__|
1307	Was there a time when you or others in your household were hungry but did not eat because there was not enough money or other resources for food?	1=Yes<br>0= No<br>98=Don't know<br>97=Refused	|__||__|
1308	Was there a time when you or others in your household went without eating for a whole day because of a lack of money or other resources?	1=Yes<br>0= No<br>98=Don't know<br>97=Refused	|__||__|

For peer review only - http://bmjopen.bmj.com/site/about/guidelines.xhtml

Module 14: Employment and social protection

S.N.	Questions	Response	
1401	Since last year, what has been the main livelihood or income source of the HH?<br><br>**(DO NOT READ LIST. PROBE FOR ONE RESPONSE)**	1 = Sale of self-produced<br>2 = horticulture crops Sale of self-produced field crops<br>3 = Own business (including commerce, livestock rearing)<br>4 = Wage employment<br>5 = Remittances<br>6 = Property income<br>7 = Government transfers/NGO support<br>8 = Pension<br>99 = Other	|__||__|
1402	Since last year, have there been other livelihood or income sources for the HH?<br><br>**(DO NOT READ LIST. PROBE FOR ALL RESPONSES)**	1 = Sale of self-produced<br>2 = horticulture crops Sale of self-produced field crops<br>3 = Own business (including commerce, livestock rearing)<br>4 = Wage employment<br>5 = Remittances<br>6 = Property income<br>7 = Government transfers/NGO support<br>8 = Pension<br>99 = Other	|__||__|
1403	Since last year, did anyone in your HH receive any kind of food or cash assistance from the government, NGO, or other agencies? *Clarify: This is not formal employment or pension. However, it may or may not be conditional on work.*	1 = Yes<br>0 = No<br>98 = Don't know	|__||__|
1404	Since last year, which members of this HH were targeted to receive this support?	1 = All HH members<br>2 = Specific HH members<br>98 = Don't know	|__||__|
1405	Which specific HH members received food or cash assistance?<br>*Clarify: This includes children whose parents receive cash on their behalf.*	Link this back to the HH roster and have interviewer select names.<br>98 = Don't know	|__||__|
1406	Which of these categories apply to the persons who received food or cash assistance?<br><br>(READ RESPONSES ALOUD. SELECT ALL THAT APPLY)	1 = Pregnant women<br>2 = Lactating women<br>3 = Children under 5 years<br>4 = Elderly<br>5 = Disabled person<br>6 = None of the above	|__||__|
1407	Since last year, which food or social assistance program did members of the HH receive support from? (DO NOT READ LIST ALOUD. PROBE FOR ALL RESPONSES)	1 = PSNP<br>2 = Community Care Coalition<br>3 = Other assistance program<br>98 = Don't know	|__||__|
1408	Since last year, what was the form of assistance that members of your HH received form these programs: food, cash or both food and cash?	1 = Cash only transfer<br>2 = Food only transfer<br>3 = Cash and food mix<br>99 = Other<br>98 = Don't know	|__||__|

For peer review only - http://bmjopen.bmj.com/site/about/guidelines.xhtml

1409	Is this HH currently receiving food or cash?	1 = Yes<br>0 = No<br>98 = Don't know	|__||__|
1410	Since last year, how has your HH used the food received? (READ RESPONSES ALOUD. SELECT ALL THAT APPLY)	1 = HH consumption<br>2 = Sold food for cash<br>3 = Other activities<br>98 = Don't know	

For peer review only

For peer review only - http://bmjopen.bmj.com/site/about/guidelines.xhtml

1501.	How do you preserve soil fertility?<br><br>***[Multiple answer is allowed!*** *Do not read the choices. Listen and mark the one they mention*]	1 = Fertilization (Chemicals, animal manure, green manure etc)
		2 = Crop rotation (Cultivation of a series of dissimilar types of crops in the same area in sequential seasons)
		3=Intercropping (Cultivation of two or more dissimilar types of crops in the same area in the same season)
		4=Tillage
		99=Other (specify): …………………………………………………………………………………
1502.	Do you apply fertilizers?	1= Yes
		0= No (Go to 1506)
1503.	Which kinds of fertilizers do you use more often?	1= Chemical fertilizers
		2 = Organic fertilizers (non-chemicals like animal manure, green manure, compost, etc.) (Go to→1505)
1504.	Which chemical fertilizers are used most often, can you specify the type/name?	1 = UREA
		2 = DAP
		3= NPS
		98= Don't know
		99= Other (specify): ………………………………..
1505.	Which organic fertilizers are used more often, can you specify the type?	1 = Livestock manure
		2 = Poultry manure
		99=Other (specify) …………………………………………
1506.	What is the most dominant cereals/crop you produce on your farm?<br>***[Only one answer is allowed!*** *Do not read the choices. Listen, mark/specify the one they mention*]	1 = Wheat
		2 = Teff
		3 = Maize
		99= Other (Specify): …………………………………
1507.	How far is your agricultural land from your house?<br>*[In case they own many farms, Consider only the one with the dominant crop]*	1= within 500 meter radius
		2= 500 -1000 meter radius
		3= 1000 - 3000 meter radius
		4= More than 3000 meter

For peer review only - http://bmjopen.bmj.com/site/about/guidelines.xhtml

Module 15: Soil information questionnaire

Observational checklist for soil sampling

For peer review only

For peer review only - http://bmjopen.bmj.com/site/about/guidelines.xhtml

1	Region
2	Zone
3	Woreda
4	Kebele
5	Gote Code
6	Household Code
	Sample code ……………………
7	**Crop history**
7.1	Last two-year crop (Please, specify the crop harvested in 2011, growing season) …………………
7.2	Last crop (Please, specify the crop harvested from the previous growing season) …………………
7.3	Crop to be planted for the current season………………………
7.4	Please write the intended planting dates (Year and Month) ………………………,
7.5	When was the **last time** the agricultural field gets tilled?
	1 = 0 to 3 months ago
	2 = 3 to 6 months ago
	1 = 6 to 12 months ago
	2 = before a year
8	**Fertilizer utilization**
8.1	Which fertilizer is applied
	1 = Chemical fertilizer
	2 = Organic fertilizer **(Go to 8.4)**
	3 = Both 1&2
	4 = Fertilizer not applied **(Go to 9)**
8.2	Which Chemical fertilizer is applied
	1 = UREA
	2 = DAP
	3 = NPS
	4 = Other, please specify……………………………………
8.3	Please write the last date (Year and Month) you applied chemical fertilizer?…………
8.4	Which Organic fertilizer is applied
	1 = Animal Manure
	2 = Green Manure
	3 = Compost
	4 = Other, please specify……………………………………
8.5	Please write the **last date** (Year and Month) you applied organic fertilizer?…………
9	**Soil characteristics**

For peer review only

For peer review only - http://bmjopen.bmj.com/site/about/guidelines.xhtml

9.1	Observation: What is the colour of the soil you are about to sample
	1 = Dark brown/Black
	2 = Red
	3 = Grey
	4 = Other, please specify...
9.1	Observation: Field area landscape
	1 = plains/level grounds
	2 = Sloppy/Inclined
9.4	Observation: Is there a standing crop on the sampling field or to the nearby farmland.
	1 = Yes
	2 = No
9.3	Observation: Is sampling field tilled/ is it being tilled at the time of sampling.
	1 = Yes
	2 = No
10	Distance of the farmland to the nearby houses
	1 = below 100meter
	2 = 100 to 500meter
	3 = 500 to 1000meter
	4 = More than 1000meter
11	Please take picture for the surrounding environment i.e., plot, houses, anything permanent or even moving cattle
12	Please capture GPS for the sampled farmland
13	Name of sample collector..
	LIST ANY ABNORMAL CONDITIONS OR SPECIFIC INFORMATION DESIRED:

For peer review only

Module 16: A Dietary assessment questionnaire

Note for the data collectors: Among the household members, this module questionnaire is to be filled for the child under-five years of age and women of the reproductive age in the household.

Part 1: Before 24-hr recall

24-hour dietary recall			
**EA code** |__|__|__| **Household code** |__|__| **Line Number** |__|__| **Child ID** |__|__| **Unique ID Woman:** |__|__|__|__|__|__|__| **Unique ID Child:** |__|__|__|__|__|__|__|__|__| Interview Date: Date - _ _/_ _/_ _ _ _ **Day -** 01=Mon 02=Tue 03=Wed 04=Thu 05=Fri 06=Sat 07=Sun<br>Date of food intake - _ _/_ _/_ _ _ _			
	**Question**	**Coding category**	**skip**
1.	Enumerator Code:	______________________________	
2.	For which target group is the recall being done?	0. Woman 1. Child	
3.	Recall number	0. Recall 1<br>1. Recall 2	
4.	Name of the woman interviewed	____________________	
5.	Age of the woman (in complete years)	Age in years (__________)	
6.	Name of child	______________________________	
7.	Date of birth (DOB): Use Ethiopian calendar	|__|__|/|__|__|/20|__|__|	
8.	Age of the child (in complete month)	________months	
9.	Child's sex:	0=Male 1= Female	
10.	Food weighing scale number:	|__|__|	
11.	Was yesterday's food intake different from your usual diet?	1=Yes 0=No	No →13
12.	If yes,	1=Holyday/celebration<br>2=I was sick<br>3=Other	
13.	Was [child name] yesterday's food intake different from your usual diet?	1=Yes<br>0=No	No →15

For peer review only

For peer review only - http://bmjopen.bmj.com/site/about/guidelines.xhtml

14.	If yes,	1=Holyday/celebration<br>2=I was sick<br>3=Other	
15.	Did you take medicine/supplement yesterday?	1=Yes<br>0=No<br>***If yes, name:*** ___________________	
16.	Did [child name] take medicine/supplement yesterday?	1=Yes<br>0=No<br>***If yes, name:***_________________	

For peer review only

Procedures to collect the required information

1. **Pass 1: list all foods and drinks consumed during the 24-hour period.**
Now I would like to ask you about the foods and drinks that [YOU/ YOUR CHILD] consumed yesterday from the time you work up until you went to sleep, sunrise yesterday to sunrise today. Please list all foods or drinks you ate, weather you ate or drank them at home or somewhere else. Please think about snacks and small meals as well as main meals.

1) "WHAT WAS THE FIRST THING [you/ your child] ATE YESTERDAY AFTER SUNRISE?"
2) "WHEN WAS THAT"
3) "DID [you/your child] HAVE ANYTHING WITH THAT?
4) "WHAT DID [you/he/she] HAVE?"
5) "WHAT IS THE NEXT THING [you/ your child] ATE OR DRANK AND WHEN WAS THAT?"
6) REPEAT questions 3-5 until you have a full record for both DAY AND NIGHT
   a. The reference period is from sunrise yesterday to sunrise this morning. If they wake up at a different time than sunrise, you can use the time from waking up yesterday until waking up today

2. **Pass 2: get more detail about each food**.
7) "NOW, PLEASE DESCRIBE EACH FOOD [you/ your child] ATE YESTERDAY"
8) "WHAT TYPE WAS IT?"
9) "WHERE DID YOU GET IT?"
10) "WHAT ARE THE INGREDIENTS?"
    a. Use standard "probes" (probing questions) to get these details for each food.
11) "HOW MUCH DID THIS RECIPE MAKE?" or "WHAT WAS THE TOTAL AMOUNT THIS MADE?"
12) "HOW WAS The Recipe PREPARED?"
    a. Identify the cooking methods used (particularly if raw, fermented, or fried in oil).

3. **Pass 3: estimate the amount consumed of each food on the list**

13) "HOW MUCH OF [name the first food] DID [you/ your child] CONSUME?"
    a. Help the mother remember and **estimate the amount** of each food or recipe that her child ate and that she herself ate.

14) "WAS ANY LEFT OVER?"
    a. If any food is leftover from what the mother served to the child, enter that amount.

15) "PLEASE HELP ME ESTIMATE THE AMOUNT OF FOOD YOU ARE OR USED IN THE RECIPE"

    Use following portion size estimation method to estimate the amount of food/ingredient eaten or used in a recipie 1. Direct weight (g) 2.Proxy weight (g) 3. Water (g) 4. Number 5. Other (specify).

4. **Pass 4: verify everything consumed**
    a. Quickly read the information back to the respondent, "HAVE I FORGOTTEN TO ADD ANYTHING?"

For peer review only - http://bmjopen.bmj.com/site/about/guidelines.xhtml

Part 2. Quick list

Pass 1

Please describe the foods the foods and drinks that [YOU/ YOUR CHILD] consumed yesterday from the time you work up until you went to sleep (sunrise yesterday to sunrise today). Please list all foods or drinks you ate, weather you ate or drank them at home or somewhere else. Please think about snacks and small meals as well as main meals.

Write down all foods and drinks mentioned. When composite dishes are mentioned, ask for the list of ingredients
When the respondent has finished, probe for meals and snacks not mentioned.

For peer review only

Early morning	Mid-morning	Noon	Afternoon	Evening	Late evening

For peer review only - http://bmjopen.bmj.com/site/about/guidelines.xhtml

For peer review only

For peer review only - http://bmjopen.bmj.com/site/about/guidelines.xhtml

Food No.	What was the first thing [YOU/ YOUR CHILD] ate or drank after sunrise yesterday? Any else?	Time of meal	Please describe this food / beverage/ ingredient:	How was this prepared ?	Place of preparation	How was the food / Ing. measured	Amount served	Amount left over	Amount eaten	Recipe information			
										State of each ingredient	Cooking method of the recipe	Total amount of recipe prepared	Links to food/ recipes
1													
	Ingredient:		Description										
					NA								
					NA								
					NA								
					NA								
					NA								
2													
					NA								
					NA								
					NA								
					NA								
					NA								
3													
					NA								
					NA								
					NA								
					NA								
					NA								

Time of meal: 1. Early morning 2. Mid-Morning 3. Noon 4. Afternoon 5. Evening 6. Late evening

Place of preparation: 1. Home 2. Outside home

How was it prepared: 1=raw/ no change/ as purchased; 2=fermented; 3=fried; 04=cooked or boiled – wet heat; 5=baked/ grilled/ broiled – dry heat; 6=local miller; 7=blanched (dipped in boiling water); 8=other

For peer review only - http://bmjopen.bmj.com/site/about/guidelines.xhtml

For peer review only

Food No.	What was the first thing [YOU/ YOUR CHILD] ate or drank after sunrise yesterday? Any else?	Time of meal	Please describe this food / beverage/ ingredient:	How was this prepared?	Place of preparation	How was the food / Ing. measured	Amount served	Amount left over	Amount eaten	Recipe information			
										State of each ingredient	Cooking method of the recipe	Total amount of recipe prepared	Links to food/ recipes
1													
	Ingredient:		Description										
					NA								
					NA								
					NA								
					NA								
					NA								
2													
					NA								
					NA								
					NA								
					NA								
					NA								
3													
					NA								
					NA								
					NA								
					NA								

For peer review only - http://bmjopen.bmj.com/site/about/guidelines.xhtml

	What was the first	Time of	Please describe this food /	How was	Place of	Ho	Am	Am	Am	Recipe information			
					NA								

Time of meal: 1. Early morning 2. Mid-Morning 3. Noon 4. Afternoon 5. Evening 6. Late evening

Place of preparation: 1. Home 2. Outside home

How was it prepared: 1=raw/ no change/ as purchased; 2=fermented; 3=fried; 04=cooked or boiled – wet heat; 5=baked/ grilled/ broiled – dry heat; 6=local miller; 7=blanched (dipped in boiling water); 8=other

For peer review only

For peer review only - http://bmjopen.bmj.com/site/about/guidelines.xhtml

Module 17: Biomarkers collection tools

PRESCHOOL AGE CHILDREN (6-59 MONTHS)
ETHIOPIAN FOOD AND NUTRITION STRATEGY BASELINE SURVEY 2020/21
Biochemical and Health Related Data Collection Tool

IDENTIFICATION	
**HH00**. CLUSTER (EA) NAME................................	**HH01**. CLUSTER NUMBER: ☐☐☐
**HH02**. HH NUMBER: ☐☐	**HH03**. RESIDENCE (RURAL=1, URBAN=2): ☐☐
**HH04**. RESPONDENT LINE NUMBER: (SHOULD BE MOTHER/CAREGIVER) ☐☐	**HH05** CHILD LINE NUMBER ☐☐
**HH06. INTERVIEWER NAME__________________ CODE:____________________**	**HH07. TEAM LEADER, NAME:__________________ CODE:____________________**
**HH08. SUPERVISOR NAME:___________________ CODE.____________________**	

PRESCHOOL CHILDREN 6-59 MONTHS OLD

PART I: CHILD HEALTH QUESTIONS

I would like to ask you some health and food questions about your child. Fill or Circle the correct answer

S.N	Questions	Response	SKIP
1	**What is the birth date of the child? In day/month/ year (How many months old is this child?)**<br><br>NOTE FOR INTERVIEWERS<br>*(Screening question to verify that the date of birth of the child)*	Birth Date:<br>____________<br><br>(Day/Month/Year)<br><br>Age in months ☐☐	
2	**Has (**child's name**) been diagnosed with anemia in the past 6 months?**	No.............................0<br><br>Yes ....1<br>Don't know.............98	

For peer review only - http://bmjopen.bmj.com/site/about/guidelines.xhtml

PART II: CHILD BIOCHEMICAL MEASUREMENT

Consent given for: (Y OR N) **PL01** Blood ☐ **PL02** Stool ☐	
**PL03** Code for Laboratory Technician: ☐☐	Lab Tech Name ____________________
**PL04** BLUE TOP TUBE (METAL FREE)<br>Not collected =00.0 Refused = 77.7	ML. ☐☐ • ☐
**PL05** PURPLE TOP TUBE (EDTA)<br>Not collected =00.0<br>Refused = 77.7	ML. ☐☐ • ☐
**PL06** RED TOP TUBE (EDTA)<br>Not collected =00.0<br>Refused = 77.7	ML. ☐☐ • ☐
**PL07** Date blood sample taken (Ethiopian Day/Month/Year)	Date: ______/_______/__________<br>Day / Month / Year
**PL08** TIME BLOOD DRAW (Ethiopian time)	Blood draw ___ ___ : ___ __ Hour Minute
**PL09 When did you eat your most recent meal (food)?** (Ethiopian time)	___ ___ : ___ ___<br>Hour Minute
**PL10** MALARIA RESULTS (RDK)	NEGATIVE..0<br>POSITIVE P FALCIPARUM ....1<br>Positive P VIVAX.................................2<br>INVALID..3
**PL11** FEVER in last 24 HR?	NO...0<br>YES 1
**PL12 HEMOGLOBIN** RESULTS	g/dL ☐☐ • ☐
**In order to determine if you have worms in the stool, we would like to collect a stool sample from your child. If you can provide this now, we appreciate it. If not now, we can come back to pick up the sample at a later time.**<br>*INSTRUCTIONS IF UNABLE TO PRODUCE AT WILL*:<br>**For stool**: We will return tomorrow to pick up your stool. We would like the freshest stool you can give us. Please use one cup to collect the first stool you pass.	
**PL13** STOOL COLLECTED?	NO............................0 YES 1
**PL14** Date stool sample taken (Ethiopian Day/Month/Year)	Date: ______/_______/__________<br>Day / Month / Year
**PL15** TIME: STOOL COLLECTED (Ethiopian time)	___ ___ : ___ ___

For peer review only - http://bmjopen.bmj.com/site/about/guidelines.xhtml

	Hour Minute
**PL16** TIME: STOOL PASSED, Ethiopian time (as recorded on cup)	___ ___ : ___ ___<br>Hour Minute
**PL17 Time Blood centrifuged (Ethiopian time)**	___ ___ : ___ ___<br>Hour Minute

Thank you for completing this interview.

INTERVIEWER'S OBSERVATIONS
TO BE FILLED IN AFTER COMPLETING INTERVIEW

COMMENTS ABOUT RESPONDENT:

____
____
____
____
____
____
____
____

SCHOOL AGE CHILDREN 6-12 YEARS
ETHIOPIAN FOOD AND NUTRITION STRATEGY BASELINE SURVEY 2020/21
Biochemical and Health Related Data Collection Tool

IDENTIFICATION	
**SG01**. CLUSTER NUMBER: ☐☐☐	
**SG02**. HH NUMBER: ☐☐	
**SG03**. RESPONDENT LINE NUMBER: (SHOULD BE MOTHER/CAREGIVER) ☐☐	
**SG04** SCHOOL CHILD LINE NUMBER ☐☐	

In general, for children 6-10 years of age: get parental report (ask the questions of the caretaker and enter the child's name into the parentheses)
For children 11-12 years of age who are present and can provide information: get self-report (ask questions directly of the child and enter "you" or "yourself" into the parentheses)

For peer review only - http://bmjopen.bmj.com/site/about/guidelines.xhtml

PART I: CHILD HEALTH RELATED QUESTIONS

No.	QUESTION	CODING CATEGORIES		SKIP
**S1**	How old are you/is your child?<br>***(Verify that the age is the same age as written on the household listing)***	[ ] Years		
**S2**	Have you/ your child ever attended school?	No…………………………<br>Yes ………………………	00<br>01	**00** → S4
**S3**	What is the highest level of school (name of child) completed?	None………………………..0<br>Primary ……………………..1		

PART II: CHILD BIOCHEMICAL MEASUREMENT

Verbal consent given for: **SL01** Blood [ ] **SL02** Urine [ ] **SL03** Stool [ ]<br>0= No OR 1= yes	
**SL04** Phlebotomist Code [ ][ ]	
**SL5** BLUE TOP TUBE (METAL FREE)<br>**Did not work =00.0**<br>**Refused = 77.7**	ML. [ ][ ] • [ ]
**SL6** PURPLE TOP TUBE (EDTA)<br>**Did not work =00.0**<br>**Refused = 77.7**	ML. [ ][ ] • [ ]
**SL7** REDTOP TUBE (EDTA)<br>**Did not work =00.0**<br>**Refused = 77.7**	ML. [ ][ ] • [ ]
**SL8** DATE BLOOD SAMPLE TAKEN (Ethiopian calendar)	Date: ______/______/__________<br>Day / Month / Year
**SL9** TIME BLOOD DRAW (Ethiopian time)	Blood draw ___ ___ : ___ ___<br>Hour Minute
**SL10 When did you eat your most recent meal (food)?** (Ethiopian time)	Last Meal Eaten ___ ___ : ___ ___<br>Hour Minute
**SL11** FEVER in last 24 HR? (Since same time yesterday)	No……………………………………………………….00<br>Yes …………………………………………………….01
**SL12** MALARIA RESULTS (RDK)	NEGATIVE……………………………..00<br>POSITIVE P *falciparum* ……………..01<br>POSITIVE P *vivax* ……………………..02<br>INVALID ……………………………………03

For peer review only - http://bmjopen.bmj.com/site/about/guidelines.xhtml

**SL13** HEMOGLOBIN RESULTS	g/dL [ ][ ] . [ ]
**SL14** Is that finger prick or venous sample taken?	Finger prick……………………………..00<br>Venous ………………………………….01
**In order to determine if you have blood in urine or worms in stool we would like to collect a urine and stool sample. If you can provide this now, we appreciate it. If not now, we can come back to pick up the sample at a later time.**	
**SL15 U**rine collected?	No……………………………………………….00<br>yes ……………………………………………..01
**SL16** Blood in urine RESULTS	Negative……………………………………….00<br>positive …………………………………………..01
**SL17** Stool collected?	No………………………………………………..00<br>yes……………………………………………….01
**SL18** Date and **t**ime when stool passed by the respondent (as recorded on cup) (Ethiopian time)	Date: __/_____/______ and ___ ___ : ___ ___<br>Day / Month /Year Hour Minute
**SL19** Date stool sample taken (Ethiopian calendar)	Date: ______/_______/__________<br>Day / Month / Year
**SL20** Time when stool collected from the respondent (Ethiopian time)	___ ___ : ___ ___<br>Hour Minute
**SL21** TIME BLOOD centrifuged (Ethiopian time)	___ ___ : ___ ___<br>Hour Minute

Thank the respondent and tell them that the lab team will be arriving later.

INTERVIEWER'S OBSERVATIONS
TO BE FILLED IN AFTER COMPLETING INTERVIEW

COMMENTS:

____

____

For peer review only - http://bmjopen.bmj.com/site/about/guidelines.xhtml

ADELESCENT GIRLS (10-19 YEARS)
ETHIOPIAN FOOD AND NUTRITION STRATEGY BASELINE SURVEY 2020/21
Biochemical and Health Related Data Collection Tool

IDENTIFICATION	
**HH00.** CLUSTER (EA) NAME………………………………	**HH01**. CLUSTER NUMBER: ☐☐☐
**HH02**. HH NUMBER: ☐☐	**HH03**. RESIDENCE (RURAL=1, URBAN=2): ☐☐
**HH04**. RESPONDENT LINE NUMBER: (SHOULD BE MOTHER/CAREGIVER) ☐☐	**HH05** WOMEN LINE NUMBER ☐☐
**HH06**. INTERVIEWER NAME__________________ CODE:____________________	**HH07**. TEAM LEADER, NAME:__________________ CODE:_____________________
**HH08.** SUPERVISOR NAME:___________________ CODE._____________________	

PART I: HEALTH RELATED QUESTIONS

I would like to ask you some health and food questions about yourself. Fill or Circle the correct answer

No.	**Question**	**Coding categories**	**Skip**
1	How old are you?<br>(*verify that the age is the same age as written on the household listing*)	☐ Years	
2	Have you been diagnosed with anemia in the past six months?	No……………………………………….0<br>Yes ………………………………….1<br>Don't know……………………….98	
3	Do you smoke? (do not include the powder and chew type)	No……………………….0<br>Yes ……………………..1	

PART II: ADOLESCENT BIOCHEMICAL MEASUREMENT

Consent given for:<br>0= No or 1= Yes	AG**01** Blood ☐	AG**L02** Stool ☐
**AG03** BLUE TOP TUBE (METAL FREE)<br>**Did not work =00.0**	ML. ☐☐ • ☐	

For peer review only - http://bmjopen.bmj.com/site/about/guidelines.xhtml

**Refused = 77.7**	
**AG04** PURPLE TOP TUBE (EDTA)<br>**Did not work =00.0**<br>**Refused = 77.7**	ML. __ __ • __
**AG05** REDTOP TUBE (EDTA)<br>**Did not work =00.0**<br>**Refused = 77.7**	ML. __ __ • __
**AG06** Date blood sample taken (Ethiopian calendar)	Date: ______/_______/___________<br>Day / Month / Year
**AG07** TIME BLOOD DRAW (Ethiopian time)	Blood draw ___ ___ : ___ ___<br>Hour Minute
**AG08 When did you eat your most recent meal (food)?** (Ethiopian date and time)	______/_______/___________ __ ___ : ___ ___<br>Date /Month/ Year Hour Minute
**AG09 Is it Finger prick or venous blood sample taken?**	01 Finger prick<br>02 Venous
**AG09** MALARIA RESULTS (RDT)	NEGATIVE....................................... 00<br>POSITIVE P *falciparum* .... 01<br>POSITIVE P *vivax* .... 02<br>POSITIVE FOR BOTH P *falciparum and* P *vivax ...... 03*<br>INVALID 04
**AG10 HEMOGLOBIN** RESULTS	g/dL __ __ • __
**In order to determine if you have worms in the stool we would like to collect a stool sample. If you can provide this now, we appreciate it. If not now, we can come back to pick up the sample at a later time.**<br>*INSTRUCTIONS IF UNABLE TO PRODUCE AT WILL*:<br>**For stool**: We will return tomorrow to pick up your stool. We would like the fresh stool you can give us. Please use one cup to collect the first stool you pass.	
**AG11** Stool collected?	No...00<br>yes 01
**AG12** Date stool sample taken (Ethiopian calendar)	Date: ______/_______/___________<br>Day / Month / Year
**AG13** Time when stool passed by the respondent (as recorded on cup) (Ethiopian time)	___ ___ : ___ ___<br>Hour Minute
**AG14** Time when stool collected from the respondent (Ethiopian time)	___ ___ : ___ ___

For peer review only - http://bmjopen.bmj.com/site/about/guidelines.xhtml

	Hour Minute
**AG15** TIME BLOOD centrifuged (Ethiopian time)	___ ___ : ___ ___<br>Hour Minute

OBSERVATIONS

TO BE FILLED IN AFTER COMPLETING INTERVIEW

COMMENTS:

____
____
____

WOMEN OF REPRODUCTIVE AGE 15-49 YEAR OLDS
ETHIOPIAN FOOD AND NUTRITION STRATEGY BASELINE SURVEY 2020/21
Biochemical and Health Related Data Collection Tool

IDENTIFICATION	
**HH00.** CLUSTER (EA) NAME................................	**HH01**. CLUSTER NUMBER:
**HH02**. HH NUMBER:	**HH03**. RESIDENCE (RURAL=1, URBAN=2):
**HH04**. RESPONDENT LINE NUMBER: (SHOULD BE MOTHER/CAREGIVER)	**HH05** WOMEN LINE NUMBER
HH06. INTERVIEWER NAME___________________ CODE:____________________	HH07. TEAM LEADER, NAME:______________ CODE:_____________________
HH08. SUPERVISOR NAME:____________________ CODE._____________________	

PART I: HEALTH RELATED QUESTIONS

S.N	QUESTION	Response	SKIP
1	How old are you?<br>(*verify that the age is the same age as written on the household listing*)	Years	
Now I would like to ask you some questions about your health. I will first ask you about the last 6 months.			
2	Have you been diagnosed with anemia in the past six months?	No...........................0	

For peer review only - http://bmjopen.bmj.com/site/about/guidelines.xhtml

		Yes ....1<br>Don't know...........98	
2	Have you been ill with malaria in the past 2 weeks?	No............................0<br><br>Yes ....1<br>Don't know...........98	
3	Do you smoke? (do not include the powder and chew type)	No...................0<br><br>Yes 1	
4	Are you currently lactating?	No...................0<br><br>Yes 1	
5	During that last pregnancy (that resulted in a live birth) did you have difficulty with your vision at night ("Dafent" night blindness in local language)?	No............................0<br><br>Yes ....1<br>Don't know............98	
6	In the first two months after delivery, did you receive a<br><br>vitamin A dose (like this)?<br><br>*SHOW THE CAPSULE*	No............................0<br><br>Yes ....1<br>Don't know...........98	

PART II: WOMEN BIOCHEMICAL MEASUREMENT

**If the women is pregnant do not collect venous blood**	
Consent given for:<br>0= No or 1= Yes	**WL01** Blood ☐ **WL02** Urine ☐ **WL03** Stool ☐
**WL4** BLUE TOP TUBE (METAL FREE)<br>**Did not work =00.0**<br>**Refused = 77.7**<br>**Pregnant = 99.9**	ML. ☐☐ • ☐
**WL5** PURPLE TOP TUBE (EDTA)<br>**Did not work =00.0**<br>**Refused = 77.7**<br>**Pregnant = 99.9**	ML. ☐☐ • ☐
**WL6** REDTOP TUBE (EDTA)<br>**Did not work =00.0**<br>**Refused = 77.7**<br>**Pregnant = 99.9**	ML. ☐☐ • ☐
**WL7** Date blood sample taken (Ethiopian calendar)	Date: ______/_______/__________

For peer review only - http://bmjopen.bmj.com/site/about/guidelines.xhtml

	Day / Month / Year
**WL8** TIME BLOOD DRAW (Ethiopian time)	Blood draw ___ ___ : ___ ___<br>Hour Minute
**WL9 When did you eat your most recent meal (food)?** (Ethiopian date and time)	______/______/__________ __ ___ : ___ ___<br>Date /Month/ Year Hour Minute
**WL 10 Finger prick or venous sample taken?**	01 Finger prick<br>02 Venous
**WL11** MALARIA RESULTS (RDT)	NEGATIVE.. 00<br>POSITIVE P *falciparum* .... 01<br>POSITIVE P *vivax* .... 02<br>POSITIVE FOR BOTH P *falciparum and* P *vivax* .... *03*<br>INVALID 04
**WL12** HEMOGLOBIN RESULTS	g/dL [ ][ ] . [ ]
**In order to determine if you have blood in the urine or worms in the stool we would like to collect a urine and stool sample. If you can provide this now, we appreciate it. If not now, we can come back to pick up the sample at a later time.**<br>*INSTRUCTIONS IF UNABLE TO PRODUCE AT WILL*:<br>**For stool**: We will return tomorrow to pick up your stool. We would like the fresh stool you can give us. Please use one cup to collect the first stool you pass.<br>**For urine**: We will return tomorrow to pick up your urine.	
**WL13 U**rine collected?	No..0<br>0 yes<br>..01
**WL14** RESULTS (blood in urine)<br>**Ask the women if she is Menstruating**<br>(Don't test if the women is in Menstruation)	Negative...00<br>positive ....01<br>Women is Menstruating..............................03
**WL15** Stool collected?	No...00<br>yes 01
**WL16** Date stool sample taken (Ethiopian calendar)	Date: ______/______/__________<br>Day / Month / Year
**WL17** Time when stool passed by the respondent (as recorded on cup) (Ethiopian time)	___ ___ : ___ ___<br>Hour Minute
**WL18** Time when stool collected from the respondent (Ethiopian time)	___ ___ : ___ ___

For peer review only - http://bmjopen.bmj.com/site/about/guidelines.xhtml

	Hour Minute
**WL19** TIME BLOOD centrifuged (Ethiopian time)	___ ___ : ___ ___<br>Hour Minute

OBSERVATIONS

TO BE FILLED IN AFTER COMPLETING INTERVIEW

COMMENTS:

____
____
____
____
____

For peer review only

For peer review only - http://bmjopen.bmj.com/site/about/guidelines.xhtml

Supplementary consent 1

1. **Informed Consent Form for Household Head**

Ethiopian Food and Nutrition Strategy (FNS) Baseline Survey

Investigator(s): Dr.Masresha Tessema (PI), Meseret W/Yohannes, Dr. Meron Girma, Alemnesh Petros, Dr Aregash Samuel, Arnaud Laillou, Stanley Chitekwe, Kaleab Baye, Ramadhani Noor, Anne Sophie Donze and other co-authors

You are being invited to take part in this research because you are head of household. There are about [16596] households taking part in this research. We will ask you about household characteristics, and socio-economic status of your household. We will also assess dietary intake, anthropometric status, nutrition sensitive and specific indicators and micronutrient status of your household member.

Box 1. Taking part in this research is voluntary

☐You **can refuse** to take part in this study.

☐You **can withdraw** your participation from the study at any time

Information related to the study

The FNS baseline survey will be conducted in the 12 regions of Ethiopia. The study population will be children age 0-59 months having caregivers/mothers, school-age children (6-12 years), adolescent girls (10-19 years), reproductive-age women (15-49 years), pregnant and lactating women, and household head. The indicators that will be collected for the survey will be dietary intake, anthropometric status, nutrition sensitive and specific indicators and micronutrient status.

The expected possible adverse effects: there is no adverse effect by participating in this study

The objective of this research: to produce information on anthropometric status, dietary intakes, and micronutrient status of different population groups in Ethiopia, and assess the coverage of direct and indirect nutrition interventions.

**Study design:** A population-based, cross-sectional design	
**The schedule of the study:** The study will be conducted from July, 2021 to April, 2023	
**Foreseeable risks and expected benefits arising from participation in the study**	
**Foreseeable risks**	**Expected benefits**
Risks to study participants for involvement in the coverage survey are low. There may be risks associated	The findings of the study will help the ministry of health and other stakeholders engaged in nutrition

For peer review only

with COVID pandemic. Interviewers will be trained to minimize this risk and will use appropriate prevention measures.	to improve and/or design appropriate health and nutrition intervention programs in the country.

Occurrences that may take place during the study period

**Occurrences**	**How to manage**
Withdrawal of volunteers from the study	In such a case, we would respect the volunteer's decision to withdraw and also get a clear understanding of the reason for their withdrawal

At the end of the study, you will not be receiving any financial benefits, but will get your results for height, weight, mid upper arm and waist circumference measurements, anemia and goiter status for time you spent and participation.

All data collected from the study will be kept confidential. If you have any questions related to the study you may contact directly Dr. Masresha Tessema who is the project PI.

The contact persons

1. Dr. Masresha Tessema Tel. [+251 919782082] E-mail: [masresha88@gmail.com] or
2. [Mr. Ibrahim Kedir] Tel. [+251 911957161] EPHI's IRB

Certificate of Consent

I have read the foregoing information. I have an opportunity to ask questions and all my quest have been answered to my satisfaction. I volunteer to give consent to participate in this research study	I confirm that the participant was given an opportunity to ask questions about the study and all questions have been answered correctly. I confirm that the consent has been given voluntarily
__________________________<br>Printed name of the participant<br>_________________________<br>Signature of the participant<br>Date _________________<br>day/month/year	_________________________________<br>Printed name of the person taking the consent<br>________________________<br>Signature of the person taking the consent<br>Date_____________________<br>day/month/year

For peer review only

2. Informed Consent Form for Women of Reproductive Age

Ethiopian Food and Nutrition Strategy (FNS) Baseline Survey

Investigator(s): Dr.Masresha Tessema (PI), Meseret W/Yohannes, Dr. Meron Girma, Alemnesh Petros, Dr Aregash Samuel, Arnaud Laillou, Stanley Chitekwe, Kaleab Baye, Ramadhani Noor, Anne Sophie Donze and other co-authors

You are being invited to take part in this research because you are women of reproductive age. There are [16596] households taking part in this research. We will assess your dietary intake, anthropometric status, nutrition sensitive and specific indicators and micronutrient status

Box 1. Taking part in this research is voluntary

☐You **can refuse** to take part in this study.

☐You **can withdraw** your participation from the study at any time

Information related to the study

The FNS baseline survey will be conducted in the 12 regions of Ethiopia. The study population will be children age 0-59 months having caregivers/mothers, school-age children (6-12 years), adolescent girls (10-19 years), reproductive-age women (15-49 years), pregnant and lactating women, and household head. The indicators that will be collected for the survey will be dietary intake, anthropometric status, nutrition sensitive and specific indicators and micronutrient status.

The expected possible adverse effects: there is no adverse effect by participating in this study

The objective of this research: to produce information on anthropometric status, dietary intakes, and micronutrient status of different population groups in Ethiopia, and assess the coverage of direct and indirect nutrition interventions.

Study design: A population-based, cross-sectional design

The schedule of the study: The study will be conducted from July, 2021 to April, 2023

Foreseeable risks and expected benefits arising from participation in the study

Foreseeable risks	Expected benefits
Risks to study participants for involvement in the coverage survey are low. There may be risks associated with COVID pandemic. Interviewers will be trained to minimize this risk and will use appropriate prevention measures.	The findings of the study will help the ministry of health and other stakeholders engaged in nutrition to improve and/or design appropriate health and nutrition intervention programs in the country.

For peer review only

Occurrences that may take place during the study period	
**Occurrences**	**How to manage**
Withdrawal of volunteers from the study	In such a case, we would respect the volunteer's decision to withdraw and also get a clear understanding of the reason for their withdrawal

At the end of the study, you will not be receiving any financial benefits, but will get your results for height, weight, mid upper arm and waist circumference measurements, anemia and goiter status for time you spent and participation.

All data collected from the study will be kept confidential. If you have any questions related to the study you may contact directly Dr. Masresha Tessema who is the project PI.

The contact persons

1. Dr. Masresha Tessema Tel. [+251 919782082] E-mail: [masresha88@gmail.com] or
2. [Mr. Ibrahim Kedir] Tel. [+251 911957161] EPHI's IRB

**Certificate of Consent**	
I have read the foregoing information. I have an opportunity to ask questions and all my quest have been answered to my satisfaction. I volunteer to give consent to participate in this research study	I confirm that the participant was given an opportunity to ask questions about the study and all questions have been answered correctly. I confirm that the consent has been given voluntarily
________________________<br>Printed name of the participant<br>_______________________<br>Signature of the participant<br>Date ________________<br>day/month/year	______________________________<br>Printed name of the person taking the consent<br>______________________<br>Signature of the person taking the consent<br>Date___________________<br>day/month/year

For peer review only

3. Informed Consent Form for Pregnant Women

**Ethiopian Food and Nutrition Strategy (FNS) Baseline Survey**
**Investigator(s):** Dr.Masresha Tessema (PI), Meseret W/Yohannes, Dr. Meron Girma, Alemnesh Petros, Dr Aregash Samuel, Arnaud Laillou, Stanley Chitekwe, Kaleab Baye, Ramadhani Noor, Anne Sophie Donze and other co-authors

You are being invited to take part in this research because you are pregnant women. There are [16596] households taking part in this research. We will assess your, anthropometric status, nutrition sensitive and specific indicators and anemia status

**Box 1. Taking part in this research is voluntary**
☐You **can refuse** to take part in this study.
☐You **can withdraw** your participation from the study at any time

Information related to the study

The FNS baseline survey will be conducted in the 12 regions of Ethiopia. The study population will be children age 0-59 months having caregivers/mothers, school-age children (6-12 years), adolescent girls (10-19 years), reproductive-age women (15-49 years), pregnant and lactating women, and household head. The indicators that will be collected for the survey will be dietary intake, anthropometric status, nutrition sensitive and specific indicators and micronutrient status.

**The expected possible adverse effects:** There is no adverse effect by participating in this study

The objective of this research: to produce information on anthropometric status, dietary intakes, and micronutrient status of different population groups in Ethiopia, and assess the coverage of direct and indirect nutrition interventions.

**Study design:** a population-based, cross-sectional design
**The schedule of the study:** The study will be conducted from July, 2021 to April, 2023

**Foreseeable risks and expected benefits arising from participation in the study**	
**Foreseeable risks**	**Expected benefits**
Risks to study participants for involvement in the coverage survey are low. There may be risks associated with COVID pandemic. Interviewers will be trained to minimize this risk and will use appropriate prevention measures.	The findings of the study will help the ministry of health and other stakeholder engaged in nutrition to improve and/or design appropriate health and nutrition intervention programs in the country.
**Occurrences that may take place during the study period**	

For peer review only

Occurrences	How to manage
Withdrawal of volunteers from the study	In such a case, we would respect the volunteer's decision to withdraw and also get a clear understanding of the reason for their withdrawal

At the end of the study, you will not be receiving any financial benefits, but will get your results for height, weight, mid upper arm circumference measurements, anemia and goiter status for time you spent and participation.

All data collected from the study will be kept confidential. If you have any questions related to the study you may contact directly Dr. Masresha Tessema who is the PI

The contact persons

1. Dr. Masresha Tessema

Tel. [+251 919782082] E-mail: [masresha88@gmail.com]

2. [Mr. Ibrahim Kedir] Tel. [+251 911957161]

**Certificate of Consent**	
I have read the foregoing information. I have an opportunity to ask questions and all my quest have been answered to my satisfaction. I volunteer to give consent to participate in this research study<br><br>________________________<br>Printed name of the participant<br>_______________________<br>Signature of the participant<br>Date _______________<br>day/month/year	I confirm that the participant was given an opportunity to ask questions about the study and all questions have been answered correctly. I confirm that the consent has been given voluntarily<br><br>______________________________<br>Printed name of the person taking the consent<br>______________________<br>Signature of the person taking the consent Date<br>___________________<br>day/month/year

4. **Informed Consent Form for Preschool Child**

Ethiopian Food and Nutrition Strategy (FNS) Baseline Survey

Investigator(s): Dr.Masresha Tessema (PI), Meseret W/Yohannes, Dr. Meron Girma, Alemnesh Petros, Dr

For peer review only

Aregash Samuel, Arnaud Laillou, Stanley Chitekwe, Kaleab Baye, Ramadhani Noor, Anne Sophie Donze and other co-authors

You are being invited to take part in this research because you are either a mother or caregiver who has a child under the age of 5 years (0-59 months). There are [16596] households taking part in this research. We would collect a sample of your child's dietary information, blood, urine and stool. And, we will also measure your child's height/ length, weight, and mid upper arm circumference. Finally we will assess, your child's eye for bitot spot

Box 1. Taking part in this research is voluntary

- You **can refuse** to take part in this study.
- You **can withdraw** your participation from the study at any time

Information related to the study

The FNS baseline survey will be conducted in the 12 regions of Ethiopia. The study population will be children age 0-59 months having caregivers/mothers, school-age children 6-12 years), adolescent girls (10-19 years), reproductive-age women (15-49 years), pregnant and lactating women, and household head. The indicators that will be collected for the survey will be dietary intake, anthropometric status, nutrition sensitive and specific indicators and micronutrient status.

The expected possible adverse effects :there is no adverse effect by participating in this study

The objective of this research: to produce information on anthropometric status, dietary intakes, and micronutrient status of different population groups in Ethiopia, and assess the coverage of direct and indirect nutrition interventions.

**Study design:** a population-based, cross-sectional design	
**The schedule of the study:** The study will be conducted from July, 2021 to April, 2023	
**Foreseeable risks and expected benefits arising from participation in the study**	
**Foreseeable risks**	**Expected benefits**
Risks to study participants for involvement in the coverage survey are low. There may be risks associated with COVID pandemic. Interviewers will be trained to minimize this risk and will use appropriate prevention measures.	The findings of the study will help the ministry of health and other stakeholder engaged in nutrition to improve and/or design appropriate health and nutrition intervention programs in the country.
**Occurrences that may take place during the study period**	
**Occurrences**	**How to manage**

For peer review only

Withdrawal of volunteers from the study	in such a case, we would respect the volunteer's decision to withdraw and also get a clear understanding of the reason for their withdrawal

At the end of the study, you will not be receiving any financial benefits, but will get your results for height/length, weight, mid upper arm circumference measurements, and anemia for time you spent and participation.

All data collected from the study will be kept confidential. If you have any questions related to the study you may contact directly Dr. Masresha Tessema who is the project principal investigator

The contact persons

1. Dr. Masresha Tessema

Tel. [+251 919782082] E-mail: [masresha88@gmail.com]

2. [Mr. Ibrahim Kedir] Tel. [+251 911957161] EPHI's IRB

**Certificate of Consent**	
I have read the foregoing information. I have an opportunity to ask questions and all my quest have been answered to my satisfaction. I volunteer to give consent to participate in this research study<br><br>________________________<br>Printed name of the participant<br>________________________<br>Signature of the participant's parent or guardian<br>Date ________________<br>day/month/year	I confirm that the participant was given an opportunity to ask questions about the study and all questions have been answered correctly. I confirm that the consent has been given voluntarily<br><br>______________________________<br>Printed name of the person taking the consent<br>______________________<br>Signature of the person taking the consent Date<br>___________________<br>day/month/year

For peer review only

5. **Informed Consent Form for School Age Children**

Ethiopian Food and Nutrition Strategy (FNS) Baseline Survey

Investigator(s): Dr.Masresha Tessema (PI), Meseret W/Yohannes, Dr. Meron Girma, Alemnesh Petros, Dr Aregash Samuel, Arnaud Laillou, Stanley Chitekwe, Kaleab Baye, Ramadhani Noor, Anne Sophie Donze and other co-authors

You are being invited to take part in this research because you are either a mother or caregiver who has a school-age child. Among children 6 – 12 years, we will collect your child's blood, urine and stool.

Box 1. Taking part in this research is voluntary

☐You **can refuse** to take part in this study.

☐You **can withdraw** your participation from the study at any time

Information related to the study

The FNS baseline survey will be conducted in the 12 regions of Ethiopia. The study population will be children age 0-59 months having caregivers/mothers, school-age children (6-12 years), adolescent girls (10-19 years), reproductive-age women (15-49 years), pregnant and lactating women, household head. The indicators that will be collected for the survey will be dietary intake, anthropometric status, nutrition sensitive and specific indicators and micronutrient status.

The expected possible adverse effects: there is no adverse effect by participating in this study

The objective of this research: to produce information on anthropometric status, dietary intakes, and micronutrient status of different population groups in Ethiopia, and assess the coverage of direct and indirect nutrition interventions.

Study design: a population-based, cross-sectional design

The schedule of the study: The study will be conducted from July, 2021 to April, 2023

**Foreseeable risks and expected benefits arising from participation in the study**	
**Foreseeable risks**	**Expected benefits**
Risks to study participants for involvement in the coverage survey are low. There may be risks associated with COVID pandemic. Interviewers will be trained to minimize this risk and will use appropriate prevention measures.	The findings of the study will help the ministry of health and other stakeholder engaged in nutrition to improve and/or design appropriate health and nutrition intervention programs in the country.

**Occurrences that may take place during the study period**	
**Occurrences**	**How to manage**

For peer review only

Withdrawal of volunteers from the study	In such a case, we would respect the volunteer's decision to withdraw and also get a clear understanding of the reason for their withdrawal

At the end of the study, you will not be receiving any financial benefits, but you will get your **anemia** and **goiter** status for time you spent and participation.

All data collected from the study will be kept confidential. If you have any questions related to the study you may contact directly Dr. Masresha Tessema who is the project's PI

The contact persons

1. Dr. Masresha Tessema

Tel. [+251 919782082] E-mail: [masresha88@gmail.com]

2. [Mr. Ibrahim Kedir] Tel. [+251 911957161] EPHI's IRB

**Certificate of Consent**	
I have read the foregoing information. I have an opportunity to ask questions and all my quest have been answered to my satisfaction. I volunteer to give consent to participate in this research study<br><br>________________________<br>Printed name of the participant<br><br>_______________________<br>Signature of the participant's parent or guardian<br><br>Date _______________<br>day/month/year	I confirm that the participant was given an opportunity to ask questions about the study and all questions have been answered correctly. I confirm that the consent has been given voluntarily<br><br>______________________________<br>Printed name of the person taking the consent<br><br>______________________<br>Signature of the person taking the consent Date<br><br>___________________<br>day/month/year

For peer review only

6. Assent form for Adolescent Girls (10-19 years)

**Ethiopian Food and Nutrition Strategy (FNS) Baseline Survey**
**Investigator(s):** Dr.Masresha Tessema (PI), Meseret W/Yohannes, Dr. Meron Girma, Alemnesh Petros, Dr Aregash Samuel, Arnaud Laillou, Stanley Chitekwe, Kaleab Baye, Ramadhani Noor, Anne Sophie Donze and other co-authors

You are being invited to take part in this research because you are Adolescent girl. There are [16596] households taking part in this research. We will measure your dietary information (for those adolescent girls aged 15-17 years), information related to nutrition -sensitive and nutrition-specific practices, blood, and stool, we will also measure your height, weight, and mid upper arm and waist circumference and your goiter status

**Box 1. Taking part in this research is voluntary**
☐You **can refuse** to take part in this study.
☐You **can withdraw** your participation from the study at any time

Information related to the study

The FNS baseline survey will be conducted in the 12 regions of Ethiopia. The study population will be children age 0-59 months having caregivers/mothers, school-age children (6-12 years), adolescent girls (10-19 years), reproductive-age women (15-49 years), pregnant and lactating women, and household head. The indicators that will be collected for the survey will be dietary intake, anthropometric status, nutrition sensitive and specific indicators and micronutrient status

**The expected possible adverse effects:** There is no adverse effect by participating in this study

The objective of this research: to produce information on anthropometric status, dietary intakes, and micronutrient status of different population groups in Ethiopia, and assess the coverage of direct and indirect nutrition interventions.

**Study design:** a population-based, cross-sectional design	
**The schedule of the study:** The study will be conducted from July, 2021 to April, 2023	
**Foreseeable risks and expected benefits arising from participation in the study**	
**Foreseeable risks**	**Expected benefits**
Risks to study participants for involvement in the coverage survey are low. There may be risks associated with COVID pandemic. Interviewers will be trained to minimize this risk and will use appropriate prevention measures.	The findings of the study will help the ministry of health and other stakeholder engaged in nutrition to improve and/or design appropriate health and nutrition intervention programs in the country.

Occurrences that may take place during the study period	
**Occurrences**	**How to manage**
Withdrawal of volunteers from the study	In such a case, we would respect the volunteer's decision to withdraw and also get a clear understanding of the reason for their withdrawal

At the end of the study, you will not be receiving any financial benefits, but will get your results for height, weight, mid upper arm and waist circumference measurements, anemia and goiter status for time you spent and participation.

All data collected from the study will be kept confidential. If you have any questions related to the study you may contact directly Dr. Masresha Tessema who is the project's PI

The contact persons

1. Dr. Masresha Tessema

Tel. [+251 919782082] E-mail: [masresha88@gmail.com]

2. [Mr. Ibrahim Kedir] Tel. [+251 911957161] EPHI's IRB

Certificate of Assent	
I have read the foregoing information. I have an opportunity to ask questions and all my quest have been answered to my satisfaction. I volunteer to give assent to participate in this research study<br><br>_________________________<br>Printed name of the participant<br><br>________________________<br>Signature of the participant<br>Date ________________<br>day/month/year	I confirm that the participant was given an opportunity to ask questions about the study and all questions have been answered correctly. I confirm that the assent has been given voluntarily<br><br>_______________________________<br>Printed name of the person taking the assent<br>______________________<br>Signature of the person taking the assent Date<br>___________________<br>day/month/year

For peer review only

STROBE Statement—checklist of items that should be included in reports of observational studies

	Item No	Recommendation	Reported on page #
**Title and abstract**	1	(*a*) Indicate the study's design with a commonly used term in the title or the abstract	Title p.1; Abstract p.2-3
		(*b*) Provide in the abstract an informative and balanced summary of what was done and what was found	
**Introduction**			
**Introduction** /rationale	4	Explain the scientific background and rationale for the investigation being reported	**Introduction** p.3-4
Objectives	5	State specific objectives, including any prespecified hypotheses	Background p.5 last statements
**Methods**			
Study design	5	Present key elements of study design early in the paper	Methods (data source) p.4
Setting	5	Describe the setting, locations, and relevant dates, including periods of recruitment, exposure, follow-up, and data collection	Methods (data source) p.5
Participants	5	(*a*) *Cohort study*—Give the eligibility criteria, and the sources and methods of selection of participants. Describe methods of follow-up *Case-control study*—Give the eligibility criteria, and the sources and methods of case ascertainment and control selection. Give the rationale for the choice of cases and controls *Cross-sectional study*—Give the eligibility criteria, and the sources and methods of selection of participants	Methods (participants) p.5-8
		(*b*) *Cohort study*—For matched studies, give matching criteria and number of exposed and unexposed *Case-control study*—For matched studies, give matching criteria and the number of controls per case	
Variables	5	Clearly define all outcomes, exposures, predictors, potential confounders, and effect modifiers. Give diagnostic criteria, if applicable	Methods (all outcome and exposure variables are listed) p.6-8
Data sources/ measurement	NA	For each variable of interest, give sources of data and details of methods of assessment (measurement). Describe comparability of assessment methods if there is more than one group	Methods (methods of measurement indicated in) p.5-8
Bias	10	Describe any efforts to address potential sources of bias	Methods (data quality indicated) p.10-11
Study size	5-6	Explain how the study size was arrived at	Methods (data source,statistical

For peer review only

For peer review only - http://bmjopen.bmj.com/site/about/guidelines.xhtml

			analysis) p.5
Quantitative variables	10	Explain how quantitative variables were handled in the analyses. If applicable, describe which groupings were chosen and why	Methods (measurement and statistical analysis sections) p. 8-9
Statistical methods	10	(*a*) Describe all statistical methods, including those used to control for confounding	Methods (analysis section) p. 8-9
		(*b*) Describe any methods used to examine subgroups and interactions	
		(*c*) Explain how missing data were addressed	
		(*d*) *Cohort study*—If applicable, explain how loss to follow-up was addressed<br>*Case-control study*—If applicable, explain how matching of cases and controls was addressed<br>*Cross-sectional study*—If applicable, describe analytical methods taking account of sampling strategy	
		(*e*) Describe any sensitivity analyses	

Continued on next page

For peer review only

**Results**			
Participants	5*	(a) Report numbers of individuals at each stage of study—eg numbers potentially eligible, examined for eligibility, confirmed eligible, included in the study, completing follow-up, and analysed	NA
		(b) Give reasons for non-participation at each stage	
		(c) Consider use of a flow diagram	
Descriptive data	14*	(a) Give characteristics of study participants (eg demographic, clinical, social) and information on exposures and potential confounders	NA
		(b) Indicate number of participants with missing data for each variable of interest	
		(c) *Cohort study*—Summarise follow-up time (eg, average and total amount)	
Outcome data	15*	*Cohort study*—Report numbers of outcome events or summary measures over time	NA
		Case-control study—Report numbers in each exposure category, or summary measures of exposure	
		Cross-sectional study—Report numbers of outcome events or summary measures	
Main results	16	(*a*) Give unadjusted estimates and, if applicable, confounder-adjusted estimates and their precision (eg, 95% confidence interval). Make clear which confounders were adjusted for and why they were included	NA
		(*b*) Report category boundaries when continuous variables were categorized	
		(*c*) If relevant, consider translating estimates of relative risk into absolute risk for a meaningful time period	
Other analyses	17	Report other analyses done—eg analyses of subgroups and interactions, and sensitivity analyses	NA
**Discussion**			
Key results	18	Summarise key results with reference to study objectives	p. 9-10
Limitations	19	Discuss limitations of the study, taking into account sources of potential bias or imprecision. Discuss both direction and magnitude of any potential bias	Discussion (strengths and weaknesses of the study) p.10-11
Interpretation	20	Give a cautious overall interpretation of results considering objectives, limitations, multiplicity of analyses, results from similar studies, and other relevant evidence	Discussion (interpretation of findings in the context of existing research, meaning of the study:

For peer review only - http://bmjopen.bmj.com/site/about/guidelines.xhtml

			p.9-11 implication
Generalisability	21	Discuss the generalisability (external validity) of the study results	Discussion (strengths and weakness of the study) p.10-11
**Other information**			
Funding	22	Give the source of funding and the role of the funders for the present study and, if applicable, for the original study on which the present article is based	Funding p.12

*Give information separately for cases and controls in case-control studies and, if applicable, for exposed and unexposed groups in cohort and cross-sectional studies.

Note: An Explanation and Elaboration article discusses each checklist item and gives methodological background and published examples of transparent reporting. The STROBE checklist is best used in conjunction with this article (freely available on the Web sites of PLoS Medicine at http://www.plosmedicine.org/, Annals of Internal Medicine at http://www.annals.org/, and Epidemiology at http://www.epidem.com/). Information on the STROBE Initiative is available at www.strobe-statement.org.

For peer review only

BMJ Open

Ethiopia National Food and Nutrition Survey to inform the Ethiopian National Food and Nutrition Strategy: a study protocol

Journal:	*BMJ Open*
Manuscript ID	bmjopen-2022-067641.R2
Article Type:	Protocol
Date Submitted by the Author:	28-Mar-2023
Complete List of Authors:	Woldeyohannes, Meseret ; Ethiopian Public Health Institute<br>Girma, Meron; Ethiopian Public Health Institute, National Information Platforms for Nutrition<br>Petros, Alemnesh; Ethiopian Public Health Institute<br>Hussen, Alemayehu ; Ethiopian Public Health Institute<br>Samuel, Aregash; Ethiopian Public Health Institute<br>Dinssa, Danial ; Ethiopian Public Health Institute, Environmental and Public Health Research<br>Challa, Feyissa; Ethiopian Public Health Institute<br>Laillou, Arnaud; UNICEF, Dakar, Senegal<br>Chitekwe, Stanley; UNICEF Ethiopia<br>Baye, Kaleab ; Addis Ababa University,<br>Noor , Ramadhani; UNICEF Ethiopia<br>Donze, Anne Sophie; UNICEF Ethiopia<br>Tollera, Getachew; Ethiopian Public Health Institute<br>Dangiso, Mesay; Ethiopian Public Health Institute<br>Tadesse, Lia; Ethiopia Ministry of Health<br>Zelalem, Meseret; Ethiopia Ministry of Health<br>Tessema, Masresha; Ethiopian Public Health Institute,
**Primary Subject Heading**:	Public health
Secondary Subject Heading:	Epidemiology
Keywords:	EPIDEMIOLOGY, NUTRITION & DIETETICS, PUBLIC HEALTH

SCHOLARONE™
Manuscripts

Ethiopia National Food and Nutrition Survey to inform the Ethiopian National Food and Nutrition Strategy: a study protocol

Meseret Woldeyohannes[1], Meron Girma[1], Alemnesh Petros[1], Alemayehu Hussen[1], Aregash Samuel[1], Danial Abera Dinssa[1], Feyissa Challa[1], Arnaud Laillou[2], Stanley Chitekwe[3], Kaleab Baye[4], Ramadhani Noor[3], Anne Sophie Donze[3], Getachew Tollera[1], Mesay Hailu Dangiso[1], Lia Tadesse[5], Meseret Zelalem[5], Masresha Tessema[1*]

[1] Ethiopian Public Health Institute, Addis Ababa, Ethiopia
[2] Regional Nutrition Specialist, United Nations Children's Fund (UNICEF) Regional Office, Dakar, Senegal
[3] United Nations Children's Fund (UNICEF), Addis Ababa 12000, Ethiopia
[4]Center for Food Science and Nutrition, Addis Ababa University, Addis Ababa 12000, Ethiopia
[5]Ethiopian Ministry of Health, Addis Ababa, Ethiopia

*Correspondence to:
Masresha Tessema
PO Box 1242
Ethiopian Public Health Institute, Addis Ababa, Ethiopia
dr.masresha.tessema@gmail.com

For peer review only - http://bmjopen.bmj.com/site/about/guidelines.xhtml

ABSTRACT

Introduction Ethiopia has made significant progress in reducing malnutrition in the past two decades. Despite such improvements, a substantial segment of the country's population remains chronically undernourished and suffers from not only micronutrient deficiencies but also from increasing diet-related non-communicable diseases such as diabetes, hypertension and cancer. This survey aims to assess anthropometric status, dietary intake and micronutrient status of Ethiopian children, women, and adolescent girls. The study will also assess coverage of direct and indirect nutrition-related interventions and map agricultural soil nutrients. The survey will serve as a baseline for the recently developed Ethiopian Food System Transformation Plan and will inform the implementation of the National Food and Nutrition Strategy.

Methods and analysis As a population-based, cross-sectional survey, the study will collect data from the ten regions and two city administrations of Ethiopia. The study population will be women of reproductive age, children aged 0-59 months, school-aged children and adolescent girls. A total of 16,596 households will be surveyed, allowing the generation of national and regional estimates. A two-stage stratified cluster sampling procedure will be used to select households. In the first stage, 639 enumeration areas will be selected using probability-proportional-to-size allocation. In the second stage, 26 eligible households will be selected within each enumeration area using systematic random selection. Primary outcomes include coverage of direct and indirect nutrition interventions, infant and young child feeding practices, food insecurity, dietary intakes, mental health, anthropometric status, micronutrient status, and soil nutrient status.

Ethics and dissemination The protocol was fully reviewed and approved by the Institutional Review Board of the Ethiopian Public Health Institute (protocol no: EPHI-IRB-317-2020). The study is based voluntary participation and written informed consent is required from study participants. The findings will be disseminated via forums and conferences and will be submitted for publication in peer-reviewed journals.

Strengths and limitations of this study

- The survey covers a large geographic area, collecting data on anthropometric status, 24hr recall quantitative dietary intakes and the determination of micronutrient status in the same participants or household, while also capturing data on the food system in Ethiopia.
- The study aims improve understanding of nutritional problems across multiple facets—from agricultural soil to people to the environment in Ethiopia.
- Inherent to the cross-sectional design of the study, the findings of this study cannot be used to establish cause and effect.
- The study design prevents us from considering seasonal differences in nutritional outcomes and determinants.

For peer review only - http://bmjopen.bmj.com/site/about/guidelines.xhtml

INTRODUCTION

Globally, one in every three population are affected by one of more forms of malnutrition.[1] Women and children are particularly vulnerable to malnutrition due to increased physiological nutrient needs required to support fetal and child growth.[2] Nutritional deprivation during early life impairs growth and development, leading to poor school performance, reduced productivity, and loss of earnings in later life.[3] Consequently, the first 1000 days of life, from conception to the child's second year of life, was recognized as a critical window of opportunity to effectively prevent malnutrition.[3 4] Adolescence is also identified as a second window of opportunity to correct nutritional inadequacies and adversities faced in early life, but little is known about this life stage.

Despite significant progress over the past two decades, the burden of malnutrition in Ethiopia remains high.[5-7] Nationally, 37% of Ethiopian children under five years of age are stunted[7], and 22% of women of reproductive age (WRA) are chronically undernourished (Body Mass Index (BMI) < 18.5 Kg/m^2).[5] Only 14% of children under two years of age consumed the minimum number of recommended food groups.[5] Furthermore, micronutrient deficiencies co-exist with chronic energy deficiency.[8] This along with the ongoing nutrition transition, characterized by shifts in diets[9], is further complicating the nutrition landscape by increasing the prevalence of overweight and non-communicable diseases.[5] Nearly a fifth (16%) of Ethiopian adults are estimated to be hypertensive, and 3% are diabetic.[10] Therefore, addressing not only undernutrition but all forms of malnutrition is critical.

The Sustainable Development Goals (SDGs) recognize the importance of nutrition, primarily driven by the need to mitigate its detrimental consequences. Further, the 2012 World Health Assembly (WHA) identified global targets to be achieved by 2025 that aims to reduce stunting, anemia, low birth weight, and childhood obesity. These targets are used to track progress in SDG goal 2: Zero hunger.[11] Recognizing the importance of good nutrition, the Government of Ethiopia has made ending malnutrition a national priority. Ethiopia started implementing its first National Nutrition Program in 2008.[12] The second phase of this program (2011-2016) was a multisectoral program aimed at accelerating progress in reducing malnutrition.[13] Moreover, Ethiopia's first Food and Nutrition Policy was endorsed in 2018[14], followed the National Food and Nutrition Strategy[15] which was launched in 2021 to provide a framework for the operationalization of the policy. Acceleration of progress in the reduction of malnutrition requires the design and implementation of direct and indirect nutrition interventions that can be implemented at scale. To this end, understanding the various factors contributing to the different forms of malnutrition is critical.

Multiple factors operating at the immediate, underlying, and basic levels contribute to malnutrition.[2] Inadequate dietary intake and poor health are immediate determinants.[2] Household food security, child care practices, access to health services, and healthy environments are underlying determinants.[16] Structural and contextual factors such as economic structures, political, environmental, social and cultural factors are the basic determinants of malnutrition.[2] The contribution of these factors varies across different contexts, and target groups, but studies capturing all these factors in a single survey are scant. The lack of timely and comprehensive information on nutritional status across critical

For peer review only - http://bmjopen.bmj.com/site/about/guidelines.xhtml

life stages and their determinants is a bottleneck that is preventing Ethiopia from designing effective interventions. Up to date and comprehensive data on the coverage of direct and indirect nutrition interventions delivered across various implementing sectors of the National Food and Nutrition Strategy is not yet available. This is unfortunate as such data could inform the implementation of the Strategy, but it can also serve as a baseline against which progress can be tracked.

Therefore, this study aims to provide the first ever comprehensive information on the nutritional status of different populations in Ethiopia to support evidence-based implementation of the National Food and Nutrition Strategy.

Objectives

The overall goal of this study will be to produce nationally and regionally representative estimates on anthropometric status, coverage of nutrition interventions, dietary intakes, and micronutrient status for children, adolescent girls, and women of reproductive age in Ethiopia.

Specific objectives include:

1. Assess the coverage of direct and indirect nutrition interventions.
2. Assess food consumption patterns and nutrients intakes of children aged 6–59 months, and women of reproductive age.
3. Assess the micronutrient status of children (vitamin A, anemia, iron, iodine and zinc), adolescent girls, and women of reproductive age (vitamin A, vitamin D, anemia, iron, iodine, zinc, folate, vitamin B_{12})
4. Assess the anthropometric status of under-5 children, school-age children (6-12 years), adolescent girls, and women of reproductive age.
5. Assess the geographical distribution of soil micronutrient status in Ethiopian agricultural soil.

METHODS AND ANALYSIS

Study design

This study is a nationally and sub-nationally (regionally) representative cross-sectional survey that will characterize dietary intake, micronutrient status, and access to nutrition-related services for different target populations. Given that soil nutrient content can influence micronutrient content of foods and hence affect nutrient intake, the soil nutrient composition will also be analyzed. The study will have four main components. The first component will assess nutrition-specific and nutrition-sensitive indicators (NSS) for all target groups (children aged 0-59 months and WRA, school-age children, and adolescent girls) using semi-structured questionnaires. The second component will measure quantitative dietary intake for children aged 6-59 months and WRA (15-49 years). The third component of the survey will collect biomarker samples from all children (6-59 months), school-age children (6-12 years), adolescent girls (10-19 years), and WRA (15-49 years). The final component of the study will measure micronutrients in agricultural soils. The study data will be collected from July 2021 to December 2023.

Setting

For peer review only - http://bmjopen.bmj.com/site/about/guidelines.xhtml

Ethiopia has an estimated population size of 120 million and is the second most populous country in Africa.[17] The majority of its population resides in rural areas (70%).[17] Agriculture accounts for 40% of the country's gross domestic product.[17] Children aged 15 years and younger make up 40% of the Ethiopian population in 2021.[18] Ethiopia is administratively divided into 10 regions and two city administrations. This study will be conducted in all of the regions and city administrations of the country. Figure 1 provides a geographic representation of the study areas.

Participants

The target population of this study are i) women of reproductive age (WRA) aged 15-49 years ii) children aged 0-59 months iii) school-age children aged 6-12 years, and iv) adolescent girls aged 10-19 years, and V) household head.

Sample size calculations

Sample size was estimated to guarantee adequate precision to generate national and regional estimates for selected indicators for each study target group. Indicators used for each target group are shown in Supplemental Table 1S. The required number of households and target groups was calculated using a single population proportion formula at the regional level. We used region-specific prevalence estimates for indicators, a 5% margin-of-error, a design effect of 1.5, a household response rate of 95%, and an individual response rate of 80%. The initial sample size was then adjusted for region-specific average household size and percentage of the target population from the total population. An indicator that provides the maximum number of households was used to estimate the final sample size for each region. Regional sample sizes were summed up to derive the total (national) sample size. Based on these calculations, the total sample size for the overall survey was 16,596 households (Supplemental Table 2S).

For WRA, dietary and biomarker data will be collected in half of the selected households within each Enumeration Area (EA). This selection will yield a total sample size of 7,386 WRA (50% of the expected 14,772 WRA). The sample size needed to assess dietary intakes and micronutrient status of WRA was calculated using the prevalence of inadequate zinc intake, which yielded the largest sample size.[8 19]

Sampling procedures

A two-stage stratified cluster sampling procedure will be used to select households. In the first stage, 639 EAs, 257 urban and 382 rural will be selected using probability-proportional-to-size allocation. We will use the 2018 Ethiopia Population and Housing Census enumeration areas sampling frame to select EAs (the Primary Sampling Units (PSUs)). The Central Statistical Agency (CSA) prepared the enumeration areas sampling frame. An EA typically contains 100-150 households. EA maps will be used to delineate the boundaries of the selected EA. In the second stage of sampling to identify eligible households, all households with the EA will be listed. A household will be eligible for selection if at least one of the study target groups are residents (de jure) or stayed at the household the night before the interview (de factor).

Twenty-six (26) eligible households will be selected within each EA using systematic random selection. All target groups will be eligible for the NSS interview in the selected households. All children aged 6-59 months will also be eligible for dietary assessment. Women residing in 13 households (Out of 26 households) who will be selected randomly will be eligible for dietary assessment. Biomarker samples

For peer review only - http://bmjopen.bmj.com/site/about/guidelines.xhtml

will be collected for all children under 5 years of age, school-age children, and adolescents in the selected households. Similar to dietary assessment, biomarker samples will be collected for women residing in half of the selected households (Figure 2).

Outcomes

Coverage of direct and indirect nutrition interventions

A structured questionnaire will be used to determine the coverage of direct and indirect nutrition interventions provided to children aged 6-59 months, WRA, and adolescent girls. Direct nutrition interventions included vitamin A supplementation, iron supplementation, zinc supplementation, growth monitoring and promotion, nutrition counseling services, and food fortification. Water, sanitation and hygiene, coverage of food or cash assistance program, women empowerment, and mental health will be some nutrition-sensitive indicators considered in this study (Table 1). We will use standard indicator definitions proposed by the Data for Decisions to Expand Nutrition Transformation project (DataDENT) to assess coverage of nutrition programs.

Anthropometric status

Using standardized procedures, anthropometric measurements, including weight, height/length, and mid-upper arm circumference, will be taken for all study target populations.[20] Anthropometric indices (weight-for-height z-scores, length/height-for-age z-scores, weight-for-age z-scores, BMI-for-age z-scores) will be calculated using the WHO 2006 child growth standards and the WHO 2007 child growth reference data. Stunting (length/height-for-age z-scores below -2 SD), wasting (weight-for-height z-scores below -2 SD), underweight (weight-for-age z-scores below -2 SD), thinness (BMI-for-age z-scores below -2 SD) and BMI will be the primary anthropometric outcomes of interest.

Infant and young child feeding practices

Infant and young child feeding practices will be assessed using the new World Health Organization (WHO) and United Nations Children's Fund (UNICEF) recommended 17 indicators to evaluate IYCF practices.[21]

Food insecurity

The Food Insecurity Experience Scale (FIES) will be used to assess household food security.[22] The FIES consists of eight questions that assess household experience related to adequate food access. Experience questions range from worrying about getting enough food to not eating for a whole day.

In addition to these outcome indicators, information on the sociodemographic characteristic of households, child health, maternal health, employment status, and household agricultural practices will be collected using structured questionnaires.

Mental health of women

Common mental health disorders will be assessed using the WHO Self-reporting questionnaire which consists of 20 questions. Women will be classified as having a common mental health disorder if the row score will be greater or equal to 6 out of 20.[23]

Assessment of dietary intakes of children and women of reproductive age

For peer review only - http://bmjopen.bmj.com/site/about/guidelines.xhtml

We will measure dietary intake for children aged 6-59 months and WRA. A one-day quantitative multiple-pass 24-hour recall will be conducted to assess dietary intakes. The interactive multiple-pass 24-hour recall interview consists of four steps designed to enhance memory.[24] All days of the week will be proportionately represented during the dietary survey to account for the day of the week effects on food intake. To account for the day-to-day variability of dietary intake within individuals, a second non-consecutive day 24-hr recall (repeat) will be collected (within 2 to 10 days of the first recall) on a randomly selected sub-sample of WRA and children. The number of repeats needed is determined by allocating for each region 50 repeats, which is then multiplied by a design effect of 1.5 and a 10% non-response rate. The number of repeats will be rounded up to 1244 recalls for each target group to ensure that the minimum number of repeats (n=83) needed from each region would be collected. Detailed non-standard recipe ingredient data will be collected for all mixed dishes that were prepared at home.

We will use 15 food groups to assess the dietary intakes of women (15-49 months) and children aged 24-59 months. These food groups were: 1) Cereals and their products, 2) Starchy Roots and tubers, and their products, 3) Pulses, and their products, 4) Vegetables and their products, 5) Fruits and their products, 6) Meat, and poultry their products, 7) Eggs and their products, 8) Fish, shellfish and their products, 9) Milk and milk products, 10) Fats and oils, 11) Nuts and seeds, 12) Sugar and sweetened products, 13) Beverages, 14) Spices and condiments, and 15) Miscellaneous. For children aged 6-23 months, we will use the updated WHO, UNICEF food groups: 1) Breastmilk, 2) Grains, roots, and tubers, 3) Pulses, nuts, and seeds, 4) Dairy products, 5) Flesh foods (meats, fish, poultry, organ meats), 6) Eggs 7) Vitamin-A rich fruits and vegetables, and 8) other fruits and vegetables. These food groups were adapted from the FAO/WHO Global Individual Food consumption data Tool (GIFT) food groups.[25]

Dietary assessment pre-survey work: We carried out pre-survey work to aid dietary data collection following recommendations set by the Intake: Center for Dietary Assessment.[26 27] An initial step will be developed a food and ingredient list containing a comprehensive list of food items, mixed dishes, and ingredients expected to be consumed by the study target groups. The food list will be generated using data from the first 2011 Ethiopian National Food Consumption Survey.[19] Other common foods consumed across the regions in Ethiopia will be derived from the 2016 Household Income and Expenditure Surveys,[28] the Ethiopian Food Composition Tables, and dietary intake data from other recent dietary assessment surveys conducted by the Ethiopian Public Health Institute (EPHI). Portion size estimation methods suitable for large-scale studies will be pre-selected for use in the survey following Intake recommendations.[29] The selected methods will be direct measurement of actual foods consumed, standard unit: size and number, proxy measurement using play dough, water, rice, and maize flour, and finally using food price to estimate the amount of food consumed. Portion size estimation methods will be assigned for all foods included in the food list.

Table 1. Nutrition direct and indirect interventions coverage

No	Indicator	Target population
	**Nutrition indirect intervention coverage**	
	Child interventions	
1.	Children received iron tablets/syrup in the last 12 months	Children aged 6-59 months

For peer review only - http://bmjopen.bmj.com/site/about/guidelines.xhtml

2.	Children received vitamin A supplements in the past 6 months	Children aged 6-59 months
3.	Children received deworming tablets in the past 6 months	Children aged 24-59 months
4.	All 8 basic vaccinations: one dose of BCG, three doses of DPT, three doses of the polio vaccine, and one dose of the measles vaccine	Children aged 9-59 months
5.	No Vaccination	Children aged 0-59 months
	Growth monitoring	
6.	Weight measured in the last 3 months	Children aged 0-23 months
7.	Height measured in the last 3 months (Optional)	Children aged 0-23 months
8.	MUAC measured in the last 3 months (Optional)	Children aged 0-23 months
	Infant and young child feeding (IYCF) counselling	
9.	Mothers with children 6-23 months received any IYCF counseling	Children aged 6-23 months
10.	Mothers with children 6-23 months received age-appropriate IYCF counseling	Children aged 6-23 months
	Early breast-feeding counseling	
11.	Women *received breastfeeding counseling with observation during the first two days after birth*	Women aged 15-49 years with a live birth in the past 5 years for the most recent birth
12.	Women *received breastfeeding counseling during the first month after birth*	Women aged 15-49 years with a live birth in the past 5 years for the most recent birth
	Coverage of Nutritional Interventions during pregnancy/antenatal Care (ANC)	
13.	Percentage of women who had 4 or more ANC visits for the most recent birth	Women aged 15-49 years with a birth in the last 5 years
14.	Percentage of women who received counseling about healthy eating during pregnancy	Women aged 15-49 years who received antenatal care for their most recent birth
15.	Percentage of women whose weight gain was monitored during pregnancy	Women aged 15-49 years who received antenatal care for their most recent birth
16.	Women received food or cash assistance during pregnancy	Women aged 15-49 years with a birth in the last 5 years
17.	Women took 90+ iron/folate tablets during pregnancy	Women aged 15-49 years with a live birth in the past 5 years for the most recent birth
18.	Women received deworming tablets during pregnancy	Women aged 15-49 years with a live birth in the past 5 years for the most recent birth
	**Nutrition indirect intervention coverage**	
19.	Basic water services	Household
20.	Basic hygiene services	Household
21.	Basic Sanitation services	Household
22.	Food insecurity (not a service hence no coverage)	Household
23.	Women received food or cash assistance during	Women aged 15-49 years with a

For peer review only - http://bmjopen.bmj.com/site/about/guidelines.xhtml

	pregnancy	live birth in the past 5 years for the most recent birth
24.	Basic water services	Household
25.	Basic hygiene services	Household
26.	Basic Sanitation services	Household
27.	Presence of common mental health disorders in the past month	Women aged 15-49 years
28.	Women empowerment	Women aged 15-49 years
29.	Livestock ownership	Household
30	Agricultural productivity by food group	Household

Assessment of micronutrient status

Blood specimens will be collected from the study population to determine serum retinol, ferritin, soluble transferrin receptor (sTfR), zinc, folate, vitamin B12, red blood cell (RBC) folate and 25-hydroxyvitamin D. Additionally, markers of inflammation, alpha(1)-acid glycoprotein (AGP), high-sensitivity C-reactive protein (hsCRP) will also be measured. We will also analyze parasites from stool specimens. All laboratory analyses will be performed at the EPHI Clinical chemistry, and Food Science and Nutrition Laboratories. Both laboratories participate in an external quality assessment scheme and are accredited by the Ethiopian National Accreditation Office (ENAO). Collection, storage, and analytical procedures for blood, urine, stool, and salt samples are described below. The details of each biomarker analysis are described in Supplemental Materials 1-11.

Blood sample collection and analysis: Venous blood samples (5-7 mL) will be collected using vacutainer tubes following standard operating procedures.[30] Trace mineral-free vacutainer tubes will be used to collect blood for trace metal analysis. After collection, blood samples will be allowed to clot for 30 minutes in cold boxes (<8 °c). Samples will then be centrifuged at 3000 rpm (revolution per minute) for 10 minutes. The separated serum will be aliquoted and stored in -20°c portable freezers in the field. Samples will then be transported to EPHI and stored at -80°c until analysis. Hemoglobin will be measured in the field using Hemocue® (Hb 301, Hemocue AB, Angelholm, Sweden)[31].[31] If the hemoglobin values are below WHO cutoff point(11g/dl), the phlebotomist will send whole blood samples to the EPHI laboratory to identify hemoglobinopathies using electrophoresis method.[32] Malaria test will be conducted on-site using Bioline™ Malaria Ag P.f rapid diagnostic test kits (RDT) for P. falciparum and P. vivax).[33] Serum soluble transferrin receptor (sTfR), AGP, hsCRP, folate, red blood cell (RBC) folate, vitamin B_{12}, and ferritin will be measured using Cobas 6000 analyzer (Roche Diagnostics GmbH, Mannheim, Germany). Serum retinol will be measured using high-performance liquid chromatography (HPLC) method[33], and serum zinc and selenium will be measured using a microwave plasma atomic emission spectrometers (MP-AES) analyzer.

Stool and urine sample collection and analysis: Stool samples will be collected using stool cups and stored in 10 % formalin to preserve the parasite until analysis.[34] A portion of each stool sample will be used to detect direct ova, larvae and cysts of intestine parasites using formal ether concentration technique.[35] Urine samples will be collected from WRA and school-age children using 60 ml urine cup

For peer review only

For peer review only - http://bmjopen.bmj.com/site/about/guidelines.xhtml

containers. Samples will be stored at -20°c. Urinary iodine excretion will be assessed by Sandell Kolthoff reaction at EPHI Laboratory using Shimadzu 1800 UV-Vis spectroscopy.[36]

Salt collection and analysis: Salt samples will be collected from households with WRA for whom dietary data will be collected. At least 25 grams (one coffee cup) of salt will be collected to determine iodine content using the iodometric titration method.[37]

Assessment of nutrients in the soil

Soil samples will be collected from three households in each EA. Zig-zag or cross-sampling method will be used to collect 10 to 20 subsamples (0-30 cm depth) constituting one composite sample. Subsamples will be collected at a separation distance of five meters. After thoroughly mixing composite samples, 1 kg soil sample will be transferred to polyethylene bags. The collected soil samples will be air-dried in wooden trays and disaggregated using a ceramic mortar and pestle (soil grinder) at the EPHI soil laboratory. Samples will then pass through a 6 mm sieve of stainless-steel screens to remove debris and homogenize the soil sample. The sieved fraction will be further pulverized to pass through a 1 mm sieve for the micronutrient analysis. Soil zinc, iron, copper, and manganese will be determined following standard procedures.[38] Micronutrient content will be determined using inductively coupled plasma-optical emission spectroscopy (ICP-OES) after extraction with diethylene triamine penta acetic acid (DPTA). Additional variables that affect the mobility of micronutrients in the soil and their uptake into crops will also be measured. These variables include soil reaction (pH), electrical conductivity, organic matter, total nitrogen, and soil organic carbon content. Data collectors will also record topography, slope, cropping history, type, and fertilizer application information. Table 2 provides a summary of procedures for each of the four components of the survey by study target groups.

For peer review only - http://bmjopen.bmj.com/site/about/guidelines.xhtml

Table 2. Summary of data collection procedures for each of the four components of the survey

	Child 0-5 months	Child 6-23 months	Child 24-59 months	School children 6-12 years	Adolescent girls 10-19 years	WRA 15-49 years	Household
**Nutrition direct and indirect intervention indicators**							
Infant and young child feeding practices	X	X					
Nutritional information for adolescent girls					X		
Food insecurity							X
Water, sanitation and hygiene practices							X
Coverage of food fortification							X
Agricultural practices							X
Mental health						X	
Anthropometric status	X	X	X		X	X	
**Dietary assessment**							
24-hr recall quantitative dietary intake		X	X			X	
**Assessment of biomarker status**							
Blood sample		X	X	X	X	X	
Urine sample				X		X	
Stool sample		X	X	X	X	X	
Salt sample collection							X
**Assessment of micronutrients in the Soil**							
Soil micronutrient assessment							X

For peer review only

Data quality assurance and analysis

Training of trainers on components of the survey will be held before training the data collectors and supervisors. After fifteen days of training on methodological procedures, questionnaires and quality assurance, the questionnaires will be tested in a pilot group (in EAs not included in the actual survey), and adapted based on the received feedback from the survey team. The questionnaires (including the food list) were translated into local languages (Amharic, Oromifa, Tigrigna, Somali, and Afar) and back-translated to English to ensure the quality of the translation. The data collectors' measurements will be standardized to ensure that the inter-observer variability is within tolerable limits. Supervisors received additional training on teamwork and on monitoring and supervising the data collection process. All data collection tools are programmed using open-source software (ODK) (Supplemental Material 12). Data quality checks will be included during ODK programming to prevent data recording errors. These include restricted responses, filter insert choices, skip patterns, and defaults. During data collection daily data tracking forms will be completed to track completed surveys for each study components to prevent missing data. High frequency checks will be identified prior to the survey's, and error tracking forms will be designed to track data quality in real-time. These checks included completeness checks, target group tracking, and duplicate ID checks. Random field supervision visits will also be made to check data quality. Every day, collected data will be sent to the EPHI central server and imported into statistical software programs as comma-separated values (CSV) files. For laboratory analysis, a quality control chart will be used to ensure the internal and external quality control materials are in the acceptable range.

The primary data analysis will focus on computing frequencies and percentages for categorical variables and summary statistics (like means, medians SD, IQR) for summarizing continuous variables. Sample weights will be constructed based on the selection probabilities of EAs, eligible households, and non-response rates. All analyses will also be adjusted for the survey design. Additional subgroup analysis will be computed for variables with adequate sample sizes for each category. The Biomarkers Reflecting Inflammation and Nutrition Determinants of Anemia (BRINDA) Working Group's regression correction approach will be used to account for inflammation in the study of all micronutrients status using the biomarkers C-reactive protein (CRP) and AGP. Geostatistical analyses will be employed to determine the spatial patterns of micronutrient distribution in the soil and blood samples. The wealth index will be constructed using principal component analysis (PCA).[39] The Rasch model will be used to construct the Food Insecurity Experience Scale (FIES). [22] All analyses will be done using STATA 16 and ArcGIS/QGIS. Anthropometric indices will be calculated using the WHO Anthro software for under five children and WHO AnthroPlus software for adolescents.

Patient and public involvement

None.

Ethics and dissemination

The study protocol is approved by the Institutional Review Board of the Ethiopian Public Health Institute (protocol no: EPHI-IRB-317-2020). Written informed consent will be obtained from each respondent and participants may withdraw at any time (Supplemental Material 13). Confidentiality of all collected data

For peer review only - http://bmjopen.bmj.com/site/about/guidelines.xhtml

will be given high priority during each stage of data handling. Individual names and personal information of respondents will be kept confidential and data sets will be kept anonymous for analysis.

The study's findings will be disseminated through several communication channels, including stakeholder workshops, various local and international conferences and technical reports. Additionally, the findings will be submitted for publication in peer-reviewed journals.

DISCUSSION

This comprehensive, nationally representative survey will for the first time characterize simultaneously the dietary intake and micronutrient status of Ethiopian children, adolescent girls and WRA. Besides, the study assesses key drivers of malnutrition including soil nutrient composition, as well as coverage of direct and indirect nutrition interventions. The survey will provide key insights informing the implementation of Ethiopia's National Food and Nutrition Strategy.

High-quality and timely data is critical to assess the burden of nutritional problems, identify vulnerable populations and priority actions, track the implementation of nutrition programs, and assess impact.[40 41] Ethiopia conducted its first-ever food consumption survey in 2011.[19] and its micronutrient survey in 2015.[42] Both surveys were collected at different times, which made it difficult to link the two surveys. Besides, the causes and solutions of malnutrition are complex and multisectoral; hence, requiring data on multiple indicators from various sectors spanning from soil nutrient, diets, and micronutrient status, to access and exposure to direct and indirect nutrition interventions. In this regard, this survey is uniquely positioned to integrate data from multiple domains to support evidence-based decision making for improved diets, nutrition, and overall wellbeing.

This study will allow us to evaluate progress relative to previous food consumption and micronutrient surveys, but, more importantly, will serve as a baseline against which future progress related to the implementation of the National Food and Nutrition Strategy will be evaluated. Furthermore, the current survey will also serve as a baseline for the Ethiopian Food System Transformation Plan by capturing the majority of indicators used for monitoring food systems-related progress, thus filling information gaps that could have impeded successful implementation of the National Food and Nutrition Strategy. By establishing 13 strategic objectives, the National Food and Nutrition Strategy is intended to be aligned with the strategic directions of the Food and Nutrition Policy. Each strategy direction includes initiatives, actions, and key performance indicators, as well as leading and collaborating sectors. The key performance indicators should be evaluated to determine the progress of each implementing sector's achievement. The current survey will provide up to date national and subnational information on the current food and nutrition situation in Ethiopia for different target populations as well as provide comprehensive list of indicators that are pertinent to the implementation of the policy.[40] In addition, this study will provide information on context-specific determinants for prioritizing direct and indirect actions that can be implemented across sectors taking into account the specific needs of different target populations.

Additionally, effective multisectoral interventions that address the immediate and underlying determinants of malnutrition must be implemented in order to accelerate the reduction of malnutrition in its all form.[40] These interventions need to address context-specific determinants to reduce

For peer review only - http://bmjopen.bmj.com/site/about/guidelines.xhtml

malnutrition effectively.[40] The lack of timely and disaggregated information on the determinants of malnutrition is a bottleneck to preventing malnutrition, particularly among the most vulnerable target populations. This study will also provide information on the coverage and quality of interventions which can be used to contextualize National Food and Nutrition Strategy monitoring frameworks, monitor implementation and track progress towards global and local targets.

Although this study will provide regionally and nationally representative estimates for key indicators and critical life stages, it has several limitations. Inherent to the cross-sectional design of the study, the findings of this study cannot be used to establish cause and effect. Additionally, the design prevents us from considering seasonal differences in nutritional outcomes and determinants. This study also relies on self-reported data, which is subject to recall bias. Notwithstanding the above-mentioned limitations, this study is uniquely designed to combine the assessment of anthropometric status, 24hr recall quantitative dietary intakes and the determination of micronutrient status in the same participants, while at the same time capturing data on the food system. Additionally, the study will be evaluating micronutrients in agricultural soil, which will expand our understanding of factors that influence nutrition. To the best of our knowledge, this will be among -if not-the first study to simultaneously collect these variables from the same household. This could contribute to a better understanding of nutritional problems across multiple facets—from soil to people to the environment. In the past, nutrition programs implemented in Ethiopia have relied on information provided from small-scale studies and population-based surveys such as the Ethiopia Demographic and Health Surveys.[5-7 43 44] Although these data sources provide some information to track progress and tailor interventions, they only provide data on a limited number of nutrition indicators and do not measure dietary intakes and assess biomarker status. This study will fill these data gaps by providing information on comprehensive indicators that show the burden and spatial distribution of micronutrient deficiencies and shifts in dietary patterns. Additionally, this study will provide information on emerging determinants such as mental health and intake of nutrients such as folate and B_{12} that have not been included in previous studies. Finally, the inclusion of adolescent girls, and school-age children, will provide vital information on nutritional indicators for these target groups, which are often not included in other nationally representative surveys. This survey will also provide information on the coverage of direct interventions implemented in the health sectors and indirect interventions implemented in the agriculture, WASH, education and social protection sectors for whom scant data exists. Hence, this study will provide valuable information that will guide the implementation of strategic actions for the reduction of malnutrition in Ethiopia.

For peer review only - http://bmjopen.bmj.com/site/about/guidelines.xhtml

Contributors: MT, AL, SC, MW, AP, AS, and MG conceived the study and drafted the original protocol. All authors participated in refining of the protocol. AH, MW, MG and MT played a major role in the statistical consideration. DAD, FC, MG, RN, and ASD helped to write the draft protocol and made a critical contribution to the content. KB, AL, GT, MHD, LT and MZ supervised manuscript preparation. All authors took responsibility for reviewing, final editing and approval of the manuscript.

Funding: This work was supported by Power of Nutrition, BMGF and World Bank through UNICEF; Nutrition International, and Global Alliance for Improved Nutrition.

Competing interests: The authors declare that they have no conflicts of interest.

Patient consent for publication: Not applicable.

For peer review only

For peer review only - http://bmjopen.bmj.com/site/about/guidelines.xhtml

References

1. Fanzo J, Hawkes C, Udomkesmalee E, et al. 2018 Global Nutrition Report. 2019
2. Olofin I, McDonald CM, Ezzati M, et al. Associations of suboptimal growth with all-cause and cause-specific mortality in children under five years: A pooled analysis of ten prospective studies. *PLoS One* 2013;8(5):e64636. doi: 10.1371/journal.pone.0064636
3. Martorell R. Improved nutrition in the first 1000 days and adult human capital and health. *Am J Hum Biol* 2017;29(2) doi: 10.1002/ajhb.22952 [published Online First: 2017/01/25]
4. United Nations. Transforming our world: The 2030 agenda for sustainable development, 2015.
5. Central Statistical Agency [Ethiopia] and ICF. Ethiopia demographic and health survey 2016. Addis Ababa, Ethiopia and Calverton, Maryland, USA: CSA and ICF, 2016.
6. Central Statistical Agency [Ethiopia] and ORC Macro. Ethiopia demographic and health survey 2000. Addis Ababa, Ethiopia and Calverton, Maryland, USA: CSA and ORC Macro, 2001.
7. Ethiopian Public Health Institute (EPHI) [Ethiopia] and ICF. Ethiopia Mini Demographic and Health Survey 2019: Key Indicators. Rockville, Maryland, USA: EPHI and ICF, 2019.
8. Ethiopian Public Health Institute. Ethiopian national micronutrient survey report Ethiopian Public Health Institute, 2016.
9. Hassen I, Dereje M, Minten B, et al. Diet transformation in Africa: the case of Ethiopia. *Agricultural Economics* 2017
10. Ethiopian Public Health Institute, Federal Ministery of Health, World Health Organization. Ethiopia STEPS report in risk factors for non-communicable diaseses and prevalence of selected NCDs Addis Ababa,Ethiopi: Ethiopian Public Health Institute 2016.
11. World Health Organization. Global nutrition monitoring framework: Operational guidance for tracking progress in meeting targets for 2025. Geneva: WHO, 2017.
12. Federal Democratic Republic of Ethiopia. National Nutrtion Program: 2013-2015. Addis Ababa,Ethiopia, 2013.
13. Federal Democratic Republic of Ethiopia. National Nutrition Program: 2016-2020. Addis Ababa,Ethiopia., 2016.
14. Federal Democratic Republic of Ethiopia. Food and Nutrition Policy, 2018.
15. Federal Democratic Republic of Ethiopia. National Food and Nutrition Strategy (2021-2023), 2021.
16. Reinhardt K, Fanzo J. Addressing Chronic Malnutrition through Multi-Sectoral, Sustainable Approaches: A Review of the Causes and Consequences. *Front Nutr* 2014;1:13. doi: 10.3389/fnut.2014.00013 [published Online First: 2014/01/01]
17. Bank W. Population projections Ethiopia 2021 [
18. Bardosh KL, Hussein JW, Sadik EA, et al. Chicken eggs, childhood stunting and environmental hygiene: an ethnographic study from the Campylobacter genomics and environmental enteric dysfunction (CAGED) project in Ethiopia. *One Health Outlook* 2020;2(1) doi: 10.1186/s42522-020-00012-9
19. Ethiopian Public Health Institute. Ethiopian national food consumption survey Ethiopian Public Health Institute, 2013.
20. UNICEF Wa. Recommendations for data collection, analysis and reporting on anthropometric indicators in children under 5 years old. : World Health Organization and the United Nations Children’s Fund (UNICEF), 2019.
21. WHO, UNICEF. Indicators for assessing infant and young child feeding practices: definitions and measurement methods. Geneva: World Health Organization and the United Nations Children’s Fund (UNICEF), 2021.
22. Ville AS, Tsun Po JY, Sen A, et al. Food security and the Food Insecurity Experience Scale (FIES): ensuring progress by 2030. *Food Security* 2019;11(3):483-91. doi: 10.1007/s12571-019-00936-9

For peer review only - http://bmjopen.bmj.com/site/about/guidelines.xhtml

23. World Health Organization. Self-reported mental health questionnaires1994.
24. Gibson RS, Ferguson EL. An interactive 24-hour recall for assessing the adequacy of iron and zinc intakes in developing countries. HarvestPlus 2008.
25. Leclercq C, Allemand P, Balcerzak A, et al. FAO/WHO GIFT (Global Individual Food consumption data Tool): a global repository for harmonised individual quantitative food consumption studies. *Proc Nutr Soc* 2019;78(4):484-95.
26. Vossenaar M, Arimond M, Deitchler M, et al. An overview of the main pre-survey tasks required for large-scale quantitative 24-Hour recall dietary surveys in low- and middle-income countries. Washington, DC: Intake – Center for Dietary Assessment/FHI Solutions., 2020.
27. Intake – Center for Dietary Assessment. Dietary Survey Protocol Template: An Outline to Assist with the Development of a Protocol for a Quantitative 24-Hour Dietary Recall Survey in a Low- or Middle-Income Country Washington, DC: Intake – Center for Dietary Assessment/FHI Solutions., 2020.
28. Central Statistical Agency[Ethiopia]. Household Consumption and Expenditure (HCE) Survey 2015/16: Analytical Report. Addis Ababa: CSA, 2016.
29. Vossenaar M, Lubowa A, Hotz C, et al. Considerations for the Selection of Portion Size Estimation Methods for Use in Quantitative 24-Hour Dietary Recall Surveys in Low- and Middle-Income Countries. Washington, DC: Intake – Center for Dietary Assessment/FHI Solutions, 2020.
30. Organization WH. Micronutrient survey manual. 2020
31. Jaggernath M, Naicker R, Madurai S, et al. Diagnostic Accuracy of the HemoCue Hb 301, STAT-Site MHgb and URIT-12 Point-of-Care Hemoglobin Meters in a Central Laboratory and a Community Based Clinic in Durban, South Africa. *PloS one* 2016;11(4):e0152184. doi: 10.1371/journal.pone.0152184 [published Online First: 2016/04/06]
32. Wieringa FT, Dahl M, Chamnan C, et al. The High Prevalence of Anemia in Cambodian Children and Women Cannot Be Satisfactorily Explained by Nutritional Deficiencies or Hemoglobin Disorders. *Nutrients* 2016;8(6) doi: 10.3390/nu8060348 [published Online First: 2016/06/25]
33. Lee GC, Jeon ES, Le DT, et al. Development and evaluation of a rapid diagnostic test for Plasmodium falciparum, P. vivax, and mixed-species malaria antigens. *Am J Trop Med Hyg* 2011;85(6):989-93. doi: 10.4269/ajtmh.2011.11-0265 [published Online First: 2011/12/07]
34. Demeke G, Fenta A, Dilnessa T. Evaluation of Wet Mount and Concentration Techniques of Stool Examination for Intestinal Parasites Identification at Debre Markos Comprehensive Specialized Hospital, Ethiopia. *Infect Drug Resist* 2021;14:1357-62. doi: 10.2147/IDR.S307683 [published Online First: 2021/04/17]
35. Kache R, Phasuk N, Viriyavejakul P, et al. Prevalence of soil-transmitted helminth infections and associated risk factors among elderly individuals living in rural areas of southern Thailand. *BMC public health* 2020;20(1):1882. doi: 10.1186/s12889-020-09986-7 [published Online First: 2020/12/09]
36. Machado A, Lima L, Mesquita RBR, et al. Improvement of the Sandell-Kolthoff reaction method (ammonium persulfate digestion) for the determination of iodine in urine samples. *Clin Chem Lab Med* 2017;55(9):e206-e08. doi: 10.1515/cclm-2016-1094 [published Online First: 2017/03/18]
37. Jooste PL, Strydom E. Methods for determination of iodine in urine and salt. *Best Pract Res Clin Endocrinol Metab* 2010;24(1):77-88. doi: 10.1016/j.beem.2009.08.006 [published Online First: 2010/02/23]
38. Lindsay WL, Norvell W. Development of a DTPA soil test for zinc, iron, manganese, and copper. *Soil Sci Soc Am J* 1978;42(3):421-28.
39. Karamizadeh S, Abdullah SM, Manaf AA, et al. An overview of principal component analysis. *Journal of Signal and Information Processing* 2020;4

For peer review only - http://bmjopen.bmj.com/site/about/guidelines.xhtml

40. Heidkamp RA, Piwoz E, Gillespie S, et al. Mobilising evidence, data, and resources to achieve global maternal and child undernutrition targets and the Sustainable Development Goals: an agenda for action. *The Lancet* 2021 doi: 10.1016/s0140-6736(21)00568-7
41. Victora CG, Christian P, Vidaletti LP, et al. Revisiting maternal and child undernutrition in low-income and middle-income countries: variable progress towards an unfinished agenda. *The Lancet* 2021 doi: 10.1016/s0140-6736(21)00394-9
42. Institute EPH. Ethiopian national micronutreint Survey. Addis Ababa, Ethiopia Ethiopian Public Health Institute

2016.
43. Central Statistical Agency [Ethiopia] and ICF. Ethiopia demographic and health survey 2011. Addis Ababa, Ethiopia and Calverton, Maryland, USA: CSA and ICF, 2011.
44. Central Statistical Agency [Ethiopia] and ORC Macro. Ethiopia demographic and health survey 2005. Addis Ababa, Ethiopia and Calverton, Maryland, USA: CSA and ORC Macro, 2006.

FIGURE TITLES

Figure 1. Map showing study enumeration areas across regions

Figure 2. Sampling frame for the Ethiopia National Food and Nutrition Survey to inform the Ethiopian National Food and Nutrition Strategy

For peer review only

For peer review only - http://bmjopen.bmj.com/site/about/guidelines.xhtml

For peer review only

For peer review only - http://bmjopen.bmj.com/site/about/guidelines.xhtml

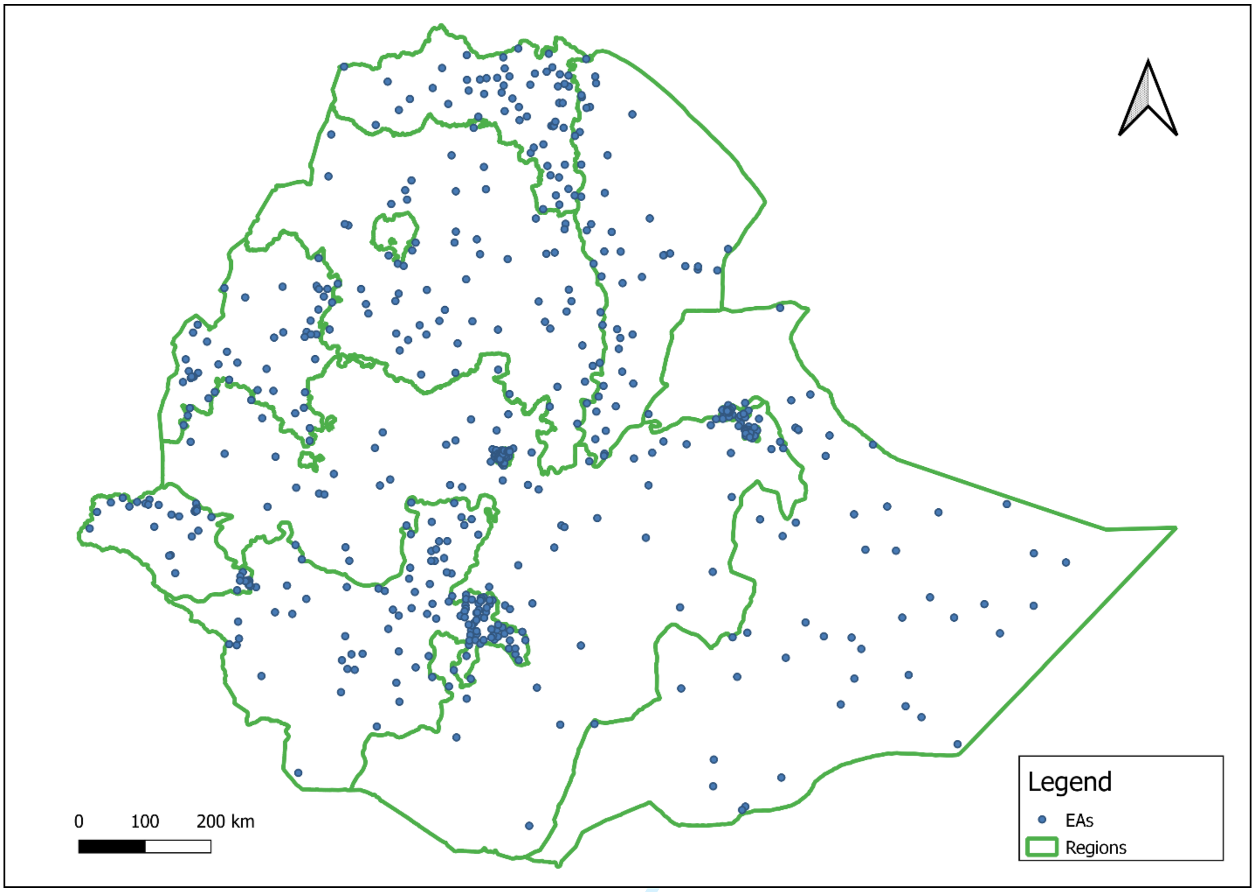

Figure 1. Map showing study enumeration areas across regions

For peer review only

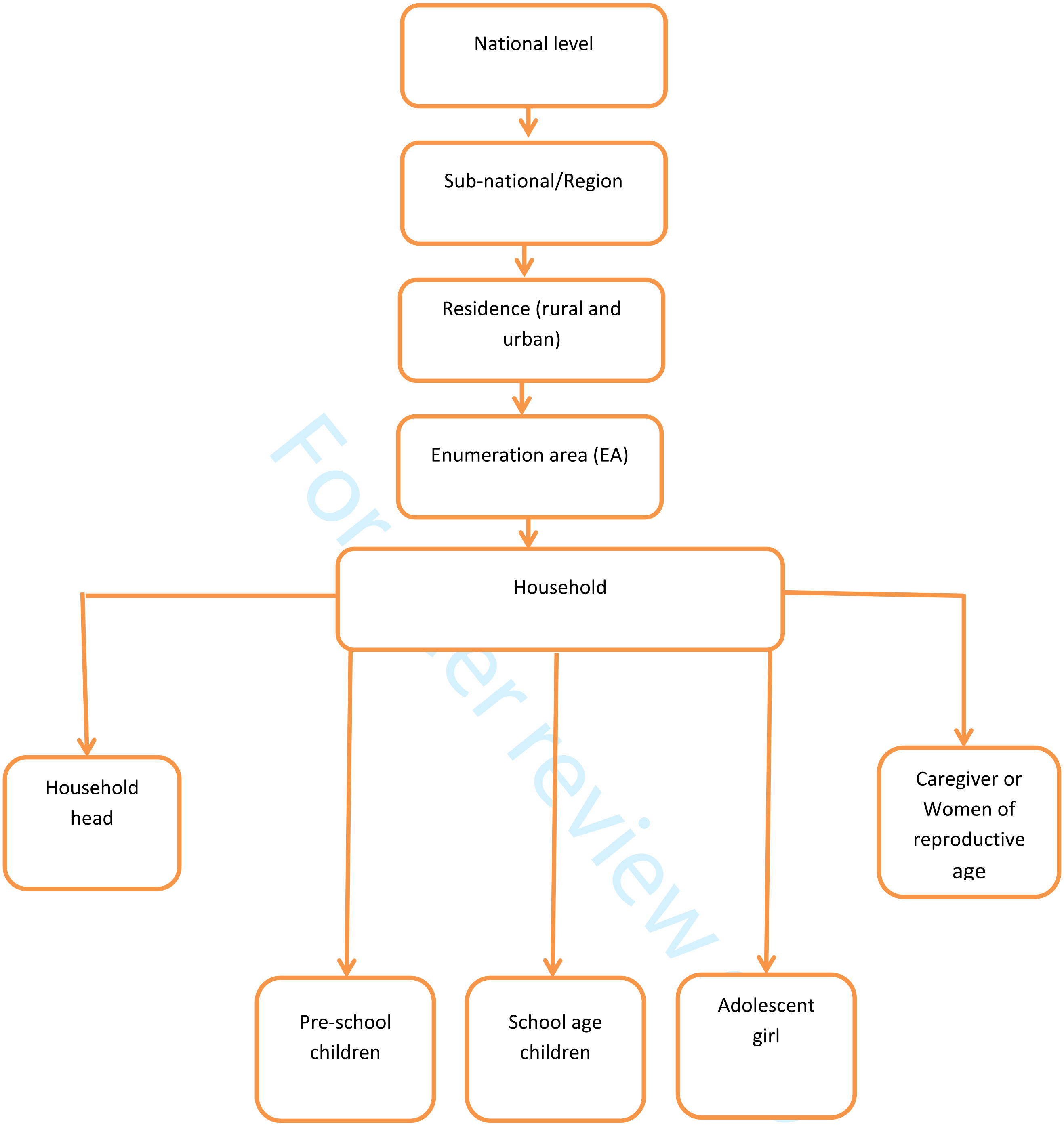

Figure 2. Sampling frame for Ethiopia National Food and Nutrition Survey to Inform the Ethiopian Food and Nutrition Strategy

For peer review only - http://bmjopen.bmj.com/site/about/guidelines.xhtml

Table 1S. Key indicators used to estimate sample size for each target group

Target Group	Key indicators used to estimate sample size
Children under 5 years of age (0-59 months)	Vitamin A deficiency<br>Total goiter prevalence<br>Stunting<br>Any anemia<br>Zinc deficiency<br>Prevalence of inadequate intake of zinc
Women of reproductive age (15-49 years)	Vitamin A deficiency<br>Total goiter prevalence<br>Any anemia<br>Zinc deficiency<br>RBC folate deficiency<br>Serum folate deficiency<br>Vitamin B_{12} deficiency<br>Iodized salt coverage<br>Prevalence of inadequate intake of iron<br>Prevalence of inadequate intake of zinc<br>Prevalence of inadequate intake vitamin A
School age children (6 to 12 years)	Vitamin A deficiency<br>Total goiter prevalence<br>Any anemia<br>Iodine deficiency<br>Zinc deficiency
Adolescent girls (10 to 19 years)	Any anemia

For peer review only

Table 2S. Sample size determination and allocation

Region	Indicator used to estimate sample size	Number of EA	Number of HH	Expected number pre-school children (0-59 months)	Expected number of WRA (15-49 years)	Expected number school age children (6-12 years)	Expected number of adolescent girls (10-19 years)	Total population (2019 year projection)
Tigray	Any anemia	55	1,432	590	1,236	753	516	5,443,095
Afar	Stunting	51	1,328	539	1,096	695	406	1,901,863
Amhara	Stunting	61	1,585	619	1,253	843	531	21,842,548
Oromia	IDD	62	1,622	891	1,539	1,111	739	37,267,225
Somali	IDD	55	1,424	855	1,268	1,000	492	6,050,851
Benishangul-Gumuz	Stunting	49	1,282	555	1,127	732	475	1,126,656
SNNPR	Any anemia	59	1,528	818	1,492	1,000	692	15,763,484
Gambela	Any anemia	47	1,211	428	1,018	568	373	463,203
Harari	Any anemia	45	1,164	375	978	499	348	257,362
Addis Ababa	IDD/ TGR	54	1,411	413	1,274	405	262	3,685,684
Dire Dawa	Stunting	47	1,215	382	1,128	480	360	492,819
Sidama	Any anemia	54	1,395	747	1,363	914	632	4,322,685
Total sample size		639	16,596	7,213	14,772	9,001	5,824	**98,617,475**

For peer review only

Name of institute	Doc. number:	Version No: 3
**ETHIOPIAN PUBLIC HEALTH INSTITUTE**	EPHI/ TL/ FNL/TM/7.2.11	Copy no______
		Page 1 of 7
Document Title: **DETERMIANTION OF IODINE FROM SALT SAMPLE**	Effective date: November 20, 2020	

1. Purpose

This procedure used to determine the amount of iodine content in the form of potassium iodate (KIO_3) by iodometric titration from iodized salt.

2. Abbreviations:

**g**	gram	**KI**	Potassium Iodide
**ppm**	Parts Per million	**H_2SO_4**	sulfuric acid
**M**	Molarity	**KIO_3**	potassium iodate
**ml**	mili liter	**IDD**	Iodine deficiency disorder
**N**	Normality	**$Na_2S_2O_3.5H_2O$**	sodium thiosulfate penta hydrate

3. Principle:

Iodine released from potassium iodate by the action of sulphuric acid and the released iodine trapped with potassium iodide and titrate with sodium thiosulphate.

4. Material and methods

4.1 Reagents

- 0.005M $Na_2S_2O_3.5H_2O$
- 2N H_2SO_4
- 10% KI
- 1% Starch

4.2 Reagents preparation:

- **1%starch:** Dissolve 1 g of soluble starch in 100ml boiled distilled water heat the solution till starch dissolve completely.
- **10% KI;** Dissolve 10gm of KI in 100ml deionized water.

Prepared by: Addisu Legesse and Teshome Assefa	Authority: EPHI

NOTE: This is a CONTROLLED document for internal use only. Any documents appearing in paper form are not controlled and should be checked against the electronic version prior to use.

For peer review only

Name of institute<br>**ETHIOPIAN PUBLIC HEALTH INSTITUTE**	Doc. number:<br>EPHI/ TL/ FNL/TM/7.2.11	Version No: 3<br>Copy no_____<br>Page 2 of 7
Document Title: **DETERMIANTION OF IODINE FROM SALT SAMPLE**	Effective date: November 20, 2020	

- **2N H_2SO_4;** Slowly add 6 ml of concentrated H_2SO_4 to 90 ml of deionized water make the final volume 100 ml.
- **0.005M $Na_2S_2O_3$;** Dissolve 1.24gm of $Na_2S_2O_3.5H_2O$ in 1000 ml of deionized water

4.3 Reagents stability and storage:

- $Na_2S_2O3.5H_2O$ & 10% KI reagents store in a cool & dark place for six months.
- H_2SO_4 store at room temperature it stable indefinitely.
- Starch should be prepared daily.

5. Supplies and Equipment

- Balance (Four-beam pan): Sensitivity = 0.01g, Capacity = 410g
- Flask, volumetric, 1000mL, 100mL
- Measuring cylinder, 10mL, 100mL
- Beakers (Pyrex)
- Flasks, Erlenmeyer (conical) with stopper, 250mL
- Pipette, volumetric, 1mL, 5mL
- Burette w/straight stopcock 10mL
- Burette stand
- Laboratory safety glasses
- Parafilm, for covering beakers
- Glass bottles with stoppers, for reagents, 250mL
- Spatula Lab single blade 150mm SS length
- Dropper bottle, glass 25-60mL
- Hot plate

6. Sample

- Sample type: salt
- Amount required: 50-100g
- Transport and storage: At room temperature avoid exposure to direct sunlight

Prepared by: Addisu Legesse and Teshome Assefa	Authority: EPHI

NOTE: This is a CONTROLLED document for internal use only. Any documents appearing in paper form are not controlled and should be checked against the electronic version prior to use.

For peer review only

- Stability: At room temperature for 3 months.

7. Special Safety and Precautions

- While titrate the sample wear eye google.
- The reaction mixture should be kept in the dark before titration because a side reaction can occur when the solution is exposed to light that causes iodide ions to be oxidized to iodine.
- Inaccurate results may occur if starch solution is used while still warm.
- If starch indicator is added too early, a strong iodine-starch complex is formed, which reacts slowly, and gives falsely elevated results.
- The reaction should be performed at mild room temperature (<30 ^{0}C), since the iodine is volatile, and the indicator solution loses sensitivity when exposed to high temperatures.

8. Procedure

8.1 Weigh 10g of the salt sample into a 250mL Erlenmeyer flask with a stopper.

8.2 Add approximately 50 mL water, swirl to dissolve salt sample.

8.3 Add 1 ml 2N H_2SO_4

8.4 Add 5mL 10% KI. The solution should turn yellow if iodine is present.

8.5 Close the flask with stopper & put it in the dark place for 10 minutes in closed box (cupboard or drawer).

8.6 Rinse and fill burette with 0.005M $Na_2S_2O_3$, and adjust level to zero.

8.7 After 10 minutes take the flask out from drawer, and add some $Na_2S_2O_3$ from the titration burette Until the solution turns pale yellow.

8.8 Add approximately 2mL of starch indicator solution (the solution should turn dark purple) and continue titrating with 0.005M $Na_2S_2O_3$ until the solution becomes pink, and finally colorless.

8.9 Record the level of thiosulfate in the burette and convert to parts per million (ppm)

NOTE: This is a CONTROLLED document for internal use only. Any documents appearing in paper form are not controlled and should be checked against the electronic version prior to use.

For peer review only - http://bmjopen.bmj.com/site/about/guidelines.xhtml

Name of institute	Doc. number:	Version No: 3
**ETHIOPIAN PUBLIC HEALTH INSTITUTE**	EPHI/ TL/ FNL/TM/7.2.11	Copy no______
		Page 4 of 7
Document Title: **DETERMIANTION OF IODINE FROM SALT SAMPLE**	Effective date: November 20, 2020	

9. Quality control:

- **Control material**: KIO_3
- **Level of iodine**: 59.3-60.3ppm
- **Stability**: stable at room temperature for long period of time.
- **Frequency**: per batch

10. Quality control preparation: Prepare 0.0047M KIO_3 in 100ml. By weighing 0.10058g in 100ml deionized water. From this solution Pipette out 1 ml into conical flask and follow the procedure proceed from step 2 as described above.

11. Calculation

Iodine(ppm) **= 10.6* V $Na_2S_2O_3$(ml)**

Where: V$Na_2S_2O_3$: Volume of sodium thiosulphate takes to titrate iodine in salt

12. Result Interpretation

- **<5ppm** to indicate salt with no added iodine
- **5-14.9** ppm to indicate inadequately iodized salt
- **15-39.9** ppm to indicate salt is adequately iodized
- **>40** ppm of iodine is not recommended.

13. Other Records

- Data Log sheet
- QC chart

14. References

- De Maeyer, Lowenstein & Thilly, 1979; World Health Organization (WHO), United Nations Children Fund (UNICEF) & ICCIDD, 2007.
- AOAC 925.56 2016

Prepared by: Addisu Legesse and Teshome Assefa	Authority: EPHI

NOTE: This is a CONTROLLED document for internal use only. Any documents appearing in paper form are not controlled and should be checked against the electronic version prior to use.

For peer review only

15. Annex

Conversion chart for iodine in fortified salt in the form of KIO_3 (PPM)

Volume Thiosulphate (mL)	Iodine (ppm)	Volume Thiosulphate (mL)	Iodine (ppm)	Volume Thiosulphate (mL)	Iodine (ppm)	Volume Thiosulphate (mL)	Iodine (ppm)	Volume Thiosulphate (mL)	Iodine (ppm)	Volume Thiosulphate (mL)	Iodine (ppm)
0.1	1.1	2.0	21.2	3.9	41.3	5.8	61.5	7.7	81.6	9.6	101.8
0.2	2.1	2.1	22.2	4.0	42.4	5.9	62.5	7.8	82.7	9.7	102.8
0.3	3.2	2.2	23.3	4.1	43.5	6.0	63.6	7.9	83.4	9.8	103.9
0.4	4.2	2.3	24.4	4.2	44.5	6.1	64.7	8.0	84.8	9.9	104.9
0.5	5.3	2.4	25.4	4.3	45.6	6.2	65.7	8.1	85.9	10.0	106.0
0.6	6.4	2.5	26.5	4.4	46.4	6.3	66.8	8.2	86.9	10.1	107.1
0.7	7.4	2.6	27.6	4.5	47.7	6.4	67.8	8.3	88.0	10.2	108.1
0.8	8.5	2.7	28.6	4.6	48.8	6.5	68.9	8.4	89.0	10.3	109.2
0.9	9.4	2.8	29.7	4.7	49.8	6.6	70.0	8.5	90.1	10.4	110.2
1.0	10.6	2.9	30.7	4.8	50.9	6.7	71.0	8.6	91.2	10.5	111.3
1.1	11.7	3.0	31.8	4.9	51.9	6.8	72.1	8.7	92.2	10.6	112.4
1.2	12.2	3.1	32.9	5.0	53.0	6.9	73.1	8.8	93.3	10.7	113.4
1.3	13.8	3.2	33.9	5.1	54.1	7.0	74.2	8.9	94.3	10.8	114.5
1.4	14.8	3.3	35.0	5.2	55.1	7.1	75.3	9.0	95.4	10.9	115.5
1.5	15.9	3.4	36.0	5.3	56.2	7.2	76.3	9.1	96.5	11.0	116.6
1.6	17.0	3.5	37.1	5.4	57.2	7.3	77.4	9.2	97.5	11.1	117.7
1.7	18.0	3.6	38.2	5.5	58.3	7.4	78.4	9.3	98.6	11.2	118.7
1.8	19.1	3.7	39.2	5.6	59.4	7.5	79.5	9.4	99.7	11.3	119.8
1.9	20.1	3.8	40.3	5.7	60.4	7.6	80.6	9.5	100.7	11.4	120.8

For peer review only - http://bmjopen.bmj.com/site/about/guidelines.xhtml

Name of institute<br>ETHIOPIAN PUBLIC HEALTH INSTITUTE	Doc. number:<br>EPHI/ TL/ FNL/TM/7.2.11	Version No: 3<br>Copy no_____<br>Page 6 of 7
Document Title: **DETERMIANTION OF IODINE FROM SALT SAMPLE**	Effective date: November 20, 2020	

Name Signature

Prepared by: -------------------------- ----------------------------

Reviewed by: ------------------------- ----------------------------

Approved by: -------------------------- -----------------------------

(Director of Food science and Nutrition research directorate)

Declaration

I, the undersigned laboratory personnel, certify that I am conducting every steps of the procedures incorporated in this SOP after a prior reading.

**Name**	**Signature and Date**
……………………………..	……………………………..
……………………………..	……………………………..
……………………………..	……………………………..
……………………………..	……………………………..
……………………………..	……………………………..

Prepared by: Addisu Legesse and Teshome Assefa	Authority: EPHI

NOTE: This is a CONTROLLED document for internal use only. Any documents appearing in paper form are not controlled and should be checked against the electronic version prior to use.

For peer review only

Name of institute	Doc. number:	Version No: 3
**ETHIOPIAN PUBLIC HEALTH INSTITUTE**	EPHI/ TL/ FNL/TM/7.2.11	Copy no______
		Page 7 of 7
Document Title: **DETERMIANTION OF IODINE FROM SALT SAMPLE**	Effective date: November 20, 2020	

Document Change History

Revision No.	Date approved	Nature of revision
1	February, 2019	Initial release
2	November, 2020	Typographical adjustment<br>Document revision table is added

Prepared by: Addisu Legesse and Teshome Assefa	Authority: EPHI

NOTE: This is a CONTROLLED document for internal use only. Any documents appearing in paper form are not controlled and should be checked against the electronic version prior to use.

For peer review only

Determination of serum retinol using hexane as the serum sample extraction procedure

For peer review only

Contents

1. Reagents and materials 2
2. Instrument (HPLC) parameters – we have Shimadzu prominence HPLC. 2
3. Sample extraction procedure 2
4. Calculation 3
5. Reference 3

For peer review only

For peer review only - http://bmjopen.bmj.com/site/about/guidelines.xhtml

1. Reagents and materials

- Methanol (HPLC grade)
- Hexane (HPLC grade)
- Ethanol (HPLC grade/AR grade)
- Normal saline solution (0.9% NaCl)
- Retinol acetate (as internal standard)
- Retinol (as external standard)
- CRM (Certified Reference Material) for serum retinol
- 15 ml centrifuge tube
- Vortex mixer
- Centrifuge (up to 4000 rpm capacity)
- Centrifuge tube rack
- Micropipettes (10-100µl and 100-1000µl)
- Micropipettes tips for both types
- Stopwatch
- HPLC Vials (1ml)

2. Instrument (HPLC) parameters – we have Shimadzu prominence HPLC.

- Detector - SPD-10A UV/VIS at 325 nm
- Column – SUPELCOSIL™LC-NH_2-NP, 25cm × 4.6mm, 5µm
- Mobile phase – HPLC grade methanol
- Elution system – isocratic

3. Sample extraction procedure

- Add 200 µl serum sample to 15ml plastic test tube.
- Add an equal volume of 50 µg/dl retinol acetate and ethanol.
- Take 200 µl of a serious of standards (10, 20, 40, 60, 75 µg/dl) to the 15 ml centrifuge tube and add the same volume of retinol acetate and normal saline solution.
- Mix the standard and sample solutions with a vortex mixer
- Add 1ml of HPLC grade hexane and mix for 45 seconds
- Centrifuge the solution at 4000rpm for 7 minutes and transfer the supernatant to other test tubes
- The solution has to be extract twice by adding 1 ml hexane.

For peer review only - http://bmjopen.bmj.com/site/about/guidelines.xhtml

- Dry the hexane using nitrogen gas and reconstitute by 500µl HPLC grade methanol
- It has to be mix for 30 minutes using a vortex mixer and transfer to 1 ml HPLC vials
- Analyze the extracted solutions using reversed-phase HPLC by isocratic elution system, at the flow rate of 1.5 ml/min, and injection volume 30µl

N: B: - Extract and analyse the CRM using the sample extraction procedure.

4. Calculation

- Plot the calibration curve using area ratio of retinol (standard) and retinol acetate (internal standard) vs concentration ratio (retinol: retinol acetate).
- From the linear equation formula, Y = mx + b, the concentration of serum retinol can be calculated.

  Where Y – is area ratio (retinol: retinol acetate) of sample

  M – is slope and b – is Y intercept

  X – is the concentration of serum retinol in µg/dl

5. Reference

Quadro, Y.-K. K. and L. (2013). Reverse-Phase High-Performance Liquid Chromatography (HPLC) Analysis of Retinol and Retinyl Esters in Mouse Serum and Tissues. *Methods Mol Biol. 2010 ; 652: 263–275. Doi:10.1007/978-1-60327-325-1_15.*, (12), 1–10. https://doi.org/10.1007/978-1-60327-325-1

von Lucke, A., Russell, R. M., Stephensen, C. B., Gannon, B. M., Craft, N. E., Haskell, M. J., … Raiten, D. J. (2016). Biomarkers of Nutrition for Development (BOND)—Vitamin A Review. *The Journal of Nutrition*, 1816s-1848s. https://doi.org/10.3945/jn.115.229708.FIGURE

Prepared by: Nahom Tefera (September 2020)

Reviewed by: Meseret W/yohannis

Approved by: Dr. Masresha Tessema

For peer review only - http://bmjopen.bmj.com/site/about/guidelines.xhtml

	Name of institute<br>**ETHIOPIAN PUBLIC HEALTH INSTITUTE**	Doc. number:<br>**EPHI/TL/FNL/TM7.2-17**	Version No: 3<br>Copy No. _____<br>Page 1 of 5
Document Title: DETERMINATION OF IODINE IN URINE		Effective date: December, 2020	

1 Scope

This test method is applicable for the determination of iodine from a urine sample.

2 Purpose

This procedure provides methods to control the amount of iodine and it's toxicity by ammonium persulfate digestion with spectrophotometric detection of the Sandell-Kolthoff Reaction method from urine samples.

3 Principle

Urine is digested with ammonium persulfate. Iodide is the catalyst in the reduction of ceric ammonium sulfate (yellow) to cerous form (colorless) and is detected by the rate of color disappearance (Sandell-Kolthoff reaction).

4 Chemicals and Apparatus

a) Chemicals

- Ammonium persulphate
- Arsenic trioxide
- Ceric ammonium sulphate
- Potassium iodate
- Deionized water
- Sodium chloride

b) Apparatus

- Hot plate
- Oven
- UV-Vis spectrophotometer
- Volumetric flasks (100 – 2000 ml)
- Beakers (100- 1000 ml)

For peer review only

- Micropipette (100 – 1000 µl)
- Pipette (5 – 10 ml)
- Vortex mixer
- Measuring cylinder (100 – 1000 ml)
- Glass test tubes (13*100 mm)
- Analytical balance (nearest to the 0.0001 g)
- Erlenmeyer flask (2000 ml)

5 Reagents preparation

a) 1 M Ammonium persulphate

- Dissolve 114.1 g $H_2 N_2 O_8 S_2$ in deionized water and makeup to 500 ml with H_2O. Store away from light. Stable for at least one month.

b) 5 N H_2SO_4

- Slowly add 139 ml concentrated (36 N) H_2SO_4 to about 700 ml deionized water (careful - this generates heat!). When cool, adjust with deionized water to a final volume of 1 liter.

c) 3.5 N H_2SO_4

- Slowly add 97 ml concentrated (36 N) H_2SO_4 to about 800 ml deionized water (careful - this generates heat!), and when cool, adjusting with deionized water to a final volume of 1 liter

d) Ceric ammonium sulphate solution

- Dissolve 48 g ceric ammonium sulphate in 1 liter 3.5 N H_2SO_4. Store in a dark bottle away from light at room temperature. The solution is stable for months.

e) Arsenious acid solution

- In a 2000 ml Erlenmeyer flask, place 20 g As_2O_3 and 50 g NaCl, then slowly add 400 ml 5 N H_2SO_4. Add water to about 1 liter, heat gently to dissolve, cool to room temperature, dilute with water to 2 liters, filter, store in a dark bottle away from light at room temperature. The solution is stable for months.

For peer review only

Name of institute		Doc. number:	Version No: 3
	**ETHIOPIAN PUBLIC HEALTH INSTITUTE**	**EPHI/TL/FNL/TM7.2-17**	Copy No. ______
			Page 3 of 5
Document Title: DETERMINATION OF IODINE IN URINE		Effective date: December, 2020	

6 Standards preparation

a) Stock standard solution (1 mg/ml)

- Dissolve 0.1685 g KIO_3 in deionized water to a final volume of 100 ml (1.68 g KIO_3 contains 1.0 g iodine). KIO_3 is preferred over KI because it is more stable. It may be more convenient to make a more concentrated solution, e.g., 10 or 100 mg iodine/ml.

b) Intermediate standard solution (1 µg/ml)

- Dilute 100 µl stock iodine standard (1mg/ml) to 100 ml deionized water. Store in a dark bottle. The solution is stable for months. Useful standards are 20, 50, 100, 150, 200, and 300 µg/l.

c) Serial standard dilutions

- From intermediate standards, prepare 50, 100, 150, 200, and 250 µg/L useful standards for calibration curve purposes.

Note: All standard solutions should store in a dark place. The solutions are stable for months.

7 Procedures

- Mix urine to suspend sediment using a vortex mixer.
- Pipette 250 µl of each urine sample into a 13 x 100 mm test tube.
- Pipette 250 µl each iodine serial standards also into a test tube.
- Add 1 ml 1.0 M ammonium persulfate solution to each tube.
- Vortex all tubes using a vortex mixer
- Heat all tubes for 60 minutes at 100° C in the oven.
- Cool tubes to room temperature in a fume hood.
- Add 2.5 ml arsenious acid solution. Mix by a vortex. Let stand for 15 minutes.
- Add 300 µl of ceric ammonium sulfate solution to each tube (quickly mixing) at 15-30 second intervals between successive tubes. A stopwatch should be used for this. With practice, a 20-second interval is convenient.

For peer review only

Name of institute		Doc. number:	Version No: 3
	**ETHIOPIAN PUBLIC HEALTH INSTITUTE**	**EPHI/TL/FNL/TM7.2-17**	Copy No. _____
			Page 4 of 5
Document Title:	DETERMINATION OF IODINE IN URINE	Effective date: December, 2020	

- Allow sitting at room temperature. Exactly 30 minutes after the addition of ceric ammonium sulfate to the first tube, read its absorbance by using UV-Vis spectrophotometer at 420 nm. Read successive tubes at the same interval as when adding the ceric ammonium sulfate.

8 Calculation

- Construct a standard curve on graph paper by plotting log absorbance of each concentration versus iodine concentration of each standard.
- Urinary Iodine in µg/l = ((log A-b)/(m)) * 10
  Where log A is log absorbance of sample, b is Y-intercept of the calibration graph, m is the slope of the graph, and 10 is used as a conversion factor when we prepare serials of standards in µg/dl (in this case we multiply the final concentration (µg/ dl) by 10 to get the concentration in µg /l).

9 Quality control and safety precautions

- In each batch, the urine quality control sample (CRM or in-house prepared) should run together with sample and standards.
- While working the urine analysis and reagent preparation wear gloves, lab coat, eye goggle, lab shoe, and mouth cover.

Remember: During the analysis of iodine from urine, you should ensure that the laboratory working environment should free from salt samples (especially iodized salt samples) to avoid contamination.

Limitations: If the urine sample is analyzed at a high temperature the loss of iodine occurred and the method has to detect very low iodine concentration in urine.

10 References

- ICCIDD, UNICEF, WHO. Dunn JT et al. Methods for measuring iodine in urine. The Netherlands, ICCIDD, 1993.

For peer review only

	Name of institute<br>**ETHIOPIAN PUBLIC HEALTH INSTITUTE**	Doc. number:<br>**EPHI/TL/FNL/TM7.2-17**	Version No: 3<br>Copy No. _____<br>Page 5 of 5
Document Title: DETERMINATION OF IODINE IN URINE		Effective date: December, 2020	

Prepared by	Signature	Date
Prepared by: Nahom Tefera	___________	_________________
Reviewed by: Meseret W/yohannes	___________	__________________
Approved by: Dr. Masresha Tessema	___________	__________________

For peer review only

0003333795190c501V10.0

AAGP2

cobas®

Tina-quant α1-Acid Glycoprotein Gen.2

Order information

REF	CONTENT		Analyzer(s) on which **cobas c** pack(s) can be used
**03333795** 190	Tina-quant α1-Acid Glycoprotein Gen.2 100 tests	System-ID 07 6758 1	Roche/Hitachi **cobas c** 311, **cobas c** 501/502
**11355279** 216	Calibrator f.a.s. Proteins (5 x 1 mL)	Code 656	
**11355279** 160	Calibrator f.a.s. Proteins (5 x 1 mL, for USA)	Code 656	
**10557897** 122	Precinorm Protein (3 x 1 mL)	Code 302	
**10557897** 160	Precinorm Protein (3 x 1 mL, for USA)	Code 302	
**11333127** 122	Precipath Protein (3 x 1 mL)	Code 303	
**11333127** 160	Precipath Protein (3 x 1 mL, for USA)	Code 303	
**05117003** 190	PreciControl ClinChem Multi 1 (20 x 5 mL)	Code 391	
**05947626** 190	PreciControl ClinChem Multi 1 (4 x 5 mL)	Code 391	
**05947626** 160	PreciControl ClinChem Multi 1 (4 x 5 mL, for USA)	Code 391	
**05117216** 190	PreciControl ClinChem Multi 2 (20 x 5 mL)	Code 392	
**05947774** 190	PreciControl ClinChem Multi 2 (4 x 5 mL)	Code 392	
**05947774** 160	PreciControl ClinChem Multi 2 (4 x 5 mL, for USA)	Code 392	
**04489357** 190	Diluent NaCl 9 % (50 mL)	System-ID 07 6869 3	

English

System information

For **cobas c** 311/501 analyzers:

AAGP2: ACN 229

For **cobas c** 502 analyzer:

AAGP2: ACN 8229

Intended use

In vitro test for the quantitative determination of α_1-acid glycoprotein in human serum and plasma on Roche/Hitachi **cobas c** systems.

Summary[1,2,3,4,5]

α_1-Acid glycoprotein is synthesized in hepatocytes and consists of a polypeptide chain having 5 carbohydrate chains N-glycosidically bonded to it (molar mass 41000 daltons). Structurally, it belongs to the lipocalin superfamily of secretory proteins (such as α_1-microglobulin and retinol-binding protein). α_1-Acid glycoprotein promotes fibroblast growth and interacts with collagen.

It is a sensitive acute phase reactant whose concentration can increase by a factor of 3 within 24-48 hours when inflammation occurs. α_1-Acid glycoprotein can also be used to differentiate between acute phase reactions (elevated serum level) and estrogen effects (normal or decreased serum level) whereas the serum level of other positive reactants such as ceruloplasmin and haptoglobin increases during such reactions. Along with haptoglobin it is perhaps the best protein for identifying slight in vivo hemolysis. An increased α_1-acid glycoprotein level and normal haptoglobin values indicate an acute phase reaction with concomitant slight in vivo hemolysis. Moderate and isolated increases occur when glomerular filtration is inhibited in the early stages of uremia. The determination is used in the assessment of the activity of acute and recurring inflammations as well as of tumors with cell necrosis.

Various assay methods for α_1-acid glycoprotein determination are available such as kinetic nephelometry, radial immunodiffusion (RID) and turbidimetry. The Roche α_1-acid glycoprotein assay is based on the principle of immunological agglutination.

Test principle[2]

Immunoturbidimetric assay.

Anti-α_1-acid glycoprotein antibodies react with antigen in the sample to form an antigen/antibody complex. Following agglutination, this is measured turbidimetrically.

Reagents - working solutions

R1 TRIS buffer: 50 mmol/L, pH 8.0; NaCl: 300 mmol/L; PEG: 7 %; preservative; stabilizer

R2 Polyclonal anti-human α_1-acid glycoprotein antibody (goat): dependent on titer; TRIS buffer: 13 mmol/L, pH 7.5; NaCl: 198 mmol/L; preservative

R1 is in position B and R2 is in position C.

Precautions and warnings

For in vitro diagnostic use.
Exercise the normal precautions required for handling all laboratory reagents.
Disposal of all waste material should be in accordance with local guidelines.
Safety data sheet available for professional user on request.

For USA: Caution: Federal law restricts this device to sale by or on the order of a physician.

Reagent handling

Ready for use

Storage and stability

AAGP2

Shelf life at 2-8 °C:	See expiration date on **cobas c** pack label.
On-board in use and refrigerated on the analyzer:	12 weeks

Diluent NaCl 9 %

Shelf life at 2-8 °C:	See expiration date on **cobas c** pack label.
On-board in use and refrigerated on the analyzer:	12 weeks

Specimen collection and preparation

For specimen collection and preparation only use suitable tubes or collection containers.

Only the specimens listed below were tested and found acceptable.
Serum
Plasma: Li-heparin and K_2-EDTA plasma.

The sample types listed were tested with a selection of sample collection tubes that were commercially available at the time of testing, i.e. not all available tubes of all manufacturers were tested. Sample collection systems from various manufacturers may contain differing materials which could affect the test results in some cases. When processing samples in primary tubes (sample collection systems), follow the instructions of the tube manufacturer.

Centrifuge samples containing precipitates before performing the assay.

Stability:[6]	< 72 hours at 4 °C
	6 months at -20 °C

For peer review only - http://bmjopen.bmj.com/site/about/guidelines.xhtml

0003333795190c501V10.0

AAGP2

cobas®

Tina-quant α1-Acid Glycoprotein Gen.2

Materials provided

See “Reagents – working solutions” section for reagents.

Materials required (but not provided)

- See “Order information” section
- General laboratory equipment

Assay

For optimum performance of the assay follow the directions given in this document for the analyzer concerned. Refer to the appropriate operator’s manual for analyzer-specific assay instructions.

The performance of applications not validated by Roche is not warranted and must be defined by the user.

Application for serum and plasma

cobas c 311 **test definition**

Assay type	2-Point End		
Reaction time/Assay points	10/6-32		
Wavelength (sub/main)	660/340 nm		
Reaction direction	Increase		
Units	g/L (µmol/L, mg/dL)		
Reagent pipetting		Diluent (H_2O)	
R1	120 µL	–	
R2	40 µL	–	
Sample volumes	*Sample*	*Sample dilution*	
		Sample	*Diluent (NaCl)*
Normal	12 µL	9 µL	180 µL
Decreased	12 µL	4 µL	122 µL
Increased	12 µL	9 µL	180 µL

cobas c 501 **test definition**

Assay type	2-Point End		
Reaction time/Assay points	10/10-48		
Wavelength (sub/main)	660/340 nm		
Reaction direction	Increase		
Units	g/L (µmol/L, mg/dL)		
Reagent pipetting		Diluent (H_2O)	
R1	120 µL	–	
R2	40 µL	–	
Sample volumes	*Sample*	*Sample dilution*	
		Sample	*Diluent (NaCl)*
Normal	12 µL	9 µL	180 µL
Decreased	12 µL	4 µL	122 µL
Increased	12 µL	9 µL	180 µL

cobas c 502 **test definition**

Assay type	2-Point End		
Reaction time/Assay points	10/10-48		
Wavelength (sub/main)	660/340 nm		
Reaction direction	Increase		
Units	g/L (µmol/L, mg/dL)		
Reagent pipetting		Diluent (H_2O)	
R1	120 µL	–	
R2	40 µL	–	
Sample volumes	*Sample*	*Sample dilution*	
		Sample	*Diluent (NaCl)*
Normal	12 µL	9 µL	180 µL
Decreased	12 µL	4 µL	122 µL
Increased	12 µL	18 µL	180 µL

Calibration

Calibrators	S1: H_2O
	S2-S6: C.f.a.s. Proteins
	Multiply the lot-specific C.f.a.s. Proteins calibrator value by the factors below to determine the standard concentrations for the 6-point calibration curve:
	S2: 0.140 S5: 1.40
	S3: 0.280 S6: 2.81
	S4: 0.700
Calibration mode	RCM2
Calibration frequency	Full calibration<br>- after reagent lot change<br>- as required following quality control procedures

Calibration interval may be extended based on acceptable verification of calibration by the laboratory.

Traceability: This method has been standardized against the reference preparation of the IRMM (Institute for Reference Materials and Measurements) BCR470/CRM470 (RPPHS - Reference Preparation for Proteins in Human Serum).[7]

Quality control

For quality control, use control materials as listed in the "Order information" section.

In addition, other suitable control material can be used.

The control intervals and limits should be adapted to each laboratory’s individual requirements. Values obtained should fall within the defined limits. Each laboratory should establish corrective measures to be taken if values fall outside the defined limits.

Follow the applicable government regulations and local guidelines for quality control.

Calculation

Roche/Hitachi **cobas c** systems automatically calculate the analyte concentration of each sample.

Conversion factors:	g/L x 25 = µmol/L	mg/dL x 0.01 = g/L
	mg/dL x 0.25 = µmol/L	g/L x 100 = mg/dL

Limitations - interference

Criterion: Recovery within ± 10 % of initial value at an α_1-acid glycoprotein concentration of 0.5 g/L (12.5 µmol/L, 50 mg/dL).

Icterus:[8] No significant interference up to an I index of 60 for conjugated and unconjugated bilirubin (approximate conjugated and unconjugated bilirubin concentration: 1026 µmol/L or 60 mg/dL).

Hemolysis:[8] No significant interference up to an H index of 1000 (approximate hemoglobin concentration: 621 µmol/L or 1000 mg/dL).

Lipemia (Intralipid):[8] No significant interference up to an L index of 650. There is poor correlation between the L index (corresponds to turbidity) and triglycerides concentration.

Rheumatoid factors up to 1200 IU/mL do not interfere.

High dose hook-effect: No false result occurs up to an α_1-acid glycoprotein concentration of 11 g/L (275 µmol/L, 1100 mg/dL).

Drugs: No interference was found at therapeutic concentrations using common drug panels.[9, 10]

In very rare cases, gammopathy, in particular type IgM (Waldenström’s macroglobulinemia), may cause unreliable results.[11]

For peer review only

For peer review only - http://bmjopen.bmj.com/site/about/guidelines.xhtml

0003333795190c501V10.0

AAGP2

Tina-quant α1-Acid Glycoprotein Gen.2

cobas®

For diagnostic purposes, the results should always be assessed in conjunction with the patient's medical history, clinical examination and other findings.

ACTION REQUIRED
Special Wash Programming: The use of special wash steps is mandatory when certain test combinations are run together on Roche/Hitachi **cobas c** systems. The latest version of the carry-over evasion list can be found with the NaOHD-SMS-SmpCln1+2-SCCS Method Sheets. For further instructions refer to the operator's manual. **cobas c** 502 analyzer: All special wash programming necessary for avoiding carry-over is available via the **cobas** link, manual input is not required.

Where required, special wash/carry-over evasion programming must be implemented prior to reporting results with this test.

Limits and ranges

Measuring range

0.1-4.0 g/L (2.5-100 µmol/L, 10-400 mg/dL)

Determine samples having higher concentrations via the rerun function. Dilution of samples via the rerun function is a 1:1.5 dilution. Results from samples diluted using the rerun function are automatically multiplied by a factor of 1.5.

Lower limits of measurement

Lower detection limit of the test

0.1 g/L (2.5 µmol/L, 10 mg/dL)

The lower detection limit represents the lowest measurable analyte level that can be distinguished from zero. It is calculated as the value lying 3 standard deviations above that of the lowest standard (standard 1 + 3 SD, repeatability, n = 21).

Expected values[12]

0.5-1.2 g/L (12.5-30 µmol/L, 50-120 mg/dL)

Each laboratory should investigate the transferability of the expected values to its own patient population and if necessary determine its own reference ranges.

Specific performance data

Representative performance data on the analyzers are given below. Results obtained in individual laboratories may differ.

Precision

Precision was determined using human samples and controls in an internal protocol with repeatability (n = 21) and intermediate precision (3 aliquots per run, 1 run per day, 21 days). The following results were obtained:

Repeatability	*Mean*	*SD*	*CV*
	g/L (µmol/L, mg/dL)	*g/L (µmol/L, mg/dL)*	*%*
Precinorm Protein	0.724 (18.1, 72.4)	0.00 (0.0, 0.0)	0.6
Precipath Protein	1.21 (30.3, 121)	0.01 (0.3, 1)	0.5
Human serum 1	0.642 (16.1, 64.2)	0.00 (0.0, 0.0)	0.7
Human serum 2	1.07 (26.8, 107)	0.01 (0.3, 1)	0.7

Intermediate precision	*Mean*	*SD*	*CV*
	g/L (µmol/L, mg/dL)	*g/L (µmol/L, mg/dL)*	*%*
Precinorm Protein	0.710 (17.8, 71.0)	0.007 (0.2, 1.0)	0.9
Precipath Protein	1.19 (30.0, 119)	0.01 (0.3, 1)	0.9
Human serum 3	0.660 (16.5, 66.0)	0.010 (0.3, 1.0)	1.5
Human serum 4	1.21 (30.3, 121)	0.02 (0.5, 2)	1.5

Method comparison

α_1-Acid glycoprotein values for human serum and plasma samples obtained on a Roche/Hitachi **cobas c** 501 analyzer (y) were compared with those determined using the corresponding reagent on a Roche/Hitachi 917 analyzer (x).

Sample size (n) = 119

Passing/Bablok[13]	Linear regression
y = 1.012x - 0.070 g/L	y = 0.998x - 0.056 g/L
τ = 0.973	r = 0.999

The sample concentrations were between 0.489 and 3.25 g/L (12.2 and 81.3 µmol/L, 48.9 and 325 mg/dL).

References

1 Schmid K. α1-Acid glycoprotein. In: The Plasma Proteins, 2nd ed. Putnam FW, ed. New York: Academic Press 1975;183-228.

2 Greiling H, Gressner AM, eds. Lehrbuch der Klinischen Chemie und Pathobiochemie, 3rd ed. Stuttgart/New York: Schattauer Verlag 1995:236.

3 Tietz NW, ed. Clinical Guide to Laboratory Tests, 3rd ed. Philadelphia, PA: WB Saunders Company 1995;66-67.

4 Ganrot K. Plasma protein pattern in acute infectious disease. Scand J Clin Lab Invest 1974;34:75-81.

5 Lievens M, Bienvenu J, Buitrago JMG, et al. Evaluation of four new Tina-quant assays for determination of α1-acid glycoprotein, α1-antitrypsin, haptoglobin and prealbumin. Clin Lab 1996;42:515-520.

6 Wu AHB, ed. Tietz Clinical Guide to Laboratory Tests, 4th ed. St. Louis (MO): Saunders Elsevier 2006:42.

7 Baudner S, Bienvenu J, Blirup-Jensen S, et al. The certification of a matrix reference material for immunochemical measurement of 14 human serum proteins CRM470. Report EUR 15243 EN 1993;1-186.

8 Glick MR, Ryder KW, Jackson SA. Graphical Comparisons of Interferences in Clinical Chemistry Instrumentation. Clin Chem 1986;32:470-475.

9 Breuer J. Report on the Symposium "Drug effects in Clinical Chemistry Methods". Eur J Clin Chem Clin Biochem 1996;34:385-386.

10 Sonntag O, Scholer A. Drug interference in clinical chemistry: recommendation of drugs and their concentrations to be used in drug interference studies. Ann Clin Biochem 2001;38:376-385.

11 Bakker AJ, Mücke M. Gammopathy interference in clinical chemistry assays: mechanisms, detection and prevention. Clin Chem Lab Med 2007;45(9):1240-1243.

12 Dati F, Schumann G, Thomas L, et al. Consensus of a group of professional societies and diagnostic companies on guidelines for interim reference ranges for 14 proteins in serum based on the standardization against the IFCC/BCR/CAP reference material (CRM 470). Eur J Clin Chem Clin Biochem 1996;34:517-520.

13 Bablok W, Passing H, Bender R, et al. A general regression procedure for method transformation. Application of linear regression procedures for method comparison studies in clinical chemistry, Part III. J Clin Chem Clin Biochem 1988 Nov;26(11):783-790.

A point (period/stop) is always used in this Method Sheet as the decimal separator to mark the border between the integral and the fractional parts of a decimal numeral. Separators for thousands are not used.

Symbols

Roche Diagnostics uses the following symbols and signs in addition to those listed in the ISO 15223-1 standard (for USA: see https://usdiagnostics.roche.com for definition of symbols used):

CONTENT	Contents of kit
	Volume after reconstitution or mixing
GTIN	Global Trade Item Number

For peer review only - http://bmjopen.bmj.com/site/about/guidelines.xhtml

0003333795190c501V10.0

AAGP2

Tina-quant α1-Acid Glycoprotein Gen.2

cobas®

FOR US CUSTOMERS ONLY: LIMITED WARRANTY

Roche Diagnostics warrants that this product will meet the specifications stated in the labeling when used in accordance with such labeling and will be free from defects in material and workmanship until the expiration date printed on the label. THIS LIMITED WARRANTY IS IN LIEU OF ANY OTHER WARRANTY, EXPRESS OR IMPLIED, INCLUDING ANY IMPLIED WARRANTY OF MERCHANTABILITY OR FITNESS FOR PARTICULAR PURPOSE. IN NO EVENT SHALL ROCHE DIAGNOSTICS BE LIABLE FOR INCIDENTAL, INDIRECT, SPECIAL OR CONSEQUENTIAL DAMAGES.

COBAS, COBAS C, PRECICONTROL, PRECINORM, PRECIPATH and TINA-QUANT are trademarks of Roche.

All other product names and trademarks are the property of their respective owners.

Additions, deletions or changes are indicated by a change bar in the margin.

© 2017, Roche Diagnostics

CE

Roche Diagnostics GmbH, Sandhofer Strasse 116, D-68305 Mannheim
www.roche.com

Roche

Distribution in USA by:
Roche Diagnostics, Indianapolis, IN
US Customer Technical Support 1-800-428-2336

For peer review only

For peer review only - http://bmjopen.bmj.com/site/about/guidelines.xhtml

ms_05894913190V7.0

Vitamin D total

cobas®

25-Hydroxyvitamin D

REF	Σ	SYSTEM
**05894913** 190	100	Elecsys 2010<br>MODULAR ANALYTICS E170<br>**cobas e** 411<br>**cobas e** 601<br>**cobas e** 602

English

Intended use

This assay is intended for the quantitative determination of total 25-hydroxyvitamin D in human serum and plasma. This assay is to be used as an aid in the assessment of vitamin D sufficiency.

The electrochemiluminescence binding assay is intended for use on Elecsys and **cobas e** immunoassay analyzers.

Summary

Vitamin D is a fat-soluble steroid hormone precursor that is mainly produced in the skin by exposure to sunlight. Vitamin D is biologically inert and must undergo two successive hydroxylations in the liver and kidney to become the biologically active 1,25-dihydroxyvitamin D.[1]

The two most important forms of vitamin D are vitamin D_3 (cholecalciferol) and vitamin D_2 (ergocalciferol). In contrast to vitamin D_3, the human body cannot produce vitamin D_2 which is taken up with fortified food or given by supplements. In human plasma vitamin D_3 and D_2 are bound to the vitamin D binding protein and transported to the liver where both are hydroxylated to form vitamin D (25-OH), i.e. 25-hydroxyvitamin D. It is commonly agreed that vitamin D (25-OH) is the metabolite to determine the overall vitamin D status as it is the major storage form of vitamin D in the human body. This primary circulating form of vitamin D is biologically inactive with levels approximately 1000-fold greater than the circulating 1,25-dihydroxyvitamin D. The half-life of circulating vitamin D (25-OH) is 2-3 weeks.

Most of the vitamin D (25-OH), measurable in serum, is vitamin D_3 (25-OH) whereas vitamin D_2 (25-OH) reaches measurable levels only in patients taking vitamin D_2 supplements.[2,3,4] Vitamin D_2 is considered to be less effective.[5]

Vitamin D is essential for bone health. In children, severe deficiency leads to bone-malformation, known as rickets. Milder degrees of insufficiency are believed to cause reduced efficiency in the utilization of dietary calcium.[6] Vitamin D deficiency causes muscle weakness; in elderly, the risk of falling has been attributed to the effect of vitamin D on muscle function.[7] Vitamin D deficiency is a common cause of secondary hyperparathyroidism.[8,9] Elevations of PTH levels, especially in elderly vitamin D deficient adults can result in osteomalacia, increased bone turnover, reduced bone mass and risk of bone fractures.[10] Low vitamin D (25-OH) concentrations are also associated with lower bone mineral density.[11] In conjunction with other clinical data, the results may be used as an aid in the assessment of bone metabolism.

So far, vitamin D has been shown to affect expression of over 200 different genes. Insufficiency has been linked to diabetes, different forms of cancer, cardiovascular disease, autoimmune diseases and innate immunity.[2]

The Elecsys Vitamin D total assay employs a vitamin D binding protein (VDBP) as capture protein to bind vitamin D_3 (25-OH) and vitamin D_2 (25-OH).

Test principle

Competition principle. Total duration of assay: 27 minutes.

- 1st incubation: By incubating the sample (15 µL) with pretreatment reagent 1 and 2, bound vitamin D (25-OH) is released from the vitamin D binding protein.
- 2nd incubation: By incubating the pretreated sample with the ruthenium labeled vitamin D binding protein, a complex between the vitamin D (25-OH) and the ruthenylated vitamin D binding protein is formed.
- 3rd incubation: After addition of streptavidin-coated microparticles and vitamin D (25-OH) labeled with biotin, unbound ruthenium labeled vitamin D binding proteins become occupied. A complex consisting of the ruthenylated vitamin D binding protein and the biotinylated vitamin D (25-OH) is formed and becomes bound to the solid phase via interaction of biotin and streptavidin.
- The reaction mixture is aspirated into the measuring cell where the microparticles are magnetically captured onto the surface of the electrode. Unbound substances are then removed with ProCell/ProCell M. Application of a voltage to the electrode then induces chemiluminescent emission which is measured by a photomultiplier.
- Results are determined via a calibration curve which is instrument-specifically generated by 2-point calibration and a master curve provided via the reagent barcode.

Reagents - working solutions

The reagent rackpack (M, R1, R2) and the pretreatment reagents (PT1, PT2) are labeled as VITD-T.

PT1 Pretreatment reagent 1 (white cap), 1 bottle, 4 mL:
Dithiothreitol 1 g/L, pH 5.5.

PT2 Pretreatment reagent 2 (gray cap), 1 bottle, 4 mL:
Sodium hydroxide 55 g/L.

M Streptavidin-coated microparticles (transparent cap), 1 bottle, 6.5 mL:
Streptavidin-coated microparticles 0.72 mg/mL; preservative.

R1 Vitamin D binding protein-BPRu (gray cap), 1 bottle, 9 mL:
Ruthenium labeled vitamin D binding protein 150 µg/L; bis-tris propane buffer 200 mmol/L; albumin (human) 25 g/L; pH 7.5; preservative.

R2 25-hydroxyvitamin D~biotin (black cap), 1 bottle, 8.5 mL:
Biotinylated vitamin D (25-OH) 14 µg/L; bis-tris propane buffer 200 mmol/L; pH 8.6; preservative.

Precautions and warnings

For in vitro diagnostic use.
Exercise the normal precautions required for handling all laboratory reagents.
Disposal of all waste material should be in accordance with local guidelines.
Safety data sheet available for professional user on request.

This kit contains components classified as follows in accordance with the Regulation (EC) No. 1272/2008:

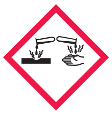

Danger

H290 May be corrosive to metals.

H314 Causes severe skin burns and eye damage.

Prevention:

P280 Wear protective gloves/ protective clothing/ eye protection/ face protection.

Response:

For peer review only - http://bmjopen.bmj.com/site/about/guidelines.xhtml

ms_05894913190V7.0

Vitamin D total

cobas®

25-Hydroxyvitamin D

P303 + P361 + P353	IF ON SKIN (or hair): Remove/Take off immediately all contaminated clothing. Rinse skin with water/shower.
P304 + P340 + P310	IF INHALED: Remove person to fresh air and keep comfortable for breathing. Immediately call a POISON CENTER or doctor/physician.
P305 + P351 + P338 + P310	IF IN EYES: Rinse cautiously with water for several minutes. Remove contact lenses, if present and easy to do. Continue rinsing. Immediately call a POISON CENTER or doctor/ physician.

Product safety labeling primarily follows EU GHS guidance.

Contact phone: all countries: +49-621-7590

All human material should be considered potentially infectious. All products derived from human blood are prepared exclusively from the blood of donors tested individually and shown to be free from HBsAg and antibodies to HCV and HIV. The testing methods applied were FDA-approved or cleared in compliance with the European Directive 98/79/EC, Annex II, List A.

However, as no testing method can rule out the potential risk of infection with absolute certainty, the material should be handled with the same level of care as a patient specimen. In the event of exposure, the directives of the responsible health authorities should be followed.[12,13]

Avoid foam formation in all reagents and sample types (specimens, calibrators and controls).

Reagent handling

The reagents in the kit have been assembled into a ready-for-use unit that cannot be separated.

All information required for correct operation is read in from the respective reagent barcodes.

Storage and stability

Store at 2-8 °C.

Do not freeze.

Store the Elecsys reagent kit **upright** in order to ensure complete availability of the microparticles during automatic mixing prior to use.

Stability:	
unopened at 2-8 °C	up to the stated expiration date
after opening at 2-8 °C	56 days (8 weeks)
on Elecsys 2010 and **cobas e** 411	21 days (3 weeks)
on MODULAR ANALYTICS E170, **cobas e** 601 and **cobas e** 602	28 days (4 weeks)

Specimen collection and preparation

Only the specimens listed below were tested and found acceptable.

Serum collected using standard sampling tubes or tubes containing separating gel.

Li-heparin, K_2- and K_3-EDTA plasma as well as Li-heparin plasma tubes containing separating gel.

Criterion: Method comparison serum versus plasma, slope 0.9-1.1 + intercept within < ± 2 x LoB + coefficient of correlation > 0.9.

Serum, Li-heparin, K_2- and K_3-EDTA plasma: Vitamin D (25-OH) is stable for 8 hours at 18-25 °C, 4 days at 2-8 °C, 24 weeks at -20 °C.

The stability of vitamin D (25-OH) found with the Elecsys Vitamin D total assay is in line with earlier studies using a vitamin D binding protein assay and mass spectrometry.[14]

The sample types listed were tested with a selection of sample collection tubes that were commercially available at the time of testing, i.e. not all available tubes of all manufacturers were tested. Sample collection systems from various manufacturers may contain differing materials which could affect the test results in some cases. When processing samples in primary tubes (sample collection systems), follow the instructions of the tube manufacturer.

Centrifuge samples containing precipitates before performing the assay.

Do not use heat-inactivated samples.

Do not use samples and controls stabilized with azide.

Ensure the samples, calibrators and controls are at 20-25 °C prior to measurement.

Due to possible evaporation effects, samples, calibrators and controls on the analyzers should be analyzed/measured within 2 hours.

Materials provided

See "Reagents – working solutions" section for reagents.

Materials required (but not provided)

- REF 05894921190, Vitamin D total CalSet, for 4 x 1 mL
- REF 05618860190, PreciControl Varia, for 2 x 3 mL each of PreciControl Varia 1 and 2
- REF 11732277122, Diluent Universal, 2 x 16 mL sample diluent or
  REF 03183971122, Diluent Universal, 2 x 36 mL sample diluent
- General laboratory equipment
- Elecsys 2010, MODULAR ANALYTICS E170 or **cobas e** analyzer

Accessories for Elecsys 2010 and **cobas e** 411 analyzers:

- REF 11662988122, ProCell, 6 x 380 mL system buffer
- REF 11662970122, CleanCell, 6 x 380 mL measuring cell cleaning solution
- REF 11930346122, Elecsys SysWash, 1 x 500 mL washwater additive
- REF 11933159001, Adapter for SysClean
- REF 11706802001, Elecsys 2010 AssayCup, 60 x 60 reaction vessels
- REF 11706799001, Elecsys 2010 AssayTip, 30 x 120 pipette tips

Accessories for MODULAR ANALYTICS E170, **cobas e** 601 and **cobas e** 602 analyzers:

- REF 04880340190, ProCell M, 2 x 2 L system buffer
- REF 04880293190, CleanCell M, 2 x 2 L measuring cell cleaning solution
- REF 03023141001, PC/CC-Cups, 12 cups to prewarm ProCell M and CleanCell M before use
- REF 03005712190, ProbeWash M, 12 x 70 mL cleaning solution for run finalization and rinsing during reagent change
- REF 03004899190, PreClean M, 5 x 600 mL detection cleaning solution
- REF 12102137001, AssayTip/AssayCup Combimagazine M, 48 magazines x 84 reaction vessels or pipette tips, waste bags
- REF 03023150001, WasteLiner, waste bags
- REF 03027651001, SysClean Adapter M

Accessories for all analyzers:

- REF 11298500316, ISE Cleaning Solution/Elecsys SysClean, 5 x 100 mL system cleaning solution

Assay

For optimum performance of the assay follow the directions given in this document for the analyzer concerned. Refer to the appropriate operator's manual for analyzer-specific assay instructions.

Resuspension of the microparticles takes place automatically prior to use. Read in the test-specific parameters via the reagent barcode. If in exceptional cases the barcode cannot be read, enter the 15-digit sequence of numbers.

MODULAR ANALYTICS E170, **cobas e** 601 and **cobas e** 602 analyzers: PreClean M solution is necessary.

Bring the cooled reagents to approximately 20 °C and place on the reagent disk (20 °C) of the analyzer. Avoid foam formation. The system automatically regulates the temperature of the reagents and the opening/closing of the bottles.

Calibration

Traceability: This method has been standardized against LC-MS/MS[15] which in turn has been standardized to the NIST standard.[16]

Every Elecsys reagent set has a barcoded label containing specific information for calibration of the particular reagent lot. The predefined master curve is adapted to the analyzer using the relevant CalSet.

For peer review only - http://bmjopen.bmj.com/site/about/guidelines.xhtml

ms_05894913190V7.0

Vitamin D total

cobas®

25-Hydroxyvitamin D

Calibration frequency: Calibration must be performed once per reagent lot using fresh reagent (i.e. not more than 24 hours since the reagent kit was registered on the analyzer). Renewed calibration is recommended as follows:

- after 1 month (28 days) when using the same reagent lot
- after 7 days (when using the same reagent kit on the analyzer)
- as required: e.g. quality control findings outside the defined limits

Quality control

For quality control, use PreciControl Varia.

In addition, other suitable control material can be used.

Controls for the various concentration ranges should be run individually at least once every 24 hours when the test is in use, once per reagent kit, and following each calibration.

The control intervals and limits should be adapted to each laboratory's individual requirements. Values obtained should fall within the defined limits. Each laboratory should establish corrective measures to be taken if values fall outside the defined limits.

Follow the applicable government regulations and local guidelines for quality control.

Calculation

The analyzer automatically calculates the analyte concentration of each sample (either in ng/mL or nmol/L).

Conversion factors:	nmol/L x 0.40 = ng/mL
	ng/mL x 2.50 = nmol/L

Limitations - interference

Samples showing visible signs of hemolysis may cause interference.

Hemoglobin concentrations > 2 g/L (> 0.124 mmol/L) may lead to elevated results.

The assay is unaffected by icterus (bilirubin < 1129 µmol/L or < 66 mg/dL), lipemia (Intralipid < 400 mg/dL) and biotin (< 287 nmol/L or < 70 ng/mL).

Criterion: For concentrations from LoQ up to 15 ng/mL, deviation is ≤ 1.5 ng/mL; for concentrations > 15 ng/mL, deviation is ≤ 10 %.

Samples should not be taken from patients receiving therapy with high biotin doses (i.e. > 5 mg/day) until at least 8 hours following the last biotin administration.

In vitro tests were performed on 17 commonly used pharmaceuticals and 5 special therapeutic drugs (Bonviva (Ibandronate), EinsAlpha (Alfacalcidol), Fosamax (Alendronate), Pamidron HEXAL (Pamidronate) and Zometa (Zoledronate)). No interference with the assay was found.

In rare cases, interference due to extremely high titers of antibodies to analyte-specific antibodies, streptavidin or ruthenium can occur. These effects are minimized by suitable test design.

For diagnostic purposes, the results should always be assessed in conjunction with the patient's medical history, clinical examination and other findings.

Limits and ranges

Measuring range

3.00-70.0 ng/mL or 7.50-175 nmol/L (defined by the Limit of Detection and the maximum of the master curve). Values below the Limit of Detection are reported as < 3.00 ng/mL (< 7.50 nmol/L). Values above the measuring range are reported as > 70.0 ng/mL (> 175 nmol/L).

Lower limits of measurement

Limit of Blank (LoB), Limit of Detection (LoD) and Limit of Quantitation (LoQ)

Limit of Blank = 2.00 ng/mL (5.00 nmol/L)

Limit of Detection = 3.00 ng/mL (7.50 nmol/L)

Limit of Quantitation = 5.00 ng/mL (12.5 nmol/L) with a total allowable relative error of ≤ 30 %

The Limit of Blank, Limit of Detection and Limit of Quantitation were determined in accordance with the CLSI (Clinical and Laboratory Standards Institute) EP17-A requirements.

The Limit of Blank is the 95th percentile value from n ≥ 60 measurements of analyte-free samples over several independent series. The Limit of Blank corresponds to the concentration below which analyte-free samples are found with a probability of 95 %.

The Limit of Detection is determined based on the Limit of Blank and the standard deviation of low concentration samples. The Limit of Detection corresponds to the lowest analyte concentration which can be detected (value above the Limit of Blank with a probability of 95 %).

The Limit of Quantitation is defined as the lowest amount of analyte in a sample that can be accurately quantitated with a total allowable relative error of ≤ 30 %.

The total error concept describes the maximum possible error of a test result taking into account the imprecision (SD) and inaccuracy (bias) of the test system. The Total Error (TE) was determined using the RMS (Root Mean Square) model (CLSI EP17-A2). The relative allowable total error refers to the respective concentration of the sample.

Dilution

Samples with vitamin D (25-OH) concentrations above the measuring range can be manually diluted with Diluent Universal or a suitable human serum with a low analyte concentration. The recommended dilution is 1:2. The concentration of the diluted sample must be > 30.0 ng/mL (> 75.0 nmol/L). After manual dilution, multiply the results by the dilution factor 2. The endogenous analyte concentration of the human serum used for dilution has to be taken into account.

Expected values

Due to different standardizations between methods, result variation may arise. Clinical assessment should be taken into consideration when interpreting results.

Health based reference values (recommended for use):

Currently there is no standard definition of the optimal vitamin D status. Many specialists consider the commonly used population based reference values too low. Health based reference values are recommended to replace population based reference values.[17]

Most experts agree that vitamin D deficiency should be defined as vitamin D (25-OH) of ≤ 20 ng/mL (≤ 50 nmol/L).[18] Vitamin D insufficiency is recognized as 21-29 ng/mL.[18] Similarly, the US National Kidney Foundation considers levels < 30 ng/mL to be insufficient or deficient.[19]

The preferred level for vitamin D (25-OH) by many experts is now recommended to be ≥ 30 ng/mL (≥ 75 nmol/L).[18,20,21,22]

Reference values measured in an apparently healthy population:

It should be taken into consideration that differences in vitamin D (25-OH) levels may exist with respect to gender, age, season, geographical latitude and ethnic groups.[18,20]

Each laboratory should investigate the transferability of the expected values to its own patient population and if necessary determine its own reference ranges.

Population based reference ranges should not be taken as clinical cutoff to recommend or dissuade from vitamin D supplementation. Guidance for supplementation should be taken from recent literature.[18,19]

A reference range study was conducted with samples from apparently healthy individuals of Caucasian heritage. The age range was 20-77 years. Samples were collected between November and July in northern Germany.

The values given are for information only and may vary from other published data.

			Gender			
	All (n = 453)		Female (n = 252)		Male (n = 201)	
Unit	ng/mL	nmol/L	ng/mL	nmol/L	ng/mL	nmol/L
Mean	20.6	51.5	21.6	54.0	19.4	48.5
2.5th percentile	5.26	13.2	6.23	15.6	4.92	12.3
97.5th percentile	47.0	118	49.9	125	42.7	107

A lower recovery may be found in particular clinical cohorts, for example dialysis patients.[23]

Specific performance data

Representative performance data on the analyzers are given below. Results obtained in individual laboratories may differ.

For peer review only - http://bmjopen.bmj.com/site/about/guidelines.xhtml

ms_05894913190V7.0

Vitamin D total

cobas®

25-Hydroxyvitamin D

Precision

Precision was determined using Elecsys reagents, pooled human sera and controls in a protocol (EP5-A2) of the CLSI (Clinical and Laboratory Standards Institute): 2 runs per day in duplication each for 21 days (n = 84). The following results were obtained:

Elecsys 2010 and **cobas e** 411 analyzers					
			**Repeatability**		
Sample	Mean		SD		CV
	ng/mL	nmol/L	ng/mL	nmol/L	%
HS[a)] 1	6.76	16.9	0.525	1.31	7.8
HS 2	15.0	37.5	0.770	1.93	5.1
HS 3	28.0	70.0	0.860	2.15	3.1
HS 4	67.0	168	1.15	2.88	1.7
PC[b)] Varia 1	19.9	49.8	0.948	2.37	4.8
PC Varia 2	38.3	95.8	1.05	2.63	2.7

a) HS = human serum

b) PC = PreciControl

Elecsys 2010 and **cobas e** 411 analyzers					
			**Intermediate precision**		
Sample	Mean		SD		CV
	ng/mL	nmol/L	ng/mL	nmol/L	%
HS 1	6.76	16.9	0.724	1.81	10.7
HS 2	15.0	37.5	1.28	3.20	8.5
HS 3	28.0	70.0	1.46	3.65	5.2
HS 4	67.0	168	1.46	3.65	2.2
PC Varia 1	19.9	49.8	1.23	3.08	6.2
PC Varia 2	38.3	95.8	1.41	3.53	3.7

MODULAR ANALYTICS E170, **cobas e** 601 and **cobas e** 602 analyzers					
			**Repeatability**		
Sample	Mean		SD		CV
	ng/mL	nmol/L	ng/mL	nmol/L	%
HS 1	8.35	20.9	0.567	1.42	6.8
HS 2	15.8	39.5	0.824	2.06	5.2
HS 3	28.3	70.8	1.11	2.78	3.9
HS 4	69.6	174	1.50	3.75	2.2
PC Varia 1	20.2	50.5	0.924	2.31	4.6
PC Varia 2	39.6	99.0	1.06	2.65	2.7

MODULAR ANALYTICS E170, **cobas e** 601 and **cobas e** 602 analyzers					
			**Intermediate precision**		
Sample	Mean		SD		CV
	ng/mL	nmol/L	ng/mL	nmol/L	%
HS 1	8.35	20.9	1.10	2.75	13.1
HS 2	15.8	39.5	1.18	2.95	7.5
HS 3	28.3	70.8	1.83	4.58	6.5
HS 4	69.6	174	2.37	5.93	3.4
PC Varia 1	20.2	50.5	0.954	2.39	4.7
PC Varia 2	39.6	99.0	1.38	3.45	3.5

Method comparison

1) A comparison of the Elecsys Vitamin D total assay (y) using samples measured with LC-MS/MS (x) gave the following correlations (ng/mL):

Number of samples measured: 903

Passing/Bablok[24] y = 1.09x - 0.510

Pearson r = 0.894

The sample concentrations were between approximately 3 ng/mL (7.5 nmol/L) and 81 ng/mL (203 nmol/L).

2) A comparison of the Elecsys Vitamin D total assay (y) using samples measured with a commercially available vitamin D (25-OH) immunoassay (x) gave the following correlations (ng/mL):

Number of samples measured: 451

Passing/Bablok[24] y = 1.29x + 1.71

Pearson r = 0.803

The sample concentrations were between approximately 5 ng/mL (12.5 nmol/L) and 81 ng/mL (203 nmol/L).

Analytical specificity

The specificity was assessed at 50 % B_0 and the results are summarized in the following table:

Cross-reactant	Cross-reactivity (%)
25-hydroxyvitamin D_3	100
25-hydroxyvitamin D_2	92
24,25-dihydroxyvitamin D_3	149
C3-epimer of 25-hydroxyvitamin D_3	91
1,25-dihydroxyvitamin D_3	non detectable
1,25-dihydroxyvitamin D_2	non detectable
Vitamin D_3	non detectable
Vitamin D_2	non detectable

Functional sensitivity

The functional sensitivity is the lowest analyte concentration that can be reproducibly measured with an intermediate precision CV of ≤ 20 %.
8 samples with concentrations between 0.722 ng/mL and 10.1 ng/mL were measured on several days. The functional sensitivity was determined to be 4.01 ng/mL (CV 18.5 %).

References

1 Holick M. Vitamin D: the underappreciated D-lightful hormone that is important for skeletal and cellular health. Curr Opin Endocrinol Diabetes 2002;9(1)87-98.

2 Holick MF. Vitamin D deficiency. N Engl J Med 2007;357:266-281.

3 Houghton LA, Vieth R. The case against ergocalciferol (vitamin D2) as a vitamin supplement. Am J Clin Nutr 2006;84:694-697.

4 Hart GR, Furniss JL, Laurie D, et al. Measurement of vitamin D Status: background, clinical use and methodologies. Clin Lab 2006;52(7-8):335-343.

5 Armas LAG, Hollis BW, Heaney RP. Vitamin D2 is much less effective than Vitamin D3 in humans. J Clin Endocrinol Metab 2004;89(11):5387-5391.

6 Steingrimsdottir L, Gunnarsson O, Indridason OS, et al. Relationship between serum parathyroid hormone levels, vitamin D sufficiency, and calcium intake. JAMA 2005 Nov 9;294(18):2336-2341.

7 Venning G. Recent developments in vitamin D deficiency and muscle weakness among elderly people. BMJ 2005;330:524-526.

8 Lips P. Vitamin D deficiency and secondary hyperparathyroidism in the elderly: consequences for bone loss and fractures and therapeutic implications. Endocr Rev 2001 Aug;22(4):447-501.

9 Souberbielle JC, Lawson-Body E, Hammadi B, et al. The use in clinical practice of parathyroid hormone normative values established in vitamin D-sufficient subjects. J Clin Endocrinol Metab 2003 Aug;88(8):3501-3504.

10 Willett AM. Vitamin D status and its relationship with parathyroid hormone and bone mineral status in older adolescents. Proceeding of the Nutrition Society 2005;64:193-203.

For peer review only - http://bmjopen.bmj.com/site/about/guidelines.xhtml

ms_05894913190V7.0

Vitamin D total

cobas®

25-Hydroxyvitamin D

11 Kuchukk NO, van Schoor NM, Pluijm SM, et al. Vitamin D status, parathyroid function, bone turnover, and BMD in postmenopausal women with osteoporosis: global perspective. J Bone Miner Res 2009;24:693-701.

12 Occupational Safety and Health Standards: Bloodborne pathogens. (29 CFR Part 1910.1030). Fed. Register.

13 Directive 2000/54/EC of the European Parliament and Council of 18 September 2000 on the protection of workers from risks related to exposure to biological agents at work.

14 Lewis JG, Elder PA. Serum 25-OH Vitamin D2 and D3 are Stable under Exaggerated Conditions. Clin Chem 2008;54:1931-1932.

15 Vogeser M, Kyriatsoulis A, Huber E, et al. Candidate Reference Method for the Quantification of Circulating 25-Hydroxyvitamin D3 by Liquid Chromatography-Tandem Mass Spectrometry. Clin Chem 2004;50:1415-1417.

16 Phinney KW. Development of a standard reference material for vitamin D in serum. Am J Clin Nutr 2008;88(suppl):511-512.

17 Bischoff-Ferrari HA, Giovannucci E, Willett WC, et al. Estimation of optimal serum concentrations of 25-hydroxyvitamin D for multiple health outcomes. Am J Clin Nutr 2006;84:18-28.

18 Holick MF. Vitamin D status: measurement, interpretation, and clinical application. Ann Epidemiol 2009;19(2):73-78.

19 KDOQI Clinical Practice Guidelines for Bone Metabolism and Disease in Children With Chronic Kidney Disease. http://www.kidney.org/PROFESSIONALS/kdoqi/guidelines_pedbone/guide8.htm

20 Souberbielle JC, Body JJ, Lappe JM, et al. Vitamin D and musculoskeletal health, cardiocascular disease, autoimmunity and cancer: Recommendations for clinical practice. Autoimmun Rev 2010;9:709-715.

21 Dawson-Hughes B, Heaney RP, Holick MF, et al. Estimates of optimal vitamin D status. Osteoporos Int 2005;16:713-716.

22 Vieth R. Why the minimum desirable serum 25-hydroxyvitamin D level should be 75 nmol/L (30 ng/mL). Best Pract Res Clin Endocrinol Metab 2011;25(4):681-691.

23 Heijboer AC, Blankenstein MA, Kema IP, et al. Accuracy of 6 routine 25-hydroxyvitamin D assays: influence of vitamin D binding protein concentration. Clin Chem 2012;58:543-548.

24 Bablok W, Passing H, Bender R, et al. A general regression procedure for method transformation. Application of linear regression procedures for method comparison studies in clinical chemistry, Part III. J Clin Chem Clin Biochem 1988 Nov;26(11):783-790.

For further information, please refer to the appropriate operator's manual for the analyzer concerned, the respective application sheets, the product information and the Method Sheets of all necessary components (if available in your country).

A point (period/stop) is always used in this Method Sheet as the decimal separator to mark the border between the integral and the fractional parts of a decimal numeral. Separators for thousands are not used.

Symbols

Roche Diagnostics uses the following symbols and signs in addition to those listed in the ISO 15223-1 standard.

CONTENT	Contents of kit
SYSTEM	Analyzers/Instruments on which reagents can be used
REAGENT	Reagent
CALIBRATOR	Calibrator
⟶	Volume after reconstitution or mixing
GTIN	Global Trade Item Number

COBAS, COBAS E, ELECSYS and PRECICONTROL are trademarks of Roche. INTRALIPID is a trademark of Fresenius Kabi AB.

All other product names and trademarks are the property of their respective owners.

Additions, deletions or changes are indicated by a change bar in the margin.

© 2015, Roche Diagnostics

CE

Roche Diagnostics GmbH, Sandhofer Strasse 116, D-68305 Mannheim
www.roche.com

Roche

For peer review only

For peer review only - http://bmjopen.bmj.com/site/about/guidelines.xhtml

ms_07212771190V1.0

Vitamin B12 II

cobas®

Vitamin B12

REF	Σ	SYSTEM
**07212771** 190	100	Elecsys 2010<br>MODULAR ANALYTICS E170<br>**cobas e** 411<br>**cobas e** 601<br>**cobas e** 602

English

Intended use

Binding assay for the in vitro quantitative determination of vitamin B12 in human serum and plasma.

The **e**lectro**c**hemi**l**uminescence **i**mmuno**a**ssay "ECLIA" is intended for use on Elecsys and **cobas e** immunoassay analyzers.

Summary

Vitamin B12, also referred to as cobalamin, is a complex organometallic compound in which a cobalt atom is situated within a corrin ring. It is a water-soluble vitamin which is synthesized by microorganisms. It cannot be synthesized in the human body and is seldom found in products of plant origin. Main sources of vitamin B12 are meat, fish, eggs and dairy products.[1] The uptake in the gastrointestinal tract depends on intrinsic factor, which is synthesized by the gastric parietal cells, and on the "cubam receptor" in the distal ileum. The most frequent cause of severe vitamin B12 deficiency is a lack of intrinsic factor due to autoimmune atrophic gastritis. The disease is historically called "pernicious anemia", even though many patients present with mainly neurologic manifestations. Examples of other causes for vitamin B12 deficiency are malabsorption due to gastrectomy, inflammatory bowel disease or dietary deficiency, e.g. in strict vegetarians (vegans).[2]

Vitamin B12 is the cofactor for two enzymes, methionine synthase and methylmalonyl CoA mutase.[2,3] Methionine synthase, located in the cytoplasm, requires vitamin B12 in the form of methylcobalamin and catalyzes the conversion of homocysteine to methionine, an essential amino acid. During this step a methyl group is transferred from methyltetrahydrofolate to the amino acid.[3] This enzyme links the methylation pathway through synthesis of the methyl donor S-Adenosyl methionine and the pathway in which purine and pyrimidine are synthesized via generation of tetrahydrofolate.[3] In the form of 5'-deoxyadenosylcobalamin, vitamin B12 is also required for the mitochondrial enzyme methylmalonyl CoA mutase, which converts methylmalonyl CoA to succinyl CoA. This is a step in the oxidation of odd-chain fatty acids and catabolism of ketogenic amino acids.[3] Thus, vitamin B12 is important for DNA synthesis, regenerating methionine for protein synthesis and methylation, as well as for the development and initial myelination of the central nervous system (CNS) and for the maintenance of normal CNS function.[2,3]

Vitamin B12 deficiencies are common in wealthier countries principally among the elderly and are most prevalent in poorer populations. In general the prevalence increases with age.[4,5]

Vitamin B12 deficiency impacts red blood cell synthesis, resulting in megaloblastic anemia due to abnormal DNA synthesis.[3] In addition it impairs neurological function, in particular demyelination of nerves in part due to abnormal methylation, leading to peripheral neuropathy, dementia, poor cognitive performance, and depression.[3] Other effects of vitamin B12 deficiency or depletion are increased risk of neural tube defects, osteoporosis, cerebrovascular and cardiovascular diseases.[3] Early diagnosis is essential, because of the latent nature of this disorder and the risk of permanent neurological damage.[3,5]

Generally, the primary test performed to confirm the diagnosis of vitamin B12 deficiency is measurement of serum vitamin B12 level.[2] Recent publications suggest that in addition the following biomarkers should be measured to improve the specificity of diagnosis: folate, methylmalonic acid (MMA), homocysteine and holotranscobalamin.[2,5,6,7]

The Elecsys Vitamin B12 II assay employs a competitive test principle using intrinsic factor specific for vitamin B12. Vitamin B12 in the sample competes with the added vitamin B12 labeled with biotin for the binding sites on the ruthenium-labeled intrinsic factor complex[a)].

a) Tris(2,2'-bipyridyl)ruthenium(II)-complex (Ru(bpy)$_3^{2+}$)

Test principle

Competition principle. Total duration of assay: 27 minutes.

- 1st incubation: By incubating the sample (15 µL) with the vitamin B12 pretreatment 1 and pretreatment 2, bound vitamin B12 is released.
- 2nd incubation: By incubating the pretreated sample with the ruthenium labeled intrinsic factor, a vitamin B12-binding protein complex is formed, the amount of which is dependent upon the analyte concentration in the sample.
- 3rd incubation: After addition of streptavidin-coated microparticles and vitamin B12 labeled with biotin, the still-vacant sites of the ruthenium labeled intrinsic factor become occupied, with formation of a ruthenium labeled intrinsic factor vitamin B12 biotin complex. The entire complex becomes bound to the solid phase via interaction of biotin and streptavidin.
- The reaction mixture is aspirated into the measuring cell where the microparticles are magnetically captured onto the surface of the electrode. Unbound substances are then removed with ProCell/ProCell M. Application of a voltage to the electrode then induces chemiluminescent emission which is measured by a photomultiplier.
- Results are determined via a calibration curve which is instrument-specifically generated by 2-point calibration and a master curve provided via the reagent barcode.

Reagents - working solutions

The reagent rackpack (M, R1, R2) and the pretreatment reagents (PT1, PT2) are labeled as B12 II.

PT1 Pretreatment reagent 1 (white cap), 1 bottle, 4 mL:
Dithiothreitol 1.028 g/L; stabilizer, pH 5.5.

PT2 Pretreatment reagent 2 (gray cap), 1 bottle, 4 mL:
Sodium hydroxide 40 g/L; sodium cyanide 2.205 g/L.

M Streptavidin-coated microparticles (transparent cap), 1 bottle, 6.5 mL:
Streptavidin-coated microparticles 0.72 mg/mL; preservative.

R1 Intrinsic factor~Ru(bpy)$_3^{2+}$ (gray cap), 1 bottle, 10 mL:
Ruthenium labeled recombinant porcine intrinsic factor 4 µg/L; cobinamide dicyanide 15 µg/L; stabilizer; human serum albumin; phosphate buffer, pH 5.5; preservative.

R2 Vitamin B12~biotin (black cap), 1 bottle, 8.5 mL:
Biotinylated vitamin B12 25 µg/L; biotin 3 µg/L; phosphate buffer, pH 7.0; preservative.

Precautions and warnings

For in vitro diagnostic use.
Exercise the normal precautions required for handling all laboratory reagents.
Disposal of all waste material should be in accordance with local guidelines.
Safety data sheet available for professional user on request.

This kit contains components classified as follows in accordance with the Regulation (EC) No. 1272/2008:

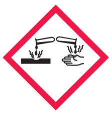

Danger

For peer review only - http://bmjopen.bmj.com/site/about/guidelines.xhtml

ms_07212771190V1.0

Vitamin B12 II

cobas®

Vitamin B12

H290 May be corrosive to metals.

H314 Causes severe skin burns and eye damage.

H412 Harmful to aquatic life with long lasting effects.

Prevention:

P234 Keep only in original container.

P264 Wash skin thoroughly after handling.

P273 Avoid release to the environment.

P280 Wear protective gloves/ protective clothing/ eye protection/ face protection.

Response:

P301 + P330 + P331 IF SWALLOWED: Rinse mouth. Do NOT induce vomiting.

P303 + P361 + P353 IF ON SKIN (or hair): Remove/Take off immediately all contaminated clothing. Rinse skin with water/shower.

P304 + P340 IF INHALED: Remove person to fresh air and keep comfortable for breathing.

P305 + P351 + P338 IF IN EYES: Rinse cautiously with water for several minutes. Remove contact lenses, if present and easy to do. Continue rinsing.

P310 Immediately call a POISON CENTER or doctor/physician.

P363 Wash contaminated clothing before reuse.

P390 Absorb spillage to prevent material damage.

Storage:

P405 Store locked up.

P406 Store in corrosive resistant stainless steel container with a resistant inner liner.

Disposal:

P501 Dispose of contents/container to an approved waste disposal plant.

Product safety labeling primarily follows EU GHS guidance.

Contact phone: all countries: +49-621-7590

All human material should be considered potentially infectious. All products derived from human blood are prepared exclusively from the blood of donors tested individually and shown to be free from HBsAg and antibodies to HCV and HIV. The testing methods applied were FDA-approved or cleared in compliance with the European Directive 98/79/EC, Annex II, List A.

However, as no testing method can rule out the potential risk of infection with absolute certainty, the material should be handled with the same level of care as a patient specimen. In the event of exposure, the directives of the responsible health authorities should be followed.[8,9]

Avoid foam formation in all reagents and sample types (specimens, calibrators and controls).

Reagent handling

The reagents in the kit have been assembled into a ready-for-use unit that cannot be separated.

All information required for correct operation is read in from the respective reagent barcodes.

Storage and stability

Store at 2-8 °C.

Do not freeze.

Store the Elecsys reagent kit **upright** in order to ensure complete availability of the microparticles during automatic mixing prior to use.

Stability:	
unopened at 2-8 °C	up to the stated expiration date
after opening at 2-8 °C	84 days (12 weeks)
on the analyzers	35 days (5 weeks) onboard<br>or<br>60 days when stored alternatively in the refrigerator and on the analyzer, with the total time onboard on the analyzer not exceeding<br>10 x 8 hours

Specimen collection and preparation

Only the specimens listed below were tested and found acceptable.

Serum collected using standard sampling tubes or tubes containing separating gel.

Na-heparin, Li-heparin, K_2-EDTA and K_3-EDTA plasma. Li-heparin plasma tubes containing separating gel can be used.

Criterion: Slope 0.9-1.1 + intercept within < ± 2x Limit of Blank (LoB) + coefficient of correlation ≥ 0.95.

Stable for 2 hours at 15-25 °C, 48 hours at 2-8 °C, 56 days at (-15)-(-25) °C. Freeze once only.

Stability of serum obtained with separating tubes: 24 hours at 2-8 °C (note the data provided by the tube manufacturer).

The sample types listed were tested with a selection of sample collection tubes that were commercially available at the time of testing, i.e. not all available tubes of all manufacturers were tested. Sample collection systems from various manufacturers may contain differing materials which could affect the test results in some cases. When processing samples in primary tubes (sample collection systems), follow the instructions of the tube manufacturer.

Centrifuge samples containing precipitates before performing the assay.

Do not use heat-inactivated samples.

Avoid hemolysis.

Do not use samples and controls stabilized with azide.

Vitamin B12 determinations should be performed on serum or plasma samples from fasting patients.

Note: Samples with extremely high total protein concentrations (e.g. patients suffering from Waldenström's macroglobulinemia) are not suitable for use in this assay, since they may lead to the formation of protein gel in the assay cup. Processing protein gel may cause a run abort. The critical protein concentration is dependent upon the individual sample composition. The formation of protein gel was seen in samples (spiked with human IgG or human serum albumin) having a total protein concentration > 160 g/L.

Ensure the samples, calibrators and controls are at 20-25 °C prior to measurement.

Due to possible evaporation effects, samples, calibrators and controls on the analyzers should be analyzed/measured within 2 hours.

Materials provided

See "Reagents – working solutions" section for reagents.

Materials required (but not provided)

- REF 07212780190, Vitamin B12 II CalSet, for 4 x 1 mL
- REF 05618860190, PreciControl Varia, for 2 x 3 mL each of PreciControl Varia 1 and 2
- REF 11732277122, Diluent Universal, 2 x 16 mL sample diluent or REF 03183971122, Diluent Universal, 2 x 36 mL sample diluent
- General laboratory equipment
- Elecsys 2010, MODULAR ANALYTICS E170 or **cobas e** analyzer

Accessories for Elecsys 2010 and **cobas e** 411 analyzers:

- REF 11662988122, ProCell, 6 x 380 mL system buffer
- REF 11662970122, CleanCell, 6 x 380 mL measuring cell cleaning solution

For peer review only - http://bmjopen.bmj.com/site/about/guidelines.xhtml

ms_07212771190V1.0

Vitamin B12 II

cobas®

Vitamin B12

- REF 11930346122, Elecsys SysWash, 1 x 500 mL washwater additive
- REF 11933159001, Adapter for SysClean
- REF 11706802001, Elecsys 2010 AssayCup, 60 x 60 reaction vessels
- REF 11706799001, Elecsys 2010 AssayTip, 30 x 120 pipette tips

Accessories for MODULAR ANALYTICS E170, **cobas e** 601 and **cobas e** 602 analyzers:

- REF 04880340190, ProCell M, 2 x 2 L system buffer
- REF 04880293190, CleanCell M, 2 x 2 L measuring cell cleaning solution
- REF 03023141001, PC/CC-Cups, 12 cups to prewarm ProCell M and CleanCell M before use
- REF 03005712190, ProbeWash M, 12 x 70 mL cleaning solution for run finalization and rinsing during reagent change
- REF 03004899190, PreClean M, 5 x 600 mL detection cleaning solution
- REF 12102137001, AssayTip/AssayCup Combimagazine M, 48 magazines x 84 reaction vessels or pipette tips, waste bags
- REF 03023150001, WasteLiner, waste bags
- REF 03027651001, SysClean Adapter M

Accessories for all analyzers:

- REF 11298500316, ISE Cleaning Solution/Elecsys SysClean, 5 x 100 mL system cleaning solution

Assay

For optimum performance of the assay follow the directions given in this document for the analyzer concerned. Refer to the appropriate operator's manual for analyzer-specific assay instructions.

Resuspension of the microparticles takes place automatically prior to use. Read in the test-specific parameters via the reagent barcode. If in exceptional cases the barcode cannot be read, enter the 15-digit sequence of numbers.

MODULAR ANALYTICS E170, **cobas e** 601 and **cobas e** 602 analyzers: PreClean M solution is necessary.

Bring the cooled reagents to approximately 20 °C and place on the reagent disk (20 °C) of the analyzer. Avoid foam formation. The system automatically regulates the temperature of the reagents and the opening/closing of the bottles.

Calibration

Traceability: This method has been standardized against the Vitamin B12 assay (REF 04745736).

Every Elecsys reagent set has a barcoded label containing specific information for calibration of the particular reagent lot. The predefined master curve is adapted to the analyzer using the relevant CalSet.

Calibration frequency: Calibration must be performed once per reagent lot using fresh reagent (i.e. not more than 24 hours since the reagent kit was registered on the analyzer). Renewed calibration is recommended as follows:

- after 1 month (28 days) when using the same reagent lot
- after 7 days (when using the same reagent kit on the analyzer)
- as required: e.g. quality control findings outside the defined limits

Quality control

For quality control, use PreciControl Varia.

In addition, other suitable control material can be used.

Controls for the various concentration ranges should be run individually at least once every 24 hours when the test is in use, once per reagent kit, and following each calibration.

The control intervals and limits should be adapted to each laboratory's individual requirements. Values obtained should fall within the defined limits. Each laboratory should establish corrective measures to be taken if values fall outside the defined limits.

Follow the applicable government regulations and local guidelines for quality control.

Calculation

The analyzer automatically calculates the analyte concentration of each sample (either in pmol/L or pg/mL).

Conversion factors:	pmol/L x 1.36 = pg/mL
	pg/mL x 0.738 = pmol/L

Limitations - interference

The assay is unaffected by icterus (bilirubin ≤ 1112 µmol/L or ≤ 65 mg/dL), hemolysis (Hb ≤ 0.025 mmol/L or ≤ 0.04 g/dL), lipemia (triglycerides ≤ 17.1 mmol/L or ≤ 1500 mg/dL), biotin (≤ 205 nmol/L or ≤ 50 ng/mL), IgG ≤ 28 g/L, IgA ≤ 16 g/L and IgM ≤ 10 g/L.

Criterion: Recovery within ± 10 % of initial value with samples > 200 pg/mL and ≤ ± 20 pg/mL with samples ≤ 200 pg/mL.

Samples should not be taken from patients receiving therapy with high biotin doses (i.e. > 5 mg/day) until at least 8 hours following the last biotin administration.

No interference was observed from rheumatoid factors up to a concentration of 1500 IU/mL.

In vitro tests were performed on 16 commonly used pharmaceuticals. No interference with the assay was found.

In rare cases, interference due to extremely high titers of antibodies to analyte-specific antibodies, streptavidin or ruthenium can occur. These effects are minimized by suitable test design.

Because intrinsic factor is typically used as the binding protein in serum vitamin B12 assays, anti-intrinsic factor antibodies (which are common in pernicious anemia) can lead to elevated vitamin B12 measurement values.[2,10,11] The Elecsys Vitamin B12 II assay is designed to avoid interference due to anti-intrinsic factor antibodies.[12]

For diagnostic purposes, the results should always be assessed in conjunction with the patient's medical history, clinical examination and other findings.

Note: The presence of immunoglobulin-vitamin B12 complexes may cause unexpectedly high values of vitamin B12.[13,14]

Limits and ranges

Measuring range

50.0-2000 pg/mL or 36.9-1476 pmol/L (defined by the Limit of Blank and the maximum of the master curve). Values below the Limit of Blank are reported as < 50.0 pg/mL or < 36.9 pmol/L. Values above the measuring range are reported as > 2000 pg/mL or > 1476 pmol/L.

Lower limits of measurement

Limit of Blank (LoB), Limit of Detection (LoD) and Limit of Quantitation (LoQ)

Limit of Blank = 50 pg/mL (36.9 pmol/L)

Limit of Detection = 100 pg/mL (73.8 pmol/L)

Limit of Quantitation = 150 pg/mL (111 pmol/L) with a allowable imprecision of ≤ 20 %

The Limit of Blank, Limit of Detection and Limit of Quantitation were determined in accordance with the CLSI (Clinical and Laboratory Standards Institute) EP17-A requirements.

The Limit of Blank is the 95th percentile value from n ≥ 60 measurements of analyte-free samples over several independent series. The Limit of Blank corresponds to the concentration below which analyte-free samples are found with a probability of 95 %.

The Limit of Detection is determined based on the Limit of Blank and the standard deviation of low concentration samples. The Limit of Detection corresponds to the lowest analyte concentration which can be detected (value above the Limit of Blank with a probability of 95 %).

The Limit of Quantitation is defined as the lowest amount of analyte in a sample that can be accurately quantitated with a allowable imprecision of ≤ 20 %.

It has been determined using low concentration vitamin B12 samples.

Dilution

Samples with vitamin B12 concentrations above the measuring range can be manually diluted 1:2 with Diluent Universal. The concentration of the diluted sample must be > 738 pmol/L or > 1000 pg/mL. After manual dilution, multiply the results by the dilution factor 2.

Note: Sample-dependent non-linearity upon dilution is seen with samples having analyte levels beyond the measuring range. As Diluent Universal may contain low levels of endogenous vitamin B12, it is recommended that linearity studies be performed using a known low analyte-containing serum

For peer review only - http://bmjopen.bmj.com/site/about/guidelines.xhtml

ms_07212771190V1.0

Vitamin B12 II

Vitamin B12

pool. Samples outside the measuring range can be diluted 1:2 with Diluent Universal; the effect of endogenous vitamin B12 concentration is insignificant at these levels.

Expected values

Because differences may exist with respect to population and dietary status, it is recommended that normal ranges be determined by each laboratory over a suitable period of time and in a statistically significant number of assays before clinical significance is attached to the results of these tests.

The values shown below were performed on samples from an apparently healthy population, using the Elecsys Vitamin B12 II assay. The calculation is based on 135 sera (68 men, 67 women). The age range was between 20 and 78 years. Pregnant women were excluded. The reference population was selected according to normal homocysteine values.

N	Median		Range (2.5^{th}-97.5^{th} percentile)	
	pg/mL	pmol/L	pg/mL	pmol/L
135	425	314	197-771	145-569

These values should only be used as guidelines.

Each laboratory should investigate the transferability of the expected values to its own patient population and if necessary determine its own reference ranges.

Specific performance data

Representative performance data on the analyzers are given below. Results obtained in individual laboratories may differ.

Precision

Precision was determined using Elecsys reagents, pooled human sera and controls in a protocol (EP5-A2) of the CLSI (Clinical and Laboratory Standards Institute): 2 runs per day in duplication each for 21 days (n = 84). The following results were obtained:

Elecsys 2010 and **cobas e** 411 analyzers					
		**Repeatability**		**Intermediate precision**	
Sample	Mean	SD	CV	SD	CV
	pg/mL	pg/mL	%	pg/mL	%
Human serum 1	176	8.86	5.0	12.7	7.2
Human serum 2	405	13.0	3.2	17.5	4.3
Human serum 3	960	19.7	2.1	31.0	3.2
Human serum 4	1230	27.4	2.2	46.4	3.8
Human serum 5	1940	40.9	2.1	72.6	3.7
PreciControl Varia1	447	12.2	2.7	18.6	4.2
PreciControl Varia2	934	20.2	2.2	38.4	4.1

Elecsys 2010 and **cobas e** 411 analyzers					
		**Repeatability**		**Intermediate precision**	
Sample	Mean	SD	CV	SD	CV
	pmol/L	pmol/L	%	pmol/L	%
Human serum 1	130	6.54	5.0	9.37	7.2
Human serum 2	299	9.59	3.2	12.9	4.3
Human serum 3	708	14.5	2.1	22.9	3.2
Human serum 4	908	20.2	2.2	34.2	3.8
Human serum 5	1432	30.2	2.1	53.6	3.7
PreciControl Varia1	330	9.00	2.7	13.7	4.2
PreciControl Varia2	689	14.9	2.2	28.3	4.1

MODULAR ANALYTICS E170, **cobas e** 601 and **cobas e** 602 analyzers					
		**Repeatability**		**Intermediate precision**	
Sample	Mean	SD	CV	SD	CV
	pg/mL	pg/mL	%	pg/mL	%
Human serum 1	176	5.84	3.3	9.14	5.2
Human serum 2	407	8.24	2.0	12.7	3.1
Human serum 3	1010	13.2	1.3	21.1	2.1
Human serum 4	1230	19.8	1.6	28.8	2.3
Human serum 5	1890	29.8	1.6	41.5	2.2
PreciControl Varia1	448	7.16	1.6	15.3	3.4
PreciControl Varia2	917	12.0	1.3	27.8	3.0

MODULAR ANALYTICS E170, **cobas e** 601 and **cobas e** 602 analyzers					
		**Repeatability**		**Intermediate precision**	
Sample	Mean	SD	CV	SD	CV
	pmol/L	pmol/L	%	pmol/L	%
Human serum 1	130	4.31	3.3	6.75	5.2
Human serum 2	300	6.08	2.0	9.37	3.1
Human serum 3	745	9.74	1.3	15.6	2.1
Human serum 4	908	14.6	1.6	21.3	2.3
Human serum 5	1395	22.0	1.6	30.6	2.2
PreciControl Varia1	331	5.28	1.6	11.3	3.4
PreciControl Varia2	677	8.86	1.3	20.5	3.0

Method comparison

a) A comparison of the Elecsys Vitamin B12 assay (calibrated with Vitamin B12 CalSet II; x) and the Elecsys Vitamin B12 II assay (calibrated with Vitamin B12 II CalSet; y) using clinical samples gave the following correlations (pg/mL):

Number of samples measured: 100

Passing/Bablok[15]	Linear regression
y = 0.952x + 15.1	y = 0.957x + 11.6
τ = 0.977	r = 0.999

The sample concentrations were between 69 and 1890 pg/mL (51 and 1395 pmol/L).

b) A comparison of the Elecsys Vitamin B12 II assay (y) and a commercially available method (x) using clinical samples gave the following correlations (pg/mL):

Number of samples measured: 106

Passing/Bablok[15]	Linear regression
y = 0.923x + 4.90	y = 0.881x + 27.6
τ = 0.952	r = 0.993

The sample concentrations were between 182 and 1797 pg/mL (134 and 1326 pmol/L).

c) A comparison of the Elecsys Vitamin B12 II assay on the **cobas e** 601 analyzer (y) and the Elecsys Vitamin B12 II assay on the **cobas e** 411 analyzer (x) using clinical samples gave the following correlations (pg/mL):

Number of samples measured: 117

Passing/Bablok[15]	Linear regression
y = 1.01x - 2.77	y = 1.01x + 3.22
τ = 0.933	r = 0.995

The sample concentrations were between 56 and 1887 pg/mL (41 and 1393 pmol/L).

For peer review only

For peer review only - http://bmjopen.bmj.com/site/about/guidelines.xhtml

ms_07212771190V1.0

Vitamin B12 II

cobas®

Vitamin B12

Analytical specificity

The following cross-reactivities were found, tested with vitamin B12 concentrations of 129 pg/mL and 550 pg/mL.

Cross-reactant	Maximum concentration tested ng/mL	Cross-reactivity %
Cobinamide dicyanide	210	0.003

References

1 Thomas L. Clinical Laboratory Diagnostics: Use and Assessment of Clinical Laboratory Results; 1st Edition, Frankfurt/Main: TH-Books-Verl.-Ges.,1998:424-431.

2 Stabler SP. Vitamin B12 deficiency. N Engl J Med 2013;368:149-160.

3 Allen LH. Vitamin B-12. Adv Nutr 2012;3(1):54-55. doi: 10.3945/an.111.001370. Epub 2012 Jan 5.

4 Allen LH. How common is vitamin B-12 deficiency? Am J Clin Nutr 2009;89(2):693S-696S. Epub 2008 Dec 30.

5 Chatthanawaree W. Biomarkers of cobalamin (vitamin B12) deficiency and its application. J Nutr Health Aging 2011 Mar;15(3):227-313.

6 Yetley EA, Pfeiffer CM, Phinney KW, et al., Biomarkers of vitamin B-12 status in NHANES: a roundtable summary. Am J Clin Nutr 2011 Jul;94(1):313S-321S.

7 Hvas AM, Nexo E. Diagnosis and treatment of vitamin B12 deficiency - an update. Haematologica 2006;91(11):1506-1512.

8 Occupational Safety and Health Standards: bloodborne pathogens. (29 CFR Part 1910.1030). Fed. Register.

9 Directive 2000/54/EC of the European Parliament and Council of 18 September 2000 on the protection of workers from risks related to exposure to biological agents at work.

10 Yang DT, Cook RJ. Spurious elevations of vitamin B12 with pernicious anemia. N Engl J Med 2012;366:1742-1743.

11 Carmel R, Agrawal YP. Failures of cobalamin assays in pernicious anemia. N Engl J Med 2012;367:385-386. [Erratum, N Engl J Med 2012;367:976.]

12 Schilling KA, Wiesgigl M. The Elecsys® Vitamin B12 assay is not affected by anti-intrinsic factor antibodies. Clin Chem Lab Med 2013 Jun 29;51(11):e251-e252.

13 Jeffery J, Millar H, MacKenzie P, et al. An IgG complexed form of vitamin B12 is a common cause of elevated serum concentrations. Clin Biochem 2010 Jan;43(1-2):82-88. doi: 10.1016/j.clinbiochem.2009.08.022. Epub 2009 Sep 8.

14 Bowen RA, Drake SK, Vanjani R, et al. Markedly increased vitamin B12 concentrations attributable to IgG-IgM-vitamin B12 immune complexes. Clin Chem 2006;52(11):2107-2114.

15 Bablok W, Passing H, Bender R, et al. A general regression procedure for method transformation. Application of linear regression procedures for method comparison studies in clinical chemistry, Part III. J Clin Chem Clin Biochem 1988 Nov;26(11):783-790.

For further information, please refer to the appropriate operator's manual for the analyzer concerned, the respective application sheets, the product information and the Method Sheets of all necessary components (if available in your country).

A point (period/stop) is always used in this Method Sheet as the decimal separator to mark the border between the integral and the fractional parts of a decimal numeral. Separators for thousands are not used.

Symbols

Roche Diagnostics uses the following symbols and signs in addition to those listed in the ISO 15223-1 standard.

CONTENT	Contents of kit
SYSTEM	Analyzers/Instruments on which reagents can be used
REAGENT	Reagent
CALIBRATOR	Calibrator
→	Volume after reconstitution or mixing
GTIN	Global Trade Item Number

COBAS, COBAS E, ELECSYS and PRECICONTROL are trademarks of Roche. INTRALIPID is a trademark of Fresenius Kabi AB.

All other product names and trademarks are the property of their respective owners.

Additions, deletions or changes are indicated by a change bar in the margin.

© 2015, Roche Diagnostics

CE

Roche Diagnostics GmbH, Sandhofer Strasse 116, D-68305 Mannheim
www.roche.com

Roche

For peer review only

For peer review only - http://bmjopen.bmj.com/site/about/guidelines.xhtml

0107227841190c501V1.0

STFR2

cobas®

Tina-quant Soluble Transferrin Receptor II

Order information

REF	CONTENT		Analyzer(s) on which **cobas c** pack(s) can be used
07227841190	Tina-quant Soluble Transferrin Receptor II(100 tests)	System-ID 07 7473 1	**cobas c** 311, **cobas c** 501/502
08753776190	Calibrator sTfR II (3 x 1 mL)	Codes 697	
08278202190	ControlSet sTfR II		
	Level I (3 x 1 mL)	Level I Code 153	
	Level II (3 x 1 mL)	Level II Code 154	
04489357190	Diluent NaCl 9 % (50 mL)	System-ID 07 6869 3	

English

System information

For **cobas c** 311/501 analyzers:

STFR2: ACN 439

For **cobas c** 502 analyzer:

STFR2: ACN 8439

Intended use

In vitro test for the quantitative determination of soluble transferrin receptor (sTfR) in human serum and plasma on **cobas c** systems.

Summary[1,2,3,4,5,6,7]

The transferrin receptor is an integral membrane glycoprotein having a molecular weight of 190 kilodalton (kDa). It consists of two identical subunits linked by disulfide bridges. Each of the monomers has an 85 kDa C-terminal component which can bind an iron-laden transferrin molecule. Proteolysis leads to the soluble form of the transferrin receptor (sTfR). In plasma, the soluble transferrin receptor is present in the form of a complex with transferrin having a molecular weight of approximately 320 kD. The serum concentration of sTfR is directly proportional to the concentration of the receptor on the membrane.

The uptake of iron by the body's cells is controlled by expression of the transferrin receptor (TfR). If the intracellular iron stores are exhausted - corresponding to a ferritin concentration of less than 12 µg/L - then more TfR is expressed. The affinity of the transferrin receptor to transferrin depends on the latter's loading state. As 80-95 % of the transferrin receptor molecules are localized on erythropoietic cells, the TfR concentration (and hence also the sTfR concentration) reflects the iron requirement of these cells. When iron deficiency exists, the sTfR concentration in serum rises even before the hemoglobin concentration is significantly depressed. The sTfR concentration can therefore describe the functional iron status while ferritin reflects the iron storage status. A precise assessment of the iron status can be obtained by determining the sTfR index (= sTfR concentration/log ferritin concentration).

As - in contrast to ferritin - the concentration of sTfR is not affected by acute-phase reactions, acute liver function disorders or malignant tumors, it is possible to differentiate between anemia of chronic disease (ACD) and iron deficiency anemia (IDA). Elevated sTfR values are also found in polycythemia, hemolytic anemia, thalassemia, hereditary spherocytosis, sickle cell anemia, megaloblastic anemia, myelodysplastic syndrome and vitamin B_{12} deficiency. Elevated sTfR concentrations occur during pregnancy when there is a deficiency of functional iron.

Parameter	Change	IDA	ACD	IDA + ACD
Ferritin	iron stores	↓	↑	— or ↑
TIBC/TRSF	iron status	↑	↓	↑ or —
Serum iron	iron status	↓	↓	↓
sTfR	functional iron deficiency	↑	—	↑

↓ decreased, ↑ increased, — unchanged

Test principle[8]

Particle enhanced immunoturbidimetric assay.

Human soluble transferrin receptor agglutinates with latex particles coated with anti-soluble transferrin receptor antibodies. The precipitate is determined photometrically.

Reagents - working solutions

R1 TRIS buffer: with bovine serum albumin; preservatives

R2 Latex particles coated with monoclonal anti-human sTfR antibodies (mouse) in glycine buffer; preservative

R1 is in position B and R2 is in position C.

Precautions and warnings

For in vitro diagnostic use.
Exercise the normal precautions required for handling all laboratory reagents.
Disposal of all waste material should be in accordance with local guidelines.
Safety data sheet available for professional user on request.

This kit contains components classified as follows in accordance with the Regulation (EC) No. 1272/2008:

Contains 2-methyl-2H-isothiazol-3-one hydrochloride

EUH 208 May produce an allergic reaction.

Product safety labeling follows EU GHS guidance.

Reagent handling

Ready for use

Carefully invert reagent container several times prior to use to ensure that the reagent components are mixed.

Storage and stability

STFR2

Shelf life at 2-8 °C:	See expiration date on **cobas c** pack label.
On-board in use and refrigerated on the analyzer:	26 weeks

Diluent NaCl 9 %

Shelf life at 2-8 °C:	See expiration date on **cobas c** pack label.
On-board in use and refrigerated on the analyzer:	12 weeks

Specimen collection and preparation

For specimen collection and preparation only use suitable tubes or collection containers.

Only the specimens listed below were tested and found acceptable.
Serum.
Plasma: Heparin (Li-, Na-, $NH4^{+}$-) plasma

The sample types listed were tested with a selection of sample collection tubes that were commercially available at the time of testing, i.e. not all available tubes of all manufacturers were tested. Sample collection systems from various manufacturers may contain differing materials which could affect the test results in some cases. When processing samples in primary tubes (sample collection systems), follow the instructions of the tube manufacturer.

Centrifuge samples containing precipitates before performing the assay.

See the limitations and interferences section for details about possible sample interferences.

Stability:	6 days at 15-25 °C
	15 days at 2-8 °C
	13 weeks at -20 °C (±5 °C) (freeze only once)

For peer review only

For peer review only - http://bmjopen.bmj.com/site/about/guidelines.xhtml

0107227841190c501V1.0

STFR2

cobas®

Tina-quant Soluble Transferrin Receptor II

Sample stability claims were established by experimental data by the manufacturer or based on reference literature and only for the temperatures/time frames as stated in the method sheet. It is the responsibility of the individual laboratory to use all available references and/or its own studies to determine specific stability criteria for its laboratory.

Materials provided

See “Reagents – working solutions” section for reagents.

Materials required (but not provided)

- See “Order information” section
- General laboratory equipment

Assay

For optimum performance of the assay follow the directions given in this document for the analyzer concerned. Refer to the appropriate operator’s manual for analyzer-specific assay instructions.

The performance of applications not validated by Roche is not warranted and must be defined by the user.

Application for serum and plasma

cobas c **311 test definition**

Assay type	2-Point End		
Reaction time/Assay points	10 / 8-21		
Wavelength (sub/main)	800/570 nm		
Reaction direction	Increase		
Unit	mg/L (mg/dL, nmol/L)		
Reagent pipetting		Diluent (H_2O)	
R1	100 µL	-	
R2	40 µL	-	
Sample volumes	*Sample*	*Sample dilution*	
		Sample	*Diluent (NaCl)*
Normal	2 µL	-	-
Decreased	2 µL	25 µL	75 µL
Increased	2 µL		

cobas c **501 test definition**

Assay type	2-Point End		
Reaction time/Assay points	10 / 13-30		
Wavelength (sub/main)	800/570 nm		
Reaction direction	Increase		
Unit	mg/L (mg/dL, nmol/L)		
Reagent pipetting		Diluent (H_2O)	
R1	100 µL	-	
R2	40 µL	-	
Sample volumes	*Sample*	*Sample dilution*	
		Sample	*Diluent (NaCl)*
Normal	2 µL	-	-
Decreased	2 µL	25 µL	75 µL
Increased	2 µL		

cobas c **502 test definition**

Assay type	2-Point End		
Reaction time/Assay points	10 / 13-30		
Wavelength (sub/main)	800/570 nm		
Reaction direction	Increase		
Unit	mg/L (mg/dL, nmol/L)		
Reagent pipetting		Diluent (H_2O)	
R1	100 µL	-	
R2	40 µL	-	
Sample volumes	*Sample*	*Sample dilution*	
		Sample	*Diluent (NaCl)*
Normal	2 µL	-	-
Decreased	2 µL	25 µL	75 µL
Increased	2 µL		

Calibration

Calibrators	S1: H_2O<br>S2-S6: Calibrator sTfR II
Calibration mode	Non-linear
Calibration frequency	Full calibration<br>• after reagent lot change<br>• after 12 weeks on-board the analyzer<br>• after 6 months when using a single reagent lot<br>• as required following quality control procedures

Calibration interval may be extended based on acceptable verification of calibration by the laboratory.

Traceability: This method has been standardized against an in-house reference preparation.

Quality control

For quality control, use control materials as listed in the "Order information" section.

In addition, other suitable control material can be used.

The control intervals and limits should be adapted to each laboratory’s individual requirements. Values obtained should fall within the defined limits. Each laboratory should establish corrective measures to be taken if values fall outside the defined limits.

Follow the applicable government regulations and local guidelines for quality control.

Calculation

cobas c systems automatically calculate the analyte concentration of each sample in the unit mg/L (mg/dL, nmol/L)

Conversion factors: mg/L x 11.8 = nmol/L[9,a)]
mg/L x 0.1 = mg/dL

a) Based on a molecular mass of 85 kDa for circulating transferrin receptor.

Limitations – interference

Criterion: Recovery within ± 0.2 mg/L (2.36 nmol/L) of initial values of samples ≤ 2 mg/L (23.6 nmol/L) and within ± 10 % for samples > 2 mg/L.

Icterus:[10] No significant interference up to an I index of 60 for conjugated and unconjugated bilirubin (approximate conjugated and unconjugated bilirubin concentration: 1026 µmol/L or 60 mg/dL).

Hemolysis:[10] No significant interference up to an H index of 1000 (approximate hemoglobin concentration: 622 µmol/L or 1000 mg/dL).

Lipemia (Intralipid):[10] No significant interference up to an L index of 2000. There is poor correlation between the L index (corresponds to turbidity) and triglycerides concentration.

Rheumatoid factors: No significant interference from rheumatoid factors up to a concentration of 1200 IU/mL.

High dose hook-effect: No false result occurs up to an sTfR concentration of 80 mg/L (944 nmol/L).

The antibodies are specific for sTfR. There is no cross-reactivity with diferrotransferrin, apotransferrin or ferritin under the assay conditions.

Drugs: No interference was found at therapeutic concentrations using common drug panels.[11,12]

For peer review only

For peer review only - http://bmjopen.bmj.com/site/about/guidelines.xhtml

0107227841190c501V1.0

STFR2

Tina-quant Soluble Transferrin Receptor II

In very rare cases, gammopathy, in particular type IgM (Waldenström's macroglobulinemia), may cause unreliable results.[13]

In very rare cases, patient samples may contain particle agglutinating proteins (e.g. heterophilic antibodies or antibodies due to abnormal immunoglobulin synthesis, such as gammopathies like MGUS* or Waldenström's macroglobulinemia) which may lead to incorrect low or high results with this assay. Correct results cannot be obtained by sample dilution and these samples should be analyzed by an alternative method.
*Monoclonal Gammopathy of unknown significance

For diagnostic purposes, the results should always be assessed in conjunction with the patient's medical history, clinical examination and other findings.

ACTION REQUIRED
Special Wash Programming: The use of special wash steps is mandatory when certain test combinations are run together on **cobas c** systems. The latest version of the carry-over evasion list can be found with the NaOHD-SMS-SmpCln1+2-SCCS Method Sheets. For further instructions refer to the operator's manual. **cobas c** 502 analyzer: All special wash programming necessary for avoiding carry-over is available via the **cobas** link, manual input is required in certain cases.

Where required, special wash/carry-over evasion programming must be implemented prior to reporting results with this test.

Limits and ranges

Measuring range

0.50-20.0 mg/L (5.9-236 nmol/L)

Determine samples having higher concentrations via the rerun function. Dilution of samples via the rerun function is a 1:4 dilution. Results from samples diluted using the rerun function are automatically multiplied by a factor of 4.

Lower limits of measurement

Limit of Blank, Limit of Detection and Limit of Quantitation

Limit of Blank	= 0.25 mg/L (2.95 nmol/L)
Limit of Detection	= 0.40 mg/L (4.72 nmol/L)
Limit of Quantitation	= 0.50 mg/L (5.90 nmol/L)

The Limit of Blank and Limit of Detection were determined in accordance with the CLSI (Clinical and Laboratory Standards Institute) EP17-A2 requirements.

The Limit of Blank is the 95th percentile value from n ≥ 60 measurements of analyte-free samples over several independent series. The Limit of Blank corresponds to the concentration below which analyte-free samples are found with a probability of 95 %.

The Limit of Detection is determined based on the Limit of Blank and the standard deviation of low concentration samples.

The Limit of Detection corresponds to the lowest analyte concentration which can be detected (value above the Limit of Blank with a probability of 95 %).

The Limit of Quantitation is the lowest analyte concentration that can be reproducibly measured with a total error of 20 %. It has been determined using low concentration sTfR samples.

Expected values

The values shown below were performed on samples from an apparently healthy population, using the Tina-quant Soluble Transferrin Receptor II assay (STFR2). The calculation is based on 165 sera (101 men, 64 women). The age range was between 22 and 83 years. The analysis of the data with the 2.5 % and the 97.5 % percentile gave a soluble transferrin receptor (sTfR) range from 1.71 mg/L (20.2 nmol/L) to 4.13 mg/L (48.7 nmol/L).

Each laboratory should investigate the transferability of the expected values to its own patient population and if necessary determine its own reference ranges.

Specific performance data

Representative performance data on the analyzers are given below. Results obtained in individual laboratories may differ.

Precision

Precision was determined using human samples and controls in accordance with the CLSI (Clinical and Laboratory Standards Institute) EP5-A3 requirements with repeatability (n = 84) and intermediate precision (2 aliquots per run, 2 runs per day, 21 days)

The following results were obtained:

Repeatability	*Mean*	*SD*	*CV*
	mg/L	*mg/L*	*%*
Control Set sTfR II L 1	2.56	0.0389	1.5
Control Set sTfR II L 2	6.91	0.0626	0.9
Human serum 1	1.21	0.0375	3.1
Human serum 2	2.00	0.0438	2.2
Human serum 3	5.27	0.0526	1.0
Human serum 4	9.23	0.108	1.2
Human serum 5	17.7	0.157	0.9

Intermediate precision	*Mean*	*SD*	*CV*
	mg/L	*mg/L*	*%*
Control Set sTfR II L 1	2.56	0.0444	1.7
Control Set sTfR II L 2	6.91	0.0732	1.1
Human serum 1	1.21	0.0388	3.2
Human serum 2	2.00	0.0475	2.4
Human serum 3	5.27	0.0675	1.3
Human serum 4	9.31	0.118	1.3
Human serum 5	17.7	0.192	1.1

Method comparison

sTfR values for human serum and plasma samples obtained on a **cobas c** 501 analyzer (y) were compared with those determined using the Soluble Transferrin Receptor assay (STFR) on a **cobas c** 501 analyzer(x).

Sample size (n) = 87

Passing/Bablok[14]	Linear regression
y = 0.987x + 0.0347 mg/L	y = 0.989x + 0.0264 mg/L
τ = 0.939	r = 0.996

The sample concentrations were between 0.660 and 19.1 mg/L.

References

1 Wick M, Pinggera W, Lehmann P, eds. Iron Metabolism, Diagnosis and Therapy of Anemias. 4th ed. Vienna/New York: Springer Verlag 1999.

2 Ferguson BJ, Skikne BS, Simpson KM, et al. Serum transferrin receptor distinguishes the anemia of chronic diseases from iron deficiency anemia. J Lab Clin Med 1992;19:385-390.

3 Baynes RD, Skikne BS, Cook JD. Circulating transferrin receptors and assessment of iron status. J Nutr Biochem 1994;5:322-330.

4 Kuiper-Kramer EPA, Huisman CMS, van Raan J, et al. Analytical and clinical implications of soluble transferrin receptors. Eur J Clin Chem Clin Biochem 1996;34:645-649.

5 Baynes RD. Assessment of iron status. Clin Biochem 1996;29:209-215.

6 Punnonen K, Irjala K, Rajamäki A. Serum transferrin receptor and its ratio to serum ferritin in the diagnosis of iron deficiency. Blood 1997;89:1052-1057.

7 Suominen P, Punnonen K, Rajamäki A, et al. Serum transferrin receptor and transferrin receptor-ferritin index identify healthy subjects with subclinical iron deficits. Blood 1998;92:2934-2939.

8 Haeckel R, ed. Evaluation methods in laboratory medicine. VCH Verlagsgesellschaft, Weinheim 1993.

9 Cook JD, Skikne BS, Baynes RD. Serum transferrin receptor. Annu Rev Med 1993;44:63-74.

10 Glick MR, Ryder KW, Jackson SA. Graphical Comparisons of Interferences in Clinical Chemistry Instrumentation. Clin Chem 1986;32:470-475.

For peer review only

For peer review only - http://bmjopen.bmj.com/site/about/guidelines.xhtml

0107227841190c501V1.0

STFR2

Tina-quant Soluble Transferrin Receptor II

cobas®

11 Breuer J. Report on the Symposium "Drug effects in Clinical Chemistry Methods". Eur J Clin Chem Clin Biochem 1996;34:385-386.

12 Sonntag O, Scholer A. Drug interference in clinical chemistry: recommendation of drugs and their concentrations to be used in drug interference studies. Ann Clin Biochem 2001;38:376-385.

13 Bakker AJ, Mücke M. Gammopathy interference in clinical chemistry assays: mechanisms, detection and prevention. Clin Chem Lab Med 2007;45(9):1240-1243.

14 Bablok W, Passing H, Bender R, et al. A general regression procedure for method transformation. Application of linear regression procedures for method comparison studies in clinical chemistry, Part III. J Clin Chem Clin Biochem 1988 Nov;26(11):783-790.

A point (period/stop) is always used in this Method Sheet as the decimal separator to mark the border between the integral and the fractional parts of a decimal numeral. Separators for thousands are not used.

Symbols

Roche Diagnostics uses the following symbols and signs in addition to those listed in the ISO 15223-1 standard (for USA: see dialog.roche.com for definition of symbols used):

CONTENT	Contents of kit
→	Volume for reconstitution
GTIN	Global Trade Item Number

COBAS, COBAS C and TINA-QUANT are trademarks of Roche.

All other product names and trademarks are the property of their respective owners.

Additions, deletions or changes are indicated by a change bar in the margin.

© 2020, Roche Diagnostics

CE

Roche Diagnostics GmbH, Sandhofer Strasse 116, D-68305 Mannheim
www.roche.com

Roche

For peer review only - http://bmjopen.bmj.com/site/about/guidelines.xhtml

ms_03737551190V14.0

Ferritin

Ferritin

cobas®

REF	Σ	SYSTEM
**03737551** 190	100	Elecsys 2010<br>MODULAR ANALYTICS E170<br>**cobas e** 411<br>**cobas e** 601<br>**cobas e** 602

English

Intended use

Immunoassay for the in vitro quantitative determination of ferritin in human serum and plasma.

The **e**lectro**c**hemi**l**uminescence **i**mmuno**a**ssay "ECLIA" is intended for use on Elecsys and **cobas e** immunoassay analyzers.

Summary

Ferritin is a macromolecule with a molecular weight of at least 440 kDa (depending on the iron content) and consists of a protein shell (apoferritin) of 24 subunits and an iron core containing an average of approx. 2500 Fe^{3+} ions (in liver and spleen ferritin).[1]

Ferritin tends to form oligomers, and when it is present in excess in the cells of the storage organs there is a tendency for condensation to semicrystalline hemosiderin to occur in the lysosomes.

At least 20 isoferritins can be distinguished with the aid of isoelectric focusing.[2] This microheterogeneity is due to differences in the contents of the acidic H and weakly basic L subunits. The basic isoferritins are responsible for the long-term iron storage function, and are found mainly in the liver, spleen, and bone marrow.[1,3]

Acidic isoferritins are found mainly in the myocardium, placenta, and tumor tissue. They have a lower iron content and presumably function as intermediaries for the transfer of iron in various syntheses.[4,5,6]

The determination of ferritin is a suitable method for ascertaining the iron metabolism situation. Determination of ferritin at the beginning of therapy provides a representative measure of the body's iron reserves. A storage deficiency in the reticulo-endothelial system (RES) can be detected at a very early stage.[7]

Clinically, a threshold value of 20 µg/L (ng/mL) has proved useful in the detection of prelatent iron deficiency. This value provides a reliable indication of exhaustion of the iron reserves that can be mobilized for hemoglobin synthesis. Latent iron deficiency is defined as a fall below the 12 µg/L (ng/mL) ferritin threshold. These two values necessitate no further laboratory elucidation, even when the blood picture is still morphologically normal. If the depressed ferritin level is accompanied by hypochromic, microcytal anemia, then manifest iron deficiency is present.[1]

When the ferritin level is elevated and the possibility of a distribution disorder can be ruled out, this is a manifestation of iron overloading in the body. 400 µg/L (ng/mL) ferritin is used as the threshold value. Elevated ferritin values are also encountered with the following tumors: acute leukemia, Hodgkin's disease and carcinoma of the lung, colon, liver and prostate. The determination of ferritin has proved to be of value in liver metastasis. Studies indicate that 76 % of all patients with liver metastasis have ferritin values above 400 µg/L (ng/mL). Reasons for the elevated values could be cell necrosis, blocked erythropoiesis or increased synthesis in tumor tissue.

Two monoclonal mouse antibodies - M-4.184 and M-3.170 - are used to form the sandwich complex in the assay.

Test principle

Sandwich principle. Total duration of assay: 18 minutes.

- 1st incubation: 10 µL of sample, a biotinylated monoclonal ferritin-specific antibody, and a monoclonal ferritin-specific antibody labeled with a ruthenium complex[a] form a sandwich complex.
- 2nd incubation: After addition of streptavidin-coated microparticles, the complex becomes bound to the solid phase via interaction of biotin and streptavidin.
- The reaction mixture is aspirated into the measuring cell where the microparticles are magnetically captured onto the surface of the electrode. Unbound substances are then removed with ProCell/ProCell M. Application of a voltage to the electrode then induces chemiluminescent emission which is measured by a photomultiplier.
- Results are determined via a calibration curve which is instrument-specifically generated by 2-point calibration and a master curve provided via the reagent barcode.

a) Tris(2,2'-bipyridyl)ruthenium(II)-complex (Ru(bpy)$_3^{2+}$)

Reagents - working solutions

The reagent rackpack is labeled as FERR.

M Streptavidin-coated microparticles (transparent cap), 1 bottle, 6.5 mL:
Streptavidin-coated microparticles 0.72 mg/mL; preservative.

R1 Anti-Ferritin-Ab~biotin (gray cap), 1 bottle, 10 mL:
Biotinylated monoclonal anti-ferritin antibody (mouse) 3.0 mg/L; phosphate buffer 100 mmol/L, pH 7.2; preservative.

R2 Anti-ferritin-Ab~Ru(bpy)$_3^{2+}$ (black cap), 1 bottle, 10 mL:
Monoclonal anti-ferritin antibody (mouse) labeled with ruthenium complex 6.0 mg/L; phosphate buffer 100 mmol/L, pH 7.2; preservative.

Precautions and warnings

For in vitro diagnostic use.
Exercise the normal precautions required for handling all laboratory reagents.
Disposal of all waste material should be in accordance with local guidelines.
Safety data sheet available for professional user on request.

Avoid foam formation in all reagents and sample types (specimens, calibrators and controls).

Reagent handling

The reagents in the kit have been assembled into a ready-for-use unit that cannot be separated.

All information required for correct operation is read in from the respective reagent barcodes.

Storage and stability

Store at 2-8 °C.

Do not freeze.

Store the Elecsys reagent kit **upright** in order to ensure complete availability of the microparticles during automatic mixing prior to use.

Stability:	
unopened at 2-8 °C	up to the stated expiration date
after opening at 2-8 °C	12 weeks
on the analyzers	6 weeks

Specimen collection and preparation

Only the specimens listed below were tested and found acceptable.

Serum collected using standard sampling tubes.

Li-, Na-heparin, K_3-EDTA and sodium citrate plasma.

When sodium citrate is used, the results must be corrected by + 10 %.

Criterion: Recovery within 90-110 % of serum value or slope 0.9-1.1 + intercept within < ± 2x analytical sensitivity (LDL) + coefficient of correlation > 0.95.

Stable for 7 days at 2-8 °C, 12 months at -20 °C.[8]

For peer review only - http://bmjopen.bmj.com/site/about/guidelines.xhtml

ms_03737551190V14.0

Ferritin

cobas®

Ferritin

The sample types listed were tested with a selection of sample collection tubes that were commercially available at the time of testing, i.e. not all available tubes of all manufacturers were tested. Sample collection systems from various manufacturers may contain differing materials which could affect the test results in some cases. When processing samples in primary tubes (sample collection systems), follow the instructions of the tube manufacturer.

Centrifuge samples containing precipitates before performing the assay.

Do not use heat-inactivated samples.

Do not use samples and controls stabilized with azide.

Ensure the samples, calibrators and controls are at 20-25 °C prior to measurement.

Due to possible evaporation effects, samples, calibrators and controls on the analyzers should be analyzed/measured within 2 hours.

Materials provided

See “Reagents – working solutions” section for reagents.

Materials required (but not provided)

- REF 03737586190, Ferritin CalSet, 4 x 1 mL
- REF 11776452122, PreciControl Tumor Marker, for 2 x 3 mL each of PreciControl Tumor Marker 1 and 2 or
  REF 05618860190, PreciControl Varia, for 2 x 3 mL each of PreciControl Varia 1 and 2
- REF 11732277122, Diluent Universal, 2 x 16 mL sample diluent or
  REF 03183971122, Diluent Universal, 2 x 36 mL sample diluent
- General laboratory equipment
- Elecsys 2010, MODULAR ANALYTICS E170 or **cobas e** analyzer

Accessories for Elecsys 2010 and **cobas e** 411 analyzers:

- REF 11662988122, ProCell, 6 x 380 mL system buffer
- REF 11662970122, CleanCell, 6 x 380 mL measuring cell cleaning solution
- REF 11930346122, Elecsys SysWash, 1 x 500 mL washwater additive
- REF 11933159001, Adapter for SysClean
- REF 11706802001, Elecsys 2010 AssayCup, 60 x 60 reaction vessels
- REF 11706799001, Elecsys 2010 AssayTip, 30 x 120 pipette tips

Accessories for MODULAR ANALYTICS E170, **cobas e** 601 and **cobas e** 602 analyzers:

- REF 04880340190, ProCell M, 2 x 2 L system buffer
- REF 04880293190, CleanCell M, 2 x 2 L measuring cell cleaning solution
- REF 03023141001, PC/CC-Cups, 12 cups to prewarm ProCell M and CleanCell M before use
- REF 03005712190, ProbeWash M, 12 x 70 mL cleaning solution for run finalization and rinsing during reagent change
- REF 12102137001, AssayTip/AssayCup Combimagazine M, 48 magazines x 84 reaction vessels or pipette tips, waste bags
- REF 03023150001, WasteLiner, waste bags
- REF 03027651001, SysClean Adapter M

Accessories for all analyzers:

- REF 11298500316, Elecsys SysClean, 5 x 100 mL system cleaning solution

Assay

For optimum performance of the assay follow the directions given in this document for the analyzer concerned. Refer to the appropriate operator’s manual for analyzer-specific assay instructions.

Resuspension of the microparticles takes place automatically prior to use. Read in the test-specific parameters via the reagent barcode. If in exceptional cases the barcode cannot be read, enter the 15-digit sequence of numbers.

Bring the cooled reagents to approx. 20 °C and place on the reagent disk (20 °C) of the analyzer. Avoid foam formation. The system automatically regulates the temperature of the reagents and the opening/closing of the bottles.

Calibration

Traceability: The Ferritin assay (REF 03737551) has been standardized against the Ferritin assay (REF 11820982). The Ferritin assay (REF 11820982) has been standardized against the Enzymun-Test Ferritin method. This in turn has been standardized against the 1st International Standard (IS) NIBSC (National Institute for Biological Standards and Control) “Reagent for Ferritin (human liver)” 80/602.

Recovery studies, including a published study,[9] to assess traceability of the Elecsys Ferritin assay to more recent international standards (2nd IS 80/578 and 3rd IS 94/572) have been conducted, with results showing very good agreement.

Every Elecsys reagent set has a barcoded label containing specific information for calibration of the particular reagent lot. The predefined master curve is adapted to the analyzer using the relevant CalSet.

Calibration frequency: Calibration must be performed once per reagent lot using fresh reagent (i.e. not more than 24 hours since the reagent kit was registered on the analyzer). Renewed calibration is recommended as follows:

- after 1 month (28 days) when using the same reagent lot
- after 7 days (when using the same reagent kit on the analyzer)
- as required: e.g. quality control findings outside the defined limits

Quality control

For quality control, use PreciControl Tumor Marker or PreciControl Varia.

In addition, other suitable control material can be used.

Controls for the various concentration ranges should be run individually at least once every 24 hours when the test is in use, once per reagent kit, and following each calibration.

The control intervals and limits should be adapted to each laboratory’s individual requirements. Values obtained should fall within the defined limits. Each laboratory should establish corrective measures to be taken if values fall outside the defined limits.

Follow the applicable government regulations and local guidelines for quality control.

Calculation

The analyzer automatically calculates the analyte concentration of each sample (either in µg/L or ng/mL).

Limitations - interference

The assay is unaffected by icterus (bilirubin < 1112 µmol/L or < 65 mg/dL), hemolysis (Hb < 0.31 mmol/L or < 0.5 g/dL), lipemia (Intralipid < 3300 mg/dL) and biotin (< 205 nmol/L or < 50 ng/mL).

Criterion: Recovery within ± 10 % of initial value.

Samples should not be taken from patients receiving therapy with high biotin doses (i.e. > 5 mg/day) until at least 8 hours following the last biotin administration.

No interference was observed from rheumatoid factors up to a concentration of 2500 IU/mL.

There is no high-dose hook effect at ferritin concentrations up to 100000 µg/L (ng/mL).

In vitro tests were performed on 19 commonly used pharmaceuticals. No interference with the assay was found.

Iron^{2+}- and iron^{3+}-ions at therapeutic concentrations do not interfere with the Elecsys Ferritin assay.

In rare cases, interference due to extremely high titers of antibodies to analyte-specific antibodies, streptavidin or ruthenium can occur. These effects are minimized by suitable test design.

For diagnostic purposes, the results should always be assessed in conjunction with the patient’s medical history, clinical examination and other findings.

Limits and ranges

Measuring range

0.500-2000 µg/L (ng/mL) (defined by the lower detection limit and the maximum of the master curve). Values below the lower detection limit are reported as < 0.500 µg/L (ng/mL). Values above the measuring range are reported as > 2000 µg/L (ng/mL) (or up to 100000 µg/L (ng/mL) for 50-fold diluted samples).

For peer review only - http://bmjopen.bmj.com/site/about/guidelines.xhtml

ms_03737551190V14.0

Ferritin

Ferritin

Lower limits of measurement

Lower detection limit of the test

Lower detection limit: 0.50 µg/L (ng/mL)

The lower detection limit represents the lowest measurable analyte level that can be distinguished from zero. It is calculated as the value lying two standard deviations above that of the lowest standard (master calibrator, standard 1 + 2 SD, repeatability study, n = 21).

Dilution

Samples with ferritin concentrations above the measuring range can be diluted with Diluent Universal. The recommended dilution is 1:50 (either automatically by the MODULAR ANALYTICS E170, Elecsys 2010 or **cobas e** analyzers or manually). The concentration of the diluted sample must be > 40 µg/L (ng/mL).

After manual dilution, multiply the result by the dilution factor.

After dilution by the analyzers, the MODULAR ANALYTICS E170, Elecsys 2010 and **cobas e** software automatically takes the dilution into account when calculating the sample concentration.

Expected values

Results of a study with the Enzymun-Test Ferritin method on samples from 224 healthy test subjects (104 women - mainly premenopausal - and 120 men) are given below. The values correspond to the 5th and 95th percentiles.[10]

Men, 20-60 years: 30-400 µg/L (ng/mL)

Women, 17-60 years: 13-150 µg/L (ng/mL)

Each laboratory should investigate the transferability of the expected values to its own patient population and if necessary determine its own reference ranges.

Specific performance data

Representative performance data on the analyzers are given below. Results obtained in individual laboratories may differ.

Precision

Precision was determined using Elecsys reagents, pooled human sera and controls in a protocol (EP5-A2) of the CLSI (Clinical and Laboratory Standards Institute): 2 runs per day in duplication each for 21 days (n = 84). The following results were obtained:

Elecsys 2010 and **cobas e** 411 analyzers					
		**Repeatability**		**Intermediate precision**	
Sample	Mean µg/L (ng/mL)	SD µg/L (ng/mL)	CV %	SD µg/L (ng/mL)	CV %
Human serum 1	1.45	0.101	7.0	0.168	11.6
Human serum 2	11.9	0.411	3.5	0.798	6.7
Human serum 3	19.2	0.780	4.1	1.47	7.7
Human serum 4	376	10.8	2.9	17.2	4.6
Human serum 5	1361	26.5	1.9	84.4	6.2
PreciControl Varia 1	134	1.96	1.5	2.75	2.1
PreciControl Varia 2	858	15.1	1.8	21.7	2.5

MODULAR ANALYTICS E170, **cobas e** 601 and **cobas e** 602 analyzers					
		**Repeatability**		**Intermediate precision**	
Sample	Mean µg/L (ng/mL)	SD µg/L (ng/mL)	CV %	SD µg/L (ng/mL)	CV %
Human serum 1	1.12	0.139	12.4	0.263	23.4
Human serum 2	12.3	0.467	3.8	0.789	6.4
Human serum 3	20.5	0.837	4.1	1.67	8.1
Human serum 4	392	8.14	2.1	16.9	4.3
Human serum 5	1449	35.6	2.5	92.8	6.4
PreciControl Varia 1	140	2.31	1.7	3.53	2.5
PreciControl Varia 2	900	14.4	1.6	25.0	2.8

Method comparison

A comparison of the Ferritin assay, REF 03737551 (y) with the Ferritin assay, REF 11820982 (x) using clinical samples gave the following correlations:

Number of samples measured: 134

Passing/Bablok[11]	Linear regression
y = 1.00x + 0.72	y = 0.99x + 4.11
τ = 0.984	r = 0.999

The sample concentrations were between approximately 2.68 and 1891 µg/L (ng/mL).

Analytical specificity

Human liver ferritin: 100 % recovery

Human spleen ferritin: 85 % recovery

Human heart ferritin: 1 % recovery

References

1 Wick M, Pinggera W, Lehmann P. Ferritin in Iron Metabolism - Diagnosis of Anemieas (second edition). Springer-Verlag 1995;ISBN 3-211-82525-8 and ISBN 0-387-82525-8.

2 Arosio P, Levi S, Gabri E, et al. Heterogeneity of ferritin II: Immunological aspects. In: Albertini A, Arosio P, Chiancone E, Drysdale J (eds). Ferritins and isoferritins as biochemical markers. Elsevier, Amsterdam 1984;33-47.

3 Kaltwasser JP, Werner E. Serumferritin: Methodische und Klinische Aspekte. Springer Verlag (1980).

4 Morikawa K, Oseko F, Morikawa S. A role for ferritin in hematopoiesis and the immune system. Leuk-Lymphoma 1995;18(5-6):429-433.

5 Borch-Iohnson B. Determination of Iron status: brief review of physiological effects on iron measures. Analyst 1995;120(3):891-903.

6 Cook JD, Skikne BS, Baynes RD. Iron deficiency: the global perspective. Adv-Exp-Med-Biol 1994;356:219-228.

7 Albertini A, Arosio P, Chiancone E, et al. (eds). Functional aspects of isoferritins. In: Jacobs A, Hodgetts J, Hoy TG. Ferritins and isoferritins as biochemical markers. Elsevier, Amsterdam 1984;113-127.

8 Guder WG, Narayanan S, Wisser H, et al. List of Analytes; Preanalytical Variables. Brochure in: Samples: From the Patient to the Laboratory. GIT-Verlag, Darmstadt 1996:14. ISBN 3-928865-22-6.

9 Blackmore S, Hamilton M, Lee A, et al. Automated immunoassay methods for ferritin: recovery studies to assess traceability to an international standard. Clin Chem Lab Med 2008;46(10):1450-1457.

10 Lotz J, Hafner G, Prellwitz W. Reference Study for Ferritin Assays. Kurzmitteilung Clin Lab 1997;43(11):993-994.

11 Bablok W, Passing H, Bender R, et al. A general regression procedure for method transformation. Application of linear regression procedures for method comparison studies in clinical chemistry, Part III. J Clin Chem Clin Biochem 1988 Nov;26(11):783-790.

For further information, please refer to the appropriate operator's manual for the analyzer concerned, the respective application sheets, the product information and the Method Sheets of all necessary components (if available in your country).

A point (period/stop) is always used in this Method Sheet as the decimal separator to mark the border between the integral and the fractional parts of a decimal numeral. Separators for thousands are not used.

For peer review only - http://bmjopen.bmj.com/site/about/guidelines.xhtml

ms_03737551190V14.0

Ferritin

Ferritin

Symbols

Roche Diagnostics uses the following symbols and signs in addition to those listed in the ISO 15223-1 standard.

CONTENT	Contents of kit
SYSTEM	Analyzers/Instruments on which reagents can be used
REAGENT	Reagent
CALIBRATOR	Calibrator
→	Volume after reconstitution or mixing

COBAS, COBAS E, ELECSYS, MODULAR and PRECICONTROL are trademarks of Roche. INTRALIPID is a trademark of Fresenius Kabi AB.

All other product names and trademarks are the property of their respective owners.

Significant additions or changes are indicated by a change bar in the margin.

© 2013, Roche Diagnostics

Roche Diagnostics GmbH, Sandhofer Strasse 116, D-68305 Mannheim
www.roche.com

For peer review only

For peer review only - http://bmjopen.bmj.com/site/about/guidelines.xhtml

05944295500V8.0

Elecsys Folate RBC

cobas®

REF	Instructions for use	Σ	SYSTEM
05944295190	05944295500	100	**cobas e** 411<br>**cobas e** 601<br>**cobas e** 602

English

System information

For **cobas e** 411 analyzer: test number 1210
For **cobas e** 601 and **cobas e** 602 analyzers: Application Code Number 272

Intended use

This assay is used for the in vitro quantitative determination of folate in erythrocytes (red blood cells, RBC). The electrochemiluminescence binding assay is intended for use on the Elecsys and **cobas e** immunoassay analyzers.

Summary

Folate belongs to the family of B-group vitamins composed of an aromatic pteridine ring linked through a methylene group to p-aminobenzoic acid and a glutamate residue. Folate (folic acid) is vital for normal cellular functions and plays an essential role in nucleic acid synthesis, methionine regeneration, shuttling and redox reactions of one-carbon units required for normal metabolism and regulation.[1,2]

The folate metabolism can be exemplified as a cycle, where folate facilitates the transfer of one-carbon units from one molecule to another required in various biochemical reactions: for example, tetrahydrofolate (THF) accepts a single carbon unit from serine, which is reduced in a number of steps to 5-methyltetrahydrofolate (5-MTHF). 5-MTHF gives its methyl group to homocysteine, which is - with involvement of methionine synthase and vitamin B12 - enzymatically converted to methionine. The resulting THF starts again the cycle of methyl group synthesis. From methionine, the methyl groups are transferred to S-adenosylmethionine (SAM).[3] SAM serves as a methyl group donor in several methylation reactions, like DNA, RNA and protein methylation.[1]

The methionine cycle is highly sensitive to folate deficiency: with a low folate status, the ability of the cell to re-methylate homocysteine is impaired and this results in increased homocysteine concentrations in plasma.[2]

Folate also plays an essential role in the synthesis of purine and pyrimidine precursors of nucleic acids. Altered distribution of methyl groups and impaired DNA synthesis play an essential role in the development of cancers. Abnormal folate status has also been linked with the development of diseases like cardiovascular diseases, neural tube defects, cleft lip and palate, late pregnancy complications, neurodegenerative and psychiatric disorders.[1,2]

Folate belongs to the group of essential vitamins, i.e. it cannot be synthesized by the human organism and therefore must be absorbed from diet. Primary sources of folates are green and leafy vegetables, sprouts, fruits, brewer's yeast and liver.[1,2]

Folate deficiency can be caused by decreased nutritional intake, poor absorption of ingested folate in the intestine or increased demand of folate, for example during physical activity or pregnancy. Deficiency of folate can also be a result of liver diseases or impaired folate metabolism due to genetic defects or drug interactions.[2]

A clinical manifestation of both folate and vitamin B12 deficiency is the so called megaloblastic (macrocytic) anemia: due to the affected DNA synthesis and cell maturation, especially involving the cells of erythropoiesis, the total count of erythrocytes is significantly reduced. The hemoglobin synthesis capacity however is normal, which leads to abnormally large erythrocyte precursors ("macrocytes" or "megaloblasts"), which have an elevated hemoglobin content ("hyperchromic anemia").[3,4]

Serum folate concentrations may be affected by recent folate intakes, whereas red blood cell (RBC) folate is a measure of the folate intake across the 90-120 days lifespan of erythrocytes. Thus, folate concentrations in RBC give a more accurate picture of a patient's underlying folate status than serum folate concentrations, and are considered by experts as the better measure for folate status.[5]

Because vitamin B12 and folate are closely interrelated in the cellular one-carbon unit metabolism, and also hematologic and clinical consequences of the two vitamin deficiency states might be similar, it is advisable to determine both parameters simultaneously in patients with the relevant symptoms of vitamin deficiency.[3,4]

Test principle

Competition principle. Total duration of assay: 27 minutes.

Whole blood treated with anticoagulants (heparin or EDTA) is mixed with ascorbic acid solution and incubated for approximately 90 minutes at 20-25 °C. Lysis of the erythrocytes takes place, with liberation and stabilization of the intracellular folate. The resulting hemolysate sample is then used for subsequent measurement.

- 1st incubation: By incubating 25 µL of hemolysate sample with the folate pretreatment reagents 1 and 2, bound folate is released from endogenous folate binding proteins.
- 2nd incubation: By incubating the pretreated sample with the ruthenium labeled folate binding protein, a folate complex is formed, the amount of which is dependent upon the analyte concentration in the sample.
- 3rd incubation: After addition of streptavidin-coated microparticles and folate labeled with biotin, the unbound sites of the ruthenium labeled folate binding protein become occupied, with formation of a ruthenium labeled folate binding protein-folate biotin complex. The entire complex becomes bound to the solid phase via interaction of biotin and streptavidin.
- The reaction mixture is aspirated into the measuring cell where the microparticles are magnetically captured onto the surface of the electrode. Unbound substances are then removed with ProCell/ProCell M. Application of a voltage to the electrode then induces chemiluminescent emission which is measured by a photomultiplier.
- Results are determined via a calibration curve which is instrument-specifically generated by 2-point calibration and a master curve provided via the reagent barcode or e-barcode.

Reagents - working solutions

The reagent rackpack (M, R1, R2) is labeled as RBC-FOL.

PT1 Pretreatment reagent 1 (white cap), 1 bottle, 4 mL:
Sodium 2-mercaptoethanesulfonate (MESNA) 40 g/L, pH 5.5.

PT2 Pretreatment reagent 2 (gray cap), 1 bottle, 5 mL:
Sodium hydroxide 25 g/L.

M Streptavidin-coated microparticles (transparent cap), 1 bottle, 6.5 mL:
Streptavidin-coated microparticles 0.72 mg/mL; preservative.

R1 Folate binding protein~Ru(bpy)$_3^{2+}$ (gray cap), 1 bottle, 9 mL:
Ruthenium labeled folate binding protein 75 µg/L; human serum albumin (stabilizer); borate/phosphate/citrate buffer 70 mmol/L, pH 5.5; preservative.

R2 Folate~biotin (black cap), 1 bottle, 8 mL:
Biotinylated folate 17 µg/L; biotin 120 µg/L; human serum albumin (stabilizer); borate buffer 100 mmol/L, pH 9.0; preservative.

Precautions and warnings

For in vitro diagnostic use.
Exercise the normal precautions required for handling all laboratory reagents.
Disposal of all waste material should be in accordance with local guidelines.
Safety data sheet available for professional user on request.

This kit contains components classified as follows in accordance with the Regulation (EC) No. 1272/2008:

2-methyl-2H-isothiazol-3-one hydrochloride

EUH 208 May produce an allergic reaction.

For peer review only

For peer review only - http://bmjopen.bmj.com/site/about/guidelines.xhtml

05944295500V8.0

Elecsys Folate RBC

cobas®

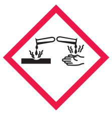

Danger

H290 May be corrosive to metals.

H314 Causes severe skin burns and eye damage.

Prevention:

P280 Wear protective gloves/ protective clothing/ eye protection/ face protection.

Response:

P301 + P330 + P331 IF SWALLOWED: Rinse mouth. Do NOT induce vomiting.

P303 + P361 + P353 IF ON SKIN (or hair): Take off immediately all contaminated clothing. Rinse skin with water.

P304 + P340 + P310 IF INHALED: Remove person to fresh air and keep comfortable for breathing. Immediately call a POISON CENTER/ doctor.

P305 + P351 + P338 + P310 IF IN EYES: Rinse cautiously with water for several minutes. Remove contact lenses, if present and easy to do. Continue rinsing. Immediately call a POISON CENTER/ doctor.

P390 Absorb spillage to prevent material damage.

Product safety labeling follows EU GHS guidance.

Contact phone: all countries: +49-621-7590

All human material should be considered potentially infectious. All products derived from human blood are prepared exclusively from the blood of donors tested individually and shown to be free from HBsAg and antibodies to HCV and HIV. The testing methods used assays approved by the FDA or cleared in compliance with the European Directive 98/79/EC, Annex II, List A.

However, as no testing method can rule out the potential risk of infection with absolute certainty, the material should be handled with the same level of care as a patient specimen. In the event of exposure, the directives of the responsible health authorities should be followed.[6,7]

Avoid foam formation in all reagents and sample types (specimens, calibrators and controls).

Reagent handling

The reagents in the kit have been assembled into a ready-for-use unit that cannot be separated.

All information required for correct operation is read in from the respective reagent barcodes.

Storage and stability

Store at 2-8 °C.

Do not freeze.

Store the Elecsys reagent kit **upright** in order to ensure complete availability of the microparticles during automatic mixing prior to use.

Stability:	
unopened at 2-8 °C	up to the stated expiration date
after opening at 2-8 °C	8 weeks
on the analyzers	2 weeks

Specimen collection and preparation

Only the specimens listed below were tested and found acceptable.

Hemolysate prepared from whole blood treated with anticoagulants (Na-heparin or K_3-EDTA).

- *For the determination of folate in RBC:* Determine hematocrit in whole blood samples and record the value.
- *Preparation of the hemolysate sample*

  Mix 3.0 mL of Folate RBC Hemolyzing Reagent (ascorbic acid solution, 0.2 %) and 100 µL of well-mixed whole blood, avoiding foam formation. Incubate with closed caps for 90 ± 15 minutes at 20-25 °C.

Stability:

Whole blood: 2 hours at 20-25 °C[8], 24 hours at 2-8 °C, 1 month at -20 °C (± 5 °C) (only EDTA-blood).

Hemolysate sample: 1 month at -20 °C (± 5 °C), freeze only once.

The sample types listed were tested with a selection of sample collection tubes that were commercially available at the time of testing, i.e. not all available tubes of all manufacturers were tested. Sample collection systems from various manufacturers may contain differing materials which could affect the test results in some cases. When processing samples in primary tubes (sample collection systems), follow the instructions of the tube manufacturer.

Samples should not subsequently be altered with additives (biocides, anti-oxidants or substances possibly changing the pH of the sample) in order to avoid erroneous folate recovery.

Ensure the samples, calibrators and controls are at 20-25 °C prior to measurement.

Due to possible evaporation effects, samples, calibrators and controls on the analyzers should be analyzed/measured within 2 hours.

If measurements cannot be carried out within 2 hours please store the hemolysate sample at -20 °C (± 5 °C).

Materials provided

See “Reagents – working solutions” section for reagents.

Materials required (but not provided)

- REF 05944309190, Folate RBC CalSet, for 4 x 1.0 mL
- REF 05944317190, Folate RBC Hemolyzing Reagent kit for 4 x 200 mL, contains ascorbic acid
- General laboratory equipment
- **cobas e** analyzer

Additional materials for the **cobas e** 411 analyzer:

- REF 11662988122, ProCell, 6 x 380 mL system buffer
- REF 11662970122, CleanCell, 6 x 380 mL measuring cell cleaning solution
- REF 11930346122, Elecsys SysWash, 1 x 500 mL washwater additive
- REF 11933159001, Adapter for SysClean
- REF 11706802001, AssayCup, 60 x 60 reaction cups
- REF 11706799001, AssayTip, 30 x 120 pipette tips
- REF 11800507001, Clean-Liner

Additional materials for **cobas e** 601 and **cobas e** 602 analyzers:

- REF 04880340190, ProCell M, 2 x 2 L system buffer
- REF 04880293190, CleanCell M, 2 x 2 L measuring cell cleaning solution
- REF 03023141001, PC/CC-Cups, 12 cups to prewarm ProCell M and CleanCell M before use
- REF 03005712190, ProbeWash M, 12 x 70 mL cleaning solution for run finalization and rinsing during reagent change
- REF 03004899190, PreClean M, 5 x 600 mL detection cleaning solution
- REF 12102137001, AssayTip/AssayCup, 48 magazines x 84 reaction cups or pipette tips, waste bags
- REF 03023150001, WasteLiner, waste bags
- REF 03027651001, SysClean Adapter M

Additional materials for all analyzers:

- REF 11298500316, ISE Cleaning Solution/Elecsys SysClean, 5 x 100 mL system cleaning solution

For peer review only - http://bmjopen.bmj.com/site/about/guidelines.xhtml

05944295500V8.0

Elecsys Folate RBC

cobas®

Assay

The well-mixed hemolysate sample is placed in the sample zone of the analyzer and recorded by entering the sample identification data. Complete determinations on the analyzer within 2 hours after finalizing the preparation of the hemolysate sample.

For optimum performance of the assay follow the directions given in this document for the analyzer concerned. Refer to the appropriate operator's manual for analyzer-specific assay instructions.

Resuspension of the microparticles takes place automatically prior to use. Read in the test-specific parameters via the reagent barcode. If in exceptional cases the barcode cannot be read, enter the 15-digit sequence of numbers.

cobas e 601 and **cobas e** 602 analyzers: PreClean M solution is necessary.

Bring the cooled reagents to approximately 20 °C and place on the reagent disk (20 °C) of the analyzer. Avoid foam formation. The system automatically regulates the temperature of the reagents and the opening/closing of the bottles.

Calibration

Traceability: This method has been standardized against the Elecsys Folate III assay (REF 04476433190)/RBC application.

The standardization of the Elecsys Folate RBC assay includes the volume correction to account for the preparation of hemolysate sample (1:31 vol/vol).

Every Elecsys reagent set has a barcoded label containing specific information for calibration of the particular reagent lot. The predefined master curve is adapted to the analyzer using the relevant CalSet.

Calibration frequency: Calibration must be performed once per reagent lot using fresh reagent (i.e. not more than 24 hours since the reagent kit was registered on the analyzer).

Calibration interval may be extended based on acceptable verification of calibration by the laboratory.

Renewed calibration is recommended as follows:

- after 1 month (28 days) when using the same reagent lot
- after 7 days (when using the same reagent kit on the analyzer)
- as required: e.g. quality control findings outside the defined limits

Quality control

For quality control, use commercially available whole blood control material.

Controls for the various concentration ranges should be run individually at least once every 24 hours when the test is in use, once per reagent kit, and following each calibration.

The control intervals and limits should be adapted to each laboratory's individual requirements. Values obtained should fall within the defined limits. Each laboratory should establish corrective measures to be taken if values fall outside the defined limits.

If necessary, repeat the measurement of the samples concerned.

Follow the applicable government regulations and local guidelines for quality control.

Calculation

1. Whole blood folate (from hemolysate sample)

The standardization of the Elecsys Folate RBC assay includes the volume correction to account for the preparation of hemolysate sample (1:31 vol/vol).

The analyzer automatically calculates the analyte concentration of each sample in nmol/L or ng/mL.

Conversion factors: nmol/L x 0.44 = ng/mL

ng/mL x 2.27 = nmol/L

2. RBC folate

To calculate the folate concentration in the erythrocyte fraction of the sample **(RBC folate)**, the predetermined sample specific hematocrit value must be taken into account using the following equation:

$$\text{RBC folate} = \frac{\text{analyzer result}}{\text{\% hematocrit}} \times 100$$

Limitations - interference

The assay is unaffected by icterus (bilirubin < 564 µmol/L or < 33 mg/dL), lipemia (Intralipid < 1500 mg/dL), biotin (< 86.1 nmol/L or < 21 ng/mL), IgG < 16 g/L and IgA < 4.0 g/L.

Criterion: Recovery within ± 10 % of initial value with samples > 155 ng/mL and ≤ ± 15.5 ng/mL with samples ≤ 155 ng/mL.

Samples should not be taken from patients receiving therapy with high biotin doses (i.e. > 5 mg/day) until at least 8 hours following the last biotin administration.

No interference was observed from rheumatoid factors up to a concentration of 1000 IU/mL.

In vitro tests were performed on 16 commonly used pharmaceuticals and in addition on human erythropoietin. No interference with the assay was found.

It is contraindicated to measure samples of patients receiving therapy with certain pharmaceuticals, e.g. methotrexate or leucovorin, because of the cross-reactivity of folate binding protein with these compounds.

In rare cases, interference due to extremely high titers of antibodies to analyte-specific antibodies, streptavidin or ruthenium can occur. These effects are minimized by suitable test design.

For diagnostic purposes, the results should always be assessed in conjunction with the patient's medical history, clinical examination and other findings.

In rare cases, samples with low erythrocyte folate concentration, but high serum folate concentration can occur. In these cases, a correction of the folate concentration in erythrocytes by the serum folate concentration with the following equation is recommended*:

* expected values can be used as an indicator for high serum folate concentration

Corrected RBC folate concentration =

$$\text{RBC folate concentration} - \left(\text{serum folate concentration} \times \frac{100 - \text{\% hematocrit}}{\text{\% hematocrit}}\right)$$

Example

RBC folate concentration: 241 (ng/mL RBC);

serum folate concentration: 10.5 (ng/mL S);

hematocrit measured (%) = 45

Corrected RBC folate concentration =

$$241 \text{ ng/mL RBC} - \left(10.5 \text{ ng/mL S} \times \frac{100 - 45}{45}\right) = 228 \text{ ng/mL RBC}$$

Limits and ranges

Measuring range

120-620 ng/mL or 272-1407 nmol/L (defined by the Limit of Quantitation and the maximum of the master curve). Values below the Limit of Quantitation are reported as < 120 ng/mL (< 272 nmol/L). Values above the measuring range are reported as > 620 ng/mL (> 1407 nmol/L). Values are not corrected for the sample hematocrit.

Lower limits of measurement

Limit of Blank, Limit of Detection and Limit of Quantitation:

Limit of Blank = 20.0 ng/mL

Limit of Detection = 46.5 ng/mL

Limit of Quantitation = 120 ng/mL

The Limit of Blank, Limit of Detection and Limit of Quantitation were determined in accordance with the CLSI (Clinical and Laboratory Standards Institute) EP17-A requirements.

The Limit of Blank is the 95th percentile value from n ≥ 60 measurements of analyte-free samples over several independent series. The Limit of Blank corresponds to the concentration below which analyte-free samples are found with a probability of 95 %.

The Limit of Detection is determined based on the Limit of Blank and the standard deviation of low concentration samples. The Limit of Detection corresponds to the lowest analyte concentration which can be detected (value above the Limit of Blank with a probability of 95 %).

The Limit of Quantitation is defined as the lowest amount of analyte in a sample that can be accurately quantitated with a total allowable relative

For peer review only - http://bmjopen.bmj.com/site/about/guidelines.xhtml

05944295500V8.0

Elecsys Folate RBC

cobas®

error of ≤ 30 %. It has been determined using low concentration folate samples.

Dilution

Hemolysate samples with folate concentrations above the measuring range can be diluted manually with Elecsys Folate RBC Hemolyzing Reagent (ascorbic acid solution, 0.2 %). The recommended dilution is 1:2. The concentration of the diluted sample must be > 265 ng/mL or > 602 nmol/L. After manual dilution, multiply the results by the dilution factor 2.

Expected values

The values shown below were measured on samples from an apparently healthy population, using the Elecsys Folate III/RBC application. The values can be applied for the Elecsys Folate RBC assay on all Elecsys and **cobas e** analyzers. The calculation is based on 290 sera (96 men, 194 women) from an European population. The age range was between 18 and 65 years. Pregnant or lactating women were excluded. The reference population was selected according to normal homocysteine values. The following values were obtained:

Whole blood folate (from hemolysate samples)					
	N	Median		2.5^{th}-97.5^{th} percentile	
		nmol/L	ng/mL	nmol/L	ng/mL
Europe	290	673	296	481-1212	212-534

The measured hematocrit value in this study showed a range from 37.1-46.1 %.

RBC folate (folate in erythrocyte fraction)					
	N	Median		2.5^{th}-97.5^{th} percentile	
		nmol/L	ng/mL	nmol/L	ng/mL
Europe	290	1657	730	1187-2854	523-1257

If pathologically low hematocrit values are considered for calculation of RBC folate in the erythrocyte fraction, elevated RBC folate concentrations may be observed. No medical conclusion should be based on the calculation considering hematocrit values in such cases. Instead, whole blood folate results (from hemolysate samples) and suitable expected values may be used.

Each laboratory should investigate the transferability of the expected values to its own patient population and if necessary determine its own reference ranges.

Specific performance data

Representative performance data on the analyzers are given below. Results obtained in individual laboratories may differ.

Precision

Precision was determined using Elecsys reagents and hemolysate samples in a protocol (EP5-A2) of the CLSI (Clinical and Laboratory Standards Institute): 2 runs per day in duplicate each for 21 days (n = 84). Results are given as whole blood folate (from hemolysate sample). The following results were obtained:

**cobas e** 411 analyzer								
			**Repeatability**			**Intermediate precision**		
Sample	Mean		SD		CV	SD		CV
	nmol/L	ng/mL	nmol/L	ng/mL	%	nmol/L	ng/mL	%
HL[a)] 1	154	68.0	11.7	5.17	7.6	21.9	9.65	14.2
HL 2	352	155	17.5	7.73	5.0	27.7	12.2	7.9
HL 3	618	272	25.4	11.2	4.1	38.4	16.9	6.2
HL 4	1195	527	38.8	17.1	3.3	56.3	24.8	4.7

a) HL = Hemolysate

**cobas e** 601 and **cobas e** 602 analyzers								
			**Repeatability**			**Intermediate precision**		
Sample	Mean		SD		CV	SD		CV
	nmol/L	ng/mL	nmol/L	ng/mL	%	nmol/L	ng/mL	%
HL 1	138	61.0	12.1	5.31	8.8	14.3	6.32	10.4
HL 2	434	191	26.1	11.5	6.0	28.4	12.5	6.5
HL 3	586	258	32.0	14.1	5.5	34.3	15.1	5.9
HL 4	1317	580	29.1	12.8	2.2	44.7	19.7	3.4

Method comparison

a) A comparison of the Elecsys Folate RBC assay (calibrated with Folate RBC CalSet; y) and the Elecsys Folate III/RBC application (calibrated with Folate III CalSet; x) using hemolyzed clinical samples gave the following correlations (ng/mL). Results are given as whole blood folate (from hemolysate sample).

Number of samples measured: 187

Passing/Bablok[9]	Linear regression
y = 1.02x - 14.1	y = 1.00x - 12.0
τ = 0.869	r = 0.985

The sample concentrations were between 151 and 551 ng/mL (343 and 1251 nmol/L).

b) A comparison of the Elecsys Folate RBC assay on the MODULAR ANALYTICS E170 analyzer (y) with the Elecsys Folate RBC assay on the Elecsys 2010 analyzer (x) (both tests have been calibrated with Folate RBC CalSet) using hemolyzed clinical samples gave the following correlations (ng/mL). Results are given as whole blood folate (from hemolysate sample).

Number of samples measured: 187

Passing/Bablok	Linear regression
y = 1.04x + 1.94	y = 1.02x + 8.07
τ = 0.814	r = 0.970

The sample concentrations were between 137 and 557 ng/mL (311 and 1264 nmol/L).

References

1 Nazki FH, Sameer AS, Ganaie BA. Folate: Metabolism, genes, polymorphisms and the associated diseases. Gene 2014;533(1):11-20.

2 Scaglione F, Panzavolta G. Folate, folic acid and 5-methyltetrahydrofolate are not the same thing. Xenobiotica 2014;44(5):480-488.

3 Reynolds EH. The neurology of folic acid deficiency. Handb Clin Neurol 2014;120:927-43.

4 Wick M, Pinggera W, Lehmann P. Clinical Aspects and Laboratory. Iron metabolism, Anemias. Springer Verlag, Wien, New York, 6th edition 2011:41-42.

5 Yetley EA, Pfeiffer CM, Phinney KW, et al. Biomarkers of folate status in NHANES: a roundtable summary. Am J Clin Nutr 2011;94(1):303S-312S.

6 Occupational Safety and Health Standards: Bloodborne pathogens. (29 CFR Part 1910.1030). Fed. Register.

7 Directive 2000/54/EC of the European Parliament and Council of 18 September 2000 on the protection of workers from risks related to exposure to biological agents at work.

8 Eijsden M, van der Wal MF, Hornstra G, et al. Can whole blood samples be stored over 24 hours without compromising stability of C-Reactive Protein, Retinol, Ferritin, Folic Acid and Fatty Acids in Epidemiology Research? Clin Chem 2005;51(1):230-232.

9 Bablok W, Passing H, Bender R, et al. A general regression procedure for method transformation. Application of linear regression procedures for method comparison studies in clinical chemistry, Part III. J Clin Chem Clin Biochem 1988 Nov;26(11):783-790.

For peer review only - http://bmjopen.bmj.com/site/about/guidelines.xhtml

05944295500V8.0

Elecsys Folate RBC

cobas®

For further information, please refer to the appropriate operator's manual for the analyzer concerned, the respective application sheets, the product information and the Method Sheets of all necessary components (if available in your country).

A point (period/stop) is always used in this Method Sheet as the decimal separator to mark the border between the integral and the fractional parts of a decimal numeral. Separators for thousands are not used.

Symbols

Roche Diagnostics uses the following symbols and signs in addition to those listed in the ISO 15223-1 standard (for USA: see dialog.roche.com for definition of symbols used):

CONTENT	Contents of kit
SYSTEM	Analyzers/Instruments on which reagents can be used
REAGENT	Reagent
CALIBRATOR	Calibrator
→	Volume after reconstitution or mixing
GTIN	Global Trade Item Number

COBAS, COBAS E, ELECSYS and PRECICONTROL are trademarks of Roche. INTRALIPID is a trademark of Fresenius Kabi AB.

All other product names and trademarks are the property of their respective owners.

Additions, deletions or changes are indicated by a change bar in the margin.

© 2020, Roche Diagnostics

CE

Roche Diagnostics GmbH, Sandhofer Strasse 116, D-68305 Mannheim
www.roche.com

Roche

For peer review only - http://bmjopen.bmj.com/site/about/guidelines.xhtml

0004628918190c501V12.0

CRPHS

cobas®

Cardiac C-Reactive Protein (Latex) High Sensitive

Order information

REF	CONTENT		Analyzer(s) on which **cobas c** pack(s) can be used
**04628918** 190	Cardiac C-Reactive Protein (Latex) High Sensitive (300 tests)	System-ID 07 6866 9	Roche/Hitachi **cobas c** 311, **cobas c** 501/502
**11355279** 216	Calibrator f.a.s. Proteins (5 x 1 mL)	Code 656	
**11355279** 160	Calibrator f.a.s. Proteins (5 x 1 mL, for USA)	Code 656	
**20766321** 322	CRP T Control N (5 x 0.5 mL)	Code 235	
**10557897** 122	Precinorm Protein (3 x 1 mL)	Code 302	
**10557897** 160	Precinorm Protein (3 x 1 mL, for USA)	Code 302	
**05117003** 190	PreciControl ClinChem Multi 1 (20 x 5 mL)	Code 391	
**05947626** 190	PreciControl ClinChem Multi 1 (4 x 5 mL)	Code 391	
**05947626** 160	PreciControl ClinChem Multi 1 (4 x 5 mL, for USA)	Code 391	
**04489357** 190	Diluent NaCl 9 % (50 mL)	System-ID 07 6869 3	

English

System information

For **cobas c** 311/501 analyzers:

CRPHS: ACN 217

For **cobas c** 502 analyzer:

CRPHS: ACN 8217

Intended use

In vitro test for the quantitative determination of C-reactive protein (CRP) in human serum and plasma on Roche/Hitachi **cobas c** systems.
Measurement of CRP is of use for the detection and evaluation of inflammatory disorders and associated diseases, infection and tissue injury. Highly sensitive measurement of CRP may also be used as an aid in the assessment of the risk of future coronary heart disease. When used as an adjunct to other laboratory evaluation methods of acute coronary syndromes, it may also be an additional independent indicator of recurrent event prognosis in patients with stable coronary disease or acute coronary syndrome.

Summary[1,2,3,4,5,6,7,8,9,10,11,12,13,14,15,16,17,18,19,20,21]

C-reactive protein is the classic acute phase protein in inflammatory reactions. It is synthesized by the liver and consists of five identical polypeptide chains that form a five-member ring having a molecular weight of 105000 daltons. CRP is the most sensitive of the acute phase reactants and its concentration increases rapidly during inflammatory processes. Complexed CRP activates the complement system beginning with C1q. CRP then initiates opsonization and phagocytosis of invading cells, but its main function is to bind and detoxify endogenous toxic substances produced as a result of tissue damage.

CRP assays are used to detect systemic inflammatory processes (apart from certain types of inflammation such as SLE and Colitis ulcerosa); to assess treatment of bacterial infections with antibiotics; to detect intrauterine infections with concomitant premature amniorrhexis; to differentiate between active and inactive forms of disease with concurrent infection, e.g. in patients suffering from SLE or Colitis ulcerosa; to therapeutically monitor rheumatic disease and assess anti-inflammatory therapy; to determine the presence of post-operative complications at an early stage, such as infected wounds, thrombosis and pneumonia, and to distinguish between infection and bone marrow transplant rejection.

Sensitive CRP measurements have been used and discussed for early detection of infection in pediatrics and risk assessment of coronary heart disease. Several studies came to the conclusion that the highly sensitive measurement of CRP could be used as a marker to predict the risk of coronary heart disease in apparently healthy persons and as an indicator of recurrent event prognosis. Increases in CRP values are non-specific and should not be interpreted without a complete clinical history. The American Heart Association and the Centers for Disease Control and Prevention have made several recommendations concerning the use of high sensitivity C-Reactive Protein (hsCRP) in cardiovascular risk assessment.[21] Testing for any risk assessment should not be performed while there is an indication of infection, systemic inflammation or trauma. Patients with persistently unexplained hsCRP levels above 10 mg/L (95.2 nmol/L) should be evaluated for non-cardiovascular etiologies. When using hsCRP to assess the risk of coronary heart disease, measurements should be made on metabolically stable patients and compared to previous values.

Optimally, the average of hsCRP results repeated two weeks apart should be used for risk assessment. Screening the entire adult population for hsCRP is not recommended, and hsCRP is not a substitute for traditional cardiovascular risk factors. Acute coronary syndrome management should not depend solely on hsCRP measurements. Similarly, application of secondary prevention measures should be based on global risk assessment and not solely on hsCRP measurements. Serial measurements of hsCRP should not be used to monitor treatment.

Various assay methods are available for CRP determination, such as nephelometry and turbidimetry. The Roche CRP assay is based on the principle of particle-enhanced immunological agglutination.

Test principle[22,23]

Particle enhanced immunoturbidimetric assay.

Human CRP agglutinates with latex particles coated with monoclonal anti-CRP antibodies. The precipitate is determined turbidimetrically.

Reagents - working solutions

R1 TRIS buffer with bovine serum albumin and immunoglobulins (mouse); preservative; stabilizers

R2 Latex particles coated with anti-CRP (mouse) in glycine buffer; preservative; stabilizers

R1 is in position B and R2 is in position C.

Precautions and warnings

For in vitro diagnostic use.
Exercise the normal precautions required for handling all laboratory reagents.
Disposal of all waste material should be in accordance with local guidelines.
Safety data sheet available for professional user on request.

For USA: Caution: Federal law restricts this device to sale by or on the order of a physician.

Reagent handling

Ready for use

Mix **cobas c** pack well before placing on the analyzer.

Carefully invert reagent container several times prior to use to ensure that the reagent components are mixed.

Storage and stability

CRPHS

Shelf life at 2-8 °C:	See expiration date on **cobas c** pack label.
On-board in use and refrigerated on the analyzer:	12 weeks

Diluent NaCl 9 %

Shelf life at 2-8 °C:	See expiration date on **cobas c** pack label.
On-board in use and refrigerated on the analyzer:	12 weeks

For peer review only - http://bmjopen.bmj.com/site/about/guidelines.xhtml

0004628918190c501V12.0

CRPHS

cobas®

Cardiac C-Reactive Protein (Latex) High Sensitive

Specimen collection and preparation

For specimen collection and preparation only use suitable tubes or collection containers.

Only the specimens listed below were tested and found acceptable.
Serum.
Plasma: Li-heparin and K_2-EDTA plasma

The sample types listed were tested with a selection of sample collection tubes that were commercially available at the time of testing, i.e. not all available tubes of all manufacturers were tested. Sample collection systems from various manufacturers may contain differing materials which could affect the test results in some cases. When processing samples in primary tubes (sample collection systems), follow the instructions of the tube manufacturer.

Centrifuge samples containing precipitates before performing the assay.

See the limitations and interferences section for details about possible sample interferences.

Sample stability claims were established by experimental data by the manufacturer or based on reference literature and only for the temperatures/time frames as stated in the method sheet. It is the responsibility of the individual laboratory to use all available references and/or its own studies to determine specific stability criteria for its laboratory.

Stability:[24]	11 days at 15-25 °C
	2 months at 2-8 °C
	3 years at (-15)-(-25) °C

Materials provided

See “Reagents – working solutions” section for reagents.

Materials required (but not provided)

- See “Order information” section
- General laboratory equipment

Assay

For optimum performance of the assay follow the directions given in this document for the analyzer concerned. Refer to the appropriate operator’s manual for analyzer-specific assay instructions.

The performance of applications not validated by Roche is not warranted and must be defined by the user.

Application for serum and plasma

cobas c 311 test definition

Assay type	Rate A	
Reaction time / Assay points	10/7-57	
Wavelength (sub/main)	– /546 nm	
Reaction direction	Increase	
Units	mg/L (nmol/L, mg/dL)	
Reagent pipetting		Diluent (H_2O)
R1	82 µL	42 µL
R2	28 µL	20 µL

Sample volumes	*Sample*	*Sample dilution*	
		Sample	*Diluent (NaCl)*
Normal	6 µL	–	–
Decreased	6 µL	10 µL	140 µL
Increased	6 µL	–	–

cobas c 501 test definition

Assay type	Rate A	
Reaction time / Assay points	10/12-70	
Wavelength (sub/main)	– /546 nm	
Reaction direction	Increase	
Units	mg/L (nmol/L, mg/dL)	
Reagent pipetting		Diluent (H_2O)
R1	82 µL	42 µL
R2	28 µL	20 µL

Sample volumes	*Sample*	*Sample dilution*	
		Sample	*Diluent (NaCl)*
Normal	6 µL	–	–
Decreased	6 µL	10 µL	140 µL
Increased	6 µL	–	–

cobas c 502 test definition

Assay type	Rate A	
Reaction time / Assay points	10/12-70	
Wavelength (sub/main)	– /546 nm	
Reaction direction	Increase	
Units	mg/L (nmol/L, mg/dL)	
Reagent pipetting		Diluent (H_2O)
R1	82 µL	42 µL
R2	28 µL	20 µL

Sample volumes	*Sample*	*Sample dilution*	
		Sample	*Diluent (NaCl)*
Normal	6 µL	–	–
Decreased	6 µL	10 µL	140 µL
Increased	12 µL	–	–

Calibration

Calibrators: S1: H_2O
S2: C.f.a.s. Proteins

Multiply the lot-specific C.f.a.s. Proteins calibrator value by the factors below to determine the standard concentrations for the 6-point calibration curve:

S2: 0.0125	S5: 0.100
S3: 0.0250	S6: 0.200
S4: 0.0500	

Calibration mode: Line Graph

Calibration frequency: Full calibration
- after reagent lot change
- as required following quality control procedures

Calibration interval may be extended based on acceptable verification of calibration by the laboratory.

Traceability: This method has been standardized against the reference preparation of the IRMM (Institute for Reference Materials and Measurements) BCR470/CRM470 (RPPHS - Reference Preparation for Proteins in Human Serum).[25]

Quality control

For quality control, use control materials as listed in the "Order information" section.

In addition, other suitable control material can be used.

The control intervals and limits should be adapted to each laboratory’s individual requirements. Values obtained should fall within the defined

For peer review only - http://bmjopen.bmj.com/site/about/guidelines.xhtml

0004628918190c501V12.0

CRPHS

cobas®

Cardiac C-Reactive Protein (Latex) High Sensitive

limits. Each laboratory should establish corrective measures to be taken if values fall outside the defined limits.

Follow the applicable government regulations and local guidelines for quality control.

Calculation

Roche/Hitachi **cobas c** systems automatically calculate the analyte concentration of each sample.

Conversion factors:	mg/L x 9.52 = nmol/L
	mg/L x 0.1 = mg/dL

Limitations - interference

| Criterion: Recovery within ± 10 % of initial values at CRP levels of 1.0 mg/L.

Icterus:[26] No significant interference up to an I index of 60 for conjugated bilirubin and unconjugated bilirubin (approximate conjugated and unconjugated bilirubin concentration: 60 mg/dL or 1026 µmol/L).

Hemolysis:[26] No significant interference up to an H index of 1000 (approximate hemoglobin concentration: 622 µmol/L or 1000 mg/dL).

Lipemia (Intralipid):[26] No significant interference up to an L index of 600. There is poor correlation between the L index (corresponds to turbidity) and triglycerides concentration.

Rheumatoid factors: No significant interference from rheumatoid factors up to a concentration of 1200 IU/mL.

Drugs: No interference was found at therapeutic concentrations using common drug panels.[27,28]

Therapeutic drugs: Significantly decreased CRP values may be obtained from samples taken from patients who have been treated with carboxypenicillins.

High dose hook-effect: No false result occurs up to a CRP concentration of 1000 mg/L.

In very rare cases, gammopathy, in particular type IgM (Waldenström's macroglobulinemia), may cause unreliable results.[29]

Although measures were taken to minimize interference caused by human anti-mouse antibodies, erroneous findings may be obtained from samples taken from patients who have been treated with monoclonal mouse antibodies or have received them for diagnostic purposes.

For diagnostic purposes, the results should always be assessed in conjunction with the patient's medical history, clinical examination and other findings.

ACTION REQUIRED
Special Wash Programming: The use of special wash steps is mandatory when certain test combinations are run together on Roche/Hitachi **cobas c** systems. The latest version of the carry-over evasion list can be found with the NaOHD-SMS-SmpCln1+2-SCCS Method Sheets. For further instructions refer to the operator's manual. **cobas c** 502 analyzer: All special wash programming necessary for avoiding carry-over is available via the **cobas** link, manual input is required in certain cases.

Where required, special wash/carry-over evasion programming must be implemented prior to reporting results with this test.

Limits and ranges

Measuring range

0.15-20.0 mg/L (1.43-190 nmol/L, 0.015-2.0 mg/dL)

Determine samples having higher concentrations via the rerun function. Dilution of samples via the rerun function is a 1:15 dilution. Results from samples diluted using the rerun function are automatically multiplied by a factor of 15.

Lower limits of measurement

Lower detection limit of the test

0.15 mg/L (1.43 nmol/L, 0.015 mg/dL)

The lower detection limit represents the lowest measurable analyte level that can be distinguished from zero. It is calculated as the value lying 3 standard deviations above that of the lowest standard (standard 1 + 3 SD, repeatability, n = 21).

Functional sensitivity

0.3 mg/L (2.96 nmol/L, 0.03 mg/dL)

The functional sensitivity is the lowest CRP concentration that can be reproducibly measured with an inter-assay coefficient of variation of < 10 %.

Expected values

Consensus reference interval for adults:[30]

IFCC/CRM 470

mg/dL	mg/L	nmol/L
< 0.5	< 5.0	< 47.6

The CDC/AHA recommended the following hsCRP cut-off points (tertiles) for CVD risk assessment:[21,31]

hsCRP level (mg/L)	hsCRP level (nmol/L)	Relative risk
< 1.0	< 9.52	low
1.0-3.0	9.52-28.6	average
> 3.0	> 28.6	high

Patients with higher hsCRP concentrations are more likely to develop myocardial infarction and severe peripheral vascular disease.

5-95 % reference intervals of neonates and children:[32]

Neonates (0-3 weeks): 0.1-4.1 mg/L (0.95-39.0 nmol/L)

Children (2 months-15 years): 0.1-2.8 mg/L (0.95-26.7 nmol/L)

It is important to monitor the CRP concentration during the acute phase of the illness.

Roche has not evaluated reference ranges in a pediatric population.

Each laboratory should investigate the transferability of the expected values to its own patient population and if necessary determine its own reference ranges.

Increases in CRP values are non-specific and should not be interpreted without a complete clinical history.

When using hsCRP to assess the risk of coronary heart disease, measurements should be made on metabolically stable patients and compared to previous values. Optimally, the average of hsCRP results repeated two weeks apart should be used for risk assessment. Measurements should be compared to previous values. When the results are being used for risk assessment, patients with persistently unexplained hsCRP levels of above 10 mg/L (95.2 nmol/L) should be evaluated for non-cardiovascular origins. Testing for any risk assessment should not be performed while there is indication of infection, systemic inflammation or trauma.[21]

Specific performance data

Representative performance data on the analyzers are given below. Results obtained in individual laboratories may differ.

Precision

Precision was determined using human samples and controls in an internal protocol with repeatability (n = 21) and intermediate precision (3 aliquots per run, 1 run per day, 21 days). The following results were obtained:

Repeatability	*Mean*	*SD*	*CV*
	mg/L (nmol/L, mg/dL)	*mg/L (nmol/L, mg/dL)*	*%*
Precinorm Protein	9.00 (85.7, 0.900)	0.10 (1.0, 0.010)	1.2
CRP T Control N	4.34 (41.3, 0.434)	0.04 (0.4, 0.004)	1.0
Human serum 1	15.9 (151, 1.59)	0.1 (1, 0.01)	0.4
Human serum 2	0.54 (5.14, 0.054)	0.01 (0.10, 0.001)	1.6

Intermediate precision	*Mean*	*SD*	*CV*
	mg/L (nmol/L, mg/dL)	*mg/L (nmol/L, mg/dL)*	*%*
Precinorm Protein	9.06 (86.3, 0.906)	0.11 (1.1, 0.011)	1.3
CRP T Control N	4.28 (40.8, 0.428)	0.11 (1.1, 0.011)	2.6
Human serum 3	13.3 (126, 1.33)	0.3 (3, 0.03)	2.1
Human serum 4	0.53 (5.05, 0.053)	0.05 (0.48, 0.005)	8.4

Method comparison

CRP values for human serum and plasma samples obtained on a Roche/Hitachi **cobas c** 501 analyzer (y) were compared with those

For peer review only - http://bmjopen.bmj.com/site/about/guidelines.xhtml

0004628918190c501V12.0

CRPHS

cobas®

Cardiac C-Reactive Protein (Latex) High Sensitive

determined using the corresponding reagent on a Roche/Hitachi 917 analyzer (x).
Sample size (n) = 192

Passing/Bablok[33]	Linear regression
y = 0.992x + 0.254 mg/L	y = 0.946x + 0.514 mg/L
τ = 0.944	r = 0.996

The sample concentrations were between 0.500 and 19.7 mg/L (4.76 and 188 nmol/L, 0.050 and 1.97 mg/dL).

References

1 Henry JB, ed. Clinical Diagnosis and Management by Laboratory Methods. Vol II. Philadelphia, Pa: WB Saunders 1979.

2 Greiling H, Gressner AM, eds. Lehrbuch der Klinischen Chemie und Pathobiochemie, 3rd ed. Stuttgart/New York: Schattauer Verlag 1995:234-236.

3 Thomas L, Messenger M. Pathobiochemie und Labordiagnostik der Entzündung. Lab med 1993;17:179–194.

4 Young B, Gleeson M, Cripps AW. C-reactive protein: A critical review. Pathology 1991;23:118-124.

5 Tietz NW. Clinical Guide to Laboratory Tests. 3rd ed. Philadelphia, Pa: WB Saunders Co; 1995.

6 Wasunna A, Whitelaw A, Gallimore R, et al. C-reactive protein and bacterial infection in preterm infants. Eur J Pediatr 1990 Mar;149(6):424-427.

7 Liuzzo G, Biasucci LM, Gallimore JR, et al. The prognostic value of C-reactive protein and serum amyloid A protein in severe unstable angina. N Engl J Med 1994;331:417-424.

8 Kuller LH, Tracy RP, Shaten J, et al. Relation of c-reactive protein and coronary heart disease in the MRFIT nested case control study. Am J Epidem 1996;144:537-547.

9 Ridker PM, Glynn RJ, Hennekens CH, et al. C-Reactive Protein Adds to the Predictive Value of Total and HDL Cholesterol in Determining Risk of First Myocardial Infarction. Circulation 1998;97:2007-2011.

10 Ridker PM, Cushman M, Stampfer MJ, et al. Plasma Concentration of C-Reactive Protein and Risk of Developing Peripheral Vascular Disease. Circulation 1998;97:425-428.

11 Ridker PM, Cushman M, Stampfer MJ, et al. Inflammation, Aspirin, and the Risk of Cardiovascular Disease in Apparently Healthy Men. N Eng J Med 1997;336(14):973-979.

12 Danesh J, Wheeler JG, Hirschfield GM, et al. C-Reactive Protein and Other Circulating Markers of Inflammation in the Prediction of Coronary Heart Disease. N Eng J Med 2004;350(14):1387-1397.

13 Ridker PM, Hennekens CH, Buring JE, et al. C-Reactive Protein and Other Markers of Inflammation in the Prediction of Cardiovascular Disease in Women. N Engl J Med 2000;342(12):836-843.

14 Tracy RP, Lemaitre RN, Psaty BM, et al. Relationship of C-Reactive Protein to Risk of Cardiovascular Disease in the Elderly. Arterioscler Thromb Vasc Biol 1997;17:1121-1127.

15 Järvisalo MJ, Harmoinen A, Hakanen M, et al. Elevated Serum C-Reactive Protein Levels and Early Arterial Changes in Healthy Children. Arterioscler Thromb Vasc Biol, (August) 2002;1323-1328.

16 Almagor M, Keren A, Banai S. Increased C-Reactive Protein Level after Coronary Stent Implantation in Patients with Stable Coronary Artery Disease. American Heart Journal 2003;145 (2):248-253.

17 Katritsis D, Korovesis S, Giazitzoglou E, et al. C-Reactive Protein Concentrations and Angiographic Characteristics of Coronary Lesions. Clin Chem 2001;47(5):882-886.

18 Beattie MS, Shlipak, MG, Liu H, et al. C-Reactive Protein and Ischemia in Users and Nonusers of β-Blockers and Statins. Circulation 2003;107:245-250.

19 Plenge JK, Hernandez TL, Weil KM, et al. Simvastatin Lowers C-Reactive Protein Within 14 Days. An Effect Independent of Low-Density Lipoprotein Cholesterol Reduction. Circulation 2002;106:1447-1452.

20 Lindahl B, Toss H, Siegbahn A, et al. Markers of Myocardial Damage and Inflammation in Relation to Long-Term Mortality in Unstable Coronary Artery Disease. N Eng J Med 2000;343(16):1139-1147.

21 Pearson TA, Mensah GA, Alexander RW, et al. Markers of Inflammation and Cardiovascular Disease. Application to Clinical and Public Health Practice. A Statement for Healthcare Professionals From the Centers for Disease Control and Prevention and the American Heart Association. Circulation 2003;107:499-511.

22 Price CP, Trull AK, Berry D, et al. Development and validation of a particle-enhanced turbidimetric immunoassay for C-reactive protein. J Immunol Methods 1987;99:205-211.

23 Eda S, Kaufmann J, Roos W, et al. Development of a New Microparticle-Enhanced Turbidimetric Assay for C-reactive Protein with Superior Features in Analytical Sensitivity and Dynamic Range. J Clin Lab Anal 1998;12:137-144.

24 Use of Anticoagulants in Diagnostic Laboratory Investigations. WHO Publication WHO/DIL/LAB/99.1 Rev. 2: Jan 2002.

25 Baudner S, Bienvenu J, Blirup-Jensen S, et al. The certification of a matrix reference material for immunochemical measurement of 14 human serum proteins CRM470. Report EUR 15243 EN 1993;1-186.

26 Glick MR, Ryder KW, Jackson SA. Graphical Comparisons of Interferences in Clinical Chemistry Instrumentation. Clin Chem 1986;32:470-475.

27 Breuer J. Report on the Symposium "Drug effects in Clinical Chemistry Methods". Eur J Clin Chem Clin Biochem 1996;34:385-386.

28 Sonntag O, Scholer A. Drug interference in clinical chemistry: recommendation of drugs and their concentrations to be used in drug interference studies. Ann Clin Biochem 2001;38:376-385.

29 Bakker AJ, Mücke M. Gammopathy interference in clinical chemistry assays: mechanisms, detection and prevention. Clin Chem Lab Med 2007;45(9):1240-1243.

30 Dati F, Schumann G, Thomas L, et al. Consensus of a group of professional societies and diagnostic companies on guidelines for interim reference ranges for 14 proteins in serum based on the standardization against the IFCC/BCR/CAP reference material (CRM 470). Eur J Clin Chem Clin Biochem 1996;34:517-520.

31 Ridker PM. Clinical Application of C-Reactive Protein for Cardiovascular Disease Detection and Prevention. Circulation 2003;107:363-369.

32 Schlebusch H, Liappis N, Kalina E, et al. High Sensitive CRP and Creatinine: Reference Intervals from Infancy to Childhood. J Lab Med 2002;26:341-346.

33 Bablok W, Passing H, Bender R, et al. A general regression procedure for method transformation. Application of linear regression procedures for method comparison studies in clinical chemistry, Part III. J Clin Chem Clin Biochem 1988 Nov;26(11):783-790.

A point (period/stop) is always used in this Method Sheet as the decimal separator to mark the border between the integral and the fractional parts of a decimal numeral. Separators for thousands are not used.

Symbols

Roche Diagnostics uses the following symbols and signs in addition to those listed in the ISO 15223-1 standard (for USA: see https://usdiagnostics.roche.com for definition of symbols used):

CONTENT	Contents of kit
→	Volume after reconstitution or mixing
GTIN	Global Trade Item Number

For peer review only

For peer review only - http://bmjopen.bmj.com/site/about/guidelines.xhtml

0004628918190c501V12.0

CRPHS

cobas®

Cardiac C-Reactive Protein (Latex) High Sensitive

FOR US CUSTOMERS ONLY: LIMITED WARRANTY

Roche Diagnostics warrants that this product will meet the specifications stated in the labeling when used in accordance with such labeling and will be free from defects in material and workmanship until the expiration date printed on the label. THIS LIMITED WARRANTY IS IN LIEU OF ANY OTHER WARRANTY, EXPRESS OR IMPLIED, INCLUDING ANY IMPLIED WARRANTY OF MERCHANTABILITY OR FITNESS FOR PARTICULAR PURPOSE. IN NO EVENT SHALL ROCHE DIAGNOSTICS BE LIABLE FOR INCIDENTAL, INDIRECT, SPECIAL OR CONSEQUENTIAL DAMAGES.

COBAS, COBAS C, PRECICONTROL and PRECINORM are trademarks of Roche.

All other product names and trademarks are the property of their respective owners.

Additions, deletions or changes are indicated by a change bar in the margin.

© 2019, Roche Diagnostics

CE

Roche Diagnostics GmbH, Sandhofer Strasse 116, D-68305 Mannheim
www.roche.com

Distribution in USA by:
Roche Diagnostics, Indianapolis, IN
US Customer Technical Support 1-800-428-2336

Roche

For peer review only

For peer review only - http://bmjopen.bmj.com/site/about/guidelines.xhtml

07559992500V4.0

Elecsys Folate III

cobas®

REF	[i]	Σ	SYSTEM
07559992190	07559992500	100	**cobas e** 411<br>**cobas e** 601<br>**cobas e** 602

English

System information

For **cobas e** 411 analyzer: test number 1520
For **cobas e** 601 and **cobas e** 602 analyzers: Application Code Number 721

Intended use

Binding assay for the in vitro quantitative determination of folate in human serum and plasma.

The binding assay is intended for use on Elecsys and **cobas e** immunoassay analyzers.

Summary

Folate belongs to the family of B-group vitamins composed of an aromatic pteridine ring linked through a methylene group to p-aminobenzoic acid and a glutamate residue. Folate (folic acid) is vital for normal cellular functions and plays an essential role in nucleic acid synthesis, methionine regeneration, shuttling and redox reactions of one-carbon units required for normal metabolism and regulation.[1,2]

The folate metabolism can be exemplified as a cycle, where folate facilitates the transfer of one-carbon units from one molecule to another required in various biochemical reactions: for example, tetrahydrofolate (THF) accepts a single carbon unit from serine, which is reduced in a number of steps to 5-methyltetrahydrofolate (5-MTHF). 5-MTHF gives its methyl group to homocysteine, which is - with involvement of methionine synthase and vitamin B12 - enzymatically converted to methionine. The resulting THF starts again the cycle of methyl group synthesis. From methionine, the methyl groups are transferred to S-adenosylmethionine (SAM).[3] SAM serves as a methyl group donor in several methylation reactions, like DNA, RNA and protein methylation.[1]

The methionine cycle is highly sensitive to folate deficiency: with a low folate status, the ability of the cell to re-methylate homocysteine is impaired and this results in increased homocysteine concentrations in plasma.[2]

Folate also plays an essential role in the synthesis of purine and pyrimidine precursors of nucleic acids. Altered distribution of methyl groups and impaired DNA synthesis play an essential role in the development of cancers. Abnormal folate status has also been linked with the development of diseases like cardiovascular diseases, neural tube defects, cleft lip and palate, late pregnancy complications, neurodegenerative and psychiatric disorders.[1,2]

Folate belongs to the group of essential vitamins, i.e. it cannot be synthesized by the human organism and therefore must be absorbed from diet. Primary sources of folates are green and leafy vegetables, sprouts, fruits, brewer's yeast and liver.[1,2]

Folate deficiency can be caused by decreased nutritional intake, poor absorption of ingested folate in the intestine or increased demand of folate, for example during physical activity or pregnancy. Deficiency of folate can also be a result of liver diseases or impaired folate metabolism due to genetic defects or drug interactions.[2]

A clinical manifestation of both folate and vitamin B12 deficiency is the so called megaloblastic (macrocytic) anemia: due to the affected DNA synthesis and cell maturation, especially involving the cells of erythropoiesis, the total count of erythrocytes is significantly reduced. The hemoglobin synthesis capacity however is normal, which leads to abnormally large erythrocyte precursors ("macrocytes" or "megaloblasts"), which have an elevated hemoglobin content ("hyperchromic anemia").[3,4]

Because vitamin B12 and folate are closely interrelated in the cellular one-carbon unit metabolism, and also hematologic and clinical consequences of the two vitamin deficiency states might be similar, it is advisable to determine both parameters simultaneously in patients with the relevant symptoms of vitamin deficiency.[3,4]

Test principle

Competition principle. Total duration of assay: 27 minutes.

- 1st incubation: By incubating 25 µL of sample with the folate pretreatment reagents 1 and 2, bound folate is released from endogenous folate binding proteins.
- 2nd incubation: By incubating the pretreated sample with the ruthenium labeled folate binding protein, a folate complex is formed, the amount of which is dependent upon the analyte concentration in the sample.
- 3rd incubation: After addition of streptavidin-coated microparticles and folate labeled with biotin, the unbound sites of the ruthenium labeled folate binding protein become occupied, with formation of a ruthenium labeled folate binding protein-folate biotin complex. The entire complex becomes bound to the solid phase via interaction of biotin and streptavidin.
- The reaction mixture is aspirated into the measuring cell where the microparticles are magnetically captured onto the surface of the electrode. Unbound substances are then removed with ProCell/ProCell M. Application of a voltage to the electrode then induces chemiluminescent emission which is measured by a photomultiplier.
- Results are determined via a calibration curve which is instrument-specifically generated by 2-point calibration and a master curve provided via the reagent barcode or e-barcode.

Reagents - working solutions

The reagent rackpack (M, R1, R2) and the pretreatment reagents (PT1, PT2) are labeled as Fol III.

PT1 Pretreatment reagent 1 (white cap), 1 bottle, 4 mL:
Sodium 2-mercaptoethanesulfonate (MESNA) 40 g/L, pH 5.5.

PT2 Pretreatment reagent 2 (gray cap), 1 bottle, 5 mL:
Sodium hydroxide 25 g/L.

M Streptavidin-coated microparticles (transparent cap), 1 bottle, 6.5 mL:
Streptavidin-coated microparticles 0.72 mg/mL; preservative.

R1 Folate binding protein~Ru(bpy)$_3^{2+}$ (gray cap), 1 bottle, 9 mL:
Ruthenium labeled folate binding protein 75 µg/L; human serum albumin (stabilizer); borate/phosphate/citrate buffer 70 mmol/L, pH 5.5; preservative.

R2 Folate~biotin (black cap), 1 bottle, 8 mL:
Biotinylated folate 17 µg/L; biotin 120 µg/L; human serum albumin (stabilizer); borate buffer 100 mmol/L, pH 9.0; preservative.

Precautions and warnings

For in vitro diagnostic use.
Exercise the normal precautions required for handling all laboratory reagents.
Disposal of all waste material should be in accordance with local guidelines.
Safety data sheet available for professional user on request.

This kit contains components classified as follows in accordance with the Regulation (EC) No. 1272/2008:

2-methyl-2H-isothiazol-3-one hydrochloride

EUH 208 May produce an allergic reaction.

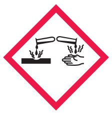

Danger

H290 May be corrosive to metals.

For peer review only

For peer review only - http://bmjopen.bmj.com/site/about/guidelines.xhtml

07559992500V4.0

Elecsys Folate III

cobas®

H314	Causes severe skin burns and eye damage.

Prevention:

P280	Wear protective gloves/ protective clothing/ eye protection/ face protection.

Response:

P301 + P330 + P331	IF SWALLOWED: Rinse mouth. Do NOT induce vomiting.
P303 + P361 + P353	IF ON SKIN (or hair): Take off immediately all contaminated clothing. Rinse skin with water.
P304 + P340 + P310	IF INHALED: Remove person to fresh air and keep comfortable for breathing. Immediately call a POISON CENTER/ doctor.
P305 + P351 + P338 + P310	IF IN EYES: Rinse cautiously with water for several minutes. Remove contact lenses, if present and easy to do. Continue rinsing. Immediately call a POISON CENTER/ doctor.
P390	Absorb spillage to prevent material damage.

Product safety labeling follows EU GHS guidance.

Contact phone: all countries: +49-621-7590

All human material should be considered potentially infectious. All products derived from human blood are prepared exclusively from the blood of donors tested individually and shown to be free from HBsAg and antibodies to HCV and HIV. The testing methods used assays approved by the FDA or cleared in compliance with the European Directive 98/79/EC, Annex II, List A.

However, as no testing method can rule out the potential risk of infection with absolute certainty, the material should be handled with the same level of care as a patient specimen. In the event of exposure, the directives of the responsible health authorities should be followed.[5,6]

Avoid foam formation in all reagents and sample types (specimens, calibrators and controls).

Reagent handling

The reagents in the kit have been assembled into a ready-for-use unit that cannot be separated.

All information required for correct operation is read in from the respective reagent barcodes.

Storage and stability

Store at 2-8 °C.

Do not freeze.

Store the Elecsys reagent kit **upright** in order to ensure complete availability of the microparticles during automatic mixing prior to use.

Stability:	
unopened at 2-8 °C	up to the stated expiration date
after opening at 2-8 °C	56 days (8 weeks)
on the analyzers	14 days (2 weeks) onboard<br>or<br>28 days (4 weeks) when stored alternatively in the refrigerator and on the analyzer, with the total time onboard on the analyzer not exceeding 10 x 8 hours

Specimen collection and preparation

Only the specimens listed below were tested and found acceptable.

Serum collected using standard sampling tubes or tubes containing separating gel.

Li-heparin plasma. Li-heparin plasma tubes containing separating gel can be used.

Criterion: Method comparison serum versus Li-heparin plasma, slope 0.9-1.1 + intercept within < ± 2x Limit of Blank (LoB), coefficient of correlation ≥ 0.95.

Serum: Stable for 2 hours at 15-25 °C, 48 hours at 2-8 °C, 28 days at -20 °C (± 5 °C). Freeze only once. Protect from light. Store the samples at 2-8 °C if they cannot be measured immediately.

Li-heparin plasma: Stable for 2 hours at 15-25 °C, 48 hours at 2-8 °C, 28 days at -20 °C (± 5 °C). Freeze only once. Protect from light. Store the samples at 2-8 °C if they cannot be measured immediately.

The sample types listed were tested with a selection of sample collection tubes that were commercially available at the time of testing, i.e. not all available tubes of all manufacturers were tested. Sample collection systems from various manufacturers may contain differing materials which could affect the test results in some cases. When processing samples in primary tubes (sample collection systems), follow the instructions of the tube manufacturer.

Samples should not subsequently be altered with additives (biocides, anti-oxidants or substances possibly changing the pH of the sample) in order to avoid erroneous folate recovery.

Centrifuge samples containing precipitates before performing the assay.

Do not use heat-inactivated samples.

Ensure the samples, calibrators and controls are at 20-25 °C prior to measurement.

Due to possible evaporation effects, samples, calibrators and controls on the analyzers should be analyzed/measured within 2 hours.

Note: Hemolysis may significantly increase folate values due to high concentrations of folate in red blood cells. Therefore, hemolyzed samples are not suitable for use in this assay. Samples for folate determinations should be collected from fasting persons.

Materials provided

See "Reagents – working solutions" section for reagents.

Materials required (but not provided)

- REF 07560001190, Folate III CalSet, for 4 x 1.0 mL
- REF 05618860190, PreciControl Varia, for 4 x 3.0 mL
- REF 11732277122, Diluent Universal, 2 x 16 mL sample diluent or REF 03183971122, Diluent Universal, 2 x 36 mL sample diluent
- General laboratory equipment
- **cobas e** analyzer

Additional materials for the **cobas e** 411 analyzer:

- REF 11662988122, ProCell, 6 x 380 mL system buffer
- REF 11662970122, CleanCell, 6 x 380 mL measuring cell cleaning solution
- REF 11930346122, Elecsys SysWash, 1 x 500 mL washwater additive
- REF 11933159001, Adapter for SysClean
- REF 11706802001, AssayCup, 60 x 60 reaction cups
- REF 11706799001, AssayTip, 30 x 120 pipette tips
- REF 11800507001, Clean-Liner

Additional materials for **cobas e** 601 and **cobas e** 602 analyzers:

- REF 04880340190, ProCell M, 2 x 2 L system buffer
- REF 04880293190, CleanCell M, 2 x 2 L measuring cell cleaning solution
- REF 03023141001, PC/CC-Cups, 12 cups to prewarm ProCell M and CleanCell M before use
- REF 03005712190, ProbeWash M, 12 x 70 mL cleaning solution for run finalization and rinsing during reagent change
- REF 03004899190, PreClean M, 5 x 600 mL detection cleaning solution
- REF 12102137001, AssayTip/AssayCup, 48 magazines x 84 reaction cups or pipette tips, waste bags
- REF 03023150001, WasteLiner, waste bags
- REF 03027651001, SysClean Adapter M

Additional materials for all analyzers:

For peer review only - http://bmjopen.bmj.com/site/about/guidelines.xhtml

07559992500V4.0

Elecsys Folate III

cobas®

- REF 11298500316, ISE Cleaning Solution/Elecsys SysClean, 5 x 100 mL system cleaning solution

Assay

For optimum performance of the assay follow the directions given in this document for the analyzer concerned. Refer to the appropriate operator's manual for analyzer-specific assay instructions.

Resuspension of the microparticles takes place automatically prior to use. Read in the test-specific parameters via the reagent barcode. If in exceptional cases the barcode cannot be read, enter the 15-digit sequence of numbers.

cobas e 601 and **cobas e** 602 analyzers: PreClean M solution is necessary.

Bring the cooled reagents to approximately 20 °C and place on the reagent disk (20 °C) of the analyzer. Avoid foam formation. The system automatically regulates the temperature of the reagents and the opening/closing of the bottles.

Calibration

Traceability: This method has been standardized against the WHO International Standard NIBSC code: 03/178.

Every Elecsys reagent set has a barcoded label containing specific information for calibration of the particular reagent lot. The predefined master curve is adapted to the analyzer using the relevant CalSet.

Calibration frequency: Calibration must be performed once per reagent lot using fresh reagent (i.e. not more than 24 hours since the reagent kit was registered on the analyzer).

Calibration interval may be extended based on acceptable verification of calibration by the laboratory.

Renewed calibration is recommended as follows:

- after 1 month (28 days) when using the same reagent lot
- after 7 days (when using the same reagent kit on the analyzer)
- as required: e.g. quality control findings outside the defined limits

Quality control

For quality control, use PreciControl Varia.

In addition, other suitable control material can be used.

Controls for the various concentration ranges should be run individually at least once every 24 hours when the test is in use, once per reagent kit, and following each calibration.

The control intervals and limits should be adapted to each laboratory's individual requirements. Values obtained should fall within the defined limits. Each laboratory should establish corrective measures to be taken if values fall outside the defined limits.

If necessary, repeat the measurement of the samples concerned.

Follow the applicable government regulations and local guidelines for quality control.

Calculation

The analyzer automatically calculates the analyte concentration of each sample (either in nmol/L or ng/mL).

Conversion factors: nmol/L x 0.44 = ng/mL

ng/mL x 2.27 = nmol/L

Limitations - interference

The assay is unaffected by icterus (bilirubin ≤ 496 µmol/L or ≤ 29 mg/dL), lipemia (Intralipid ≤ 1500 mg/dL), biotin (≤ 86.1 nmol/L or ≤ 21 ng/mL), IgG ≤ 16 g/L, IgA ≤ 4.0 g/L and IgM ≤ 10 g/L.

Criterion: Recovery within ± 10 % of initial value with samples > 4 ng/mL and ≤ ± 0.4 ng/mL with samples ≤ 4 ng/mL.

Hemolysis may significantly increase folate values due to high concentrations of folate in red blood cells. Therefore, hemolyzed samples are not suitable for use in this assay.

Samples should not be taken from patients receiving therapy with high biotin doses (i.e. > 5 mg/day) until at least 8 hours following the last biotin administration.

No interference was observed from rheumatoid factors up to a concentration of 1000 IU/mL.

In vitro tests were performed on 16 commonly used pharmaceuticals and in addition on human erythropoietin. No interference with the assay was found.

It is contraindicated to measure samples of patients receiving therapy with certain pharmaceuticals, e.g. methotrexate or leucovorin, because of the cross-reactivity of folate binding protein with these compounds.

Samples with extremely high total protein concentrations (hyperproteinemia) are not suitable for use in this assay. Hyperproteinemia may be caused by, but not limited to, the following conditions: Lymphoma[7,8], bone marrow disorders such as multiple myeloma, monoclonal gammopathy of undetermined significance (MGUS), Waldenström macroglobulinemia, plasmocytoma[7,8,9,10,11,12,13], Amyloidosis[13,14]. Respective samples may lead to the formation of protein gel in the assay cup, which may cause a run abort. The critical total protein concentration is dependent upon the individual sample composition.

In rare cases, interference due to extremely high titers of antibodies to streptavidin and ruthenium can occur. These effects are minimized by suitable test design.

For diagnostic purposes, the results should always be assessed in conjunction with RBC folate, the patient's medical history, clinical examination, and other findings.

Limits and ranges

Measuring range

0.6-20.0 ng/mL or 1.36-45.4 nmol/L (defined by the Limit of Blank and the maximum of the master curve). Values below the Limit of Blank are reported as < 0.6 ng/mL (< 1.36 nmol/L). Values above the measuring range are reported as > 20.0 ng/mL (> 45.4 nmol/L).

Lower limits of measurement

Limit of Blank, Limit of Detection and Limit of Quantitation

Limit of Blank = 0.6 ng/mL (1.36 nmol/L)

Limit of Detection = 1.2 ng/mL (2.72 nmol/L)

Limit of Quantitation = 2.0 ng/mL (4.54 nmol/L)

The Limit of Blank, Limit of Detection and Limit of Quantitation were determined in accordance with the CLSI (Clinical and Laboratory Standards Institute) EP17-A requirements.

The Limit of Blank is the 95th percentile value from n ≥ 60 measurements of analyte-free samples over several independent series. The Limit of Blank corresponds to the concentration below which analyte-free samples are found with a probability of 95 %.

The Limit of Detection is determined based on the Limit of Blank and the standard deviation of low concentration samples. The Limit of Detection corresponds to the lowest analyte concentration which can be detected (value above the Limit of Blank with a probability of 95 %).

The Limit of Quantitation is defined as the lowest amount of analyte in a sample that can be accurately quantitated with a total allowable relative error of ≤ 20 %.

It has been determined using low concentration folate samples.

Dilution

Samples with folate concentrations above the measuring range can be diluted manually with Diluent Universal. The recommended dilution is 1:2. The concentration of the diluted sample must be > 8.5 ng/mL or > 19.3 nmol/L. After manual dilution, multiply the results by the dilution factor 2.

Expected values

Referring to "The American Journal of Clinical Nutrition"[15] serum folate (folic acid) values were found as follows:

Sex	Age	N	Median		2.5th-97.5th percentile	
	years		ng/mL	nmol/L	ng/mL	nmol/L
Both	all	23345	13.0	29.5	4.6-34.8	10.4-78.9
Male	all	11387	12.3	27.9	4.5-32.2	10.2-73.0
Female	all	11958	13.6	30.1	4.8-37.3	10.9-84.5
Both	4-11	3595	17.2	39.0	8.6-37.7	19.5-85.4
Both	12-19	6390	12.1	27.4	5.0-27.2	11.3-61.6
Both	20-59	8689	11.6	26.3	4.4-31.0	10.0-70.2

For peer review only - http://bmjopen.bmj.com/site/about/guidelines.xhtml

07559992500V4.0

Elecsys Folate III

cobas®

Sex	Age	N	Median		2.5th-97.5th percentile	
	years		ng/mL	nmol/L	ng/mL	nmol/L
Both	≥ 60	4671	16.6	37.6	5.6-45.8	12.7-103.8

These values were obtained in the USA during the National Health and Nutrition Examination Survey (NHANES), 1999-2004.

The values shown below were performed on samples from an apparently healthy population, using the Elecsys Folate III assay.

The calculation is based on 404 sera (177 men, 227 women). The age range was between 20 and 65 years. Pregnant or lactating women were excluded. The reference population was selected according to normal homocysteine values.

N	Median		2.5th-97.5th percentile	
	ng/mL	nmol/L	ng/mL	nmol/L
404	8.94	20.3	3.89-26.8	8.83-60.8

Please note: These values should only be used as a guideline.

It should be taken into consideration that differences in the expected values may exist with respect to population and dietary status.

Each laboratory should investigate the transferability of the expected values to its own patient population and if necessary determine its own reference ranges.

Folate deficient sample values

25 samples considered to be deficient[a)] in serum folate concentration were assessed using the Elecsys Folate III assay. All samples were found to be below the 2.5th percentile as given in the table above.

a) Folate deficiency was assessed by measurement of serum folate by two commercially available folate assays.

Specific performance data

Representative performance data on the analyzers are given below. Results obtained in individual laboratories may differ.

Precision

Precision was determined using Elecsys reagents, pooled human sera and controls in a protocol (EP5-A2) of the CLSI (Clinical and Laboratory Standards Institute): 2 runs per day in duplicate each for 21 days (n = 84). The following results were obtained:

**cobas e** 411 analyzer					
		**Repeatability**		**Intermediate precision**	
Sample	Mean	SD	CV	SD	CV
	ng/mL	ng/mL	%	ng/mL	%
Human serum 1	1.88	0.150	8.0	0.205	10.9
Human serum 2	3.92	0.200	5.1	0.318	8.1
Human serum 3	11.9	0.346	2.9	0.571	4.8
Human serum 4	13.4	0.301	2.2	0.574	4.3
Human serum 5	17.8	0.440	2.5	0.666	3.7
PreciControl Varia1	3.24	0.215	6.6	0.309	9.5
PreciControl Varia2	11.6	0.314	2.7	0.566	4.9

**cobas e** 411 analyzer					
		**Repeatability**		**Intermediate precision**	
Sample	Mean	SD	CV	SD	CV
	nmol/L	nmol/L	%	nmol/L	%
Human serum 1	4.27	0.341	8.0	0.465	10.9
Human serum 2	8.90	0.454	5.1	0.722	8.1
Human serum 3	27.0	0.785	2.9	1.30	4.8
Human serum 4	30.4	0.683	2.2	1.30	4.3
Human serum 5	40.4	0.999	2.5	1.51	3.7
PreciControl Varia1	7.35	0.488	6.6	0.701	9.5
PreciControl Varia2	26.3	0.713	2.7	1.28	4.9

**cobas e** 601 and **cobas e** 602 analyzers					
		**Repeatability**		**Intermediate precision**	
Sample	Mean	SD	CV	SD	CV
	ng/mL	ng/mL	%	ng/mL	%
Human serum 1	1.66	0.255	15.4	0.268	16.1
Human serum 2	4.10	0.219	5.4	0.303	7.4
Human serum 3	11.1	0.449	4.1	0.503	4.6
Human serum 4	12.2	0.454	3.7	0.467	3.8
Human serum 5	16.4	0.502	3.1	0.625	3.8
PreciControl Varia1	2.34	0.189	8.1	0.228	9.8
PreciControl Varia2	10.1	0.443	4.4	0.489	4.9

**cobas e** 601 and **cobas e** 602 analyzers					
		**Repeatability**		**Intermediate precision**	
Sample	Mean	SD	CV	SD	CV
	nmol/L	nmol/L	%	nmol/L	%
Human serum 1	3.77	0.579	15.4	0.608	16.1
Human serum 2	9.31	0.497	5.4	0.688	7.4
Human serum 3	25.2	1.02	4.1	1.14	4.6
Human serum 4	27.7	1.03	3.7	1.06	3.8
Human serum 5	37.2	1.14	3.1	1.42	3.8
PreciControl Varia1	5.31	0.429	8.1	0.518	9.8
PreciControl Varia2	22.9	1.01	4.4	1.11	4.9

Method comparison

a) A comparison of the Elecsys Folate III assay (traceable to WHO IS 03/178; y) and the Elecsys Folate III assay prior to standardization against WHO IS 03/178 (x) using clinical samples gave the following correlations (ng/mL):

Number of samples measured: 113

Passing/Bablok[16]	Linear regression
y = 1.14x - 1.97	y = 1.11x - 1.77
τ = 0.939	r = 0.994

The sample concentrations were between 2.1 and 18 ng/mL (4.8 and 41 nmol/L).

b) A comparison of the Elecsys Folate III assay (y) with a commercially available method (x) using clinical samples gave the following correlations (ng/mL):

Number of samples measured: 106

Passing/Bablok[16]	Linear regression
y = 0.980x - 0.095	y = 1.09x - 0.659
τ = 0.924	r = 0.984

The sample concentrations were between 1.9 and 17 ng/mL (4.3 and 39 nmol/L).

For peer review only - http://bmjopen.bmj.com/site/about/guidelines.xhtml

07559992500V4.0

Elecsys Folate III

cobas®

c) A comparison of the Elecsys Folate III assay on the **cobas e** 601 analyzer (y) with the Elecsys Folate III assay on the **cobas e** 411 analyzer (x) using clinical samples gave the following correlations (ng/mL):

Number of samples measured: 105

Passing/Bablok[16]	Linear regression
y = 1.05x - 0.303	y = 0.981x + 0.143
τ = 0.868	r = 0.982

The sample concentrations were between 1.6 and 19 ng/mL (3.6 and 43 nmol/L).

Analytical specificity

The following cross-reactivities were found, tested with folate concentrations of approximately 3.5 ng/mL, 10 ng/mL and 19 ng/mL.

Cross-reactant	Concentration tested ng/mL	Cross-reactivity %
Amethopterin	750	2.5
Aminopterin	750	4.4
Folinic acid	750	0.7

References

1 Nazki FH, Sameer AS, Ganaie BA. Folate: Metabolism, genes, polymorphisms and the associated diseases. Gene 2014;533(1):11-20.

2 Scaglione F, Panzavolta G. Folate, folic acid and 5-methyltetrahydrofolate are not the same thing. Xenobiotica 2014;44(5):480-488.

3 Reynolds EH. The neurology of folic acid deficiency. Handb Clin Neurol 2014;120:927-43.

4 Wick M, Pinggera W, Lehmann P. Clinical Aspects and Laboratory. Iron metabolism, Anemias. Springer Verlag, Wien, New York, 6th edition 2011:41-42.

5 Occupational Safety and Health Standards: Bloodborne pathogens. (29 CFR Part 1910.1030). Fed. Register.

6 Directive 2000/54/EC of the European Parliament and Council of 18 September 2000 on the protection of workers from risks related to exposure to biological agents at work.

7 Wu AHB. Tietz clinical guide to laboratory tests, 4th ed. St. Louis, Saunders/Elsevier 2006:608-609, 916-917.

8 Paricaud K, Moulis G, Combis MS, et al. Causes of protidemia above 100 g/L. Eur J Intern Med 2014;25:e123.

9 Filippatos TD, Liamis G, Christopoulou F, et al. Ten common pitfalls in the evaluation of patients with hyponatremia. Eur J Intern Med 2016;29:22-25.

10 Mailankody S, Landgren O. Monoclonal gammopathy of undetermined significance and Waldenström's macroglobulinemia. Best Pract Res Clin Haematol 2016;29:187-193.

11 Morel P, Duhamel A, Gobbi P, et al. International prognostic scoring system for Waldenström macroglobulinemia. Blood 2009;113:4163-4170.

12 Rajkumar SV. Multiple Myeloma. Curr Probl Cancer 2009;33:7-64.

13 Gertz MA. Immunoglobulin light chain amyloidosis: 2016 update on diagnosis, prognosis, and treatment. Am J Hematol 2016;91:947-956.

14 Wu AHB. Tietz clinical guide to laboratory tests, 4th ed. St. Louis, Saunders/Elsevier 2006: 916-917, 925.

15 Pfeiffer CM, Johnson CL, Jain RB, et al. Trends in blood folate and vitamin B-12 concentrations in the United States, 1988-2004. Am J Clin Nutr 2007;86:718-727.

16 Bablok W, Passing H, Bender R, et al. A general regression procedure for method transformation. Application of linear regression procedures for method comparison studies in clinical chemistry, Part III. J Clin Chem Clin Biochem 1988 Nov;26(11):783-790.

For further information, please refer to the appropriate operator's manual for the analyzer concerned, the respective application sheets, the product information and the Method Sheets of all necessary components (if available in your country).

A point (period/stop) is always used in this Method Sheet as the decimal separator to mark the border between the integral and the fractional parts of a decimal numeral. Separators for thousands are not used.

Symbols

Roche Diagnostics uses the following symbols and signs in addition to those listed in the ISO 15223-1 standard (for USA: see dialog.roche.com for definition of symbols used):

CONTENT	Contents of kit
SYSTEM	Analyzers/Instruments on which reagents can be used
REAGENT	Reagent
CALIBRATOR	Calibrator
→	Volume after reconstitution or mixing
GTIN	Global Trade Item Number

COBAS, COBAS E, ELECSYS and PRECICONTROL are trademarks of Roche. INTRALIPID is a trademark of Fresenius Kabi AB.

All other product names and trademarks are the property of their respective owners.

Additions, deletions or changes are indicated by a change bar in the margin.

© 2019, Roche Diagnostics

CE

Roche Diagnostics GmbH, Sandhofer Strasse 116, D-68305 Mannheim
www.roche.com

Roche

For peer review only - http://bmjopen.bmj.com/site/about/guidelines.xhtml

Data collection tool
Household Listing Form

FNS/1
FNS EA no_______

Serial no of structure	Address description of structure	Residence (Y/N)	Serial no House/HH in the structure	Name of HH head	Occupied (Y/N)	Serial no occupied EA	Remark(mark "X" for selected HH)

For peer review only

Annex 2: Nutrition direct and indirect interventions/indicators assessment questionnaire

Nutrition direct and indirect Interventions Questionnaire

Module 1: Household identifier, characteristics and socio-demographic status

**Household identifier and characteristics**			
101	Region Code	|__||__|	
102	Woreda Code	|__||__|	
103	Kebele Code	|__||__|	
104	Gote Code	|__||__|	
105	Household Code	|__||__| |__||__||__||__||__||__|	GPS Coordinates
106	Unique Household Code	|__||__||__| Region Code; |__||__| Woreda Code; |__||__| Kebele Code; |__||__| EA Code	|__||__||__| Household Code
107	Residence	1 = Urban<br>2 = Rural	|__|
108	Do you own this house?	1 = Yes<br>0 = No	|__|
109	What is the main material of the walls?<br><br>**Observe**	1 = No walls<br>2 = Natural materials (cane, wood, mud, straw)<br>3 = Stone with mud<br>4 = Stone/bricks with cement<br>99 = Other	|__||__|
110	What is the main floor material?<br><br>**Observe**	1 = Natural floor (earth/sand/dung)<br>2 = Rudimentary floor (wood/palm/bamboo)<br>3=Finished floor (polished wood/ vinyl/ tiles/cement/carpet)<br>99 = Other	|__||__|
111	What is the main material of the roof?<br>**Observe**	1 = Thatch/grass or leaves<br>2 = Iron sheets or tiles<br>99 = Other	|__||__|
112	What type of fuel does your household mostly use for cooking?<br><br>**Do not read list**	1 = Dung<br>2 = Firewood/straw<br>3 = Charcoal<br>4 = Kerosene<br>5 = Gas (methane/biogas)<br>6 = Electricity<br>99 = Other	|__||__|
113	Is the house connected to electricity?	1 = Yes<br>0 = No	|__|
114	In total, how many of the following items are owned by residents of this household?<br><br>**Add the household total for each item**	A kerosene lamp/pressure lamp	|__|
115		Mobile phone	|__|
116		Cart	|__|
117		Bicycle	|__|
118		Motorcycle	|__|
119		Radio	|__|
120		Television	|__|

For peer review only

For peer review only - http://bmjopen.bmj.com/site/about/guidelines.xhtml

121		Car/tractor/Bajaj	|__|
122	Does this household own any livestock, herds, other farm animals, or poultry?	1 = Yes<br>0 = No **(Go to→131)**	
123	In total, how many of the following animals are owned by residents of this household?<br>**Add the household total for each item**	Milk cows, oxen or bulls?	|__|
124		Other cattle?	|__|
125		Horses, donkeys, or mules?	|__|
126		Camels	|__|
127		Goats?	|__|
128		Sheep?	|__|
129		Chickens or other poultry?	|__|
130		Beehives?	|__|
131	Does any member of this household own any agricultural land?	1 = Yes<br>0 = No	|__|
132	How often does anyone smoke inside your house?<br><br>*Would you say daily, weekly, monthly, less often than once a month, or never?*	1 = Daily<br>2 = Weekly<br>3 = Monthly<br>4 = Less once monthly<br>5 = Never	|__|
**Household head socio-demographic status**			
133	Age in years		|__||__|
134	Marital status	1. Single<br>2. Married<br>3. Divorced<br>4. Separated<br>5. Widowed	|__|
135	What is the highest level of school the head of the household completed?	1. None<br>2. Primary<br>3. Secondary<br>4. Technical/vocational certificate<br>5. Higher / university/ college<br>98.Don't know<br>99.Other (specify)_______	|__||__|
136	What is the religion of the head of the HH?	1. Orthodox<br>2. Protestant<br>3. Catholic/ other Christian<br>4. Muslim<br>5. No religion<br>98.Don't know<br>99.Other religion (specify)___________	|__||__|
137	Ethnicity	Specify	

For peer review only - http://bmjopen.bmj.com/site/about/guidelines.xhtml

Module 2: Child health

Now I would like to ask some questions about the health of your children born in the last 5 years. We will talk about each separately, starting with the youngest.			
201	Child's code		|__||__|
202	Mother's name	Mother's given name	
203	Mother's age		
204	Mother's education level	1=None<br>2=Primary<br>3=Secondary<br>4=Technical/vocational certificate<br>5=Higher / university/ college<br>98=Don't know<br>99. Other (specify)_________	
205	Mother's marital status	1=Single<br>2 =Married<br>3=Divorced<br>4 =Separated<br>5=Widowed	
206	Mother's religion	1=Orthodox<br>2=Protestant<br>3=Catholic/ other Christian<br>4=Muslim<br>5=No religion<br>98=Don't know<br>99=Other religion (specify)______	
207	Mother's ethnicity	Specify	
208	Child's name	Child's given name	
209	Child (NAME) sex	1 = Boy<br>2 = Girl	|__|
210	Child (NAME) age?	Age in months or age at the time of the child's death	|__||__|
211	In the last six months, was (NAME) given any vitamin A supplement?	1 = Yes<br>0 = No<br>98 = Don't know	|__||__|
212	When was the child (NAME) given the vitamin A supplement?	Specify	
213	In the last 12 months, was (NAME) given any iron tablet or syrup or supplement?	1 = Yes<br>0 = No<br>98 = Don't know	|__||__|
214	In the last 6 months, was (name) given any medicine for intestinal worms?	1 = Yes<br>0 = No<br>98 = Don't know	|__||__|
215	In the last 3 months, has any healthcare provider measured?	1 = Yes, 0 = No, 98 = Don't know	
		Weight	|__||__|
		Height/length	|__||__|
		MUAC	|__||__|
216	Has (name) had diarrhea in the last 2 weeks?	1 = Yes<br>0 = No **(Go to 224)**	|__||__|

For peer review only - http://bmjopen.bmj.com/site/about/guidelines.xhtml

For peer review only

		98 = Don't know	
217	Now I would like to know how much was the child given to drink during diarrhea, including breast milk. Was the child given less than usual to drink, about the same amount, or more than usual to drink?	1 = Much less<br>2 = Somewhat less<br>3 = About the same<br>4 = More<br>5 = Nothing to drink<br>98 = Don't know	|__|
218	When the child had diarrhea, was he/she given less than usual to eat, about the same amount, more than usual, or nothing to eat?	1 = Much less<br>2 = Somewhat less<br>3 = About the same<br>4 = More<br>5 = Nothing to drink<br>98 = Don't know	|__|
219	Did you seek advice or treatment for the diarrhea from any source?	1 = Yes<br>0 = No **(Go to 221)**	|__|
220	Where did you seek advice or treatment? Anywhere else?<br><br>*Probe to identify the type of source.*<br>*If unable to determine if public, private, or NGO sector, record '21' and write the name Of the place(s).*	1= Government hospital<br>2= Government health center<br>3= Government health post<br>4 = Mobile clinic<br>5 = Community health worker/ fieldworker<br>6 = Other public sector (specify)<br>7 = Private hospital<br>8 = Private clinic<br>9 = Pharmacy<br>10 = Private doctor<br>11 = Mobile clinic<br>12 = Community health worker/fieldworker<br>13 = Other private medical sector (specify)<br>14 = NGO hospital<br>15 = NGO clinic<br>16 = Other NGO medical sector (specify)<br>17 = Shop<br>18 = Traditional practitioner<br>19 = Market<br>20 = Itinerant drug seller<br>99 = Other (specify)________	|__||__|
	Was (name) given any of the following at any time since (name) started having diarrhea:	1 = Yes, 0 = No, 98 = Don't know	
221		Fluid from ORS packet	|__||__|
222		Zinc	|__||__|
223		Homemade fluid	|__||__|
224	Has (name) been ill with a fever at any time in the last 2 weeks?	1 = Yes<br>0 = No (**Go to 226**)<br>98 = Don't know	|__||__|
225	Where did you seek advice or treatment for	1= Government hospital	

For peer review only - http://bmjopen.bmj.com/site/about/guidelines.xhtml

	fever?<br>Anywhere else?<br><br>*Probe to identify the type of source.*<br>*If unable to determine if public, private, or NGO sector, record '21' and write the name Of the place(s).*	2= Government health center<br>3= Government health post<br>4 = Mobile clinic<br>5 = Community health worker/ fieldworker<br>6 = Other public sector (specify)<br>7 = Private hospital<br>8 = Private clinic<br>9 = Pharmacy<br>10 = Private doctor<br>11 = Mobile clinic<br>12 = Community health worker/fieldworker<br>13 = Other private medical sector (specify)<br>14 = NGO hospital<br>15 = NGO clinic<br>16 = Other NGO medical sector (specify)<br>17 = Shop<br>18 = Traditional practitioner<br>19 = Market<br>20 = Itinerant drug seller<br>99 = Other (specify)______	|__||__|
226	Was Child (Name) ever breastfed?	1 = Yes<br>0 = No (**Go 228**)	|__|
227	How many months the child (NAME) was breastfed?		|__|
**Anthropometric and clinical nutrition assessment**			
228	Weight		|__||__|
229	Height/length		|__||__|
230	MUAC		|__||__|
231	Presence of bilateral oedema for children 6-59 months	1 = Yes<br>0 = No	|__|
232	Bitot spot	1 = Yes<br>0 = No	

For peer review only

Module 3: Infant and young child feeding practices

**For children 0-23 months**			
This module is to be administered to the mother/caregiver of children born 0-23 months before the survey living with respondents. Verify that the respondent you are speaking to is the mother/caregiver of the child.			
301	Was Child (Name) ever breastfed?	1 = Yes<br>0 = No (**Go to 304)**<br>98 = Don't know	|__||__|
302	Was Child (NAME) given the first milk (colostrum) after birth?	1 = Yes<br>0 = No<br>98 = Don't know	|__||__|
303	How long after birth did you first put (NAME) to the breast, even if your breast milk did not arrive?	1 = Immediately after birth, or within 1 hour<br>2 = Between 1 and 24 hours<br>3 = More than 24 hours after delivery<br>98 = Don't know	|__||__|
304	Child (NAME) alive now?	1 = Yes<br>0 = No (**Go to 401**)	|__||__|
305	Was *(NAME)* breastfed yesterday from sunrise until today sunrise?<br>NB: Breastfeeding could be by the mother herself or by wet mother.	1 = Yes<br>0 = No (**Go to 307**)<br>98 = Don't know	|__||__|
306	*Sometimes babies are fed breast milk in different ways, for example by spoon, cup or bottle. This can happen when the mother cannot always be with her baby. Sometimes babies are breastfed by another woman, or given breast milk from another woman by spoon, cup or bottle or some other way. This can happen if a mother cannot breastfeed her own baby.*<br><br>Did *(NAME)* consume breast milk in any of these ways yesterday from sunrise until today sunrise?	1 = Yes<br>0 = No<br>98 = Don't know	|__||__|
307	Now I would like to ask you about some medicines and vitamins that are sometimes given to infants.<br><br>Was *(NAME)* given any vitamin drops or other medicines as drops yesterday from sunrise until today sunrise?	1 = Yes<br>0 = No<br>98 = Don't know	|__||__|
308	Was *(NAME)* given Lemlem or ORS in the last two weeks?	1 = Yes<br>0 = No<br>98 = Don't know	|__||__|

For peer review only - http://bmjopen.bmj.com/site/about/guidelines.xhtml

309	In the last 6 months, did any healthcare provider or community health worker talk with you about how and what to feed your child?		1 = Yes<br>0 = No<br>98 = Don't know	|__||__|
310	Now, I would like to ask you about some liquids that *(NAME)* may have had yesterday from sunrise until today sunrise?<br><br>**If yes to Q310, read the list of liquids starting with 'plain water'.**		Did *(NAME)* have any *(item from list)*?<br>1 = Yes<br>0 = No **(Go to 321)**<br>98 = Don't know	|__||__|
311		Plain water	1 = Yes<br>0 = No<br>98 = Don't know	|__||__|
312		Infant formula such as S-26?	1 = Yes<br>0 = No **(Go to 314)**<br>98 = Don't know	|__||__|
313		How many times infant formula such as S-26?		|__||__|
314		Milk such as tinned, powdered, or fresh animal milk?	1 = Yes<br>0 = No **(Go to 316)**<br>98 = Don't know	|__||__|
315		How many times milk drink?		|__||__|
316		Yogurt drink?	1 = Yes<br>0 = No<br>98 = Don't know	|__||__|
317		Chocolate flavored drink?	1 = Yes<br>0 = No<br>98 = Don't know	|__||__|
318		Sodas, malt drinks or energy drinks?	1 = Yes<br>0 = No<br>98 = Don't know	|__||__|
319		Clear broth or clear soup?	1 = Yes<br>0 = No<br>98 = Don't know	|__||__|
320		Any other liquids?	1 = Yes<br>0 = No<br>98 = Don't know	|__||__|

Now I would like to ask you about foods that (NAME) had yesterday during the day or night. I am interested in foods your child ate whether at home or somewhere else. I will ask you about different types of foods, and I would like to know whether your child ate the food even if it was combined with other foods. Please do not answer 'yes' for any food or ingredient used in a small amount to add flavor to a dish.

OTHER FOODS: Please write down other foods in this box that respondent mentioned but are not in the list below

Yesterday during the day or at night, did (NAME) eat:

321	Did the child ate any solid or semi-solid food yesterday?	Eaten?<br>1 = Yes

For peer review only - http://bmjopen.bmj.com/site/about/guidelines.xhtml

			0 = No **(Go to 342)**<br>98 = Don't know
322	Yogurt, other than yogurt drink?		|__|
323	How many times did child (NAME) eat yogurt?		|__|
324	Injera, bread, rice, noodles, pasta, macaroni, porridge, or other foods made from grains such as tef, oats, maize, barley?		|__|
325	Any commercially fortified baby food like Fafa, Hilina, Cerilak, Cerifam, Mother Choice?		
326	Pumpkin, carrots, squash, or sweet potatoes that are yellow or orange inside?		|__|
327	White potatoes, white yams, bulla, kocho, manioc, cassava or any other foods made from roots?		|__|
328	Any dark green leafy vegetables (kale, dark green lettuce, moringa …)?		|__|
329	Any other vegetable?		
330	Ripe mangoes, ripe papayas (insert other local vitamin a-rich fruits)?		|__|
331	Any other fruit?		|__|
332	Liver, kidney, heart, or other organ meats?		|__|
333	Any meat, such as beef, pork, lamb, goat, chicken?		|__|
334	Egg?		|__|
335	Fresh or dried fish, shellfish, or seafood?		|__|
336	Any foods made from beans, peas, lentils, nuts, or seeds?		|__|
337	Cheese or other food made from milk?		|__|
338	Any sugary foods such as chocolates, sweets, candies, pastries, cakes, or biscuits		|__|
339	Any savory junk foods, such as crisps/chips/salted biscuits/instant noodles?		|__|
340	Any other solid, semi-solid, or soft food?		|__|
341	How many times did *(NAME)* eat solid, semi-solid, or soft foods other than liquids yesterday during the day or at night?	Fill in the number of times.<br>98 = Don't know	|__||__|
342	Did *(NAME)* drink anything from a bottle with a nipple yesterday during the day or night?	1 = Yes<br>0 = No<br>98 = Don't know	|__||__|

For peer review only - http://bmjopen.bmj.com/site/about/guidelines.xhtml

Module 4: KAP of mothers or caregivers on children's care and feeding

I am going to read you some knowledge questions about breastfeeding. Please tell me your answers on these questions.			
401	How long after birth should a baby start breastfeeding?	1 = Immediately, within 1 hour of delivery<br>2 = Some hours later but within 24 hours<br>3 = After 1 day<br>4 = After 2 days<br>5 = After >3 days<br>6 = Does not think a baby should be breastfed<br>98 = Don't know	|__||__|
402	How long should a baby receive nothing other than breast milk?	1 = From birth to six months<br>2 = Other<br>98 = Don't know	|__||__|
403	How often should a baby younger than six months be breastfed or fed with breast milk?	1 = On-demand, whenever the baby wants<br>2 = Other<br>98 = Don't know	|__||__|
404	How much should a child be fed when he/she is sick?	1 = Less frequent than usual<br>2 = Same as usual<br>3 = More than usual<br>98 = Don't know	|__||__|
405	How often should a child be fed when he/she is sick?	1 = Less frequently than usual<br>2 = Same as usual<br>3 = More frequently than usual<br>98 = Don't know	|__||__|
406	At what age should a baby first start to receive foods in addition to breast milk?	Months of age (Specify)<br>98= Don't know	|__||__|
407	At what age should children begin observing fasting days if that is their culture or religion?<br>(If <2 years, enter age in months.)	Years of age (Specify)<br>98=Don't know/remember	|__||__|
408	Have you ever heard of child stunting?	1 = Yes<br>0 = No<br>98 = Don't know	|__||__|
409	What age are children at the highest risk of becoming stunted?	Years of age (Specify)<br>Months of age (Specify)<br>98 = Don't know/remember	|__||__|
410	What are the consequences of stunting for young children?<br>Mark all that are mentioned by the respondent	1 = Higher risk of severe infectious diseases<br>2 = Poor educational performance<br>3 = Weaker immune system<br>4 = Low adult wages<br>5 = Lost productivity<br>6 = Excessive weight gain in later life	|__||__|

For peer review only - http://bmjopen.bmj.com/site/about/guidelines.xhtml

		7 = Increased risk of nutrition-related chronic diseases in adult life<br>8 = Increased mortality rate<br>98= Don’t know<br>99 = Other	
411	Poor diet during pregnancy and the first two years of child age can cause child stunting	1 = Agree<br>2 = Do not agree<br>98 = Don’t know	I__II__I
I am going to read you some statements about breastfeeding and complementary feeding made by other mothers who live in a community like yours. Please tell me if you agree with these statements. Remember, there are no correct answers! I would like to know your opinion.			
412	The colostrum (the "first yellowish milk") is not good for the baby and should be discarded	1 = Strongly disagree<br>2 = Disagree<br>3 = Agree somewhat<br>4 = Agree<br>5 = Strongly agree<br>98 = Don’t know	I__II__I
413	It is good to exclusively breastfeed give a baby only breast milk and no other foods or liquids for the first six months	1 = Strongly disagree<br>2 = Disagree<br>3 = Agree somewhat<br>4 = Agree<br>5 = Strongly agree<br>98 = Don’t know	I__II__I
414	If a child is sick (for example has fever/diarrhea) breastfeeding must be continued	1 = Strongly disagree<br>2 = Disagree<br>3 = Agree somewhat<br>4 = Agree<br>5 = Strongly agree<br>98 = Don’t know	I__II__I
415	A child should eat eggs, cow milk, or meat even on fasting days	1 = Strongly disagree<br>2 = Disagree<br>3 = Agree somewhat<br>4 = Agree<br>5 = Strongly agree<br>98 = Don’t know	I__II__I
416	Eating a meal from different food groups is not necessary until children are old enough to go to school	1 = Strongly disagree<br>2 = Disagree<br>3 = Agree somewhat<br>4 = Agree<br>5 = Strongly agree<br>98 = Don’t know	I__II__I
417	It is good to feed a two years child at least four times each day	1 = Strongly disagree<br>2 = Disagree<br>3 = Agree somewhat<br>4 = Agree<br>5 = Strongly agree<br>98 = Don’t know	I__II__I
418	A mother should eat nutritious food	1 = Strongly disagree	I__II__I

For peer review only

For peer review only - http://bmjopen.bmj.com/site/about/guidelines.xhtml

	(four) times daily from the time of pregnancy	2 = Disagree<br>3 = Agree somewhat<br>4 = Agree<br>5 = Strongly agree<br>98 = Don't know	
419	A mother should take iron folic acid tablets during pregnancy	1 = Strongly disagree<br>2 = Disagree<br>3 = Agree somewhat<br>4 = Agree<br>5 = Strongly agree<br>98 = Don't know	I__I I__I
420	A mother should take iodized salt during pregnancy	1 = Strongly disagree<br>2 = Disagree<br>3 = Agree somewhat<br>4 = Agree<br>5 = Strongly agree<br>98 = Don't know	I__I I__I
421	A mother should take de-worming medicines during pregnancy	1 = Strongly disagree<br>2 = Disagree<br>3 = Agree somewhat<br>4 = Agree<br>5 = Strongly agree<br>98 = Don't know	I__I I__I

For peer review only

Module 5: Adolescent girls (10-19 Years)

Hint; This section is administered for Adolescent girls 10-19 years old. Provide a paper copy of both the informed consent and Assent Form to the respondent Read the consent (for mothers of adolescent girls) and Assent (adolescent girls) form			
501	Girl's code		|__||__|
502	Girl's name	Given name	
503	Girl's age		
504	Girl's education level	1=None<br>1=Primary<br>2=Secondary<br>3=Technical/vocational certificate<br>98=Don't know<br>99=Other (specify)	
505	Girl's marital status	1 = Single<br>2 = Married<br>3 = Divorced<br>4 = Separated<br>5 = Widowed	
506	Girl's religion	1=Orthodox<br>2=Protestant<br>3=Catholic/ other Christian<br>4=Muslim<br>5=No religion<br>98=Don't know<br>Other religion (specify)	
507	Girl's ethnicity	specify	
508	Are you currently a student	1 = Yes<br>0 = No	|__|
509	Were you given any iron/foliate tablets at school or out of school? (***show the tablet)***	1 = Yes<br>0 = No (**Go to 511)**	|__|
510	How many weeks per month have you taken the iron tablets?	Weeks per month (specify)<br>98 = don't know	|__||__|
511	Were you given any drug for intestinal worms at school or out of school in the last six months?	1 = Yes<br>0 = No	|__|
512	Have you received any nutrition counseling in the last six months?	1 = Yes<br>0 = No	|__|
513	Did you receive nutritional assessment services in health facilities when you went for any kind of health service?	1 = Yes<br>0 = No	|__|
514	Is there any food taboo for adolescent girls in your community?	1 = Yes<br>0 = No (**Go to 516**)	|__|
515	Mention types of food taboo?		
Anthropometry and clinical nutrition assessment			
516	Weight (in kg)		
517	Height (in CM)		
518	Waist circumference (in CM)		

For peer review only - http://bmjopen.bmj.com/site/about/guidelines.xhtml

519	Goiter	1 = Yes, 0 = No

For peer review only

For peer review only - http://bmjopen.bmj.com/site/about/guidelines.xhtml

Module 6: Reproductive age women (15-49 Years)

For currently pregnant women			
601	Woman's code		
602	Woman's name	Given name	
603	Woman's age		
604	Woman's education level	1=None<br>2=Primary<br>3=Secondary<br>4=Technical/vocational certificate<br>5=Higher / university/ college<br>98=Don't know<br>99=Other (specify)	
605	Woman's marital status	1 = Single<br>2 = Married<br>3 = Divorced<br>4 = Separated<br>5 = Widowed	
606	Woman's religion	1=Orthodox<br>2=Protestant<br>3=Catholic/ other Christian<br>4=Muslim<br>5=No religion<br>98=Don't know<br>99=Other religion (specify)	
607	Woman's ethnicity	Specify	
608	What was your age at first marriage	Year (specify)<br>96=Not applicable<br>98=Don't know	|__||__|
609	What was your age at your first pregnancy	Year (specify)<br>96=Not applicable<br>98=Don't know	|__||__|
610	Were you pregnant in the last 3 years?	1= Yes<br>0=No **(Go to 629)**	
611	Did you see anyone for antenatal care for the pregnancy?	1 = Yes<br>0 = No (**Go to 629**)	|__|
612	Whom did you see for antenatal care?<br>Probe to identify each type of person and record all	1=Health personnel<br>2 = Doctor<br>3 = Nurse<br>4 = Midwife<br>5= Health officer<br>6 = Health extension worker<br>7 = Other person<br>8 = Traditional birth Attendant<br>99 = Other (specify)	|__||__|
613	Where did you receive antenatal care for this pregnancy?<br>Anywhere else?	1= My home<br>2 = Her home<br>3 = Other home<br>4 = Health center	|__||__|

For peer review only

		5 = Government Hospital<br>6 = Private Clinic or Hospital<br>7 = Health post<br>8 = NGO clinic or hospital<br>99 = Other SPECIFY	
614	How many months pregnant were you when you first received antenatal care for this pregnancy?	Months (Specify)<br>98 = Don't know	|__||__|
615	How many times did you receive antenatal care during this pregnancy?	Number of times (specify)<br>98 = Don't know	|__||__|
616	As part of your antenatal care during this pregnancy, were any of the following done at least once:	1 = Yes 0 = No	
		Was your blood pressure measured?	|__|
		Did you give a urine sample?	|__|
		Did you give a blood sample?	|__|
		Did a health care provider talk with you about which foods to eat while pregnant?	|__|
		Did a health care provider weigh you?	|__|
		Did a health care provider talk with you about your weight?	|__|
		Did a health care provider talk with you about breastfeeding?	|__|
617	During this pregnancy, did you ever receive food or cash assistance from government, an NGO, religious institution or other group?	1 = Yes<br>0 = No (**Go to 619**)	|__|
618	What type of assistance did you receive?	1 = Cash only<br>2 = Food only<br>3 = Cash and food mix<br>99 = Other (specify)	|__||__|
619	During this pregnancy, were you given or did you buy any iron tablets or iron syrup?<br><br>***show tablets/syrup/multiple micronutrient supplement***	1 = Yes<br>0 = No **(Go to 622)**<br>98 = Don't Know **(Go to 622)**	|__||__|
620	Where did you get the iron tablet or syrup from?	1 = Govt. Health Facility<br>2 = Private Health Facility<br>3 = Mobile Clinic<br>4 = CHW<br>5 = [Mass Distribution Campaign – Add Local Name]<br>6 = Pharmacy<br>7 = Shop/Market<br>8 = School<br>98 = Don't Know<br>99= Other	|__||__|
621	During this pregnancy, for how many days did you take the iron tablets?<br>***If answer is not numeric, probe for***	Number of days (specify)<br>98 = Don't Know	|__||__|

For peer review only - http://bmjopen.bmj.com/site/about/guidelines.xhtml

	approximate number of days.		
622	During this pregnancy, did you take any drug for intestinal worms?	1 = Yes<br>0 = No<br>98 = Don't Know	|__||__|
623	During this pregnancy, did any health care provider talk with you about breastfeeding?	1 = Yes<br>0 = No<br>98 = Don't Know	|__||__|
624	During this pregnancy, did you practice fasting?	1 = Yes<br>0 = No<br>98 = Don't Know	|__||__|
625	At your last ANC visit, did the health provider weigh you?	1 = Yes<br>0 = No<br>98 = Don't Know	|__||__|
626	During this pregnancy has your health provider given you information about your weight gain?	1 = Yes<br>0 = No (**Go to 629**)<br>98 = Don't Know	|__||__|
627	During your pregnancy have you been thin for your height?	1 = Yes<br>0 = No<br>98 = Don't Know	|__||__|
628	Did you received treatment for malnutrition?	1 = Yes<br>0 = No	|__||__|
**Anthropometry**			
629	Weight (in kg)		
630	Height (in CM)		
631	MUAC		
632	Waist circumference (in cm)		
633	Goiter	1 = Yes<br>0 = No	

For peer review only

Module 7: Women Dietary Diversity

"Now I'd like to ask you about foods and drinks that you ate or drank yesterday during the day or night, whether you ate it at home or anywhere else.		
Yesterday, during the day or at night did you eat or drink:		
S.N	Question	Response<br>1 = Yes<br>0 = No
701	Woman's code	
702	Woman's name	Given name
703	Woman's age	
704	Woman's education level	0=None<br>1=Primary<br>2=Secondary<br>3=Technical/vocational certificate<br>4=Higher / university/ college<br>98=Don't know<br>99=Other (specify)
705	Woman's marital status	1= Single<br>2= Married<br>3= Divorced<br>4= Separated<br>5= Widowed
706	Woman's religion	1=Orthodox<br>2=Protestant<br>3=Catholic/other Christian<br>4=Muslim<br>5=No religion<br>98=Don't know<br>99=Other religion (specify)
707	Woman's ethnicity	Specify
708	Any vegetables or roots that are orange-colored inside, like: pumpkin, carrots, squash, or sweet potatoes that are yellow or orange inside	|__|
709	Any white roots and tubers or plantains, such as: white potatoes, white yams, manioc/cassava/yucca, cocoyam, taro or any other foods made from white-fleshed roots or tubers, or plantains	|__|
710	Any dark green leafy vegetables, such as: [list examples of any medium-to-dark green leafy vegetables including wild/foraged leaves]	|__|
711	Any fruits that are dark yellow or orange inside, like: ripe mango, ripe papaya	|__|
712	Any other fruits	|__|
713	Any other vegetables	|__|
714	Any meat made from animal organs, such as: liver, kidney, heart or other organ meats or blood-based foods, including from wild game	|__|
715	Any other types of meat or poultry, like: beef, pork, lamb, goat, rabbit, wild game meat, chicken, duck, other birds	|__|

For peer review only - http://bmjopen.bmj.com/site/about/guidelines.xhtml

716	Any eggs	|__|
717	Any fish or seafood, whether fresh or dried	|__|
718	Any beans or peas, such as: mature beans or peas (fresh or dried seed), lentils, or bean/pea products, including hummus, tofu and tempeh	|__|
719	Any nuts or seeds, like tree nut, groundnut/peanut, or certain seeds or nut/seed "butters" or pastes	|__|
720	Any milk or milk products, such as: milk, cheese, yoghurt or other milk products, but not including butter, ice cream, cream or sour cream	|__|
721	Any oils and fats	|__|
722	Any savory and fried snacks, such as: crisps and chips, fried dough, other fried snacks	|__|
723	Any sweets, such as: sugary foods, such as chocolates, candies, cookies/sweet biscuits and cakes, sweet pastries or ice cream	|__|
724	Any sugar-sweetened beverages, like: sweetened fruit juices and "juice drinks", soft drinks/fizzy drinks, chocolate drinks, malt drinks, yoghurt drinks, sweet tea or coffee with sugar	|__|
725	Any condiments and seasonings, such as: ingredients used in small quantities for flavour, such as chilies, spices, herbs, fish powder, tomato paste, flavor cubes or seeds	|__|
726	Any other beverages and foods like tela, tej, bordea, arkea, cheka, tselo	|__|
727	Did you eat anything (meal or snack) OUTSIDE of the home yesterday?	|__|
728	Did you fast yesterday during the day or night?	|__|

For peer review only

For peer review only - http://bmjopen.bmj.com/site/about/guidelines.xhtml

Module 8: SELF-REPORTING QUESTIONNAIRE (SRQ-20)

No	Question	Answer<br>1 = Yes<br>0 = No	
801	Do you often have headaches?		|__|
802	Is your appetite poor?		|__|
803	Do you sleep badly?		|__|
804	Are you easily frightened?		|__|
805	Do your hands shake?		|__|
806	Do you feel nervous, tense or worried?		|__|
807	Is your digestion poor?		|__|
808	Do you have trouble thinking clearly?		|__|
809	Do you feel unhappy?		|__|
810	Do you cry more than usual?		|__|
811	Do you find it difficult to enjoy your daily activities?		|__|
812	Do you find it difficult to make decisions?		|__|
813	Is your daily work suffering?		|__|
814	Are you unable to play a useful part in life?		|__|
815	Have you lost interest in things?		|__|
816	Do you feel that you are a worthless person?		|__|
817	Has the thought of ending your life been on your mind?		|__|
818	Do you feel tired all the time?		|__|
819	Do you have uncomfortable feelings in your stomach?		|__|
820	Are you easily tired?		|__|

For peer review only

Module 9: Women empowerment

901	Identify the most senior mother of the mothers who have a selected child. She is the mother who should respond to the rest of this interview from this point.		|__||__|
902	In the past major growing season (Meher) and minor growing (Belg) season, not including the current season, did you work on the family farm?	1 = Yes<br>0 = No → **Go to 913)**	|__|
903	What sort of work you did on the family farm?	1 = Home (kitchen) gardening	|__|
904		2 = Fieldwork	|__|
905		3 = Cash crop farming	|__|
906		4 = Dairy processing	|__|
907		5 = Poultry rearing	
908		6 = Raising livestock	|__|
909		7 = Fishpond/ aquaculture	|__|
910		99 = Other (specify)	|__|
911	From the work that you did on the farm did your household earn any money?	1 = Yes<br>0 = No<br>98 = Don't know	|__|
912	Who usually decides how the money you earn will be used?<br><br>**READ THE LIST.**	1 = Self<br>2 = Husband<br>3 = Self and husband jointly<br>4 = Someone else	|__|
913	Who usually makes decisions about major household purchases/sell such as cattle or livestock?<br><br>**READ THE LIST.**	1 = Self<br>2 = Husband<br>3 = Self and husband jointly<br>4 = Someone else	|__|
914	Who usually makes decisions about minor household purchases/sell such as spices/oils, soap, utensils, or daily household needs?<br><br>**READ THE LIST.**	1 = Self<br>2 = Husband<br>3 = Self and husband jointly<br>4 = Someone else	|__|
915	Who usually makes decisions about health care for your children?<br><br>**READ THE LIST.**	1 = Self<br>2 = Husband<br>3 = Self and husband jointly<br>4 = Someone else	|__|
916	Do you have husband?	1 = Yes<br>0 = No (**Go to 918**)	|__|
917	Who usually decides how the money your husband earns will be used?<br><br>**READ THE LIST.**	1 = Self<br>2 = Husband<br>3 = Self and husband jointly<br>4 = Someone else	

For peer review only

For peer review only - http://bmjopen.bmj.com/site/about/guidelines.xhtml

918	Do you have children?	1 = Yes<br>0 = No (**Go to 923)**	
919	Does your husband help you care for the children?	1 = Yes<br>0 = No (**Go to 923)**	|__|
920	Does he help care for the children almost every day, at least once a week, or rarely?	1 = Every day<br>2 = At least once a week<br>3 = Rarely	|__|
921	Does your husband help you with household chores like cooking, cleaning the house, fetching water, collecting firewood or other domestic work?	1 = Yes<br>0 = No (**Go to 923)**	|__|
922	Does he help almost every day, at least once a week, or rarely?	1 = Every day<br>2 = At least once a week<br>3 = Rarely	|__|

923	At any time during the previous major seasons (Meher) and minor season (Belg) not including the current season,<br><br>Did you:	Use improved seed varieties for any of your crops?	1 = Yes<br>0 = No<br>98 = Don't know	|__||__|
924		Keep improved varieties of livestock?	1 = Yes<br>0 = No<br>98 = Don't know	|__||__|
925		Use animal manure to improve you crops yield?	1 = Yes<br>0 = No<br>98 = Don't know	|__||__|
926		Use any other source of fertilizer on your crops?	1 = Yes<br>0 = No<br>98 = Don't know	|__||__|
927		Irrigate your crops?	1 = Yes<br>0 = No<br>98 = Don't know	|__||__|
928		Rotate your crops from one field to another when planting?	1 = Yes<br>0 = No<br>98 = Don't know	|__||__|
929		Harvest water during the rains?	1 = Yes<br>0 = No<br>98 = Don't know	|__||__|
930		Practice intercropping?	1 = Yes<br>0 = No<br>98 = Don't know	|__||__|
931	Have you ever taken any steps to reduce soil erosion on your farm?		1 = Yes<br>0 = No (**Go to 936)**<br>98 = Don't know (**Go to 936)**	|__||__|
	What steps did you take to reduce soil erosion?		For each mentioned: 1=Yes 0=No	
932			Plant trees or shrubs	|__|
933			Terracing	|__|

For peer review only

For peer review only - http://bmjopen.bmj.com/site/about/guidelines.xhtml

934		Use drainage system	|__|
935		Other	|__|
936	Have you received any inputs for your farm from a social/government program?	1 = Yes<br>0 = No (**Go to 1001)**	|__|
937	What farm inputs have you received?	For each mentioned: 1=Yes 0=No	
938		Seeds	|__|
939		Improved seeds	|__|
940		Livestock or poultry	|__|
941		Improved varieties of livestock/ poultry	|__|
942		Aquaculture (fish)	|__|
943		Fertilizer	|__|
944		Other	|__|

For peer review only

For peer review only - http://bmjopen.bmj.com/site/about/guidelines.xhtml

Module 10: WASH

No	Question	choices	
1001	What is the main source of drinking water for the household?<br><br>**Do not read list**	1 = Piped into dwelling<br>2 = Piped to yard/plot<br>3 = Piped to neighbour<br>4 = Public tap/standpipe<br>5 = Tube well or borehole<br>6 = Protected well<br>7 = Unprotected well<br>8 = Protected spring<br>9 = Unprotected spring<br>10 = Rainwater<br>11 = Tanker truck<br>12 = Cart with small tank<br>13 = Surface water (river/dam/ Lake/pond/stream/canal/ irrigation channel)<br>14 = Bottled water<br>99 = Other	|__||__|
1002	Do you do anything to your household water to make it safer to drink?	1 = Yes<br>0 = No **(Go to 1004)**<br>98 = Don't know **(Go to 1004)**	|__||__|
1003	What is the main thing you do to make the water safer?	1 = Boil<br>2 = Add bleach/chlorine<br>3 = Strain through a cloth<br>4 = Use water filter (ceramic/ Sand/composite/etc)<br>5 = Solar disinfection<br>6 = Let it stand and settle<br>7 = Other(specify)<br>98 = Don't know	|__||__|
1004	What kind of toilet facility do members of your household usually use? If not possible to determine, ask for Permission to observe the facility.	1 = Flush to piped sewer system<br>2 = Flush to septic tank<br>3 = Flush to pit latrine<br>4 = Flush to somewhere else<br>5 = Flush, don't know where<br>6 = Ventilated improved pit latrine<br>7 = Pit latrine with slab<br>8 = Pit latrine without slab/open pit<br>9 = Composting toilet<br>10 = Bucket toilet<br>11 = Hanging toilet/hanging latrine<br>12 = No facility/bush/field<br>99 = Other (specify)________	|__||__|
1005	How does your HH primarily dispose of HH waste?	1 = Collected by municipality<br>2 = Buried<br>3 = Collected by private establishment<br>4 = Dumped in street/open space	|__||__|

For peer review only - http://bmjopen.bmj.com/site/about/guidelines.xhtml

		5 = Disposed in the compound<br>6 = Dumped in river<br>7 = Burned<br>98 = Other	
1006	Do you have separate cooking room?	1 = Yes<br>2 = No **(Go to 1008)**	
1007	Does the stove or cooking room have a chimney?	1 = yes<br>2 = No	|__||__|
1008	What type of fuel or energy source is used in this cook stove?	1 = Alcohol/ethanol<br>2 = Gasoline/diesel<br>3 = Kerosene/paraffin<br>4 = Coal/lignite<br>5 = Charcoal<br>6 = Wood<br>7 = Straw/shrubs/grass<br>8 = Agricultural crop<br>9 = Animal dung/waste<br>10 = Processed biomass (pellets) or woodchips<br>11 = Garbage/plastic<br>12 = Sawdust<br>96 = Other (specify)	|__||__|
1009	Do you have a confined space (beret/gata) to keep livestock?	1 = Yes<br>0 = No<br>96 = Not applicable	|__|
1010	Do you keep poultry in cages/confined systems (kote)?	1 = Yes<br>0 = No<br>96 = Not applicable	|__|
1011	What do you think are the activities before which you should wash your hands with soap?<br><br>**DO NOT PROMPT.**	For each mentioned: 1=**Yes 0=No**	
1012		Before preparing food	|__|
1013		Before touching or eating food	|__|
1014		Before feeding a child or other person	|__|
1015		Praying	|__|
1016		Don't know	|__|
1017	What do you think are the activities after which you should wash your hands with soap?<br><br>**DO NOT PROMPT.**	For each mentioned: **1=Yes 0=No**	
1018		After defecation or urinating	|__|
1019		After handling animals and their waste	|__|
1020		After housework or fieldwork	|__|
1021		After touching pets or handling animals and their waste	|__|
1022		After blowing nose or coughing	|__|
1023		After cleaning a child's bottom	|__|
1024		None	|__|
1025	What do you think are the reasons to keep poultry and livestock in a confined space?<br>For each mentioned: **1=Yes 0=No**	To keep out of house<br>|__|	
1026		To keep away from water source	|__|
1027		To reduce infectious disease	|__|

For peer review only - http://bmjopen.bmj.com/site/about/guidelines.xhtml

For peer review only

1028	**DO NOT PROMPT.**	To protect livestock/poultry	**|__|**
1029		Other	

For peer review only

Module 11: Food fortification (household coverage of fortifiable foods)

SN	Question	Response	
1101	Does your household use cooking oil to prepare foods or add to foods at home?	1 = Yes<br>0 = No →**Go to 1103**	|__|
1102	The last time your household get cooking oil, where did you get it from?	1=Purchased from market/shop/kiosk/wholesaler/street vendor<br>2= Homemade or obtained from local farm or local small factory/processor….<br><br>3= Received from food aid/social protection program<br>4 = Other (specify): _____________<br><br>98= Don't know/remember	|__||__|
1103	Does your household prepare foods using wheat flour at home, such as bread, kita, injera?	1 = Yes<br>0 = No →**Go to 1105**	|__|
1104	The last time your household get wheat flour, where did you get it from?	1=Purchased from market/shop/kiosk/wholesaler/street vendor<br>2= Homemade or obtained from local farm or local small factory/processor….<br><br>3= Received from food aid/social protection program<br>4 = Other (specify): _____________<br><br>98= Don't know/remember	|__||__|
1105	I would like to check whether the salt used in your household is iodized. May I have a sample of the salt used to cook meals in your household? Test salt for iodine	1=Iodine present<br>2= No iodine<br>3= Household uses salt but there is no salt in household<br>4= Household does not use salt<br>5= Salt not tested, specify reason_________	|__|
1106	The last time your household get salt, where did you get it from?	1=Purchased from market/shop/kiosk/wholesaler/street vendor<br>2= Homemade or obtained from local farm or local small factory/processor….<br><br>3= Received from food aid/social protection program<br>98= Don't know/remember	|__||__|

For peer review only - http://bmjopen.bmj.com/site/about/guidelines.xhtml

		99 = Other (specify): ______________	

For peer review only

For peer review only - http://bmjopen.bmj.com/site/about/guidelines.xhtml

Module 12 – Agriculture practices

About the household			
1201	Does any member of the household own any agricultural land (purchased or own?	1 = Yes<br>0 = No (**Go to 1212)**	|__|
1202	How many hectares of agricultural land do members of this household own?<br><br>*Note: Convert local land measurement unit into hector after discussing with agriculture focal person/AEW.*	Enter total number of hectares (If less than 1, Enter in decimals (example 0.5)<br>Enter 9999 if hectares are not known	|__|__|__|__|
1203	In the past major growing season (Meher) and minor growing (Belg) season, not including the current season, did you work on the family farm?	1 = Yes<br>0 = No (**Go to 1211)**	|__|
	What sort of work did you do on the family farm?<br><br>READ THE LIST	1 = Yes 0 = No	
		1 = Home (kitchen) gardening	|__|
1204		2 = Fieldwork	|__|
1205		3 = Cash crop farming	|__|
1206		4 = Producing dairy	|__|
1207		5 = Rearing poultry	|__|
1208		6 = Raising livestock	|__|
1209		7 = Fishpond/ aquaculture	|__|
1210		99 = Other ………………….	|__|
1211	Does this household own any livestock, herds, other farm animals, or poultry?	1 = Yes<br>0 = No → **Go to 1221**	|__|
	How many of the following animals do this household own?	For each: Enter number. **If none, enter 0**	
1212		Chickens	|__|__|__|
1213		Goats	|__|__|__|
1214		Sheep	|__|__|__|
1215		Donkeys	|__|__|__|
1216		Horses	|__|__|__|
1217		Mules	|__|__|__|
1218		Camels	|__|__|__|
1219		Milk cows	|__|__|__|
1220		Oxen	|__|__|__|

For peer review only

For peer review only - http://bmjopen.bmj.com/site/about/guidelines.xhtml

In the past 2 growing seasons (Meher and Belg), not including the current season, please describe all the crops (cereals, legumes, vegetables, fruits, seeds, and other crops) grown on your household farm.

Please also describe all animal source foods (meat, eggs, milk, dairy, fish, other) that you have produced on your household farm in same period.

Write down all crops and animals mentioned by the respondent. When the respondent has finished, probe for crops and animal source foods not mentioned. Then ask about production/yields in the relevant units. Ask the respondent to estimate the amount of the total production that went to sales, food consumption, and storage/losses/other uses.

	Group	Crop	Did HH cultivate crop? 1 = yes 0 = No	Season 1=Meher 2=Belg 3=Both	Amount	During the previous Major seasons (Meher) and Minor season (Belg) not including the current season		
						Sold	Consumed	Storage, losses, animal feed or other uses
1221	Staples	Maize	|__|		|__|	|__||__|	|__||__|	|__||__|
1222		Teff	|__|		|__|	|__||__|	|__||__|	|__||__|
1223		Wheat	|__|		|__|	|__||__|	|__||__|	|__||__|
1224		Barley	|__|		|__|	|__||__|	|__||__|	|__||__|
1225		Sorghum	|__|		|__|	|__||__|	|__||__|	|__||__|
1226		Millet	|__|		|__|	|__||__|	|__||__|	|__||__|
1227		Rice	|__|		|__|	|__||__|	|__||__|	|__||__|
1228		Emmer wheat (oaths)	|__|		|__|	|__||__|	|__||__|	|__||__|
1229		Other cereals	|__|		|__|	|__||__|	|__||__|	|__||__|
1230	Pulses (legumes)	Bean	|__|		|__|	|__||__|	|__||__|	|__||__|
1231		Haricot bean	|__|		|__|	|__||__|	|__||__|	|__||__|
1232		Lentil (Miser)	|__|		|__|	|__||__|	|__||__|	|__||__|
1233		Grass pea (guaya)	|__|		|__|	|__||__|	|__||__|	|__||__|
1234		Chickpea	|__|		|__|	|__||__|	|__||__|	|__||__|
1235		Field pea (Ater)	|__|		|__|	|__||__|	|__||__|	|__||__|
1236		Soya bean	|__|		|__|	|__||__|	|__||__|	|__||__|

For peer review only

For peer review only - http://bmjopen.bmj.com/site/about/guidelines.xhtml

<table>
<tr><td>1237</td><td></td><td>Other legumes</td><td>|__|</td><td></td><td>|__|</td><td>|__||__|</td><td>|__||__|</td><td>|__||__|</td></tr>
<tr><td>1238</td><td rowspan="8">Oil Crops</td><td>Niger seed (Nug)</td><td>|__|</td><td></td><td>|__|</td><td>|__||__|</td><td>|__||__|</td><td>|__||__|</td></tr>
<tr><td>1239</td><td>Sunflower</td><td>|__|</td><td></td><td>|__|</td><td>|__||__|</td><td>|__||__|</td><td>|__||__|</td></tr>
<tr><td>1240</td><td>Sesame</td><td>|__|</td><td></td><td>|__|</td><td>|__||__|</td><td>|__||__|</td><td>|__||__|</td></tr>
<tr><td>1241</td><td>Linseed</td><td>|__|</td><td></td><td>|__|</td><td>|__||__|</td><td>|__||__|</td><td>|__||__|</td></tr>
<tr><td>1242</td><td>Rapeseed (Gomenzer)</td><td>|__|</td><td></td><td>|__|</td><td>|__||__|</td><td>|__||__|</td><td>|__||__|</td></tr>
<tr><td>1243</td><td>Lupine (Gibto)</td><td>|__|</td><td></td><td>|__|</td><td>|__||__|</td><td>|__||__|</td><td>|__||__|</td></tr>
<tr><td>1244</td><td>Nuts</td><td></td><td></td><td></td><td>|__||__|</td><td>|__||__|</td><td>|__||__|</td></tr>
<tr><td>1245</td><td>Other oil crops</td><td>|__|</td><td></td><td>|__|</td><td>|__||__|</td><td>|__||__|</td><td>|__||__|</td></tr>
<tr><td>1246</td><td rowspan="15">Root crops/ tubers/ vegetables</td><td>Cassava</td><td>|__|</td><td></td><td>|__|</td><td>|__||__|</td><td>|__||__|</td><td>|__||__|</td></tr>
<tr><td>1247</td><td>Enset</td><td>|__|</td><td></td><td>|__|</td><td>|__||__|</td><td>|__||__|</td><td>|__||__|</td></tr>
<tr><td>1248</td><td>Irish potato</td><td>|__|</td><td></td><td>|__|</td><td>|__||__|</td><td>|__||__|</td><td>|__||__|</td></tr>
<tr><td>1249</td><td>Sweet potato</td><td>|__|</td><td></td><td>|__|</td><td>|__||__|</td><td>|__||__|</td><td>|__||__|</td></tr>
<tr><td>1250</td><td>Sweet potato - orange flesh</td><td>|__|</td><td></td><td>|__|</td><td>|__||__|</td><td>|__||__|</td><td>|__||__|</td></tr>
<tr><td>1251</td><td>Onion</td><td>|__|</td><td></td><td>|__|</td><td>|__||__|</td><td>|__||__|</td><td>|__||__|</td></tr>
<tr><td>1252</td><td>Pepper</td><td>|__|</td><td></td><td>|__|</td><td>|__||__|</td><td>|__||__|</td><td>|__||__|</td></tr>
<tr><td>1253</td><td>Tomato</td><td>|__|</td><td></td><td>|__|</td><td>|__||__|</td><td>|__||__|</td><td>|__||__|</td></tr>
<tr><td>1254</td><td>Cabbage</td><td>|__|</td><td></td><td>|__|</td><td>|__||__|</td><td>|__||__|</td><td>|__||__|</td></tr>
<tr><td>1255</td><td>Other light green leafy vegetables</td><td>|__|</td><td></td><td>|__|</td><td>|__||__|</td><td>|__||__|</td><td>|__||__|</td></tr>
<tr><td>1256</td><td>Kale</td><td>|__|</td><td></td><td>|__|</td><td>|__||__|</td><td>|__||__|</td><td>|__||__|</td></tr>
<tr><td>1257</td><td>Other dark green leafy vegetables</td><td>|__|</td><td></td><td>|__|</td><td>|__||__|</td><td>|__||__|</td><td>|__||__|</td></tr>
<tr><td>1258</td><td>Carrot</td><td>|__|</td><td></td><td>|__|</td><td>|__||__|</td><td>|__||__|</td><td>|__||__|</td></tr>
<tr><td>1259</td><td>Other roots or tubers</td><td>|__|</td><td></td><td>|__|</td><td>|__||__|</td><td>|__||__|</td><td>|__||__|</td></tr>
<tr><td>1260</td><td>Other vegetables</td><td>|__|</td><td></td><td>|__|</td><td>|__||__|</td><td>|__||__|</td><td>|__||__|</td></tr>
<tr><td>1261</td><td rowspan="6">Perennial crops/ fruits</td><td>Coffee</td><td>|__|</td><td></td><td>|__|</td><td>|__||__|</td><td>|__||__|</td><td>|__||__|</td></tr>
<tr><td>1262</td><td>Chat (khat)</td><td>|__|</td><td></td><td>|__|</td><td>|__||__|</td><td>|__||__|</td><td>|__||__|</td></tr>
<tr><td>1263</td><td>Banana</td><td>|__|</td><td></td><td>|__|</td><td>|__||__|</td><td>|__||__|</td><td>|__||__|</td></tr>
<tr><td>1264</td><td>Orange</td><td>|__|</td><td></td><td>|__|</td><td>|__||__|</td><td>|__||__|</td><td>|__||__|</td></tr>
<tr><td>1265</td><td>Mango</td><td>|__|</td><td></td><td>|__|</td><td>|__||__|</td><td>|__||__|</td><td>|__||__|</td></tr>
<tr><td>1266</td><td>Hop</td><td>|__|</td><td></td><td>|__|</td><td>|__||__|</td><td>|__||__|</td><td>|__||__|</td></tr>
</table>

For peer review only - http://bmjopen.bmj.com/site/about/guidelines.xhtml

		(Gesho)						
1267		Avocado	|__|		|__|	|__||__|	|__||__|	|__||__|
1268		Lemon	|__|		|__|	|__||__|	|__||__|	|__||__|
1269		Papaya	|__|		|__|	|__||__|	|__||__|	|__||__|
1270		Guava	|__|		|__|	|__||__|	|__||__|	|__||__|
1271		Water Melon	|__|		|__|	|__||__|	|__||__|	|__||__|
1272		Tirngo fruit	|__|		|__|	|__||__|	|__||__|	|__||__|
1273		Other perennial crops	|__|		|__|	|__||__|	|__||__|	|__||__|
1274		Other fruits	|__|		|__|	|__||__|	|__||__|	|__||__|

For peer review only - http://bmjopen.bmj.com/site/about/guidelines.xhtml

<table>
<tr><th rowspan="3"></th><th rowspan="3">Group</th><th rowspan="3">Animal source food (unit)</th><th rowspan="3">Does HH produce?
1=yes
0=no
<u>(If no, skip to the next item)</u></th><th rowspan="3">Amount</th><th colspan="4">During the previous Major seasons (Meher) and Minor season (Belg) not including the current season</th></tr>
<tr><th rowspan="2">Season
1=Meher
2=Belg
3=Both</th><th colspan="3">How much?</th></tr>
<tr><th>Sold</th><th>Consumed</th><th>Storage, losses, animal feed or others</th></tr>
<tr><td>1275</td><td rowspan="11">All</td><td>Chicken eggs</td><td></td><td>|__|</td><td>|__|</td><td>|__|__|</td><td>|__|__|</td><td>|__|__|</td></tr>
<tr><td>1276</td><td>Chicken meat</td><td></td><td>|__|</td><td>|__|</td><td>|__|__|</td><td>|__|__|</td><td>|__|__|</td></tr>
<tr><td>1277</td><td>Goat milk</td><td></td><td>|__|</td><td>|__|</td><td>|__|__|</td><td>|__|__|</td><td>|__|__|</td></tr>
<tr><td>1278</td><td>Goat meat</td><td></td><td>|__|</td><td>|__|</td><td>|__|__|</td><td>|__|__|</td><td>|__|__|</td></tr>
<tr><td>1279</td><td>Camel milk</td><td></td><td>|__|</td><td>|__|</td><td>|__|__|</td><td>|__|__|</td><td>/__/__/</td></tr>
<tr><td>1280</td><td>Sheep meat</td><td></td><td>|__|</td><td>|__|</td><td>|__|__|</td><td>|__|__|</td><td>/__/__/</td></tr>
<tr><td>1281</td><td>Cow milk</td><td></td><td>|__|</td><td>|__|</td><td>|__|__|</td><td>|__|__|</td><td>/__/__/</td></tr>
<tr><td>1282</td><td>Cow other dairy</td><td></td><td>|__|</td><td>|__|</td><td>|__|__|</td><td>|__|__|</td><td>/__/__/</td></tr>
<tr><td>1283</td><td>Beef</td><td></td><td>|__|</td><td>|__|</td><td>|__|__|</td><td>|__|__|</td><td>/__/__/</td></tr>
<tr><td>1284</td><td>Other meat (e.g. camel, horse)</td><td></td><td>|__|</td><td>|__|</td><td>|__|__|</td><td>|__|__|</td><td>/__/__/</td></tr>
<tr><td>1285</td><td>Farmed fish</td><td></td><td>|__|</td><td>|__|</td><td>|__|__|</td><td>|__|__|</td><td>/__/__/</td></tr>
</table>

For peer review only

Module 13: Household food insecurity

Now I would like to ask you some questions about food. During the last 12 MONTHS, was there a time when:			
SN	Questions	Answers	
1301	You or others in your household worried about not having enough food to eat because of a lack of money or other resources?	1=Yes<br>0= No<br>98=Don't know<br>97=Refused	|__||__|
1302	Still thinking about the last 12 MONTHS, was there a time when you or others in your household were unable to eat healthy and nutritious food because of a lack of money or other resources?	1=Yes<br>0= No<br>98=Don't know<br>97=Refused	|__||__|
1303	Was there a time when you or others in your household ate only a few kinds of foods because of a lack of money or other resources?	1=Yes<br>0= No<br>98=Don't know<br>97=Refused	|__||__|
1304	Was there a time when you or others in your household had to skip a<br>meal because there was not enough money or other resources to get<br>food?	1=Yes<br>0= No<br>98=Don't know<br>97=Refused	|__||__|
1305	Still thinking about the last 12 MONTHS, was there a time when you or others in your household ate less than you thought you should because of a lack of money or other resources?	1=Yes<br>0= No<br>98=Don't know<br>97=Refused	|__||__|
1306	Was there a time when your household ran out of food because of a lack of money or other resources?	1=Yes<br>0= No<br>98=Don't know<br>97=Refused	|__||__|
1307	Was there a time when you or others in your household were hungry but did not eat because there was not enough money or other resources for food?	1=Yes<br>0= No<br>98=Don't know<br>97=Refused	|__||__|
1308	Was there a time when you or others in your household went without eating for a whole day because of a lack of money or other resources?	1=Yes<br>0= No<br>98=Don't know<br>97=Refused	|__||__|

For peer review only

For peer review only - http://bmjopen.bmj.com/site/about/guidelines.xhtml

Module 14: Employment and social protection

S.N.	Questions	Response	
1401	Since last year, what has been the main livelihood or income source of the HH?<br><br>**(DO NOT READ LIST. PROBE FOR ONE RESPONSE)**	1 = Sale of self-produced<br>2 = horticulture crops Sale of self-produced field crops<br>3 = Own business (including commerce, livestock rearing)<br>4 = Wage employment<br>5 = Remittances<br>6 = Property income<br>7 = Government transfers/NGO support<br>8 = Pension<br>99 = Other	|__||__|
1402	Since last year, have there been other livelihood or income sources for the HH?<br><br>**(DO NOT READ LIST. PROBE FOR ALL RESPONSES)**	1 = Sale of self-produced<br>2 = horticulture crops Sale of self-produced field crops<br>3 = Own business (including commerce, livestock rearing)<br>4 = Wage employment<br>5 = Remittances<br>6 = Property income<br>7 = Government transfers/NGO support<br>8 = Pension<br>99 = Other	|__||__|
1403	Since last year, did anyone in your HH receive any kind of food or cash assistance from the government, NGO, or other agencies? *Clarify: This is not formal employment or pension. However, it may or may not be conditional on work.*	1 = Yes<br>0 = No<br>98 = Don't know	|__||__|
1404	Since last year, which members of this HH were targeted to receive this support?	1 = All HH members<br>2 = Specific HH members<br>98 = Don't know	|__||__|
1405	Which specific HH members received food or cash assistance?<br>*Clarify: This includes children whose parents receive cash on their behalf.*	Link this back to the HH roster and have interviewer select names.<br>98 = Don't know	|__||__|
1406	Which of these categories apply to the persons who received food or cash assistance?<br><br>(READ RESPONSES ALOUD. SELECT ALL THAT APPLY)	1 = Pregnant women<br>2 = Lactating women<br>3 = Children under 5 years<br>4 = Elderly<br>5 = Disabled person<br>6 = None of the above	|__||__|
1407	Since last year, which food or social assistance program did members of the HH receive support from? (DO NOT READ LIST ALOUD. PROBE FOR ALL RESPONSES)	1 = PSNP<br>2 = Community Care Coalition<br>3 = Other assistance program<br>98 = Don't know	|__||__|
1408	Since last year, what was the form of assistance that members of your HH received form these programs: food, cash or both food and cash?	1 = Cash only transfer<br>2 = Food only transfer<br>3 = Cash and food mix<br>99 = Other<br>98 = Don't know	|__||__|

For peer review only - http://bmjopen.bmj.com/site/about/guidelines.xhtml

1409	Is this HH currently receiving food or cash?	1 = Yes<br>0 = No<br>98 = Don’t know	|__||__|
1410	Since last year, how has your HH used the food received? (READ RESPONSES ALOUD. SELECT ALL THAT APPLY)	1 = HH consumption<br>2 = Sold food for cash<br>3 = Other activities<br>98 = Don’t know	

For peer review only

1501.	How do you preserve soil fertility?<br><br>***[Multiple answer is allowed**! Do not read the choices. Listen and mark the one they mention*]	1 = Fertilization (Chemicals, animal manure, green manure etc)
		2 = Crop rotation (Cultivation of a series of dissimilar types of crops in the same area in sequential seasons)
		3=Intercropping (Cultivation of two or more dissimilar types of crops in the same area in the same season)
		4=Tillage
		99=Other (specify): ....
1502.	Do you apply fertilizers?	1= Yes
		0= No (Go to 1506)
1503.	Which kinds of fertilizers do you use more often?	1= Chemical fertilizers
		2 = Organic fertilizers (non-chemicals like animal manure, green manure, compost, etc.) (Go to→1505)
1504.	Which chemical fertilizers are used most often, can you specify the type/name?	1 = UREA
		2 = DAP
		3= NPS
		98= Don't know
		99= Other (specify): ....
1505.	Which organic fertilizers are used more often, can you specify the type?	1 = Livestock manure
		2 = Poultry manure
		99=Other (specify) ....
1506.	What is the most dominant cereals/crop you produce on your farm?<br>***[Only one answer is allowed**! Do not read the choices. Listen, mark/specify the one they mention*]	1 = Wheat
		2 = Teff
		3 = Maize
		99= Other (Specify): ....
1507.	How far is your agricultural land from your house?<br>*[In case they own many farms, Consider only the one with the dominant crop]*	1= within 500 meter radius
		2= 500 -1000 meter radius
		3= 1000 - 3000 meter radius
		4= More than 3000 meter

For peer review only - http://bmjopen.bmj.com/site/about/guidelines.xhtml

Module 15: Soil information questionnaire

Observational checklist for soil sampling

For peer review only

For peer review only - http://bmjopen.bmj.com/site/about/guidelines.xhtml

1	Region
2	Zone
3	Woreda
4	Kebele
5	Gote Code
6	Household Code
	Sample code …………………..
7	**Crop history**
7.1	Last two-year crop (Please, specify the crop harvested in 2011, growing season) …………………
7.2	Last crop (Please, specify the crop harvested from the previous growing season) …………………
7.3	Crop to be planted for the current season………………………
7.4	Please write the intended planting dates (Year and Month) ……………………….,
7.5	When was the **last time** the agricultural field gets tilled?
	1 = 0 to 3 months ago
	2 = 3 to 6 months ago
	1 = 6 to 12 months ago
	2 = before a year
8	**Fertilizer utilization**
8.1	Which fertilizer is applied
	1 = Chemical fertilizer
	2 = Organic fertilizer **(Go to 8.4)**
	3 = Both 1&2
	4 = Fertilizer not applied **(Go to 9)**
8.2	Which Chemical fertilizer is applied
	1 = UREA
	2 = DAP
	3 = NPS
	4 = Other, please specify……………………………………
8.3	Please write the last date (Year and Month) you applied chemical fertilizer?…………
8.4	Which Organic fertilizer is applied
	1 = Animal Manure
	2 = Green Manure
	3 = Compost
	4 = Other, please specify……………………………………
8.5	Please write the **last date** (Year and Month) you applied organic fertilizer?…………
9	**Soil characteristics**

For peer review only

9.1	Observation: What is the colour of the soil you are about to sample
	1 = Dark brown/Black
	2 = Red
	3 = Grey
	4 = Other, please specify…………………………………..
9.1	Observation: Field area landscape
	1 = plains/level grounds
	2 = Sloppy/Inclined
9.4	Observation: Is there a standing crop on the sampling field or to the nearby farmland.
	1 = Yes
	2 = No
9.3	Observation: Is sampling field tilled/ is it being tilled at the time of sampling.
	1 = Yes
	2 = No
10	Distance of the farmland to the nearby houses
	1 = below 100meter
	2 = 100 to 500meter
	3 = 500 to 1000meter
	4 = More than 1000meter
11	Please take picture for the surrounding environment i.e., plot, houses, anything permanent or even moving cattle
12	Please capture GPS for the sampled farmland
13	Name of sample collector………………………………………….
	LIST ANY ABNORMAL CONDITIONS OR SPECIFIC INFORMATION DESIRED:

For peer review only

Module 16: A Dietary assessment questionnaire

Note for the data collectors: Among the household members, this module questionnaire is to be filled for the child under-five years of age and women of the reproductive age in the household.

Part 1: Before 24-hr recall

**24-hour dietary recall**			
**EA code |__|__|__| Household code |__|__| Line Number |__|__| Child ID |__|__|**<br>**Unique ID Woman: |__|__|__|__|__|__|__| Unique ID Child: |__|__|__|__|__|__|__|__|__|**<br><br>Interview Date: Date - _ _/_ _/_ _ _ _ **Day -** 01=Mon 02=Tue 03=Wed 04=Thu 05=Fri 06=Sat 07=Sun<br>Date of food intake - _ _/_ _/_ _ _ _			
	**Question**	**Coding category**	**skip**
1.	Enumerator Code:	______________________________	
2.	For which target group is the recall being done?	0. Woman 1. Child	
3.	Recall number	0. Recall 1<br>1. Recall 2	
4.	Name of the woman interviewed	____________________	
5.	Age of the woman (in complete years)	Age in years (__________)	
6.	Name of child	______________________________	
7.	Date of birth (DOB): Use Ethiopian calendar	|__|__|/|__|__|/20|__|__|	
8.	Age of the child (in complete month)	________months	
9.	Child's sex:	0=Male 1= Female	
10.	Food weighing scale number:	|__|__|	
11.	Was yesterday's food intake different from your usual diet?	1=Yes 0=No	No →13
12.	If yes,	1=Holyday/celebration<br>2=I was sick<br>3=Other	
13.	Was [child name] yesterday's food intake different from your usual diet?	1=Yes<br>0=No	No →15

For peer review only - http://bmjopen.bmj.com/site/about/guidelines.xhtml

14.	If yes,	1=Holyday/celebration<br>2=I was sick<br>3=Other	
15.	Did you take medicine/supplement yesterday?	1=Yes<br>0=No<br>***If yes, name:*** ___________________	
16.	Did [child name] take medicine/supplement yesterday?	1=Yes<br>0=No<br>***If yes, name:***_________________	

For peer review only

For peer review only - http://bmjopen.bmj.com/site/about/guidelines.xhtml

Procedures to collect the required information

1. **Pass 1: list all foods and drinks consumed during the 24-hour period.**
   Now I would like to ask you about the foods and drinks that [YOU/ YOUR CHILD] consumed yesterday from the time you work up until you went to sleep, sunrise yesterday to sunrise today. Please list all foods or drinks you ate, weather you ate or drank them at home or somewhere else. Please think about snacks and small meals as well as main meals.

   1) "WHAT WAS THE FIRST THING [you/ your child] ATE YESTERDAY AFTER SUNRISE?"
   2) "WHEN WAS THAT"
   3) "DID [you/your child] HAVE ANYTHING WITH THAT?
   4) "WHAT DID [you/he/she] HAVE?"
   5) "WHAT IS THE NEXT THING [you/ your child] ATE OR DRANK AND WHEN WAS THAT?"
   6) REPEAT questions 3-5 until you have a full record for both DAY AND NIGHT
      a. The reference period is from sunrise yesterday to sunrise this morning. If they wake up at a different time than sunrise, you can use the time from waking up yesterday until waking up today

2. **Pass 2: get more detail about each food**.
   7) "NOW, PLEASE DESCRIBE EACH FOOD [you/ your child] ATE YESTERDAY"
   8) "WHAT TYPE WAS IT?"
   9) "WHERE DID YOU GET IT?"
   10) "WHAT ARE THE INGREDIENTS?"
      a. Use standard "probes" (probing questions) to get these details for each food.
   11) "HOW MUCH DID THIS RECIPE MAKE?" or "WHAT WAS THE TOTAL AMOUNT THIS MADE?"
   12) "HOW WAS The Recipe PREPARED?"
      a. Identify the cooking methods used (particularly if raw, fermented, or fried in oil).

3. **Pass 3: estimate the amount consumed of each food on the list**

   13) "HOW MUCH OF [name the first food] DID [you/ your child] CONSUME?"
      a. Help the mother remember and **estimate the amount** of each food or recipe that her child ate and that she herself ate.

   14) "WAS ANY LEFT OVER?"
      a. If any food is leftover from what the mother served to the child, enter that amount.

   15) "PLEASE HELP ME ESTIMATE THE AMOUNT OF FOOD YOU ARE OR USED IN THE RECIPE"

   Use following portion size estimation method to estimate the amount of food/ingredient eaten or used in a recipie 1. Direct weight (g) 2.Proxy weight (g) 3. Water (g) 4. Number 5. Other (specify).

4. **Pass 4: verify everything consumed**
   a. Quickly read the information back to the respondent, "HAVE I FORGOTTEN TO ADD ANYTHING?"

For peer review only - http://bmjopen.bmj.com/site/about/guidelines.xhtml

Part 2. Quick list

Pass 1

Please describe the foods the foods and drinks that [YOU/ YOUR CHILD] consumed yesterday from the time you work up until you went to sleep (sunrise yesterday to sunrise today). Please list all foods or drinks you ate, weather you ate or drank them at home or somewhere else. Please think about snacks and small meals as well as main meals.

Write down all foods and drinks mentioned. When composite dishes are mentioned, ask for the list of ingredients
When the respondent has finished, probe for meals and snacks not mentioned.

For peer review only

Early morning	Mid-morning	Noon	Afternoon	Evening	Late evening

For peer review only - http://bmjopen.bmj.com/site/about/guidelines.xhtml

For peer review only

For peer review only - http://bmjopen.bmj.com/site/about/guidelines.xhtml

Food No.	What was the first thing [YOU/ YOUR CHILD] ate or drank after sunrise yesterday? Any else?	Time of meal	Please describe this food / beverage/ ingredient:	How was this prepared ?	Place of preparation	How was the food / Ing. [illegible]	Amount served	Amount left over	Amount eaten	Recipe information: State of each [illegible]	Cooking method of [illegible]	Total amount of recipe [illegible]	Links to food/ [illegible]
1													
	Ingredient:		Description										
					NA								
					NA								
					NA								
					NA								
					NA								
2													
					NA								
					NA								
					NA								
					NA								
					NA								
3													
					NA								
					NA								
					NA								
					NA								
					NA								

Time of meal: 1. Early morning 2. Mid-Morning 3. Noon 4. Afternoon 5. Evening 6. Late evening

Place of preparation: 1. Home 2. Outside home

How was it prepared: 1=raw/ no change/ as purchased; 2=fermented; 3=fried; 04=cooked or boiled – wet heat; 5=baked/ grilled/ broiled – dry heat; 6=local miller; 7=blanched (dipped in boiling water); 8=other

For peer review only - http://bmjopen.bmj.com/site/about/guidelines.xhtml

For peer review only

Food No.	What was the first thing [YOU/ YOUR CHILD] ate or drank after sunrise yesterday? Any else?	Time of meal	Please describe this food / beverage/ ingredient:	How was this prepared?	Place of preparation	How was the food / Ing. [illegible]	Amount served	Amount left over	Amount eaten	Recipe information			
										State of each [illegible]	Cooking method of [illegible]	Total amount of recipe [illegible]	Links to food/ [illegible]
1													
	Ingredient:		Description										
					NA								
					NA								
					NA								
					NA								
					NA								
2													
					NA								
					NA								
					NA								
					NA								
					NA								
3													
					NA								
					NA								
					NA								
					NA								

For peer review only

For peer review only - http://bmjopen.bmj.com/site/about/guidelines.xhtml

	What was the first	Time of	Please describe this food /	How was	Place of	Ho	Am	Am	Am	Recipe information			
					NA								

Time of meal: 1. Early morning 2. Mid-Morning 3. Noon 4. Afternoon 5. Evening 6. Late evening

Place of preparation: 1. Home 2. Outside home

How was it prepared: 1=raw/ no change/ as purchased; 2=fermented; 3=fried; 04=cooked or boiled – wet heat; 5=baked/ grilled/ broiled – dry heat; 6=local miller; 7=blanched (dipped in boiling water); 8=other

For peer review only

Module 17: Biomarkers collection tools

PRESCHOOL AGE CHILDREN (6-59 MONTHS)
ETHIOPIAN FOOD AND NUTRITION STRATEGY BASELINE SURVEY 2020/21
Biochemical and Health Related Data Collection Tool

IDENTIFICATION	
**HH00.** CLUSTER (EA) NAME................................	**HH01**. CLUSTER NUMBER: ☐☐☐
**HH02**. HH NUMBER: ☐☐	**HH03**. RESIDENCE (RURAL=1, URBAN=2): ☐☐
**HH04**. RESPONDENT LINE NUMBER: (SHOULD BE MOTHER/CAREGIVER) ☐☐	**HH05** CHILD LINE NUMBER ☐☐
**HH06. INTERVIEWER NAME**__________________ **CODE:**___________________	**HH07. TEAM LEADER, NAME**:__________________ **CODE:**____________________
**HH08. SUPERVISOR NAME:**____________________ **CODE.**____________________	

PRESCHOOL CHILDREN 6-59 MONTHS OLD

PART I: CHILD HEALTH QUESTIONS

I would like to ask you some health and food questions about your child. Fill or Circle the correct answer

S.N	Questions	Response	SKIP
1	**What is the birth date of the child? In day/month/ year (How many months old is this child?)**<br><br>NOTE FOR INTERVIEWERS<br>*(Screening question to verify that the date of birth of the child)*	Birth Date: ____________<br><br>(Day/Month/Year)<br><br>Age in months ☐☐	
2	**Has (**child's name**) been diagnosed with anemia in the past 6 months?**	No............................0<br>Yes ....1<br>Don't know.............98	

For peer review only - http://bmjopen.bmj.com/site/about/guidelines.xhtml

PART II: CHILD BIOCHEMICAL MEASUREMENT

Consent given for: (Y OR N) **PL01** Blood ☐ **PL02** Stool ☐	
**PL03** Code for Laboratory Technician: ☐☐	Lab Tech Name ___________________________
**PL04** BLUE TOP TUBE (METAL FREE)<br>Not collected =00.0 Refused = 77.7	ML. ☐☐ • ☐
**PL05** PURPLE TOP TUBE (EDTA)<br>Not collected =00.0<br>Refused = 77.7	ML. ☐☐ • ☐
**PL06** RED TOP TUBE (EDTA)<br>Not collected =00.0<br>Refused = 77.7	ML. ☐☐ • ☐
**PL07** Date blood sample taken (Ethiopian Day/Month/Year)	Date: _______/________/___________<br>Day / Month / Year
**PL08** TIME BLOOD DRAW (Ethiopian time)	Blood draw ___ ___ : ___ __ Hour Minute
**PL09 When did you eat your most recent meal (food)?** (Ethiopian time)	___ ___ : ___ ___<br>Hour Minute
**PL10** MALARIA RESULTS (RDK)	NEGATIVE...0<br>POSITIVE P FALCIPARUM ....1<br>Positive P VIVAX.................................2<br>INVALID...3
**PL11** FEVER in last 24 HR?	NO...0<br>YES 1
**PL12 HEMOGLOBIN** RESULTS	g/dL ☐☐ • ☐
**In order to determine if you have worms in the stool, we would like to collect a stool sample from your child. If you can provide this now, we appreciate it. If not now, we can come back to pick up the sample at a later time.**<br>*INSTRUCTIONS IF UNABLE TO PRODUCE AT WILL*:<br>**For stool**: We will return tomorrow to pick up your stool. We would like the freshest stool you can give us. Please use one cup to collect the first stool you pass.	
**PL13** STOOL COLLECTED?	NO............................0 YES 1
**PL14** Date stool sample taken (Ethiopian Day/Month/Year)	Date: _______/________/___________<br>Day / Month / Year
**PL15** TIME: STOOL COLLECTED (Ethiopian time)	___ ___ : ___ ___

For peer review only - http://bmjopen.bmj.com/site/about/guidelines.xhtml

	Hour Minute
**PL16** TIME: STOOL PASSED, Ethiopian time (as recorded on cup)	___ ___ : ___ ___<br>Hour Minute
**PL17 Time Blood centrifuged (Ethiopian time)**	___ ___ : ___ ___<br>Hour Minute

Thank you for completing this interview.

INTERVIEWER'S OBSERVATIONS

TO BE FILLED IN AFTER COMPLETING INTERVIEW

COMMENTS ABOUT RESPONDENT:

____

____

____

____

____

____

____

____

SCHOOL AGE CHILDREN 6-12 YEARS

ETHIOPIAN FOOD AND NUTRITION STRATEGY BASELINE SURVEY 2020/21

Biochemical and Health Related Data Collection Tool

IDENTIFICATION	
**SG01**. CLUSTER NUMBER: ☐☐☐	
**SG02**. HH NUMBER: ☐☐	
**SG03**. RESPONDENT LINE NUMBER: (SHOULD BE MOTHER/CAREGIVER) ☐☐	
**SG04** SCHOOL CHILD LINE NUMBER ☐☐	

In general, for children 6-10 years of age: get parental report (ask the questions of the caretaker and enter the child's name into the parentheses)
For children 11-12 years of age who are present and can provide information: get self-report (ask questions directly of the child and enter "you" or "yourself" into the parentheses)

For peer review only - http://bmjopen.bmj.com/site/about/guidelines.xhtml

PART I: CHILD HEALTH RELATED QUESTIONS

No.	QUESTION	CODING CATEGORIES		SKIP
**S1**	How old are you/is your child?<br>***(Verify that the age is the same age as written on the household listing)***	Years		
**S2**	Have you/ your child ever attended school?	No............................<br>Yes	00<br>01	**00** →S4
**S3**	What is the highest level of school (name of child) completed?	None............................0<br>Primary 1		

PART II: CHILD BIOCHEMICAL MEASUREMENT

Verbal consent given for: **SL01** Blood **SL02** Urine **SL03** Stool<br>0= No OR 1= yes	
**SL04** Phlebotomist Code	
**SL5** BLUE TOP TUBE (METAL FREE)<br>**Did not work =00.0**<br>**Refused = 77.7**	ML. __ __ • __
**SL6** PURPLE TOP TUBE (EDTA)<br>**Did not work =00.0**<br>**Refused = 77.7**	ML. __ __ • __
**SL7** REDTOP TUBE (EDTA)<br>**Did not work =00.0**<br>**Refused = 77.7**	ML. __ __ • __
**SL8** DATE BLOOD SAMPLE TAKEN (Ethiopian calendar)	Date: ______/_______/___________<br>Day / Month / Year
**SL9** TIME BLOOD DRAW (Ethiopian time)	Blood draw ___ ___ : ___ ___<br>Hour Minute
**SL10 When did you eat your most recent meal (food)?** (Ethiopian time)	Last Meal Eaten ___ ___ : ___ ___<br>Hour Minute
**SL11** FEVER in last 24 HR? (Since same time yesterday)	No..00<br>Yes 01
**SL12** MALARIA RESULTS (RDK)	NEGATIVE....................................00<br>POSITIVE P *falciparum* ....01<br>POSITIVE P *vivax* ....02<br>INVALID 03

For peer review only - http://bmjopen.bmj.com/site/about/guidelines.xhtml

**SL13** HEMOGLOBIN RESULTS	g/dL [ ][ ] . [ ]
**SL14** Is that finger prick or venous sample taken?	Finger prick……………………………….00<br>Venous ………………………………….01
**In order to determine if you have blood in urine or worms in stool we would like to collect a urine and stool sample. If you can provide this now, we appreciate it. If not now, we can come back to pick up the sample at a later time.**	
**SL15 U**rine collected?	No…………………………………………………00<br>yes ………………………………………………….01
**SL16** Blood in urine RESULTS	Negative……………………………………………00<br>positive ……………………………………………….01
**SL17** Stool collected?	No…………………………………………………….00<br>yes…………………………………………………….01
**SL18** Date and **t**ime when stool passed by the respondent (as recorded on cup) (Ethiopian time)	Date: __/_____/______ and ___ ___ : ___ ___<br>Day / Month /Year Hour Minute
**SL19** Date stool sample taken (Ethiopian calendar)	Date: _______/________/___________<br>Day / Month / Year
**SL20** Time when stool collected from the respondent (Ethiopian time)	___ ___ : ___ ___<br>Hour Minute
**SL21** TIME BLOOD centrifuged (Ethiopian time)	___ ___ : ___ ___<br>Hour Minute

Thank the respondent and tell them that the lab team will be arriving later.

<u>INTERVIEWER'S OBSERVATIONS</u>

TO BE FILLED IN AFTER COMPLETING INTERVIEW

COMMENTS:

____
____

____
____

For peer review only - http://bmjopen.bmj.com/site/about/guidelines.xhtml

ADELESCENT GIRLS (10-19 YEARS)
ETHIOPIAN FOOD AND NUTRITION STRATEGY BASELINE SURVEY 2020/21
Biochemical and Health Related Data Collection Tool

IDENTIFICATION	
**HH00.** CLUSTER (EA) NAME.................................	**HH01**. CLUSTER NUMBER: ☐☐☐
**HH02**. HH NUMBER: ☐☐	**HH03**. RESIDENCE (RURAL=1, URBAN=2): ☐☐
**HH04**. RESPONDENT LINE NUMBER: (SHOULD BE MOTHER/CAREGIVER) ☐☐	**HH05** WOMEN LINE NUMBER ☐☐
**HH06**. INTERVIEWER NAME__________________ CODE:____________________	**HH07**. TEAM LEADER, NAME:__________________ CODE:_____________________
**HH08.** SUPERVISOR NAME:____________________ CODE.____________________	

PART I: HEALTH RELATED QUESTIONS

I would like to ask you some health and food questions about yourself. Fill or Circle the correct answer

No.	Question	Coding categories	Skip
1	How old are you?<br>(*verify that the age is the same age as written on the household listing*)	☐ Years	
2	Have you been diagnosed with anemia in the past six months?	No...0<br>Yes ....1<br>Don't know............................98	
3	Do you smoke? (do not include the powder and chew type)	No...........................0<br>Yes 1	

PART II: ADOLESCENT BIOCHEMICAL MEASUREMENT

Consent given for: 0= No or 1= Yes AG**01** Blood ☐ AG**L02** Stool ☐	
**AG03** BLUE TOP TUBE (METAL FREE)<br>**Did not work =00.0**	ML. ☐☐ ● ☐

For peer review only - http://bmjopen.bmj.com/site/about/guidelines.xhtml

**Refused = 77.7**	
**AG04** PURPLE TOP TUBE (EDTA)<br>**Did not work =00.0**<br>**Refused = 77.7**	ML. ☐☐ • ☐
**AG05** REDTOP TUBE (EDTA)<br>**Did not work =00.0**<br>**Refused = 77.7**	ML. ☐☐ • ☐
**AG06** Date blood sample taken (Ethiopian calendar)	Date: ______/_______/___________<br>Day / Month / Year
**AG07** TIME BLOOD DRAW (Ethiopian time)	Blood draw ___ ___ : ___ ___<br>Hour Minute
**AG08 When did you eat your most recent meal (food)?** (Ethiopian date and time)	______/_______/___________ __ ___ : ___ ___<br>Date /Month/ Year Hour Minute
**AG09 Is it Finger prick or venous blood sample taken?**	01 Finger prick<br>02 Venous
**AG09** MALARIA RESULTS (RDT)	NEGATIVE.. 00<br>POSITIVE P *falciparum* .... 01<br>POSITIVE P *vivax* .... 02<br>POSITIVE FOR BOTH P *falciparum and* P *vivax ...... 03*<br>INVALID 04
**AG10 HEMOGLOBIN** RESULTS	g/dL ☐☐ • ☐
**In order to determine if you have worms in the stool we would like to collect a stool sample. If you can provide this now, we appreciate it. If not now, we can come back to pick up the sample at a later time.**<br>*INSTRUCTIONS IF UNABLE TO PRODUCE AT WILL*:<br>**For stool**: We will return tomorrow to pick up your stool. We would like the fresh stool you can give us. Please use one cup to collect the first stool you pass.	
**AG11** Stool collected?	No..00<br>yes 01
**AG12** Date stool sample taken (Ethiopian calendar)	Date: ______/_______/___________<br>Day / Month / Year
**AG13** Time when stool passed by the respondent (as recorded on cup) (Ethiopian time)	___ ___ : ___ ___<br>Hour Minute
**AG14** Time when stool collected from the respondent (Ethiopian time)	___ ___ : ___ ___

For peer review only - http://bmjopen.bmj.com/site/about/guidelines.xhtml

	Hour Minute
**AG15** TIME BLOOD centrifuged (Ethiopian time)	___ ___ : ___ ___<br>Hour Minute

OBSERVATIONS
TO BE FILLED IN AFTER COMPLETING INTERVIEW

COMMENTS:
____
____
____

WOMEN OF REPRODUCTIVE AGE 15-49 YEAR OLDS
ETHIOPIAN FOOD AND NUTRITION STRATEGY BASELINE SURVEY 2020/21
Biochemical and Health Related Data Collection Tool

IDENTIFICATION	
**HH00.** CLUSTER (EA) NAME.................................	**HH01**. CLUSTER NUMBER: ☐☐☐
**HH02**. HH NUMBER: ☐☐	**HH03**. RESIDENCE (RURAL=1, URBAN=2): ☐☐
**HH04**. RESPONDENT LINE NUMBER: (SHOULD BE MOTHER/CAREGIVER) ☐☐	**HH05** WOMEN LINE NUMBER ☐☐
HH06. INTERVIEWER NAME___________________<br>CODE:_____________________	HH07. TEAM LEADER, NAME:______________<br>CODE:_____________________
HH08. SUPERVISOR NAME:____________________<br>CODE._____________________	

PART I: HEALTH RELATED QUESTIONS

**S.N**	**QUESTION**	**Response**	**SKIP**
1	How old are you?<br>(*verify that the age is the same age as written on the household listing*)	☐☐<br>Years	
Now I would like to ask you some questions about your health. I will first ask you about the last 6 months.			
2	Have you been diagnosed with anemia in the past six months?	No...........................0	

For peer review only - http://bmjopen.bmj.com/site/about/guidelines.xhtml

		Yes ……………………1<br>Don't know…………98	
2	Have you been ill with malaria in the past 2 weeks?	No…………………………0<br><br>Yes ……………………1<br>Don't know…………98	
3	Do you smoke? (do not include the powder and chew type)	No………………..0<br><br>Yes ……………..1	
4	Are you currently lactating?	No………………..0<br><br>Yes ……………..1	
5	During that last pregnancy (that resulted in a live birth) did you have difficulty with your vision at night ("Dafent" night blindness in local language)?	No…………………………0<br><br>Yes ……………………1<br>Don't know…………98	
6	In the first two months after delivery, did you receive a<br><br>vitamin A dose (like this)?<br><br>*SHOW THE CAPSULE*	No…………………………0<br><br>Yes ………………..1<br>Don't know…………98	

PART II: WOMEN BIOCHEMICAL MEASUREMENT

**If the women is pregnant do not collect venous blood**	
Consent given for:<br>0= No or 1= Yes	**WL01** Blood ☐ **WL02** Urine ☐ **WL03** Stool ☐
**WL4** BLUE TOP TUBE (METAL FREE)<br>**Did not work =00.0**<br>**Refused = 77.7**<br>**Pregnant = 99.9**	ML. ☐☐ • ☐
**WL5** PURPLE TOP TUBE (EDTA)<br>**Did not work =00.0**<br>**Refused = 77.7**<br>**Pregnant = 99.9**	ML. ☐☐ • ☐
**WL6** REDTOP TUBE (EDTA)<br>**Did not work =00.0**<br>**Refused = 77.7**<br>**Pregnant = 99.9**	ML. ☐☐ • ☐
**WL7** Date blood sample taken (Ethiopian calendar)	Date: ______/_______/___________

For peer review only - http://bmjopen.bmj.com/site/about/guidelines.xhtml

	Day / Month / Year
**WL8** TIME BLOOD DRAW (Ethiopian time)	Blood draw ___ ___ : ___ ___<br>Hour Minute
**WL9 When did you eat your most recent meal (food)?** (Ethiopian date and time)	______/_______/__________ __ ___ : ___ ___<br>Date /Month/ Year Hour Minute
**WL 10 Finger prick or venous sample taken?**	01 Finger prick<br>02 Venous
**WL11** MALARIA RESULTS (RDT)	NEGATIVE.. 00<br>POSITIVE P *falciparum* .... 01<br>POSITIVE P *vivax* .... 02<br>POSITIVE FOR BOTH P *falciparum and* P *vivax* .... *03*<br>INVALID 04
**WL12** HEMOGLOBIN RESULTS	g/dL [ ][ ].[ ]
**In order to determine if you have blood in the urine or worms in the stool we would like to collect a urine and stool sample. If you can provide this now, we appreciate it. If not now, we can come back to pick up the sample at a later time.**<br>*INSTRUCTIONS IF UNABLE TO PRODUCE AT WILL*:<br>**For stool**: We will return tomorrow to pick up your stool. We would like the fresh stool you can give us. Please use one cup to collect the first stool you pass.<br>**For urine**: We will return tomorrow to pick up your urine.	
**WL13 U**rine collected?	No..0<br>0 yes<br>...01
**WL14** RESULTS (blood in urine)<br>**Ask the women if she is Menstruating**<br>(Don't test if the women is in Menstruation)	Negative..00<br>positive ....01<br>Women is Menstruating.............................03
**WL15** Stool collected?	No...00<br>yes 01
**WL16** Date stool sample taken (Ethiopian calendar)	Date: ______/_______/__________<br>Day / Month / Year
**WL17** Time when stool passed by the respondent (as recorded on cup) (Ethiopian time)	___ ___ : ___ ___<br>Hour Minute
**WL18** Time when stool collected from the respondent (Ethiopian time)	___ ___ : ___ ___

For peer review only - http://bmjopen.bmj.com/site/about/guidelines.xhtml

	Hour Minute
**WL19** TIME BLOOD centrifuged (Ethiopian time)	___ ___ : ___ ___<br>Hour Minute

OBSERVATIONS

TO BE FILLED IN AFTER COMPLETING INTERVIEW

COMMENTS:

____
____
____
____
____

For peer review only

Supplementary consent 1

1. Informed Consent Form for Household Head

Ethiopian Food and Nutrition Strategy (FNS) Baseline Survey

Investigator(s): Dr.Masresha Tessema (PI), Meseret W/Yohannes, Dr. Meron Girma, Alemnesh Petros, Dr Aregash Samuel, Arnaud Laillou, Stanley Chitekwe, Kaleab Baye, Ramadhani Noor, Anne Sophie Donze and other co-authors

You are being invited to take part in this research because you are head of household. There are about [16596] households taking part in this research. We will ask you about household characteristics, and socio-economic status of your household. We will also assess dietary intake, anthropometric status, nutrition sensitive and specific indicators and micronutrient status of your household member.

Box 1. Taking part in this research is voluntary

☐You **can refuse** to take part in this study.

☐You **can withdraw** your participation from the study at any time

Information related to the study

The FNS baseline survey will be conducted in the 12 regions of Ethiopia. The study population will be children age 0-59 months having caregivers/mothers, school-age children (6-12 years), adolescent girls (10-19 years), reproductive-age women (15-49 years), pregnant and lactating women, and household head. The indicators that will be collected for the survey will be dietary intake, anthropometric status, nutrition sensitive and specific indicators and micronutrient status.

The expected possible adverse effects: there is no adverse effect by participating in this study

The objective of this research: to produce information on anthropometric status, dietary intakes, and micronutrient status of different population groups in Ethiopia, and assess the coverage of direct and indirect nutrition interventions.

**Study design:** A population-based, cross-sectional design	
**The schedule of the study:** The study will be conducted from July, 2021 to April, 2023	
**Foreseeable risks and expected benefits arising from participation in the study**	
**Foreseeable risks**	**Expected benefits**
Risks to study participants for involvement in the coverage survey are low. There may be risks associated	The findings of the study will help the ministry of health and other stakeholders engaged in nutrition

For peer review only

with COVID pandemic. Interviewers will be trained to minimize this risk and will use appropriate prevention measures.	to improve and/or design appropriate health and nutrition intervention programs in the country.

Occurrences that may take place during the study period

**Occurrences**	**How to manage**
Withdrawal of volunteers from the study	In such a case, we would respect the volunteer's decision to withdraw and also get a clear understanding of the reason for their withdrawal

At the end of the study, you will not be receiving any financial benefits, but will get your results for height, weight, mid upper arm and waist circumference measurements, anemia and goiter status for time you spent and participation.

All data collected from the study will be kept confidential. If you have any questions related to the study you may contact directly Dr. Masresha Tessema who is the project PI.

The contact persons

1. Dr. Masresha Tessema Tel. [+251 919782082] E-mail: [masresha88@gmail.com] or
2. [Mr. Ibrahim Kedir] Tel. [+251 911957161] EPHI's IRB

**Certificate of Consent**	
I have read the foregoing information. I have an opportunity to ask questions and all my quest have been answered to my satisfaction. I volunteer to give consent to participate in this research study<br><br>_________________________<br>Printed name of the participant<br><br>________________________<br>Signature of the participant<br>Date ________________<br>day/month/year	I confirm that the participant was given an opportunity to ask questions about the study and all questions have been answered correctly. I confirm that the consent has been given voluntarily<br><br>_______________________________<br>Printed name of the person taking the consent<br><br>______________________<br>Signature of the person taking the consent<br>Date___________________<br>day/month/year

For peer review only

2. Informed Consent Form for Women of Reproductive Age

Ethiopian Food and Nutrition Strategy (FNS) Baseline Survey

Investigator(s): Dr.Masresha Tessema (PI), Meseret W/Yohannes, Dr. Meron Girma, Alemnesh Petros, Dr Aregash Samuel, Arnaud Laillou, Stanley Chitekwe, Kaleab Baye, Ramadhani Noor, Anne Sophie Donze and other co-authors

You are being invited to take part in this research because you are women of reproductive age. There are [16596] households taking part in this research. We will assess your dietary intake, anthropometric status, nutrition sensitive and specific indicators and micronutrient status

Box 1. Taking part in this research is voluntary

- You **can refuse** to take part in this study.
- You **can withdraw** your participation from the study at any time

Information related to the study

The FNS baseline survey will be conducted in the 12 regions of Ethiopia. The study population will be children age 0-59 months having caregivers/mothers, school-age children (6-12 years), adolescent girls (10-19 years), reproductive-age women (15-49 years), pregnant and lactating women, and household head. The indicators that will be collected for the survey will be dietary intake, anthropometric status, nutrition sensitive and specific indicators and micronutrient status.

The expected possible adverse effects: there is no adverse effect by participating in this study

The objective of this research: to produce information on anthropometric status, dietary intakes, and micronutrient status of different population groups in Ethiopia, and assess the coverage of direct and indirect nutrition interventions.

Study design: A population-based, cross-sectional design

The schedule of the study: The study will be conducted from July, 2021 to April, 2023

Foreseeable risks and expected benefits arising from participation in the study

**Foreseeable risks**	**Expected benefits**
Risks to study participants for involvement in the coverage survey are low. There may be risks associated with COVID pandemic. Interviewers will be trained to minimize this risk and will use appropriate prevention measures.	The findings of the study will help the ministry of health and other stakeholders engaged in nutrition to improve and/or design appropriate health and nutrition intervention programs in the country.

For peer review only

Occurrences that may take place during the study period	
**Occurrences**	**How to manage**
Withdrawal of volunteers from the study	In such a case, we would respect the volunteer's decision to withdraw and also get a clear understanding of the reason for their withdrawal

At the end of the study, you will not be receiving any financial benefits, but will get your results for height, weight, mid upper arm and waist circumference measurements, anemia and goiter status for time you spent and participation.

All data collected from the study will be kept confidential. If you have any questions related to the study you may contact directly Dr. Masresha Tessema who is the project PI.

The contact persons

1. Dr. Masresha Tessema Tel. [+251 919782082] E-mail: [masresha88@gmail.com] or
2. [Mr. Ibrahim Kedir] Tel. [+251 911957161] EPHI's IRB

Certificate of Consent	
I have read the foregoing information. I have an opportunity to ask questions and all my quest have been answered to my satisfaction. I volunteer to give consent to participate in this research study	I confirm that the participant was given an opportunity to ask questions about the study and all questions have been answered correctly. I confirm that the consent has been given voluntarily
________________________<br>Printed name of the participant<br>________________________<br>Signature of the participant<br>Date ________________<br>day/month/year	______________________________<br>Printed name of the person taking the consent<br>______________________<br>Signature of the person taking the consent<br>Date___________________<br>day/month/year

For peer review only

3. Informed Consent Form for Pregnant Women

Ethiopian Food and Nutrition Strategy (FNS) Baseline Survey

Investigator(s): Dr.Masresha Tessema (PI), Meseret W/Yohannes, Dr. Meron Girma, Alemnesh Petros, Dr Aregash Samuel, Arnaud Laillou, Stanley Chitekwe, Kaleab Baye, Ramadhani Noor, Anne Sophie Donze and other co-authors

You are being invited to take part in this research because you are pregnant women. There are [16596] households taking part in this research. We will assess your, anthropometric status, nutrition sensitive and specific indicators and anemia status

Box 1. Taking part in this research is voluntary

- You **can refuse** to take part in this study.
- You **can withdraw** your participation from the study at any time

Information related to the study

The FNS baseline survey will be conducted in the 12 regions of Ethiopia. The study population will be children age 0-59 months having caregivers/mothers, school-age children (6-12 years), adolescent girls (10-19 years), reproductive-age women (15-49 years), pregnant and lactating women, and household head. The indicators that will be collected for the survey will be dietary intake, anthropometric status, nutrition sensitive and specific indicators and micronutrient status.

The expected possible adverse effects: There is no adverse effect by participating in this study

The objective of this research: to produce information on anthropometric status, dietary intakes, and micronutrient status of different population groups in Ethiopia, and assess the coverage of direct and indirect nutrition interventions.

Study design: a population-based, cross-sectional design

The schedule of the study: The study will be conducted from July, 2021 to April, 2023

**Foreseeable risks and expected benefits arising from participation in the study**	
**Foreseeable risks**	**Expected benefits**
Risks to study participants for involvement in the coverage survey are low. There may be risks associated with COVID pandemic. Interviewers will be trained to minimize this risk and will use appropriate prevention measures.	The findings of the study will help the ministry of health and other stakeholder engaged in nutrition to improve and/or design appropriate health and nutrition intervention programs in the country.

Occurrences that may take place during the study period

For peer review only - http://bmjopen.bmj.com/site/about/guidelines.xhtml

Occurrences	How to manage
Withdrawal of volunteers from the study	In such a case, we would respect the volunteer's decision to withdraw and also get a clear understanding of the reason for their withdrawal

At the end of the study, you will not be receiving any financial benefits, but will get your results for height, weight, mid upper arm circumference measurements, anemia and goiter status for time you spent and participation.

All data collected from the study will be kept confidential. If you have any questions related to the study you may contact directly Dr. Masresha Tessema who is the PI

The contact persons

1. Dr. Masresha Tessema

Tel. [+251 919782082] E-mail: [masresha88@gmail.com]

2. [Mr. Ibrahim Kedir] Tel. [+251 911957161]

Certificate of Consent	
I have read the foregoing information. I have an opportunity to ask questions and all my quest have been answered to my satisfaction. I volunteer to give consent to participate in this research study<br><br>________________________<br>Printed name of the participant<br>________________________<br>Signature of the participant<br>Date _______________<br>day/month/year	I confirm that the participant was given an opportunity to ask questions about the study and all questions have been answered correctly. I confirm that the consent has been given voluntarily<br><br>______________________________<br>Printed name of the person taking the consent<br>______________________<br>Signature of the person taking the consent Date<br>___________________<br>day/month/year

4. Informed Consent Form for Preschool Child

Ethiopian Food and Nutrition Strategy (FNS) Baseline Survey

Investigator(s): Dr.Masresha Tessema (PI), Meseret W/Yohannes, Dr. Meron Girma, Alemnesh Petros, Dr

For peer review only

Aregash Samuel, Arnaud Laillou, Stanley Chitekwe, Kaleab Baye, Ramadhani Noor, Anne Sophie Donze and other co-authors

You are being invited to take part in this research because you are either a mother or caregiver who has a child under the age of 5 years (0-59 months). There are [16596] households taking part in this research. We would collect a sample of your child's dietary information, blood, urine and stool. And, we will also measure your child's height/ length, weight, and mid upper arm circumference. Finally we will assess, your child's eye for bitot spot

**Box 1. Taking part in this research is voluntary**
☐You **can refuse** to take part in this study.
☐You **can withdraw** your participation from the study at any time

Information related to the study

The FNS baseline survey will be conducted in the 12 regions of Ethiopia. The study population will be children age 0-59 months having caregivers/mothers, school-age children 6-12 years), adolescent girls (10-19 years), reproductive-age women (15-49 years), pregnant and lactating women, and household head. The indicators that will be collected for the survey will be dietary intake, anthropometric status, nutrition sensitive and specific indicators and micronutrient status.

**The expected possible adverse effects :**there is no adverse effect by participating in this study

The objective of this research: to produce information on anthropometric status, dietary intakes, and micronutrient status of different population groups in Ethiopia, and assess the coverage of direct and indirect nutrition interventions.

**Study design:** a population-based, cross-sectional design	
**The schedule of the study:** The study will be conducted from July, 2021 to April, 2023	
**Foreseeable risks and expected benefits arising from participation in the study**	
**Foreseeable risks**	**Expected benefits**
Risks to study participants for involvement in the coverage survey are low. There may be risks associated with COVID pandemic. Interviewers will be trained to minimize this risk and will use appropriate prevention measures.	The findings of the study will help the ministry of health and other stakeholder engaged in nutrition to improve and/or design appropriate health and nutrition intervention programs in the country.
**Occurrences that may take place during the study period**	
**Occurrences**	**How to manage**

For peer review only

Withdrawal of volunteers from the study	in such a case, we would respect the volunteer's decision to withdraw and also get a clear understanding of the reason for their withdrawal

At the end of the study, you will not be receiving any financial benefits, but will get your results for height/length, weight, mid upper arm circumference measurements, and anemia for time you spent and participation.

All data collected from the study will be kept confidential. If you have any questions related to the study you may contact directly Dr. Masresha Tessema who is the project principal investigator

The contact persons

1. Dr. Masresha Tessema

Tel. [+251 919782082] E-mail: [masresha88@gmail.com]

2. [Mr. Ibrahim Kedir] Tel. [+251 911957161] EPHI's IRB

**Certificate of Consent**	
I have read the foregoing information. I have an opportunity to ask questions and all my quest have been answered to my satisfaction. I volunteer to give consent to participate in this research study<br>_________________________<br>Printed name of the participant<br>________________________<br>Signature of the participant's parent or guardian<br>Date ________________<br>day/month/year	I confirm that the participant was given an opportunity to ask questions about the study and all questions have been answered correctly. I confirm that the consent has been given voluntarily<br>________________________________<br>Printed name of the person taking the consent<br>_______________________<br>Signature of the person taking the consent Date<br>____________________<br>day/month/year

For peer review only

5. **Informed Consent Form for School Age Children**

**Ethiopian Food and Nutrition Strategy (FNS) Baseline Survey**
**Investigator(s):** Dr.Masresha Tessema (PI), Meseret W/Yohannes, Dr. Meron Girma, Alemnesh Petros, Dr Aregash Samuel, Arnaud Laillou, Stanley Chitekwe, Kaleab Baye, Ramadhani Noor, Anne Sophie Donze and other co-authors

You are being invited to take part in this research because you are either a mother or caregiver who has a school-age child. Among children 6 – 12 years, we will collect your child's blood, urine and stool.

**Box 1. Taking part in this research is voluntary**
☐You **can refuse** to take part in this study.
☐You **can withdraw** your participation from the study at any time

Information related to the study

The FNS baseline survey will be conducted in the 12 regions of Ethiopia. The study population will be children age 0-59 months having caregivers/mothers, school-age children (6-12 years), adolescent girls (10-19 years), reproductive-age women (15-49 years), pregnant and lactating women, household head. The indicators that will be collected for the survey will be dietary intake, anthropometric status, nutrition sensitive and specific indicators and micronutrient status.

**The expected possible adverse effects:** there is no adverse effect by participating in this study

The objective of this research: to produce information on anthropometric status, dietary intakes, and micronutrient status of different population groups in Ethiopia, and assess the coverage of direct and indirect nutrition interventions.

**Study design:** a population-based, cross-sectional design	
**The schedule of the study:** The study will be conducted from July, 2021 to April, 2023	
**Foreseeable risks and expected benefits arising from participation in the study**	
**Foreseeable risks**	**Expected benefits**
Risks to study participants for involvement in the coverage survey are low. There may be risks associated with COVID pandemic. Interviewers will be trained to minimize this risk and will use appropriate prevention measures.	The findings of the study will help the ministry of health and other stakeholder engaged in nutrition to improve and/or design appropriate health and nutrition intervention programs in the country.
**Occurrences that may take place during the study period**	
**Occurrences**	**How to manage**

For peer review only

Withdrawal of volunteers from the study	In such a case, we would respect the volunteer's decision to withdraw and also get a clear understanding of the reason for their withdrawal

At the end of the study, you will not be receiving any financial benefits, but you will get your **anemia** and **goiter** status for time you spent and participation.

All data collected from the study will be kept confidential. If you have any questions related to the study you may contact directly Dr. Masresha Tessema who is the project's PI

The contact persons

1. Dr. Masresha Tessema

Tel. [+251 919782082] E-mail: [masresha88@gmail.com]

2. [Mr. Ibrahim Kedir] Tel. [+251 911957161] EPHI's IRB

Certificate of Consent	
I have read the foregoing information. I have an opportunity to ask questions and all my quest have been answered to my satisfaction. I volunteer to give consent to participate in this research study<br><br>________________________<br>Printed name of the participant<br><br>_______________________<br>Signature of the participant's parent or guardian<br><br>Date _______________<br>day/month/year	I confirm that the participant was given an opportunity to ask questions about the study and all questions have been answered correctly. I confirm that the consent has been given voluntarily<br><br>______________________________<br>Printed name of the person taking the consent<br><br>______________________<br>Signature of the person taking the consent Date<br><br>___________________<br>day/month/year

For peer review only

6. Assent form for Adolescent Girls (10-19 years)

Ethiopian Food and Nutrition Strategy (FNS) Baseline Survey

Investigator(s): Dr.Masresha Tessema (PI), Meseret W/Yohannes, Dr. Meron Girma, Alemnesh Petros, Dr Aregash Samuel, Arnaud Laillou, Stanley Chitekwe, Kaleab Baye, Ramadhani Noor, Anne Sophie Donze and other co-authors

You are being invited to take part in this research because you are Adolescent girl. There are [16596] households taking part in this research. We will measure your dietary information (for those adolescent girls aged 15-17 years), information related to nutrition -sensitive and nutrition-specific practices, blood, and stool, we will also measure your height, weight, and mid upper arm and waist circumference and your goiter status

Box 1. Taking part in this research is voluntary

☐You **can refuse** to take part in this study.

☐You **can withdraw** your participation from the study at any time

Information related to the study

The FNS baseline survey will be conducted in the 12 regions of Ethiopia. The study population will be children age 0-59 months having caregivers/mothers, school-age children (6-12 years), adolescent girls (10-19 years), reproductive-age women (15-49 years), pregnant and lactating women, and household head. The indicators that will be collected for the survey will be dietary intake, anthropometric status, nutrition sensitive and specific indicators and micronutrient status

The expected possible adverse effects: There is no adverse effect by participating in this study

The objective of this research: to produce information on anthropometric status, dietary intakes, and micronutrient status of different population groups in Ethiopia, and assess the coverage of direct and indirect nutrition interventions.

Study design: a population-based, cross-sectional design

The schedule of the study: The study will be conducted from July, 2021 to April, 2023

Foreseeable risks and expected benefits arising from participation in the study

Foreseeable risks	Expected benefits
Risks to study participants for involvement in the coverage survey are low. There may be risks associated with COVID pandemic. Interviewers will be trained to minimize this risk and will use appropriate prevention measures.	The findings of the study will help the ministry of health and other stakeholder engaged in nutrition to improve and/or design appropriate health and nutrition intervention programs in the country.

For peer review only

Occurrences that may take place during the study period	
**Occurrences**	**How to manage**
Withdrawal of volunteers from the study	In such a case, we would respect the volunteer's decision to withdraw and also get a clear understanding of the reason for their withdrawal

At the end of the study, you will not be receiving any financial benefits, but will get your results for height, weight, mid upper arm and waist circumference measurements, anemia and goiter status for time you spent and participation.

All data collected from the study will be kept confidential. If you have any questions related to the study you may contact directly Dr. Masresha Tessema who is the project's PI

The contact persons

1. Dr. Masresha Tessema

Tel. [+251 919782082] E-mail: [masresha88@gmail.com]

2. [Mr. Ibrahim Kedir] Tel. [+251 911957161] EPHI's IRB

Certificate of Assent	
I have read the foregoing information. I have an opportunity to ask questions and all my quest have been answered to my satisfaction. I volunteer to give assent to participate in this research study<br><br>________________________<br>Printed name of the participant<br><br>_______________________<br>Signature of the participant<br>Date ________________<br>day/month/year	I confirm that the participant was given an opportunity to ask questions about the study and all questions have been answered correctly. I confirm that the assent has been given voluntarily<br><br>______________________________<br>Printed name of the person taking the assent<br><br>______________________<br>Signature of the person taking the assent Date<br><br>___________________<br>day/month/year

For peer review only

STROBE Statement—checklist of items that should be included in reports of observational studies

	Item No	Recommendation	Reported on page #
**Title and abstract**	1	(*a*) Indicate the study's design with a commonly used term in the title or the abstract	Title p.1; Abstract p.2-3
		(*b*) Provide in the abstract an informative and balanced summary of what was done and what was found	
**Introduction**			
**Introduction** /rationale	4	Explain the scientific background and rationale for the investigation being reported	**Introduction** p.3-4
Objectives	5	State specific objectives, including any prespecified hypotheses	Background p.5 last statements
**Methods**			
Study design	5	Present key elements of study design early in the paper	Methods (data source) p.4
Setting	5	Describe the setting, locations, and relevant dates, including periods of recruitment, exposure, follow-up, and data collection	Methods (data source) p.5
Participants	5	(*a*) *Cohort study*—Give the eligibility criteria, and the sources and methods of selection of participants. Describe methods of follow-up<br>*Case-control study*—Give the eligibility criteria, and the sources and methods of case ascertainment and control selection. Give the rationale for the choice of cases and controls<br>*Cross-sectional study*—Give the eligibility criteria, and the sources and methods of selection of participants	Methods (participants) p.5-8
		(*b*) *Cohort study*—For matched studies, give matching criteria and number of exposed and unexposed<br>*Case-control study*—For matched studies, give matching criteria and the number of controls per case	
Variables	5	Clearly define all outcomes, exposures, predictors, potential confounders, and effect modifiers. Give diagnostic criteria, if applicable	Methods (all outcome and exposure variables are listed) p.6-8
Data sources/ measurement	NA	For each variable of interest, give sources of data and details of methods of assessment (measurement). Describe comparability of assessment methods if there is more than one group	Methods (methods of measurement indicated in) p.5-8
Bias	10	Describe any efforts to address potential sources of bias	Methods (data quality indicated) p.10-11
Study size	5-6	Explain how the study size was arrived at	Methods (data source,statistical

For peer review only

			analysis) p.5
Quantitative variables	10	Explain how quantitative variables were handled in the analyses. If applicable, describe which groupings were chosen and why	Methods (measurement and statistical analysis sections) p. 8-9
Statistical methods	10	(*a*) Describe all statistical methods, including those used to control for confounding	Methods (analysis section) p. 8-9
		(*b*) Describe any methods used to examine subgroups and interactions	
		(*c*) Explain how missing data were addressed	
		(*d*) *Cohort study*—If applicable, explain how loss to follow-up was addressed<br>*Case-control study*—If applicable, explain how matching of cases and controls was addressed<br>*Cross-sectional study*—If applicable, describe analytical methods taking account of sampling strategy	
		(*e*) Describe any sensitivity analyses	

Continued on next page

For peer review only

**Results**			
Participants	5*	(a) Report numbers of individuals at each stage of study—eg numbers potentially eligible, examined for eligibility, confirmed eligible, included in the study, completing follow-up, and analysed	NA
		(b) Give reasons for non-participation at each stage	
		(c) Consider use of a flow diagram	
Descriptive data	14*	(a) Give characteristics of study participants (eg demographic, clinical, social) and information on exposures and potential confounders	NA
		(b) Indicate number of participants with missing data for each variable of interest	
		(c) *Cohort study*—Summarise follow-up time (eg, average and total amount)	
Outcome data	15*	*Cohort study*—Report numbers of outcome events or summary measures over time	NA
		Case-control study—Report numbers in each exposure category, or summary measures of exposure	
		Cross-sectional study—Report numbers of outcome events or summary measures	
Main results	16	(*a*) Give unadjusted estimates and, if applicable, confounder-adjusted estimates and their precision (eg, 95% confidence interval). Make clear which confounders were adjusted for and why they were included	NA
		(*b*) Report category boundaries when continuous variables were categorized	
		(*c*) If relevant, consider translating estimates of relative risk into absolute risk for a meaningful time period	
Other analyses	17	Report other analyses done—eg analyses of subgroups and interactions, and sensitivity analyses	NA
**Discussion**			
Key results	18	Summarise key results with reference to study objectives	p. 9-10
Limitations	19	Discuss limitations of the study, taking into account sources of potential bias or imprecision. Discuss both direction and magnitude of any potential bias	Discussion (strengths and weaknesses of the study) p.10-11
Interpretation	20	Give a cautious overall interpretation of results considering objectives, limitations, multiplicity of analyses, results from similar studies, and other relevant evidence	Discussion (interpretation of findings in the context of existing research, meaning of the study:

For peer review only - http://bmjopen.bmj.com/site/about/guidelines.xhtml

			p.9-11 implication
Generalisability	21	Discuss the generalisability (external validity) of the study results	Discussion (strengths and weakness of the study) p.10-11
**Other information**			
Funding	22	Give the source of funding and the role of the funders for the present study and, if applicable, for the original study on which the present article is based	Funding p.12

*Give information separately for cases and controls in case-control studies and, if applicable, for exposed and unexposed groups in cohort and cross-sectional studies.

Note: An Explanation and Elaboration article discusses each checklist item and gives methodological background and published examples of transparent reporting. The STROBE checklist is best used in conjunction with this article (freely available on the Web sites of PLoS Medicine at http://www.plosmedicine.org/, Annals of Internal Medicine at http://www.annals.org/, and Epidemiology at http://www.epidem.com/). Information on the STROBE Initiative is available at www.strobe-statement.org.

For peer review only
